# Genomic Insights into Drug Resistance and Virulence Platforms, CRISPR-Cas Systems and Phylogeny of Commensal *E. coli* from Wildlife

**DOI:** 10.3390/microorganisms9050999

**Published:** 2021-05-05

**Authors:** Carla Andrea Alonso, María de Toro, Fernando de la Cruz, Carmen Torres

**Affiliations:** 1 Área de Bioquímica y Biología Molecular, Universidad de La Rioja, 26006 Logroño, Spain; caalonso@riojasalud.es; 2 Servicio de Microbiología, Hospital San Pedro, 26006 Logroño, Spain; 3 Plataforma de Genómica y Bioinformática, Centro de Investigación Biomédica de La Rioja (CIBIR), 26006 Logroño, Spain; mthernando@riojasalud.es; 4 Departamento de Biología Molecular, Universidad de Cantabria and Instituto de Biomedicina y Biotecnología de Cantabria (Universidad de Cantabria-CSIC), 39011 Santander, Spain; fernando.cruz@unican.es

**Keywords:** *E. coli*, CRISPR-Cas, antimicrobial resistance, wild animals, WGS, PLACNETw

## Abstract

Commensal bacteria act as important reservoirs of virulence and resistance genes. However, existing data are generally only focused on the analysis of human or human-related bacterial populations. There is a lack of genomic studies regarding commensal bacteria from hosts less exposed to antibiotics and other selective forces due to human activities, such as wildlife. In the present study, the genomes of thirty-eight *E. coli* strains from the gut of various wild animals were sequenced. The analysis of their *accessory* genome yielded a better understanding of the role of the mobilome on inter-bacterial dissemination of mosaic virulence and resistance plasmids. The study of the presence and composition of the CRISPR/Cas systems in *E. coli* from wild animals showed some viral and plasmid sequences among the spacers, as well as the relationship between CRISPR/Cas and *E. coli* phylogeny. Further, we constructed a single nucleotide polymorphisms-based *core* tree with *E. coli* strains from different sources (humans, livestock, food and extraintestinal environments). Bacteria from humans or highly human-influenced settings exhibit similar genetic patterns in CRISPR-Cas systems, plasmids or virulence/resistance genes-carrying modules. These observations, together with the absence of significant genetic changes in their *core* genome, suggest an ongoing flow of both mobile elements and *E. coli* lineages between human and natural ecosystems.

## 1. Introduction

*Escherichia coli* is a well-known commensal of the gut of humans and a wide-range of other animals, but it can also reproduce and persist for long periods of time in extraintestinal natural environments. Beside these habitats, some strains of *E. coli* have the potential to cause severe intestinal and extraintestinal illness, such as meningitis, septicemia, pneumonia or urinary tract infections. This diversity in terms of niche distribution and host–pathogen interactions is due to the high plasticity of the *E. coli* genome, which allows the bacteria to adapt to the varying selective pressures exerted by the different environments. All this, plus the easy and fast growth characteristics of *E. coli*, makes this species an excellent model to study the evolution and epidemiology of antimicrobial resistance or bacterial virulence [1].

The dynamics of bacterial genomes involve both single mutations in the *core* genome (microevolution) as well as horizontal gene transfer events (macroevolution) that shape a species pangenome [2]. Both types of evolutionary phenomena have influenced the acquisition of new phenotypes and host/niche adaptation. Horizontal gene transfer (HGT) is largely mediated by mobile genetic elements (MGEs) such as insertion sequences (ISs), transposons, integrons, prophages, integrative conjugative elements, genomic islands and plasmids. Human activities promote the use of selective agents (antibiotics, disinfectants, heavy metals) that induce a bacterial SOS response and, thus, increase HGT rates [3]. This leads to the assembly of mosaic genetic elements and the development of antimicrobial and/or virulence platforms of increasing complexity. Besides the selective pressure exerted by chemicals, the genetic background of bacteria also seems to play a role in the antimicrobial resistance phenotype. As an example, the strong correlation observed between *E. coli* phylogroup B2 and antimicrobial susceptibility patterns [4]. 

Classically, PCR-based genotypic tests and other traditional typing methods (serotyping, multilocus enzyme electrophoresis, pulsed-field gel electrophoresis, multilocus sequence typing and phylogrouping multiplex PCR) were used to ascertain the genetic relatedness and transmission dynamics of bacteria [5]. The advent of whole genome sequencing (WGS) technology provides a finer-scale analysis tool that enables the high resolution detection of antimicrobial resistance and virulence determinants, the analysis of genome structural variations and genetic linkages or the identification of potential adaptations of the bacterial genomes to the host conditions, among others. Since most genome sequencing projects focus on the analysis of multi-drug resistant clones, clinically relevant pathogens or epidemiological outbreaks, there is a lack of genomic data on commensal *E. coli* populations, even more so in those non-directly associated to humans. The study of randomly selected microbial communities, less exposed to antibiotics and other selective forces associated with the human environment, could provide new insights into the evolution of *E. coli* genomes and the flow of clinically relevant genes among different ecosystems. 

In the present work, a collection of 38 *E. coli* genomes from the gut of various wild animals were sequenced. Analysis of their *accessory* genome yielded a better understanding of the role of the mobilome on inter-bacterial dissemination of mosaic virulence and resistance platforms. The study of the presence and composition of the CRISPR/Cas systems in *E. coli* from wild animals showed some phage- and plasmid-related sequences among the spacers, as well as the relationship between CRISPR/Cas and *E. coli* phylogeny. Further, we constructed a single nucleotide polymorphism (SNP)-based *core* tree with *E. coli* strains from different origins (humans, livestock, food and extraintestinal environments) in order to elucidate whether the commensal population of wild animals is subjected to a parallel independent microevolution or, on the contrary, an on-going inter-host transmission is more likely occurring. 

## 2. Materials and Methods

### 2.1. Bacterial Collection, Antibiotic Susceptibility Testing and Phylotyping

In this work, 38 *E. coli* strains randomly selected among those previously isolated and identified as part of other studies [6,7,8,9] were subjected to sequencing. The basic genomic features of these 38 genomes are included in Supplementary Materials Table S1. The strains were isolated from fecal specimens (or intestine segments with fecal content in the case of wild boars) collected between 2013 and 2015 in various geographic locations of Spain (Castilla-La Mancha, Cádiz, Aragón) from healthy wild animals as previously described [6,7,8,9]. Eleven strains were isolated from red deer (*Cervus elaphus*), 11 from wild boars (*Sus scrofa*), 9 from small rodents ((mice, *n* = 8 (*Apodemus sylvaticus* and *Mus musculus*); rats, *n* = 1 (*Rattus rattus*)), and 7 from birds of prey (eagles, *n* = 2 (*Aquila chrysaetos* and *Circaetus gallicus*); vultures, *n* = 4 (*Gyps fulvus*); osprey, *n* = 1 (*Gypaetus barbatus*)). Intestine segments with fecal content were collected from wild boars legally hunted in their own habitat during the regular hunting season (October to February 2014–2015) approved by the Consejería de Agricultura of Castilla-La Mancha [8]; no approval was needed from an ethical committee since the sacrifice of animals was not performed for research purposes, following the Spanish Policy for Animal Protection RD53/2013 and the European Union Directive 2010/63.

All isolates were tested for antimicrobial susceptibility with ampicillin, amoxicillin/clavulanate, ceftazidime, ceftriaxone, cefoxitin, imipenem, nalidixic acid, ciprofloxacin, gentamicin, amikacin, tobramycin, streptomycin, chloramphenicol, sulfonamides, trimethoprim/sulfamethoxazole and tetracycline, using the CLSI disk-diffusion method. Identification of the *E. coli* phylogroups was analyzed by a multiplex PCR-based assay [10], followed by in silico validation. 

For comparative phylogenomic analysis, another 242 *E. coli* full genomes were taken from the NCBI database. These belonged to bacteria from different sources: humans, livestock, wild animals and the environment. Information about all these genomes is included in Supplementary Materials Table S2. 

### 2.2. Sequencing of the E. coli Genomes

Genomic DNA from *E. coli* strains was extracted using the QIAmp DNA Mini Kit (Qiagen, Hilden, Germany). Subsequently, libraries were prepared with the TruSeq DNA PCR-free Sample Preparation Kit (Illumina, San Diego, CA, USA) and 100 bp paired-end reads were sequenced in a HiSeq 1500 System (Health in Code Facility). Illumina reads were quality checked with FastQC software (https://www.bioinformatics.babraham.ac.uk/projects/fastqc/) and trimmed to optimize their quality with TrimGalore (https://www.bioinformatics.babraham.ac.uk/projects/trim_galore).

### 2.3. Genome Analysis

Clean reads were uploaded to the PLACNETw plasmid reconstruction tool [11] in order to assemble and to elucidate genome components. After manual pruning based on scaffold link and coverage analysis as well as reference sequences comparisons, chromosomal and plasmid sequences were identified, clustered and downloaded in separated files. Besides genome reconstruction, PLACNETw enabled the classification of the plasmids by identification of relaxases, plasmid replication initiator proteins and incompatibility groups (Inc).

*E. coli* MLST profiles were predicted in silico using the Achtman 7 gene scheme at EnteroBase (http://enterobase.warwick.ac.uk (accessed on 18 December 2017)). The genomes of six strains showing novel MLST alleles or allele combinations were uploaded to the database for sequence type (ST) assignment. ResFinder 3.1 and VirulenceFinder 2.0 (90% identity and 60% minimum length) were used to estimate the antimicrobial resistance and virulence gene content, respectively [12,13]. To determine the presence of CRISPR/Cas systems and the number and sequences of spacers, the CRISPRfinder program was employed, with manual validation [14]. Spacer homologues were identified by BLASTn search against the nucleotide collection (nr/nt) database (query coverage 100%, identity ≥88%), excluding matches to other clustered, regularly interspaced, short palindromic repeats (CRISPR) sequences. 

Whenever necessary, contigs were manually assembled, curated and annotated using additional bioinformatic tools as: Artemis and SnapGene softwares, BLAST (www.ncbi.nlm.nih.gov/BLAST), ORFfinder (www.ncbi.nlm.nih.gov/projects/gorf), ISfinder (www-is.biotoul.fr) and UniProt database (http://www.uniprot.org (accessed on 22 December 2017)). To visualize resistance and virulence gene comparisons, EasyFig 2.1 was used [15]. 

Phylogenetic analysis of the *cas* set of genes or specific virulence determinants were carried out using MEGA7 software [16] from alignments generated with CLUSTALW. Concatenated amino acid or nucleotide sequences trees were constructed using the UPGMA method, with distances calculated by the Poisson correction or maximum composite likelihood, respectively, on a pairwise-deletion comparison. 

For the phylogenomic study, the *core* genome was defined as the assembly of genes present in all the genomes analyzed, with more than 80% similarity and 60% coverage. Genes were clustered with CD-HIT-EST [17], selecting those that were present at least in one copy per genome. This subset was concatenated and aligned with Muscle (v3.8.31) (http://www.drive5.com/muscle). SNPs were extracted and visualized in a SNP-matrix with HarvestTools (v1.0) (https://harvest.readthedocs.io/en/latest/content/harvest-tools.html). Finally, RAxML [18] was used to build the core genome phylogenetic tree, by using 100 replicates for bootstrap determination.

### 2.4. Nucleotide Sequence Accession Numbers

Raw sequence reads reported in this paper were deposited under NCBI BioProject PRJNA699864. 

## 3. Results and Discussion

Our analysis of the 38 genomes of *E. coli* isolated from wild animals focused on their CRISPR/Cas systems (Section 3.1), plasmidome and resistome (Section 3.2), virulome (Section 3.3) and phylogenomics (Section 3.4). 

### 3.1. CRISPR/Cas Systems

The discovery of CRISPRs was reported in the late 1980s in *Bacteria* and, a few years later, in *Archaea* [19,20]. CRISPRs are direct repeats of around 21–47 bp length separated by other short sequences named *spacers*, which share homology with phage- and plasmid-related segments. Frequently, CRISPR loci are found associated with genes encoding nucleases, the *cas* genes. Although different biological roles have been proposed, various functional assays demonstrated that CRISPR/Cas acts as an adaptive and heritable immunity system able to target and neutralize exogenous DNA through a pathway similar to RNA interference (RNAi) in eukaryotes [21]. Two monophyletic CRISPR/Cas systems have been reported in *E. coli*: I-E and I-F1. Subtype I-E consists of two CRISPR arrays (CRISPR-1, CRISPR-2) and a set of eight *cas* genes, while subtype I-F1 has six *cas* genes flanked by CRISPR-3 and CRISPR-4 arrays [22]. In contrast to the I-E system, which seems to be inactive due to H-NS regulation under laboratory conditions [23], the I-F system was demonstrated to be constitutively expressed, interfering targeted foreign elements [24,25].

We examined the CRISPR/Cas and spacer repertoire in our collection of 38 *E. coli* from wildlife. CRISPR/Cas I-E and I-F1 systems were detected in 28 (73.4%) and four genomes (10.5%), respectively. As commonly occurs, none carried both I-E and I-F1 subtypes simultaneously. 

**Figure 1:**
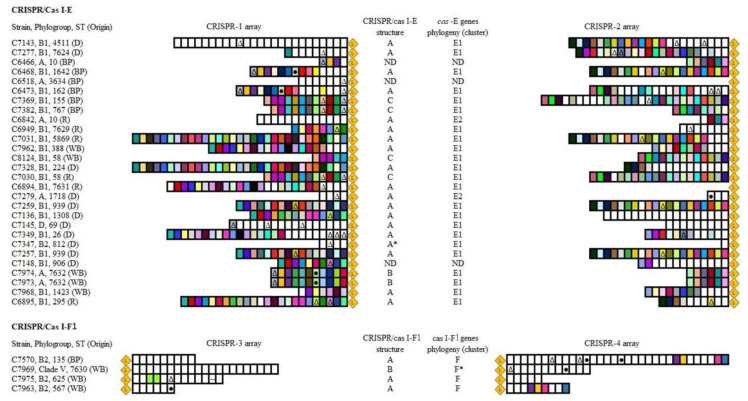
Scheme of the CRISPR arrays located in CRISPR/Cas I-E and I-F1 systems found in the genome of our *E. coli* collection. Spacers are represented as vertical boxes and are oriented with respect to the leader (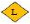). Boxes with the same color represent spacers that appear in ≥2 strains. There were no overlapping spacer sequences between CRISPR 1, 2, 3 and 4 arrays, thus colors must be independently interpreted for each array. The different symbols indicate spacers matching at least 88% with phages (∆), plasmids (●) or IS (~)-like sequences in databases. Origin: deer (D); bird of prey (BP); rodent (R); wild boar (WB). CRISPR/cas I-E (A, A *, B, C) and I-F1 structures (A, B) as well as *cas* genes clusters (E1, E2, F, F*) correspond to those represented in Supplementary Materials Figures S1 and S2, respectively. ND: non-determined.

Supplementary Materials Figure S1 shows the different genetic organizations for I-E and I-F1 systems regarding the module located between the two CRISPR arrays, comprising the *cas* set of genes and adjacent ORFs. The *cas* genes nomenclature follows the recommendations of Makarova et al., 2020 [26]. As other authors described, we found a higher diversity of structures in I-E systems, probably due to deletions and rearrangements promoted by IS elements [27]. In fact, a truncated IS*186*, which has been suggested to guide the CRISPR/Cas evolution owing to its affinity for GC-rich regions (very frequent in distinct points of the system), was found inserted within the *cas3* gene in C7973 and C7974 strains. Among the remaining *cas* genes, only *cas2* was present (variant B in Supplementary Materials Figure S1). Further, in the strain C7347 (phylogroup B2) carrying the CRISPR/Cas I-E a partial deletion of *cas7* and *cse1* genes and the absence of the complete copy of *cse2* was observed (variant A * in Supplementary Materials Figure S1). However, CRISPR 1 and 2 arrays were present, bearing low number of spacers (four and one, respectively), which probably reflect the non-functionality of the system and the progressive loss of spacers (Figure 1). 

In addition, the evolutionary relationship of *cas* genes inferred from the phylogenetic tree of the concatenated amino acid sequences of *cas*-E and *cas*-F1 genes showed, as previously reported [27], two clearly differentiated clusters in CRISPR/Cas I-E systems (E1 and E2) and a minor heterogeneity among CRISPR/Cas I-F1 (Supplementary Materials Figure S2). As it was described among ECOR collection [27], the majority of the *E. coli* strains from wildlife, belonging to a variety of phylogroups (A, B1, D, B2), were included in cluster E1, whereas cluster E2 comprised few strains of the A group. Although there was a limited number of strains, the *cas*-F1 set of genes exhibited lower genetic variability and, although the concatenated amino acid sequence differed by 8.6% between clade V and B2 strains (C7969 vs. C7570/C7975/C7963), they shared a common origin, in contrast to what it is proposed for E1 and E2 variants. Notably, the genetic sequence of *cas*-F1 cluster and adjacent genes in clade V strain suggests a close relation with *Escherichia marmotae*, a previously described species isolated from the feces of wild marmots in China [28].

Concerning the CRISPR arrays, there were a total of 404, 331, 48 and 58 spacers in CRISPR-1 to -4, respectively; 108, 63, 1 and 4 appeared in more than one isolate, regardless of the clonal relatedness of bacteria (Figure 1). The analyses of the spacers of CRISPR loci have been used in several bacterial species for strain typing. As shown in Figure 1 for the studied *E. coli* collection, a good correlation between CRISPR-1/-2 arrays and ST was observed, with some exceptions. We found strains sharing a large CRISPR-1 array that were ascribed to different ST, and even phylogroup (7031 and C7328). The coincidence in spacer segments between non-clonally related strains can be explained by recombination between homologous CRISPR arrays [27], vertical inheritance and differential spacer deletion [29] or a combination of both. Moreover, in agreement with Díez-Villaseñor et al., 2010 [30], we observed that CRISPR-3/-4 arrays contained a larger proportion of unique spacers than CRISPR-1/-2 (90.6% vs. 30.7%), which seem to reflect the higher activity of CRISPR/Cas I-F1 systems demonstrated in the aforementioned experimental studies.

**Figure 2:**
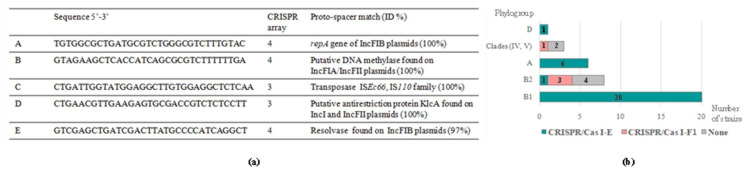
(**a**) Complete spacer sequences matching at least 97% with known plasmids or IS regions. (**b**) Distribution of CRISPR/Cas I-E and I-F1 systems among *E. coli* phylogroups.

Regarding the homology of spacer sequences with known genes (proto-spacers), we found significant differences among CRISPR arrays. Globally, among the 841 spacers present in CRISPR 1 to 4, 59 (7.0%) showed high homology (≥88%) to distinct mobile elements available in public databases. Specifically, in decreasing order of prevalence, 49 (5.8%), 9 (1.1%) and 1 (0.1%) matched viral, plasmid and IS proto-spacers. However, according to previous studies, the distribution of these homologues varies among CRISPR/Cas I-E and I-F1 [22,30]. Supporting the findings of Díez-Villaseñor et al., 2010 [30], and contrary to Aydin et al., 2017 [22], a significant number of spacers in CRISPR-1 and -2 showed homology with different regions of *E. coli* prophage genomes (percentage among the total viral proto-spacers in I-E/reference sequence): E24377A (46.7%/CP000800.1), P1 (15.5%/AF234172), D6 (2.2%/MF356679) and uncharacterized bacteriophages (35.6%). In CRISPR/Cas I-F1 systems, apart from phages, various spacers matched at least 97% with conserved regions of plasmids. We also identified one spacer 100% identical to a specific region of the IS*Ec66* element (IS*110* family, IS*1111* group). The complete nucleotide sequences of these plasmid- or IS-proto-spacers are shown in Figure 2a. Previous research has established a positive correlation between the presence of CRISPR/Cas I-F1 systems and antimicrobial susceptible phenotypes in *E. coli* [22]. Authors concluded that the presence of type I-F1 spacers matching conserved regions within IncI1-, IncFII- and IncFIB-plasmids interfered with the acquisition of resistance plasmids. It is worth noting that despite the different geographical and host origins of the strains, two out of the five spacers they reported in *E. coli* from clinical human specimens were also present in our collection of wildlife commensal strains. This is most likely due to the existence of common ancestral CRISPR loci which evolve, retaining certain spacers in response to the environmental selective pressures. In this regard, the detection of two novel plasmid proto-spacers in CRISPR/Cas I-F1 systems belonging to *E. coli* from wildlife may reflect an improvement in the fitness of bacteria lacking resistance plasmids in environments less exposed to antibiotics.

As previously reported [22,30,31], we observed a clear dependence of the CRISPR/Cas subtype on phylogeny (Figure 2b). On the one hand, concerning CRISPR/Cas I-F1, we mainly found it in B2 group strains (three out of four I-F1-harboring strains). As mentioned before, the presence of this I-F1 system has been associated to low levels of antimicrobial resistance, a frequent pattern observed in B2 isolates [4]. However, some members of the B2 phylogroup, such as those belonging to ST131, have contributed in the last decades to the global dissemination of multi-drug resistance [32]. Notably, in accordance with Aydin et al., 2017 [22], the two strains of the studied collection ascribed to the ST131 complex did not carry CRISPR/Cas systems, which may favor them in the uptake of resistance and virulence determinants under selective environmental conditions. In addition, this suggests a divergent evolution of B2 group members prior to the acquisition of *cas* genes. The remaining strain of the collection carrying a CRISPR/Cas I-F1 system belonged to *Escherichia* cryptic clade V (new ST7630). On the other hand, considering CRISPR/Cas I-E systems, its presence was confirmed in strains ascribed to phylogroups B1, A, D and, more relevantly, B2. Previous analyses on large collections reported the absence of CRISPR/Cas I-E in isolates belonging to B2 group [22,30] or, less frequently, an incomplete I-E system lacking CRISPR 1 array as a consequence of the deletion/truncation of one or more *cas* genes [31].

### 3.2. Plasmidome and Resistome

PLACNETw reconstructions revealed the presence of plasmids in 29/38 *E. coli* genomes (76.3%), showing ample diversity in size, replicon families and number/isolate, which ranged from one to up to five. 

As discussed by Smilie et al., 2010 [33], plasmid size distributions follow a bimodal curve, with average sizes of 5 kb for small plasmids and 200 kb for larger ones. Table 1 summarizes the sizes and genetic features of small plasmids that were present in 28.9% of the isolates. In most cases, they only contained genes involved in plasmid replication and/or mobility. However, they showed a high phylogenetic diversity, encoding replication and relaxase proteins belonging to four different families. Among mobilizable small plasmids, we observed associations between the MOB_P51_ relaxase subfamily and RNAI-encoding loci, which characterized ColE1 plasmids (such as C7382-1), but MOB_P51_ was also present in plasmids with a non-typeable replicon sequence. The same was shown for small MOB_Q_ plasmids, often but not always carrying a Rep similar to that involved in pColE2 replication [col(156)]. The relation of MOB_P5_ and MOB_Q_ small plasmids with diverse replication systems has been already pointed out [2]. Further, due to its scarcity among *Enterobacteriaceae*, it is worth noting the detection of a 3348 bp plasmid (C7382-2) showing the conserved domains of MOB_V2_ and a RepL-like replication protein. Besides small mobilizable plasmids, we found several mini-plasmids carrying barely the information for self-perpetuation (plasmids C6466-2, C7369-1, C7347). They show the smallest length of the entire collection (1.5–2.2 kb) and are homologous to the pKST21 family [col(MG828)], a mosaic group of plasmids only transferable by vertical inheritance that need two *rep* genes (*repA*-*repB*) for autonomous replication [34]. 

Regarding adaptive and cargo genes of small plasmids, most appeared to be cryptic, since their detectable ORFs (one to three) encoded unknown hypothetical proteins. They could represent adaptive genes to the less studied natural environment. Only three of the small plasmids were involved in the production of colicins, carrying specifically the colE1, colN and colE8 operons. Interestingly, comparing our set of small plasmids with those deposited in public databases, five of them were identical or highly similar (≥99%) to others previously reported among *Enterobacteriaceae* of human and livestock origin (Table 1). Hence, according to previous observations, these small replicons, a priori imposing a metabolic cost but scarce benefit to the cell, are common and circulate among *E. coli* from both clinical and environmental settings [35]. The question about whether these structures are simply selfish genetic elements or play a role as moldable vectors in the acquisition of adaptive genes, remains unclear. However, latest evidence points towards the second scenario [36].

Before focusing on larger plasmids, it is necessary to remark the identification of extrachromosomal prophages or phage-like elements in two strains. A 5386 bp sequence, assembled in a unique contig, gave a perfect BLASTn match with the genome of the coliphage phi-X174 (C7347 strain). Further, the reconstruction of the C7136 genome evidenced a large cluster of 13 contigs (~90 kb), well-differentiated from the rest of the genome elements but showing a poor coverage. It contains features and large sequence segments highly homologues to P1 bacteriophages, known to lysogenize bacteria and replicate as independent low-copy number plasmid-like elements [37]. The *repA* protein of P1 and P7 phages belonged to IncY, the incompatibility group associated to C7136 strain. These plasmid-like elements have been involved in the spread of relevant antimicrobial resistance determinants (*bla*_SHV-12_, *mcr*-1…) and are distributed both in human as well as natural environments [37,38]. 

As detailed in Table 2, among the large conjugative plasmids residing in *E. coli* from wildlife, members of the MOB_F12_ subfamily (IncF complex) were predominant, followed by MOB_P12_ (mainly, IncI1). This was not surprising, since these conjugative replicons are well-known to have higher prevalence in *E. coli*, in part due to the lower fitness cost they impose based on their capability for self-transfer repression [39]. The most remarkable was the heterogeneity of plasmids found among our antimicrobial resistance (AMR) strains. Although the resistance rate was low among this collection of *E. coli* from wildlife (21.0%), AMR gene-carrying plasmids belonged to a variety of MOB/Inc families (MOB_F12_/IncF, MOB_P12_/IncI1, MOB_F11_/IncN; MOB_P3_/IncX1; -/IncR). In contrast to what happens in wild natural environments, the overrepresentation of particular AMR-plasmids or clones seems to be more frequent in human-influenced settings. The high antimicrobial pressures tend to select well-adapted replicons (or even clones) with inherent or newly acquired resistance genes to proliferate and rapidly spread.

**Figure 3:**
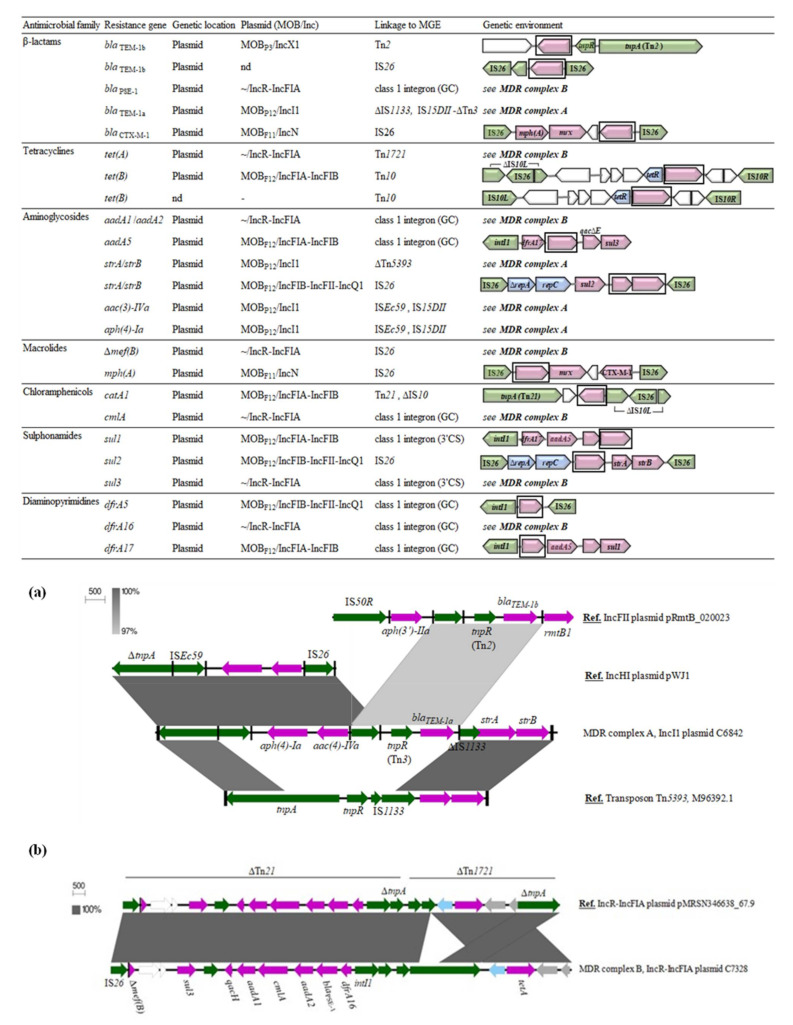
Genetic environment and location of antimicrobial resistance determinants identified in *E. coli* from wildlife. (**a**) BLAST comparison of the novel resistance complex ∆Tn*5393*-*bla*_TEM-1a_-*strA*-*strB* (MDR complex A) with other reference sequences from public databases; (**b**) BLAST comparison of a hybrid Tn*21-1721* transposon carrying an atypical class 1 integron and *tet*(A) in non-conjugative IncR-IncFIA plasmids (MDR complex B). Genes are colored according to their function: purple, resistance to drug or metal; green, recombination/transposition; blue, replication and regulation of gene expression; grey, known function; white, unknown function.

Figure 3 summarizes the genetic surrounding of the main resistance determinants found in the present study. Most of them were included in larger resistance complexes involving different hybrid transposons and insertion sequences, but the resolution of the complete structure was not possible in all cases due to the limitations of short-read sequence data. We therefore showed those well-characterized gene associations owing to a high coverage and previous experimental assays. It should be noted that resistance gene patterns predicted by in silico analyses were consistent with the phenotypic resistance patterns obtained by the disk-diffusion method.

Among the five *bla*_TEM-1_ genes detected, three different genetic surroundings could be identified. The variant *bla*_TEM-1b_, more prevalent, was located upstream of the Tn*2 tnpR* gene, as commonly occurs, but was also found flanked by two IS*26* elements. The *bla*_TEM-1a_ variant, different in three nucleotides but encoding the same TEM-1 protein, was found in a truncated Tn*3* transposon. An IS*26* element was inserted downstream of the Tn*3 tnpR* gene, likely recruiting this IS*26*-*tnpR* (Tn*3*)-*bla*_TEM-1a_ gene pool and integrating it in a ∆Tn*5393* resistance complex by homologous recombination, since no direct target site duplications were identified. Thus, the ∆Tn*5393*-*aph(4)-Ia*-*aac4-IVa*-*bla*_TEM-1a_-*strA*-*strB* complex (MDR complex A in Figure 3a), not previously described, emerged from diverse insertion events within the original Tn*5393* backbone. Probably, the *tnpA* transposase gene was disrupted by the inclusion of the genetic unit IS*Ec59*-*aph(4)-Ia*-*aac4*-IS*26*, frequently found in many bacterial genomes, and was followed by the insertion of the IS*26*-*tnpR*(Tn*3*)-*bla*_TEM-1a_ segment due to a recombinatory event between two directly oriented IS*26.*

The IS*26*-∆*repA*-*repC*-*sul2*-*strA*-*strB*-IS*26* composite transposon, conferring resistance towards sulfonamides and streptomycin, was present in the multireplicon IncFIA-IncFIB-IncQ1 carried by C8124 strain. This cluster, composed by part of the IncQ1 prototype plasmid RSF1010 (M28829), also derived from the Tn*5393* transposon. In this case, Tn*5393* harboring *strA* and *strB* genes was likely transposed into the resistance gene region of RSF1010, containing the *sul2* gene and a mobile element named CR2 [40]. Secondary events removed parts of Tn*5393* and CR2 regions, leading to the conserved ∆*repA*-*repC*-*sul2*-*strA*-*strB* configuration [40], which is widely spread as an IS*26*-bounded transposon. This MGE is present in many bacterial plasmids and integrated chromosomal structures from human, veterinary and environmental settings [40]

Regarding tetracycline efflux pumps encoding genes, *tet(A)* and *tet(B)*, they were located at the well-characterized transposons Tn*1721* and Tn*10*, respectively. In C6468 isolate, a copy of IS*26* disrupted the IS*10*-L transposase gene. Furthermore, three class 1 integron structures were identified, containing the following gene cassette arrangements: (i) *dfrA5*; (ii) *dfrA17*-*aadA5* and (iii) *dfrA16*-*bla*_PSE-1_-*aadA2*-*cmlA*-*aadA1*. The integron carrying the *dfrA17*-*aadA5* array showed the classical 3′-conserved segment (3′-CS), composed by a truncated *qac∆E1* and the sulfonamide resistance *sul1* genes. The results of a Norwegian survey suggested a linkage between this integron and a subpopulation of *E. coli* adapted to a human host [41]. We found this element in two different *E. coli* from wild birds (C6468, C6473), belonging to different STs and harboring distinct MOB_F12_ plasmids. However, although we could not achieve the complete assembly of these plasmids, they showed a very similar resistance gene content. Hence, the spread of *dfrA17*-*aadA5* array seems to be more associated to a stable plasmid-encoded resistance complex rather than to certain clonal lineages. The larger *dfrA16*-*bla*_PSE-1_-*aadA2*-*cmlA*-*aadA1* array, which will be mentioned latter, was an atypical *sul3*-associated class 1 integron. Notably, *dfrA5* gene cassette carrying integron lacked the 3′-CS due to an IS*26*-mediated deletion. The *intI1*-*dfrA5*-IS*26* configuration has been less reported in conventional PCR-based studies targeting integrons, owing to the absence of the hybridization site of the 3′-CS primer. However, as shown, they are present in both human and animal *E. coli* populations [42].

**Figure 4:**
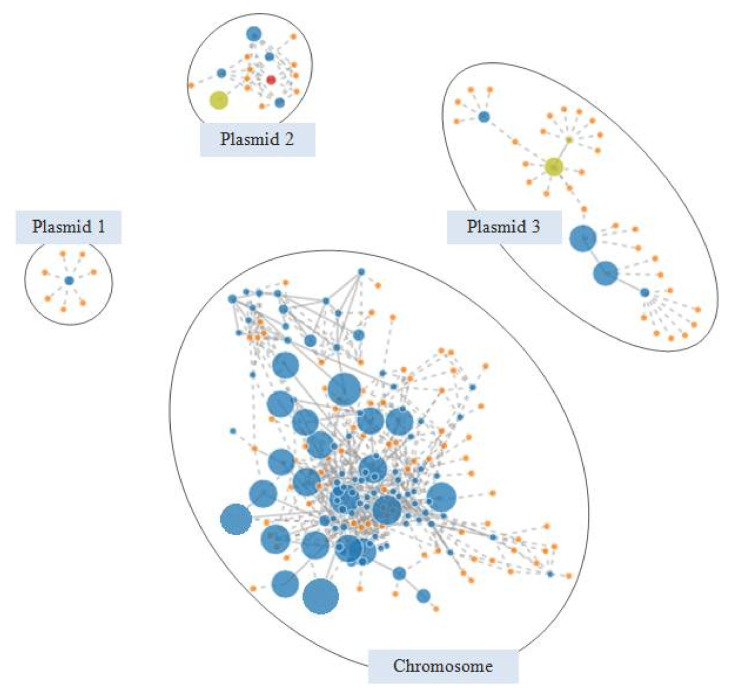
PLACNETw reconstruction of the *E. coli* C7328 genome. After a manual pruning of the original network, four components were differentiated: the chromosome, plasmid 1 (small cryptic plasmid), plasmid 2 (IncN replicon) and plasmid 3 (IncR-IncFIA multi-replicon). Contigs are represented as blue nodes of size proportional to their length. Orange nodes indicate reference genomes. Contigs encoding plasmid relaxases and replication proteins are colored in red and yellow, respectively.

Figure 4 shows the PLACNETw reconstruction of the extended-spectrum β-lactamase producing C7328 strain. Plasmid 2 showed a MOB_F11_ relaxase (red node) and an IncN replicon type (yellow node) and harbored the *bla*_CTX-M-1_ in an IS*26* composite transposon. An incomplete macrolide resistance cluster, with an entire *mphA* and a truncated *mrx* gene, were located downstream of *bla*_CTX-M-1_, leading to the gene block IS*26*-*mph(A)*-∆*mrx*-*orf477*-*bla*_CTX-M-1_-∆IS*Ecp1*-IS*26*. This module, first reported in IncN plasmids of human clinical isolates, has also been described in IncI1 and IncB/O replicons, which suggest the occurrence of IS*26-*mediated transposition events between different plasmid backbones [43,44]. In addition, C7328 strain carried a nontransferable IncR-FIA plasmid of around 70 kb, lacking the relaxase gene (plasmid 3 in Figure 4). Its backbone was highly homologous (99% coverage, 100% identity) to plasmid pMRSN346638_67.9, reported in a clinical *E. coli* isolate [45]. Further, both plasmids carried *aadA1*/*aadA2*, *cmlA1*, *bla*_PSE-1_, *dfrA16*, *sul3* and *tet*(A) genes, encoding for resistance against aminoglycosides (streptomycin), phenicols, β-lactams (penicillin, carbenicillin), trimethoprim, sulfonamides and tetracycline, respectively. Their resistance regions, almost identical, consisted of a hybrid ∆Tn*21*/Tn*1721* transposon containing a large class 1 integron (MDR complex B in Figure 3b). The C7328 strain sequenced in this work corresponded to that included in a previous study carried out using conventional PCR-based approaches [7]. There, we demonstrated the conjugal transferability of the CTX-M-1 encoding gene, but we could not determine neither its complete genetic surrounding nor the genetic basis behind the impossibility of a parallel transference of the class 1 integron and the *tet*(A) determinant. WGS data enabled the genome reconstruction of the strain, which provides a better understanding of the genetic background and the mechanisms involved in the antimicrobial resistance spread.

### 3.3. Virulome

It is necessary to specify that, although a web-based program was used to search for the virulence traits, the high sequence homology between particular determinants (e.g., *iss*/*bor* or *cva*/*mchF*) required manual validation. Unlike acquired AMR, preferentially plasmid-encoded, virulence genes were found in a variety of MGE, including pathogenicity islands (PAIs), prophages and large virulent plasmids. Among the 25 detected virulence-related genes, the highest frequencies were observed for *gad*, *lpfA* and *iss*. Notably, at least one of the two isoenzymes of Gad (glutamate descarboxilase A and B), both in most cases, were found encoded in all genomes, invariably located in previously defined chromosomal *loci* [46]. These stress-related genes, involved in the decarboxylation of glutamate for the maintenance of neutral cytoplasmic pH, seem to be essential in the colonization of the host gut.

**Figure 5:**
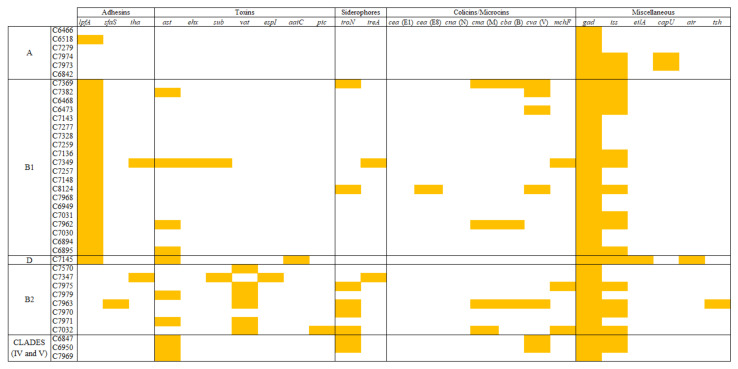
Distribution of the virulence determinants among *E. coli* phylogroups.

As shown in Figure 5, the occurrence of some virulence factors (VFs) varied among *E. coli* phylogroups. For instance, the fimbrial adhesin gene *lpfA* was present in all strains belonging to phylogenetic group B1, but it was absent in B2 isolates. On the contrary, vacuolin autotransporter toxin (*vat*) and, to a lesser extent, enterobactin siderophore receptor (*iroN*) were more prevalent among B2 phylogroup. These differences in VF distribution seem to be in relation with the propensity of certain *E. coli* phylogroups to cause particular infections. In this sense, *lpfA* carriage and B1 phylogroup have been associated to persistent mastitis in cattle [47], whilst B2 phylogroup is more related with avian pathogenic (APEC) and human extraintestinal pathogenic (ExPEC) *E. coli* strains.

**Figure 6:**
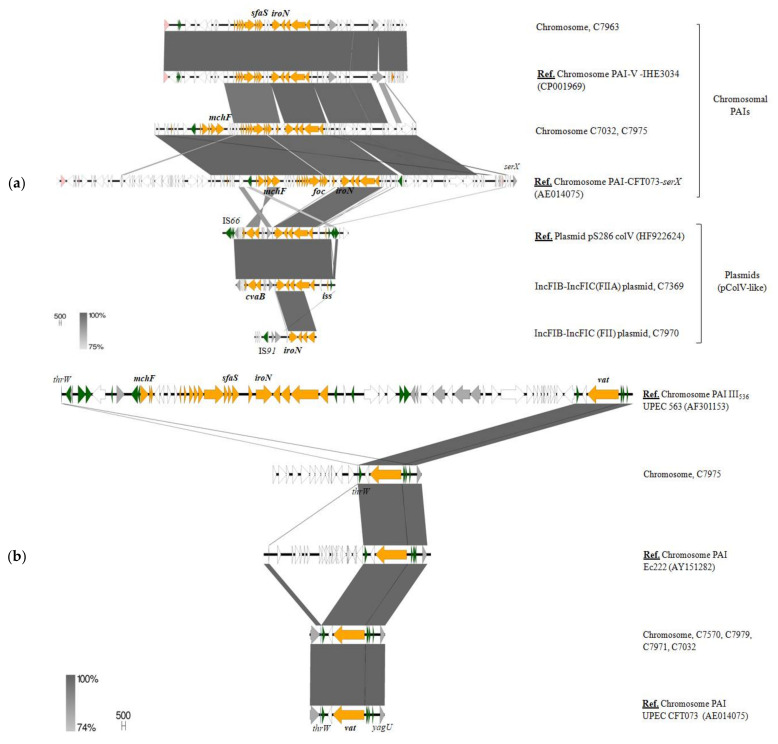
Genetic location and surrounding of *iroN* (**a**) and *vat* (**b**) virulence genes in some of the *E. coli* strains from our collection. The reference genomes used for BLASTn comparisons are indicated as “Ref. Genetic location-Strain name-(accession number)”. Genes are colored according to their function: orange, virulence genes; green, recombination/transposition; pink, prophage genes; grey, known function; white, unknown function.

Focusing on these latter ExPEC/APEC-associated VFs, *vat* and *iroNBCDE* cluster, our results suggest that their linkage to B2 phylogroup is in relation to their preferential location in chromosomal PAIs (Figure 6). As it will be described, *iroN* was also found in B1 and clade IV strains, but in these cases, encoded in large pColV-like MOB_F12_ plasmids (Figure 6a). Regarding *vat*, as previously described in human UPEC (CFT073) and avian APEC (Ec222) strains, it was located on a PAI inserted at the *thrW* tRNA locus, between the *proA* and *yagU* genes. This site seems to be a hotspot for recombination and has been found to carry different VFs such as *iro*N, *sfaS* and microcins in some *E. coli* isolates harboring PAI-III_536_ [48]. However, six out of the eight strains belonging to the B2 phylogroup of our collection only carried *vat* at this insertion point (Figure 6b). Most of them also harbored *iroN*, but in a different genomic location. In fact, this siderophore gene was occupying two different PAIs inserted in the *serX* locus, the so-called PAI-CFT073-*serX* and the island V described in IHE3034 strain [49] (Figure 6a). Apart from the iron locus, PAI-CFT073-*serX* co-harbored the genetic system encoding microcin H47 (*mcmA*, *mchF*, *mchB*, *mchC*) and the *foc* operon, encoding F1C fimbriae. The genetically related SfaS adhesin, also known to play a role in uropathogenesis, was adjacent to *iroNBCDE* locus in PAI-V-IHE3034. However, as mentioned before, the plasmid-borne *iro* cluster was also frequent among *E. coli* from wildlife belonging to various phylogroups (B1, B2, clade IV). pColV-plasmids, coding for the production of colicin V (*cva* gene), were found in 18.4% of the strains. All belonged to MOB_F12_ family and carried different virulence traits such as *iroN*, *iss* (increased serum survival), *tsh* (temperature-sensitive hemagglutinin), *cma* (colicin M) and/or *cmb* (colicin B). These plasmids have been associated to the APEC pathotype and, although their background and virulence region is variable, the so-called “conserved” portion (containing among others *iroN* cluster) shows high homology with certain chromosomal PAIs [50]. Indeed, most of the pColV plasmids identified in *E. coli* from wildlife contained *iss*, *iroN* and *cva* (C7369, C8124, C6847 and C6950 strains), a set of genes associated to the conserved virulence region of these plasmids. Taking into consideration the frequent occurrence of the mentioned ExPEC/APEC-related VFs (e.g., Vat serin-protease, *iroNBCDE* cluster) among the commensal *E. coli* population, these traits seem to primarily contribute to bacterial fitness, competitiveness and gut colonization, rather than being directly involved in infection.

In this regard, bacteriocin synthesis has also been linked to ExPEC strains due to the positive correlation observed between bacteriocin-encoding genes and other virulence determinants [51]. The present study on commensal *E. coli* corroborated this virulence gene association for particular bacteriocins, such as colicins V, B and M and microcin H47. As detailed in Table 3, colicins V, B and M were located on large MOB_F12_ plasmids, while microcin H47 was mainly found in chromosomal PAIs. Both platforms usually co-harbored *iroN* and other aforementioned virulence determinants. However, colE1, E8 and N encoding genes occupied very small plasmids, lacking additional traits. The integration of bacteriocins–siderophores apparently helps *E. coli* to adapt to competitive environments, such as the gut, thereby posing an advantage for commensal populations. However, in combination with additional VFs, these strains can also be more prone to invade other tissues causing extraintestinal infections [52]. 

The Iss factor, frequently associated with colibacillosis in poultry and found on pColV/BM plasmids, was detected in the genome of 55.3% *E. coli* strains. The high prevalence of this gene, located on both plasmids and/or prophage-like elements on the chromosome, has been previously reported [53]. However, three *iss* alleles have been differentiated according to their sequence [54]. The tree of Supplementary Materials Figure S3 was constructed based on most of the *iss* sequences from our collection as well as reference genes from Johnson et al., 2008 [54] and Xu et al., 2018 [53]. Johnson et al., 2008 [54] reported a positive correlation between *iss* type 3 and human ExPEC as well as *iss* type 1 and avian APEC. Xu et al., 2018 [53] demonstrated that differences in the *iss* nucleotide sequence and mRNA copy affect its virulence and serum resistance. As shown in Supplementary Materials Figure S3, the *iss* sequence associated with higher serum livability clustered with *iss* type 1 group, while other representing a lower livability (“type 13”) clustered with *iss* type 2 group. Although all *iss* alleles were represented among *E. coli* from wildlife, most of them encoded the chromosomal, and apparently less virulent, *iss* type 2. 

Considering the VFs involved, to a greater or lesser extent, in intestinal colonization and pathogenicity, we reported *astA*, *aaiC*, *air*, *eilA*, *pic*, *hlyA*, *subA, espI*, *ireA*, *iha* and *capU* (Figure 4). EAST-1 heat-stable toxin encoding gene (*astA*) was mainly found embedded within the putative IS*1414* transposase, as previously described [55]. The C7145 strain harbored different VFs related to the enteroaggregative *E. coli* (EAEC) pathotype (Table 2). In addition to *astA*, it also carried *aaiC* (type VI secretion protein), *air* (enteroaggregative immunoglobulin-repeat protein) and *eilA* (salmonella Hil-A homolog) determinants. Although the type VI secretion system (*aaiA*-*aaiY* genes) was first described in a PAI inserted at *pheU* in the chromosome of the prototype EAEC 042 strain, in C7145, a partial cluster *(aacA*-*P*) was identified on a plasmid. This cluster shared 94% identity with that described in the pAA700-09 plasmid, which also comprised 16 genes [56]. The *air* and *eilA* determinants were found to be chromosomally co-located at *selC* locus. Regarding Pic serine protease autotransporter, implicated in intestinal colonization, it was identified in the chromosome of the C7032 strain showing a configuration highly homologous to that reported in the CFT073 strain. In C7349, genes encoding α-hemolysin (*hlyCABD*) were harbored in a MOB_F12_ plasmid, as widely occur in enteropathogenic strains [57]. Finally, the hexosiltransferase homolog CapU, which is frequently plasmid-borne in EAEC strains, was located in the chromosome of C7973 and C7974 strains, conserving the configuration reported at the same locus in the prototype EAEC 042 strain. 

### 3.4. Phylogenomics

A phylogenetic tree based on single nucleotide polymorphisms (SNPs) in the *core* genome was constructed in order to examine the occurrence of potential adaptive mutations associated with the host. In a first approach, with the aim of defining the genetic diversity of our *E. coli* collection, we performed a phylogenetic analysis considering only the *core* SNPs of the 38 genomes from wild animals. The resulting tree was highly concordant with the population structure inferred by MLST. Thus, additional *E. coli* strains were selected from Enterobase, based on MLST criteria. Up to 31 *E. coli* genomes per sequence type, representing various sources and geographic locations, were downloaded from the NCBI BioProjects database. Information about these genomes is detailed in Supplementary Materials Table S2. The final dataset for the phylogenomic reconstruction, represented by the tree in Supplementary Materials Figure S4, comprised 280 genomes, including those belonging to our collection (*n =* 38). Of the total gene clusters (108,518), only 0.6% (674) were shared among all genomes, resulting in a *core* length of 575,173.5 ± 3802.95 bp. This small *core* genome evidenced the high genetic diversity of the studied collection, particularly highlighted by the remarkable divergence of cryptic *Escherichia* clades (C6847 and C6950 strains-Clade IV/ST322; C7969 strain-Clade V/ST7630). Among the five major clades defined by Walk et al., 2009 [58] (I to V), members of clades II, III, IV and V appeared overrepresented in samples from soils, rivers and aquatic sediments. Wild animals seemed to be spillover hosts of these environmentally well-adapted *Escherichia* lineages. Luo et al., 2009 [59] analyzed nine genomes belonging to these cryptic clades and, by comparing them with enteric *E. coli* strains (including clade I), demonstrated that both differed in the content of a significant number of genes. Further, they did not find the genetic exchange of *core* genes between enteric and environmental clades, contrary to what it was observed within members of each group. Thus, they suggested that the evolution of *Escherichia* genus appeared to be mainly driven by gene acquisition and/or deletion, rather than homologous recombination, which supports our observations. The other obvious reason that could affect the *core* size is the high number of sequences taken from public databases. When using sequence data from different research groups, two questions affecting its quality should be taken into consideration: genome annotation and sequence quality [60]. Although we avoided the bias by assembling and predicting CDS from all reference genomes using Velvet [61] and Prodigal software [62], the quality of the sequences likely varies. 

**Figure 7:**
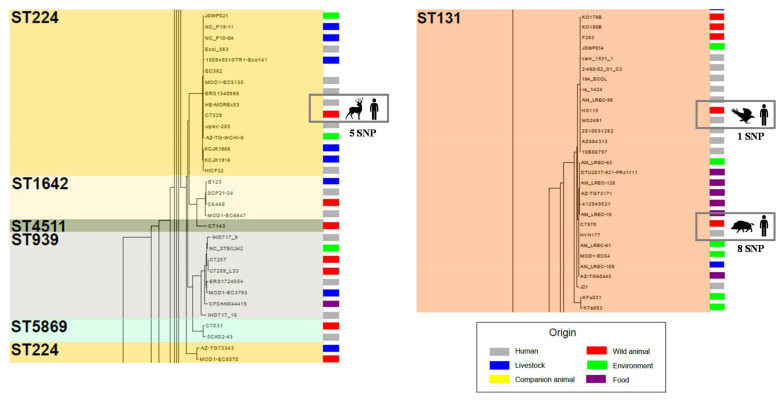
Overview of two of the most populated branches of the *core* genome tree. The very low number of SNP differences observed between strains from wild animals and humans are highlighted in squares. Moreover, ST224 cluster also shows the higher discriminatory power of WGS when compared to conventional MLST typing.

As previously observed [60], the *core* genome tree was highly consistent with MLST data as well as with the phylogroup distribution of our bacterial collection. Only ST7632, ST1642, ST4511, ST939, ST5869 and ST767 fit inside other ST clusters. ST767 and ST7632 are single locus variants of ST58 and ST10, respectively, which explains their close relation. The ST224 clade is split by ST939 and ST1642, which show two locus change, but also by ST5869 and ST4511. These latter ones shared only two and three alleles with ST224, respectively, a fact that remarks the higher resolution of WGS data (Figure 7). Although MLST is highly discriminatory, discordant relationships might be attributed to recombination events in MLST loci.

Regarding the distinct clusters of the tree, most of them contained strains recovered from different sources and distant locations. Further, in many cases, the SNP number (Figure 7, Supplementary Materials Table S3) was significantly lower between strains from different species of animals than within members of the same species. We can point out various examples observed among some of the most populated tree branches (ST10, ST58, ST224 and ST131 clusters). For instance, in the ST58 clade, with an average of 282.8 SNPs between its members, the C7030 strain belonging to a rodent showed the closest relationship with the bovine origin strain ERS1801995 (33 SNPs). A similar observation was reported among members of the widely distributed ST10 (SNP average: 670.6). The *core* genome of the C6842 isolate (rodent, Spain) shared more similarities with MOD1-EC6577 (bovine, Canada) than with other *E. coli* from wildlife hosts. More remarkable was the extremely low number of SNPs (≤10) that differentiated the C7328 strain (deer, Spain) from others belonging to humans (HE-MDREc53, USA; MOD1EC5135, USA; Ecol583, USA) or poultry (NC_P10-04, Uganda), even more so considering the average SNP within ST224 (*n* = 625.8) (Figure 7). That was also the case (≤10 SNP) between C7970 (wild boar, Spain) and HVH 177 (human, Denmark), two members of the major ST131 cluster (SNP average = 259.6) (Figure 7). Thus, no clear evidence of adaptive mutations associated to the *core* genome were found in strains from different hosts. Although further studies with a more homogeneous dataset are required to generalize this assumption, our results reflect an on-going inter-host transmission of *E. coli* strains. Adaptation to the host environment does not seem to leave a genetic mark in the bacterial *core* genome.

## 4. Conclusions

This study contributes to better characterize the commensal gut niche from wildlife, which is crucial for understanding the adaptive pathways and genome diversification of *E. coli*, the role of different elements in bacterial fitness and the flow of genes and gene modules among interconnected ecosystems. Whole genome sequencing enables a more consistent characterization of the composition and diversity of the plasmidome, facilitating the study of small replicons, the differentiation of extra-chromosomal phages or phage-like elements and the detection of non-typeable plasmids. Further, it offers a general view of different genomic regions related to immunity, phylogeny, pathogenesis or resistance, which favors more robust assumptions than those made on the basis of a few set of genes screened by conventional methods. 

Our analyses seem to rule out the occurrence of a parallel evolution of the *E. coli* genome in the gut of wild animals. Bacteria from humans or highly human-influenced settings exhibit similar genetic patterns in CRISPR-Cas systems, plasmids or virulence/resistance gene-carrying modules. These observations, together with the absence of significant genetic changes in their *core* genome, suggest an ongoing flow of both mobile elements and *E. coli* lineages between human and natural ecosystems. 

## Figures and Tables

**Table 1:** Diversity and genetic features of small plasmids found among studied *E. coli* collection. Plasmids have been named according to the strain in which they are harbored (if ≥1 per isolate, they appear enumerated). Those replicons unable to be typed by using PlasmidFinder database are indicated as “nd”.

Small Plasmid	Size (bp)	Relaxasa	Replicon Type	Accessory Genes (Proteins)	≥99% Identical Plasmids (Strain Host)
C7369-2	3371	MOB_P51_	nd	*orf1* (hypothetical potein)	CP012927 (human)
C8124-2	4633	MOB_P51_	col(RNAI)	*orf1* (hypothetical potein)	-
C7382-1	6493	MOB_P51_	col(RNAI)	*cea* (colicin E1), *cei* (colicin E1 immunity), *cel* (colicin E1 lysis), *exc1* (entry exclusion 1), *exc2* (entry exclusion 2)	-
C7962	4203	MOB_P51_	nd	*orf1*, *orf2*, *orf3* (hypothetical poteins)	-
C7971	4182	MOB_P51_	nd	*orf1*, *orf2*, *orf3* (hypothetical poteins)	-
C7969	5428	-	col(RNAI)	*cna* (colicin N), *cni* (colicin N immunity), *cnl* (colicin N lysis), *exc1* (entry exclusion 1), *exc2* (entry exclusion 2)	-
C6466-1	3609	-	col(RNAI)	*orf1* (hypothetical potein)	KU166868 (human), CP006052 (chicken)
C6466-2	1506	-	col(MG828)	*orf1* (hypothetical)	CP010877 (human)
C7369-1	2255	-	col(MG828)	*orf1*, *orf2*, *orf3* (hypothetical)	-
C7347	1736	-	col(MG828)	*orf1* (hypothetical)	-
C6466-3	5132	MOB_Q12_	col(156)	*orf1* (hypothetical)	-
C7369-3	3904	MOB_Q12_	col(156)	*orf1* (hypothetical)	CP012638 (human), CP019896 (beef)
C8124-3	8138	MOB_Qu_	col(156)	*col* (colicin E8), *cei* (3 genes) (colicin immunity), *cel* (2 genes) (colicin lysis); *orf1*, *orf2*, *orf3* (hypothetical)	AP010964 (human)
C8124-1	4110	MOB_Qu_	nd	-	-
C7382-2	3348	MOB_V2_	nd	*orf1* (hypothetical)	-
C7328	3087	-	Col440I	*orf1*, *orf2*, *orf3* (hypothetical)	-
C7974	2717	-	nd	*orf1*, *orf2*, *orf3* (hypothetical)	-
C7973	2717	-	nd	*orf1*, *orf2*, *orf3* (hypothetical)	-

**Table 2:** Genetic features of commensal *E. coli* isolates from wildlife based on in silico WGS analysis.

Strain, Origin ^a^	No. Plasm.	Inc/Rep Types, Phages	Relaxase Protein ^c^	Resistance Genes		Virulence Genes		CRISPR/Cas Systems		ST/PG ^e^
				Chromosome	Plasmids	Chromosome	Plasmids	Subtype	No. Spacers	
C6466, BP	5	IncFII, IncX1, col(156), col(MG828) (2)	MOB_P3_, MOB_Q12_		-	*gad*	-	I-E	4, 6	10/A
C6468, BP	1	IncFIA-IncFIB	MOB_F12_		*tet*(B), *bla*_TEM-1b_, *catA1*, *dfrA17*, *aadA5, aph(3’)-Ia*	*gad*, *lpfA*, *iss*	-	I-E	14, 20	1642/B1
C6473, BP	1	IncFIB-IncFIC(FIIA)	MOB_F12_		*tet*(B), *bla*_TEM-1b_, *catA1*, *dfrA17, aadA5*	*gad*, *lpfA*	*iss*, *cva*	I-E	16, 20	162/B1
C6518, BP	1	IncX1	MOB_P3_		*bla*_TEM-1b_	*gad*, *lpfA*	-	I-E	7, ND	3634/A
C6842, R	1	IncI1	MOB_P12_		*bla*_TEM-1a_, *strA*, *strB*, a*ph(4)-Ia*, *aac(3)-IVa*	*gad*, *iss*	-	I-E	13, 4	10/A
C6847, R	2	IncFIA-IncFII, IncX1-X4	MOB_F12_		-	*gad*, ast	*iss*, *iroN, cva*	-	-	322/Cl. IV
C6894, R	-	-	-		-	*gad*, *lpfA*	-	I-E	26, ND	7631/B1
C6895, R	1	IncFIA-IncFIB-IncFIC(FII)	MOB_F12_		-	*gad*, *lpfA*, *iss*	*astA*	I-E	24, 10	295/B1
C6949, R	nd	IncFII, IncY	nd		-	*gad*, *lpfA*	-	I-E	8, 7	7629/B1
C6950, R	2	IncFIA-IncFII, IncX1-X4	MOB_F12_		-	*gad*, ast	*iss*, *iroN, cva*	-	-	322/Cl. IV
C7030, R	-	-	-		-	*gad*, *lpfA*	-	I-E	12, 20	58/B1
C7031, R	-	-	-		-	*gad*, *lpfA*, *iss*	-	I-E	31, 23	5869/B1
C7032, R	1	IncFIB-IncFII	MOB_F12_		-	*gad*, *iss*, *vat*, *pic, mcmA*, *mchB*, *mchC*, *mchF, iroN*	*cma*	-	-	104/B2
C7136, D	1	IncFII, IncY (phage-like plasmid)	-		-	*gad*, *lpfA*, *iss*	-	I-E	14, 18	1308/B1
C7143, D	-	-			-	*gad*, *lpfA*	-	I-E	25, 23	4511/B1
C7145, D	1	IncB/O/K/Z	MOB_P12_		-	*gad*, *lpfA*, *iss*, *air*, *eilA*	*aaiC*, *astA*	I-E	17, 9	69/D
C7148, D	1	IncFIA-IncFIB-IncFII	MOB_F12_		-	*gad*, *lpfA*	-	I-E	10, 13	906/B1
C7257, D	-	-	-		-	*gad*, *lpfA*	-	I-E	16, 20	939/B1
C7259, D	-	-	-		-	*gad*, *lpfA*	-	I-E	16, 20	939/B1
C7277, D	1	IncI1	MOB_P12_		-	*gad*, *lpfA*	-	I-E	9, 22	7624/B1
C7279, D	-	-	-	*tet*(B)		*gad*, *iss*	-	I-E	4, 3	1718/A
C7328, D	3	IncN, IncR-IncFIA (HI1), SCP ^b^	MOB_F11_		*tet*(A), *bla*_PSE-1_, *bla*_CTX-M-1_, *dfrA16*, *aadA2*, *mph*(A), *sul3*	*gad*, *lpfA*	-	I-E	31, 15	224/B1
C7347, D	1	col(MG828), phage φX174	-		-	*gad, iha*, *ireA*, *subA*, *espI*	-	I-E	4, 1	812/B2
C7349, D	1	IncFIB-IncFII	MOB_F12_		-	*gad*, *lpfA*, *iss*, *iha*, *ireA*, *astA*, *mchC*, *mchB*, *mchF, subA*^d^	*ehxA* (*hlyA*)	I-E	26, 12	26/B1
C7369, BP	4	IncFIB-IncFIC(FIIA), col(MG828), col(156), SCP ^b^	MOB_F12_, MOB_P51_, MOB_Q12_		-	*gad*, *lpfA*, *iss*	*iss*, *iroN*, *cva*, *cma*, *cba*	I-E	12, 27	155/B1
C7382, BP	3	IncFIB-IncC(FIIA), colRNAI, SCP ^b^	MOB_F12_, MOB_P51_, MOB_V2_		-	*gad*, *lpfA*, *iss*, *ast*	*iss*, *cva*, *cea*	I-E	11, 8	767/B1
C7570, BP	-	-			-	*gad*, *vat*	-	I-F1	9, 32	135/B2
C7962, WB	2	IncFII, SCP ^b^	MOB_F12_, MOB_P51_		-	*gad*, *lpfA*, *iss*, *ast*	*ast*, *cma*, *cba*	I-E	20, 11	388/B1
C7963, WB	1	IncFIB-IncFII	MOB_F12_		-	*gad*, *iss*, *iroN*, *vat*, *sfaS*	*iss*, *cva*, *cma*, *cba*, *tsh*	I-F1	6, 9	567/B2
C7968, WB	1	IncFIA-IncFIB	nd		-	*gad*, *lpfA*	-	I-E	7, 12	1423/B1
C7969, WB	2	IncFIB-FII, col(RNAI)	nd		-	*gad, astA*	*cna*	I-F1	21, 12	7630/Cl.V
C7970, WB	1	IncFIB-IncFIC (FII)	MOB_F12_		-	*gad*, *iss*	*iroN*	-	-	131/B2
C7971, WB	1	SCP ^b^	MOB_P51_		-	*gad*, *astA*, *vat*	-	-	-	1170/B2
C7973, WB	2	IncI1, col(RNAI)	MOB_P12_	*tet*(B) ^d^	-	*gad*, *iss*, *capU*	-	I-E	11, 6	7632/A
C7974, WB	nd	IncQ1, col(RNAI)	MOB_P12_	*tet*(B) ^d^	*tet*(A), *bla*_TEM-1b_, *strA*, *strB*, *sul2*	*gad*, *iss*, *capU*	-	I-E	11, 6	7632/A
C7975, WB	-	-			-	*gad*, *iss*, *iroN*, *vat*, *mcmA*, *mchC*, *mchB*, *mchF*	-	I-F1	13, 5	625/B2
C7979, WB	1	IncFIC(FII)	MOB_F12_		-	*gad*, *astA*, *vat*	-	-	-	1317/B2
C8124, WB	4	IncFIB-IncFII-IncQ1, col(RNAI), col(156), SCP ^b^	MOB_F12_, MOB_P51_, MOB_Qu_		*dfrA5*, *strA, strB, sul2*	*gad*, *lpfA*, *iss*	*iss*, *iroN*, *cva*, *cea*	I-E	5, 13	58/B1

^a^ BP: bird of prey; D: deer; WB: wild boar; R: rodent. ^b^ SCP: The strain harbors a small cryptic plasmid but its replicase shows not significant homology with known proteins. ^c^ nd: “non-determined” (plasmid number is unclear due to a complex contig assembly and genome reconstruction) or “not detected” (relaxase and REP domains are not present or could not be found). ^d^ Encoded in fragmented contigs of unclear origin (plasmid or chromosome). ^e^ Sequence type/phylogroup (Cl.: cryptic clade).

**Table 3:** Distribution and genetic location of bacteriocins encoding genes among commensal *E. coli* from wildlife.

Colicin/Microcin	Gene Cluster	Genetic Location	Plasmid or PAI Features		Strain (Phylogroup)	Host
			MOB/Inc	Size (kb)		
E1	*cea*, *cei*, *cel*	Plasmid	MOB_P51_/col(RNAI)	6.6	C7382 (B1)	Bird of prey
E8	*cea*, *cei*, *cel*	Plasmid	MOB_Qu_/col(156)	8.1	C8124 (B1)	Wild boar
H47	*mchB*, *mchC*, *mchF*, *mcmA*	Chromosome	PAI-CFT073-*serX*	-	C7975 (B2)	Wild boar
H47	*mchB*, *mchC*, *mchF*, *mcmA*	Chromosome	PAI-CFT073-*serX*	-	C7032 (B2)	Rodent
H47-like	*mchB*, *mchC*, *mchF*	Chromosome		-	C7349 (B1)	Deer
M, B	*cma-cmi*, *cba-cbi*	Plasmid	MOB_F12_/IncFIB-IncFIC(FIIA)	~120	C7369 (B1)	Bird of prey
M, B	*cma-cmi*, *cba-cbi*	Plasmid	MOB_F12_/IncFII	~100	C7962 (B1)	Wild boar
M, B	*cma-cmi*, *cba-cbi*	Plasmid	MOB_F12_/IncFIB-IncFII	~130	C7963 (B2)	Wild boar
M, B (truncated)	*cma-cmi*, *cba-cbi*	Plasmid	MOB_F12_/IncFIB-IncFII	~120	C7032 (B2)	Rodent
N	*cna*, *cni*, *cnl*	Plasmid	-/col(RNAI)	5.4	C7969 (Clade V)	Wild boar
V	*cvaA*, *cvaB*, *cvaC*	Plasmid	MOB_F12_/IncFIB-IncFIC(FIIA)	~150	C6473 (B1)	Bird of prey
V	*cvaA*, *cvaB*, *cvaC*	Plasmid	MOB_F12_/IncFIA-IncFII	~150	C6847 (Clade IV)	Rodent
V	*cvaA*, *cvaB*, *cvaC*	Plasmid	MOB_F12_/IncFIA-IncFII	~150	C6950 (Clade IV)	Rodent
V	*cvaA*, *cvaB*, *cvaC*	Plasmid	MOB_F12_/IncFIB-IncFII	~130	C7963 (B2)	Wild boar
V	*cvaA*, *cvaB*, *cvaC*	Plasmid	MOB_F12_/IncFIB-IncFII-IncQ1	~150	C8124 (B1)	Wild boar
V	*cvaA*, *cvaB*, *cvaC*	Plasmid	MOB_F12_/IncFIB-IncFIC(FIIA)	~100	C7382 (B1)	Bird of prey
V	*cvaA*, *cvaB*, *cvaC*	Plasmid	MOB_F12_/IncFIB-IncFIC(FIIA)	~120	C7369 (B1)	Bird of prey

## Data Availability

Raw sequence reads reported in this paper were deposited under NCBI BioProject PRJNA699864.

## Supplementary Materials

The following are available online at https://www.mdpi.com/article/10.3390/microorganisms9050999/s1. Figure S1: Structural diversity of the CRISPR/Cas I-E and I-F1 systems regarding the module located between the two CRISPR arrays, comprising the *cas* set of genes and adjacent ORFs. CRISPR arrays depicted in this figure illustrate only its position within the CRISPR/Cas systems (the spacer repertoire of each isolate is represented in Fig. 1). The *cas*-E (*cas2, cas1, cas6, cas5, cas7, cse2, cse1,* and *cas3*) and *cas*-F1 (*cas1-cas2/3-csy1-csy2-csy3-cas6f*) genes, in pink, are located between CRISPR-1/CRISPR-2 loci in I-E system and CRISPR-3/CRISPR 4 in I-F1 system., Figure S2: Phylogenetic tree constructed according to the concatenated amino acid sequences of the *cas* genes. Colour circles denote the different phylogenetic groups (dark blue: B1; red: A; green: D; pink: B2; light blue: Clade V). The concatenated *cas*-E genes of *E. coli* K-12 MG1655 are also included as reference., Figure S3: Phylogenetic tree constructed according to the nucleotide sequences of 21 putative *iss* genes from our *E. coli* collection. Some reference sequences used by Jonhson *et al*. 2008 to define the different *iss* alleles are marked with a red circle. Two reference sequences, distinctly associated with high (AF042279) and low (*iss* type13) serum livability by Xu *et al*. 2018, appear indicated with a blue circle., Figure S4: Phylogenetic relationship of the 38 studied *E. coli* and 242 reference genomes (Table S2). The tree was based on a 575,173.5 ± 3,802.95 bp *core* genome including 674 orthologous common genes, from a total of 108,518 gene clusters, with at least 80% identity and 60% coverage and using 100 bootstrapping replicates. ST types are highlighted in different colors and origin has been indicated in the right part. The pairwise SNP distance matrix used to build the tree is shown in Table S3; Table S1: Genomic features of the 38 *E. coli* genomes from wildlife. Table S2: Information about the genome sequences downloaded from the NCBI for the construction of the phylogenomic *core* tree. Table S3: Pairwise SNP distance matrix calculated from the *core* genome in the set of 242 *E. coli* full genomes from the NCBI database plus 38 *E. coli* genomes from this study. Genomes are ordered according to their Sequence Type (ST).
