# Supplementary material for: Genomic Insights into Drug Resistance and Virulence Platforms, CRISPR-Cas Systems and Phylogeny of Commensal E. coli from Wildlife
Source: Microorganisms. 2021 May 5;9(5):999. doi: 10.3390/microorganisms9050999 (PMC8148099; doi:10.3390/microorganisms9050999)
Supplement: Supplementary file 1 [file microorganisms-09-00999-s001.zip › microorganisms-1173796-supplementary.pdf]

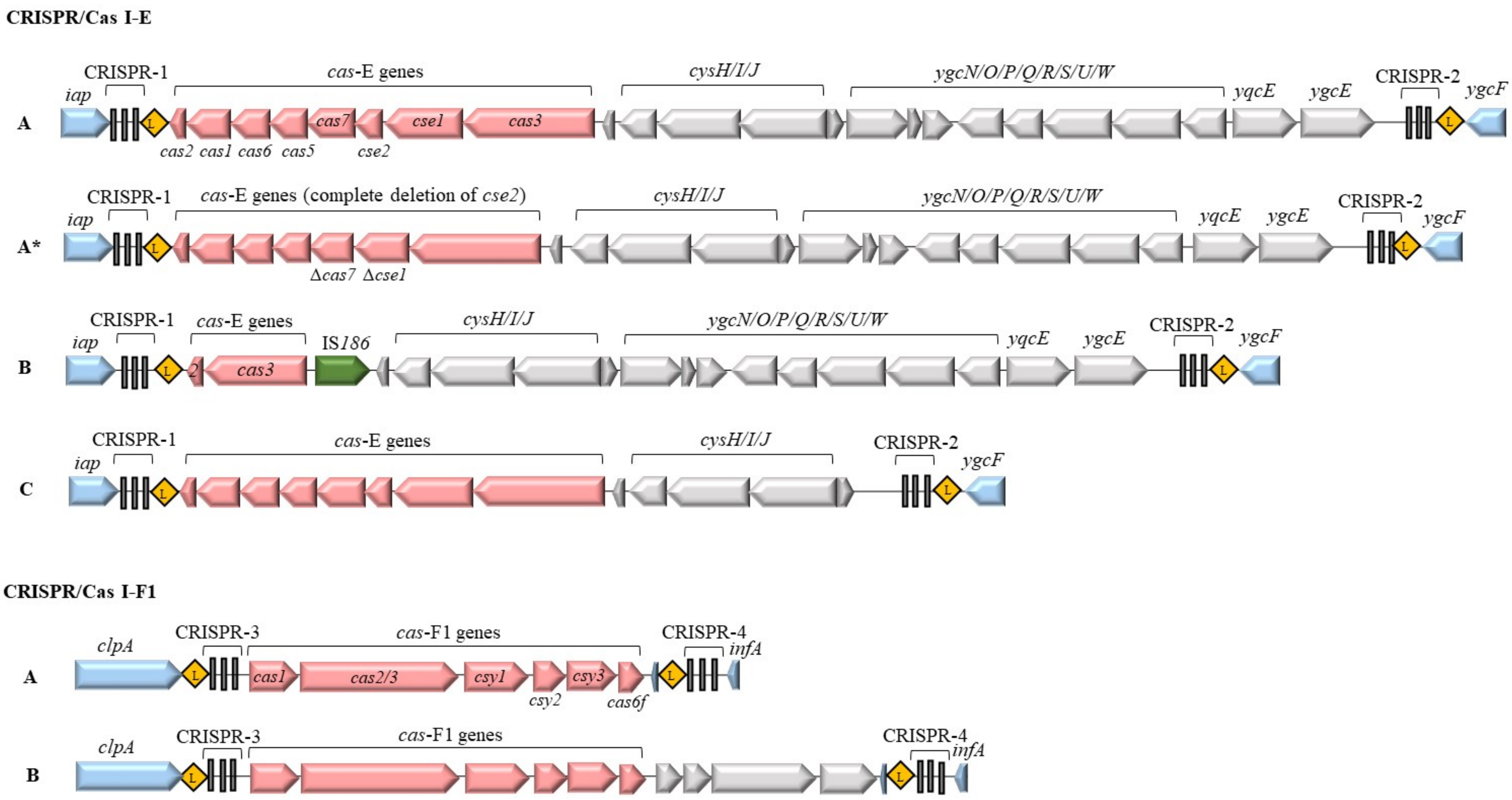

Figure S1. Structural diversity of the CRISPR/Cas I-E and I-F1 systems regarding the module located between the two CRISPR arrays, comprising the *cas* set of genes and adjacent ORFs. CRISPR arrays depicted in this figure illustrate only its position within the CRISPR/Cas systems (the spacer repertoire of each isolate is represented in Fig. 1). The *cas*-E (*cas2*, *cas1*, *cas6*, *cas5*, *cas7*, *cse2*, *cse1*, and *cas3*) and *cas*-F1 (*cas1*-*cas2*/*3*-*csy1*-*csy2*-*csy3*-*cas6f*) genes, in pink, are located between CRISPR-1/CRISPR-2 loci in I-E system and CRISPR-3/CRISPR 4 in I-F1 system.

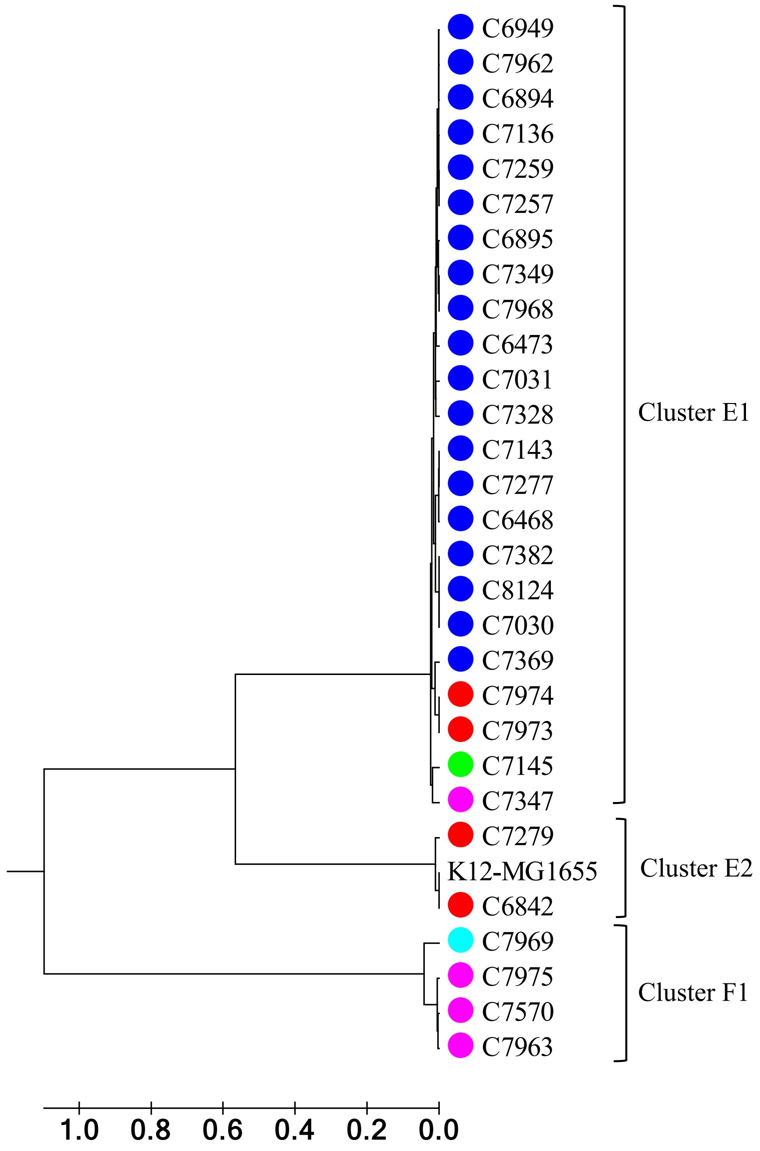

Figure S2. Phylogenetic tree constructed according to the concatenated amino acid sequences of the *cas* genes. Colour circles denote the different phylogenetic groups (dark blue: B1; red: A; green: D; pink: B2; light blue: Clade V). The concatenated *cas*-E genes of *E. coli* K-12 MG1655 are also included as reference.

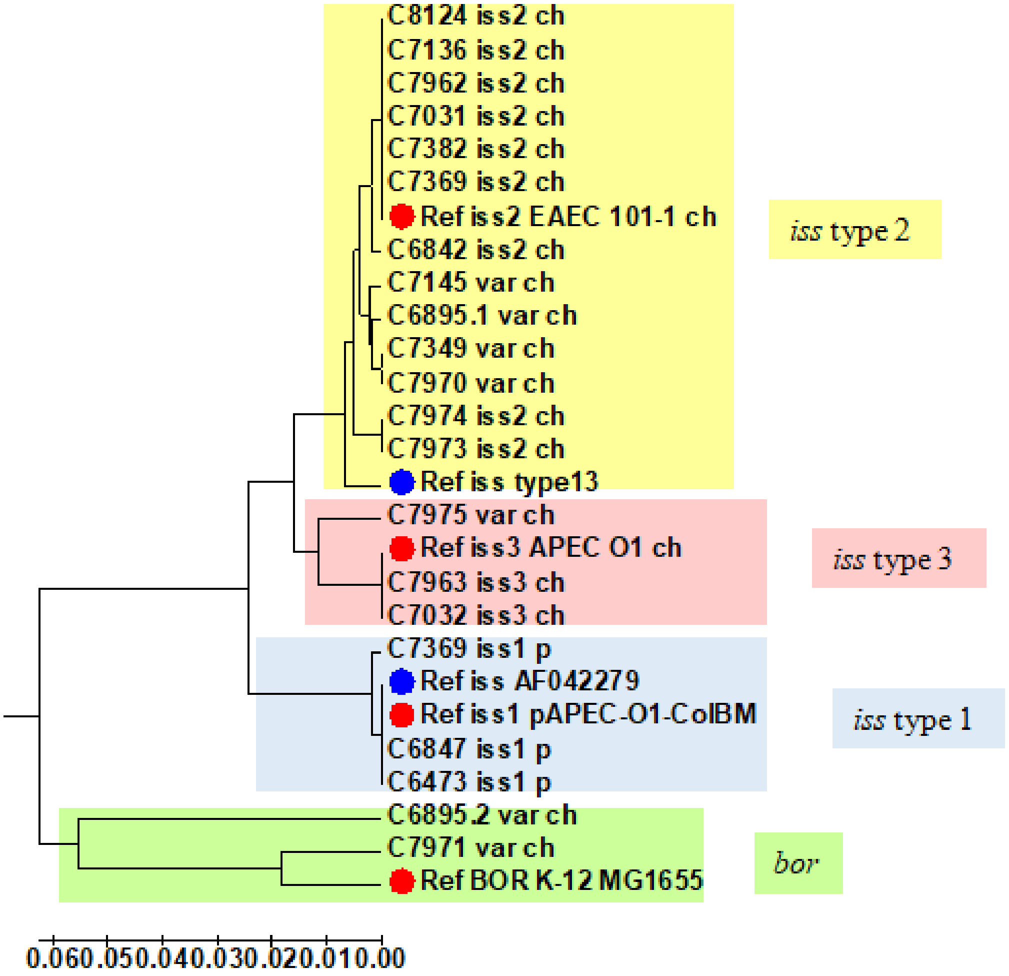

Figure S3. Phylogenetic tree constructed according to the nucleotide sequences of 21 putative *iss* genes from our *E. coli* collection. Some reference sequences used by Jonhson *et al.* 2008 to define the different *iss* alleles are marked with a red circle. Two reference sequences, distinctly associated with high (AF042279) and low (*iss* type13) serum livability by Xu *et al.* 2018, appear indicated with a blue circle.

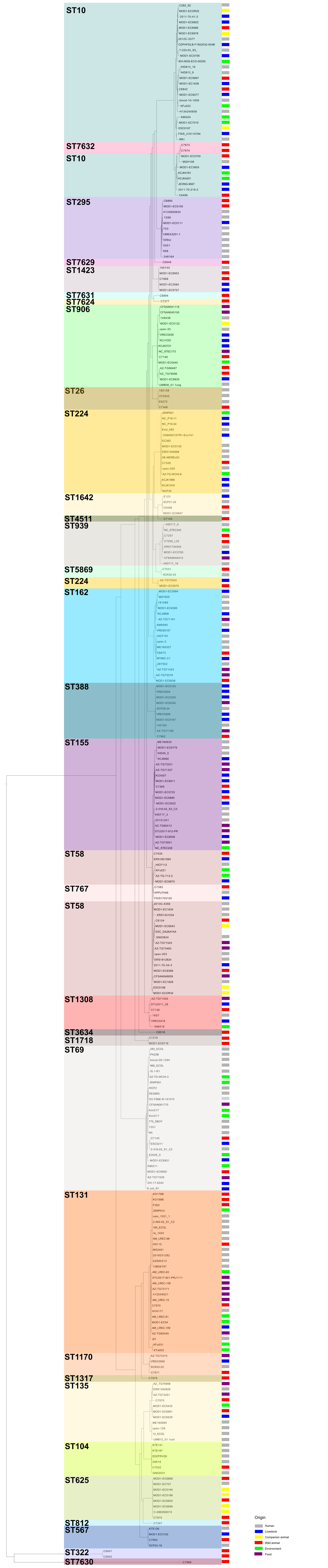

Figure S4. Phylogenetic relationship of the 38 studied *E. coli* and 242 reference genomes (Table S2). The tree was based on a 575,173.5 ± 3,802.95 bp core genome including 674 orthologous common genes, from a total of 108,518 gene clusters, with at least 80% identity and 60% coverage and using 100 bootstrapping replicates. ST types are highlighted in different colors and origin has been indicated in the right part. The pairwise SNP distance matrix used to build the tree is shown in Table S3.

Table S1. Genomic features of the 38 *E. coli* genomes from wildlife

Strain	Host	Kmer length[a]	Total number of contigs	N50	Length of longest contig (bp)	Total bases in contigs (bp)	Number of contigs >1kb	Library size[b]
C6466	Vulture	93	120	210,402	725,488	4,796,454	59	565±141
C6468	Eagle	93	90	470,973	1,125,083	4,874,589	36	558±139
C6473	Eagle	93	112	445,115	650,426	4,988,081	52	574±146
C6518	Vulture	93	189	65,665	249,393	4,294,006	135	563±140
C6842	Mouse	93	195	325,346	475,721	5,182,180	70	542±136
C6847	Mouse	93	153	134,247	360,317	4,901,269	92	480±137
C6894	Mouse	93	85	293,031	571,175	4,743,069	27	529±140
C6895	Mouse	93	114	236,272	782,976	4,892,961	40	540±137
C6949	Mouse	93	228	210,874	463,618	4,962,978	103	515±135
C6950	Mouse	93	119	165,860	381,364	4,897,213	78	535±141
C7030	Mouse	93	128	234,293	410,351	4,865,538	54	512±134
C7031	Mouse	93	97	428,503	784,883	4,763,670	35	498±128
C7032	Rat	89	93	530,999	979,793	5,126,798	43	555±139
C7136	Deer	93	166	180,234	696,338	4,866,503	65	529±143
C7143	Deer	89	65	342,216	869,130	4,676,925	25	561±144
C7145	Deer	85	97	362,421	792,041	4,941,527	36	577±149
C7148	Deer	89	96	268,729	719,168	4,862,403	37	523±140
C7257	Deer	93	71	752,146	1,231,007	4,779,929	28	511±137
C7259	Deer	93	72	752,403	1,230,184	4,784,578	29	519±136
C7277	Deer	93	98	461,644	665,005	4,735,353	34	577±145
C7279	Deer	85	115	274,335	856,709	4,742,823	41	533±139
C7328	Deer	93	121	324,391	809,631	4,848,065	55	513±135
C7347	Deer	89	285	215,722	553,632	5,357,341	128	510±133
C7349	Deer	93	362	159,157	624,325	5,670,872	137	514±134
C7369	Vulture	93	154	254,251	527,553	5,016,477	62	527±133
C7382	Vulture	93	194	146,319	359,912	4,855,394	94	513±131
C7570	Osprey	89	54	1,186,763	1,198,710	4,675,804	16	545±144
C7962	Wild boar	93	261	163,773	285,618	5,294,199	106	526±142
C7963	Wild boar	85	71	383,603	771,743	4,921,820	32	565±148
C7968	Wild boar	89	104	299,133	721,689	4,827,556	40	585±154
C7969	Wild boar	93	109	341,003	858,984	4,619,792	47	502±132
C7970	Wild boar	89	159	468,673	651,730	5,356,538	50	576±151
C7971	Wild boar	85	214	281,351	1,516,878	5,177,062	64	528±134
C7973	Wild boar	93	158	183,741	396,053	4,889,167	73	562±143
C7974	Wild boar	93	162	170,712	395,963	4,916,442	72	568±147
C7975	Wild boar	93	108	572,135	738,094	5,170,693	42	574±148
C7979	Wild boar	93	87	730,474	1,227,241	4,781,399	27	578±150
C8124	Wild boar	89	168	133,480	420,216	4,963,309	79	501±130

[a]Selected Kmer length by VelvetOptimizer and Velvet software implemented in PLACNETw. The Kmer screening was performed from 69 to 101 bp.

[b]Theoretical insert size of 550bp, according to the TruSeq PCR-Free Illumina protocol size selection for these libraries.

Table S2. Information about the genome sequences downloaded from the NCBI for the construction of the phylogenomic *core* tree.

Strain name[a]	Source	Host	Country	Biosample	Bioproject
**ST10 (N=31)**					
2011-70-219-2	Feces	Swine	Denmark	SAMEA3268855	PRJEB8647
2011-70-41-2	Feces	Swine	Denmark	SAMEA3268833	PRJEB8647
2012C-3377	Feces	Human	US	SAMN02991194	PRJNA218110
7-233-03_S3_	Feces	Human	Tanzania	SAMN02687488	PRJNA233845
blood-10-1009	Feces	Human	USA	SAMN02801879	PRJNA248737
C260_92	Feces	Human	Italy	SAMN02435961	PRJNA79239
CDPHFDLB-F1602032-004B	Feces	Bovine	USA	SAMN05275919	PRJNA277984
ESC0167	Feces	Dog	USA	SAMN02368174	PRJNA203445
FSIS 210115784	Feces	Bovine	USA	SAMN04293387	PRJNA292667
H134240608	Feces	Human	UK	SAMN03492675	PRJNA248042
IEH-NGS-ECO-00205	Vegetables	Vegetables	USA	SAMN04429914	PRJNA230969
IHD813_16	Feces	Human	Cambodia	SAMN03326089	PRJNA274331
IHD813_9	Feces	Human	Cambodia	SAMN03326082	PRJNA274331
JEONG-9567	Feces	Bovine	USA	SAMN04160767	PRJNA298331
KCJK4181	Soil	Environmental	USA	SAMN05363780	PRJNA298331
KCJK4201	Soil	Environmental	USA	SAMN05408390	PRJNA298331
KFu023	Water	River/Environmental	Japan	SAMD00053131	PRJDB4884
KMi024	Water	River/Environmental	Japan	SAMD00053162	PRJDB4884
MGH108	Feces	Human	Not Available	SAMN03280204	PRJNA271899
MOD1-EC1638	Feces	Swine	Italy	SAMN05607400	PRJNA230969
MOD1-EC3605	Feces	Bovine	USA	SAMN05596397	PRJNA230969
MOD1-EC5700	Feces	Pronghorn	USA	SAMN05452920	PRJNA230969
MOD1-EC5706	Feces	Poultry	USA	SAMN05452915	PRJNA230969
MOD1-EC6577	Feces	Bovine	Canada	SAMN04992402	PRJNA230969
MOD1-EC6802	Feces	Bovine	USA	SAMN04992166	PRJNA230969
MOD1-EC6897	Feces	Mink	USA	SAMN04992263	PRJNA230969
MOD1-EC6966	Feces	Slender-horned gazel	USA	SAMN04992332	PRJNA230969
MOD1-EC6978	Feces	Dog	USA	SAMN04992344	PRJNA230969
MOD1-EC7010	Water	Environmental	USA	SAMN04992376	PRJNA230969

MOD1-ECOR25	Feces	Dog	USA	SAMN04158362	PRJNA230969
RR1	Feces	Human	Not Available	SAMN03384316	PRJNA272568
**ST26 (N=3)**					
93272	Feces	Human	UK	SAMN06017286	PRJNA315192
182138	Feces	Human	UK	SAMN06029396	PRJNA315192
272422	Feces	Human	UK	SAMN06040333	PRJNA315192
**ST58 (N=20)**					
2011-70-34-3	Feces	Swine	Denmark	SAMEA3268813	PRJEB8647
2013C-4350	Feces	Human	USA	SAMN03019947	PRJNA218110
AZ-TG-713-2	Water	Environmental	USA	SAMN04450992	PRJNA230968
AZ-TG71543	Meat	Turkey	USA	SAMN02463308	PRJNA230968
AZ-TG73483	Meat	Chicken	USA	SAMN02628625	PRJNA230968
CFSAN046659	Spinach	Vegetables	USA	SAMN05414504	PRJNA312475
ERS1341034	Feces	Human	Netherlands	SAMEA4429585	PRJEB15226
ERS1801995	Feces	Bovine	Spain	SAMEA104142977	PRJEB21546
ERS1812824	Feces	Human	Congo	SAMEA104153806	PRJEB21637
ESC0198	Feces	Dog	USA	SAMN02368195	PRJNA203445
GN03624	Feces	Human	USA	SAMN04388560	PRJNA290784
HICF112	Feces	Human	UK	SAMN04357562	PRJNA306133
KFu021	Water	River/Environmental	Japan	SAMD00053129	PRJDB4884
MOD1-EC1626	Feces	Human	Germany	SAMN05607409	PRJNA230969
MOD1-EC1634	Feces	Human	Canada	SAMN05607404	PRJNA230969
MOD1-EC6368	Feces	Parrot	USA	SAMN04992984	PRJNA230969
MOD1-EC6870	Feces	Bovine	USA	SAMN04992236	PRJNA230969
MOD1-EC6943	Feces	Dog	USA	SAMN04992309	PRJNA230969
MOD1-ECOR34	Feces	Dog	USA	SAMN04913880	PRJNA230969
upec-203	Feces	Human	USA	SAMN02802024	PRJNA248737
**ST69 (N=24)**					
1351	Feces	Human	Germany	SAMEA3180512	PRJEB8084
18.1-R1	Feces	Human	Not Available	SAMN05567362	PRJNA335932
2-316-03_S1_C2	Feces	Human	Tanzania	SAMN02689038	PRJNA233728
283_ECOL	Feces	Human	USA	SAMN03197477	PRJNA267549
775_SBOY	Feces	Human	USA	SAMN03197985	PRJNA267549

966_ECOL	Feces	Human	USA	SAMN03198186	PRJNA267549
AZ-TG-WCHI-3	Water	Wastewater	USA	SAMN04448145	PRJNA230968
AZ-TG71539	Meat	Turkey	USA	SAMN02463307	PRJNA230968
blood-09-1294	Feces	Human	USA	SAMN02801858	PRJNA248737
CFSAN061770	Cheese	Food	Egypt	SAMN06928086	PRJNA230969
E. coli_R1	Feces	Swine	Denmark	SAMEA4464657	PRJEB15511
E2026_9	Water	Environmental	Australia	SAMN06109006	PRJNA356186
EC-F86E-R-141010	Feces	Human	Singapore	SAMEA3920428	PRJEB13304
ESC0211	Feces	Sheep	Papua New Guinea	SAMN02368206	PRJNA203445
HICF2	Feces	Human	UK	SAMN04357497	PRJNA306133
JSWP001	Water	Sewage	Japan	SAMD00076198	PRJDB5602
KMi011	Water	River/Environmental	Japan	SAMD00053149	PRJDB4884
Kmi017	Water	River/Environmental	Japan	SAMD00053155	PRJDB4884
MOD1-EC6831	Feces	Bovine	USA	SAMN04992197	PRJNA230969
MOD1-EC6900	Feces	Mink	USA	SAMN04992266	PRJNA230969
NA	Feces	Human	Pakistan	SAMN03074765	PRJNA261540
OH-17-6342	Feces	Bovine	USA	SAMN07229622	PRJNA338676
PA20B	Feces	Human	Australia	SAMEA2392724	PRJEB5742
SEQ895	Feces	Human	USA	SAMN00623094	PRJNA66227
**ST104 (N=5)**					
29618	Feces	Human	UK	SAMN05965842	PRJNA315192
EDZFRVQ5	Feces	Human	Denmark	SAMEA104060891	PRJEB20792
GN02531	Feces	Human	USA	SAMN03922992	PRJNA290784
KTE141	Feces	Human	Denmark	SAMN00854673	PRJNA164985
KTE187	Feces	Human	Not Available	SAMN00974042	PRJNA163387
**ST131 (N=28)**					
412049521	Meat	Chicken	Denmark	SAMEA3952454	PRJEB13885
2010031282	Feces	Human	Spain	SAMEA1486614	PRJEB2968
10B06797	Feces	Human	Thailand	SAMN04159585	PRJNA297860
184_ECOL	Feces	Human	USA	SAMN03197375	PRJNA267549
2-460-02_S1_C2	Feces	Human	Tanzania	SAMN02689041	PRJNA233730
AM_LREC-109	Feces	Swine	Spain	SAMEA92141668	PRJEB19190
AM_LREC-128	Meat	Chicken	Spain	SAMEA92140918	PRJEB19190

AM_LREC-15	Meat	Chicken	Spain	SAMEA92136418	PRJEB19190
AM_LREC-61	Water	Sewage	Spain	SAMEA92116918	PRJEB19190
AM_LREC-63	Water	River/Environmental	Spain	SAMEA92138668	PRJEB19190
AM_LREC-98	Feces	Human	Spain	SAMEA92127418	PRJEB19190
AZ-TG60445	Meat	Turkey	USA	SAMN02442854	PRJNA230968
AZ-TG73171	Meat	Chicken	USA	SAMN02628547	PRJNA230968
AZ684313	Feces	Human	France	SAMN04159647	PRJNA297860
cam_1531_1	Feces	Human	Cambodia	SAMN04159619	PRJNA297860
DTU2017-821-PRJ1111	Meat	Chicken	Germany	SAMEA104205911	PRJEB22091
F283	Feces	Raven	USA	SAMN04273110	PRJNA295914
HS115	Feces	Raven	Serbia	SAMN04273112	PRJNA295914
HVH 177 (4-2876612)	Feces	Human	Denmark	SAMN01885818	PRJNA186205
J21	Feces	Human	China	SAMN04273114	PRJNA295914
JSWP014	Water	Sewage	Japan	SAMD00076211	PRJDB5602
KFu031	Water	River/Environmental	Japan	SAMD00053139	PRJDB4884
KO178B	Feces	Raven	Czech Republic	SAMN04273115	PRJNA295914
KO198B	Feces	Raven	Serbia	SAMN04273116	PRJNA295914
KTa003	Water	River/Environmental	Japan	SAMD00053193	PRJDB4884
la_1424	Feces	Human	Laos	SAMN04159627	PRJNA297860
MOD1-EC54	Feces	Water	USA	SAMN05440287	PRJNA230969
MS2481	Feces	Human	Australia	SAMEA1486653	PRJEB2968
**ST135 (N=10)**					
12_ECOL	Feces	Human	USA	SAMN03197161	PRJNA267549
AZ_TG76998	Meat	Chicken	USA	SAMN03295432	PRJNA230968
AZ-TG73251	Meat	Chicken	USA	SAMN02628567	PRJNA230968
ERS1340929	Feces	Human	Netherlands	SAMEA4429480	PRJEB15226
ME160685	Feces	Human	Ireland	SAMEA104091836	PRJEB19435
MOD1-EC5432	Soil	Environmental	USA	SAMN06240039	PRJNA230969
MOD1-EC6529	Feces	Chicken	India	SAMN04993151	PRJNA230969
MOD1-EC6891	Feces	Mink	USA	SAMN04992257	PRJNA230969
UMB12_01.1uot	Feces	Human	Not Available	SAMN04606432	PRJNA269984
upec-128	Feces	Human	USA	SAMN02801940	PRJNA248737
**ST155 (N=19)**					

20151201	Feces	Human	Vietnam	SAMEA104188693	PRJEB21997
2-316-03_S3_C3	Feces	Human	Tanzania	SAMN02680240	PRJNA233839
AZ-TG60412	Meat	Chicken	USA	SAMN02442818	PRJNA230968
AZ-TG71327	Meat	Chicken	USA	SAMN02463254	PRJNA230968
AZ-TG73331	Meat	Chicken	USA	SAMN02628587	PRJNA230968
AZ-TG73651	Meat	Chicken	USA	SAMN02628666	PRJNA230968
DTU2017-812-PR	Meat	Chicken	Denmark	SAMEA104205902	PRJEB22091
IHD45_5	Feces	Human	Cambodia	SAMN03325983	PRJNA274331
IHD717_3	Feces	Human	Cambodia	SAMN03326060	PRJNA274331
KCh007	Feces	Chicken	Japan	SAMD00029119	PRJDB3552
KCJ9492	Feces	Chicken	USA	SAMN04510562	PRJNA298331
ME160633	Feces	Human	Ireland	SAMEA104083455	PRJEB19435
MOD1-EC5522	Feces	Chicken	USA	SAMN05440399	PRJNA230969
MOD1-EC5722	Feces	Chicken	USA	SAMN05440438	PRJNA230969
MOD1-EC6779	Feces	Human	Zambia	SAMN04992127	PRJNA230969
MOD1-EC6811	Feces	Bovine	USA	SAMN04992175	PRJNA230969
MOD1-EC6885	Feces	Mink	USA	SAMN04992251	PRJNA230969
MOD1-EC6936	Feces	Bovine	USA	SAMN04992302	PRJNA230969
NC_STEC228	Soil	Environmental	USA	SAMN06645904	PRJNA293225
**ST162 (N=16)**					
181089	Feces	Human	UK	SAMN05171060	PRJNA315192
287552	Feces	Human	UK	SAMN06006011	PRJNA315192
AMA940	Feces	Human	Denmark	SAMEA3712541	PRJEB12145
AZ-TG71191	Meat	Bovine	USA	SAMN02463220	PRJNA230968
AZ-TG71423	Meat	Turkey	USA	SAMN02463278	PRJNA230968
AZ-TG73319	Meat	Chicken	USA	SAMN02628584	PRJNA230968
HICF191	Feces	Human	UK	SAMN04357615	PRJNA306133
KCJ3858	Feces	Bovine	USA	SAMN04396087	PRJNA298331
ME160327	Feces	Human	Ireland	SAMEA90399418	PRJEB19435
MOD1-EC3564	Feces	Bovine	USA	SAMN05596239	PRJNA230969
MOD1-EC6385	Feces	Human	Argentina	SAMN04993002	PRJNA230969
MOD1-EC6938	Feces	Okapi	USA	SAMN04992304	PRJNA230969
MS7925	Feces	Human	Australia	SAMN07173914	PRJNA383436
MT66C.C1	Feces	Chicken	Vietman	SAMEA104188708	PRJEB21997

upec-3	Feces	Human	USA	SAMN02802119	PRJNA248737
VRES0107	Feces	Bovine	UK	SAMEA3753383	PRJEB8776
**ST224 (N=16)**					
1508493 1STR1-Eco 141	Feces	Bovine	Belgium	SAMEA104142822	PRJEB21546
AZ-TG-WCHI-8	Wastewater	Environmental	USA	SAMN04450979	PRJNA230968
AZ-TG73343	Meat	Chicken	USA	SAMN02628590	PRJNA230968
Ecol_583	Feces	Human	USA	SAMN05511172	PRJNA316786
ERS1340998	Feces	Human	Netherlands	SAMEA4429549	PRJEB15226
HE-MDREc53	Feces	Human	USA	SAMD00052673	PRJDB4868
HICF32	Feces	Human	UK	SAMN04357516	PRJNA306133
HICF32	Feces	Human	UK	SAMN04357516	PRJNA306133
JSWP021	Sewage	Environmental	Japan	SAMD00076218	PRJDB5602
KCJK1866	Feces	Bovine	USA	SAMN05757714	PRJNA298331
KCJK1916	Feces	Bovine	USA	SAMN05757745	PRJNA298331
MOD1-EC5070	Feces	Rhinoceros	USA	SAMN04279407	PRJNA230969
MOD1-EC5135	Feces	Human	USA	SAMN04279473	PRJNA230969
NC_P10-04	Feces	Poultry	Uganda	SAMN06677723	PRJNA293225
NC_P19-11	Feces	Poultry	Uganda	SAMN06848984	PRJNA293225
upec-205	Feces	Human	USA	SAMN02802026	PRJNA248737
**ST295 (N=10)**					
703	Feces	Human	Guatemala	SAMEA1317744	PRJEB2796
998	Feces	Human	Egypt	SAMEA1317826	PRJEB2796
1599	Feces	Human	Argentina	SAMEA1317753	PRJEB2796
5051	Feces	Human	India	SAMEA1031195	PRJEB2581
246164	Feces	Human	UK	SAMN05163814	PRJNA315192
509sc	Feces	Human	Mexico	SAMEA1531067	PRJEB2827
H124600634	Feces	Human	UK	SAMN03492212	PRJNA259645
MOD1-EC5105	Feces	White-tailed deer	USA	SAMN04444411	PRJNA230969
MOD1-EC5111	Feces	Bovine	USA	SAMN04279447	PRJNA230969
UMEA 3201-1	Feces	Human	Sweden	SAMN01885926	PRJNA186316
**ST388 (N=9)**					
140183	Feces	Human	UK	SAMN06015110	PRJNA315192
AZ-TG71195	Meat	Beef	USA	SAMN02463221	PRJNA230968

MOD1-EC3330	Feces	Bovine	USA	SAMN05595849	PRJNA230969
MOD1-EC5153	Feces	Bovine	USA	SAMN04279491	PRJNA230969
MOD1-EC5167	Feces	Swine	USA	SAMN04279506	PRJNA230969
MOD1-EC6332	Meat	Beef	USA	SAMN05440432	PRJNA230969
SCP29-34	Feces	Human	Netherlands	SAMEA4428949	PRJEB15226
VREC0504	Feces	Bovine	UK	SAMEA3751071	PRJEB8774
VREC0506	Feces	Bovine	UK	SAMEA3752553	PRJEB8774
**ST567 (N=3)**					
KTE126	Feces	Human	Denmark	SAMN00854665	PRJNA164969
MOD1-EC3102	Feces	Swine	USA	SAMN05597370	PRJNA230969
SCP24-18	Feces	Human	Netherlands	SAMEA4429670	PRJEB15226
**ST625 (N=7)**					
C-0863N0015	Feces	Dog	UK	SAMEA3681630	PRJEB11950
MOD1-EC5194	Feces	Dog	USA	SAMN04279533	PRJNA230969
MOD1-EC5196	Feces	Cat	USA	SAMN04448470	PRJNA230969
MOD1-EC6825	Feces	Mink	USA	SAMN04992191	PRJNA230969
MOD1-EC6868	Feces	Turttle	USA	SAMN04992234	PRJNA230969
MOD1-EC6946	Feces	Dog	USA	SAMN04992312	PRJNA230969
MOD1-EC707	Feces	Human	USA	SAMN05591522	PRJNA230969
**ST767 (N=2)**					
FSIS1703155	Feces	Swine	USA	SAMN07450955	PRJNA292667
KPPUTH06	Feces	Human	Thailand	SAMN07450700	PRJNA389557
**ST906 (N=14)**					
149438	Feces	Human	UK	SAMN05966787	PRJNA315192
AZ_TG78596	Feces	White-tailed deer	USA	SAMN04125194	PRJNA230968
AZ-TG98487	Feces	Elk	USA	SAMN07549825	PRJNA230968
CFSAN041116	Lettuce	Vegetables	USA	SAMN04273142	PRJNA230969
CFSAN045100	Leafy Greens	Vegetables	USA	SAMN04422341	PRJNA230969
KCJ1232	Feces	Bovine	USA	SAMN04191558	PRJNA298331
KCJK2721	Feces	Bovine	USA	SAMN05163873	PRJNA298331
MOD1-EC5122	Feces	Dog	USA	SAMN04279460	PRJNA230969
MOD1-EC5440	Soil	Environmental	USA	SAMN05452839	PRJNA230969
MOD1-EC6835	Feces	Bovine	USA	SAMN04992201	PRJNA230969

NC_STEC173	Cucumber	Vegetables	Not Available	SAMN06645880	PRJNA293225
UMB08_01.1uog	Feces	Human	USA	SAMN04606426	PRJNA269984
upec-33	Feces	Human	USA	SAMN02802123	PRJNA248737
VREC0426	Feces	Bovine	UK	SAMEA3472083	PRJEB8774
**ST939 (N=6)**					
CFSAN044415	Meat	Bovine	USA	SAMN04902864	PRJNA230969
ERS1724554	Feces	Human	Denmark	SAMEA104060661	PRJEB20792
IHD717_16	Feces	Human	Cambodia	SAMN03326073	PRJNA274331
IHD717_9	Feces	Human	Cambodia	SAMN03326066	PRJNA274331
MOD1-EC3793	Feces	Bovine	USA	SAMN05440254	PRJNA230969
NC_STEC242	Manure	Environmental	USA	SAMN07469552	PRJNA293225
**ST1170 (N=3)**					
AZ-TG73315	Meat	Chicken	USA	SAMN02628583	PRJNA230968
SCK53-23	Feces	Human	Netherlands	SAMEA4429607	PRJEB15226
VREC0559	Feces	Turkey	UK	SAMEA3753304	PRJEB8774
**ST1308 (N=5)**					
1657	Feces	Human	Indonesia	SAMEA1031202	PRJEB2581
AZ-TG71555	Meat	Turkey	USA	SAMN02463311	PRJNA230968
DTU2011_26	Feces	Swine	Denmark	SAMEA3268837	PRJEB8647
KMi019	Water	River/Environment	Japan	SAMD00053157	PRJDB4884
VREC0418	Feces	Bovine	UK	SAMEA3472069	PRJEB8774
**ST1423 (N=4)**					
195745	Feces	Human	UK	SAMN05170676	PRJNA315192
MOD1-EC3584	Feces	Bovine	USA	SAMN05596345	PRJNA230969
MOD1-EC5737	Feces	Bovine	USA	SAMN05439294	PRJNA230969
MOD1-EC6953	Feces	Klipspringer	USA	SAMN04992319	PRJNA230969
**ST1642 (N=3)**					
E123	Feces	Chicken	Denmark	SAMN05981813	PRJNA352460
MOD1-EC6847	Feces	Human	USA	SAMN04992213	PRJNA230969
SCP21-24	Feces	Human	Netherlands	SAMEA4428509	PRJEB15226
**ST1718 (N=1)**					
MOD1-EC6716	Feces	Parrot	USA	SAMN04992546	PRJNA230969
**ST5869 (N=1)**					

SCK02-43	Feces	Human	Netherlands	SAMEA4427901	PRJEB15226

[a]ST : Sequence Type

Table S3. Pairwise SNP distance matrix calculated from the core genome in the set of 2

		C6466	C6468	C6473	C6518	C6842	C6847	C6894
ST10	C6466		3593	3613	3404	809	24961	3597
	C260_92	828	3525	3539	3344	346	24897	3525
	MOD1-ECOR25	830	3523	3539	3346	348	24901	3525
	2011-70-41-2	859	3549	3589	3376	378	24918	3562
	MOD1-EC6802	836	3540	3592	3420	361	24904	3539
	MOD1-EC6966	834	3562	3617	3429	367	24925	3561
	MOD1-EC6978	834	3562	3617	3429	367	24925	3561
	2012C-3377	831	3580	3582	3405	346	24938	3578
	CDPHFDLB-F1602032-004B	833	3579	3585	3407	348	24941	3582
	7-233-03_S3_	904	3584	3642	3399	410	24943	3617
	MOD1-EC5706	1047	3621	3625	3474	536	24940	3640
	IEH-NGS-ECO-00205	841	3587	3617	3396	385	24930	3599
	IHD813_16	1050	3700	3729	3526	412	24952	3689
	IHD813_9	1050	3700	3729	3526	412	24952	3689
	MOD1-EC6897	902	3637	3660	3417	261	24917	3616
	MOD1-EC1638	902	3626	3658	3417	251	24927	3613
	C6842	809	3505	3520	3352		24901	3484
	MOD1-EC6577	884	3546	3581	3407	172	24912	3533
	blood-10-1009	908	3606	3616	3372	271	24917	3582
	KFu023	1064	3633	3630	3177	450	24932	3606
	H134240608	944	3648	3645	3410	296	24920	3646
	KMi024	1209	3541	3543	3448	711	24914	3558
	MOD1-EC7010	1073	3610	3642	3449	535	24927	3612
	ESC0167	1040	3630	3675	3515	536	24931	3638
	FSIS_210115784	957	3527	3554	3409	538	24900	3515
	RR1	1017	3574	3628	3411	518	24921	3573
	MOD1-EC5700	993	3558	3615	3453	1076	24946	3516
	MGH108	1243	3693	3718	3526	1205	24889	3636
	MOD1-EC3605	871	3629	3657	3509	904	24880	3635
	KCJK4181	683	3578	3602	3415	806	24923	3567
	KCJK4201	683	3578	3602	3415	806	24923	3567
	JEONG-9567	686	3581	3605	3417	809	24927	3569
	2011-70-219-2	729	3603	3590	3410	852	24932	3535
ST7632	C7973	987	3558	3612	3441	1078	24938	3518
	C7974	987	3558	3612	3441	1078	24938	3518
ST295	C6895	3554	1969	1948	3823	3469	24987	1778
	MOD1-EC5105	3547	1945	1950	3831	3465	24984	1769
	H124600634	3556	1935	1941	3840	3473	24989	1757
	1599	3520	1974	1965	3805	3451	24958	1836
	MOD1-EC5111	3520	1933	1929	3814	3456	24971	1759
	703	3556	1943	1940	3836	3461	24988	1769
	UMEA3201-1	3555	1942	1939	3835	3460	24987	1768
	509sc	3536	1925	1931	3810	3456	24988	1767
	5051	3499	1965	1967	3809	3467	24980	1791
	998	3536	1985	1980	3771	3474	24960	1800
	246164	3518	2051	2072	3758	3474	24986	1870
ST7629	C6949	3588	1946	1977	3788	3540	24974	1902
ST1423	195745	3406	1865	1838	3665	3332	25011	1447
	MOD1-EC6953	3401	1860	1833	3660	3327	25010	1442
	C7968	3407	1866	1839	3666	3333	25016	1448
	MOD1-EC3584	3401	1860	1833	3660	3327	25010	1442
	MOD1-EC5737	3406	1865	1838	3665	3332	25015	1447
ST7631	C6894	3597	1813	1819	3763	3484	25051	

**ST7624**	**C7277**	3658	2086	1905	3847	3555	25036	1618
**ST906**	**CFSAN041116**	3590	1772	1815	3787	3478	25018	1604
	**CFSAN045100**	3589	1771	1814	3786	3477	25020	1603
	**149438**	3584	1764	1809	3779	3472	25015	1598
	**MOD1-EC5122**	3586	1766	1811	3781	3474	25016	1600
	**upec-33**	3649	1774	1763	3782	3533	25008	1524
	**VREC0426**	3658	1783	1772	3791	3542	25016	1533
	**KCJ1232**	3658	1783	1772	3791	3542	25017	1533
	**KCJK2721**	3613	1703	1760	3772	3505	25005	1519
	**NC_STEC173**	3636	1722	1781	3791	3528	25017	1540
	**C7148**	3565	1742	1784	3765	3485	24999	1528
	**MOD1-EC5440**	3605	1695	1752	3764	3497	24998	1511
	**AZ-TG98487**	3604	1745	1831	3784	3520	25028	1515
	**AZ_TG78596**	3604	1745	1831	3784	3520	25028	1515
	**MOD1-EC6835**	3593	1734	1820	3774	3509	25015	1504
	**UMB08_01.1uog**	3618	1716	1813	3761	3508	25009	1535
**ST26**	**182138**	3584	1909	1857	3812	3494	24978	1655
	**272422**	3615	1940	1888	3843	3525	25006	1686
	**93272**	3586	1911	1859	3814	3496	24980	1657
	**C7349**	3584	1909	1857	3812	3494	24978	1655
**ST224**	**JSWP021**	3765	1892	2110	3840	3675	24974	2079
	**NC_P19-11**	3768	1894	2112	3842	3678	24976	2082
	**NC_P10-04**	3762	1888	2106	3836	3672	24972	2076
	**Ecol_583**	3759	1885	2103	3833	3669	24967	2073
	**1508493 1STR1-Eco 141**	3768	1893	2113	3836	3681	24972	2071
	**EC362**	3759	1883	2101	3833	3667	24969	2071
	**MOD1-EC5135**	3761	1887	2105	3835	3671	24971	2075
	**ERS1340998**	3768	1876	2120	3832	3678	24967	2076
	**HE-MDREc53**	3761	1887	2105	3835	3671	24971	2075
	**C7328**	3760	1886	2104	3834	3670	24969	2074
	**upec-205**	3721	1881	2115	3828	3627	24970	2065
	**AZ-TG-WCHI-8**	3762	1902	2158	3848	3662	24963	2096
	**KCJK1866**	3689	1885	2113	3783	3615	24998	2095
	**KCJK1916**	3689	1885	2113	3783	3615	24998	2095
	**HICF32**	3689	1885	2113	3783	3615	24998	2095
	**AZ-TG73343**	3748	1961	2133	3923	3657	24964	2067
	**MOD1-EC5070**	3744	1889	2055	3944	3675	24987	1993
**ST14642**	**E123**	3605	34	2068	3859	3509	24974	1829
	**SCP21-24**	3593	10	2067	3853	3505	24973	1813
	**C6468**	3593		2067	3853	3505	24975	1813
	**MOD1-EC6847**	3588	74	2071	3852	3514	24983	1787
**ST4511**	**C7143**	3553	1347	1985	3818	3484	24983	1902
**ST939**	**IHD717_9**	3671	2060	1972	3836	3557	24959	1947
	**NC_STEC242**	3707	2181	2092	3848	3605	24969	2050
	**C7257**	3692	2174	2127	3837	3604	24973	2065
	**C7259_L33**	3692	2174	2127	3837	3604	24973	2065
	**ERS1724554**	3685	2213	2147	3829	3591	24978	2082
	**MOD1-EC3793**	3675	2136	2127	3820	3574	24984	2026
	**CFSAN044415**	3713	2120	2143	3818	3621	24967	2044
	**IHD717_16**	3787	2149	2196	3805	3695	24971	2043
**ST5869**	**C7031**	3745	2028	2246	3908	3659	24986	2284
	**SCK02-43**	3735	2028	2281	3903	3659	25012	2307
	**MOD1-EC3564**	3572	1995	569	3820	3491	24972	1768
	**MS7925**	3577	2006	585	3820	3503	24981	1779
	**181089**	3583	1939	487	3830	3497	24982	1696

ST162	MOD1-EC6385	3583	1941	489	3830	3497	24985	1700
	KCJ3858	3583	1969	451	3838	3497	24991	1696
	AZ-TG71191	3583	2057	473	3780	3491	24986	1768
	AMA940	3563	1957	353	3761	3513	24978	1776
	VRES0107	3563	1957	353	3760	3513	24975	1776
	HICF191	3594	2041	38	3800	3520	24976	1792
	upec-3	3596	2043	40	3802	3522	24978	1794
	ME160327	3596	2043	40	3802	3522	24978	1794
	C6473	3613	2067		3810	3520	24976	1819
	MT66C.C1	3591	2040	40	3797	3523	24979	1799
	287552	3573	2030	138	3759	3501	24966	1819
	AZ-TG71423	3574	2031	139	3760	3502	24968	1820
	AZ-TG73319	3588	1823	659	3865	3532	25027	1675
	MOD1-EC6938	3590	1815	660	3877	3536	25029	1669
ST388	MOD1-EC5153	3532	1958	1946	3795	3495	25041	1931
	VREC0504	3532	1960	1946	3793	3495	25041	1933
	MOD1-EC3330	3524	1950	1938	3787	3487	25035	1923
	MOD1-EC6332	3523	1949	1937	3786	3486	25034	1922
	SCP29-34	3531	1973	1965	3812	3490	25036	1942
	VREC0506	3523	1949	1939	3788	3486	25032	1922
	MOD1-EC5167	3524	1950	1938	3787	3487	25035	1923
	140183	3538	1964	1952	3801	3501	25042	1937
	AZ-TG71195	3526	1993	1941	3790	3482	25028	1967
	C7962	3549	2028	1987	3807	3514	25051	1953
ST155	ME160633	3803	1959	2162	4030	3734	25029	2013
	MOD1-EC6779	3799	1974	2170	4031	3736	25037	2021
	IHD45_5	3821	1977	2178	4046	3752	25050	2029
	KCJ9492	3830	1952	2123	4033	3761	25040	1982
	AZ-TG73331	3720	1838	2008	3905	3663	25045	1828
	AZ-TG71327	3727	1829	2000	3893	3654	25040	1827
	KCh007	3727	1829	2000	3893	3654	25040	1827
	MOD1-EC6811	3725	1827	1998	3891	3652	25038	1825
	C7369	3737	1845	2006	3900	3662	25050	1831
	MOD1-EC5722	3765	1830	1942	3913	3689	25042	1795
	MOD1-EC6885	3759	1815	1983	3914	3688	25058	1805
	MOD1-EC5522	3808	1891	2073	3979	3741	25044	1879
	2-316-03_S3_C3	3720	1888	2006	3867	3707	25037	1886
	IHD717_3	3761	1815	1951	3920	3686	25056	1815
	20151201	3787	1834	1941	3895	3704	25059	1821
	AZ-TG60412	3782	1842	1957	3897	3705	25056	1836
	DTU2017-812-PR	3787	1847	1962	3902	3710	25061	1841
	MOD1-EC6936	3764	1862	1943	3911	3683	25047	1834
	AZ-TG73651	3736	1838	1977	3901	3661	25045	1834
	NC_STEC228	3731	1833	1970	3896	3654	25043	1829
	C7030	3740	1805	1904	3917	3671	25038	1849
	ERS1801995	3748	1813	1905	3921	3671	25042	1850
	HICF112	3848	2002	2061	3965	3771	25036	2008
	KFu021	3857	2011	2070	3974	3780	25044	2017
	AZ-TG-713-2	3841	1995	2054	3958	3764	25029	2001
	MOD1-EC6870	3793	1884	1911	3923	3706	25027	1916
	2013C-4350	3756	1819	1901	3924	3671	25041	1860
	MOD1-EC1634	3761	1820	1902	3927	3676	25038	1860
	ERS1341034	3773	2099	2084	3995	3735	25035	2111
	C8124	3753	1964	2007	3876	3704	25019	1970
	MOD1-EC6943	3760	1967	2003	3871	3697	25017	1969

**ST58**	**ESC_DA2647AA**	3784	1977	2013	3895	3716	25026	1979
	**GN03624**	3779	1980	2016	3888	3711	25034	1982
	**AZ-TG71543**	3801	1958	1995	3936	3734	25021	1989
	**AZ-TG73483**	3786	1896	1976	3910	3692	25045	1940
	**upec-203**	3767	1889	1966	3903	3682	25040	1933
	**ERS1812824**	3765	1887	1964	3899	3680	25037	1929
	**2011-70-34-3**	3736	1809	1907	3904	3651	25033	1869
	**MOD1-EC6368**	3748	1816	1879	3920	3670	25028	1854
	**CFSAN046659**	3726	1833	1937	3925	3691	25047	1885
	**MOD1-EC1626**	3719	1824	1930	3918	3684	25040	1876
	**ESC0198**	3759	1769	1831	3935	3672	25029	1803
	**MOD1-ECOR34**	3759	1769	1831	3935	3672	25029	1803
**ST767**	**C7382**	3742	1871	1936	3924	3670	25038	1908
	**KPPUTH06**	3737	1866	1931	3919	3665	25033	1904
	**FSIS1703155**	3740	1869	1934	3922	3668	25034	1907
**ST1308**	**AZ-TG71555**	3524	1887	1863	3704	3455	24942	1918
	**DTU2011_26**	3525	1888	1862	3705	3457	24941	1919
	**C7136**	3512	1875	1851	3692	3445	24932	1906
	**1657**	3684	2078	2065	3808	3615	24944	2147
	**VREC0418**	3544	1919	1903	3714	3487	24961	1988
	**KMi019**	3738	2186	2047	3946	3680	25174	2179
**ST3634**	**C6518**	3404	3853	3810		3352	25018	3763
**ST69**	**283_ECOL**	6647	6612	6733	6928	6598	25059	6658
	**PA20B**	6649	6614	6735	6930	6600	25061	6660
	**blood-09-1294**	6646	6611	6732	6927	6597	25057	6657
	**966_ECOL**	6648	6613	6734	6929	6599	25058	6659
	**18.1-R1**	6785	6662	6778	7004	6741	25031	6705
	**AZ-TG-WCHI-3**	6772	6650	6790	6992	6731	25033	6694
	**JSWP001**	6778	6656	6795	7006	6733	25040	6698
	**HICF2**	6814	6698	6821	7036	6773	25019	6738
	**SEQ895**	6828	6712	6835	7050	6787	25033	6752
	**EC-F86E-R-141010**	6791	6688	6806	7013	6737	25018	6733
	**CFSAN061770**	6870	6728	6847	7088	6829	25028	6768
	**Kmi017**	6800	6682	6813	7022	6753	25015	6722
	**Kmi017**	6829	6712	6843	7051	6782	25045	6752
	**775_SBOY**	6791	6665	6800	7003	6744	25010	6705
	**1351**	6806	6680	6817	7026	6759	25017	6718
	**NA**	6806	6688	6819	7028	6759	25023	6728
	**C7145**	6979	6843	6952	7176	6934	25057	6866
	**ESC0211**	6974	6838	6947	7171	6929	25052	6861
	**2-316-03_S1_C2**	6938	6805	6911	7141	6877	25043	6832
	**E2026_9**	6802	6705	6838	6985	6770	25028	6730
	**MOD1-EC6831**	6924	6825	6942	7136	6877	25056	6842
	**KMi011**	6787	6712	6817	6993	6740	25023	6725
	**MOD1-EC6900**	6781	6706	6811	6987	6734	25018	6719
	**AZ-TG71539**	6786	6705	6810	6984	6739	25019	6718
	**OH-17-6342**	6769	6683	6782	6962	6720	25010	6690
	**E.coli_R1**	6821	6726	6804	7022	6773	25033	6748
**ST1718**	**C7279**	4192	3637	3298	4449	4169	25029	3429
	**MOD1-EC6716**	4181	3626	3287	4438	4158	25018	3418
	**KO178B**	7533	7580	7541	7769	7481	25534	7605
	**KO198B**	7533	7580	7541	7769	7481	25534	7605
	**F283**	7532	7579	7540	7768	7480	25533	7604
	**JSWP014**	7531	7578	7539	7767	7479	25532	7603
	**cam_1531_1**	7533	7580	7541	7767	7481	25534	7605

ST131	2-460-02_S1_C2	7531	7578	7539	7767	7479	25532	7603
	184_ECOL	7533	7580	7541	7769	7481	25534	7605
	la_1424	7531	7578	7539	7767	7479	25532	7603
	AM_LREC-98	7534	7581	7542	7770	7482	25535	7606
	HS115	7531	7578	7539	7767	7479	25532	7603
	MS2481	7530	7577	7538	7766	7478	25531	7602
	2010031282	7531	7578	7539	7767	7479	25532	7603
	AZ684313	7532	7579	7540	7768	7480	25533	7604
	10B06797	7530	7579	7540	7768	7480	25533	7604
	AM_LREC-63	7613	7692	7682	7866	7577	25552	7705
	DTU2017-821-PRJ1111	7617	7696	7686	7870	7581	25553	7709
	AM_LREC-128	7617	7696	7686	7870	7581	25555	7709
	AZ-TG73171	7620	7699	7689	7873	7584	25558	7712
	412049521	7544	7653	7637	7834	7512	25546	7660
	AM_LREC-15	7614	7693	7683	7867	7578	25553	7706
	C7970	7607	7684	7674	7861	7571	25535	7695
	HVH177	7605	7682	7672	7859	7569	25532	7693
	AM_LREC-61	7610	7687	7677	7864	7574	25539	7698
	MOD1-EC54	7605	7682	7672	7859	7569	25534	7693
	AM_LREC-109	7606	7684	7674	7860	7570	25535	7695
	AZ-TG60445	7612	7688	7678	7866	7576	25538	7701
	J21	7515	7547	7557	7797	7451	25544	7570
	KFu031	7509	7596	7588	7769	7466	25510	7607
	KTa003	7507	7598	7598	7769	7464	25514	7609
ST1170	AZ-TG73315	7701	7745	7732	7925	7648	25595	7770
	VREC0559	7701	7745	7732	7925	7648	25595	7770
	SCK53-23	7708	7750	7737	7929	7655	25600	7775
	C7971	7754	7777	7775	7964	7705	25610	7812
ST1317	C7979	7703	7772	7755	7931	7650	25585	7778
ST135	AZ_TG76998	7561	7620	7616	7718	7527	25519	7622
	ERS1340929	7564	7623	7619	7721	7530	25521	7625
	AZ-TG73251	7571	7628	7626	7728	7537	25525	7630
	C7570	7556	7620	7618	7733	7522	25479	7613
	MOD1-EC5432	7546	7600	7590	7706	7508	25516	7602
	MOD1-EC6891	7554	7608	7598	7713	7516	25523	7610
	MOD1-EC6529	7545	7599	7589	7705	7507	25515	7601
	ME160685	7615	7674	7666	7776	7567	25549	7665
	upec-128	7617	7676	7668	7778	7569	25551	7667
	12_ECOL	7621	7680	7672	7782	7573	25555	7671
	UMB12_01.1uot	7634	7693	7685	7795	7586	25566	7684
ST104	KTE187	7604	7588	7599	7753	7541	25497	7605
	KTE141	7605	7589	7600	7754	7540	25498	7606
	EDZFRVQ5	7605	7589	7600	7754	7542	25498	7606
	29618	7606	7590	7601	7755	7543	25499	7607
	C7032	7604	7588	7599	7753	7541	25497	7605
	GN02531	7606	7589	7601	7755	7543	25505	7607
ST625	MOD1-EC6868	7576	7625	7619	7777	7527	25524	7625
	MOD1-EC707	7575	7624	7618	7776	7526	25523	7624
	MOD1-EC5194	7574	7623	7617	7775	7525	25522	7623
	MOD1-EC5196	7573	7622	7616	7774	7524	25521	7622
	MOD1-EC6825	7575	7624	7618	7776	7526	25523	7624
	MOD1-EC6946	7574	7623	7617	7775	7525	25522	7623
	C-0863N0015	7557	7600	7594	7756	7508	25545	7600
	C7975	7577	7626	7620	7778	7528	25525	7626
ST812	C7347	7646	7689	7690	7800	7587	25557	7705

**ST567**	**KTE126**	7691	7701	7668	7804	7643	25570	7692
	**MOD1-EC3102**	7694	7704	7671	7807	7646	25572	7695
	**C7963**	7692	7702	7669	7805	7644	25569	7693
	**SCP24-18**	7689	7700	7666	7802	7641	25572	7690
**ST322**	**C6847**	24961	24975	24976	25018	24901		25051
	**C6950**	24960	24974	24975	25017	24900	1	25050
**ST7630**	**C7969**	30934	30957	31009	31054	30907	27325	30973

242 E.coli full genomes from the NCBI database plus 38 E. coli genomes from this study

C6895	C6949	C6950	C7030	C7031	C7032	C7136	C7143	C7145	C7148	C7257	C7259_L33
3554	3588	24960	3740	3745	7604	3512	3553	6979	3565	3692	3692
3481	3540	24896	3690	3656	7542	3450	3492	6972	3510	3635	3635
3483	3540	24900	3690	3656	7544	3448	3494	6976	3510	3635	3635
3536	3598	24917	3725	3707	7569	3488	3516	6972	3551	3672	3672
3513	3558	24903	3704	3685	7595	3448	3529	6981	3522	3663	3663
3543	3576	24924	3738	3698	7550	3474	3541	6983	3546	3678	3678
3543	3576	24924	3738	3698	7550	3474	3541	6983	3546	3678	3678
3537	3595	24937	3747	3709	7577	3489	3561	7009	3563	3690	3690
3539	3597	24940	3749	3712	7581	3492	3561	7011	3566	3692	3692
3576	3623	24942	3789	3747	7535	3523	3587	6969	3597	3720	3720
3614	3618	24939	3792	3746	7528	3553	3626	6973	3612	3717	3717
3584	3617	24929	3777	3720	7581	3504	3580	6995	3571	3700	3700
3649	3725	24951	3867	3820	7616	3637	3694	7009	3705	3779	3779
3649	3725	24951	3867	3820	7616	3637	3694	7009	3705	3779	3779
3576	3652	24916	3797	3765	7578	3563	3631	6985	3615	3724	3724
3574	3655	24926	3794	3763	7583	3560	3620	6997	3612	3721	3721
3469	3540	24900	3671	3659	7541	3445	3484	6934	3485	3604	3604
3495	3577	24911	3724	3704	7563	3498	3526	6945	3542	3647	3647
3570	3609	24916	3776	3746	7569	3547	3602	6930	3598	3697	3697
3611	3669	24931	3783	3796	7537	3540	3596	6977	3623	3739	3739
3623	3691	24919	3815	3782	7593	3579	3640	6988	3617	3759	3759
3493	3546	24913	3671	3677	7544	3433	3525	6941	3558	3657	3657
3595	3627	24926	3771	3714	7554	3535	3581	6942	3611	3692	3692
3628	3641	24930	3801	3739	7590	3562	3632	7002	3623	3714	3714
3491	3542	24899	3674	3653	7536	3461	3533	6945	3506	3606	3606
3555	3578	24920	3710	3682	7597	3535	3566	6983	3570	3645	3645
3482	3536	24945	3645	3643	7587	3486	3489	6978	3464	3635	3635
3606	3643	24888	3804	3748	7606	3623	3638	6995	3623	3724	3724
3584	3643	24879	3751	3714	7544	3582	3601	6958	3603	3699	3699
3493	3544	24922	3697	3680	7556	3508	3556	6958	3557	3624	3624
3493	3544	24922	3697	3680	7556	3508	3556	6958	3557	3624	3624
3495	3547	24926	3699	3683	7558	3510	3559	6959	3560	3628	3628
3516	3571	24931	3693	3706	7579	3517	3549	6972	3527	3625	3625
3474	3530	24937	3637	3639	7581	3480	3487	6972	3470	3631	3631
3474	3530	24937	3637	3639	7581	3480	3487	6972	3470	3631	3631
	1798	24986	1844	2442	7611	1812	2034	6859	1813	2380	2380
75	1778	24983	1845	2427	7611	1803	2021	6849	1804	2363	2363
127	1781	24988	1833	2417	7612	1805	2033	6854	1786	2362	2362
196	1810	24957	1840	2391	7622	1837	2051	6837	1783	2337	2337
107	1769	24970	1815	2417	7597	1791	2033	6845	1788	2363	2363
149	1781	24987	1841	2419	7614	1809	2037	6850	1792	2375	2375
148	1780	24986	1840	2418	7613	1808	2036	6849	1791	2374	2374
191	1792	24987	1814	2419	7612	1771	2051	6846	1803	2367	2367
195	1816	24979	1853	2425	7609	1805	2049	6851	1780	2382	2382
253	1850	24959	1885	2445	7567	1819	2090	6817	1828	2389	2389
460	1922	24985	1969	2494	7566	1892	2166	6835	1906	2448	2448
1798		24973	1914	2333	7606	1762	1983	6932	1894	2397	2397
1794	1849	25010	1830	2250	7579	1896	1820	6832	1745	2102	2102
1789	1844	25009	1825	2245	7576	1891	1815	6827	1740	2097	2097
1795	1850	25015	1831	2251	7582	1897	1821	6833	1746	2103	2103
1789	1844	25009	1825	2245	7576	1891	1815	6827	1740	2097	2097
1794	1849	25014	1830	2250	7581	1896	1820	6832	1745	2102	2102
1778	1902	25050	1849	2284	7605	1906	1902	6866	1528	2065	2065

1993	2016	25035	1875	2250	7643	2045	1984	7013	1717	2164	2164
1857	1948	25017	1733	2144	7618	1920	1846	6885	315	2201	2201
1856	1947	25019	1732	2143	7617	1919	1845	6884	314	2200	2200
1849	1937	25014	1725	2136	7612	1912	1838	6874	307	2195	2195
1851	1942	25015	1727	2138	7614	1914	1840	6879	309	2197	2197
1811	1911	25007	1699	2164	7604	1898	1852	6892	230	2166	2166
1820	1919	25015	1708	2173	7613	1907	1861	6901	239	2175	2175
1820	1920	25016	1708	2173	7613	1907	1861	6901	239	2175	2175
1812	1895	25004	1672	2105	7584	1869	1787	6881	75	2141	2141
1843	1924	25016	1697	2122	7587	1904	1806	6896	112	2158	2158
1813	1894	24998	1689	2110	7567	1906	1783	6880		2170	2170
1804	1887	24997	1664	2097	7576	1861	1779	6873	67	2133	2133
1874	1953	25027	1683	2145	7610	1929	1847	6881	383	2166	2166
1874	1953	25027	1683	2145	7610	1929	1847	6881	383	2166	2166
1863	1942	25014	1672	2134	7600	1918	1836	6870	372	2155	2155
1830	1855	25008	1638	2137	7605	1901	1814	6864	259	2179	2179
1862	1817	24977	1735	2145	7578	1793	1862	6944	1465	2054	2054
1893	1848	25005	1766	2176	7606	1824	1893	6972	1496	2085	2085
1864	1819	24979	1737	2147	7580	1795	1864	6946	1467	2056	2056
1862	1817	24977	1735	2145	7578	1793	1862	6944	1465	2054	2054
2221	2250	24973	2186	1869	7635	2186	2031	6971	1903	1981	1981
2223	2252	24975	2188	1872	7639	2188	2033	6973	1905	1983	1983
2217	2246	24971	2182	1866	7633	2182	2027	6967	1899	1977	1977
2214	2243	24966	2179	1863	7630	2179	2024	6964	1896	1974	1974
2202	2246	24971	2161	1895	7639	2181	2052	6975	1900	2006	2006
2212	2243	24968	2177	1871	7630	2177	2022	6968	1894	1982	1982
2216	2245	24970	2181	1865	7632	2181	2026	6966	1898	1976	1976
2225	2258	24966	2190	1870	7633	2188	2035	6977	1887	1989	1989
2216	2245	24970	2181	1865	7632	2181	2026	6966	1898	1976	1976
2215	2244	24968	2180	1864	7631	2180	2025	6965	1897	1975	1975
2228	2237	24969	2177	1837	7622	2129	2010	6966	1898	1930	1930
2233	2240	24962	2184	1864	7643	2226	2029	6951	1957	1968	1968
2236	2275	24997	2187	1870	7642	2216	1973	6970	1900	1985	1985
2236	2275	24997	2187	1870	7642	2216	1973	6970	1900	1985	1985
2236	2275	24997	2187	1870	7642	2216	1973	6970	1900	1985	1985
2272	2235	24963	2144	1599	7696	2108	1865	6955	2032	1462	1462
2236	2138	24986	2051	1668	7688	2008	1902	6955	1949	1585	1585
1977	1968	24973	1825	2048	7589	1889	1369	6839	1758	2190	2190
1969	1946	24972	1805	2028	7588	1875	1347	6843	1742	2174	2174
1969	1946	24974	1805	2028	7588	1875	1347	6843	1742	2174	2174
1956	1924	24982	1797	2016	7585	1862	1333	6839	1726	2162	2162
2034	1983	24982	1897	1956	7616	1892		6960	1783	1996	1996
2246	2275	24958	2155	1854	7651	2168	1963	6960	1991	396	396
2327	2386	24968	2283	1709	7697	2267	1957	6945	2121	151	151
2380	2397	24972	2344	1702	7715	2310	1996	6951	2170		
2380	2397	24972	2344	1702	7715	2310	1996	6951	2170		
2367	2445	24977	2341	1778	7712	2317	2015	6962	2161	237	237
2334	2384	24983	2307	1704	7717	2277	1957	6959	2137	203	203
2377	2326	24966	2291	1609	7690	2285	1902	6950	2101	322	322
2374	2440	24970	2292	1741	7704	2370	2021	6913	2061	745	745
2442	2333	24985	2263		7765	2360	1956	7011	2110	1702	1702
2458	2351	25011	2260	160	7758	2372	1965	7022	2115	1757	1757
1866	1950	24971	1819	2208	7568	1837	1892	6900	1784	2042	2042
1881	1962	24980	1836	2226	7586	1852	1901	6920	1795	2060	2060
1842	1912	24981	1768	2178	7574	1763	1840	6891	1735	1988	1988

1846	1916	24984	1772	2180	7576	1767	1842	6896	1737	1990	1990
1877	1910	24990	1794	2208	7584	1776	1854	6894	1753	2006	2006
1950	1981	24985	1847	2193	7564	1855	1903	6920	1733	2051	2051
1911	1947	24977	1889	2216	7582	1819	1907	6919	1823	2076	2076
1911	1947	24974	1889	2218	7584	1819	1907	6921	1823	2076	2076
1938	1950	24975	1879	2220	7603	1838	1959	6945	1759	2103	2103
1940	1952	24977	1881	2222	7605	1840	1961	6947	1761	2105	2105
1940	1952	24977	1881	2222	7603	1840	1961	6947	1761	2105	2105
1948	1977	24975	1904	2246	7599	1851	1985	6952	1784	2127	2127
1937	1947	24978	1874	2225	7603	1833	1962	6940	1766	2108	2108
1963	1957	24965	1902	2261	7585	1859	1978	6954	1778	2094	2094
1964	1958	24967	1903	2262	7586	1860	1979	6955	1779	2095	2095
1847	1883	25026	1725	2123	7589	1759	1808	6916	1652	2015	2015
1859	1873	25028	1715	2127	7590	1749	1810	6914	1646	2019	2019
1954	1973	25040	1880	2244	7643	1867	1868	6914	1865	2236	2236
1956	1975	25040	1882	2246	7645	1869	1870	6914	1865	2238	2238
1946	1965	25034	1872	2236	7635	1859	1860	6906	1854	2228	2228
1945	1964	25033	1871	2235	7634	1858	1859	6905	1856	2227	2227
1959	1958	25035	1885	2237	7645	1872	1865	6914	1868	2247	2247
1945	1964	25031	1871	2235	7634	1858	1859	6905	1856	2227	2227
1946	1965	25034	1872	2236	7635	1859	1860	6906	1857	2228	2228
1960	1978	25041	1886	2250	7649	1873	1874	6920	1871	2242	2242
1973	2011	25027	1916	2275	7632	1897	1899	6919	1897	2271	2271
2052	1975	25050	1892	2291	7633	1900	1936	6896	1913	2290	2290
2135	2147	25028	544	2491	7679	2091	2146	6934	1952	2464	2464
2141	2156	25036	564	2507	7686	2106	2159	6941	1960	2480	2480
2151	2165	25049	562	2505	7694	2109	2160	6951	1968	2478	2478
2126	2128	25039	539	2470	7670	2064	2123	6935	1943	2433	2433
1943	2001	25044	318	2376	7679	1944	1991	6989	1765	2325	2325
1918	2000	25039	309	2357	7675	1943	1972	6989	1756	2320	2320
1918	2000	25039	309	2357	7675	1943	1972	6989	1756	2320	2320
1916	1998	25037	307	2355	7672	1941	1970	6986	1754	2318	2318
1934	2008	25049	315	2369	7679	1957	1980	6997	1768	2326	2326
1925	1984	25041	299	2354	7660	1919	1973	6995	1752	2303	2303
1914	1974	25057	273	2345	7683	1921	1966	6995	1740	2308	2308
2009	2073	25043	411	2423	7644	2011	2045	6948	1802	2391	2391
1960	2017	25036	320	2382	7662	1968	2023	6984	1793	2367	2367
1908	1970	25055	217	2335	7685	1931	1974	6982	1728	2318	2318
1911	2001	25058	285	2346	7690	1968	1987	7015	1746	2337	2337
1925	1995	25055	242	2354	7692	1958	1993	7009	1753	2337	2337
1930	2000	25060	247	2359	7694	1963	1998	7015	1758	2342	2342
1925	1963	25046	282	2330	7671	1966	1959	6997	1721	2345	2345
1929	1997	25044	262	2354	7683	1962	1979	6996	1751	2331	2331
1920	1992	25042	255	2349	7674	1957	1974	6987	1746	2326	2326
1844	1914	25037		2263	7647	1819	1897	6970	1689	2344	2344
1848	1925	25041	33	2266	7650	1829	1902	6975	1690	2347	2347
2018	2092	25035	299	2460	7607	1966	2076	6889	1874	2517	2517
2027	2101	25043	308	2469	7616	1975	2085	6898	1883	2526	2526
2011	2085	25028	292	2453	7600	1959	2069	6882	1867	2510	2510
1939	2011	25026	256	2340	7643	1870	1980	6947	1776	2415	2415
1852	1934	25040	67	2275	7646	1829	1911	6966	1697	2359	2359
1853	1935	25037	67	2276	7649	1830	1912	6966	1698	2359	2359
2074	2206	25034	494	2527	7553	2067	2144	6881	1938	2611	2611
1952	2036	25018	247	2393	7638	1931	2017	6986	1820	2461	2461
1939	2041	25016	250	2396	7626	1934	2014	6979	1819	2464	2464

1957	2057	25025	268	2407	7637	1944	2024	6984	1829	2475	2475
1952	2060	25033	263	2410	7642	1947	2027	6995	1832	2478	2478
1958	2018	25020	248	2385	7630	1985	2016	6936	1813	2478	2478
1896	1999	25044	149	2347	7654	1896	1970	6993	1766	2434	2434
1889	1992	25039	142	2340	7642	1889	1963	6986	1759	2427	2427
1887	1990	25036	140	2338	7640	1887	1961	6984	1757	2425	2425
1844	1933	25032	64	2255	7643	1839	1893	6970	1707	2336	2336
1845	1928	25027	53	2268	7632	1829	1904	6969	1693	2351	2351
1884	1936	25046	76	2282	7667	1851	1925	6990	1725	2360	2360
1875	1929	25039	67	2275	7660	1844	1916	6983	1716	2355	2355
1814	1856	25028	266	2208	7633	1793	1895	6929	1635	2278	2278
1814	1856	25028	266	2208	7633	1793	1895	6929	1635	2278	2278
1866	1974	25037	114	2315	7648	1865	1937	6975	1738	2398	2398
1861	1969	25032	109	2310	7643	1860	1932	6970	1734	2393	2393
1864	1972	25033	112	2313	7646	1863	1935	6973	1737	2396	2396
1824	1774	24941	1831	2372	7524	32	1904	6848	1918	2322	2322
1825	1775	24940	1832	2373	7521	33	1905	6847	1919	2323	2323
1812	1762	24931	1819	2360	7511		1892	6836	1906	2310	2310
2050	1998	24943	1974	2489	7573	569	2118	6931	2067	2445	2445
1875	1778	24960	1787	2397	7527	378	1981	6854	1922	2299	2299
2147	1935	25173	2103	2566	7768	811	2287	7092	2185	2537	2537
3823	3788	25017	3917	3908	7753	3692	3818	7176	3765	3837	3837
6608	6720	25058	6726	6773	7765	6613	6725	569	6648	6710	6710
6610	6722	25060	6728	6775	7767	6615	6727	571	6650	6712	6712
6607	6719	25056	6725	6772	7764	6612	6724	568	6647	6709	6709
6609	6721	25057	6727	6774	7766	6614	6726	570	6649	6711	6711
6668	6779	25030	6795	6851	7739	6666	6781	442	6709	6780	6780
6658	6766	25032	6782	6835	7745	6659	6767	431	6700	6764	6764
6670	6772	25039	6786	6845	7754	6659	6779	435	6702	6774	6774
6692	6802	25018	6800	6875	7733	6691	6815	373	6740	6806	6806
6706	6816	25032	6814	6891	7748	6705	6829	389	6754	6822	6822
6671	6795	25017	6777	6867	7737	6688	6807	429	6735	6798	6798
6726	6836	25027	6840	6909	7750	6733	6855	378	6772	6848	6848
6684	6794	25014	6792	6869	7724	6675	6799	391	6724	6798	6798
6713	6824	25044	6822	6899	7753	6705	6829	421	6754	6828	6828
6667	6777	25009	6793	6852	7715	6658	6782	392	6707	6781	6781
6688	6798	25016	6790	6873	7726	6677	6801	391	6726	6796	6796
6690	6800	25022	6798	6875	7730	6681	6805	397	6730	6804	6804
6859	6932	25056	6970	7011	7859	6836	6960		6880	6951	6951
6854	6927	25051	6965	7006	7854	6831	6955	23	6875	6946	6946
6820	6916	25042	6924	6969	7832	6805	6924	150	6834	6917	6917
6704	6799	25027	6833	6873	7763	6682	6808	424	6738	6813	6813
6830	6911	25055	6938	7000	7869	6810	6929	511	6866	6923	6923
6686	6798	25022	6814	6881	7749	6685	6794	434	6742	6812	6812
6680	6792	25017	6808	6875	7743	6679	6788	428	6736	6806	6806
6679	6791	25018	6807	6874	7744	6678	6787	431	6735	6805	6805
6650	6767	25009	6776	6846	7702	6652	6761	535	6705	6781	6781
6701	6825	25032	6829	6885	7651	6722	6800	702	6753	6844	6844
3512	3562	25028	3646	3813	7770	3462	3641	7063	3564	3684	3684
3501	3551	25017	3635	3802	7759	3451	3630	7053	3553	3673	3673
7617	7582	25533	7680	7700	3426	7505	7551	8011	7516	7703	7703
7617	7582	25533	7680	7700	3426	7505	7551	8011	7516	7703	7703
7616	7581	25532	7679	7699	3425	7504	7550	8010	7515	7702	7702
7615	7580	25531	7678	7698	3425	7503	7549	8009	7514	7701	7701
7617	7582	25533	7680	7700	3425	7505	7551	8011	7516	7703	7703

7615	7580	25531	7678	7698	3425	7503	7549	8011	7514	7701	7701
7617	7582	25533	7680	7700	3425	7505	7551	8011	7516	7703	7703
7615	7580	25531	7678	7698	3423	7503	7549	8009	7514	7701	7701
7618	7583	25534	7681	7701	3426	7506	7552	8012	7517	7704	7704
7615	7580	25531	7678	7698	3423	7503	7549	8009	7514	7701	7701
7614	7579	25530	7677	7697	3422	7502	7548	8008	7513	7700	7700
7615	7580	25531	7678	7698	3423	7503	7549	8009	7514	7701	7701
7616	7581	25532	7679	7699	3424	7504	7550	8010	7515	7702	7702
7616	7581	25532	7679	7699	3424	7504	7550	8010	7515	7702	7702
7724	7705	25551	7770	7847	3289	7646	7691	8045	7654	7799	7799
7728	7709	25552	7774	7851	3293	7648	7695	8049	7658	7803	7803
7728	7709	25554	7774	7851	3293	7650	7695	8049	7658	7803	7803
7731	7712	25557	7777	7854	3296	7653	7698	8052	7661	7806	7806
7685	7662	25545	7731	7804	3359	7605	7648	8051	7613	7754	7754
7725	7706	25552	7771	7848	3290	7647	7692	8046	7655	7800	7800
7717	7696	25534	7760	7839	3302	7638	7683	8037	7646	7791	7791
7715	7694	25531	7758	7837	3300	7636	7681	8036	7644	7789	7789
7720	7699	25538	7763	7842	3302	7641	7686	8040	7649	7794	7794
7715	7694	25533	7758	7837	3297	7636	7681	8035	7644	7789	7789
7717	7696	25534	7760	7839	3301	7638	7683	8037	7646	7791	7791
7721	7700	25537	7765	7843	3301	7643	7687	8040	7650	7797	7797
7581	7556	25543	7596	7690	3381	7540	7550	7994	7500	7676	7676
7624	7620	25509	7678	7754	3442	7549	7589	7998	7551	7703	7703
7624	7620	25513	7678	7758	3502	7545	7593	7992	7549	7707	7707
7757	7755	25594	7829	7891	3464	7686	7744	8062	7680	7835	7835
7757	7755	25594	7829	7891	3464	7686	7744	8062	7680	7835	7835
7762	7760	25599	7834	7896	3471	7691	7749	8069	7685	7840	7840
7793	7798	25609	7866	7927	3473	7720	7775	8100	7722	7872	7872
7757	7765	25584	7840	7903	3443	7699	7751	8050	7694	7851	7851
7627	7616	25518	7676	7760	2339	7534	7630	7804	7574	7705	7705
7630	7619	25520	7679	7763	2342	7537	7633	7807	7577	7708	7708
7635	7626	25524	7684	7768	2344	7544	7640	7815	7582	7715	7715
7638	7637	25478	7689	7764	2382	7541	7629	7804	7572	7720	7720
7606	7598	25515	7655	7731	2304	7511	7601	7835	7553	7682	7682
7614	7606	25522	7663	7739	2312	7519	7609	7843	7561	7690	7690
7605	7597	25514	7654	7730	2303	7510	7600	7834	7552	7681	7681
7676	7673	25548	7728	7792	2304	7585	7679	7856	7615	7749	7749
7678	7675	25550	7730	7794	2306	7587	7681	7858	7617	7751	7751
7682	7679	25554	7734	7798	2311	7591	7685	7863	7621	7755	7755
7695	7692	25565	7747	7811	2323	7604	7698	7875	7634	7768	7768
7611	7606	25496	7647	7765	4	7511	7616	7859	7567	7715	7715
7612	7607	25497	7648	7766	5	7512	7617	7860	7568	7716	7716
7612	7607	25497	7648	7766	5	7512	7617	7860	7568	7716	7716
7613	7608	25498	7649	7767	6	7513	7618	7861	7569	7717	7717
7611	7606	25496	7647	7765		7511	7616	7859	7567	7715	7715
7613	7608	25504	7649	7767	39	7513	7617	7861	7569	7717	7717
7624	7605	25523	7688	7770	2625	7550	7619	7882	7563	7726	7726
7623	7604	25522	7687	7769	2624	7549	7618	7881	7562	7725	7725
7622	7603	25521	7686	7768	2623	7548	7617	7880	7561	7724	7724
7621	7602	25520	7685	7767	2622	7547	7616	7879	7560	7723	7723
7623	7604	25522	7687	7769	2624	7549	7618	7881	7562	7725	7725
7622	7603	25521	7686	7768	2623	7548	7617	7880	7561	7724	7724
7603	7580	25544	7663	7745	2652	7525	7594	7907	7540	7707	7707
7625	7606	25524	7689	7771	2626	7551	7620	7883	7564	7727	7727
7709	7678	25556	7769	7850	2557	7627	7714	7897	7664	7810	7810

7689	7693	25569	7740	7818	2514	7616	7700	8067	7648	7786	7786
7692	7696	25571	7743	7821	2517	7619	7703	8072	7651	7789	7789
7690	7694	25568	7741	7819	2515	7617	7701	8070	7649	7787	7787
7687	7691	25571	7738	7816	2515	7614	7698	8065	7646	7784	7784
24987	24974	1	25038	24986	25497	24932	24983	25057	24999	24973	24973
24986	24973		25037	24985	25496	24931	24982	25056	24998	24972	24972
31010	30961	27324	31004	30941	31225	30908	30950	30795	30919	30961	30961

[illegible]. Genomes are ordered according to their Sequence Type (ST).

C7277	C7279	C7328	C7347	C7349	C7369	C7382	C7570	C7962	C7963	C7963	C7968	C7969
3658	4192	3760	7646	3584	3737	3742	7556	3549	7692	7692	3407	30934
3576	4161	3678	7573	3516	3671	3690	7499	3536	7643	7643	3365	30906
3576	4159	3680	7573	3516	3671	3690	7501	3534	7643	7643	3365	30906
3617	4186	3706	7599	3560	3729	3739	7529	3568	7666	7666	3401	30939
3588	4167	3707	7632	3535	3714	3711	7547	3552	7680	7680	3378	30890
3616	4189	3719	7589	3558	3746	3740	7526	3583	7637	7637	3404	30900
3616	4189	3719	7589	3558	3746	3740	7526	3583	7637	7637	3404	30900
3631	4200	3735	7607	3574	3736	3728	7546	3589	7680	7680	3418	30927
3630	4204	3738	7613	3578	3738	3730	7552	3591	7682	7682	3422	30931
3674	4241	3752	7584	3613	3771	3767	7498	3617	7627	7627	3444	30930
3721	4228	3756	7586	3635	3779	3775	7506	3642	7650	7650	3519	30935
3646	4226	3736	7615	3595	3758	3760	7547	3610	7672	7672	3441	30917
3759	4290	3840	7671	3706	3844	3856	7604	3691	7723	7723	3543	30934
3759	4290	3840	7671	3706	3844	3856	7604	3691	7723	7723	3543	30934
3689	4225	3778	7623	3616	3774	3786	7555	3626	7677	7677	3467	30927
3687	4222	3775	7627	3613	3771	3783	7560	3623	7681	7681	3463	30935
3555	4169	3670	7587	3494	3662	3670	7522	3514	7644	7644	3333	30907
3610	4202	3705	7606	3541	3715	3721	7540	3553	7668	7668	3382	30912
3681	4227	3740	7604	3587	3760	3786	7539	3603	7681	7681	3436	30921
3706	4269	3783	7582	3612	3764	3787	7531	3664	7651	7651	3437	30933
3705	4267	3804	7618	3625	3805	3815	7554	3668	7700	7700	3484	30969
3644	4165	3684	7611	3533	3650	3662	7522	3544	7648	7648	3432	30914
3702	4224	3735	7605	3625	3756	3774	7521	3620	7660	7660	3502	30950
3719	4234	3750	7636	3642	3790	3800	7550	3647	7683	7683	3528	30954
3610	4140	3658	7589	3499	3663	3683	7503	3532	7634	7634	3409	30910
3666	4164	3703	7663	3570	3707	3710	7577	3572	7700	7700	3455	30913
3597	4190	3685	7646	3508	3642	3646	7584	3530	7707	7707	3388	30918
3712	4196	3783	7650	3661	3753	3791	7583	3662	7718	7718	3512	30941
3708	4214	3772	7588	3604	3752	3739	7522	3642	7668	7668	3503	30915
3637	4167	3690	7611	3539	3700	3685	7527	3568	7668	7668	3439	30910
3637	4167	3690	7611	3539	3700	3685	7527	3568	7668	7668	3439	30910
3640	4169	3693	7613	3542	3702	3687	7530	3570	7670	7670	3442	30912
3622	4155	3708	7647	3556	3672	3694	7564	3592	7694	7694	3403	30928
3597	4186	3680	7638	3510	3626	3636	7576	3532	7703	7703	3394	30908
3597	4186	3680	7638	3510	3626	3636	7576	3532	7703	7703	3394	30908
1993	3512	2215	7709	1862	1934	1866	7638	2052	7690	7690	1795	31010
1984	3507	2184	7708	1855	1961	1891	7637	2043	7695	7695	1786	31012
1970	3497	2190	7708	1839	1945	1875	7637	2023	7694	7694	1768	31017
2007	3479	2181	7712	1884	1952	1882	7644	2062	7697	7697	1807	30987
1968	3489	2184	7695	1840	1927	1843	7621	2025	7681	7681	1772	30991
1976	3489	2196	7706	1853	1953	1883	7637	2034	7694	7694	1780	31013
1975	3488	2195	7705	1852	1952	1882	7636	2033	7693	7693	1779	31012
1952	3469	2194	7702	1841	1932	1858	7633	2022	7690	7690	1756	31007
1988	3503	2211	7702	1867	1963	1897	7627	2051	7685	7685	1794	31008
1986	3478	2244	7678	1906	1997	1929	7586	2062	7665	7665	1824	30999
2021	3560	2297	7653	1965	2075	2012	7582	2132	7641	7641	1870	30998
2016	3562	2244	7678	1817	2008	1974	7637	1975	7694	7694	1850	30961
1691	3508	2125	7674	1616	1879	1876	7566	1807	7639	7639	25	30971
1686	3503	2120	7671	1611	1874	1871	7563	1802	7636	7636	20	30968
1692	3509	2126	7677	1617	1880	1877	7569	1808	7642	7642		30972
1686	3503	2120	7671	1611	1874	1871	7563	1802	7636	7636	20	30966
1691	3508	2125	7676	1616	1879	1876	7568	1807	7641	7641	27	30973
1618	3429	2074	7705	1655	1831	1908	7613	1953	7693	7693	1448	30973

	3541	2261	7737	1838	1958	1927	7673	2009	7731	7731	1692	31031
1751	3535	1929	7702	1473	1826	1782	7617	1945	7701	7701	1744	30944
1750	3534	1928	7701	1472	1825	1781	7617	1944	7700	7700	1743	30944
1745	3527	1921	7696	1467	1818	1774	7610	1937	7695	7695	1736	30935
1747	3529	1923	7698	1469	1820	1776	7612	1939	7697	7697	1738	30938
1738	3534	1914	7701	1421	1772	1748	7612	1944	7690	7690	1697	30928
1747	3543	1923	7710	1430	1781	1757	7621	1953	7699	7699	1706	30936
1747	3543	1923	7710	1430	1781	1757	7621	1953	7699	7699	1706	30939
1710	3546	1867	7680	1424	1743	1721	7590	1908	7665	7665	1709	30924
1735	3567	1884	7691	1455	1768	1746	7599	1931	7676	7676	1730	30937
1717	3564	1897	7664	1465	1768	1738	7572	1913	7649	7649	1746	30919
1702	3538	1859	7672	1416	1735	1713	7582	1900	7657	7657	1701	30920
1788	3618	1909	7700	1514	1754	1732	7611	1920	7689	7689	1740	30936
1788	3618	1909	7700	1514	1754	1732	7611	1920	7689	7689	1740	30936
1777	3608	1898	7690	1503	1743	1721	7603	1909	7679	7679	1729	30922
1770	3589	1924	7696	1512	1711	1687	7610	1888	7687	7687	1682	30937
1838	3562	2108	7667	4	1842	1780	7600	1920	7663	7663	1617	30946
1869	3593	2139	7696	35	1873	1811	7629	1951	7692	7692	1648	30973
1840	3564	2110	7669	6	1844	1782	7602	1922	7665	7665	1619	30948
1838	3562	2108	7667		1842	1780	7600	1920	7663	7663	1617	30946
2266	3812	10	7729	2114	2242	2248	7632	2161	7702	7702	2132	30948
2269	3814	12	7731	2116	2245	2250	7634	2163	7704	7704	2134	30951
2263	3808	6	7725	2110	2239	2244	7628	2157	7698	7698	2128	30947
2260	3805	3	7722	2107	2236	2241	7625	2154	7695	7695	2125	30944
2262	3799	47	7733	2089	2218	2231	7634	2148	7706	7706	2115	30944
2258	3803	11	7724	2105	2234	2239	7627	2152	7697	7697	2123	30944
2262	3807	5	7724	2109	2238	2243	7627	2156	7697	7697	2127	30946
2277	3814	20	7725	2124	2243	2250	7628	2169	7698	7698	2142	30948
2262	3807	5	7724	2109	2238	2243	7627	2156	7697	7697	2127	30945
2261	3806		7723	2108	2237	2242	7626	2155	7696	7696	2126	30945
2194	3789	77	7724	2113	2248	2239	7623	2130	7697	7697	2133	30938
2287	3791	92	7731	2074	2229	2234	7639	2193	7708	7708	2174	30952
2186	3790	121	7707	2139	2244	2249	7618	2103	7682	7682	2033	30969
2186	3790	121	7707	2139	2244	2249	7618	2103	7682	7682	2033	30969
2186	3790	121	7707	2139	2244	2249	7618	2103	7682	7682	2033	30969
2226	3673	1677	7795	1932	2191	2190	7704	2100	7774	7774	2106	30950
2151	3696	1591	7792	1864	2124	2105	7699	2063	7776	7776	2089	30955
2100	3649	1906	7690	1927	1849	1875	7621	2048	7706	7706	1884	30950
2086	3637	1886	7689	1909	1845	1871	7620	2028	7702	7702	1866	30956
2086	3637	1886	7689	1909	1845	1871	7620	2028	7702	7702	1866	30957
2071	3620	1886	7685	1889	1835	1861	7616	2034	7698	7698	1852	30961
1984	3641	2025	7714	1862	1980	1937	7629	1936	7701	7701	1821	30950
2089	3687	1877	7753	1892	2181	2209	7665	2253	7730	7730	2056	30967
2161	3688	1998	7791	2013	2299	2337	7712	2265	7773	7773	2114	30967
2164	3684	1975	7810	2054	2326	2398	7720	2290	7787	7787	2103	30961
2164	3684	1975	7810	2054	2326	2398	7720	2290	7787	7787	2103	30961
2205	3655	2056	7812	2075	2343	2397	7725	2355	7790	7790	2144	30968
2122	3654	2010	7813	2008	2309	2363	7727	2324	7797	7797	2106	30966
2174	3688	1908	7783	2057	2291	2353	7687	2218	7755	7755	2108	30947
2154	3711	1919	7787	2148	2304	2347	7696	2256	7780	7780	2083	30948
2250	3813	1864	7850	2145	2369	2315	7764	2291	7819	7819	2251	30941
2260	3817	1917	7843	2148	2346	2308	7755	2300	7816	7816	2284	30934
1789	3361	2099	7688	1846	1942	1860	7604	1895	7645	7645	1796	31006
1804	3376	2119	7709	1855	1955	1873	7625	1906	7665	7665	1805	31020
1735	3338	2045	7695	1776	1881	1807	7610	1851	7654	7654	1736	31009

1737	3340	2047	7697	1778	1885	1811	7612	1853	7656	7656	1740	31010
1768	3371	2079	7706	1802	1893	1833	7620	1861	7662	7662	1764	31010
1858	3334	2091	7672	1745	1932	1865	7577	1913	7626	7626	1889	30986
1856	3353	2136	7701	1827	1976	1932	7611	1926	7652	7652	1819	31001
1856	3352	2136	7703	1827	1976	1932	7613	1928	7654	7654	1819	30997
1882	3289	2081	7700	1829	1994	1924	7622	1959	7657	7657	1815	31007
1884	3291	2083	7702	1831	1996	1926	7624	1961	7659	7659	1817	31007
1884	3291	2083	7700	1831	1996	1926	7622	1961	7657	7657	1817	31007
1905	3298	2104	7690	1857	2006	1936	7618	1987	7669	7669	1839	31009
1887	3286	2078	7700	1836	1995	1925	7622	1964	7660	7660	1822	31002
1893	3261	2072	7682	1878	2001	1959	7599	1970	7642	7642	1816	31004
1894	3262	2073	7683	1879	2002	1960	7600	1971	7643	7643	1817	31007
1854	3384	2082	7714	1623	1810	1765	7632	1927	7677	7677	1776	31002
1848	3378	2072	7715	1619	1820	1775	7633	1921	7681	7681	1772	31001
1964	3557	2116	7722	1866	1942	1930	7652	181	7691	7691	1751	30983
1966	3559	2116	7724	1868	1944	1932	7654	183	7693	7693	1753	30983
1955	3549	2107	7714	1858	1934	1922	7644	175	7683	7683	1742	30974
1955	3548	2107	7713	1857	1933	1921	7643	174	7682	7682	1742	30975
1969	3546	2135	7726	1877	1953	1933	7658	198	7695	7695	1762	30975
1955	3548	2107	7713	1857	1933	1921	7643	174	7682	7682	1742	30977
1956	3549	2108	7714	1858	1934	1922	7644	173	7683	7683	1743	30975
1970	3563	2122	7728	1872	1948	1936	7656	189	7695	7695	1757	30986
2002	3585	2154	7707	1903	1935	1929	7641	224	7698	7698	1788	30973
2009	3572	2155	7708	1920	1956	1942	7636		7675	7675	1808	30999
2137	3729	2365	7814	2005	337	594	7727	2105	7796	7796	2067	31016
2143	3730	2379	7821	2020	361	614	7732	2110	7803	7803	2072	31030
2153	3745	2383	7829	2021	351	608	7742	2121	7813	7813	2083	31031
2116	3696	2346	7807	1966	364	609	7718	2090	7791	7791	2038	31025
1961	3659	2222	7799	1843	81	354	7718	1965	7782	7782	1881	31011
1958	3652	2229	7795	1838	52	315	7716	1958	7773	7773	1874	31009
1958	3652	2229	7795	1838	52	315	7716	1958	7773	7773	1874	31009
1956	3650	2227	7792	1836	50	313	7713	1956	7770	7770	1872	31006
1958	3668	2237	7799	1842		319	7720	1956	7777	7777	1880	31008
1934	3630	2213	7774	1801	95	352	7699	1941	7774	7774	1849	31011
1930	3634	2203	7801	1814	87	339	7724	1930	7783	7783	1852	31016
2015	3670	2283	7770	1915	238	479	7680	2001	7747	7747	1934	30999
1975	3636	2252	7781	1921	283	384	7692	1965	7755	7755	1905	31001
1930	3626	2199	7804	1828	170	281	7722	1926	7781	7781	1812	31012
1929	3627	2222	7807	1850	226	338	7725	1954	7795	7795	1819	31016
1959	3647	2224	7807	1847	183	295	7727	1949	7797	7797	1837	31010
1964	3652	2229	7809	1852	188	300	7729	1954	7799	7799	1842	31018
1937	3647	2216	7789	1879	237	325	7708	1950	7780	7780	1857	31008
1965	3651	2226	7802	1847	133	272	7720	1953	7780	7780	1835	31005
1960	3644	2223	7793	1842	122	261	7711	1946	7771	7771	1830	30994
1875	3646	2180	7769	1735	315	114	7689	1892	7741	7741	1831	31004
1878	3655	2191	7772	1736	316	115	7692	1895	7743	7743	1832	31010
2058	3747	2389	7769	1948	510	323	7643	2046	7745	7745	2028	31008
2067	3756	2398	7778	1957	519	332	7652	2055	7754	7754	2037	31016
2051	3740	2382	7762	1941	503	316	7636	2039	7738	7738	2021	31001
1968	3702	2251	7771	1808	463	280	7679	1971	7746	7746	1914	31023
1885	3650	2201	7767	1745	326	109	7690	1903	7744	7744	1837	31009
1886	3653	2201	7770	1746	326	109	7691	1903	7745	7745	1838	31009
2117	3747	2457	7669	2047	736	543	7570	2139	7653	7653	2125	30989
1987	3666	2312	7756	1878	496	259	7676	1994	7728	7728	1958	30996
1988	3667	2317	7744	1875	485	248	7664	1993	7713	7713	1955	30995

1998	3676	2328	7753	1883	503	266	7673	2003	7724	7724	1965	30998
2001	3680	2331	7760	1888	498	261	7680	2006	7729	7729	1968	31009
2017	3732	2301	7746	1869	492	247	7667	2027	7738	7738	1964	30994
1954	3703	2270	7774	1808	402	163	7694	1973	7742	7742	1904	31012
1947	3696	2263	7762	1798	395	142	7682	1966	7730	7730	1897	31001
1941	3694	2261	7760	1796	393	140	7680	1964	7728	7728	1895	30996
1893	3664	2184	7764	1747	323	107	7685	1912	7735	7735	1847	31007
1878	3656	2195	7747	1741	319	114	7672	1899	7740	7740	1834	31004
1911	3682	2197	7787	1770	359	140	7710	1928	7761	7761	1865	31001
1902	3677	2190	7782	1763	350	131	7703	1921	7756	7756	1858	30996
1887	3610	2102	7748	1719	497	342	7677	1816	7732	7732	1831	31008
1887	3610	2102	7748	1719	497	342	7677	1816	7732	7732	1831	31008
1927	3678	2242	7769	1780	319		7687	1942	7744	7744	1877	30995
1922	3674	2237	7764	1775	314	11	7682	1937	7739	7739	1872	30988
1925	3677	2240	7767	1778	317	14	7685	1940	7742	7742	1875	30991
2057	3474	2192	7640	1805	1969	1877	7553	1912	7630	7630	1909	30923
2058	3475	2193	7637	1806	1970	1878	7550	1913	7627	7627	1910	30914
2045	3462	2180	7627	1793	1957	1865	7541	1900	7617	7617	1897	30908
2272	3689	2353	7702	1964	2064	2008	7620	2158	7673	7673	2150	30933
2103	3529	2177	7636	1807	1929	1835	7551	1963	7629	7629	2009	30936
2391	3755	2450	7856	2086	2199	2149	7793	2138	7848	7848	2240	31149
3847	4449	3834	7800	3812	3900	3924	7733	3807	7805	7805	3666	31054
6762	6896	6755	7817	6712	6755	6729	7727	6707	7984	7984	6592	30819
6764	6898	6757	7819	6714	6757	6731	7729	6709	7986	7986	6594	30821
6761	6895	6754	7816	6711	6754	6728	7726	6706	7983	7983	6591	30817
6763	6897	6756	7818	6713	6756	6730	7728	6708	7985	7985	6593	30820
6825	6965	6818	7794	6782	6817	6793	7688	6763	7976	7976	6668	30807
6814	6958	6807	7804	6770	6807	6781	7694	6749	7968	7968	6658	30799
6816	6958	6811	7815	6778	6821	6795	7703	6757	7974	7974	6662	30809
6852	6930	6847	7793	6808	6827	6803	7683	6793	7954	7954	6692	30797
6866	6944	6861	7808	6822	6841	6817	7700	6807	7971	7971	6706	30811
6845	6935	6837	7797	6799	6808	6782	7687	6780	7958	7958	6685	30810
6894	6948	6875	7808	6846	6867	6843	7698	6831	7971	7971	6730	30804
6836	6920	6833	7788	6800	6819	6795	7676	6777	7947	7947	6682	30795
6866	6950	6863	7817	6830	6849	6825	7705	6807	7976	7976	6712	30825
6819	6919	6814	7780	6783	6820	6796	7667	6760	7936	7936	6665	30791
6838	6916	6829	7788	6798	6817	6793	7678	6779	7949	7949	6686	30797
6842	6926	6839	7792	6806	6825	6801	7682	6783	7953	7953	6688	30801
7013	7063	6965	7897	6944	6997	6975	7804	6896	8070	8070	6833	30795
7008	7058	6960	7892	6939	6992	6970	7799	6891	8065	8065	6828	30788
6967	7017	6931	7879	6914	6951	6929	7779	6885	8049	8049	6795	30780
6840	6940	6837	7824	6814	6860	6836	7706	6768	7987	7987	6704	30779
6960	7021	6950	7908	6924	6963	6941	7811	6873	8073	8073	6808	30813
6831	6914	6836	7799	6809	6841	6817	7681	6775	7964	7964	6694	30792
6825	6908	6830	7793	6803	6835	6811	7675	6769	7958	7958	6688	30787
6824	6905	6827	7794	6802	6834	6810	7676	6768	7959	7959	6687	30790
6796	6907	6803	7754	6776	6803	6779	7641	6745	7921	7921	6674	30787
6844	6959	6849	7683	6817	6843	6827	7572	6801	7851	7851	6714	30819
3541		3806	7865	3562	3668	3678	7774	3572	7896	7896	3509	30974
3530	25	3795	7852	3551	3657	3667	7761	3561	7883	7883	3498	30966
7619	7792	7575	3525	7542	7720	7678	3434	7652	3414	3414	7575	31138
7619	7792	7575	3525	7542	7720	7678	3434	7652	3414	3414	7575	31138
7618	7791	7574	3524	7541	7719	7677	3433	7651	3413	3413	7574	31137
7617	7790	7573	3524	7540	7718	7676	3433	7650	3413	3413	7573	31136
7619	7790	7575	3524	7542	7720	7678	3433	7652	3413	3413	7575	31138

7617	7790	7573	3522	7540	7718	7676	3431	7650	3411	3411	7573	31136
7619	7792	7575	3524	7542	7720	7678	3433	7652	3413	3413	7575	31138
7617	7790	7573	3522	7540	7718	7676	3431	7650	3411	3411	7573	31136
7620	7793	7576	3525	7543	7721	7679	3434	7653	3414	3414	7576	31139
7617	7790	7573	3522	7540	7718	7676	3431	7650	3411	3411	7573	31136
7616	7789	7572	3521	7539	7717	7675	3430	7649	3410	3410	7572	31135
7617	7790	7573	3522	7540	7718	7676	3431	7650	3411	3411	7573	31136
7618	7791	7574	3523	7541	7719	7677	3432	7651	3412	3412	7574	31137
7618	7791	7574	3523	7541	7719	7677	3432	7651	3412	3412	7574	31137
7757	7875	7712	3403	7675	7806	7768	3309	7684	3292	3292	7649	31177
7761	7879	7716	3407	7679	7810	7772	3313	7688	3296	3296	7653	31182
7761	7878	7716	3407	7679	7810	7772	3313	7688	3296	3296	7653	31182
7764	7881	7719	3410	7682	7813	7775	3316	7691	3299	3299	7656	31185
7712	7847	7673	3478	7626	7767	7729	3377	7649	3358	3358	7606	31166
7758	7876	7713	3404	7676	7807	7769	3310	7685	3293	3293	7650	31177
7747	7870	7706	3386	7666	7796	7758	3308	7676	3291	3291	7641	31164
7745	7868	7704	3384	7664	7794	7756	3306	7674	3289	3289	7639	31161
7750	7873	7709	3388	7669	7799	7761	3310	7679	3293	3293	7644	31163
7745	7868	7704	3383	7664	7794	7756	3305	7674	3288	3288	7639	31162
7747	7870	7706	3387	7666	7796	7758	3307	7676	3290	3290	7641	31163
7751	7874	7710	3385	7672	7801	7763	3307	7682	3292	3292	7645	31166
7622	7797	7563	3493	7528	7642	7594	3393	7575	3375	3375	7508	31134
7649	7776	7621	3620	7600	7724	7680	3507	7624	3481	3481	7554	31149
7651	7784	7625	3672	7600	7724	7680	3536	7620	3524	3524	7550	31159
7815	7894	7756	3478	7733	7876	7831	3454	7727	3316	3316	7706	31273
7815	7894	7756	3478	7733	7876	7831	3454	7727	3316	3316	7706	31273
7820	7899	7761	3486	7738	7881	7836	3462	7732	3324	3324	7711	31276
7857	7938	7791	3495	7766	7911	7868	3478	7767	3320	3320	7745	31290
7825	7930	7779	3489	7724	7883	7842	3419	7755	3338	3338	7705	31215
7670	7766	7619	2640	7596	7707	7674	406	7631	2460	2460	7567	31211
7673	7769	7622	2643	7599	7710	7677	409	7634	2463	2463	7570	31216
7680	7779	7631	2647	7606	7715	7682	397	7639	2465	2465	7577	31213
7673	7774	7626	2683	7600	7720	7687		7636	2467	2467	7569	31187
7652	7758	7603	2536	7575	7688	7654	502	7605	2357	2357	7551	31199
7660	7766	7611	2544	7583	7696	7662	510	7613	2365	2365	7559	31206
7651	7757	7602	2535	7574	7687	7653	501	7604	2356	2356	7550	31198
7709	7825	7679	2601	7645	7761	7729	803	7679	2449	2449	7611	31203
7711	7827	7681	2603	7647	7763	7731	805	7681	2451	2451	7613	31207
7715	7831	7685	2608	7651	7767	7735	810	7685	2456	2456	7617	31209
7728	7844	7698	2620	7664	7780	7748	822	7698	2468	2468	7630	31219
7643	7770	7631	2557	7578	7679	7648	2382	7633	2515	2515	7582	31225
7644	7771	7632	2558	7579	7680	7649	2383	7634	2516	2516	7583	31226
7644	7771	7632	2558	7579	7680	7649	2383	7634	2516	2516	7583	31226
7645	7772	7633	2559	7580	7681	7650	2384	7635	2517	2517	7584	31227
7643	7770	7631	2557	7578	7679	7648	2382	7633	2515	2515	7582	31225
7645	7772	7633	2558	7580	7681	7650	2380	7635	2517	2517	7584	31227
7672	7824	7654	2661	7588	7716	7684	2656	7634	2600	2600	7589	31234
7671	7823	7653	2660	7587	7715	7683	2655	7633	2599	2599	7588	31233
7670	7822	7652	2659	7586	7714	7682	2654	7632	2598	2598	7587	31232
7669	7821	7651	2658	7585	7713	7681	2653	7631	2597	2597	7586	31231
7671	7823	7653	2660	7587	7715	7683	2655	7633	2599	2599	7588	31233
7670	7822	7652	2659	7586	7714	7682	2654	7632	2598	2598	7587	31230
7651	7853	7629	2691	7563	7691	7659	2686	7609	2629	2629	7566	31248
7673	7825	7655	2662	7589	7717	7685	2657	7635	2601	2601	7590	31233
7737	7865	7723		7667	7799	7769	2683	7708	2613	2613	7677	31220

7730	7895	7695	2612	7662	7776	7743	2466	7674	25	25	7641	31237
7733	7897	7698	2615	7665	7779	7746	2469	7677	28	28	7644	31239
7731	7896	7696	2613	7663	7777	7744	2467	7675			7642	31234
7728	7893	7693	2613	7660	7774	7741	2467	7672	41	41	7639	31235
25036	25029	24969	25557	24978	25050	25038	25479	25051	25569	25569	25016	27325
25035	25028	24968	25556	24977	25049	25037	25478	25050	25568	25568	25015	27324
31031	30974	30945	31220	30946	31008	30995	31187	30999	31234	31234	30972	

C7970	C7971	C7973	C7974	C7975	C7979	C8124	KCh007	HE-MDREc53	KFu023	KFu031
7607	7754	987	987	7577	7703	3753	3727	3761	1064	7509
7556	7688	1129	1129	7509	7620	3676	3673	3679	597	7460
7556	7690	1131	1131	7511	7620	3676	3673	3681	599	7460
7599	7731	1184	1184	7534	7663	3727	3719	3707	676	7502
7609	7739	1148	1148	7556	7683	3699	3704	3708	616	7511
7576	7704	1153	1153	7515	7650	3732	3738	3720	576	7474
7576	7704	1153	1153	7515	7650	3732	3738	3720	576	7474
7584	7716	1147	1147	7550	7660	3723	3732	3736	553	7486
7588	7722	1149	1149	7556	7664	3725	3734	3739	555	7490
7556	7693	1231	1231	7502	7646	3782	3763	3753	629	7491
7549	7690	1115	1115	7512	7634	3771	3773	3757	734	7486
7588	7728	1157	1157	7550	7670	3767	3760	3737	538	7499
7653	7795	1201	1201	7611	7737	3859	3840	3841	675	7507
7653	7795	1201	1201	7611	7737	3859	3840	3841	675	7507
7605	7745	1172	1172	7561	7689	3788	3770	3779	516	7455
7609	7749	1162	1162	7567	7693	3787	3767	3776	514	7461
7571	7705	1078	1078	7528	7650	3704	3654	3671	450	7466
7587	7731	1149	1149	7558	7673	3731	3709	3706	530	7517
7580	7719	1138	1138	7563	7664	3794	3757	3741	378	7499
7569	7706	1319	1319	7549	7655	3738	3761	3784		7463
7602	7742	1161	1161	7572	7688	3815	3799	3805	582	7520
7587	7719	1167	1167	7547	7661	3677	3640	3685	977	7480
7597	7742	1069	1069	7528	7669	3759	3744	3736	795	7529
7612	7753	1082	1082	7554	7690	3784	3782	3751	803	7446
7558	7698	975	975	7516	7633	3699	3651	3659	758	7474
7613	7752	1014	1014	7577	7687	3696	3701	3704	776	7534
7626	7768	62	62	7592	7713	3673	3636	3686	1326	7536
7624	7785	547	547	7592	7720	3754	3745	3784	1453	7550
7556	7702	692	692	7548	7644	3780	3744	3773	1136	7448
7570	7724	625	625	7543	7659	3707	3694	3691	1053	7472
7570	7724	625	625	7543	7659	3707	3694	3691	1053	7472
7573	7727	628	628	7546	7662	3709	3696	3694	1056	7475
7595	7759	548	548	7569	7686	3704	3666	3709	1092	7511
7617	7759			7580	7706	3669	3626	3681	1319	7529
7617	7759			7580	7706	3669	3626	3681	1319	7529
7717	7793	3474	3474	7625	7757	1952	1918	2216	3611	7624
7715	7792	3468	3468	7620	7754	1959	1947	2185	3624	7621
7716	7796	3476	3476	7622	7752	1956	1937	2191	3621	7621
7714	7798	3403	3403	7627	7753	1963	1940	2182	3598	7623
7700	7782	3449	3449	7605	7740	1922	1915	2185	3606	7608
7711	7793	3466	3466	7619	7754	1934	1941	2197	3620	7624
7712	7794	3465	3465	7618	7753	1933	1940	2196	3619	7625
7711	7797	3444	3444	7617	7756	1965	1920	2195	3608	7622
7714	7790	3413	3413	7614	7753	1966	1949	2212	3586	7611
7687	7772	3438	3438	7594	7737	1942	1985	2245	3590	7597
7666	7748	3418	3418	7565	7713	2011	2063	2298	3562	7575
7696	7798	3530	3530	7606	7765	2036	2000	2245	3669	7620
7636	7742	3393	3393	7587	7702	1957	1873	2126	3436	7549
7635	7739	3388	3388	7584	7699	1952	1868	2121	3431	7548
7641	7745	3394	3394	7590	7705	1958	1874	2127	3437	7554
7635	7739	3388	3388	7584	7699	1952	1868	2121	3431	7548
7640	7744	3393	3393	7589	7704	1957	1873	2126	3436	7553
7695	7812	3518	3518	7626	7778	1970	1827	2075	3606	7607

7747	7857	3597	3597	7673	7825	1987	1958	2262	3706	7649
7692	7761	3481	3481	7627	7742	1886	1814	1930	3589	7598
7690	7759	3480	3480	7626	7739	1885	1813	1929	3588	7598
7687	7754	3475	3475	7621	7734	1878	1806	1922	3583	7593
7689	7756	3477	3477	7623	7736	1880	1808	1924	3585	7595
7678	7745	3548	3548	7607	7722	1852	1760	1915	3644	7585
7687	7754	3557	3557	7616	7731	1861	1769	1924	3652	7594
7687	7754	3557	3557	7616	7731	1861	1769	1924	3653	7594
7661	7731	3508	3508	7582	7707	1825	1731	1868	3612	7568
7673	7744	3531	3531	7589	7719	1850	1756	1885	3635	7580
7646	7722	3470	3470	7564	7694	1820	1756	1898	3623	7551
7653	7723	3500	3500	7574	7699	1817	1723	1860	3604	7560
7686	7764	3531	3531	7605	7742	1812	1742	1910	3633	7596
7686	7764	3531	3531	7605	7742	1812	1742	1910	3633	7596
7676	7754	3520	3520	7594	7732	1801	1731	1899	3622	7586
7669	7748	3525	3525	7592	7720	1781	1699	1925	3635	7584
7666	7766	3510	3510	7589	7724	1878	1838	2109	3612	7600
7695	7793	3541	3541	7618	7751	1909	1869	2140	3643	7629
7668	7768	3512	3512	7591	7726	1880	1840	2111	3614	7602
7666	7766	3510	3510	7589	7724	1878	1838	2109	3612	7600
7712	7797	3685	3685	7661	7785	2318	2234	11	3788	7627
7714	7799	3688	3688	7663	7787	2320	2237	13	3791	7629
7708	7793	3682	3682	7657	7781	2314	2231	7	3785	7623
7705	7790	3679	3679	7654	7778	2311	2228	4	3782	7620
7716	7801	3689	3689	7663	7789	2302	2210	48	3764	7629
7707	7790	3677	3677	7656	7780	2309	2226	12	3780	7620
7707	7792	3681	3681	7656	7780	2313	2230	6	3784	7622
7712	7797	3688	3688	7657	7785	2322	2229	21	3791	7627
7707	7792	3681	3681	7656	7780	2313	2230		3784	7622
7706	7791	3680	3680	7655	7779	2312	2229	5	3783	7621
7709	7790	3655	3655	7656	7776	2312	2240	78	3787	7612
7716	7807	3678	3678	7669	7791	2306	2221	93	3779	7633
7699	7783	3681	3681	7638	7771	2319	2236	122	3728	7614
7699	7783	3681	3681	7638	7771	2319	2236	122	3728	7614
7699	7783	3681	3681	7638	7771	2319	2236	122	3728	7614
7767	7844	3663	3663	7716	7826	2234	2187	1678	3785	7674
7764	7844	3639	3639	7717	7826	2169	2120	1592	3806	7667
7683	7780	3564	3564	7628	7775	1976	1835	1907	3636	7599
7684	7777	3558	3558	7626	7772	1964	1829	1887	3633	7596
7684	7777	3558	3558	7626	7772	1964	1829	1887	3633	7596
7682	7777	3565	3565	7623	7769	1956	1819	1887	3619	7594
7683	7775	3487	3487	7620	7751	2017	1972	2026	3596	7589
7747	7834	3584	3584	7685	7812	2270	2169	1878	3702	7658
7784	7866	3638	3638	7709	7842	2398	2287	1999	3750	7696
7791	7872	3631	3631	7727	7851	2461	2320	1976	3739	7703
7791	7872	3631	3631	7727	7851	2461	2320	1976	3739	7703
7795	7885	3634	3634	7728	7859	2453	2329	2057	3725	7705
7797	7885	3607	3607	7727	7864	2416	2295	2011	3724	7709
7763	7831	3640	3640	7695	7815	2414	2281	1909	3756	7675
7758	7850	3693	3693	7709	7837	2409	2292	1920	3828	7680
7839	7927	3639	3639	7771	7903	2393	2357	1865	3796	7754
7838	7929	3629	3629	7763	7905	2390	2334	1918	3796	7757
7672	7776	3546	3546	7579	7749	1857	1936	2100	3642	7585
7693	7795	3562	3562	7600	7770	1873	1951	2120	3658	7601
7686	7778	3543	3543	7590	7759	1885	1877	2046	3613	7592

7688	7780	3543	3543	7592	7761	1887	1881	2048	3613	7594
7691	7783	3535	3535	7596	7764	1909	1889	2080	3613	7597
7662	7763	3560	3560	7570	7735	1944	1928	2092	3611	7571
7688	7787	3536	3536	7599	7762	2018	1970	2137	3635	7596
7690	7789	3536	3536	7601	7764	2018	1970	2137	3635	7598
7688	7787	3599	3599	7614	7767	1994	1990	2082	3646	7600
7690	7789	3601	3601	7616	7769	1996	1992	2084	3648	7602
7688	7787	3601	3601	7616	7767	1996	1992	2084	3648	7600
7674	7775	3612	3612	7620	7755	2007	2000	2105	3630	7588
7686	7789	3592	3592	7613	7769	1993	1987	2079	3646	7602
7674	7773	3572	3572	7597	7751	2021	1993	2073	3626	7584
7675	7772	3573	3573	7598	7752	2022	1994	2074	3627	7585
7702	7811	3548	3548	7610	7786	1876	1800	2083	3614	7622
7703	7812	3544	3544	7608	7787	1878	1812	2073	3615	7623
7703	7786	3515	3515	7657	7776	1982	1944	2117	3646	7617
7705	7788	3515	3515	7659	7778	1984	1946	2117	3646	7619
7695	7778	3507	3507	7649	7768	1974	1936	2108	3638	7609
7694	7777	3506	3506	7648	7767	1973	1935	2108	3637	7608
7703	7788	3514	3514	7659	7774	1961	1955	2136	3643	7619
7694	7777	3506	3506	7648	7767	1973	1935	2108	3637	7608
7695	7778	3507	3507	7649	7768	1974	1936	2109	3638	7609
7707	7790	3521	3521	7661	7780	1988	1950	2123	3652	7621
7684	7771	3501	3501	7653	7757	2012	1937	2155	3605	7602
7676	7767	3532	3532	7635	7755	1994	1958	2156	3664	7624
7816	7933	3706	3706	7717	7890	733	331	2366	3878	7738
7821	7944	3718	3718	7724	7897	739	355	2380	3889	7735
7831	7948	3718	3718	7734	7905	751	347	2384	3894	7753
7805	7922	3727	3727	7719	7881	728	358	2347	3903	7727
7802	7915	3603	3603	7716	7887	505	81	2223	3763	7728
7796	7909	3626	3626	7713	7883	486	2	2230	3761	7722
7796	7909	3626	3626	7713	7883	486		2230	3761	7722
7793	7906	3624	3624	7710	7880	484	16	2228	3759	7719
7796	7911	3626	3626	7717	7883	496	52	2238	3764	7724
7770	7883	3652	3652	7715	7857	484	99	2214	3767	7696
7801	7914	3646	3646	7727	7888	462	90	2204	3790	7727
7772	7879	3713	3713	7690	7847	600	236	2284	3850	7683
7775	7889	3595	3595	7704	7857	428	277	2253	3774	7687
7798	7909	3652	3652	7726	7880	402	164	2200	3790	7721
7799	7914	3686	3686	7744	7884	450	227	2223	3780	7722
7803	7909	3671	3671	7744	7882	425	184	2225	3766	7722
7807	7914	3676	3676	7746	7884	430	189	2230	3771	7726
7782	7895	3673	3673	7727	7861	435	238	2217	3766	7681
7802	7911	3633	3633	7720	7883	439	129	2227	3767	7723
7791	7902	3622	3622	7711	7874	436	118	2224	3758	7714
7760	7866	3637	3637	7689	7840	247	309	2181	3783	7678
7763	7869	3645	3645	7695	7843	254	314	2192	3784	7681
7740	7864	3747	3747	7669	7826	386	498	2390	3861	7702
7749	7873	3756	3756	7678	7835	395	507	2399	3870	7711
7733	7857	3740	3740	7662	7819	379	491	2383	3854	7695
7759	7869	3694	3694	7677	7842	419	451	2252	3819	7684
7756	7866	3645	3645	7694	7840	256	318	2202	3785	7678
7759	7869	3650	3650	7695	7841	258	318	2202	3788	7681
7634	7779	3661	3661	7630	7747	586	732	2458	3822	7577
7761	7875	3669	3669	7673	7837		486	2313	3738	7690
7751	7863	3666	3666	7664	7829	23	479	2318	3730	7682

7760	7872	3685	3685	7675	7838	47	497	2329	3746	7691
7767	7879	3680	3680	7680	7845	42	492	2332	3743	7698
7741	7865	3713	3713	7678	7821	193	486	2302	3836	7696
7784	7888	3669	3669	7696	7856	180	396	2271	3821	7704
7774	7878	3659	3659	7684	7846	155	389	2264	3814	7694
7772	7876	3657	3657	7682	7844	153	387	2262	3810	7692
7755	7859	3627	3627	7687	7835	247	313	2185	3794	7673
7735	7841	3645	3645	7684	7815	252	315	2196	3760	7653
7780	7886	3659	3659	7706	7860	267	353	2198	3819	7695
7773	7879	3652	3652	7699	7853	258	344	2191	3812	7688
7745	7848	3653	3653	7674	7826	475	491	2103	3780	7660
7745	7848	3653	3653	7674	7826	475	491	2103	3780	7660
7758	7868	3636	3636	7685	7842	259	315	2243	3787	7680
7753	7863	3631	3631	7680	7837	254	310	2238	3782	7675
7756	7866	3634	3634	7683	7840	257	313	2241	3785	7678
7651	7733	3492	3492	7564	7712	1943	1955	2193	3550	7562
7648	7728	3493	3493	7561	7707	1944	1956	2194	3552	7559
7638	7720	3480	3480	7551	7699	1931	1943	2181	3540	7549
7734	7807	3644	3644	7617	7753	2081	2054	2354	3698	7651
7663	7731	3510	3510	7557	7720	1899	1915	2178	3579	7578
7887	7963	3723	3723	7786	7948	2221	2195	2451	3769	7802
7861	7964	3441	3441	7778	7931	3876	3893	3835	3177	7769
7941	8013	6640	6640	7787	7947	6720	6749	6756	6658	7885
7943	8015	6642	6642	7789	7949	6722	6751	6758	6660	7887
7940	8012	6639	6639	7786	7946	6719	6748	6755	6657	7884
7942	8014	6641	6641	7788	7948	6721	6750	6757	6659	7886
7912	7990	6780	6780	7784	7924	6791	6813	6819	6770	7841
7930	8004	6771	6771	7780	7938	6780	6801	6808	6782	7855
7941	8013	6781	6781	7786	7947	6780	6813	6812	6795	7864
7920	7994	6815	6815	7769	7932	6794	6821	6848	6825	7854
7935	8011	6829	6829	7786	7949	6808	6835	6862	6839	7869
7924	7996	6793	6793	7771	7934	6767	6800	6838	6826	7858
7931	8009	6871	6871	7788	7949	6834	6861	6876	6879	7871
7913	7987	6795	6795	7762	7923	6786	6813	6834	6805	7835
7942	8015	6825	6825	7791	7952	6816	6843	6864	6834	7864
7906	7980	6784	6784	7753	7916	6787	6814	6815	6792	7826
7913	7989	6799	6799	7764	7925	6784	6811	6830	6811	7833
7917	7993	6801	6801	7768	7929	6792	6819	6840	6811	7839
8037	8100	6972	6972	7883	8050	6986	6989	6966	6977	7998
8032	8095	6967	6967	7878	8045	6981	6984	6961	6972	7993
8021	8085	6937	6937	7870	8029	6924	6943	6932	6932	7954
7959	8025	6797	6797	7795	7961	6823	6852	6838	6816	7862
8044	8114	6917	6917	7893	8052	6929	6955	6951	6916	7893
7926	7998	6784	6784	7778	7937	6798	6833	6837	6800	7871
7920	7992	6778	6778	7774	7931	6792	6827	6831	6794	7865
7921	7993	6781	6781	7775	7932	6791	6826	6828	6797	7864
7890	7966	6757	6757	7744	7899	6760	6795	6804	6793	7835
7799	7869	6801	6801	7669	7808	6817	6845	6850	6802	7744
7870	7938	4186	4186	7825	7930	3666	3652	3807	4269	7776
7859	7927	4175	4175	7812	7919	3655	3641	3796	4258	7765
263	2067	7543	7543	3503	2147	7683	7720	7576	7483	815
263	2067	7543	7543	3503	2147	7683	7720	7576	7483	815
262	2066	7542	7542	3502	2146	7682	7719	7575	7482	814
262	2066	7541	7541	3502	2146	7681	7718	7574	7481	814
262	2066	7543	7543	3502	2147	7683	7720	7576	7483	814

262	2066	7541	7541	3502	2147	7681	7718	7574	7481	814
262	2066	7543	7543	3502	2147	7683	7720	7576	7483	814
260	2064	7541	7541	3500	2145	7681	7718	7574	7481	812
263	2067	7544	7544	3503	2148	7684	7721	7577	7484	815
260	2064	7541	7541	3500	2145	7681	7718	7574	7481	812
259	2063	7540	7540	3499	2144	7680	7717	7573	7480	811
260	2064	7541	7541	3500	2145	7681	7718	7574	7481	812
261	2065	7542	7542	3501	2146	7682	7719	7575	7482	813
261	2065	7542	7542	3501	2146	7682	7719	7575	7482	813
81	1928	7625	7625	3368	2026	7771	7806	7713	7575	783
85	1932	7629	7629	3372	2030	7775	7810	7717	7579	787
85	1932	7629	7629	3372	2030	7775	7810	7717	7579	787
88	1935	7632	7632	3375	2033	7778	7813	7720	7582	790
182	2013	7566	7566	3430	2108	7744	7767	7674	7526	874
82	1929	7626	7626	3369	2027	7772	7807	7714	7576	784
	1905	7617	7617	3379	2015	7761	7796	7707	7569	775
8	1903	7615	7615	3377	2013	7759	7794	7705	7567	773
20	1907	7620	7620	3381	2017	7764	7799	7710	7572	777
17	1902	7615	7615	3376	2012	7759	7794	7705	7567	772
21	1904	7616	7616	3378	2014	7761	7796	7707	7568	774
29	1914	7622	7622	3378	2018	7766	7801	7711	7574	784
239	2066	7521	7521	3463	2128	7678	7642	7564	7490	720
775	2192	7529	7529	3594	2245	7690	7722	7622	7463	
846	2249	7529	7529	3650	2296	7690	7722	7626	7461	98
1882	217	7708	7708	3379	1917	7834	7874	7757	7653	2113
1882	217	7708	7708	3379	1917	7834	7874	7757	7653	2113
1889	224	7715	7715	3387	1924	7839	7879	7762	7660	2122
1905		7759	7759	3405	1924	7875	7909	7792	7706	2192
2015	1924	7706	7706	3359		7837	7883	7780	7655	2245
3278	3440	7575	7575	2644	3388	7661	7703	7620	7538	3451
3281	3443	7578	7578	2647	3391	7664	7706	7623	7541	3454
3285	3447	7585	7585	2651	3393	7669	7711	7632	7548	3458
3308	3478	7576	7576	2657	3419	7676	7716	7627	7531	3507
3233	3370	7562	7562	2557	3326	7642	7684	7604	7511	3408
3241	3378	7570	7570	2565	3334	7650	7692	7612	7519	3416
3232	3369	7561	7561	2556	3325	7641	7683	7603	7510	3407
3280	3399	7630	7630	2581	3308	7715	7757	7680	7568	3474
3282	3401	7632	7632	2583	3310	7717	7759	7682	7570	3476
3287	3406	7636	7636	2588	3315	7721	7763	7686	7573	3481
3299	3417	7649	7649	2600	3327	7734	7776	7699	7587	3493
3302	3473	7581	7581	2626	3443	7638	7675	7632	7537	3442
3303	3474	7582	7582	2627	3444	7639	7676	7633	7536	3443
3303	3474	7582	7582	2627	3444	7639	7676	7633	7538	3443
3304	3475	7583	7583	2628	3445	7640	7677	7634	7539	3444
3302	3473	7581	7581	2626	3443	7638	7675	7632	7537	3442
3302	3473	7583	7583	2626	3441	7640	7677	7634	7539	3442
3378	3404	7579	7579	13	3358	7672	7712	7655	7548	3593
3377	3403	7578	7578	12	3357	7671	7711	7654	7547	3592
3376	3402	7577	7577	11	3356	7670	7710	7653	7546	3591
3375	3401	7576	7576	10	3355	7669	7709	7652	7545	3590
3377	3403	7578	7578	12	3357	7671	7711	7654	7547	3592
3376	3402	7577	7577	11	3356	7670	7710	7653	7546	3591
3408	3434	7560	7560	44	3388	7647	7687	7630	7527	3623
3379	3405	7580	7580		3359	7673	7713	7656	7549	3594
3386	3495	7638	7638	2662	3489	7756	7795	7724	7582	3620

3290	3319	7702	7702	2600	3337	7727	7772	7696	7650	3480
3293	3322	7705	7705	2603	3340	7730	7775	7699	7653	3483
3291	3320	7703	7703	2601	3338	7728	7773	7697	7651	3481
3288	3320	7700	7700	2601	3335	7725	7770	7694	7648	3478
25535	25610	24938	24938	25525	25585	25019	25040	24971	24932	25510
25534	25609	24937	24937	25524	25584	25018	25039	24970	24931	25509
31164	31290	30908	30908	31233	31215	30996	31009	30945	30933	31149

KMi011	Kmi017	KMi024	KFu021	Kmi017	KMi019	KTa003	JSWP001	JSWP014	JSWP021
6787	6800	1209	3857	6829	3738	7507	6778	7531	3765
6739	6759	722	3823	6788	3700	7458	6745	7468	3683
6743	6763	724	3823	6792	3698	7458	6749	7468	3685
6755	6769	702	3841	6798	3743	7500	6753	7515	3711
6748	6768	739	3849	6797	3708	7509	6748	7521	3712
6751	6770	741	3885	6799	3722	7472	6750	7488	3724
6751	6770	741	3885	6799	3722	7472	6750	7488	3724
6776	6796	734	3878	6825	3737	7484	6776	7496	3740
6778	6798	736	3880	6827	3740	7488	6778	7499	3743
6789	6778	758	3797	6807	3764	7489	6764	7448	3757
6795	6782	681	3792	6811	3815	7484	6762	7441	3761
6799	6807	745	3884	6836	3752	7497	6793	7480	3741
6830	6814	838	3929	6843	3874	7505	6796	7580	3845
6830	6814	838	3929	6843	3874	7505	6796	7580	3845
6804	6790	793	3859	6819	3789	7453	6772	7532	3783
6816	6802	783	3856	6831	3789	7459	6784	7536	3780
6740	6753	711	3780	6782	3680	7464	6733	7479	3675
6736	6742	779	3830	6771	3731	7515	6728	7512	3710
6788	6784	780	3833	6813	3774	7497	6770	7491	3745
6800	6805	977	3870	6834	3769	7461	6795	7481	3788
6818	6818	859	3874	6847	3819	7518	6790	7512	3809
6749	6749		3732	6779	3706	7478	6733	7501	3689
6752	6753	564	3809	6782	3788	7527	6735	7498	3740
6813	6811	625	3848	6840	3827	7444	6795	7521	3755
6736	6752	628	3788	6781	3721	7472	6734	7488	3663
6788	6784	694	3816	6813	3784	7532	6764	7533	3708
6790	6801	1179	3764	6831	3723	7536	6783	7550	3690
6790	6786	1209	3864	6816	3843	7548	6780	7536	3788
6783	6779	1007	3832	6808	3832	7446	6763	7478	3777
6749	6754	942	3814	6783	3766	7470	6736	7496	3695
6749	6754	942	3814	6783	3766	7470	6736	7496	3695
6750	6755	944	3816	6784	3768	7473	6737	7499	3698
6781	6795	957	3792	6825	3770	7511	6783	7496	3713
6784	6795	1167	3756	6825	3723	7529	6781	7541	3685
6784	6795	1167	3756	6825	3723	7529	6781	7541	3685
6686	6684	3493	2027	6713	2147	7624	6670	7615	2221
6676	6674	3497	2034	6703	2147	7621	6656	7613	2190
6681	6679	3503	2026	6709	2137	7621	6659	7614	2196
6664	6662	3466	2029	6692	2174	7623	6644	7612	2187
6672	6670	3480	2004	6700	2131	7608	6652	7598	2190
6668	6666	3491	2022	6696	2153	7624	6648	7605	2202
6667	6665	3490	2021	6695	2152	7625	6647	7606	2201
6680	6687	3487	2029	6717	2115	7622	6669	7601	2200
6698	6700	3488	2018	6730	2136	7611	6680	7601	2217
6666	6653	3469	1977	6683	2163	7597	6635	7573	2250
6684	6675	3434	2073	6705	2222	7575	6659	7532	2303
6798	6794	3546	2101	6824	1935	7620	6772	7580	2250
6693	6681	3431	2036	6711	2239	7545	6661	7568	2131
6688	6676	3426	2031	6706	2234	7544	6656	7567	2126
6694	6682	3432	2037	6712	2240	7550	6662	7573	2132
6688	6676	3426	2031	6706	2234	7544	6656	7567	2126
6693	6681	3431	2036	6711	2239	7549	6661	7572	2131
6725	6722	3558	2017	6752	2179	7609	6698	7603	2079

6831	6836	3644	2067	6866	2391	7651	6816	7617	2266
6752	6738	3534	1953	6768	2209	7596	6716	7558	1935
6751	6737	3533	1952	6767	2208	7596	6715	7558	1934
6743	6729	3528	1945	6759	2201	7591	6707	7553	1927
6746	6732	3530	1947	6762	2203	7593	6710	7555	1929
6758	6759	3591	1919	6789	2167	7583	6737	7562	1920
6767	6768	3600	1928	6798	2176	7592	6746	7571	1929
6767	6768	3600	1928	6798	2176	7592	6746	7571	1929
6748	6734	3563	1892	6764	2148	7566	6712	7527	1873
6763	6749	3585	1915	6779	2183	7578	6727	7539	1890
6742	6724	3558	1883	6754	2185	7549	6702	7514	1903
6740	6726	3555	1884	6756	2140	7558	6704	7519	1865
6748	6736	3564	1885	6766	2214	7594	6714	7558	1915
6748	6736	3564	1885	6766	2214	7594	6714	7558	1915
6737	6725	3553	1874	6755	2203	7584	6703	7548	1904
6733	6722	3562	1852	6752	2196	7582	6700	7567	1930
6809	6800	3533	1957	6830	2086	7600	6778	7540	2114
6837	6828	3564	1988	6858	2117	7629	6806	7569	2145
6811	6802	3535	1959	6832	2088	7602	6780	7542	2116
6809	6800	3533	1957	6830	2086	7600	6778	7540	2114
6842	6839	3689	2404	6869	2456	7631	6817	7579	
6844	6841	3692	2406	6871	2458	7633	6819	7581	18
6838	6835	3686	2400	6865	2452	7627	6813	7575	12
6835	6832	3683	2397	6862	2449	7624	6810	7572	9
6846	6843	3695	2379	6873	2446	7633	6821	7583	53
6839	6836	3681	2395	6866	2447	7624	6814	7574	17
6837	6834	3685	2399	6864	2451	7626	6812	7574	11
6848	6845	3692	2392	6875	2458	7631	6823	7579	26
6837	6834	3685	2399	6864	2451	7626	6812	7574	11
6836	6833	3684	2398	6863	2450	7625	6811	7573	10
6837	6832	3681	2383	6862	2399	7616	6810	7570	83
6822	6819	3710	2374	6849	2492	7637	6787	7583	98
6841	6838	3661	2405	6868	2473	7618	6816	7566	127
6841	6838	3661	2405	6868	2473	7618	6816	7566	127
6841	6838	3661	2405	6868	2473	7618	6816	7566	127
6814	6808	3732	2300	6838	2355	7674	6788	7655	1683
6814	6808	3722	2233	6838	2233	7667	6786	7646	1597
6708	6678	3549	2009	6708	2194	7601	6654	7577	1912
6712	6682	3541	2011	6712	2186	7598	6656	7578	1892
6712	6682	3541	2011	6712	2186	7598	6656	7578	1892
6708	6678	3550	2001	6708	2170	7596	6652	7576	1892
6794	6799	3525	2085	6829	2287	7593	6779	7549	2031
6821	6807	3610	2323	6837	2397	7658	6783	7657	1883
6806	6792	3660	2451	6822	2494	7696	6768	7694	2004
6812	6798	3657	2526	6828	2537	7707	6774	7701	1981
6812	6798	3657	2526	6828	2537	7707	6774	7701	1981
6827	6809	3656	2507	6839	2595	7709	6787	7703	2062
6820	6806	3638	2475	6836	2538	7713	6786	7707	2016
6811	6797	3678	2465	6827	2428	7679	6773	7673	1914
6786	6772	3750	2466	6802	2587	7686	6746	7670	1925
6881	6869	3677	2469	6899	2566	7758	6845	7698	1869
6892	6880	3673	2462	6910	2595	7761	6856	7697	1922
6753	6744	3494	1959	6774	2076	7595	6726	7529	2105
6773	6764	3506	1984	6794	2086	7611	6748	7550	2125
6768	6756	3511	1962	6786	2002	7602	6740	7555	2051

6771	6759	3511	1964	6789	2006	7604	6743	7557	2053
6769	6757	3511	1986	6787	2015	7607	6741	7560	2085
6789	6779	3489	2017	6809	2086	7581	6761	7529	2097
6794	6782	3517	2075	6812	2041	7606	6766	7557	2142
6796	6784	3517	2075	6814	2041	7608	6768	7559	2142
6810	6806	3529	2061	6836	2027	7610	6788	7553	2087
6812	6808	3531	2063	6838	2029	7612	6790	7555	2089
6812	6808	3531	2063	6838	2029	7610	6790	7553	2089
6817	6813	3543	2070	6843	2047	7598	6795	7539	2110
6805	6801	3526	2060	6831	2032	7612	6785	7551	2084
6819	6817	3508	2090	6847	2048	7594	6801	7539	2078
6820	6818	3509	2091	6848	2049	7595	6802	7540	2079
6790	6773	3558	1929	6803	2002	7636	6753	7566	2088
6788	6771	3564	1925	6801	2010	7637	6751	7567	2078
6755	6736	3522	2060	6766	2103	7613	6716	7611	2122
6755	6736	3522	2062	6766	2105	7615	6716	7613	2122
6747	6728	3514	2052	6758	2095	7605	6708	7603	2113
6746	6727	3513	2051	6757	2094	7604	6707	7602	2113
6737	6712	3527	2061	6742	2108	7615	6692	7611	2141
6746	6727	3515	2051	6757	2094	7604	6707	7602	2113
6747	6728	3514	2052	6758	2095	7605	6708	7603	2114
6761	6742	3528	2066	6772	2109	7617	6722	7615	2128
6760	6741	3507	2058	6771	2132	7598	6723	7592	2160
6775	6777	3544	2055	6807	2138	7620	6757	7650	2161
6775	6753	3728	778	6783	2341	7738	6751	7738	2370
6779	6760	3727	794	6790	2356	7735	6758	7741	2384
6792	6770	3740	796	6800	2359	7753	6768	7753	2388
6776	6754	3749	777	6784	2336	7727	6752	7727	2352
6833	6811	3649	538	6841	2216	7728	6813	7724	2227
6833	6813	3640	507	6843	2195	7722	6813	7718	2234
6833	6813	3640	507	6843	2195	7722	6813	7718	2234
6830	6810	3638	505	6840	2193	7719	6810	7715	2232
6841	6819	3650	519	6849	2199	7724	6821	7718	2242
6839	6817	3683	523	6847	2190	7696	6819	7692	2219
6839	6817	3678	529	6847	2173	7727	6819	7723	2209
6830	6807	3736	668	6837	2271	7690	6834	7690	2289
6825	6808	3650	453	6838	2230	7687	6806	7679	2258
6826	6804	3686	459	6834	2193	7721	6802	7718	2205
6848	6839	3698	535	6869	2222	7722	6841	7704	2228
6853	6831	3702	494	6861	2212	7722	6833	7723	2230
6859	6837	3707	499	6867	2217	7726	6839	7727	2235
6834	6815	3682	498	6845	2226	7681	6817	7692	2222
6840	6818	3657	462	6848	2228	7723	6816	7722	2231
6831	6809	3646	451	6839	2221	7714	6807	7711	2228
6814	6792	3671	308	6822	2103	7678	6786	7678	2186
6819	6797	3675	317	6827	2103	7681	6793	7681	2197
6830	6730	3723	19	6760	2278	7702	6718	7682	2395
6839	6739	3732		6769	2287	7711	6727	7691	2404
6823	6723	3716	22	6753	2271	7695	6711	7675	2388
6808	6782	3698	364	6812	2174	7684	6757	7685	2257
6810	6788	3671	323	6818	2109	7678	6788	7674	2207
6811	6789	3676	323	6819	2110	7681	6789	7677	2207
6753	6754	3715	633	6784	2358	7581	6791	7562	2463
6798	6786	3677	395	6816	2221	7690	6780	7681	2318
6791	6779	3668	398	6809	2214	7682	6781	7671	2323

6796	6784	3687	416	6814	2226	7691	6786	7680	2334
6807	6795	3682	411	6825	2229	7698	6797	7687	2337
6825	6728	3716	378	6758	2265	7696	6728	7677	2307
6837	6815	3698	407	6845	2176	7704	6817	7702	2276
6830	6808	3688	400	6838	2169	7694	6810	7692	2269
6828	6806	3686	398	6836	2167	7692	6808	7690	2267
6814	6792	3651	328	6822	2108	7673	6790	7673	2190
6813	6791	3676	314	6821	2106	7653	6789	7653	2201
6834	6812	3693	350	6841	2134	7695	6810	7698	2203
6827	6805	3686	341	6834	2127	7688	6803	7691	2196
6794	6772	3659	540	6802	2035	7662	6760	7661	2108
6794	6772	3659	540	6802	2035	7662	6760	7661	2108
6817	6795	3662	332	6825	2149	7680	6795	7676	2248
6812	6790	3657	327	6820	2144	7675	6790	7671	2243
6815	6793	3660	330	6823	2147	7678	6793	7674	2246
6697	6687	3443	1987	6717	823	7558	6671	7516	2198
6696	6686	3446	1988	6716	824	7555	6670	7513	2199
6685	6675	3433	1975	6705	811	7545	6659	7503	2186
6798	6788	3628	2126	6818	777	7647	6770	7599	2359
6721	6711	3487	1969	6741	596	7574	6693	7528	2183
6959	6949	3706	2287	6979		7798	6931	7752	2456
6993	7022	3448	3974	7051	3946	7769	7006	7767	3840
390	219	6594	6673	249	6877	7887	215	7904	6761
392	221	6596	6675	251	6879	7889	217	7906	6763
389	218	6593	6672	248	6876	7886	214	7903	6760
391	220	6595	6674	250	6878	7888	216	7905	6762
276	105	6731	6744	135	6939	7827	37	7873	6824
265	94	6719	6733	124	6923	7841	22	7891	6813
269	98	6733	6727	128	6931	7850		7902	6817
201	30	6769	6747	60	6957	7846	112	7881	6853
217	46	6783	6761	76	6971	7861	128	7896	6867
256	86	6745	6722	116	6954	7850	162	7885	6843
257	86	6825	6787	116	6999	7871	154	7898	6881
179		6749	6739	30	6949	7825	98	7874	6839
209	30	6779	6769		6979	7854	128	7903	6869
200	29	6740	6740	59	6932	7820	91	7867	6820
187	16	6755	6737	46	6953	7829	98	7874	6835
185	14	6755	6745	44	6955	7829	104	7878	6845
434	391	6941	6898	421	7092	7992	435	8009	6971
429	386	6936	6893	416	7087	7987	430	8004	6966
425	377	6892	6857	407	7059	7947	435	7967	6937
201	225	6771	6846	255	6954	7854	283	7903	6843
347	363	6861	6951	393	7084	7883	453	8006	6956
	179	6749	6839	209	6959	7861	269	7892	6842
10	173	6743	6833	203	6953	7855	263	7886	6836
21	184	6748	6832	214	6952	7860	266	7887	6833
125	286	6729	6801	316	6926	7829	372	7858	6809
292	455	6774	6858	485	6989	7734	537	7765	6855
6914	6920	4165	3756	6950	3755	7784	6958	7790	3812
6904	6910	4154	3745	6940	3744	7773	6948	7779	3801
7894	7876	7503	7693	7905	7754	880	7904	3	7581
7894	7876	7503	7693	7905	7754	880	7904	3	7581
7893	7875	7502	7692	7904	7753	879	7903	2	7580
7892	7874	7501	7691	7903	7752	879	7902		7579
7894	7876	7503	7693	7905	7754	879	7904	4	7581

7894	7876	7501	7691	7905	7752	879	7904	6	7579
7894	7876	7503	7693	7905	7754	879	7904	6	7581
7892	7874	7501	7691	7903	7752	877	7902	4	7579
7895	7877	7504	7694	7906	7755	880	7905	7	7582
7892	7874	7501	7691	7903	7752	877	7902	4	7579
7891	7873	7500	7690	7902	7751	876	7901	3	7578
7892	7874	7501	7691	7903	7752	877	7902	4	7579
7893	7875	7502	7692	7904	7753	878	7903	5	7580
7893	7875	7502	7692	7904	7753	878	7903	5	7580
7931	7918	7593	7759	7947	7895	850	7947	285	7718
7935	7922	7597	7763	7951	7897	854	7951	289	7722
7935	7922	7597	7763	7951	7899	854	7951	289	7722
7938	7925	7600	7766	7954	7902	857	7954	292	7725
7941	7924	7535	7729	7953	7859	941	7949	384	7679
7932	7919	7594	7760	7948	7896	851	7948	286	7719
7926	7913	7587	7749	7942	7887	846	7941	262	7712
7925	7912	7585	7747	7941	7885	844	7940	260	7710
7929	7916	7590	7752	7945	7890	848	7944	264	7715
7924	7911	7585	7747	7940	7885	843	7939	259	7710
7926	7913	7586	7749	7942	7887	845	7941	259	7712
7929	7916	7592	7754	7945	7891	855	7944	269	7716
7871	7866	7505	7705	7895	7789	785	7894	323	7569
7871	7835	7480	7711	7864	7802	98	7864	814	7627
7861	7825	7478	7711	7854	7798		7850	879	7631
7953	7940	7661	7826	7968	7927	2173	7967	2045	7762
7953	7940	7661	7826	7968	7927	2173	7967	2045	7762
7960	7947	7668	7831	7975	7932	2182	7974	2052	7767
7998	7987	7719	7873	8015	7963	2249	8013	2066	7797
7937	7923	7661	7835	7952	7948	2296	7947	2146	7785
7693	7688	7537	7641	7717	7778	3492	7718	3409	7625
7696	7691	7540	7644	7720	7781	3495	7721	3412	7628
7704	7699	7547	7649	7728	7788	3499	7726	3416	7637
7681	7676	7522	7652	7705	7793	3536	7703	3433	7632
7724	7719	7520	7620	7748	7755	3449	7746	3364	7609
7732	7727	7528	7628	7756	7763	3457	7754	3372	7617
7723	7718	7519	7619	7747	7754	3448	7745	3363	7608
7730	7725	7583	7693	7754	7838	3498	7749	3415	7685
7732	7727	7585	7695	7756	7840	3500	7751	3417	7687
7737	7732	7589	7699	7761	7844	3505	7756	3422	7691
7749	7744	7602	7712	7773	7857	3517	7768	3434	7704
7749	7724	7544	7616	7753	7768	3502	7754	3425	7635
7750	7725	7543	7617	7754	7769	3503	7755	3426	7636
7750	7725	7545	7617	7754	7769	3503	7755	3426	7636
7751	7726	7546	7618	7755	7770	3504	7756	3427	7637
7749	7724	7544	7616	7753	7768	3502	7754	3425	7635
7751	7726	7546	7618	7755	7768	3502	7756	3425	7637
7777	7761	7546	7677	7790	7785	3649	7785	3501	7660
7776	7760	7545	7676	7789	7784	3648	7784	3500	7659
7775	7759	7544	7675	7788	7783	3647	7783	3499	7658
7774	7758	7543	7674	7787	7782	3646	7782	3498	7657
7776	7760	7545	7676	7789	7784	3648	7784	3500	7659
7775	7759	7544	7675	7788	7783	3647	7783	3499	7658
7802	7786	7527	7652	7815	7760	3679	7766	3531	7635
7778	7762	7547	7678	7791	7786	3650	7786	3502	7661
7799	7788	7611	7778	7817	7856	3672	7815	3524	7729

7961	7944	7647	7753	7973	7847	3523	7971	3412	7701
7966	7949	7650	7756	7978	7850	3526	7976	3415	7704
7964	7947	7648	7754	7976	7848	3524	7974	3413	7702
7959	7942	7645	7751	7971	7845	3521	7969	3410	7699
25023	25015	24914	25044	25045	25174	25514	25040	25532	24974
25022	25014	24913	25043	25044	25173	25513	25039	25531	24973
30792	30795	30914	31016	30825	31149	31159	30809	31136	30948

1657	5051	VREC0418	VREC0426	VREC0506	VREC0559	C-0863N0015	AMA940	703
3684	3499	3544	3658	3523	7701	7557	3563	3556
3628	3485	3487	3563	3504	7631	7489	3525	3471
3626	3485	3485	3563	3502	7633	7491	3525	3471
3670	3531	3532	3600	3540	7674	7514	3565	3530
3632	3502	3495	3575	3520	7682	7536	3581	3497
3654	3538	3507	3599	3551	7647	7495	3586	3525
3654	3538	3507	3599	3551	7647	7495	3586	3525
3667	3540	3526	3616	3557	7659	7530	3583	3528
3670	3542	3529	3620	3559	7665	7536	3586	3530
3696	3575	3560	3654	3585	7641	7482	3606	3586
3747	3603	3599	3669	3610	7634	7492	3630	3611
3682	3537	3541	3635	3577	7671	7530	3601	3571
3803	3646	3677	3757	3663	7736	7591	3710	3641
3803	3646	3677	3757	3663	7736	7591	3710	3641
3717	3581	3591	3667	3598	7686	7541	3637	3576
3718	3571	3596	3664	3595	7690	7547	3637	3565
3615	3467	3487	3542	3486	7648	7508	3513	3461
3668	3526	3538	3593	3527	7674	7538	3560	3485
3710	3572	3575	3635	3592	7664	7543	3614	3574
3698	3586	3579	3652	3637	7653	7527	3635	3620
3752	3604	3621	3676	3645	7685	7552	3667	3616
3628	3488	3487	3600	3515	7661	7527	3517	3491
3705	3584	3580	3664	3590	7685	7508	3621	3605
3749	3595	3606	3676	3617	7694	7534	3641	3618
3643	3478	3515	3553	3506	7641	7496	3538	3519
3712	3540	3561	3624	3555	7693	7557	3576	3545
3648	3411	3514	3551	3504	7717	7572	3542	3478
3758	3613	3640	3684	3642	7730	7572	3657	3588
3763	3561	3621	3658	3614	7647	7528	3645	3581
3691	3500	3534	3609	3539	7669	7523	3549	3479
3691	3500	3534	3609	3539	7669	7523	3549	3479
3693	3502	3536	3612	3541	7672	7526	3552	3481
3690	3455	3545	3611	3563	7690	7549	3524	3498
3644	3413	3510	3557	3506	7708	7560	3536	3466
3644	3413	3510	3557	3506	7708	7560	3536	3466
2050	195	1875	1820	1945	7757	7603	1911	149
2053	180	1859	1811	1936	7752	7598	1889	130
2043	162	1854	1793	1916	7756	7600	1884	118
2080	241	1901	1790	1955	7758	7605	1907	165
2039	164	1846	1795	1918	7742	7583	1865	104
2059	178	1868	1809	1930	7751	7597	1892	
2058	177	1867	1808	1929	7752	7596	1891	5
2021	178	1830	1795	1927	7755	7595	1885	140
2041		1858	1821	1946	7754	7592	1912	178
2069	198	1878	1863	1954	7730	7572	1928	206
2121	437	1915	1923	2028	7708	7543	1992	423
1998	1816	1778	1919	1964	7755	7580	1947	1781
2149	1793	2008	1705	1741	7703	7563	1818	1779
2144	1788	2003	1700	1736	7700	7560	1813	1774
2150	1794	2009	1706	1742	7706	7566	1819	1780
2144	1788	2003	1700	1736	7700	7560	1813	1774
2149	1793	2008	1705	1741	7705	7565	1818	1779
2147	1791	1988	1533	1922	7770	7600	1776	1769

2272	1988	2103	1747	1955	7815	7651	1856	1976
2066	1858	1938	374	1886	7722	7603	1848	1846
2065	1857	1937	373	1885	7720	7602	1847	1845
2058	1850	1930	366	1878	7715	7597	1842	1838
2060	1852	1932	368	1880	7717	7599	1844	1840
2071	1812	1922	21	1878	7707	7583	1788	1800
2080	1821	1931		1887	7716	7592	1797	1809
2080	1821	1931	34	1887	7716	7592	1797	1809
2030	1813	1885	188	1849	7693	7558	1787	1801
2065	1844	1920	225	1872	7706	7565	1808	1832
2067	1780	1922	239	1856	7680	7540	1823	1792
2022	1805	1877	180	1841	7685	7550	1779	1793
2079	1881	1934	482	1869	7722	7581	1849	1867
2079	1881	1934	482	1869	7722	7581	1849	1867
2068	1870	1923	471	1858	7712	7570	1838	1856
2078	1835	1933	337	1862	7706	7568	1843	1815
1964	1867	1807	1430	1857	7733	7563	1827	1853
1995	1898	1838	1461	1888	7760	7592	1858	1884
1966	1869	1809	1432	1859	7735	7565	1829	1855
1964	1867	1807	1430	1857	7733	7563	1827	1853
2359	2217	2183	1929	2113	7762	7635	2142	2202
2361	2219	2185	1931	2115	7764	7637	2144	2204
2355	2213	2179	1925	2109	7758	7631	2138	2198
2352	2210	2176	1922	2106	7755	7628	2135	2195
2358	2214	2187	1926	2100	7766	7637	2162	2199
2350	2208	2176	1920	2104	7755	7630	2135	2193
2354	2212	2178	1924	2108	7757	7630	2137	2197
2349	2221	2185	1913	2121	7762	7631	2152	2206
2354	2212	2178	1924	2108	7757	7630	2137	2197
2353	2211	2177	1923	2107	7756	7629	2136	2196
2302	2214	2126	1938	2080	7755	7630	2147	2209
2401	2233	2229	1989	2145	7773	7643	2204	2218
2374	2232	2214	1932	2055	7748	7612	2137	2217
2374	2232	2214	1932	2055	7748	7612	2137	2217
2374	2232	2214	1932	2055	7748	7612	2137	2217
2330	2280	2145	2055	2065	7804	7690	2128	2271
2208	2236	2023	2012	2028	7804	7691	2028	2233
2094	1981	1937	1799	1969	7748	7602	1967	1951
2078	1965	1919	1783	1949	7745	7600	1957	1943
2078	1965	1919	1783	1949	7745	7600	1957	1943
2065	1966	1902	1767	1955	7745	7597	1973	1944
2118	2049	1981	1861	1859	7744	7594	1907	2037
2271	2242	2131	1990	2190	7796	7665	1933	2243
2394	2323	2254	2120	2202	7828	7689	2053	2324
2445	2382	2299	2175	2227	7835	7707	2076	2375
2445	2382	2299	2175	2227	7835	7707	2076	2375
2475	2383	2333	2140	2292	7847	7708	2124	2366
2427	2338	2282	2142	2261	7847	7707	2061	2321
2397	2371	2254	2112	2155	7793	7675	2094	2364
2435	2370	2296	2058	2195	7812	7683	2175	2363
2489	2425	2397	2173	2235	7891	7745	2216	2419
2491	2447	2399	2194	2244	7893	7737	2251	2441
2072	1871	1876	1791	1854	7733	7553	515	1835
2088	1886	1886	1802	1865	7752	7574	511	1848
2006	1853	1811	1707	1801	7739	7564	423	1841

2010	1857	1815	1709	1803	7741	7566	425	1845
2015	1888	1824	1725	1811	7744	7570	387	1876
2084	1971	1913	1743	1867	7720	7544	465	1949
2037	1912	1865	1797	1878	7748	7573		1892
2037	1912	1865	1797	1878	7750	7575	20	1892
2041	1943	1876	1747	1911	7744	7588	332	1923
2043	1945	1878	1749	1913	7746	7590	334	1925
2043	1945	1878	1749	1913	7744	7590	334	1925
2065	1967	1903	1772	1939	7732	7594	353	1940
2046	1948	1877	1754	1916	7746	7587	327	1912
2074	1970	1905	1766	1924	7730	7571	397	1944
2075	1971	1906	1767	1925	7729	7572	398	1945
1980	1866	1829	1685	1876	7777	7584	607	1854
1992	1866	1823	1679	1870	7778	7582	603	1844
2125	1955	1930	1896	33	7746	7631	1885	1939
2127	1957	1932	1896	35	7748	7633	1887	1941
2117	1947	1922	1885	27	7738	7623	1877	1931
2116	1946	1921	1887	26	7737	7622	1876	1930
2130	1960	1935	1907	48	7746	7633	1904	1928
2116	1946	1921	1887		7737	7622	1878	1930
2117	1947	1922	1888	25	7738	7623	1877	1931
2131	1961	1936	1902	41	7750	7635	1891	1945
2150	1990	1964	1928	76	7731	7627	1913	1961
2158	2051	1963	1953	174	7727	7609	1926	2034
2142	2142	2057	1956	2082	7900	7691	2127	2138
2157	2156	2072	1972	2091	7907	7698	2147	2142
2158	2160	2073	1972	2098	7915	7708	2143	2156
2117	2135	2044	1947	2067	7889	7693	2088	2131
2075	1964	1924	1778	1942	7880	7690	1975	1948
2054	1949	1915	1769	1935	7874	7687	1970	1941
2054	1949	1915	1769	1935	7874	7687	1970	1941
2052	1947	1913	1767	1933	7871	7684	1968	1939
2064	1963	1929	1781	1933	7876	7691	1976	1953
2049	1946	1907	1765	1918	7848	7689	1936	1935
2058	1935	1893	1753	1907	7879	7701	1946	1917
2156	2016	1995	1815	1978	7847	7714	2037	2016
2123	1925	1960	1843	1935	7851	7678	2003	1955
2086	1921	1923	1739	1905	7871	7700	1948	1909
2113	1936	1956	1756	1914	7872	7718	1977	1914
2103	1946	1946	1762	1928	7871	7718	1969	1938
2108	1951	1951	1767	1933	7876	7720	1974	1943
2113	1934	1954	1778	1923	7853	7701	1967	1920
2087	1950	1950	1760	1932	7873	7694	1973	1950
2082	1945	1945	1755	1925	7864	7685	1964	1935
1974	1853	1787	1708	1871	7829	7663	1889	1841
1976	1855	1793	1709	1874	7832	7669	1895	1853
2117	2009	1960	1919	2042	7817	7643	2066	2013
2126	2018	1969	1928	2051	7826	7652	2075	2022
2110	2002	1953	1912	2035	7810	7636	2059	2006
2013	1962	1856	1765	1950	7832	7651	1916	1940
1986	1861	1803	1716	1882	7829	7668	1897	1849
1987	1862	1804	1717	1882	7832	7669	1898	1850
2241	2055	2061	1992	2105	7746	7654	2133	2078
2081	1966	1899	1861	1973	7834	7647	2018	1934
2082	1967	1902	1860	1972	7826	7638	2013	1933

2095	1975	1918	1870	1982	7835	7649	2029	1941
2098	1980	1921	1873	1985	7842	7654	2032	1946
2130	1978	1941	1858	2008	7822	7652	1998	1933
2044	1926	1864	1785	1952	7855	7670	1968	1920
2037	1919	1857	1778	1945	7845	7658	1958	1913
2035	1917	1855	1776	1943	7843	7656	1956	1911
1970	1853	1802	1726	1891	7822	7661	1888	1853
1977	1856	1797	1712	1878	7804	7658	1899	1857
2005	1881	1809	1744	1907	7849	7680	1898	1871
1998	1872	1802	1735	1900	7842	7673	1891	1862
1970	1809	1747	1654	1795	7811	7648	1812	1799
1970	1809	1747	1654	1795	7811	7648	1812	1799
2008	1897	1835	1757	1921	7831	7659	1932	1883
2003	1892	1830	1753	1916	7826	7654	1927	1878
2006	1895	1833	1756	1919	7829	7657	1930	1881
581	1817	390	1919	1870	7699	7538	1831	1821
582	1818	391	1920	1871	7694	7535	1830	1822
569	1805	378	1907	1858	7686	7525	1819	1809
	2041	330	2080	2116	7771	7591	2037	2059
330	1858		1931	1921	7695	7531	1865	1868
777	2136	596	2176	2094	7927	7760	2041	2153
3808	3809	3714	3791	3788	7925	7756	3761	3836
6716	6624	6639	6692	6657	7966	7767	6702	6590
6718	6626	6641	6694	6659	7968	7769	6704	6592
6715	6623	6638	6691	6656	7965	7766	6701	6589
6717	6625	6640	6693	6658	7967	7768	6703	6591
6774	6688	6698	6753	6713	7944	7764	6769	6653
6758	6674	6687	6744	6699	7958	7760	6758	6640
6770	6680	6693	6746	6707	7967	7766	6766	6648
6796	6708	6719	6784	6743	7949	7793	6790	6674
6810	6722	6733	6798	6757	7966	7810	6804	6688
6788	6696	6714	6779	6730	7951	7795	6787	6664
6838	6742	6761	6816	6781	7966	7812	6816	6708
6788	6700	6711	6768	6727	7940	7786	6782	6666
6818	6730	6741	6798	6757	7968	7815	6812	6696
6771	6683	6694	6751	6710	7933	7777	6769	6649
6792	6704	6715	6770	6729	7942	7788	6786	6670
6794	6706	6717	6774	6733	7946	7792	6788	6672
6931	6851	6854	6901	6905	8062	7907	6919	6850
6926	6846	6849	6896	6900	8057	7902	6914	6845
6900	6805	6823	6866	6863	8043	7894	6886	6798
6795	6701	6718	6777	6753	7978	7819	6807	6686
6923	6813	6846	6885	6875	8072	7917	6909	6820
6798	6698	6721	6767	6746	7953	7802	6794	6668
6792	6692	6714	6761	6740	7947	7798	6788	6662
6791	6691	6714	6760	6739	7948	7799	6787	6661
6767	6668	6690	6730	6716	7921	7768	6759	6638
6829	6717	6752	6778	6772	7824	7693	6801	6684
3689	3503	3529	3543	3548	7894	7853	3353	3489
3678	3492	3518	3532	3537	7883	7840	3342	3478
7601	7603	7530	7573	7604	2046	3532	7559	7607
7601	7603	7530	7573	7604	2046	3532	7559	7607
7600	7602	7529	7572	7603	2045	3531	7558	7606
7599	7601	7528	7571	7602	2045	3531	7557	7605
7601	7603	7530	7573	7604	2045	3531	7559	7607

7599	7601	7528	7571	7602	2045	3531	7557	7605
7601	7603	7530	7573	7604	2045	3531	7559	7607
7599	7601	7528	7571	7602	2043	3529	7557	7605
7602	7604	7531	7574	7605	2046	3532	7560	7608
7599	7601	7528	7571	7602	2043	3529	7557	7605
7598	7600	7527	7570	7601	2042	3528	7556	7604
7599	7601	7528	7571	7602	2043	3529	7557	7605
7600	7602	7529	7572	7603	2044	3530	7558	7606
7600	7602	7529	7572	7603	2044	3530	7558	7606
7742	7721	7671	7695	7702	1910	3396	7696	7718
7744	7725	7673	7699	7706	1914	3400	7700	7722
7746	7725	7675	7699	7706	1914	3400	7700	7722
7749	7728	7678	7702	7709	1917	3403	7703	7725
7705	7682	7634	7654	7667	1995	3458	7651	7679
7743	7722	7672	7696	7703	1911	3397	7697	7719
7734	7714	7663	7687	7694	1882	3408	7688	7711
7732	7712	7661	7685	7692	1880	3406	7686	7709
7737	7717	7666	7690	7697	1884	3410	7691	7714
7732	7712	7661	7685	7692	1879	3405	7686	7709
7734	7714	7663	7687	7694	1881	3407	7688	7711
7739	7718	7668	7691	7700	1891	3407	7693	7715
7636	7588	7565	7541	7593	2043	3492	7571	7575
7651	7611	7578	7594	7608	2113	3623	7596	7624
7647	7611	7574	7592	7604	2173	3679	7606	7624
7771	7754	7695	7716	7737	2	3408	7748	7751
7771	7754	7695	7716	7737		3408	7748	7751
7776	7759	7700	7721	7742	15	3416	7753	7756
7807	7790	7731	7754	7777	217	3434	7787	7793
7753	7753	7720	7731	7767	1917	3388	7762	7754
7617	7620	7540	7619	7638	3414	2672	7609	7628
7620	7623	7543	7622	7641	3417	2675	7612	7631
7627	7628	7550	7627	7646	3421	2680	7619	7636
7620	7627	7551	7621	7643	3454	2686	7611	7637
7590	7599	7513	7598	7612	3344	2586	7583	7607
7598	7607	7521	7606	7620	3352	2594	7591	7615
7589	7598	7512	7597	7611	3343	2585	7582	7606
7676	7669	7599	7658	7686	3383	2610	7659	7677
7678	7671	7601	7660	7688	3385	2612	7661	7679
7682	7675	7605	7664	7692	3390	2617	7665	7683
7695	7688	7618	7677	7705	3401	2629	7678	7696
7573	7609	7527	7613	7634	3464	2652	7582	7614
7574	7610	7528	7614	7635	3465	2653	7583	7615
7574	7610	7528	7614	7635	3465	2653	7583	7615
7575	7611	7529	7615	7636	3466	2654	7584	7616
7573	7609	7527	7613	7634	3464	2652	7582	7614
7575	7611	7529	7615	7636	3464	2652	7584	7616
7616	7613	7556	7615	7647	3378	43	7598	7618
7615	7612	7555	7614	7646	3377	42	7597	7617
7614	7611	7554	7613	7645	3376	41	7596	7616
7613	7610	7553	7612	7644	3375	40	7595	7615
7615	7612	7555	7614	7646	3377	42	7597	7617
7614	7611	7554	7613	7645	3376	41	7596	7616
7591	7592	7531	7592	7622	3408		7573	7597
7617	7614	7557	7616	7648	3379	44	7599	7619
7702	7702	7636	7710	7713	3478	2691	7701	7706

7672	7684	7628	7698	7681	3315	2628	7651	7693
7675	7687	7631	7701	7684	3318	2631	7654	7696
7673	7685	7629	7699	7682	3316	2629	7652	7694
7670	7682	7626	7696	7679	3316	2629	7649	7691
24944	24980	24961	25016	25032	25595	25545	24978	24988
24943	24979	24960	25015	25031	25594	25544	24977	24987
30933	31008	30936	30936	30977	31273	31248	31001	31013

998	1599	VRES0107	EC-F86E-R-141010	412049521	VREC0504	EC362	MS2481
3536	3520	3563	6791	7544	3532	3759	7530
3503	3445	3525	6760	7495	3513	3677	7467
3503	3445	3525	6764	7495	3511	3679	7467
3527	3496	3565	6761	7552	3549	3703	7514
3529	3473	3581	6781	7548	3529	3704	7520
3557	3501	3586	6797	7515	3560	3718	7487
3557	3501	3586	6797	7515	3560	3718	7487
3560	3504	3583	6783	7523	3566	3734	7495
3562	3506	3586	6785	7527	3568	3737	7498
3556	3558	3606	6777	7508	3594	3751	7447
3581	3540	3630	6769	7501	3619	3755	7440
3545	3541	3601	6832	7527	3586	3735	7479
3651	3640	3710	6810	7600	3672	3837	7579
3651	3640	3710	6810	7600	3672	3837	7579
3586	3566	3637	6791	7552	3607	3781	7531
3576	3555	3637	6798	7556	3604	3772	7535
3474	3451	3513	6737	7512	3495	3667	7478
3536	3479	3560	6740	7528	3536	3702	7511
3558	3553	3614	6807	7527	3601	3743	7490
3590	3598	3635	6826	7526	3646	3780	7480
3607	3602	3667	6800	7548	3654	3801	7511
3469	3466	3517	6745	7535	3522	3681	7500
3558	3546	3621	6741	7548	3599	3736	7497
3585	3561	3641	6815	7559	3626	3749	7520
3509	3446	3538	6763	7498	3515	3655	7487
3556	3490	3576	6781	7559	3564	3702	7532
3450	3415	3542	6793	7575	3513	3682	7549
3565	3543	3657	6780	7577	3651	3780	7535
3562	3518	3645	6775	7511	3623	3769	7477
3489	3418	3549	6753	7514	3548	3689	7495
3489	3418	3549	6753	7514	3548	3689	7495
3491	3420	3552	6754	7517	3550	3692	7498
3464	3441	3524	6794	7538	3572	3707	7495
3438	3403	3536	6793	7566	3515	3677	7540
3438	3403	3536	6793	7566	3515	3677	7540
253	196	1911	6671	7685	1956	2212	7614
234	177	1889	6663	7683	1947	2181	7612
224	167	1884	6675	7684	1927	2187	7613
283		1907	6660	7682	1966	2178	7611
208	151	1865	6657	7668	1929	2181	7597
206	165	1892	6664	7679	1941	2193	7604
205	164	1891	6663	7680	1940	2192	7605
206	215	1885	6685	7679	1938	2191	7600
198	241	1912	6696	7682	1957	2208	7600
	283	1928	6651	7663	1965	2241	7572
407	498	1992	6670	7643	2039	2296	7531
1850	1810	1947	6795	7662	1975	2243	7579
1823	1806	1818	6684	7603	1752	2122	7567
1818	1801	1813	6679	7600	1747	2117	7566
1824	1807	1819	6685	7606	1753	2123	7572
1818	1801	1813	6679	7600	1747	2117	7566
1823	1806	1818	6684	7605	1752	2122	7571
1800	1836	1776	6733	7660	1933	2071	7602

1986	2007	1856	6845	7712	1966	2258	7616
1900	1827	1848	6749	7657	1895	1926	7557
1899	1826	1847	6748	7655	1894	1925	7557
1892	1819	1842	6740	7652	1887	1918	7552
1894	1821	1844	6743	7654	1889	1920	7554
1854	1781	1788	6770	7645	1887	1911	7561
1863	1790	1797	6779	7654	1896	1920	7570
1863	1790	1797	6779	7654	1896	1920	7570
1855	1782	1787	6745	7628	1858	1864	7526
1886	1813	1808	6760	7640	1881	1881	7538
1828	1783	1823	6735	7613	1865	1894	7513
1847	1774	1779	6737	7620	1850	1856	7518
1911	1852	1849	6747	7649	1880	1906	7557
1911	1852	1849	6747	7649	1880	1906	7557
1900	1841	1838	6736	7639	1869	1895	7547
1869	1800	1843	6733	7636	1873	1921	7566
1906	1884	1827	6799	7626	1868	2105	7539
1937	1915	1858	6827	7655	1899	2136	7568
1908	1886	1829	6801	7628	1870	2107	7541
1906	1884	1827	6799	7626	1868	2105	7539
2250	2187	2142	6843	7679	2122	17	7578
2252	2189	2144	6845	7681	2124	19	7580
2246	2183	2138	6839	7675	2118	13	7574
2243	2180	2135	6836	7672	2115	10	7571
2247	2184	2162	6848	7683	2109	44	7582
2241	2178	2135	6842	7674	2113		7573
2245	2182	2137	6838	7674	2117	12	7573
2254	2191	2152	6849	7679	2130	27	7578
2245	2182	2137	6838	7674	2117	12	7573
2244	2181	2136	6837	7673	2116	11	7572
2215	2194	2147	6836	7676	2089	84	7569
2266	2203	2204	6821	7683	2154	99	7582
2265	2228	2137	6842	7666	2062	128	7565
2265	2228	2137	6842	7666	2062	128	7565
2265	2228	2137	6842	7666	2062	128	7565
2283	2255	2128	6813	7730	2074	1674	7654
2241	2190	2028	6813	7727	2037	1588	7645
1993	1982	1967	6686	7652	1980	1903	7576
1985	1974	1957	6688	7653	1960	1883	7577
1985	1974	1957	6688	7653	1960	1883	7577
1986	1975	1973	6689	7651	1966	1883	7575
2090	2051	1907	6807	7648	1870	2022	7548
2257	2230	1933	6807	7710	2201	1884	7656
2338	2286	2053	6792	7747	2213	2005	7693
2389	2337	2076	6798	7754	2238	1982	7700
2389	2337	2076	6798	7754	2238	1982	7700
2376	2328	2124	6814	7758	2303	2053	7702
2335	2283	2061	6806	7760	2272	2017	7706
2378	2326	2094	6797	7726	2166	1915	7672
2377	2329	2175	6772	7727	2204	1926	7669
2445	2391	2218	6867	7804	2246	1871	7697
2467	2413	2253	6878	7803	2255	1924	7696
1886	1880	515	6724	7637	1861	2104	7528
1899	1893	511	6749	7658	1872	2118	7549
1877	1856	423	6759	7651	1808	2042	7554

1881	1860	425	6762	7653	1810	2044	7556
1912	1891	387	6760	7656	1818	2076	7559
1998	1976	465	6782	7631	1876	2088	7528
1928	1907	20	6787	7651	1887	2135	7556
1928	1907		6789	7653	1887	2135	7558
1963	1948	332	6809	7651	1918	2078	7552
1965	1950	334	6811	7653	1920	2080	7554
1965	1950	334	6811	7651	1920	2080	7552
1980	1965	353	6806	7637	1946	2101	7538
1952	1937	327	6806	7649	1923	2075	7550
1984	1969	397	6822	7637	1931	2069	7538
1985	1970	398	6823	7638	1932	2070	7539
1910	1869	607	6778	7666	1887	2079	7565
1900	1859	603	6776	7667	1881	2069	7566
1963	1964	1885	6739	7676	28	2113	7610
1965	1966	1887	6739	7678		2113	7612
1955	1956	1877	6731	7668	36	2104	7602
1954	1955	1876	6730	7667	35	2104	7601
1962	1969	1904	6715	7676	67	2132	7610
1954	1955	1878	6730	7667	35	2104	7601
1955	1956	1877	6731	7668	34	2105	7602
1969	1970	1891	6745	7680	50	2119	7614
1985	1986	1913	6746	7657	85	2151	7591
2062	2062	1928	6780	7649	183	2152	7649
2182	2137	2127	6738	7785	2093	2362	7737
2190	2143	2147	6745	7790	2102	2376	7740
2200	2155	2143	6755	7800	2109	2380	7752
2175	2130	2088	6739	7774	2078	2343	7726
1992	1947	1975	6800	7773	1953	2219	7723
1985	1940	1970	6800	7767	1946	2226	7717
1985	1940	1970	6800	7767	1946	2226	7717
1983	1938	1968	6797	7764	1944	2224	7714
1997	1952	1976	6808	7767	1944	2234	7717
1979	1934	1936	6806	7741	1929	2210	7691
1961	1916	1946	6806	7772	1918	2200	7722
2060	2015	2037	6790	7736	1989	2280	7689
1923	1960	2003	6795	7746	1946	2249	7678
1953	1908	1948	6791	7769	1916	2196	7717
1962	1923	1977	6824	7770	1925	2219	7703
1982	1937	1969	6816	7774	1939	2221	7722
1987	1942	1974	6822	7778	1944	2226	7726
1962	1927	1967	6818	7753	1934	2213	7691
1994	1949	1973	6801	7773	1943	2223	7721
1979	1934	1964	6796	7762	1936	2220	7710
1885	1840	1889	6777	7731	1882	2177	7677
1897	1852	1895	6782	7734	1885	2188	7680
1968	2020	2066	6713	7720	2053	2386	7681
1977	2029	2075	6722	7729	2062	2395	7690
1961	2013	2059	6706	7713	2046	2379	7674
1972	1941	1916	6763	7730	1961	2248	7684
1893	1848	1897	6785	7727	1893	2198	7673
1894	1849	1898	6786	7730	1893	2198	7676
2058	2085	2133	6737	7647	2116	2454	7561
1942	1963	2018	6767	7744	1984	2309	7680
1941	1962	2013	6766	7734	1983	2314	7670

1949	1970	2029	6787	7743	1993	2325	7679
1954	1975	2032	6782	7750	1996	2328	7686
2000	1952	1998	6729	7711	2019	2300	7676
1964	1919	1968	6802	7755	1963	2267	7701
1957	1912	1958	6795	7745	1956	2260	7691
1955	1910	1956	6793	7743	1954	2258	7689
1897	1852	1888	6774	7726	1902	2181	7672
1901	1856	1899	6776	7706	1889	2192	7652
1915	1870	1898	6797	7751	1918	2196	7697
1906	1861	1891	6790	7744	1911	2189	7690
1843	1798	1812	6767	7710	1806	2099	7660
1843	1798	1812	6767	7710	1806	2099	7660
1929	1882	1932	6782	7729	1932	2239	7675
1924	1877	1927	6777	7724	1927	2234	7670
1927	1880	1930	6780	7727	1930	2237	7673
1831	1849	1831	6700	7618	1881	2189	7515
1832	1850	1830	6699	7615	1882	2190	7512
1819	1837	1819	6688	7605	1869	2177	7502
2069	2080	2037	6788	7705	2127	2350	7598
1878	1901	1865	6714	7634	1932	2176	7527
2163	2174	2041	6954	7859	2105	2447	7751
3771	3805	3760	7013	7834	3793	3833	7766
6577	6586	6704	281	7951	6666	6758	7903
6579	6588	6706	283	7953	6668	6760	7905
6576	6585	6703	280	7950	6665	6757	7902
6578	6587	6705	282	7952	6667	6759	7904
6640	6649	6771	175	7920	6722	6821	7872
6627	6636	6760	162	7938	6708	6810	7890
6635	6644	6768	162	7949	6716	6814	7901
6661	6670	6792	60	7932	6752	6850	7880
6675	6684	6806	76	7946	6766	6864	7895
6651	6660	6789		7935	6739	6842	7884
6695	6704	6818	138	7945	6790	6878	7897
6653	6662	6784	86	7924	6736	6836	7873
6683	6692	6814	116	7953	6766	6866	7902
6636	6645	6771	103	7911	6719	6817	7866
6657	6666	6788	90	7924	6738	6832	7873
6659	6668	6790	92	7928	6742	6842	7877
6817	6837	6921	429	8051	6914	6968	8008
6812	6832	6916	424	8046	6909	6963	8003
6761	6798	6888	419	8034	6872	6934	7966
6648	6682	6809	301	7965	6762	6840	7902
6796	6808	6911	441	8052	6884	6953	8005
6666	6664	6796	256	7941	6755	6839	7891
6660	6658	6790	250	7935	6749	6833	7885
6659	6657	6789	255	7936	6748	6830	7886
6636	6634	6761	326	7907	6725	6806	7857
6682	6680	6803	528	7816	6781	6852	7764
3478	3479	3352	6935	7847	3559	3803	7789
3467	3468	3341	6925	7836	3548	3792	7778
7575	7614	7561	7887	385	7615	7576	4
7575	7614	7561	7887	385	7615	7576	4
7574	7613	7560	7886	384	7614	7575	3
7573	7612	7559	7885	384	7613	7574	3
7575	7614	7561	7887	384	7615	7576	3

7573	7612	7559	7887	384	7613	7574	3
7575	7614	7561	7887	384	7615	7576	3
7573	7612	7559	7885	382	7613	7574	1
7576	7615	7562	7888	385	7616	7577	4
7573	7612	7559	7885	382	7613	7574	1
7572	7611	7558	7884	381	7612	7573	
7573	7612	7559	7885	382	7613	7574	1
7574	7613	7560	7886	383	7614	7575	2
7574	7613	7560	7886	383	7614	7575	2
7694	7721	7698	7929	107	7713	7713	282
7698	7725	7702	7933	111	7717	7717	286
7698	7725	7702	7933	109	7717	7717	286
7701	7728	7705	7936	112	7720	7720	289
7663	7682	7653	7935		7678	7674	381
7695	7722	7699	7930	112	7714	7714	283
7687	7714	7690	7924	182	7705	7707	259
7685	7712	7688	7923	180	7703	7705	257
7690	7717	7693	7927	184	7708	7710	261
7685	7712	7688	7922	175	7703	7705	256
7687	7714	7690	7924	183	7705	7707	256
7691	7718	7695	7927	191	7711	7711	266
7602	7579	7573	7877	349	7604	7564	320
7597	7623	7598	7858	874	7619	7620	811
7597	7623	7608	7850	941	7615	7624	876
7730	7758	7750	7951	1995	7748	7755	2042
7730	7758	7750	7951	1995	7748	7755	2042
7735	7763	7755	7958	2002	7753	7760	2049
7772	7798	7789	7996	2013	7788	7790	2063
7737	7753	7764	7934	2108	7778	7780	2144
7579	7633	7611	7699	3342	7649	7620	3406
7582	7636	7614	7702	3345	7652	7623	3409
7587	7641	7621	7710	3353	7657	7632	3413
7586	7644	7613	7687	3377	7654	7627	3430
7558	7610	7585	7730	3301	7623	7604	3361
7566	7618	7593	7738	3309	7631	7612	3369
7557	7609	7584	7729	3300	7622	7603	3360
7630	7684	7661	7736	3348	7697	7680	3412
7632	7686	7663	7738	3350	7699	7682	3414
7636	7690	7667	7743	3355	7703	7686	3419
7649	7703	7680	7755	3367	7716	7699	3431
7567	7622	7584	7737	3359	7645	7630	3422
7568	7623	7585	7738	3360	7646	7631	3423
7568	7623	7585	7738	3360	7646	7631	3423
7569	7624	7586	7739	3361	7647	7632	3424
7567	7622	7584	7737	3359	7645	7630	3422
7569	7624	7586	7739	3359	7647	7632	3422
7593	7626	7600	7770	3429	7658	7655	3498
7592	7625	7599	7769	3428	7657	7654	3497
7591	7624	7598	7768	3427	7656	7653	3496
7590	7623	7597	7767	3426	7655	7652	3495
7592	7625	7599	7769	3428	7657	7654	3497
7591	7624	7598	7768	3427	7656	7653	3496
7572	7605	7575	7795	3458	7633	7630	3528
7594	7627	7601	7771	3430	7659	7656	3499
7678	7712	7703	7797	3478	7724	7724	3521

7664	7696	7653	7955	3357	7692	7696	3409
7667	7699	7656	7960	3360	7695	7699	3412
7665	7697	7654	7958	3358	7693	7697	3410
7662	7694	7651	7953	3355	7690	7694	3407
24960	24958	24975	25018	25546	25041	24969	25531
24959	24957	24974	25017	25545	25040	24968	25530
30999	30987	30997	30810	31166	30983	30944	31135

2010031282	SCK02-43	SCP21-24	SCP29-34	ERS1340929	ERS1340998	ERS1341034	SCK53-23
7531	3735	3593	3531	7564	3768	3773	7708
7468	3656	3525	3494	7513	3686	3719	7638
7468	3656	3523	3492	7515	3688	3719	7640
7515	3707	3549	3546	7545	3714	3791	7681
7521	3685	3540	3510	7561	3715	3773	7689
7488	3698	3562	3541	7540	3727	3807	7654
7488	3698	3562	3541	7540	3727	3807	7654
7496	3709	3580	3547	7562	3743	3764	7666
7499	3712	3579	3549	7566	3746	3766	7672
7448	3747	3584	3597	7516	3760	3840	7648
7441	3746	3621	3622	7524	3764	3806	7641
7480	3720	3587	3585	7565	3744	3801	7678
7580	3820	3700	3665	7610	3848	3927	7745
7580	3820	3700	3665	7610	3848	3927	7745
7532	3765	3637	3600	7561	3786	3857	7695
7536	3763	3626	3597	7566	3783	3854	7699
7479	3659	3505	3490	7530	3678	3735	7655
7512	3702	3546	3521	7546	3715	3811	7681
7491	3746	3606	3604	7549	3748	3758	7671
7481	3796	3633	3643	7541	3791	3822	7660
7512	3782	3648	3657	7566	3812	3815	7692
7501	3673	3541	3527	7540	3692	3715	7668
7498	3714	3610	3602	7539	3743	3828	7692
7521	3739	3630	3629	7568	3758	3859	7703
7488	3653	3527	3530	7521	3666	3706	7648
7533	3682	3574	3545	7583	3711	3809	7700
7550	3633	3558	3512	7585	3693	3664	7724
7536	3738	3693	3634	7581	3791	3884	7737
7478	3706	3629	3620	7528	3780	3780	7653
7496	3670	3578	3527	7525	3698	3714	7676
7496	3670	3578	3527	7525	3698	3714	7676
7499	3673	3581	3529	7528	3701	3716	7679
7496	3696	3603	3573	7572	3716	3698	7697
7541	3629	3558	3514	7578	3688	3661	7715
7541	3629	3558	3514	7578	3688	3661	7715
7615	2458	1969	1959	7630	2225	2074	7762
7613	2443	1945	1950	7629	2194	2086	7757
7614	2439	1935	1930	7629	2200	2077	7761
7612	2413	1974	1969	7636	2191	2085	7763
7598	2439	1933	1932	7613	2194	2060	7747
7605	2441	1943	1928	7631	2206	2078	7756
7606	2440	1942	1927	7630	2205	2077	7757
7601	2441	1925	1937	7627	2204	2082	7760
7601	2447	1965	1960	7623	2221	2055	7759
7573	2467	1985	1962	7582	2254	2058	7735
7532	2503	2051	2042	7578	2307	2102	7712
7580	2351	1946	1958	7619	2258	2206	7760
7568	2283	1865	1761	7567	2141	2124	7708
7567	2278	1860	1756	7564	2136	2119	7705
7573	2284	1866	1762	7570	2142	2125	7711
7567	2278	1860	1756	7564	2136	2119	7705
7572	2283	1865	1761	7569	2141	2124	7710
7603	2307	1813	1942	7625	2076	2111	7775

7617	2260	2086	1969	7673	2277	2117	7820
7558	2157	1772	1906	7617	1919	2020	7727
7558	2156	1771	1905	7616	1918	2019	7725
7553	2149	1764	1898	7609	1911	2012	7720
7555	2151	1766	1900	7611	1913	2014	7722
7562	2185	1774	1898	7613	1904	1983	7712
7571	2194	1783	1907	7622	1913	1992	7721
7571	2194	1783	1907	7622	1913	1992	7721
7527	2110	1703	1869	7595	1857	1955	7698
7539	2127	1722	1892	7604	1874	1980	7711
7514	2115	1742	1868	7577	1887	1938	7685
7519	2102	1695	1861	7587	1849	1947	7690
7558	2150	1745	1889	7616	1899	1938	7727
7558	2150	1745	1889	7616	1899	1938	7727
7548	2139	1734	1878	7608	1888	1927	7717
7567	2142	1716	1882	7611	1914	1929	7711
7540	2148	1909	1877	7599	2124	2047	7738
7569	2179	1940	1908	7628	2155	2078	7765
7542	2150	1911	1879	7601	2126	2049	7740
7540	2148	1909	1877	7599	2124	2047	7738
7579	1922	1892	2141	7628	26	2463	7767
7581	1925	1894	2143	7630	28	2465	7769
7575	1919	1888	2137	7624	22	2459	7763
7572	1916	1885	2134	7621	19	2456	7760
7583	1948	1893	2128	7630	63	2438	7771
7574	1924	1883	2132	7623	27	2454	7760
7574	1918	1887	2136	7623	21	2458	7762
7579	1923	1876	2149	7624		2467	7767
7574	1918	1887	2136	7623	21	2458	7762
7573	1917	1886	2135	7622	20	2457	7761
7570	1890	1881	2094	7619	93	2434	7760
7583	1917	1902	2173	7635	108	2443	7778
7566	1913	1885	2083	7614	137	2464	7753
7566	1913	1885	2083	7614	137	2464	7753
7566	1913	1885	2083	7614	137	2464	7753
7655	1654	1961	2073	7693	1693	2396	7809
7646	1721	1889	2028	7688	1607	2325	7809
7577	2048	32	1993	7624	1896	2100	7753
7578	2028		1973	7623	1876	2099	7750
7578	2028	10	1973	7623	1876	2099	7750
7576	2018	74	1979	7619	1874	2091	7750
7549	1965	1347	1865	7633	2035	2144	7749
7657	1909	2060	2210	7653	1867	2430	7801
7694	1764	2181	2222	7700	1988	2558	7833
7701	1757	2174	2247	7708	1989	2611	7840
7701	1757	2174	2247	7708	1989	2611	7840
7703	1817	2213	2314	7713	2046	2601	7852
7707	1747	2136	2281	7715	2000	2573	7852
7673	1652	2120	2175	7673	1898	2556	7798
7670	1786	2149	2213	7681	1909	2551	7817
7698	160	2028	2237	7763	1870	2527	7896
7697		2028	2246	7755	1923	2524	7898
7529	2245	1995	1844	7602	2115	2005	7738
7550	2263	2006	1855	7623	2135	2021	7757
7555	2215	1939	1827	7610	2061	1999	7744

7557	2217	1941	1829	7612	2063	2001	7746
7560	2241	1969	1837	7620	2095	2023	7749
7529	2200	2057	1889	7577	2107	2060	7725
7557	2251	1957	1904	7612	2152	2133	7753
7559	2253	1957	1904	7614	2152	2133	7755
7553	2255	2041	1937	7623	2097	2110	7749
7555	2257	2043	1939	7625	2099	2112	7751
7553	2257	2043	1939	7623	2099	2112	7749
7539	2281	2067	1965	7619	2120	2084	7737
7551	2260	2040	1942	7623	2094	2112	7751
7539	2296	2030	1950	7600	2088	2140	7735
7540	2297	2031	1951	7601	2089	2141	7734
7566	2132	1823	1904	7635	2098	1997	7782
7567	2136	1815	1898	7636	2088	1998	7783
7611	2253	1958	65	7650	2130	2114	7751
7613	2255	1960	67	7652	2130	2116	7753
7603	2245	1950	59	7642	2121	2106	7743
7602	2244	1949	58	7641	2121	2105	7742
7611	2246	1973		7654	2149	2113	7751
7602	2244	1949	48	7641	2121	2105	7742
7603	2245	1950	57	7642	2122	2106	7743
7615	2259	1964	73	7654	2136	2120	7755
7592	2284	1993	108	7639	2166	2111	7736
7650	2300	2028	198	7634	2169	2139	7732
7738	2470	1959	2102	7725	2371	984	7905
7741	2486	1974	2107	7730	2385	978	7912
7753	2484	1977	2118	7740	2389	1000	7920
7727	2449	1952	2087	7716	2362	977	7894
7724	2355	1838	1962	7708	2234	748	7885
7718	2334	1829	1955	7706	2229	732	7879
7718	2334	1829	1955	7706	2229	732	7879
7715	2332	1827	1953	7703	2227	730	7876
7718	2346	1845	1953	7710	2243	736	7881
7692	2333	1830	1938	7689	2229	699	7853
7723	2326	1815	1927	7714	2213	705	7884
7690	2404	1891	1998	7667	2293	678	7852
7679	2363	1888	1951	7684	2262	572	7856
7718	2316	1815	1925	7712	2209	647	7876
7704	2327	1834	1934	7715	2232	659	7877
7723	2335	1842	1948	7717	2234	653	7876
7727	2340	1847	1953	7719	2239	658	7881
7692	2311	1862	1929	7700	2226	639	7858
7722	2331	1838	1952	7710	2232	689	7878
7711	2326	1833	1945	7701	2229	682	7869
7678	2260	1805	1885	7679	2190	494	7834
7681	2263	1813	1888	7682	2201	496	7837
7682	2453	2002	2052	7635	2383	624	7822
7691	2462	2011	2061	7644	2392	633	7831
7675	2446	1995	2045	7628	2376	617	7815
7685	2333	1884	1976	7671	2245	653	7837
7674	2272	1819	1896	7680	2211	496	7834
7677	2273	1820	1896	7681	2211	496	7837
7562	2524	2099	2113	7563	2467		7751
7681	2390	1964	1961	7664	2322	586	7839
7671	2393	1967	1960	7652	2327	582	7831

7680	2404	1977	1970	7661	2338	600	7840
7687	2407	1980	1973	7668	2341	595	7847
7677	2382	1958	1994	7655	2311	607	7827
7702	2344	1896	1966	7684	2280	578	7860
7692	2337	1889	1959	7672	2273	571	7850
7690	2335	1887	1957	7670	2271	569	7848
7673	2250	1809	1905	7675	2194	503	7827
7653	2265	1816	1892	7662	2205	470	7809
7698	2279	1833	1921	7700	2207	532	7854
7691	2272	1824	1914	7693	2200	523	7847
7661	2203	1769	1809	7665	2112	703	7816
7661	2203	1769	1809	7665	2112	703	7816
7676	2308	1871	1933	7677	2250	543	7836
7671	2303	1866	1928	7672	2245	538	7831
7674	2306	1869	1931	7675	2248	541	7834
7516	2384	1887	1884	7549	2200	2079	7704
7513	2385	1888	1885	7546	2201	2080	7699
7503	2372	1875	1872	7537	2188	2067	7691
7599	2491	2078	2130	7620	2349	2241	7776
7528	2399	1919	1935	7543	2185	2061	7700
7752	2595	2186	2108	7781	2458	2358	7932
7767	3903	3853	3812	7721	3832	3995	7929
7904	6784	6612	6642	7745	6767	6858	7973
7906	6786	6614	6644	7747	6769	6860	7975
7903	6783	6611	6641	7744	6766	6857	7972
7905	6785	6613	6643	7746	6768	6859	7974
7873	6862	6662	6698	7706	6830	6776	7951
7891	6846	6650	6684	7712	6819	6786	7965
7902	6856	6656	6692	7721	6823	6791	7974
7881	6886	6698	6728	7698	6859	6770	7956
7896	6902	6712	6742	7715	6873	6784	7973
7885	6878	6688	6715	7702	6849	6737	7958
7898	6920	6728	6766	7713	6887	6780	7973
7874	6880	6682	6712	7691	6845	6754	7947
7903	6910	6712	6742	7720	6875	6784	7975
7867	6863	6665	6695	7682	6826	6751	7940
7874	6884	6680	6714	7693	6841	6754	7949
7878	6886	6688	6718	7697	6851	6760	7953
8009	7022	6843	6914	7807	6977	6881	8069
8004	7017	6838	6909	7802	6972	6876	8064
7967	6980	6805	6874	7786	6943	6836	8050
7903	6884	6705	6738	7721	6849	6759	7985
8006	7011	6825	6878	7828	6962	6865	8081
7892	6892	6712	6737	7696	6848	6753	7960
7886	6886	6706	6731	7690	6842	6747	7954
7887	6885	6705	6730	7691	6839	6754	7955
7858	6857	6683	6707	7656	6815	6719	7928
7765	6896	6726	6763	7587	6861	6742	7831
7790	3817	3637	3546	7769	3814	3747	7899
7779	3806	3626	3535	7756	3803	3736	7888
5	7699	7580	7613	3413	7581	7564	2053
5	7699	7580	7613	3413	7581	7564	2053
4	7698	7579	7612	3412	7580	7563	2052
4	7697	7578	7611	3412	7579	7562	2052
4	7697	7580	7613	3412	7581	7564	2052

4	7697	7578	7611	3410	7579	7562	2052
4	7699	7580	7613	3412	7581	7564	2052
2	7697	7578	7611	3410	7579	7562	2050
5	7700	7581	7614	3413	7582	7565	2053
2	7697	7578	7611	3410	7579	7562	2050
1	7696	7577	7610	3409	7578	7561	2049
	7697	7578	7611	3410	7579	7562	2050
3	7698	7579	7612	3411	7580	7563	2051
3	7698	7579	7612	3411	7580	7563	2051
283	7846	7692	7711	3276	7718	7684	1917
287	7850	7696	7715	3280	7722	7688	1921
287	7850	7696	7715	3280	7722	7688	1921
290	7853	7699	7718	3283	7725	7691	1924
382	7803	7653	7676	3345	7679	7647	2002
284	7847	7693	7712	3277	7719	7685	1918
260	7838	7684	7703	3281	7712	7634	1889
258	7836	7682	7701	3279	7710	7632	1887
262	7841	7687	7706	3283	7715	7637	1891
257	7836	7682	7701	3278	7710	7632	1886
257	7838	7684	7703	3280	7712	7634	1888
267	7842	7688	7709	3280	7716	7639	1898
321	7689	7547	7590	3368	7569	7556	2050
812	7757	7596	7619	3454	7627	7577	2122
877	7761	7598	7615	3495	7631	7581	2182
2043	7893	7745	7746	3417	7762	7746	15
2043	7893	7745	7746	3417	7762	7746	15
2050	7898	7750	7751	3425	7767	7751	
2064	7929	7777	7788	3443	7797	7779	224
2145	7905	7772	7774	3391	7785	7747	1924
3407	7752	7620	7651	11	7621	7560	3422
3410	7755	7623	7654		7624	7563	3425
3414	7760	7628	7659	62	7633	7571	3429
3431	7755	7620	7658	409	7628	7570	3462
3362	7723	7600	7625	287	7605	7542	3352
3370	7731	7608	7633	295	7613	7550	3360
3361	7722	7599	7624	286	7604	7541	3351
3413	7791	7674	7699	635	7681	7611	3391
3415	7793	7676	7701	637	7683	7613	3393
3420	7797	7680	7705	642	7687	7618	3398
3432	7810	7693	7718	654	7700	7630	3409
3423	7758	7588	7645	2342	7633	7553	3471
3424	7759	7589	7646	2343	7634	7554	3472
3424	7759	7589	7646	2343	7634	7554	3472
3425	7760	7590	7647	2344	7635	7555	3473
3423	7758	7588	7645	2342	7633	7553	3471
3423	7760	7589	7647	2340	7635	7554	3471
3499	7762	7625	7658	2646	7656	7629	3386
3498	7761	7624	7657	2645	7655	7628	3385
3497	7760	7623	7656	2644	7654	7627	3384
3496	7759	7622	7655	2643	7653	7626	3383
3498	7761	7624	7657	2645	7655	7628	3385
3497	7760	7623	7656	2644	7654	7627	3384
3529	7737	7600	7633	2675	7631	7654	3416
3500	7763	7626	7659	2647	7657	7630	3387
3522	7843	7689	7726	2643	7725	7669	3486

3410	7815	7701	7694	2462	7697	7652	3323
3413	7818	7704	7697	2465	7700	7655	3326
3411	7816	7702	7695	2463	7698	7653	3324
3408	7813	7700	7692	2462	7695	7650	3324
25532	25012	24973	25036	25521	24967	25035	25600
25531	25011	24972	25035	25520	24966	25034	25599
31136	30934	30956	30975	31216	30948	30989	31276

SCP24-18	E.coli_l	509sc	ME160327	AM_LREC-61	AM_LREC-98	AM_LREC-15	AM_LREC-63
7689	6821	3536	3596	7610	7534	7614	7613
7640	6740	3469	3552	7559	7471	7563	7562
7640	6744	3469	3552	7559	7471	7563	7562
7663	6785	3509	3576	7602	7518	7606	7605
7677	6780	3495	3571	7612	7524	7616	7615
7634	6779	3523	3596	7579	7491	7583	7582
7634	6779	3523	3596	7579	7491	7583	7582
7677	6778	3526	3608	7587	7499	7591	7590
7679	6780	3528	3611	7591	7502	7595	7594
7624	6820	3570	3632	7559	7451	7563	7562
7647	6801	3595	3655	7552	7444	7556	7555
7669	6800	3548	3630	7591	7483	7595	7594
7720	6857	3648	3719	7656	7583	7660	7659
7720	6857	3648	3719	7656	7583	7660	7659
7674	6831	3583	3650	7608	7535	7612	7611
7678	6843	3572	3648	7612	7539	7616	7615
7641	6773	3456	3522	7574	7482	7578	7577
7665	6767	3505	3571	7590	7515	7594	7593
7678	6790	3584	3634	7583	7494	7587	7586
7648	6802	3608	3648	7572	7484	7576	7575
7697	6812	3593	3675	7605	7515	7609	7608
7645	6774	3487	3531	7590	7504	7594	7593
7657	6781	3579	3630	7600	7501	7604	7603
7680	6842	3589	3661	7615	7524	7619	7618
7631	6765	3503	3538	7561	7491	7565	7564
7697	6817	3545	3604	7616	7536	7620	7619
7704	6812	3456	3595	7629	7553	7635	7634
7715	6795	3596	3704	7627	7539	7631	7630
7665	6785	3558	3673	7559	7481	7563	7562
7665	6779	3485	3589	7573	7499	7577	7576
7665	6779	3485	3589	7573	7499	7577	7576
7667	6780	3487	3592	7576	7502	7580	7579
7691	6811	3482	3577	7598	7499	7604	7603
7700	6801	3444	3601	7620	7544	7626	7625
7700	6801	3444	3601	7620	7544	7626	7625
7687	6701	191	1940	7720	7618	7725	7724
7692	6696	172	1937	7718	7616	7723	7722
7691	6694	160	1913	7719	7617	7724	7723
7694	6680	215	1950	7717	7615	7722	7721
7678	6688	146	1914	7703	7601	7708	7707
7691	6684	140	1925	7714	7608	7719	7718
7690	6683	139	1924	7715	7609	7720	7719
7687	6696		1916	7714	7604	7719	7718
7682	6717	178	1945	7717	7604	7722	7721
7662	6682	206	1965	7690	7576	7695	7694
7638	6700	439	2055	7669	7535	7675	7674
7691	6825	1792	1952	7699	7583	7706	7705
7636	6713	1755	1816	7639	7571	7645	7644
7633	6708	1750	1811	7638	7570	7644	7643
7639	6714	1756	1817	7644	7576	7650	7649
7633	6708	1750	1811	7638	7570	7644	7643
7638	6713	1755	1816	7643	7575	7649	7648
7690	6748	1767	1794	7698	7606	7706	7705

7728	6844	1952	1884	7750	7620	7758	7757
7698	6763	1830	1792	7695	7561	7701	7700
7697	6762	1829	1791	7693	7561	7699	7698
7692	6754	1822	1786	7690	7556	7696	7695
7694	6757	1824	1788	7692	7558	7698	7697
7687	6769	1786	1740	7681	7565	7687	7686
7696	6778	1795	1749	7690	7574	7696	7695
7696	6778	1795	1749	7690	7574	7696	7695
7662	6759	1787	1737	7664	7530	7670	7669
7673	6774	1818	1758	7676	7542	7682	7681
7646	6753	1803	1761	7649	7517	7655	7654
7654	6751	1779	1729	7656	7522	7662	7661
7686	6755	1853	1808	7689	7561	7695	7694
7686	6755	1853	1808	7689	7561	7695	7694
7676	6744	1842	1797	7679	7551	7685	7684
7684	6746	1799	1790	7672	7570	7678	7677
7660	6817	1841	1831	7669	7543	7676	7675
7689	6845	1872	1862	7698	7572	7705	7704
7662	6819	1843	1833	7671	7545	7678	7677
7660	6817	1841	1831	7669	7543	7676	7675
7699	6855	2200	2089	7715	7582	7719	7718
7701	6857	2202	2091	7717	7584	7721	7720
7695	6851	2196	2085	7711	7578	7715	7714
7692	6848	2193	2082	7708	7575	7712	7711
7703	6859	2197	2092	7719	7586	7723	7722
7694	6852	2191	2080	7710	7577	7714	7713
7694	6850	2195	2084	7710	7577	7714	7713
7695	6861	2204	2099	7715	7582	7719	7718
7694	6850	2195	2084	7710	7577	7714	7713
7693	6849	2194	2083	7709	7576	7713	7712
7694	6850	2187	2094	7712	7573	7716	7715
7705	6837	2216	2151	7719	7586	7723	7722
7679	6854	2215	2092	7702	7569	7706	7705
7679	6854	2215	2092	7702	7569	7706	7705
7679	6854	2215	2092	7702	7569	7706	7705
7771	6841	2273	2122	7770	7658	7776	7775
7773	6837	2225	2030	7767	7649	7773	7772
7704	6725	1933	2059	7686	7580	7692	7691
7700	6726	1925	2043	7687	7581	7693	7692
7700	6726	1925	2043	7687	7581	7693	7692
7696	6722	1926	2047	7685	7579	7691	7690
7698	6800	2051	1961	7686	7552	7692	7691
7727	6837	2235	1954	7750	7660	7756	7755
7770	6842	2316	2074	7787	7697	7793	7792
7784	6844	2367	2105	7794	7704	7800	7799
7784	6844	2367	2105	7794	7704	7800	7799
7787	6864	2362	2136	7798	7706	7804	7803
7794	6857	2313	2116	7800	7710	7806	7805
7752	6843	2356	2119	7766	7676	7772	7771
7777	6814	2355	2174	7761	7673	7769	7768
7816	6885	2419	2222	7842	7701	7848	7847
7813	6896	2441	2257	7841	7700	7847	7846
7642	6764	1863	560	7675	7532	7681	7680
7662	6781	1876	573	7696	7553	7702	7701
7651	6778	1834	475	7689	7558	7695	7694

7653	6781	1838	477	7691	7560	7697	7696
7659	6779	1869	439	7694	7563	7700	7699
7623	6797	1944	463	7665	7532	7671	7670
7649	6801	1885	334	7691	7560	7697	7696
7651	6803	1885	334	7693	7562	7699	7698
7654	6818	1914	2	7691	7556	7697	7696
7656	6820	1916	4	7693	7558	7699	7698
7654	6820	1916		7691	7556	7697	7696
7666	6804	1931	40	7677	7542	7683	7682
7657	6816	1903	21	7689	7554	7695	7694
7639	6832	1933	117	7677	7542	7683	7682
7640	6833	1934	118	7678	7543	7684	7683
7674	6802	1847	643	7705	7569	7710	7709
7678	6803	1837	641	7706	7570	7711	7710
7688	6781	1936	1920	7706	7614	7712	7711
7690	6781	1938	1920	7708	7616	7714	7713
7680	6773	1928	1912	7698	7606	7704	7703
7679	6772	1927	1911	7697	7605	7703	7702
7692	6763	1937	1939	7706	7614	7712	7711
7679	6772	1927	1913	7697	7605	7703	7702
7680	6773	1928	1912	7698	7606	7704	7703
7692	6785	1942	1926	7710	7618	7716	7715
7695	6759	1958	1955	7687	7595	7693	7692
7672	6801	2022	1961	7679	7653	7685	7684
7793	6788	2117	2141	7819	7741	7827	7826
7800	6792	2120	2149	7824	7744	7832	7831
7810	6803	2135	2157	7834	7756	7842	7841
7788	6787	2110	2102	7808	7730	7816	7815
7779	6840	1927	1997	7805	7727	7813	7812
7770	6845	1920	1992	7799	7721	7807	7806
7770	6845	1920	1992	7799	7721	7807	7806
7767	6841	1918	1990	7796	7718	7804	7803
7774	6843	1932	1996	7799	7721	7807	7806
7771	6821	1914	1955	7773	7695	7781	7780
7780	6844	1896	1968	7804	7726	7812	7811
7744	6842	1995	2043	7775	7693	7776	7775
7752	6844	1962	1997	7778	7682	7786	7785
7778	6845	1888	1942	7801	7721	7809	7808
7792	6844	1892	1949	7802	7707	7810	7809
7794	6849	1917	1965	7806	7726	7814	7813
7796	6855	1922	1970	7810	7730	7818	7817
7777	6830	1925	1951	7785	7695	7793	7792
7777	6852	1929	1965	7805	7725	7813	7812
7768	6843	1914	1960	7794	7714	7802	7801
7738	6829	1814	1881	7763	7681	7771	7770
7740	6831	1826	1875	7766	7684	7774	7773
7742	6849	2020	2054	7743	7685	7751	7750
7751	6858	2029	2063	7752	7694	7760	7759
7735	6842	2013	2047	7736	7678	7744	7743
7743	6853	1921	1904	7762	7688	7770	7769
7741	6824	1822	1883	7759	7677	7767	7766
7742	6825	1823	1884	7762	7680	7770	7769
7650	6742	2082	2112	7637	7565	7685	7684
7725	6817	1965	1996	7764	7684	7772	7771
7710	6807	1964	1993	7754	7674	7762	7761

7721	6812	1972	2003	7763	7683	7771	7770
7726	6823	1977	2006	7770	7690	7778	7777
7735	6810	1952	2011	7744	7680	7752	7751
7739	6853	1893	1966	7787	7705	7795	7794
7727	6846	1886	1956	7777	7695	7785	7784
7725	6844	1884	1954	7775	7693	7783	7782
7732	6828	1826	1891	7758	7676	7766	7765
7737	6805	1830	1884	7738	7656	7746	7745
7758	6851	1844	1916	7783	7701	7791	7790
7753	6844	1835	1909	7776	7694	7784	7783
7729	6811	1772	1806	7748	7664	7754	7753
7729	6811	1772	1806	7748	7664	7754	7753
7741	6827	1858	1926	7761	7679	7769	7768
7736	6822	1853	1921	7756	7674	7764	7763
7739	6825	1856	1924	7759	7677	7767	7766
7627	6734	1783	1852	7654	7519	7660	7659
7624	6733	1784	1851	7651	7516	7657	7656
7614	6722	1771	1840	7641	7506	7647	7646
7670	6829	2021	2043	7737	7602	7743	7742
7626	6752	1830	1878	7666	7531	7672	7671
7845	6989	2115	2029	7890	7755	7896	7895
7802	7022	3810	3802	7864	7770	7867	7866
7979	662	6611	6728	7944	7907	7952	7951
7981	664	6613	6730	7946	7909	7954	7953
7978	661	6610	6727	7943	7906	7951	7950
7980	663	6612	6729	7945	7908	7953	7952
7971	512	6674	6794	7915	7876	7919	7918
7963	524	6661	6782	7933	7894	7937	7936
7969	537	6669	6790	7944	7905	7948	7947
7949	473	6695	6816	7923	7884	7926	7925
7966	489	6709	6830	7938	7899	7941	7940
7953	528	6685	6811	7927	7888	7930	7929
7966	533	6729	6842	7934	7901	7940	7939
7942	455	6687	6808	7916	7877	7919	7918
7971	485	6717	6838	7945	7906	7948	7947
7931	470	6670	6795	7909	7870	7910	7909
7944	459	6691	6812	7916	7877	7919	7918
7948	461	6693	6814	7920	7881	7923	7922
8065	702	6846	6947	8040	8012	8046	8045
8060	697	6841	6942	8035	8007	8041	8040
8044	675	6802	6906	8024	7970	8029	8028
7982	449	6698	6833	7962	7906	7963	7962
8068	623	6814	6937	8047	8009	8050	8049
7959	292	6680	6812	7929	7895	7932	7931
7953	286	6674	6806	7923	7889	7926	7925
7954	293	6673	6805	7924	7890	7927	7926
7916	287	6650	6777	7893	7861	7898	7897
7846		6696	6820	7802	7768	7805	7804
7893	6959	3469	3291	7873	7793	7876	7875
7880	6949	3458	3280	7862	7782	7865	7864
3411	7767	7603	7555	265	8	287	286
3411	7767	7603	7555	265	8	287	286
3410	7766	7602	7554	264	7	286	285
3410	7765	7601	7553	264	7	286	285
3410	7767	7603	7555	264	7	286	285

3408	7767	7601	7553	264	7	286	285
3410	7767	7603	7555	264	7	286	285
3408	7765	7601	7553	262	5	284	283
3411	7768	7604	7556	265		287	286
3408	7765	7601	7553	262	5	284	283
3407	7764	7600	7552	261	4	283	282
3408	7765	7601	7553	262	5	284	283
3409	7766	7602	7554	263	6	285	284
3409	7766	7602	7554	263	6	285	284
3289	7804	7718	7696	83	286	11	
3293	7808	7722	7700	87	290	15	8
3293	7808	7722	7700	87	290	15	10
3296	7811	7725	7703	90	293	18	13
3355	7816	7679	7651	184	385	112	107
3290	7805	7719	7697	84	287		11
3288	7799	7711	7688	20	263	82	81
3286	7798	7709	7686	18	261	80	79
3290	7802	7714	7691		265	84	83
3285	7797	7709	7686	19	260	75	74
3287	7799	7711	7688	23	260	83	82
3289	7802	7715	7692	31	270	91	90
3372	7744	7565	7571	241	324	263	262
3478	7744	7622	7600	777	815	784	783
3521	7734	7622	7610	848	880	851	850
3316	7824	7755	7744	1884	2046	1911	1910
3316	7824	7755	7744	1884	2046	1911	1910
3324	7831	7760	7749	1891	2053	1918	1917
3320	7869	7797	7787	1907	2067	1929	1928
3335	7808	7756	7767	2017	2148	2027	2026
2459	7584	7624	7620	3280	3410	3274	3273
2462	7587	7627	7623	3283	3413	3277	3276
2464	7595	7632	7630	3287	3417	3285	3284
2467	7572	7633	7622	3310	3434	3310	3309
2356	7615	7603	7594	3235	3365	3233	3232
2364	7623	7611	7602	3243	3373	3241	3240
2355	7614	7602	7593	3234	3364	3232	3231
2448	7617	7673	7668	3282	3416	3282	3281
2450	7619	7675	7670	3284	3418	3284	3283
2455	7624	7679	7674	3289	3423	3289	3288
2467	7636	7692	7687	3301	3435	3301	3300
2515	7651	7612	7603	3302	3426	3290	3289
2516	7652	7613	7604	3303	3427	3291	3290
2516	7652	7613	7604	3303	3427	3291	3290
2517	7653	7614	7605	3304	3428	3292	3291
2515	7651	7612	7603	3302	3426	3290	3289
2517	7653	7614	7605	3302	3426	3290	3289
2600	7668	7616	7615	3380	3502	3368	3367
2599	7667	7615	7614	3379	3501	3367	3366
2598	7666	7614	7613	3378	3500	3366	3365
2597	7665	7613	7612	3377	3499	3365	3364
2599	7667	7615	7614	3379	3501	3367	3366
2598	7666	7614	7613	3378	3500	3366	3365
2629	7693	7595	7590	3410	3532	3397	3396
2601	7669	7617	7616	3381	3503	3369	3368
2613	7683	7702	7700	3388	3525	3404	3403

40	7848	7689	7656	3292	3413	3292	3291
43	7853	7692	7659	3295	3416	3295	3294
41	7851	7690	7657	3293	3414	3293	3292
	7846	7687	7654	3290	3411	3290	3289
25572	25033	24988	24978	25539	25535	25553	25552
25571	25032	24987	24977	25538	25534	25552	25551
31235	30819	31007	31007	31163	31139	31177	31177

AM_LREC-128	AM_LREC-109	ERS1724554	EDZFRVQ5	ME160633	ME160685	1508493 1
7617	7606	3685	7605	3803	7615	3768
7566	7555	3624	7543	3751	7560	3686
7566	7555	3624	7545	3751	7562	3688
7609	7598	3657	7570	3790	7596	3719
7619	7608	3637	7596	3763	7616	3700
7586	7575	3666	7551	3805	7579	3722
7586	7575	3666	7551	3805	7579	3722
7594	7583	3679	7578	3808	7603	3741
7598	7587	3681	7582	3810	7607	3744
7566	7555	3708	7536	3848	7558	3758
7559	7548	3704	7529	3851	7560	3762
7598	7587	3688	7582	3838	7606	3746
7663	7652	3772	7617	3928	7641	3851
7663	7652	3772	7617	3928	7641	3851
7615	7604	3717	7579	3858	7598	3789
7619	7608	3714	7584	3855	7602	3785
7581	7570	3591	7542	3734	7567	3681
7597	7586	3636	7564	3797	7583	3716
7590	7579	3701	7570	3833	7586	3741
7579	7568	3725	7538	3878	7568	3764
7612	7601	3745	7594	3876	7603	3815
7597	7586	3656	7545	3728	7583	3695
7607	7596	3689	7555	3826	7580	3748
7622	7611	3716	7591	3860	7611	3766
7568	7557	3595	7537	3731	7558	3633
7623	7612	3635	7598	3769	7626	3711
7638	7625	3642	7588	3712	7638	3694
7634	7623	3739	7607	3839	7634	3792
7566	7555	3695	7545	3831	7573	3781
7580	7569	3616	7557	3774	7578	3696
7580	7569	3616	7557	3774	7578	3696
7583	7572	3620	7559	3776	7581	3699
7607	7594	3613	7580	3746	7619	3714
7629	7616	3634	7582	3706	7630	3689
7629	7616	3634	7582	3706	7630	3689
7728	7717	2367	7612	2135	7676	2202
7726	7715	2358	7612	2138	7677	2193
7727	7716	2365	7613	2128	7675	2193
7725	7714	2328	7623	2137	7684	2184
7711	7700	2352	7598	2112	7661	2181
7722	7711	2366	7615	2138	7677	2199
7723	7712	2365	7614	2137	7676	2198
7722	7711	2362	7613	2117	7673	2197
7725	7714	2383	7610	2142	7669	2214
7698	7687	2376	7568	2182	7630	2247
7678	7666	2460	7567	2254	7624	2299
7709	7696	2445	7607	2147	7673	2246
7648	7636	2143	7580	2066	7608	2114
7647	7635	2138	7577	2061	7605	2109
7653	7641	2144	7583	2067	7611	2115
7647	7635	2138	7577	2061	7605	2109
7652	7640	2143	7582	2066	7610	2114
7709	7695	2082	7606	2013	7665	2071

7761	7747	2205	7644	2137	7709	2262
7704	7692	2185	7619	1994	7659	1932
7702	7690	2184	7618	1993	7658	1931
7699	7687	2179	7613	1986	7651	1924
7701	7689	2181	7615	1988	7653	1926
7690	7678	2131	7605	1947	7649	1917
7699	7687	2140	7614	1956	7658	1926
7699	7687	2140	7614	1956	7658	1926
7673	7661	2136	7585	1927	7633	1870
7685	7673	2153	7588	1952	7642	1887
7658	7646	2161	7568	1952	7615	1900
7665	7653	2128	7577	1919	7625	1862
7698	7686	2161	7611	1938	7654	1912
7698	7686	2161	7611	1938	7654	1912
7688	7676	2150	7601	1927	7646	1901
7681	7669	2182	7606	1895	7649	1927
7679	7666	2075	7579	2005	7645	2089
7708	7695	2106	7607	2036	7674	2120
7681	7668	2077	7581	2007	7647	2091
7679	7666	2075	7579	2005	7645	2089
7722	7712	2062	7636	2370	7685	53
7724	7714	2064	7640	2373	7687	55
7718	7708	2058	7634	2367	7681	49
7715	7705	2055	7631	2364	7678	46
7726	7716	2063	7640	2346	7687	
7717	7707	2053	7631	2362	7680	44
7717	7707	2057	7633	2366	7680	48
7722	7712	2046	7634	2371	7681	63
7717	7707	2057	7633	2366	7680	48
7716	7706	2056	7632	2365	7679	47
7719	7709	2005	7623	2376	7676	120
7726	7716	2045	7644	2371	7692	135
7709	7699	2066	7643	2372	7660	164
7709	7699	2066	7643	2372	7660	164
7709	7699	2066	7643	2372	7660	164
7779	7767	1407	7697	2321	7726	1682
7776	7764	1638	7689	2244	7728	1596
7695	7683	2225	7590	1977	7677	1913
7696	7684	2213	7589	1959	7674	1893
7696	7684	2213	7589	1959	7674	1893
7694	7682	2185	7586	1951	7670	1889
7695	7683	2015	7617	2146	7679	2052
7759	7747	459	7652	2325	7702	1908
7796	7784	214	7698	2443	7741	2029
7803	7791	237	7716	2464	7749	2006
7803	7791	237	7716	2464	7749	2006
7807	7795		7713	2483	7748	2063
7809	7797	156	7718	2449	7756	2049
7775	7763	353	7691	2425	7714	1939
7772	7758	774	7705	2442	7724	1950
7851	7839	1778	7766	2491	7792	1895
7850	7838	1817	7759	2470	7791	1948
7684	7672	2073	7569	2094	7647	2114
7705	7693	2088	7587	2113	7668	2129
7698	7686	2001	7575	2039	7655	2054

7700	7688	2003	7577	2043	7657	2056
7703	7691	2019	7585	2051	7665	2088
7674	7662	2078	7565	2095	7630	2100
7700	7688	2124	7583	2127	7659	2162
7702	7690	2124	7585	2127	7661	2162
7700	7688	2134	7604	2139	7668	2090
7702	7690	2136	7606	2141	7670	2092
7700	7688	2136	7604	2141	7668	2092
7686	7674	2147	7600	2162	7666	2113
7698	7686	2129	7604	2138	7670	2087
7686	7674	2111	7586	2140	7646	2081
7687	7675	2112	7587	2141	7647	2082
7713	7702	2064	7590	1992	7678	2081
7714	7703	2054	7591	1986	7679	2071
7715	7703	2301	7644	2091	7695	2109
7717	7705	2303	7646	2093	7697	2109
7707	7695	2293	7636	2083	7687	2100
7706	7694	2292	7635	2082	7686	2100
7715	7703	2314	7646	2102	7699	2128
7706	7694	2292	7635	2082	7686	2100
7707	7695	2293	7636	2083	7687	2101
7719	7707	2307	7650	2097	7699	2115
7696	7684	2329	7633	2097	7686	2147
7688	7676	2355	7634	2105	7679	2148
7830	7816	2483	7680		7774	2346
7835	7821	2499	7687	46	7779	2360
7845	7831	2501	7695	44	7789	2364
7819	7805	2474	7671	73	7765	2327
7816	7802	2334	7680	334	7759	2203
7810	7796	2329	7676	331	7757	2210
7810	7796	2329	7676	331	7757	2210
7807	7793	2327	7673	329	7754	2208
7810	7796	2343	7680	337	7761	2218
7784	7770	2327	7661	359	7740	2194
7815	7801	2311	7684	339	7765	2184
7779	7772	2408	7645	479	7716	2264
7789	7775	2368	7663	528	7735	2233
7812	7798	2325	7686	415	7763	2180
7813	7799	2350	7691	483	7766	2203
7817	7803	2350	7693	440	7768	2205
7821	7807	2355	7695	445	7770	2210
7796	7782	2346	7672	494	7751	2221
7816	7802	2350	7684	414	7761	2207
7805	7791	2345	7675	411	7752	2204
7774	7760	2341	7648	544	7728	2161
7777	7763	2352	7651	553	7731	2172
7754	7740	2498	7608	769	7684	2370
7763	7749	2507	7617	778	7693	2379
7747	7733	2491	7601	762	7677	2363
7773	7759	2392	7644	681	7718	2232
7770	7756	2354	7647	575	7731	2186
7773	7759	2354	7650	575	7732	2186
7688	7634	2601	7554	984	7611	2438
7775	7761	2453	7639	733	7715	2302
7765	7751	2456	7627	738	7703	2307

7774	7760	2466	7638	756	7712	2321
7781	7767	2469	7643	751	7719	2320
7755	7741	2485	7631	742	7708	2295
7798	7784	2424	7655	655	7735	2260
7788	7774	2417	7643	648	7723	2253
7786	7772	2415	7641	646	7721	2251
7769	7755	2363	7644	568	7724	2185
7749	7735	2353	7633	557	7711	2176
7794	7780	2373	7668	586	7749	2195
7787	7773	2368	7661	577	7742	2188
7757	7745	2269	7634	692	7711	2087
7757	7745	2269	7634	692	7711	2087
7772	7758	2397	7649	594	7729	2231
7767	7753	2392	7644	589	7724	2226
7770	7756	2395	7647	592	7727	2229
7663	7651	2329	7525	2103	7597	2193
7660	7648	2330	7522	2104	7594	2194
7650	7638	2317	7512	2091	7585	2181
7746	7734	2475	7574	2142	7676	2358
7675	7663	2333	7528	2057	7599	2187
7899	7887	2595	7769	2341	7838	2446
7870	7860	3829	7754	4030	7776	3836
7955	7941	6721	7766	6689	7776	6765
7957	7943	6723	7768	6691	7778	6767
7954	7940	6720	7765	6688	7775	6764
7956	7942	6722	7767	6690	7777	6766
7922	7912	6794	7740	6756	7736	6828
7940	7930	6787	7746	6745	7742	6817
7951	7941	6787	7755	6751	7749	6821
7929	7920	6817	7734	6761	7732	6857
7944	7935	6833	7749	6775	7749	6871
7933	7924	6814	7738	6738	7736	6848
7943	7931	6859	7751	6801	7747	6885
7922	7913	6809	7725	6753	7725	6843
7951	7942	6839	7754	6783	7754	6873
7913	7906	6792	7716	6754	7716	6824
7922	7913	6807	7727	6751	7727	6839
7926	7917	6815	7731	6759	7731	6849
8049	8037	6962	7860	6934	7856	6975
8044	8032	6957	7855	6929	7851	6970
8032	8021	6928	7833	6888	7833	6941
7966	7961	6816	7764	6794	7755	6847
8053	8044	6936	7870	6897	7863	6960
7935	7926	6827	7750	6775	7730	6846
7929	7920	6821	7744	6769	7724	6840
7930	7921	6820	7745	6768	7725	6837
7901	7890	6796	7703	6737	7686	6811
7808	7799	6864	7652	6788	7617	6859
7878	7870	3655	7771	3729	7825	3799
7867	7859	3644	7760	3718	7812	3788
290	260	7705	3427	7740	3416	7585
290	260	7705	3427	7740	3416	7585
289	259	7704	3426	7739	3415	7584
289	259	7703	3426	7738	3415	7583
289	259	7705	3426	7740	3415	7585

289	259	7703	3426	7738	3413	7583
289	259	7705	3426	7740	3415	7585
287	257	7703	3424	7738	3413	7583
290	260	7706	3427	7741	3416	7586
287	257	7703	3424	7738	3413	7583
286	256	7702	3423	7737	3412	7582
287	257	7703	3424	7738	3413	7583
288	258	7704	3425	7739	3414	7584
288	258	7704	3425	7739	3414	7584
10	82	7803	3290	7826	3281	7722
14	86	7807	3294	7830	3285	7726
	86	7807	3294	7830	3285	7726
11	89	7810	3297	7833	3288	7729
109	183	7758	3360	7785	3348	7683
15	83	7804	3291	7827	3282	7723
85	21	7795	3303	7816	3280	7716
83	19	7793	3301	7814	3278	7714
87	23	7798	3303	7819	3282	7719
78	18	7793	3298	7814	3277	7714
86		7795	3302	7816	3279	7716
94	20	7801	3302	7821	3279	7720
266	236	7682	3382	7662	3371	7573
787	774	7705	3443	7738	3474	7629
854	845	7709	3503	7738	3498	7633
1914	1881	7847	3465	7900	3383	7766
1914	1881	7847	3465	7900	3383	7766
1921	1888	7852	3472	7905	3391	7771
1932	1904	7885	3474	7933	3399	7801
2030	2014	7859	3444	7890	3308	7789
3277	3277	7710	2340	7722	632	7627
3280	3280	7713	2343	7725	635	7630
3288	3284	7720	2345	7730	625	7639
3313	3307	7725	2383	7727	803	7634
3236	3232	7687	2305	7703	642	7611
3244	3240	7695	2313	7711	650	7619
3235	3231	7686	2304	7702	641	7610
3285	3279	7748	2305	7774		7687
3287	3281	7750	2307	7776	18	7689
3292	3286	7754	2312	7780	23	7693
3304	3298	7767	2324	7793	35	7706
3293	3301	7712	3	7679	2304	7639
3294	3302	7713	4	7680	2305	7640
3294	3302	7713		7680	2305	7640
3295	3303	7714	7	7681	2306	7641
3293	3301	7712	5	7679	2304	7639
3293	3301	7714	40	7681	2303	7641
3371	3377	7727	2626	7716	2580	7662
3370	3376	7726	2625	7715	2579	7661
3369	3375	7725	2624	7714	2578	7660
3368	3374	7724	2623	7713	2577	7659
3370	3376	7726	2625	7715	2579	7661
3369	3375	7725	2624	7714	2578	7660
3400	3407	7708	2653	7691	2610	7637
3372	3378	7728	2627	7717	2581	7663
3407	3387	7812	2558	7814	2601	7733

3295	3289	7789	2515	7795	2448	7705
3298	3292	7792	2518	7798	2451	7708
3296	3290	7790	2516	7796	2449	7706
3293	3287	7787	2516	7793	2448	7703
25555	25535	24978	25498	25029	25549	24972
25554	25534	24977	25497	25028	25548	24971
31182	31163	30968	31226	31016	31203	30944

ERS1801995	ERS1812824	20151201	MT66C.C1	DTU2017-812-PR	DTU2017-821-PRJ1111	PA20B
3748	3765	3787	3591	3787	7617	6649
3691	3683	3702	3550	3710	7566	6606
3691	3683	3702	3550	3710	7566	6610
3733	3736	3768	3569	3776	7609	6616
3712	3721	3755	3568	3763	7619	6615
3746	3750	3787	3591	3795	7586	6617
3746	3750	3787	3591	3795	7586	6617
3748	3736	3761	3608	3769	7594	6643
3750	3738	3763	3611	3771	7598	6645
3789	3773	3816	3633	3824	7566	6625
3793	3779	3804	3655	3812	7559	6629
3778	3766	3792	3628	3797	7598	6654
3867	3878	3896	3718	3902	7663	6661
3867	3878	3896	3718	3902	7663	6661
3797	3806	3826	3649	3832	7615	6637
3794	3805	3823	3647	3829	7619	6649
3671	3680	3704	3523	3710	7581	6600
3724	3729	3751	3570	3757	7597	6589
3777	3800	3769	3632	3768	7590	6631
3784	3810	3780	3646	3771	7579	6660
3816	3829	3831	3675	3837	7612	6663
3675	3686	3698	3526	3707	7597	6596
3773	3793	3804	3629	3812	7607	6600
3803	3802	3836	3660	3844	7622	6658
3678	3701	3717	3539	3717	7568	6599
3708	3719	3759	3611	3759	7623	6631
3639	3661	3698	3602	3688	7638	6648
3808	3818	3815	3699	3823	7634	6631
3752	3762	3797	3671	3787	7566	6626
3705	3721	3749	3580	3750	7580	6601
3705	3721	3749	3580	3750	7580	6601
3707	3723	3751	3583	3752	7583	6602
3701	3712	3709	3568	3718	7607	6648
3645	3657	3686	3592	3676	7629	6642
3645	3657	3686	3592	3676	7629	6642
1848	1887	1911	1937	1930	7728	6610
1855	1912	1930	1928	1949	7726	6600
1831	1901	1916	1920	1935	7727	6605
1852	1910	1923	1937	1942	7725	6588
1827	1869	1898	1901	1917	7711	6596
1853	1911	1914	1912	1943	7722	6592
1852	1910	1913	1911	1942	7723	6591
1826	1884	1892	1903	1922	7722	6613
1855	1917	1936	1948	1951	7725	6626
1897	1955	1962	1952	1987	7698	6579
1981	2034	2036	2044	2063	7678	6601
1925	1990	2001	1947	2000	7709	6722
1831	1894	1818	1821	1841	7648	6593
1826	1889	1813	1816	1836	7647	6588
1832	1895	1819	1822	1842	7653	6594
1826	1889	1813	1816	1836	7647	6588
1831	1894	1818	1821	1841	7652	6593
1850	1929	1821	1799	1841	7709	6660

1878	1941	1929	1887	1964	7761	6764
1734	1801	1799	1797	1812	7704	6656
1733	1800	1798	1796	1811	7702	6655
1726	1793	1791	1791	1804	7699	6647
1728	1795	1793	1793	1806	7701	6650
1700	1767	1747	1745	1758	7690	6685
1709	1776	1756	1754	1767	7699	6694
1709	1776	1756	1754	1767	7699	6694
1673	1740	1718	1742	1731	7673	6660
1698	1765	1743	1763	1756	7685	6675
1690	1757	1746	1766	1758	7658	6650
1665	1732	1710	1734	1723	7665	6652
1684	1751	1729	1813	1752	7698	6662
1684	1751	1729	1813	1752	7698	6662
1673	1740	1718	1802	1741	7688	6651
1639	1706	1710	1795	1699	7681	6648
1736	1796	1850	1836	1852	7679	6714
1767	1827	1881	1867	1883	7708	6742
1738	1798	1852	1838	1854	7681	6716
1736	1796	1850	1836	1852	7679	6714
2197	2267	2228	2084	2235	7722	6763
2199	2269	2230	2086	2237	7724	6765
2193	2263	2224	2080	2231	7718	6759
2190	2260	2221	2077	2228	7715	6756
2172	2251	2203	2087	2210	7726	6767
2188	2258	2219	2075	2226	7717	6760
2192	2262	2223	2079	2230	7717	6758
2201	2271	2232	2094	2239	7722	6769
2192	2262	2223	2079	2230	7717	6758
2191	2261	2222	2078	2229	7716	6757
2188	2258	2233	2089	2240	7719	6756
2193	2253	2216	2148	2223	7726	6743
2198	2268	2229	2087	2236	7709	6762
2198	2268	2229	2087	2236	7709	6762
2198	2268	2229	2087	2236	7709	6762
2148	2208	2197	2121	2197	7779	6736
2055	2127	2130	2031	2130	7776	6736
1831	1893	1838	2054	1851	7695	6610
1813	1887	1834	2040	1847	7696	6614
1813	1887	1834	2040	1847	7696	6614
1805	1879	1826	2044	1839	7694	6610
1902	1961	1987	1962	1998	7695	6727
2156	2228	2186	1959	2191	7759	6737
2284	2356	2304	2079	2309	7796	6722
2347	2425	2337	2108	2342	7803	6712
2347	2425	2337	2108	2342	7803	6712
2352	2415	2350	2129	2355	7807	6723
2318	2380	2316	2109	2321	7809	6722
2292	2372	2300	2124	2305	7775	6705
2295	2373	2307	2177	2312	7772	6696
2266	2338	2346	2225	2359	7851	6775
2263	2335	2327	2260	2340	7850	6786
1825	1875	1926	561	1942	7684	6664
1836	1885	1941	576	1957	7705	6684
1768	1821	1886	478	1883	7698	6676

1772	1825	1890	480	1887	7700	6679
1794	1847	1898	442	1895	7703	6677
1845	1898	1925	468	1938	7674	6699
1895	1956	1977	327	1974	7700	6704
1895	1956	1977	327	1974	7702	6706
1873	1952	1947	19	1968	7700	6728
1875	1954	1949	21	1970	7702	6730
1875	1954	1949	21	1970	7700	6730
1905	1964	1941	40	1962	7686	6735
1886	1955	1946		1967	7698	6723
1914	1983	1952	110	1973	7686	6739
1915	1984	1953	111	1974	7687	6740
1729	1791	1837	638	1812	7713	6691
1727	1797	1841	628	1816	7714	6689
1883	1952	1923	1923	1942	7715	6668
1885	1954	1925	1923	1944	7717	6668
1875	1944	1915	1915	1934	7707	6660
1874	1943	1914	1914	1933	7706	6659
1888	1957	1934	1942	1953	7715	6644
1874	1943	1914	1916	1933	7706	6659
1875	1944	1915	1915	1934	7707	6660
1889	1958	1929	1929	1948	7719	6674
1918	1975	1928	1953	1947	7696	6673
1895	1964	1954	1964	1954	7688	6709
553	646	483	2138	445	7830	6691
573	666	489	2146	465	7835	6698
569	664	497	2156	459	7845	6708
546	641	474	2101	436	7819	6692
327	410	241	1988	203	7816	6749
314	387	227	1987	189	7810	6751
314	387	227	1987	189	7810	6751
312	385	225	1985	187	7807	6748
316	393	226	1995	188	7810	6757
301	382	197	1953	159	7784	6755
280	365	198	1961	160	7815	6755
406	505	342	2056	304	7779	6807
327	410	253	1992	215	7789	6744
224	307	142	1937	104	7812	6740
288	367		1946	86	7813	6775
245	324	69	1962	43	7817	6767
250	329	86	1967		7821	6773
285	356	125	1948	99	7796	6751
261	344	167	1968	129	7816	6754
256	335	160	1959	122	7805	6745
33	140	285	1874	247	7774	6728
	141	288	1886	250	7777	6733
308	389	526	2051	490	7754	6666
317	398	535	2060	499	7763	6675
301	382	519	2044	483	7747	6659
265	346	479	1901	443	7773	6716
64	135	299	1888	261	7770	6724
64	137	299	1889	261	7773	6725
496	569	659	2112	658	7688	6860
254	153	450	1993	430	7775	6722
251	130	441	1994	421	7765	6715

269	154	459	2004	439	7774	6720
264	149	454	2007	434	7781	6731
250	145	437	2009	411	7755	6664
150	43	376	1967	338	7798	6751
143	18	369	1957	331	7788	6744
141		367	1955	329	7786	6742
69	136	301	1892	263	7769	6728
53	140	273	1890	235	7749	6727
89	162	329	1911	291	7794	6748
80	153	320	1904	282	7787	6741
279	370	503	1801	465	7757	6700
279	370	503	1801	465	7757	6700
115	140	338	1925	300	7772	6731
110	135	333	1920	295	7767	6726
113	138	336	1923	298	7770	6729
1841	1899	1980	1845	1975	7661	6627
1842	1900	1981	1844	1976	7658	6626
1829	1887	1968	1833	1963	7648	6615
1976	2035	2113	2046	2108	7744	6718
1793	1855	1956	1877	1951	7673	6641
2103	2167	2222	2032	2217	7897	6879
3921	3899	3895	3797	3902	7870	6930
6731	6740	6773	6721	6771	7955	6
6733	6742	6775	6723	6773	7957	
6730	6739	6772	6720	6770	7954	5
6732	6741	6774	6722	6772	7956	7
6797	6808	6825	6790	6823	7922	224
6785	6798	6827	6777	6825	7940	213
6793	6808	6841	6785	6839	7951	217
6805	6814	6847	6809	6845	7929	227
6819	6828	6861	6823	6859	7944	243
6782	6793	6824	6806	6822	7933	283
6845	6854	6887	6835	6885	7943	274
6797	6806	6839	6801	6837	7922	221
6827	6836	6869	6831	6867	7951	251
6798	6807	6840	6788	6838	7913	224
6795	6804	6837	6805	6835	7922	225
6803	6812	6845	6807	6843	7926	227
6975	6984	7015	6940	7015	8049	571
6970	6979	7010	6935	7010	8044	566
6929	6938	6959	6899	6969	8032	553
6838	6847	6876	6826	6878	7966	424
6943	6952	6981	6930	6981	8053	576
6819	6828	6848	6805	6859	7935	392
6813	6822	6842	6799	6853	7929	386
6812	6821	6841	6798	6852	7930	385
6781	6790	6810	6770	6821	7901	493
6831	6844	6844	6816	6855	7808	664
3655	3694	3627	3286	3652	7879	6898
3644	3683	3616	3275	3641	7868	6888
7683	7692	7706	7553	7729	290	7908
7683	7692	7706	7553	7729	290	7908
7682	7691	7705	7552	7728	289	7907
7681	7690	7704	7551	7727	289	7906
7683	7692	7706	7553	7729	289	7908

7681	7690	7704	7551	7727	289	7908
7683	7692	7706	7553	7729	289	7908
7681	7690	7704	7551	7727	287	7906
7684	7693	7707	7554	7730	290	7909
7681	7690	7704	7551	7727	287	7906
7680	7689	7703	7550	7726	286	7905
7681	7690	7704	7551	7727	287	7906
7682	7691	7705	7552	7728	288	7907
7682	7691	7705	7552	7728	288	7907
7773	7782	7809	7694	7817	8	7953
7777	7786	7813	7698	7821		7957
7777	7786	7813	7698	7821	14	7957
7780	7789	7816	7701	7824	17	7960
7734	7743	7770	7649	7778	111	7953
7774	7783	7810	7695	7818	15	7954
7763	7772	7799	7686	7807	85	7943
7761	7770	7797	7684	7805	83	7942
7766	7775	7802	7689	7810	87	7946
7761	7770	7797	7684	7805	78	7941
7763	7772	7799	7686	7807	86	7943
7768	7777	7804	7690	7812	94	7946
7599	7608	7645	7569	7653	266	7898
7681	7692	7722	7602	7726	787	7887
7681	7692	7722	7612	7726	854	7889
7832	7843	7872	7746	7876	1914	7968
7832	7843	7872	7746	7876	1914	7968
7837	7848	7877	7751	7881	1921	7975
7869	7876	7914	7789	7914	1932	8015
7843	7844	7884	7769	7884	2030	7949
7679	7667	7712	7620	7716	3277	7744
7682	7670	7715	7623	7719	3280	7747
7687	7675	7720	7630	7724	3288	7752
7692	7680	7725	7622	7729	3313	7729
7658	7648	7693	7594	7697	3236	7772
7666	7656	7701	7602	7705	3244	7780
7657	7647	7692	7593	7696	3235	7771
7731	7721	7766	7670	7770	3285	7778
7733	7723	7768	7672	7772	3287	7780
7737	7727	7772	7676	7776	3292	7785
7750	7740	7785	7689	7789	3304	7797
7650	7640	7690	7603	7694	3293	7767
7651	7641	7691	7604	7695	3294	7768
7651	7641	7691	7604	7695	3294	7768
7652	7642	7692	7605	7696	3295	7769
7650	7640	7690	7603	7694	3293	7767
7652	7642	7692	7605	7696	3293	7770
7694	7681	7743	7612	7745	3371	7788
7693	7680	7742	7611	7744	3370	7787
7692	7679	7741	7610	7743	3369	7786
7691	7678	7740	7609	7742	3368	7785
7693	7680	7742	7611	7744	3370	7787
7692	7679	7741	7610	7743	3369	7786
7669	7656	7718	7587	7720	3400	7769
7695	7682	7744	7613	7746	3372	7789
7772	7760	7807	7700	7809	3407	7819

7742	7727	7794	7659	7798	3295	7983
7745	7730	7797	7662	7801	3298	7988
7743	7728	7795	7660	7799	3296	7986
7740	7725	7792	7657	7796	3293	7981
25042	25037	25059	24979	25061	25553	25061
25041	25036	25058	24978	25060	25552	25060
31010	30996	31016	31002	31018	31182	30821

1351	2011-70-34-3	2011-70-41-2	DTU2011_26	2011-70-219-2	HVH17	ESC0167	ESC0211
6806	3736	859	3525	729	7605	1040	6974
6765	3684	254	3462	859	7554	493	6967
6769	3684	256	3460	861	7554	495	6971
6775	3715		3500	938	7597	499	6967
6774	3720	314	3460	889	7607	496	6976
6776	3747	320	3486	890	7574	507	6978
6776	3747	320	3486	890	7574	507	6978
6802	3742	320	3501	871	7582	501	7004
6804	3744	322	3504	873	7586	503	7006
6784	3777	394	3535	971	7554	484	6964
6788	3785	533	3565	853	7547	357	6968
6813	3774	374	3516	886	7586	499	6990
6820	3843	600	3649	986	7651	452	7004
6820	3843	600	3649	986	7651	452	7004
6796	3781	462	3575	947	7603	419	6980
6808	3770	452	3572	947	7607	413	6992
6759	3651	378	3457	852	7569	536	6929
6748	3702	441	3510	931	7585	582	6940
6790	3775	499	3559	901	7578	633	6925
6811	3794	676	3552	1092	7567	803	6972
6822	3798	526	3591	973	7600	655	6983
6755	3651	702	3446	957	7585	625	6936
6759	3753	497	3547	859	7595	376	6937
6817	3780	499	3574	806	7610		6997
6758	3666	469	3473	722	7556	395	6940
6790	3708	631	3547	800	7611	526	6978
6805	3631	1186	3499	564	7624	1084	6973
6790	3782	1281	3636	812	7622	1179	6990
6785	3736	908	3595	548	7554	857	6953
6760	3699	862	3521	310	7568	764	6953
6760	3699	862	3521	310	7568	764	6953
6761	3701	865	3523	313	7571	767	6954
6801	3692	938	3530		7593	806	6967
6799	3627	1184	3493	548	7615	1082	6967
6799	3627	1184	3493	548	7615	1082	6967
6688	1844	3536	1825	3516	7715	3628	6854
6678	1847	3522	1816	3507	7713	3613	6844
6683	1839	3532	1818	3516	7714	3622	6849
6666	1852	3496	1850	3441	7712	3561	6832
6674	1826	3513	1804	3479	7698	3617	6840
6670	1853	3530	1822	3498	7709	3618	6845
6669	1852	3529	1821	3497	7710	3617	6844
6691	1826	3509	1784	3482	7709	3589	6841
6704	1853	3531	1818	3455	7712	3595	6846
6657	1897	3527	1832	3464	7685	3585	6812
6679	1978	3523	1905	3431	7664	3569	6830
6798	1933	3598	1775	3571	7694	3641	6927
6685	1846	3400	1909	3402	7634	3527	6827
6680	1841	3395	1904	3397	7633	3522	6822
6686	1847	3401	1910	3403	7639	3528	6828
6680	1841	3395	1904	3397	7633	3522	6822
6685	1846	3400	1909	3402	7638	3527	6827
6718	1869	3562	1919	3535	7693	3638	6861

6838	1893	3617	2058	3622	7745	3719	7008
6740	1751	3538	1933	3541	7690	3614	6880
6739	1750	3537	1932	3540	7688	3613	6879
6731	1743	3532	1925	3535	7685	3608	6869
6734	1745	3534	1927	3537	7687	3610	6874
6761	1717	3591	1911	3602	7676	3667	6887
6770	1726	3600	1920	3611	7685	3676	6896
6770	1726	3600	1920	3611	7685	3676	6896
6736	1690	3565	1882	3566	7659	3639	6876
6751	1715	3588	1917	3589	7671	3662	6891
6726	1707	3551	1919	3527	7644	3623	6875
6728	1682	3557	1874	3558	7651	3631	6868
6738	1701	3570	1942	3577	7684	3646	6876
6738	1701	3570	1942	3577	7684	3646	6876
6727	1690	3559	1931	3566	7674	3635	6865
6724	1656	3566	1914	3571	7667	3640	6859
6798	1747	3560	1806	3556	7664	3642	6939
6826	1778	3591	1837	3587	7693	3673	6967
6800	1749	3562	1808	3558	7666	3644	6941
6798	1747	3560	1806	3556	7664	3642	6939
6835	2190	3711	2199	3713	7710	3755	6966
6837	2192	3714	2201	3716	7712	3758	6968
6831	2186	3708	2195	3710	7706	3752	6962
6828	2183	3705	2192	3707	7703	3749	6959
6839	2185	3719	2194	3714	7714	3766	6970
6832	2181	3703	2190	3707	7705	3749	6963
6830	2185	3707	2194	3709	7705	3751	6961
6841	2194	3714	2201	3716	7710	3758	6972
6830	2185	3707	2194	3709	7705	3751	6961
6829	2184	3706	2193	3708	7704	3750	6960
6828	2181	3685	2142	3681	7707	3751	6961
6815	2184	3712	2239	3704	7714	3756	6946
6834	2191	3645	2229	3709	7697	3751	6965
6834	2191	3645	2229	3709	7697	3751	6965
6834	2191	3645	2229	3709	7697	3751	6965
6806	2164	3747	2121	3682	7765	3784	6950
6806	2075	3747	2021	3668	7762	3770	6950
6676	1825	3563	1902	3607	7681	3644	6834
6680	1809	3549	1888	3603	7682	3630	6838
6680	1809	3549	1888	3603	7682	3630	6838
6676	1817	3558	1875	3604	7680	3639	6834
6801	1893	3516	1905	3549	7681	3632	6955
6805	2145	3631	2181	3580	7745	3671	6955
6790	2273	3679	2280	3630	7782	3721	6940
6796	2336	3672	2323	3625	7789	3714	6946
6796	2336	3672	2323	3625	7789	3714	6946
6807	2363	3657	2330	3613	7793	3716	6957
6804	2309	3642	2290	3603	7795	3698	6954
6795	2291	3689	2298	3640	7761	3737	6945
6770	2284	3763	2383	3719	7756	3807	6908
6873	2255	3707	2373	3706	7837	3739	7006
6884	2250	3707	2385	3696	7836	3739	7017
6748	1817	3550	1850	3530	7670	3621	6895
6768	1831	3563	1865	3533	7691	3626	6915
6760	1776	3559	1776	3548	7684	3644	6886

6763	1780	3559	1780	3548	7686	3644	6891
6761	1802	3559	1789	3532	7689	3644	6889
6783	1843	3547	1868	3541	7660	3628	6915
6786	1888	3565	1830	3524	7686	3641	6914
6788	1888	3565	1830	3524	7688	3641	6916
6810	1889	3574	1849	3575	7686	3659	6940
6812	1891	3576	1851	3577	7688	3661	6942
6812	1891	3576	1851	3577	7686	3661	6942
6817	1907	3589	1862	3590	7672	3675	6947
6805	1892	3569	1844	3568	7684	3660	6935
6821	1918	3547	1870	3550	7672	3638	6949
6822	1919	3548	1871	3551	7673	3639	6950
6771	1727	3598	1772	3575	7700	3663	6911
6769	1739	3592	1762	3571	7701	3669	6909
6738	1900	3549	1880	3572	7701	3626	6909
6738	1902	3549	1882	3572	7703	3626	6909
6730	1892	3541	1872	3564	7693	3618	6901
6729	1891	3540	1871	3563	7692	3617	6900
6714	1905	3546	1885	3573	7701	3629	6909
6729	1891	3540	1871	3563	7692	3617	6900
6730	1892	3541	1872	3564	7693	3618	6901
6744	1906	3555	1886	3578	7705	3632	6915
6743	1929	3543	1910	3556	7682	3619	6914
6779	1912	3568	1913	3592	7674	3647	6891
6751	568	3790	2104	3746	7814	3860	6929
6758	588	3792	2119	3745	7819	3862	6936
6768	584	3806	2122	3758	7829	3872	6946
6752	561	3815	2077	3767	7803	3881	6930
6809	340	3721	1957	3649	7800	3789	6984
6811	313	3719	1956	3666	7794	3782	6984
6811	313	3719	1956	3666	7794	3782	6984
6808	311	3717	1954	3664	7791	3780	6981
6817	323	3729	1970	3672	7794	3790	6992
6815	316	3754	1932	3698	7768	3823	6990
6815	299	3744	1934	3692	7799	3818	6990
6805	433	3800	2024	3757	7770	3869	6943
6806	344	3750	1981	3627	7773	3790	6979
6802	241	3750	1944	3688	7796	3820	6977
6837	301	3768	1981	3709	7797	3836	7010
6829	258	3771	1971	3713	7801	3839	7004
6835	263	3776	1976	3718	7805	3844	7010
6813	296	3773	1979	3697	7780	3817	6992
6816	268	3726	1975	3673	7800	3785	6991
6807	263	3721	1970	3658	7789	3782	6982
6790	64	3725	1832	3693	7758	3801	6965
6795	69	3733	1842	3701	7761	3803	6970
6728	319	3832	1979	3783	7738	3839	6884
6737	328	3841	1988	3792	7747	3848	6893
6721	312	3825	1972	3776	7731	3832	6877
6778	276	3771	1883	3740	7757	3834	6942
6786	75	3737	1842	3703	7754	3801	6961
6787	75	3742	1843	3708	7757	3806	6961
6754	503	3791	2080	3698	7632	3859	6876
6784	247	3727	1944	3704	7759	3784	6981
6777	246	3726	1947	3703	7749	3781	6974

6782	265	3745	1957	3727	7758	3787	6979
6793	260	3740	1960	3722	7765	3798	6990
6726	239	3781	1998	3740	7739	3811	6931
6813	148	3748	1909	3724	7782	3814	6988
6806	138	3738	1902	3714	7772	3804	6981
6804	136	3736	1900	3712	7770	3802	6979
6790		3715	1852	3692	7753	3780	6965
6789	66	3733	1842	3703	7733	3802	6964
6810	88	3747	1864	3702	7778	3806	6985
6803	79	3740	1857	3695	7771	3799	6978
6770	292	3732	1806	3702	7743	3798	6924
6770	292	3732	1806	3702	7743	3798	6924
6793	107	3739	1878	3694	7756	3800	6970
6788	102	3734	1873	3689	7751	3795	6965
6791	105	3737	1876	3690	7754	3798	6968
6689	1851	3498	45	3529	7649	3572	6843
6688	1852	3500		3530	7646	3574	6842
6677	1839	3488	33	3517	7636	3562	6831
6792	1970	3670	582	3690	7732	3749	6926
6715	1802	3532	391	3545	7661	3606	6849
6953	2108	3743	824	3770	7885	3827	7087
7026	3904	3376	3705	3410	7859	3515	7171
223	6726	6614	6624	6646	7940	6656	564
225	6728	6616	6626	6648	7942	6658	566
222	6725	6613	6623	6645	7939	6655	563
224	6727	6615	6625	6647	7941	6657	565
105	6792	6754	6677	6786	7911	6795	437
94	6780	6747	6670	6773	7929	6785	426
98	6790	6753	6670	6783	7940	6795	430
34	6800	6789	6702	6815	7919	6831	368
50	6814	6803	6716	6829	7934	6845	384
90	6774	6761	6699	6794	7923	6815	424
90	6840	6845	6744	6871	7930	6887	373
16	6792	6769	6686	6795	7912	6811	386
46	6822	6798	6716	6825	7941	6840	416
33	6793	6760	6669	6782	7905	6802	387
	6790	6775	6688	6801	7912	6817	386
22	6798	6775	6692	6801	7916	6817	392
391	6970	6972	6847	6972	8036	7002	23
386	6965	6967	6842	6967	8031	6997	
381	6924	6933	6816	6926	8020	6951	137
223	6831	6782	6693	6794	7958	6828	419
371	6936	6908	6821	6916	8043	6839	506
187	6814	6755	6696	6781	7925	6813	429
181	6808	6749	6690	6775	7919	6807	423
186	6807	6754	6689	6778	7920	6812	426
288	6776	6735	6663	6761	7889	6799	530
459	6828	6785	6733	6811	7798	6842	697
6916	3664	4186	3475	4155	7868	4234	7058
6906	3653	4175	3464	4144	7857	4223	7048
7876	7675	7517	7515	7498	261	7523	8006
7876	7675	7517	7515	7498	261	7523	8006
7875	7674	7516	7514	7497	260	7522	8005
7874	7673	7515	7513	7496	260	7521	8004
7876	7675	7517	7515	7498	260	7523	8006

7876	7673	7515	7513	7496	260	7521	8006
7876	7675	7517	7515	7498	260	7523	8006
7874	7673	7515	7513	7496	258	7521	8004
7877	7676	7518	7516	7499	261	7524	8007
7874	7673	7515	7513	7496	258	7521	8004
7873	7672	7514	7512	7495	257	7520	8003
7874	7673	7515	7513	7496	258	7521	8004
7875	7674	7516	7514	7497	259	7522	8005
7875	7674	7516	7514	7497	259	7522	8005
7918	7765	7605	7656	7603	79	7618	8040
7922	7769	7609	7658	7607	83	7622	8044
7922	7769	7609	7660	7607	83	7622	8044
7925	7772	7612	7663	7610	86	7625	8047
7924	7726	7552	7615	7538	180	7559	8046
7919	7766	7606	7657	7604	80	7619	8041
7913	7755	7599	7648	7595	8	7612	8032
7912	7753	7597	7646	7593		7610	8031
7916	7758	7602	7651	7598	18	7615	8035
7911	7753	7597	7646	7593	15	7610	8030
7913	7755	7598	7648	7594	19	7611	8032
7916	7760	7604	7653	7600	27	7617	8035
7866	7591	7522	7550	7497	237	7567	7989
7833	7673	7502	7559	7511	773	7446	7993
7829	7673	7500	7555	7511	844	7444	7987
7942	7822	7674	7694	7690	1880	7694	8057
7942	7822	7674	7694	7690	1880	7694	8057
7949	7827	7681	7699	7697	1887	7703	8064
7989	7859	7731	7728	7759	1903	7753	8095
7925	7835	7663	7707	7686	2013	7690	8045
7690	7672	7542	7543	7569	3276	7565	7799
7693	7675	7545	7546	7572	3279	7568	7802
7701	7680	7552	7553	7579	3283	7575	7810
7678	7685	7529	7550	7564	3306	7550	7799
7721	7651	7523	7520	7556	3231	7548	7830
7729	7659	7531	7528	7564	3239	7556	7838
7720	7650	7522	7519	7555	3230	7547	7829
7727	7724	7596	7594	7619	3278	7611	7851
7729	7726	7598	7596	7621	3280	7613	7853
7734	7730	7602	7600	7624	3285	7617	7858
7746	7743	7615	7613	7638	3297	7630	7870
7726	7643	7569	7521	7579	3300	7590	7854
7727	7644	7568	7522	7580	3301	7589	7855
7727	7644	7570	7522	7580	3301	7591	7855
7728	7645	7571	7523	7581	3302	7592	7856
7726	7643	7569	7521	7579	3300	7590	7854
7728	7645	7571	7523	7581	3300	7592	7856
7763	7686	7533	7560	7568	3376	7553	7877
7762	7685	7532	7559	7567	3375	7552	7876
7761	7684	7531	7558	7566	3374	7551	7875
7760	7683	7530	7557	7565	3373	7550	7874
7762	7685	7532	7559	7567	3375	7552	7876
7761	7684	7531	7558	7566	3374	7551	7875
7788	7661	7514	7535	7549	3406	7534	7902
7764	7687	7534	7561	7569	3377	7554	7878
7788	7764	7599	7637	7647	3384	7636	7892

7946	7734	7665	7626	7693	3288	7682	8062
7951	7737	7668	7629	7696	3291	7685	8067
7949	7735	7666	7627	7694	3289	7683	8065
7944	7732	7663	7624	7691	3286	7680	8060
25017	25033	24918	24941	24932	25532	24931	25052
25016	25032	24917	24940	24931	25531	24930	25051
30797	31007	30939	30914	30928	31161	30954	30788

ESC0198	AZ-TG60445	AZ-TG60412	2-316-03_S3_C3	7-233-03_S3_	2-316-03_S1_C2
3759	7612	3782	3720	904	6938
3697	7561	3705	3696	351	6931
3697	7561	3705	3696	353	6935
3732	7604	3771	3750	394	6933
3701	7614	3758	3724	383	6940
3741	7581	3790	3760	328	6942
3741	7581	3790	3760	328	6942
3754	7589	3764	3755	300	6968
3756	7593	3766	3757	302	6970
3798	7561	3819	3760		6918
3801	7554	3807	3759	191	6922
3774	7593	3792	3741	304	6948
3868	7658	3897	3864	618	6962
3868	7658	3897	3864	618	6962
3798	7610	3827	3794	470	6938
3795	7614	3824	3791	470	6950
3672	7576	3705	3707	410	6877
3725	7592	3752	3758	461	6898
3775	7585	3763	3710	485	6919
3780	7574	3766	3774	629	6932
3822	7605	3832	3759	546	6950
3659	7592	3702	3650	758	6892
3766	7602	3807	3765	551	6891
3798	7617	3839	3790	484	6951
3677	7563	3712	3658	567	6908
3713	7618	3754	3708	649	6945
3659	7631	3683	3605	1233	6943
3793	7629	3818	3783	1328	6936
3773	7561	3782	3751	999	6913
3702	7575	3745	3667	912	6919
3702	7575	3745	3667	912	6919
3704	7578	3747	3669	915	6920
3702	7600	3713	3627	971	6926
3653	7622	3671	3595	1231	6937
3653	7622	3671	3595	1231	6937
1814	7721	1925	1960	3576	6820
1801	7719	1944	1969	3585	6810
1789	7720	1930	1961	3592	6815
1798	7718	1937	1960	3558	6798
1783	7704	1912	1935	3556	6806
1799	7715	1938	1955	3586	6798
1798	7716	1937	1954	3585	6797
1772	7715	1917	1962	3570	6802
1809	7718	1946	1925	3575	6805
1843	7691	1982	1923	3556	6761
1927	7670	2058	1957	3535	6779
1856	7700	1995	2017	3623	6916
1830	7640	1836	1904	3443	6794
1825	7639	1831	1899	3438	6789
1831	7645	1837	1905	3444	6795
1825	7639	1831	1899	3438	6789
1830	7644	1836	1904	3443	6794
1803	7701	1836	1886	3617	6832

1887	7751	1959	1975	3674	6967
1675	7696	1807	1889	3588	6849
1674	7694	1806	1888	3587	6848
1667	7691	1799	1881	3582	6840
1669	7693	1801	1883	3584	6843
1645	7682	1753	1834	3645	6857
1654	7691	1762	1843	3654	6866
1654	7691	1762	1843	3654	6866
1618	7665	1726	1808	3617	6845
1643	7677	1751	1833	3640	6860
1635	7650	1753	1793	3597	6834
1610	7657	1718	1800	3609	6837
1627	7690	1747	1805	3623	6845
1627	7690	1747	1805	3623	6845
1616	7680	1736	1794	3612	6834
1586	7673	1694	1776	3618	6832
1719	7672	1847	1921	3613	6914
1750	7701	1878	1952	3644	6942
1721	7674	1849	1923	3615	6916
1719	7672	1847	1921	3613	6914
2108	7716	2230	2258	3757	6937
2110	7718	2232	2260	3760	6939
2104	7712	2226	2254	3754	6933
2101	7709	2223	2251	3751	6930
2087	7720	2205	2233	3758	6941
2099	7711	2221	2249	3751	6934
2103	7711	2225	2253	3753	6932
2112	7716	2234	2262	3760	6943
2103	7711	2225	2253	3753	6932
2102	7710	2224	2252	3752	6931
2099	7713	2235	2245	3749	6930
2110	7720	2218	2236	3748	6917
2109	7703	2231	2261	3691	6936
2109	7703	2231	2261	3691	6936
2109	7703	2231	2261	3691	6936
2091	7771	2192	2198	3772	6915
1998	7768	2125	2125	3784	6915
1791	7687	1846	1882	3590	6801
1769	7688	1842	1888	3584	6805
1769	7688	1842	1888	3584	6805
1763	7686	1834	1880	3587	6801
1895	7687	1993	2023	3587	6924
2107	7753	2186	2218	3669	6926
2229	7790	2304	2336	3721	6911
2278	7797	2337	2367	3720	6917
2278	7797	2337	2367	3720	6917
2269	7801	2350	2368	3708	6928
2255	7803	2316	2340	3696	6925
2206	7769	2300	2328	3741	6916
2214	7762	2307	2337	3803	6877
2208	7843	2354	2382	3747	6969
2203	7842	2335	2363	3747	6980
1725	7677	1937	1910	3587	6851
1740	7698	1952	1933	3585	6871
1664	7691	1878	1912	3611	6858

1668	7693	1882	1914	3611	6863
1688	7696	1890	1922	3611	6861
1748	7667	1933	1979	3594	6879
1812	7693	1969	2003	3606	6886
1812	7695	1969	2003	3606	6888
1804	7692	1963	1995	3630	6904
1806	7694	1965	1997	3632	6906
1806	7692	1965	1997	3632	6906
1831	7678	1957	2006	3642	6911
1801	7690	1962	1992	3633	6899
1815	7678	1968	1998	3609	6913
1816	7679	1969	1999	3610	6914
1616	7707	1807	1873	3634	6882
1606	7708	1811	1869	3640	6880
1804	7709	1937	1944	3594	6872
1806	7711	1939	1946	3594	6872
1796	7701	1929	1936	3586	6864
1795	7700	1928	1935	3585	6863
1809	7709	1948	1951	3597	6874
1795	7700	1928	1935	3585	6863
1796	7701	1929	1936	3586	6864
1810	7713	1943	1950	3600	6878
1842	7690	1942	1962	3581	6877
1816	7682	1949	1965	3617	6885
692	7821	440	528	3848	6888
712	7826	460	548	3850	6891
710	7836	454	546	3866	6905
687	7810	431	523	3875	6889
498	7807	198	290	3775	6943
491	7801	184	277	3763	6943
491	7801	184	277	3763	6943
489	7798	182	275	3761	6940
497	7801	183	283	3771	6951
481	7775	154	265	3801	6949
457	7806	155	247	3800	6949
570	7777	299	389	3855	6919
538	7780	210		3760	6926
435	7803	99	165	3802	6936
503	7804	69	253	3816	6959
460	7808		210	3819	6963
465	7812	43	215	3824	6969
490	7787	94	212	3801	6933
476	7807	124	232	3774	6950
471	7796	117	217	3765	6941
266	7765	242	320	3789	6924
279	7768	245	327	3789	6929
531	7745	485	444	3788	6848
540	7754	494	453	3797	6857
524	7738	478	437	3781	6841
450	7764	438	490	3812	6916
287	7761	256	335	3789	6920
287	7764	256	335	3792	6920
703	7639	653	572	3840	6836
475	7766	425	428	3782	6924
480	7756	416	425	3761	6917

490	7765	434	443	3785	6922
493	7772	429	438	3780	6933
474	7746	406	469	3776	6884
379	7789	333	419	3796	6947
372	7779	326	412	3775	6940
370	7777	324	410	3773	6938
292	7760	258	344	3777	6924
283	7740	230	328	3788	6923
304	7785	286	364	3785	6944
295	7778	277	355	3778	6937
	7750	460	538	3798	6883
	7750	460	538	3798	6883
342	7763	295	384	3767	6929
337	7758	290	379	3762	6924
340	7761	293	382	3765	6927
1805	7656	1970	1980	3533	6817
1806	7653	1971	1981	3535	6816
1793	7643	1958	1968	3523	6805
1970	7739	2103	2123	3696	6900
1747	7668	1946	1960	3560	6823
2035	7891	2212	2230	3764	7059
3935	7866	3897	3867	3399	7141
6698	7944	6765	6742	6623	551
6700	7946	6767	6744	6625	553
6697	7943	6764	6741	6622	550
6699	7945	6766	6743	6624	552
6767	7915	6817	6813	6764	442
6754	7933	6819	6802	6754	431
6760	7944	6833	6806	6764	435
6780	7923	6839	6816	6798	363
6794	7938	6853	6830	6812	379
6767	7927	6816	6795	6777	419
6810	7934	6879	6856	6854	364
6772	7916	6831	6808	6778	377
6802	7945	6861	6838	6807	407
6769	7909	6832	6809	6769	390
6770	7916	6829	6806	6784	381
6778	7920	6837	6814	6784	383
6929	8040	7009	6984	6969	150
6924	8035	7004	6979	6964	137
6883	8024	6963	6926	6918	
6809	7962	6872	6839	6822	432
6918	8047	6975	6930	6928	574
6794	7929	6853	6825	6789	425
6788	7923	6847	6819	6783	419
6787	7924	6846	6818	6788	426
6764	7893	6815	6787	6769	498
6811	7802	6849	6844	6820	675
3610	7874	3647	3636	4241	7017
3599	7863	3636	3625	4230	7007
7663	270	7725	7681	7450	7969
7663	270	7725	7681	7450	7969
7662	269	7724	7680	7449	7968
7661	269	7723	7679	7448	7967
7663	269	7725	7681	7450	7969

7661	269	7723	7679	7448	7969
7663	269	7725	7681	7450	7969
7661	267	7723	7679	7448	7967
7664	270	7726	7682	7451	7970
7661	267	7723	7679	7448	7967
7660	266	7722	7678	7447	7966
7661	267	7723	7679	7448	7967
7662	268	7724	7680	7449	7968
7662	268	7724	7680	7449	7968
7753	90	7813	7785	7562	8028
7757	94	7817	7789	7566	8032
7757	94	7817	7789	7566	8032
7760	97	7820	7792	7569	8035
7710	191	7774	7746	7508	8034
7754	91	7814	7786	7563	8029
7745	29	7803	7775	7556	8021
7743	27	7801	7773	7554	8020
7748	31	7806	7778	7559	8024
7743	26	7801	7773	7554	8019
7745	20	7803	7775	7555	8021
7750		7808	7780	7561	8024
7579	246	7649	7685	7520	7980
7660	784	7722	7687	7491	7954
7662	855	7722	7687	7489	7947
7811	1891	7871	7851	7641	8043
7811	1891	7871	7851	7641	8043
7816	1898	7876	7856	7648	8050
7848	1914	7909	7889	7693	8085
7826	2018	7882	7857	7646	8029
7662	3277	7714	7681	7513	7783
7665	3280	7717	7684	7516	7786
7670	3284	7722	7689	7523	7794
7677	3307	7727	7692	7498	7779
7641	3232	7695	7662	7486	7814
7649	3240	7703	7670	7494	7822
7640	3231	7694	7661	7485	7813
7711	3279	7768	7735	7558	7833
7713	3281	7770	7737	7560	7835
7717	3286	7774	7741	7564	7840
7730	3298	7787	7754	7577	7852
7633	3301	7692	7662	7535	7832
7634	3302	7693	7663	7534	7833
7634	3302	7693	7663	7536	7833
7635	3303	7694	7664	7537	7834
7633	3301	7692	7662	7535	7832
7635	3301	7694	7664	7536	7834
7673	3377	7743	7703	7501	7869
7672	3376	7742	7702	7500	7868
7671	3375	7741	7701	7499	7867
7670	3374	7740	7700	7498	7866
7672	3376	7742	7702	7500	7868
7671	3375	7741	7701	7499	7867
7648	3407	7718	7678	7482	7894
7674	3378	7744	7704	7502	7870
7748	3385	7807	7781	7584	7879

7731	3291	7796	7754	7626	8046
7734	3294	7799	7757	7629	8051
7732	3292	7797	7755	7627	8049
7729	3289	7794	7752	7624	8044
25029	25538	25056	25037	24943	25043
25028	25537	25055	25036	24942	25042
31008	31166	31010	31001	30930	30780

2-460-02_S1_C2	AZ-TG73171	AZ-TG73251	AZ-TG73331	AZ-TG73319	AZ-TG73315	AZ-TG73343
7531	7620	7571	3720	3588	7701	3748
7468	7569	7520	3670	3562	7631	3688
7468	7569	7522	3670	3562	7633	3688
7515	7612	7552	3721	3598	7674	3747
7521	7622	7568	3706	3583	7682	3710
7488	7589	7547	3738	3605	7647	3734
7488	7589	7547	3738	3605	7647	3734
7496	7597	7569	3735	3617	7659	3743
7499	7601	7573	3737	3620	7665	3746
7448	7569	7523	3775	3634	7641	3772
7441	7562	7531	3778	3661	7634	3768
7480	7601	7572	3757	3633	7671	3758
7580	7666	7617	3851	3717	7736	3848
7580	7666	7617	3851	3717	7736	3848
7532	7618	7568	3781	3646	7686	3785
7536	7622	7573	3778	3644	7690	3782
7479	7584	7537	3663	3532	7648	3657
7512	7600	7553	3722	3577	7674	3694
7491	7593	7556	3758	3616	7664	3769
7481	7582	7548	3763	3614	7653	3785
7512	7615	7573	3803	3683	7685	3795
7501	7600	7547	3649	3558	7661	3732
7498	7610	7546	3759	3637	7685	3771
7521	7625	7575	3789	3663	7694	3784
7488	7571	7528	3666	3536	7641	3685
7533	7626	7590	3706	3610	7693	3705
7550	7641	7593	3627	3556	7717	3667
7536	7637	7588	3742	3684	7730	3762
7478	7569	7535	3738	3644	7647	3755
7496	7583	7538	3677	3572	7669	3687
7496	7583	7538	3677	3572	7669	3687
7499	7586	7541	3679	3575	7672	3691
7496	7610	7579	3649	3575	7690	3682
7541	7632	7585	3603	3548	7708	3663
7541	7632	7585	3603	3548	7708	3663
7615	7731	7635	1943	1847	7757	2272
7613	7729	7634	1956	1860	7752	2265
7614	7730	7634	1948	1846	7756	2275
7612	7728	7641	1947	1869	7758	2255
7598	7714	7618	1922	1836	7742	2267
7605	7725	7636	1948	1854	7751	2271
7606	7726	7635	1947	1853	7752	2270
7601	7725	7632	1927	1847	7755	2273
7601	7728	7628	1964	1866	7754	2280
7573	7701	7587	1992	1910	7730	2283
7532	7681	7583	2070	1987	7708	2325
7580	7712	7626	2001	1883	7755	2235
7568	7651	7574	1880	1775	7703	2105
7567	7650	7571	1875	1770	7700	2100
7573	7656	7577	1881	1776	7706	2106
7567	7650	7571	1875	1770	7700	2100
7572	7655	7576	1880	1775	7705	2105
7603	7712	7630	1828	1675	7770	2067

7617	7764	7680	1961	1854	7815	2226
7558	7707	7622	1823	1698	7722	2080
7558	7705	7621	1822	1697	7720	2079
7553	7702	7614	1815	1692	7715	2072
7555	7704	7616	1817	1694	7717	2074
7562	7693	7618	1769	1676	7707	2046
7571	7702	7627	1778	1685	7716	2055
7571	7702	7627	1778	1685	7716	2055
7527	7676	7600	1740	1627	7693	2025
7539	7688	7609	1765	1648	7706	2042
7514	7661	7582	1765	1652	7680	2032
7519	7668	7592	1732	1619	7685	2017
7558	7701	7621	1751	1725	7722	2063
7558	7701	7621	1751	1725	7722	2063
7548	7691	7613	1740	1714	7712	2052
7567	7684	7616	1708	1673	7706	2086
7540	7682	7606	1843	1623	7733	1932
7569	7711	7635	1874	1654	7760	1963
7542	7684	7608	1845	1625	7735	1934
7540	7682	7606	1843	1623	7733	1932
7579	7725	7637	2227	2088	7762	1683
7581	7727	7639	2230	2090	7764	1685
7575	7721	7633	2224	2084	7758	1679
7572	7718	7630	2221	2081	7755	1676
7583	7729	7639	2203	2081	7766	1682
7574	7720	7632	2219	2079	7755	1674
7574	7720	7632	2223	2083	7757	1678
7579	7725	7633	2234	2098	7762	1693
7574	7720	7632	2223	2083	7757	1678
7573	7719	7631	2222	2082	7756	1677
7570	7722	7628	2233	2121	7755	1626
7583	7729	7644	2218	2080	7773	1629
7566	7712	7623	2229	2083	7748	1685
7566	7712	7623	2229	2083	7748	1685
7566	7712	7623	2229	2083	7748	1685
7655	7782	7700	2182	2085	7804	
7646	7779	7695	2115	1989	7804	303
7577	7698	7629	1844	1831	7748	1971
7578	7699	7628	1838	1823	7745	1961
7578	7699	7628	1838	1823	7745	1961
7576	7697	7624	1830	1835	7745	1941
7549	7698	7640	1991	1808	7744	1865
7657	7762	7660	2182	1892	7796	1570
7694	7799	7707	2300	1996	7828	1489
7701	7806	7715	2325	2015	7835	1462
7701	7806	7715	2325	2015	7835	1462
7703	7810	7720	2334	2064	7847	1407
7707	7812	7722	2300	2038	7847	1435
7673	7778	7680	2290	2059	7793	1368
7670	7775	7688	2309	2187	7812	1627
7698	7854	7768	2376	2123	7891	1599
7697	7853	7760	2355	2132	7893	1654
7529	7687	7609	1939	840	7733	2137
7550	7708	7630	1956	855	7752	2150
7555	7701	7617	1882	739	7739	2079

7557	7703	7619	1886	741	7741	2083
7560	7706	7627	1894	711	7744	2107
7529	7677	7584	1941	709	7720	2136
7557	7703	7619	1975	607	7748	2128
7559	7705	7621	1975	607	7750	2128
7553	7703	7630	1995	641	7744	2120
7555	7705	7632	1997	643	7746	2122
7553	7703	7630	1997	643	7744	2122
7539	7689	7626	2008	659	7732	2133
7551	7701	7630	1988	638	7746	2121
7539	7689	7607	1994	708	7730	2145
7540	7690	7608	1995	709	7729	2146
7566	7716	7640	1813		7777	2085
7567	7717	7641	1809	28	7778	2077
7611	7718	7655	1951	1885	7746	2074
7613	7720	7657	1953	1887	7748	2074
7603	7710	7647	1943	1877	7738	2066
7602	7709	7646	1942	1876	7737	2065
7611	7718	7659	1962	1904	7746	2073
7602	7709	7646	1942	1876	7737	2065
7603	7710	7647	1943	1877	7738	2066
7615	7722	7659	1957	1891	7750	2080
7592	7699	7644	1945	1908	7731	2108
7650	7691	7639	1965	1927	7727	2100
7738	7833	7730	334	1992	7900	2321
7741	7838	7735	358	2012	7907	2335
7753	7848	7745	346	2008	7915	2337
7727	7822	7721	359	1953	7889	2292
7724	7819	7713		1813	7880	2182
7718	7813	7711	81	1800	7874	2187
7718	7813	7711	81	1800	7874	2187
7715	7810	7708	79	1798	7871	2185
7718	7813	7715	81	1810	7876	2191
7692	7787	7694	101	1777	7848	2161
7723	7818	7719	95	1792	7879	2163
7690	7782	7675	249	1883	7847	2264
7679	7792	7689	290	1873	7851	2198
7718	7815	7717	177	1790	7871	2169
7704	7816	7720	241	1837	7872	2197
7723	7820	7722	198	1807	7871	2192
7727	7824	7724	203	1812	7876	2197
7692	7799	7705	252	1817	7853	2178
7722	7819	7715	166	1807	7873	2192
7711	7808	7706	155	1798	7864	2187
7678	7777	7684	318	1725	7829	2144
7681	7780	7687	327	1729	7832	2148
7682	7757	7640	529	1920	7817	2291
7691	7766	7649	538	1929	7826	2300
7675	7750	7633	522	1913	7810	2284
7685	7776	7676	482	1788	7832	2197
7674	7773	7685	337	1731	7829	2151
7677	7776	7686	337	1732	7832	2151
7562	7691	7571	748	1997	7746	2396
7681	7778	7669	505	1876	7834	2234
7671	7768	7657	502	1873	7826	2233

7680	7777	7666	520	1883	7835	2243
7687	7784	7673	515	1886	7842	2246
7677	7758	7660	504	1864	7822	2262
7702	7801	7689	419	1800	7855	2217
7692	7791	7677	412	1793	7845	2210
7690	7789	7675	410	1791	7843	2208
7673	7772	7680	340	1727	7822	2164
7653	7752	7667	331	1730	7804	2150
7698	7797	7705	362	1763	7849	2180
7691	7790	7698	353	1756	7842	2171
7661	7760	7670	498	1616	7811	2091
7661	7760	7670	498	1616	7811	2091
7676	7775	7682	354	1765	7831	2190
7671	7770	7677	349	1760	7826	2185
7674	7773	7680	352	1763	7829	2188
7516	7666	7556	1956	1771	7699	2120
7513	7663	7553	1957	1772	7694	2121
7503	7653	7544	1944	1759	7686	2108
7599	7749	7627	2075	1980	7771	2330
7528	7678	7550	1924	1829	7695	2145
7752	7902	7788	2216	2002	7927	2355
7767	7873	7728	3905	3865	7925	3923
7906	7958	7750	6747	6689	7966	6734
7908	7960	7752	6749	6691	7968	6736
7905	7957	7749	6746	6688	7965	6733
7907	7959	7751	6748	6690	7967	6735
7875	7925	7711	6812	6755	7944	6797
7893	7943	7717	6803	6739	7958	6790
7904	7954	7726	6813	6753	7967	6788
7883	7932	7706	6819	6781	7949	6818
7898	7947	7723	6833	6795	7966	6832
7887	7936	7710	6800	6778	7951	6813
7900	7946	7721	6859	6805	7966	6858
7876	7925	7699	6811	6773	7940	6808
7905	7954	7728	6841	6803	7968	6838
7869	7916	7690	6812	6760	7933	6789
7876	7925	7701	6809	6771	7942	6806
7880	7929	7705	6817	6779	7946	6814
8011	8052	7815	6989	6916	8062	6955
8006	8047	7810	6984	6911	8057	6950
7969	8035	7794	6943	6882	8043	6915
7905	7969	7729	6852	6800	7978	6799
8008	8056	7836	6955	6912	8072	6926
7894	7938	7704	6833	6790	7953	6814
7888	7932	7698	6827	6784	7947	6808
7889	7933	7699	6826	6783	7948	6807
7860	7904	7664	6795	6755	7921	6779
7767	7811	7595	6840	6802	7824	6841
7790	7881	7779	3659	3384	7894	3673
7779	7870	7766	3648	3373	7883	3662
7	293	3417	7726	7568	2046	7657
7	293	3417	7726	7568	2046	7657
6	292	3416	7725	7567	2045	7656
6	292	3416	7724	7566	2045	7655
6	292	3416	7726	7568	2045	7657

	292	3414	7724	7566	2045	7655
6	292	3416	7726	7568	2045	7657
4	290	3414	7724	7566	2043	7655
7	293	3417	7727	7569	2046	7658
4	290	3414	7724	7566	2043	7655
3	289	3413	7723	7565	2042	7654
4	290	3414	7724	7566	2043	7655
5	291	3415	7725	7567	2044	7656
5	291	3415	7725	7567	2044	7656
285	13	3284	7812	7709	1910	7775
289	17	3288	7816	7713	1914	7779
289	11	3288	7816	7713	1914	7779
292		3291	7819	7716	1917	7782
384	112	3353	7773	7666	1995	7730
286	18	3285	7813	7710	1911	7776
262	88	3285	7802	7702	1882	7767
260	86	3283	7800	7700	1880	7765
264	90	3287	7805	7705	1884	7770
259	81	3282	7800	7700	1879	7765
259	89	3284	7802	7702	1881	7767
269	97	3284	7807	7707	1891	7771
323	269	3372	7648	7558	2043	7652
814	790	3458	7728	7622	2113	7674
879	857	3499	7728	7636	2173	7674
2045	1917	3421	7880	7777		7804
2045	1917	3421	7880	7777	2	7804
2052	1924	3429	7885	7782	15	7809
2066	1935	3447	7915	7811	217	7844
2147	2033	3393	7887	7786	1917	7826
3407	3280	59	7705	7632	3414	7690
3410	3283	62	7708	7635	3417	7693
3414	3291		7713	7640	3421	7700
3431	3316	397	7718	7632	3454	7704
3362	3239	275	7686	7605	3344	7663
3370	3247	283	7694	7613	3352	7671
3361	3238	274	7685	7604	3343	7662
3413	3288	625	7759	7678	3383	7726
3415	3290	627	7761	7680	3385	7728
3420	3295	632	7765	7684	3390	7732
3432	3307	644	7778	7697	3401	7745
3425	3296	2344	7679	7589	3464	7696
3426	3297	2345	7680	7590	3465	7697
3426	3297	2345	7680	7590	3465	7697
3427	3298	2346	7681	7591	3466	7698
3425	3296	2344	7679	7589	3464	7696
3425	3296	2342	7681	7591	3464	7698
3501	3374	2650	7715	7609	3378	7715
3500	3373	2649	7714	7608	3377	7714
3499	3372	2648	7713	7607	3376	7713
3498	3371	2647	7712	7606	3375	7712
3500	3373	2649	7714	7608	3377	7714
3499	3372	2648	7713	7607	3376	7713
3531	3403	2680	7690	7584	3408	7690
3502	3375	2651	7716	7610	3379	7716
3522	3410	2647	7799	7714	3478	7795

3410	3298	2464	7781	7676	3315	7773
3413	3301	2467	7784	7679	3318	7776
3411	3299	2465	7782	7677	3316	7774
3408	3296	2464	7779	7674	3316	7771
25532	25558	25525	25045	25027	25595	24964
25531	25557	25524	25044	25026	25594	24963
31136	31185	31213	31011	31002	31273	30950

AZ-TG73651	AZ-TG73483	blood-09-1294	blood-10-1009	upec-128	upec-203	upec-205	upec-3
3736	3786	6646	908	7617	3767	3721	3596
3682	3696	6603	429	7562	3685	3655	3552
3682	3696	6607	431	7564	3685	3657	3552
3726	3748	6613	499	7598	3738	3685	3576
3709	3739	6612	467	7618	3723	3684	3571
3745	3771	6614	449	7581	3752	3696	3596
3745	3771	6614	449	7581	3752	3696	3596
3737	3759	6640	433	7605	3738	3712	3608
3739	3761	6642	435	7609	3740	3715	3611
3774	3796	6622	485	7560	3775	3749	3632
3778	3802	6626	590	7562	3781	3753	3655
3769	3787	6651	404	7608	3768	3725	3630
3847	3890	6658	502	7643	3880	3819	3719
3847	3890	6658	502	7643	3880	3819	3719
3777	3818	6634	315	7600	3808	3757	3650
3774	3817	6646	341	7604	3807	3754	3648
3661	3692	6597	271	7569	3682	3627	3522
3720	3741	6586	349	7585	3731	3674	3571
3765	3809	6628		7588	3802	3747	3634
3767	3821	6657	378	7570	3814	3787	3648
3804	3841	6660	387	7605	3831	3789	3675
3657	3698	6593	780	7585	3688	3681	3531
3751	3802	6597	619	7582	3795	3727	3630
3785	3814	6655	633	7613	3804	3751	3661
3660	3713	6596	565	7560	3703	3659	3538
3706	3742	6628	589	7628	3721	3688	3604
3631	3673	6645	1145	7640	3663	3660	3595
3772	3830	6628	1295	7636	3820	3763	3704
3757	3774	6623	969	7575	3764	3753	3673
3705	3744	6598	849	7580	3723	3667	3589
3705	3744	6598	849	7580	3723	3667	3589
3707	3746	6599	852	7583	3725	3670	3592
3673	3724	6645	901	7621	3714	3681	3577
3633	3669	6639	1138	7632	3659	3655	3601
3633	3669	6639	1138	7632	3659	3655	3601
1929	1896	6607	3570	7678	1889	2228	1940
1946	1921	6597	3567	7679	1914	2197	1937
1934	1910	6602	3576	7677	1903	2203	1913
1949	1919	6585	3553	7686	1912	2194	1950
1924	1892	6593	3557	7663	1871	2197	1914
1950	1920	6589	3574	7679	1913	2209	1925
1949	1919	6588	3573	7678	1912	2208	1924
1929	1893	6610	3584	7675	1886	2187	1916
1950	1926	6623	3572	7671	1919	2214	1945
1994	1964	6576	3558	7632	1957	2215	1965
2066	2046	6598	3438	7626	2036	2304	2055
1997	1999	6719	3609	7675	1992	2237	1952
1834	1903	6590	3435	7610	1896	2132	1816
1829	1898	6585	3430	7607	1891	2127	1811
1835	1904	6591	3436	7613	1897	2133	1817
1829	1898	6585	3430	7607	1891	2127	1811
1834	1903	6590	3435	7612	1896	2132	1816
1834	1940	6657	3582	7667	1933	2065	1794

1965	1954	6761	3681	7711	1947	2194	1884
1805	1810	6653	3572	7661	1803	1944	1792
1804	1809	6652	3571	7660	1802	1943	1791
1797	1802	6644	3566	7653	1795	1936	1786
1799	1804	6647	3568	7655	1797	1938	1788
1751	1776	6682	3627	7651	1769	1929	1740
1760	1785	6691	3635	7660	1778	1938	1749
1760	1785	6691	3636	7660	1778	1938	1749
1724	1749	6657	3599	7635	1742	1882	1737
1749	1774	6672	3622	7644	1767	1899	1758
1751	1766	6647	3598	7617	1759	1898	1761
1716	1741	6649	3591	7627	1734	1874	1729
1745	1760	6659	3602	7656	1753	1928	1808
1745	1760	6659	3602	7656	1753	1928	1808
1734	1749	6648	3591	7648	1742	1917	1797
1692	1715	6645	3600	7651	1708	1939	1790
1847	1808	6711	3587	7647	1798	2113	1831
1878	1839	6739	3618	7676	1829	2144	1862
1849	1810	6713	3589	7649	1800	2115	1833
1847	1808	6711	3587	7647	1798	2113	1831
2231	2276	6760	3745	7687	2269	83	2089
2234	2278	6762	3748	7689	2271	85	2091
2228	2272	6756	3742	7683	2265	79	2085
2225	2269	6753	3739	7680	2262	76	2082
2207	2260	6764	3741	7689	2253	120	2092
2223	2267	6757	3743	7682	2260	84	2080
2227	2271	6755	3741	7682	2264	78	2084
2232	2280	6766	3748	7683	2273	93	2099
2227	2271	6755	3741	7682	2264	78	2084
2226	2270	6754	3740	7681	2263	77	2083
2237	2267	6753	3747	7678	2260		2094
2222	2262	6740	3736	7694	2255	165	2151
2233	2277	6759	3685	7662	2270	194	2092
2233	2277	6759	3685	7662	2270	194	2092
2233	2277	6759	3685	7662	2270	194	2092
2192	2217	6733	3769	7728	2210	1626	2122
2125	2136	6733	3791	7730	2129	1522	2030
1850	1902	6607	3609	7679	1895	1901	2059
1838	1896	6611	3606	7676	1889	1881	2043
1838	1896	6611	3606	7676	1889	1881	2043
1828	1888	6607	3601	7672	1881	1881	2047
1979	1970	6724	3602	7681	1963	2010	1961
2184	2237	6734	3654	7704	2230	1832	1954
2302	2365	6719	3702	7743	2358	1953	2074
2331	2434	6709	3697	7751	2427	1930	2105
2331	2434	6709	3697	7751	2427	1930	2105
2350	2424	6720	3701	7750	2417	2005	2136
2316	2389	6719	3682	7758	2382	1965	2116
2298	2381	6702	3716	7716	2374	1863	2119
2301	2382	6693	3788	7726	2375	1874	2174
2354	2347	6772	3746	7794	2340	1837	2222
2331	2344	6783	3746	7793	2337	1890	2257
1939	1887	6661	3611	7649	1877	2098	560
1952	1897	6681	3628	7670	1887	2118	573
1878	1833	6673	3607	7657	1823	2056	475

1882	1837	6676	3607	7659	1827	2058	477
1890	1859	6674	3607	7667	1849	2090	439
1927	1910	6696	3593	7632	1900	2124	463
1973	1968	6701	3614	7661	1958	2147	334
1973	1968	6703	3614	7663	1958	2147	334
1963	1964	6725	3632	7670	1954	2092	2
1965	1966	6727	3634	7672	1956	2094	
1965	1966	6727	3634	7670	1956	2094	4
1977	1976	6732	3616	7668	1966	2115	40
1968	1967	6720	3632	7672	1957	2089	21
1972	1995	6736	3612	7648	1985	2083	117
1973	1996	6737	3613	7649	1986	2084	118
1807	1800	6688	3616	7680	1793	2121	643
1817	1806	6686	3617	7681	1799	2111	641
1941	1961	6665	3601	7697	1954	2089	1920
1943	1963	6665	3601	7699	1956	2089	1920
1933	1953	6657	3593	7689	1946	2080	1912
1932	1952	6656	3592	7688	1945	2080	1911
1952	1966	6641	3604	7701	1959	2094	1939
1932	1952	6656	3592	7688	1945	2080	1913
1933	1953	6657	3593	7689	1946	2081	1912
1947	1967	6671	3607	7701	1960	2095	1926
1942	1984	6670	3560	7688	1977	2127	1955
1953	1973	6706	3603	7681	1966	2130	1961
414	655	6688	3833	7776	648	2376	2141
434	675	6695	3838	7781	668	2390	2149
428	673	6705	3849	7791	666	2394	2157
433	650	6689	3858	7767	643	2357	2102
166	419	6746	3758	7761	412	2233	1997
129	396	6748	3757	7759	389	2240	1992
129	396	6748	3757	7759	389	2240	1992
127	394	6745	3755	7756	387	2238	1990
133	402	6754	3760	7763	395	2248	1996
182	391	6752	3762	7742	384	2224	1955
183	374	6752	3785	7765	367	2214	1968
315	514	6804	3841	7718	507	2294	2043
232	419	6741	3710	7737	412	2245	1997
121	316	6737	3787	7765	309	2210	1942
167	376	6772	3769	7768	369	2233	1949
124	333	6764	3763	7770	326	2235	1965
129	338	6770	3768	7772	331	2240	1970
180	365	6748	3747	7753	358	2219	1951
	353	6751	3765	7763	346	2237	1965
33	344	6742	3756	7754	337	2234	1960
262	149	6725	3776	7730	142	2177	1881
261	150	6730	3777	7733	143	2188	1875
453	398	6663	3824	7686	391	2374	2054
462	407	6672	3833	7695	400	2383	2063
446	391	6656	3817	7679	384	2367	2047
406	355	6713	3803	7720	348	2264	1904
277	144	6721	3776	7733	137	2198	1883
277	146	6722	3781	7734	139	2198	1884
689	578	6857	3758	7613	571	2434	2112
439	180	6719	3794	7717	155	2312	1996
436	157	6712	3786	7705	132	2317	1993

454	181	6717	3805	7714	156	2328	2003
449	176	6728	3802	7721	151	2331	2006
445	172	6661	3795	7710	147	2322	2011
353		6748	3809	7737	37	2267	1966
346	37	6741	3802	7725		2260	1956
344	43	6739	3800	7723	18	2258	1954
268	148	6725	3775	7726	138	2181	1891
262	149	6724	3753	7713	142	2191	1884
304	183	6745	3799	7751	164	2194	1916
295	174	6738	3792	7744	155	2187	1909
476	379	6697	3775	7713	372	2099	1806
476	379	6697	3775	7713	372	2099	1806
272	163	6728	3786	7731	142	2239	1926
267	158	6723	3781	7726	137	2234	1921
270	161	6726	3784	7729	140	2237	1924
1974	1908	6624	3557	7599	1901	2141	1852
1975	1909	6623	3559	7596	1902	2142	1851
1962	1896	6612	3547	7587	1889	2129	1840
2087	2044	6715	3710	7678	2037	2302	2043
1950	1864	6638	3575	7601	1857	2126	1878
2228	2176	6876	3774	7840	2169	2399	2029
3901	3910	6927	3372	7778	3903	3828	3802
6752	6749	3	6629	7778	6742	6754	6728
6754	6751	5	6631	7780	6744	6756	6730
6751	6748		6628	7777	6741	6753	6727
6753	6750	4	6630	7779	6743	6755	6729
6816	6817	221	6745	7738	6810	6817	6794
6806	6807	210	6757	7744	6800	6806	6782
6816	6817	214	6770	7751	6810	6810	6790
6826	6823	224	6804	7734	6816	6846	6816
6840	6837	240	6818	7751	6830	6860	6830
6801	6802	280	6807	7738	6795	6836	6811
6866	6863	271	6860	7749	6856	6874	6842
6818	6815	218	6784	7727	6808	6832	6808
6848	6845	248	6813	7756	6838	6862	6838
6819	6816	221	6775	7718	6809	6813	6795
6816	6813	222	6790	7729	6806	6828	6812
6824	6821	224	6790	7733	6814	6838	6814
6996	6993	568	6930	7858	6986	6966	6947
6991	6988	563	6925	7853	6981	6961	6942
6950	6947	550	6919	7835	6940	6930	6906
6859	6856	421	6804	7757	6849	6826	6833
6962	6961	573	6885	7865	6954	6950	6937
6840	6837	389	6788	7732	6830	6837	6812
6834	6831	383	6782	7726	6824	6831	6806
6833	6830	382	6787	7727	6823	6828	6805
6802	6799	490	6781	7688	6792	6804	6777
6852	6853	661	6790	7619	6846	6850	6820
3651	3703	6895	4227	7827	3696	3789	3291
3640	3692	6885	4216	7814	3685	3778	3280
7724	7704	7905	7493	3418	7694	7572	7557
7724	7704	7905	7493	3418	7694	7572	7557
7723	7703	7904	7492	3417	7693	7571	7556
7722	7702	7903	7491	3417	7692	7570	7555
7724	7704	7905	7493	3417	7694	7572	7557

7722	7702	7905	7491	3415	7692	7570	7555
7724	7704	7905	7493	3417	7694	7572	7557
7722	7702	7903	7491	3415	7692	7570	7555
7725	7705	7906	7494	3418	7695	7573	7558
7722	7702	7903	7491	3415	7692	7570	7555
7721	7701	7902	7490	3414	7691	7569	7554
7722	7702	7903	7491	3415	7692	7570	7555
7723	7703	7904	7492	3416	7693	7571	7556
7723	7703	7904	7492	3416	7693	7571	7556
7812	7794	7950	7586	3283	7784	7715	7698
7816	7798	7954	7590	3287	7788	7719	7702
7816	7798	7954	7590	3287	7788	7719	7702
7819	7801	7957	7593	3290	7791	7722	7705
7773	7755	7950	7527	3350	7745	7676	7653
7813	7795	7951	7587	3284	7785	7716	7699
7802	7784	7940	7580	3282	7774	7709	7690
7800	7782	7939	7578	3280	7772	7707	7688
7805	7787	7943	7583	3284	7777	7712	7693
7800	7782	7938	7578	3279	7772	7707	7688
7802	7784	7940	7579	3281	7774	7709	7690
7807	7789	7943	7585	3281	7779	7713	7694
7648	7620	7895	7487	3373	7610	7580	7573
7723	7704	7884	7499	3476	7694	7612	7602
7723	7704	7886	7497	3500	7694	7616	7612
7873	7855	7965	7664	3385	7845	7755	7746
7873	7855	7965	7664	3385	7845	7755	7746
7878	7860	7972	7671	3393	7850	7760	7751
7911	7888	8012	7719	3401	7878	7790	7789
7883	7856	7946	7664	3310	7846	7776	7769
7707	7681	7741	7546	634	7669	7616	7622
7710	7684	7744	7549	637	7672	7619	7625
7715	7689	7749	7556	627	7677	7628	7632
7720	7694	7726	7539	805	7682	7623	7624
7688	7662	7769	7527	644	7650	7600	7596
7696	7670	7777	7535	652	7658	7608	7604
7687	7661	7768	7526	643	7649	7599	7595
7761	7735	7775	7586	18	7723	7676	7670
7763	7737	7777	7588		7725	7678	7672
7767	7741	7782	7592	25	7729	7682	7676
7780	7754	7794	7605	37	7742	7695	7689
7683	7654	7764	7569	2306	7642	7622	7605
7684	7655	7765	7568	2307	7643	7623	7606
7684	7655	7765	7570	2307	7643	7623	7606
7685	7656	7766	7571	2308	7644	7624	7607
7683	7654	7764	7569	2306	7642	7622	7605
7685	7656	7767	7571	2305	7644	7624	7607
7719	7695	7785	7562	2582	7683	7655	7615
7718	7694	7784	7561	2581	7682	7654	7614
7717	7693	7783	7560	2580	7681	7653	7613
7716	7692	7782	7559	2579	7680	7652	7612
7718	7694	7784	7561	2581	7682	7654	7614
7717	7693	7783	7560	2580	7681	7653	7613
7694	7670	7766	7543	2612	7658	7630	7590
7720	7696	7786	7563	2583	7684	7656	7616
7802	7774	7816	7604	2603	7762	7724	7702

7779	7741	7980	7680	2450	7729	7696	7658
7782	7744	7985	7683	2453	7732	7699	7661
7780	7742	7983	7681	2451	7730	7697	7659
7777	7739	7978	7678	2450	7727	7694	7656
25045	25045	25057	24917	25551	25040	24970	24978
25044	25044	25056	24916	25550	25039	24969	24977
31005	31012	30817	30921	31207	31001	30938	31007

upec-33	AZ-TG71327	AZ-TG71195	AZ-TG71423	NA	12_ECOL	184_ECOL	283_ECOL
3649	3727	3526	3574	6806	7621	7533	6647
3554	3673	3473	3531	6765	7566	7470	6604
3554	3673	3471	3531	6769	7568	7470	6608
3591	3719	3543	3548	6775	7602	7517	6614
3566	3704	3527	3543	6774	7622	7523	6613
3590	3738	3556	3570	6776	7585	7490	6615
3590	3738	3556	3570	6776	7585	7490	6615
3607	3732	3525	3589	6802	7609	7498	6641
3611	3734	3527	3592	6804	7613	7501	6643
3645	3763	3581	3610	6784	7564	7450	6623
3660	3773	3578	3634	6788	7566	7443	6627
3626	3760	3546	3609	6813	7612	7482	6652
3749	3840	3653	3699	6820	7647	7582	6659
3749	3840	3653	3699	6820	7647	7582	6659
3659	3770	3588	3630	6796	7604	7534	6635
3656	3767	3585	3628	6808	7608	7538	6647
3533	3654	3482	3502	6759	7573	7481	6598
3584	3709	3547	3549	6748	7589	7514	6587
3627	3757	3560	3613	6790	7592	7493	6629
3644	3761	3605	3627	6811	7573	7483	6658
3667	3799	3613	3654	6824	7609	7514	6661
3591	3640	3507	3509	6755	7589	7503	6594
3655	3744	3590	3608	6759	7586	7500	6598
3667	3782	3619	3639	6817	7617	7523	6656
3544	3651	3508	3514	6758	7564	7490	6597
3615	3701	3561	3586	6790	7632	7535	6629
3542	3636	3508	3581	6807	7644	7552	6646
3675	3745	3634	3682	6792	7640	7538	6629
3649	3744	3580	3654	6785	7579	7480	6624
3600	3694	3532	3563	6760	7584	7498	6599
3600	3694	3532	3563	6760	7584	7498	6599
3603	3696	3534	3566	6761	7587	7501	6600
3602	3666	3556	3551	6801	7624	7498	6646
3548	3626	3501	3573	6801	7636	7543	6640
3548	3626	3501	3573	6801	7636	7543	6640
1811	1918	1973	1964	6690	7682	7617	6608
1802	1947	1977	1957	6680	7683	7615	6598
1784	1937	1960	1947	6685	7681	7616	6603
1781	1940	1986	1970	6668	7690	7614	6586
1786	1915	1949	1934	6676	7667	7600	6594
1800	1941	1961	1945	6672	7683	7607	6590
1799	1940	1960	1944	6671	7682	7608	6589
1786	1920	1958	1934	6693	7679	7603	6611
1812	1949	1990	1971	6706	7675	7603	6624
1854	1985	1985	1985	6659	7636	7575	6577
1914	2063	2067	2073	6681	7630	7534	6599
1911	2000	2011	1958	6800	7679	7582	6720
1696	1873	1787	1816	6687	7614	7570	6591
1691	1868	1782	1811	6682	7611	7569	6586
1697	1874	1788	1817	6688	7617	7575	6592
1691	1868	1782	1811	6682	7611	7569	6586
1696	1873	1787	1816	6687	7616	7574	6591
1524	1827	1967	1820	6728	7671	7605	6658

1738	1958	2002	1894	6842	7715	7619	6762
365	1814	1927	1810	6744	7665	7560	6654
364	1813	1926	1809	6743	7664	7560	6653
357	1806	1919	1804	6735	7657	7555	6645
359	1808	1921	1806	6738	7659	7557	6648
	1760	1919	1758	6765	7655	7564	6683
21	1769	1928	1767	6774	7664	7573	6692
25	1769	1928	1767	6774	7664	7573	6692
179	1731	1890	1755	6740	7639	7529	6658
216	1756	1913	1776	6755	7648	7541	6673
230	1756	1897	1779	6730	7621	7516	6648
171	1723	1882	1747	6732	7631	7521	6650
473	1742	1910	1822	6742	7660	7560	6660
473	1742	1910	1822	6742	7660	7560	6660
462	1731	1899	1811	6731	7652	7550	6649
328	1699	1903	1808	6728	7655	7569	6646
1421	1838	1903	1879	6806	7651	7542	6712
1452	1869	1934	1910	6834	7680	7571	6740
1423	1840	1905	1881	6808	7653	7544	6714
1421	1838	1903	1879	6806	7651	7542	6712
1920	2234	2160	2079	6845	7691	7581	6761
1922	2237	2162	2081	6847	7693	7583	6763
1916	2231	2156	2075	6841	7687	7577	6757
1913	2228	2153	2072	6838	7684	7574	6754
1917	2210	2147	2082	6849	7693	7585	6765
1911	2226	2151	2070	6842	7686	7576	6758
1915	2230	2155	2074	6840	7686	7576	6756
1904	2229	2166	2089	6851	7687	7581	6767
1915	2230	2155	2074	6840	7686	7576	6756
1914	2229	2154	2073	6839	7685	7575	6755
1929	2240	2127	2084	6838	7682	7572	6754
1980	2221	2176	2145	6825	7698	7585	6741
1923	2236	2102	2082	6844	7666	7568	6760
1923	2236	2102	2082	6844	7666	7568	6760
1923	2236	2102	2082	6844	7666	7568	6760
2046	2187	2108	2146	6814	7732	7657	6734
2003	2120	2077	2058	6814	7734	7648	6734
1790	1835	1992	2051	6684	7683	7579	6608
1774	1829	1993	2031	6688	7680	7580	6612
1774	1829	1993	2031	6688	7680	7580	6612
1758	1819	2001	2035	6684	7676	7578	6608
1852	1972	1899	1979	6805	7685	7551	6725
1981	2169	2228	1966	6813	7708	7659	6735
2111	2287	2240	2086	6798	7747	7696	6720
2166	2320	2271	2095	6804	7755	7703	6710
2166	2320	2271	2095	6804	7755	7703	6710
2131	2329	2329	2112	6815	7754	7705	6721
2133	2295	2298	2092	6812	7762	7709	6720
2103	2281	2197	2109	6803	7720	7675	6703
2049	2292	2233	2162	6778	7730	7672	6694
2164	2357	2275	2262	6875	7798	7700	6773
2185	2334	2284	2297	6886	7797	7699	6784
1782	1936	1887	614	6750	7653	7531	6662
1793	1951	1903	629	6770	7674	7552	6682
1698	1877	1839	531	6762	7661	7557	6674

1700	1881	1841	533	6765	7663	7559	6677
1716	1889	1849	495	6763	7671	7562	6675
1734	1928	1901	507	6785	7636	7531	6697
1788	1970	1913	398	6788	7665	7559	6702
1788	1970	1913	398	6790	7667	7561	6704
1738	1990	1953	116	6812	7674	7555	6726
1740	1992	1955	118	6814	7676	7557	6728
1740	1992	1955	118	6814	7674	7555	6728
1763	2000	1941	139	6819	7672	7541	6733
1745	1987	1953	111	6807	7676	7553	6721
1757	1993	1967	17	6823	7652	7541	6737
1758	1994	1968		6824	7653	7542	6738
1676	1800	1908	709	6779	7684	7568	6689
1670	1812	1909	699	6777	7685	7569	6687
1887	1944	83	1932	6742	7701	7613	6666
1887	1946	85	1932	6742	7703	7615	6666
1876	1936	77	1924	6734	7693	7605	6658
1878	1935	76	1923	6733	7692	7604	6657
1898	1955	108	1951	6718	7705	7613	6642
1878	1935	76	1925	6733	7692	7604	6657
1879	1936	75	1924	6734	7693	7605	6658
1893	1950	91	1938	6748	7705	7617	6672
1919	1937		1968	6747	7692	7594	6671
1944	1958	224	1971	6783	7685	7652	6707
1947	331	2097	2141	6759	7780	7740	6689
1963	355	2106	2147	6766	7785	7743	6696
1963	347	2113	2159	6776	7795	7755	6706
1938	358	2110	2104	6760	7771	7729	6690
1769	81	1945	1995	6817	7765	7726	6747
1760		1937	1994	6819	7763	7720	6749
1760	2	1937	1994	6819	7763	7720	6749
1758	16	1935	1992	6816	7760	7717	6746
1772	52	1935	2002	6825	7767	7720	6755
1756	99	1923	1960	6823	7746	7694	6753
1744	90	1940	1968	6823	7771	7725	6753
1806	236	2022	2059	6813	7723	7692	6805
1834	277	1962	1999	6814	7741	7681	6742
1730	164	1932	1944	6810	7769	7720	6738
1747	227	1928	1953	6845	7772	7706	6773
1753	184	1942	1969	6837	7774	7725	6765
1758	189	1947	1974	6843	7776	7729	6771
1769	238	1937	1955	6821	7757	7694	6749
1751	129	1942	1973	6824	7767	7724	6752
1746	118	1925	1966	6815	7758	7713	6743
1699	309	1916	1903	6798	7734	7680	6726
1700	314	1918	1915	6803	7737	7683	6731
1910	498	2049	2082	6736	7690	7684	6664
1919	507	2058	2091	6745	7699	7693	6673
1903	491	2042	2075	6729	7683	7677	6657
1756	451	1957	1932	6788	7724	7687	6714
1707	318	1914	1921	6794	7737	7676	6722
1708	318	1914	1922	6795	7738	7679	6723
1983	732	2111	2141	6760	7618	7564	6858
1852	486	2012	2022	6792	7721	7683	6720
1851	479	2004	2023	6785	7709	7673	6713

1861	497	2014	2033	6790	7718	7682	6718
1864	492	2017	2036	6801	7725	7689	6729
1849	486	2010	2044	6734	7714	7679	6662
1776	396	1984	1996	6821	7741	7704	6749
1769	389	1977	1986	6814	7729	7694	6742
1767	387	1975	1984	6812	7727	7692	6740
1717	313	1929	1919	6798	7730	7675	6726
1703	315	1895	1917	6797	7717	7655	6725
1735	353	1954	1940	6818	7755	7700	6746
1726	344	1947	1933	6811	7748	7693	6739
1645	491	1842	1816	6778	7717	7663	6698
1645	491	1842	1816	6778	7717	7663	6698
1748	315	1929	1960	6801	7735	7678	6729
1744	310	1924	1955	6796	7730	7673	6724
1747	313	1927	1958	6799	7733	7676	6727
1910	1955	1909	1872	6693	7603	7518	6625
1911	1956	1910	1871	6692	7600	7515	6624
1898	1943	1897	1860	6681	7591	7505	6613
2071	2054	2150	2075	6794	7682	7601	6716
1922	1915	1964	1906	6717	7605	7530	6639
2167	2195	2132	2049	6955	7844	7754	6877
3782	3893	3790	3760	7028	7782	7769	6928
6683	6749	6671	6738	225	7783	7906	
6685	6751	6673	6740	227	7785	7908	6
6682	6748	6670	6737	224	7782	7905	3
6684	6750	6672	6739	226	7784	7907	5
6744	6813	6702	6807	111	7743	7875	222
6735	6801	6710	6798	100	7749	7893	211
6737	6813	6723	6802	104	7756	7904	215
6775	6821	6757	6826	36	7739	7883	225
6789	6835	6771	6840	52	7756	7898	241
6770	6800	6746	6823	92	7743	7887	281
6807	6861	6795	6852	92	7754	7900	272
6759	6813	6741	6818	14	7732	7876	219
6789	6843	6771	6848	44	7761	7905	249
6742	6814	6724	6805	35	7722	7869	222
6761	6811	6743	6822	22	7734	7876	223
6765	6819	6747	6824		7738	7880	225
6892	6989	6919	6955	397	7863	8011	569
6887	6984	6914	6950	392	7858	8006	564
6857	6943	6877	6914	383	7840	7969	551
6768	6852	6767	6841	231	7761	7905	422
6876	6955	6889	6945	369	7870	8008	574
6758	6833	6760	6820	185	7737	7894	390
6752	6827	6754	6814	179	7731	7888	384
6751	6826	6753	6813	190	7731	7889	383
6721	6795	6730	6785	292	7693	7860	491
6769	6845	6759	6833	461	7624	7767	662
3534	3652	3585	3262	6926	7831	7792	6896
3523	3641	3574	3251	6916	7818	7781	6886
7564	7720	7594	7542	7880	3423	7	7906
7564	7720	7594	7542	7880	3423	7	7906
7563	7719	7593	7541	7879	3422	6	7905
7562	7718	7592	7540	7878	3422	6	7904
7564	7720	7594	7542	7880	3422	6	7906

7562	7718	7592	7540	7880	3420	6	7906
7564	7720	7594	7542	7880	3422		7906
7562	7718	7592	7540	7878	3420	2	7904
7565	7721	7595	7543	7881	3423	7	7907
7562	7718	7592	7540	7878	3420	4	7904
7561	7717	7591	7539	7877	3419	3	7903
7562	7718	7592	7540	7878	3420	4	7904
7563	7719	7593	7541	7879	3421	5	7905
7563	7719	7593	7541	7879	3421	5	7905
7686	7806	7692	7683	7922	3288	285	7951
7690	7810	7696	7687	7926	3292	289	7955
7690	7810	7696	7687	7926	3292	289	7955
7693	7813	7699	7690	7929	3295	292	7958
7645	7767	7657	7638	7928	3355	384	7951
7687	7807	7693	7684	7923	3289	286	7952
7678	7796	7684	7675	7917	3287	262	7941
7676	7794	7682	7673	7916	3285	260	7940
7681	7799	7687	7678	7920	3289	264	7944
7676	7794	7682	7673	7915	3284	259	7939
7678	7796	7684	7675	7917	3286	259	7941
7682	7801	7690	7679	7920	3286	269	7944
7532	7642	7583	7558	7870	3378	323	7896
7585	7722	7602	7585	7839	3481	814	7885
7583	7722	7598	7595	7829	3505	879	7887
7707	7874	7731	7729	7946	3390	2045	7966
7707	7874	7731	7729	7946	3390	2045	7966
7712	7879	7736	7734	7953	3398	2052	7973
7745	7909	7771	7772	7993	3406	2066	8013
7722	7883	7757	7752	7929	3315	2147	7947
7610	7703	7636	7598	7694	639	3409	7742
7613	7706	7639	7601	7697	642	3412	7745
7618	7711	7644	7608	7705	632	3416	7750
7612	7716	7641	7600	7682	810	3433	7727
7589	7684	7610	7574	7725	649	3364	7770
7597	7692	7618	7582	7733	657	3372	7778
7588	7683	7609	7573	7724	648	3363	7769
7649	7757	7686	7647	7731	23	3415	7776
7651	7759	7688	7649	7733	25	3417	7778
7655	7763	7692	7653	7738		3422	7783
7668	7776	7705	7666	7750	42	3434	7795
7604	7675	7632	7586	7730	2311	3425	7765
7605	7676	7633	7587	7731	2312	3426	7766
7605	7676	7633	7587	7731	2312	3426	7766
7606	7677	7634	7588	7732	2313	3427	7767
7604	7675	7632	7586	7730	2311	3425	7765
7606	7677	7634	7588	7732	2310	3425	7768
7606	7712	7652	7597	7767	2587	3501	7786
7605	7711	7651	7596	7766	2586	3500	7785
7604	7710	7650	7595	7765	2585	3499	7784
7603	7709	7649	7594	7764	2584	3498	7783
7605	7711	7651	7596	7766	2586	3500	7785
7604	7710	7650	7595	7765	2585	3499	7784
7583	7687	7627	7572	7792	2617	3531	7767
7607	7713	7653	7598	7768	2588	3502	7787
7701	7795	7707	7683	7792	2608	3524	7817

7689	7772	7697	7642	7950	2455	3412	7981
7692	7775	7700	7645	7955	2458	3415	7986
7690	7773	7698	7643	7953	2456	3413	7984
7687	7770	7695	7640	7948	2455	3410	7979
25008	25040	25028	24968	25023	25555	25534	25059
25007	25039	25027	24967	25022	25554	25533	25058
30928	31009	30973	31007	30801	31209	31138	30819

775_SBOY	966_ECOL	C260_92	IHD45_5	IHD717_3	IHD717_9	IHD717_16	IHD813_9	IHD813_16
6791	6648	828	3821	3761	3671	3787	1050	1050
6750	6605		3767	3703	3592	3726	576	576
6754	6609	14	3767	3703	3592	3726	578	578
6760	6615	254	3806	3750	3631	3763	600	600
6759	6614	195	3785	3731	3621	3755	593	593
6761	6616	192	3821	3767	3639	3770	599	599
6761	6616	192	3821	3767	3639	3770	599	599
6787	6642	142	3824	3762	3647	3781	588	588
6789	6644	144	3826	3764	3649	3783	590	590
6769	6624	351	3866	3802	3669	3803	618	618
6773	6628	459	3867	3801	3666	3802	653	653
6798	6653	191	3854	3790	3657	3792	623	623
6805	6660	576	3942	3876	3736	3872		
6805	6660	576	3942	3876	3736	3872		
6781	6636	428	3872	3806	3681	3815	195	195
6793	6648	428	3869	3803	3678	3812	191	191
6744	6599	346	3752	3686	3557	3695	412	412
6733	6588	399	3815	3735	3606	3744	444	444
6775	6630	429	3849	3787	3654	3788	502	502
6792	6659	597	3894	3790	3702	3828	675	675
6811	6662	484	3892	3826	3714	3835	563	563
6740	6595	722	3740	3686	3610	3750	838	838
6744	6599	511	3842	3790	3649	3783	630	630
6802	6657	493	3872	3820	3671	3807	452	452
6743	6598	430	3747	3695	3563	3703	651	651
6775	6630	550	3787	3731	3598	3738	622	622
6790	6647	1142	3728	3662	3586	3697	1207	1207
6793	6630	1278	3851	3799	3683	3764	1292	1292
6770	6625	962	3847	3777	3656	3791	1029	1029
6745	6600	784	3786	3720	3585	3714	916	916
6745	6600	784	3786	3720	3585	3714	916	916
6746	6601	787	3788	3722	3589	3718	919	919
6782	6647	859	3758	3688	3580	3719	986	986
6784	6641	1129	3718	3652	3584	3693	1201	1201
6784	6641	1129	3718	3652	3584	3693	1201	1201
6667	6609	3481	2151	1908	2246	2374	3649	3649
6657	6599	3478	2156	1917	2229	2357	3655	3655
6662	6604	3484	2144	1909	2228	2350	3657	3657
6645	6587	3445	2155	1908	2230	2329	3640	3640
6653	6595	3455	2130	1883	2231	2351	3636	3636
6649	6591	3471	2156	1909	2243	2363	3641	3641
6648	6590	3470	2155	1908	2242	2362	3640	3640
6670	6612	3469	2135	1888	2235	2355	3648	3648
6683	6625	3485	2160	1921	2242	2370	3646	3646
6636	6578	3503	2200	1953	2257	2377	3651	3651
6658	6600	3486	2272	2035	2332	2444	3655	3655
6777	6721	3540	2165	1970	2275	2440	3725	3725
6664	6592	3364	2082	1811	2055	2082	3542	3542
6659	6587	3359	2077	1806	2050	2077	3537	3537
6665	6593	3365	2083	1812	2056	2083	3543	3543
6659	6587	3359	2077	1806	2050	2077	3537	3537
6664	6592	3364	2082	1811	2055	2082	3542	3542
6705	6659	3525	2029	1815	1947	2043	3689	3689

6819	6763	3576	2153	1930	2089	2154	3759	3759
6721	6655	3499	2010	1784	2026	2066	3694	3694
6720	6654	3498	2009	1783	2025	2065	3693	3693
6712	6646	3493	2002	1776	2020	2058	3688	3688
6715	6649	3495	2004	1778	2022	2060	3690	3690
6742	6684	3554	1963	1730	1981	2049	3749	3749
6751	6693	3563	1972	1739	1990	2058	3757	3757
6751	6693	3563	1972	1739	1990	2058	3758	3758
6717	6659	3526	1943	1703	1962	2032	3721	3721
6732	6674	3549	1968	1728	1979	2049	3744	3744
6707	6649	3510	1968	1728	1991	2061	3705	3705
6709	6651	3518	1935	1695	1954	2024	3713	3713
6719	6661	3527	1954	1724	1973	2051	3722	3722
6719	6661	3527	1954	1724	1973	2051	3722	3722
6708	6650	3516	1943	1713	1962	2040	3711	3711
6705	6647	3527	1911	1671	1992	2070	3724	3724
6783	6713	3516	2021	1828	1892	2148	3706	3706
6811	6741	3547	2052	1859	1923	2179	3737	3737
6785	6715	3518	2023	1830	1894	2150	3708	3708
6783	6713	3516	2021	1828	1892	2148	3706	3706
6820	6762	3683	2388	2205	1883	1925	3845	3845
6822	6764	3686	2391	2207	1885	1927	3848	3848
6816	6758	3680	2385	2201	1879	1921	3842	3842
6813	6755	3677	2382	2198	1876	1918	3839	3839
6824	6766	3686	2364	2180	1908	1950	3851	3851
6817	6759	3677	2380	2196	1884	1926	3837	3837
6815	6757	3679	2384	2200	1878	1920	3841	3841
6826	6768	3686	2389	2209	1867	1909	3848	3848
6815	6757	3679	2384	2200	1878	1920	3841	3841
6814	6756	3678	2383	2199	1877	1919	3840	3840
6813	6755	3655	2394	2210	1832	1874	3819	3819
6800	6742	3672	2389	2183	1864	1935	3832	3832
6819	6761	3617	2390	2206	1946	1921	3794	3794
6819	6761	3617	2390	2206	1946	1921	3794	3794
6819	6761	3617	2390	2206	1946	1921	3794	3794
6789	6735	3688	2337	2169	1570	1627	3848	3848
6789	6735	3692	2260	2102	1550	1692	3860	3860
6661	6609	3526	1995	1809	2072	2165	3702	3702
6665	6613	3525	1977	1815	2060	2149	3700	3700
6665	6613	3525	1977	1815	2060	2149	3700	3700
6661	6609	3528	1969	1807	2048	2145	3709	3709
6782	6726	3492	2160	1974	1963	2021	3694	3694
6790	6736	3592	2339	2169		897	3736	3736
6775	6721	3640	2457	2287	245	710	3784	3784
6781	6711	3635	2478	2318	396	745	3779	3779
6781	6711	3635	2478	2318	396	745	3779	3779
6792	6722	3624	2501	2325	459	774	3772	3772
6789	6721	3612	2467	2291	419	728	3761	3761
6780	6704	3654	2441	2279	534	762	3804	3804
6755	6695	3726	2456	2288	897		3872	3872
6852	6774	3656	2505	2335	1854	1741	3820	3820
6863	6785	3656	2484	2316	1909	1786	3820	3820
6731	6663	3500	2112	1906	1887	2148	3671	3671
6751	6683	3502	2129	1929	1907	2166	3683	3683
6743	6675	3533	2055	1855	1835	2094	3702	3702

6746	6678	3533	2059	1859	1837	2096	3702	3702
6744	6676	3533	2067	1867	1853	2114	3702	3702
6766	6698	3531	2109	1914	1902	2161	3680	3680
6769	6703	3525	2143	1948	1933	2175	3710	3710
6771	6705	3525	2143	1948	1933	2175	3710	3710
6793	6727	3550	2155	1940	1952	2172	3717	3717
6795	6729	3552	2157	1942	1954	2174	3719	3719
6795	6729	3552	2157	1942	1954	2174	3719	3719
6800	6734	3539	2178	1951	1972	2196	3729	3729
6788	6722	3550	2156	1937	1959	2177	3718	3718
6804	6738	3530	2158	1943	1965	2161	3698	3698
6805	6739	3531	2159	1944	1966	2162	3699	3699
6760	6690	3562	2008	1790	1892	2187	3717	3717
6758	6688	3563	2004	1786	1896	2191	3727	3727
6719	6667	3513	2107	1914	2199	2202	3672	3672
6719	6667	3513	2109	1916	2201	2204	3672	3672
6711	6659	3505	2099	1906	2191	2194	3664	3664
6710	6658	3504	2098	1905	2190	2193	3663	3663
6695	6643	3494	2118	1925	2210	2213	3665	3665
6710	6658	3504	2098	1905	2190	2195	3663	3663
6711	6659	3505	2099	1906	2191	2194	3664	3664
6725	6673	3519	2113	1920	2205	2208	3678	3678
6724	6672	3473	2113	1932	2228	2233	3653	3653
6760	6708	3536	2121	1926	2253	2256	3691	3691
6754	6690	3751	44	415	2325	2442	3928	3928
6761	6697	3753	68	435	2341	2458	3930	3930
6771	6707	3767		433	2339	2456	3942	3942
6755	6691	3776	85	410	2312	2429	3951	3951
6812	6748	3670	346	177	2182	2309	3851	3851
6814	6750	3673	347	164	2169	2292	3840	3840
6814	6750	3673	347	164	2169	2292	3840	3840
6811	6747	3671	345	162	2167	2290	3838	3838
6820	6756	3671	351	170	2181	2304	3844	3844
6818	6754	3669	375	152	2170	2295	3879	3879
6818	6754	3697	355	134	2165	2286	3880	3880
6807	6806	3759	495	276	2246	2360	3935	3935
6809	6743	3696	546	165	2218	2337	3864	3864
6805	6739	3703	433		2169	2288	3876	3876
6840	6774	3702	497	142	2186	2307	3896	3896
6832	6766	3705	454	99	2186	2307	3897	3897
6838	6772	3710	459	104	2191	2312	3902	3902
6816	6750	3703	508	153	2194	2315	3887	3887
6819	6753	3682	428	121	2184	2301	3847	3847
6810	6744	3671	425	106	2179	2296	3832	3832
6793	6727	3690	562	217	2155	2292	3867	3867
6798	6732	3691	569	224	2156	2295	3867	3867
6731	6665	3814	787	450	2314	2457	3920	3920
6740	6674	3823	796	459	2323	2466	3929	3929
6724	6658	3807	780	443	2307	2450	3913	3913
6770	6715	3726	699	403	2202	2342	3894	3894
6789	6723	3693	591	232	2166	2307	3867	3867
6790	6724	3696	591	232	2166	2307	3872	3872
6751	6859	3719	1000	647	2430	2551	3927	3927
6787	6721	3676	751	402	2270	2409	3859	3859
6780	6714	3666	756	399	2267	2412	3851	3851

6785	6719	3690	774	417	2278	2423	3870	3870
6796	6730	3685	769	412	2281	2426	3865	3865
6729	6663	3674	758	401	2285	2426	3891	3891
6816	6750	3696	673	316	2237	2382	3890	3890
6809	6743	3685	666	309	2230	2375	3880	3880
6807	6741	3683	664	307	2228	2373	3878	3878
6793	6727	3684	584	241	2145	2284	3843	3843
6792	6726	3662	573	226	2156	2299	3866	3866
6813	6747	3696	604	261	2173	2311	3891	3891
6806	6740	3689	595	252	2166	2304	3884	3884
6769	6699	3697	710	435	2107	2214	3868	3868
6769	6699	3697	710	435	2107	2214	3868	3868
6796	6730	3690	608	281	2209	2347	3856	3856
6791	6725	3685	603	276	2204	2343	3851	3851
6794	6728	3688	606	279	2207	2346	3854	3854
6670	6626	3460	2121	1943	2180	2382	3647	3647
6669	6625	3462	2122	1944	2181	2383	3649	3649
6658	6614	3450	2109	1931	2168	2370	3637	3637
6771	6717	3628	2158	2086	2271	2435	3803	3803
6694	6640	3487	2073	1923	2131	2296	3677	3677
6932	6878	3700	2359	2193	2397	2587	3874	3874
7003	6929	3344	4046	3920	3836	3805	3526	3526
222	5	6604	6706	6738	6735	6694	6659	6659
224	7	6606	6708	6740	6737	6696	6661	6661
221	4	6603	6705	6737	6734	6693	6658	6658
223		6605	6707	6739	6736	6695	6660	6660
98	223	6721	6771	6809	6791	6752	6796	6796
87	212	6732	6760	6798	6775	6736	6784	6784
91	216	6745	6768	6802	6783	6746	6796	6796
47	226	6779	6778	6812	6817	6780	6834	6834
63	242	6793	6792	6826	6831	6796	6848	6848
103	282	6760	6755	6791	6807	6772	6810	6810
103	273	6835	6818	6852	6857	6812	6890	6890
29	220	6759	6770	6804	6807	6772	6814	6814
59	250	6788	6800	6834	6837	6802	6843	6843
	223	6750	6771	6805	6790	6755	6805	6805
33	224	6765	6768	6802	6805	6770	6820	6820
35	226	6765	6776	6810	6813	6778	6820	6820
392	570	6972	6951	6982	6960	6913	7009	7009
387	565	6967	6946	6977	6955	6908	7004	7004
390	552	6931	6905	6936	6926	6877	6962	6962
209	423	6774	6811	6845	6822	6785	6835	6835
384	575	6903	6914	6948	6932	6897	6857	6857
200	391	6739	6792	6826	6821	6786	6830	6830
194	385	6733	6786	6820	6815	6780	6824	6824
191	384	6738	6785	6819	6814	6779	6829	6829
301	492	6719	6754	6788	6790	6757	6810	6810
470	663	6740	6803	6845	6837	6814	6857	6857
6919	6897	4161	3745	3626	3687	3711	4290	4290
6909	6887	4150	3734	3615	3676	3700	4279	4279
7869	7907	7470	7755	7720	7659	7672	7582	7582
7869	7907	7470	7755	7720	7659	7672	7582	7582
7868	7906	7469	7754	7719	7658	7671	7581	7581
7867	7905	7468	7753	7718	7657	7670	7580	7580
7869	7907	7470	7755	7720	7659	7672	7582	7582

7869	7907	7468	7753	7718	7657	7670	7580	7580
7869	7907	7470	7755	7720	7659	7672	7582	7582
7867	7905	7468	7753	7718	7657	7670	7580	7580
7870	7908	7471	7756	7721	7660	7673	7583	7583
7867	7905	7468	7753	7718	7657	7670	7580	7580
7866	7904	7467	7752	7717	7656	7669	7579	7579
7867	7905	7468	7753	7718	7657	7670	7580	7580
7868	7906	7469	7754	7719	7658	7671	7581	7581
7868	7906	7469	7754	7719	7658	7671	7581	7581
7909	7952	7562	7841	7808	7755	7768	7659	7659
7913	7956	7566	7845	7812	7759	7772	7663	7663
7913	7956	7566	7845	7812	7759	7772	7663	7663
7916	7959	7569	7848	7815	7762	7775	7666	7666
7911	7952	7495	7800	7769	7710	7727	7600	7600
7910	7953	7563	7842	7809	7756	7769	7660	7660
7906	7942	7556	7831	7798	7747	7758	7653	7653
7905	7941	7554	7829	7796	7745	7756	7651	7651
7909	7945	7559	7834	7801	7750	7761	7656	7656
7904	7940	7554	7829	7796	7745	7756	7651	7651
7906	7942	7555	7831	7798	7747	7758	7652	7652
7909	7945	7561	7836	7803	7753	7762	7658	7658
7859	7897	7444	7677	7644	7632	7645	7606	7606
7826	7886	7460	7753	7721	7658	7680	7507	7507
7820	7888	7458	7753	7721	7658	7686	7505	7505
7933	7967	7631	7915	7871	7796	7812	7736	7736
7933	7967	7631	7915	7871	7796	7812	7736	7736
7940	7974	7638	7920	7876	7801	7817	7745	7745
7980	8014	7688	7948	7909	7834	7850	7795	7795
7916	7948	7620	7905	7880	7812	7837	7737	7737
7679	7743	7510	7737	7709	7650	7678	7607	7607
7682	7746	7513	7740	7712	7653	7681	7610	7610
7690	7751	7520	7745	7717	7660	7688	7617	7617
7667	7728	7499	7742	7722	7665	7696	7604	7604
7710	7771	7487	7718	7690	7633	7655	7588	7588
7718	7779	7495	7726	7698	7641	7663	7596	7596
7709	7770	7486	7717	7689	7632	7654	7587	7587
7716	7777	7560	7789	7763	7702	7724	7641	7641
7718	7779	7562	7791	7765	7704	7726	7643	7643
7722	7784	7566	7795	7769	7708	7730	7647	7647
7735	7796	7579	7808	7782	7721	7743	7660	7660
7715	7766	7542	7694	7685	7651	7704	7616	7616
7716	7767	7541	7695	7686	7652	7705	7615	7615
7716	7767	7543	7695	7686	7652	7705	7617	7617
7717	7768	7544	7696	7687	7653	7706	7618	7618
7715	7766	7542	7694	7685	7651	7704	7616	7616
7717	7769	7544	7696	7687	7653	7706	7618	7618
7752	7787	7508	7733	7725	7684	7708	7610	7610
7751	7786	7507	7732	7724	7683	7707	7609	7609
7750	7785	7506	7731	7723	7682	7706	7608	7608
7749	7784	7505	7730	7722	7681	7705	7607	7607
7751	7786	7507	7732	7724	7683	7707	7609	7609
7750	7785	7506	7731	7723	7682	7706	7608	7608
7777	7768	7489	7708	7700	7665	7683	7591	7591
7753	7788	7509	7734	7726	7685	7709	7611	7611
7780	7818	7573	7829	7804	7753	7787	7671	7671

7933	7982	7642	7812	7780	7729	7779	7722	7722
7938	7987	7645	7815	7783	7732	7781	7725	7725
7936	7985	7643	7813	7781	7730	7780	7723	7723
7931	7980	7640	7810	7778	7727	7777	7720	7720
25010	25058	24897	25050	25056	24959	24971	24952	24952
25009	25057	24896	25049	25055	24958	24970	24951	24951
30791	30820	30906	31031	31012	30967	30948	30934	30934

AZ_TG76998	H124600634	H134240608	AZ-TG71191	2012C-3377	2013C-4350	MGH108
7561	3556	944	3583	831	3756	1243
7510	3484	484	3531	142	3693	1278
7512	3484	486	3531	144	3693	1280
7542	3532	526	3547	320	3737	1281
7558	3507	523	3542	200	3718	1292
7537	3535	529	3579	141	3754	1295
7537	3535	529	3579	141	3754	1295
7559	3539	481	3581		3752	1290
7563	3541	483	3584	60	3754	1292
7513	3592	546	3594	300	3789	1328
7521	3618	640	3617	393	3795	1217
7562	3578	477	3605	163	3770	1314
7607	3657	563	3680	588	3867	1292
7607	3657	563	3680	588	3867	1292
7558	3592	412	3609	440	3797	1263
7563	3582	402	3607	440	3794	1253
7527	3473	296	3491	346	3671	1205
7543	3503	369	3538	413	3722	1237
7546	3576	387	3593	433	3776	1295
7538	3621	582	3611	553	3785	1453
7561	3625		3640	481	3816	1282
7537	3503	859	3489	734	3671	1209
7536	3609	679	3591	558	3781	1038
7565	3622	655	3628	501	3801	1179
7518	3507	667	3505	488	3684	1155
7580	3543	632	3586	549	3718	1064
7582	3470	1162	3562	1150	3645	559
7578	3614	1282	3642	1290	3810	
7525	3584	985	3629	971	3748	858
7522	3493	923	3551	787	3707	807
7522	3493	923	3551	787	3707	807
7525	3495	926	3553	790	3709	810
7569	3516	973	3541	871	3703	812
7575	3476	1161	3560	1147	3645	547
7575	3476	1161	3560	1147	3645	547
7627	127	3623	1950	3537	1852	3606
7626	108	3616	1959	3531	1855	3612
7626		3625	1947	3539	1839	3614
7633	167	3602	1976	3504	1848	3543
7610	92	3607	1940	3502	1833	3589
7628	118	3616	1949	3528	1849	3588
7627	117	3615	1948	3527	1848	3587
7624	160	3593	1944	3526	1822	3596
7620	162	3604	1971	3540	1861	3613
7579	224	3607	1998	3560	1893	3565
7575	437	3574	2077	3542	1981	3570
7616	1781	3691	1981	3595	1934	3643
7564	1767	3483	1888	3417	1836	3511
7561	1762	3478	1883	3412	1831	3506
7567	1768	3484	1889	3418	1837	3512
7561	1762	3478	1883	3412	1831	3506
7566	1767	3483	1888	3417	1836	3511
7622	1757	3646	1768	3578	1860	3636

7670	1970	3705	1858	3631	1885	3712
7614	1830	3612	1782	3552	1741	3600
7613	1829	3611	1781	3551	1740	3599
7606	1822	3606	1776	3546	1733	3594
7608	1824	3608	1778	3548	1735	3596
7610	1784	3667	1734	3607	1707	3675
7619	1793	3676	1743	3616	1716	3684
7619	1793	3676	1743	3616	1716	3684
7592	1785	3639	1709	3579	1680	3635
7601	1816	3660	1730	3602	1705	3658
7574	1786	3617	1733	3563	1697	3623
7584	1777	3631	1701	3571	1672	3627
7613	1855	3674	1763	3580	1691	3650
7613	1855	3674	1763	3580	1691	3650
7605	1844	3663	1752	3569	1680	3639
7608	1803	3660	1755	3580	1646	3644
7596	1839	3625	1745	3574	1745	3661
7625	1870	3656	1776	3605	1776	3692
7598	1841	3627	1747	3576	1747	3663
7596	1839	3625	1745	3574	1745	3661
7625	2196	3809	2097	3740	2207	3788
7627	2198	3812	2099	3743	2209	3791
7621	2192	3806	2093	3737	2203	3785
7618	2189	3803	2090	3734	2200	3782
7627	2193	3815	2100	3741	2186	3792
7620	2187	3801	2088	3734	2198	3780
7620	2191	3805	2092	3736	2202	3784
7621	2200	3812	2107	3743	2211	3791
7620	2191	3805	2092	3736	2202	3784
7619	2190	3804	2091	3735	2201	3783
7616	2203	3789	2124	3712	2198	3763
7632	2218	3792	2153	3727	2191	3779
7611	2211	3749	2094	3674	2208	3784
7611	2211	3749	2094	3674	2208	3784
7611	2211	3749	2094	3674	2208	3784
7690	2275	3795	2136	3743	2151	3762
7685	2231	3817	2048	3747	2068	3740
7621	1955	3649	2065	3583	1823	3699
7620	1935	3648	2057	3580	1819	3693
7620	1935	3648	2057	3580	1819	3693
7616	1936	3657	2047	3583	1813	3700
7630	2033	3640	1903	3561	1911	3638
7650	2228	3714	1902	3647	2166	3683
7697	2309	3762	2022	3695	2294	3733
7705	2362	3759	2051	3690	2359	3724
7705	2362	3759	2051	3690	2359	3724
7710	2365	3745	2078	3679	2354	3739
7712	2320	3730	2058	3667	2320	3712
7670	2348	3778	2083	3709	2304	3739
7678	2350	3835	2161	3781	2307	3764
7760	2417	3782	2193	3709	2275	3748
7752	2439	3782	2200	3709	2272	3738
7599	1857	3616	497	3556	1831	3611
7620	1864	3631	508	3558	1844	3623
7607	1827	3652	412	3589	1768	3664

7609	1831	3652	414	3589	1772	3664
7617	1862	3650	376	3589	1794	3658
7574	1947	3640		3581	1845	3642
7609	1884	3667	465	3583	1897	3657
7611	1884	3667	465	3583	1897	3657
7620	1911	3673	461	3606	1881	3702
7622	1913	3675	463	3608	1883	3704
7620	1913	3675	463	3608	1883	3704
7616	1941	3645	473	3582	1901	3718
7620	1920	3675	468	3608	1888	3699
7597	1946	3653	506	3588	1920	3681
7598	1947	3654	507	3589	1921	3682
7632	1846	3683	709	3617	1731	3684
7633	1842	3684	717	3618	1729	3694
7647	1925	3654	1874	3566	1891	3651
7649	1927	3654	1876	3566	1893	3651
7639	1917	3646	1866	3558	1883	3643
7638	1916	3645	1865	3557	1882	3642
7651	1930	3657	1889	3547	1896	3634
7638	1916	3645	1867	3557	1882	3642
7639	1917	3646	1866	3558	1883	3643
7651	1931	3660	1880	3572	1897	3657
7636	1960	3613	1901	3525	1914	3634
7631	2023	3668	1913	3589	1903	3662
7722	2128	3876	2095	3808	575	3839
7727	2134	3878	2101	3810	595	3841
7737	2144	3892	2109	3824	591	3851
7713	2119	3901	2054	3833	568	3860
7705	1948	3803	1941	3735	337	3742
7703	1937	3799	1928	3732	318	3745
7703	1937	3799	1928	3732	318	3745
7700	1935	3797	1926	3730	316	3743
7707	1945	3805	1932	3736	326	3753
7686	1928	3807	1899	3739	311	3788
7711	1915	3830	1916	3762	290	3783
7664	1994	3862	1999	3816	426	3841
7681	1961	3759	1979	3755	335	3783
7709	1909	3826	1914	3762	232	3799
7712	1916	3831	1925	3761	299	3815
7714	1930	3832	1933	3764	256	3818
7716	1935	3837	1938	3769	261	3823
7697	1920	3806	1917	3762	286	3814
7707	1934	3804	1927	3737	277	3772
7698	1929	3797	1922	3730	266	3757
7676	1833	3815	1847	3747	67	3804
7679	1831	3816	1845	3748	64	3808
7632	2017	3865	2008	3869	314	3855
7641	2026	3874	2017	3878	323	3864
7625	2010	3858	2001	3862	307	3848
7668	1938	3812	1866	3781	271	3824
7677	1839	3816	1845	3752		3810
7678	1840	3821	1846	3755	20	3815
7560	2077	3815	2060	3764	496	3884
7661	1956	3815	1944	3723	256	3754
7649	1953	3811	1939	3719	245	3749

7658	1961	3830	1949	3743	255	3768
7665	1966	3825	1952	3738	258	3763
7652	1946	3819	1957	3724	238	3831
7681	1910	3841	1910	3759	144	3830
7669	1903	3831	1900	3738	137	3820
7667	1901	3829	1898	3736	135	3818
7672	1839	3798	1843	3742	75	3782
7659	1838	3790	1844	3724	69	3811
7697	1861	3835	1878	3743	105	3828
7690	1852	3828	1871	3736	96	3821
7662	1789	3822	1748	3754	287	3793
7662	1789	3822	1748	3754	287	3793
7674	1875	3815	1865	3728	109	3791
7669	1870	3810	1860	3723	104	3786
7672	1873	3813	1863	3726	107	3789
7546	1817	3589	1867	3499	1841	3635
7543	1818	3591	1868	3501	1842	3636
7534	1805	3579	1855	3489	1829	3623
7617	2043	3752	2084	3667	1986	3758
7540	1854	3621	1913	3526	1803	3640
7778	2137	3819	2086	3737	2109	3843
7718	3840	3410	3780	3405	3924	3526
7742	6603	6661	6697	6641	6722	6629
7744	6605	6663	6699	6643	6724	6631
7741	6602	6660	6696	6640	6721	6628
7743	6604	6662	6698	6642	6723	6630
7703	6663	6773	6763	6757	6788	6782
7709	6651	6785	6755	6767	6780	6766
7718	6659	6790	6761	6776	6788	6780
7695	6687	6836	6787	6816	6796	6804
7712	6701	6850	6801	6830	6810	6818
7699	6675	6800	6782	6783	6785	6780
7710	6721	6894	6813	6872	6836	6862
7688	6679	6818	6779	6796	6788	6786
7717	6709	6847	6809	6825	6818	6816
7679	6662	6811	6766	6787	6789	6793
7690	6683	6822	6783	6802	6786	6790
7694	6685	6824	6785	6802	6794	6792
7804	6854	6988	6920	7009	6966	6995
7799	6849	6983	6915	7004	6961	6990
7783	6815	6950	6879	6968	6920	6936
7718	6699	6847	6804	6811	6829	6805
7825	6825	6943	6916	6940	6934	6924
7693	6681	6818	6789	6776	6810	6790
7687	6675	6812	6783	6770	6804	6784
7688	6674	6815	6782	6775	6803	6787
7653	6651	6796	6754	6756	6778	6698
7584	6694	6812	6797	6778	6824	6795
7766	3497	4267	3334	4200	3650	4196
7753	3486	4256	3323	4189	3639	4185
3410	7616	7514	7531	7498	7676	7538
3410	7616	7514	7531	7498	7676	7538
3409	7615	7513	7530	7497	7675	7537
3409	7614	7512	7529	7496	7674	7536
3409	7616	7514	7531	7498	7676	7538

3407	7614	7512	7529	7496	7674	7536
3409	7616	7514	7531	7498	7676	7538
3407	7614	7512	7529	7496	7674	7536
3410	7617	7515	7532	7499	7677	7539
3407	7614	7512	7529	7496	7674	7536
3406	7613	7511	7528	7495	7673	7535
3407	7614	7512	7529	7496	7674	7536
3408	7615	7513	7530	7497	7675	7537
3408	7615	7513	7530	7497	7675	7537
3273	7723	7608	7670	7590	7766	7630
3277	7727	7612	7674	7594	7770	7634
3277	7727	7612	7674	7594	7770	7634
3280	7730	7615	7677	7597	7773	7637
3342	7684	7548	7631	7523	7727	7577
3274	7724	7609	7671	7591	7767	7631
3278	7716	7602	7662	7584	7756	7624
3276	7714	7600	7660	7582	7754	7622
3280	7719	7605	7665	7587	7759	7627
3275	7714	7600	7660	7582	7754	7622
3277	7716	7601	7662	7583	7756	7623
3277	7720	7605	7667	7589	7761	7629
3365	7580	7522	7540	7472	7592	7550
3451	7621	7520	7571	7486	7678	7550
3492	7621	7518	7581	7484	7678	7548
3414	7756	7685	7720	7659	7829	7730
3414	7756	7685	7720	7659	7829	7730
3422	7761	7692	7725	7666	7834	7737
3440	7796	7742	7763	7716	7866	7785
3388	7752	7688	7735	7660	7840	7720
	7626	7561	7574	7559	7677	7578
11	7629	7566	7577	7562	7680	7581
59	7634	7573	7584	7569	7685	7588
406	7637	7554	7577	7546	7690	7583
284	7605	7542	7555	7532	7656	7565
292	7613	7550	7563	7540	7664	7573
283	7604	7541	7554	7531	7655	7564
632	7675	7603	7630	7603	7731	7634
634	7677	7605	7632	7605	7733	7636
639	7681	7609	7636	7609	7737	7640
651	7694	7622	7649	7622	7750	7653
2339	7612	7593	7564	7577	7646	7606
2340	7613	7592	7565	7576	7647	7607
2340	7613	7594	7565	7578	7647	7607
2341	7614	7595	7566	7579	7648	7608
2339	7612	7593	7564	7577	7646	7606
2337	7614	7595	7566	7579	7648	7608
2643	7621	7571	7569	7549	7693	7591
2642	7620	7570	7568	7548	7692	7590
2641	7619	7569	7567	7547	7691	7589
2640	7618	7568	7566	7546	7690	7588
2642	7620	7570	7568	7548	7692	7590
2641	7619	7569	7567	7547	7691	7589
2672	7600	7552	7544	7530	7668	7572
2644	7622	7572	7570	7550	7694	7592
2640	7708	7618	7672	7607	7767	7650

2459	7693	7699	7625	7679	7743	7717
2462	7696	7702	7628	7682	7746	7720
2460	7694	7700	7626	7680	7744	7718
2459	7691	7697	7623	7677	7741	7715
25519	24989	24920	24986	24938	25041	24889
25518	24988	24919	24985	24937	25040	24888
31211	31017	30969	30986	30927	31009	30941

AZ-TG71543	AZ-TG71539	AZ-TG71555	SEQ895	AZ_TG78596	JEONG-9567	KCJ1232	FSIS
3801	6786	3524	6828	3604	686	3658	957
3674	6738	3460	6793	3527	787	3563	430
3674	6742	3458	6797	3527	789	3563	432
3781	6754	3498	6803	3570	865	3600	469
3762	6747	3458	6802	3539	812	3575	451
3765	6750	3484	6804	3563	809	3599	474
3765	6750	3484	6804	3563	809	3599	474
3724	6775	3499	6830	3580	790	3616	488
3726	6777	3502	6832	3583	792	3620	490
3776	6788	3533	6812	3623	915	3654	567
3742	6794	3563	6816	3638	797	3669	456
3759	6798	3514	6841	3603	845	3635	504
3891	6829	3647	6848	3722	919	3758	651
3891	6829	3647	6848	3722	919	3758	651
3819	6803	3573	6824	3632	880	3668	614
3817	6815	3570	6836	3629	880	3665	611
3734	6739	3455	6787	3520	809	3542	538
3736	6735	3508	6776	3571	859	3593	589
3795	6787	3557	6818	3602	852	3636	565
3836	6797	3550	6839	3633	1056	3653	758
3819	6815	3589	6850	3674	926	3676	667
3716	6748	3443	6783	3564	944	3600	628
3818	6751	3545	6787	3632	801	3664	395
3811	6812	3572	6845	3646	767	3676	395
3738	6735	3471	6786	3525	661	3553	
3718	6787	3545	6818	3583	703	3624	462
3722	6787	3498	6835	3525	645	3551	977
3831	6787	3635	6818	3650	810	3684	1155
3782	6782	3594	6813	3636	477	3658	878
3730	6748	3520	6788	3564	19	3609	658
3730	6748	3520	6788	3564	19	3609	658
3732	6749	3522	6789	3568		3612	661
3740	6778	3529	6829	3577	313	3611	722
3713	6781	3492	6829	3531	628	3557	975
3713	6781	3492	6829	3531	628	3557	975
1958	6679	1824	6706	1874	3495	1820	3491
1952	6669	1815	6696	1865	3486	1811	3509
1946	6674	1817	6701	1855	3495	1793	3507
1952	6657	1849	6684	1852	3420	1790	3446
1911	6665	1803	6692	1857	3447	1795	3496
1933	6661	1821	6688	1867	3481	1809	3519
1932	6660	1820	6687	1864	3480	1808	3518
1952	6673	1783	6709	1853	3487	1795	3503
1978	6691	1817	6722	1881	3502	1821	3478
2000	6659	1831	6675	1911	3491	1863	3509
2067	6677	1904	6697	1968	3448	1923	3446
2018	6791	1774	6816	1953	3547	1920	3542
1963	6686	1908	6705	1739	3441	1705	3408
1958	6681	1901	6700	1734	3436	1700	3403
1964	6687	1909	6706	1740	3442	1706	3409
1958	6681	1903	6700	1734	3436	1700	3403
1963	6686	1908	6705	1739	3441	1705	3408
1989	6718	1918	6752	1515	3569	1533	3515

2017	6824	2057	6866	1788	3640	1747	3610
1883	6745	1932	6768	552	3542	374	3491
1882	6744	1931	6767	551	3541	373	3490
1875	6736	1924	6759	544	3536	366	3485
1877	6739	1926	6762	546	3538	368	3487
1849	6751	1910	6789	473	3603	25	3544
1858	6760	1919	6798	482	3612	34	3553
1858	6760	1919	6798	482	3612		3553
1822	6741	1881	6764	340	3567	188	3516
1847	6756	1916	6779	377	3590	225	3539
1813	6735	1918	6754	383	3560	239	3506
1814	6733	1873	6756	332	3559	180	3508
1823	6741	1941	6766		3568	482	3525
1823	6741	1941	6766		3568	482	3525
1812	6730	1930	6755	27	3557	471	3514
1778	6726	1913	6752	318	3574	337	3521
1869	6802	1805	6822	1514	3542	1430	3499
1900	6830	1836	6850	1545	3573	1461	3530
1871	6804	1807	6824	1516	3544	1432	3501
1869	6802	1805	6822	1514	3542	1430	3499
2307	6833	2198	6867	1915	3698	1929	3663
2309	6835	2200	6869	1917	3701	1931	3666
2303	6829	2194	6863	1911	3695	1925	3660
2300	6826	2191	6860	1908	3692	1922	3657
2295	6837	2193	6871	1912	3699	1926	3633
2300	6830	2189	6864	1906	3692	1920	3655
2302	6828	2193	6862	1910	3694	1924	3659
2311	6839	2200	6873	1899	3701	1913	3666
2302	6828	2193	6862	1910	3694	1924	3659
2301	6827	2192	6861	1909	3693	1923	3658
2322	6828	2141	6860	1928	3670	1938	3659
2291	6813	2238	6847	1969	3689	1989	3662
2308	6832	2228	6866	1912	3694	1932	3659
2308	6832	2228	6866	1912	3694	1932	3659
2308	6832	2228	6866	1912	3694	1932	3659
2262	6807	2120	6832	2063	3691	2055	3685
2191	6807	2020	6832	1978	3673	2012	3671
1959	6701	1901	6708	1761	3585	1799	3543
1958	6705	1887	6712	1745	3581	1783	3527
1958	6705	1887	6712	1745	3581	1783	3527
1956	6701	1874	6708	1729	3582	1767	3516
2016	6787	1904	6829	1847	3559	1861	3533
2285	6814	2180	6831	1973	3589	1990	3563
2413	6799	2279	6816	2117	3639	2120	3613
2478	6805	2322	6822	2166	3628	2175	3606
2478	6805	2322	6822	2166	3628	2175	3606
2485	6820	2329	6833	2161	3620	2140	3595
2436	6813	2289	6830	2133	3606	2142	3594
2425	6804	2297	6821	2080	3649	2112	3633
2426	6779	2382	6796	2051	3718	2058	3703
2385	6874	2372	6891	2145	3683	2173	3653
2382	6885	2384	6902	2150	3673	2194	3653
1883	6746	1849	6766	1809	3543	1791	3539
1884	6766	1864	6786	1820	3546	1802	3555
1894	6761	1775	6778	1737	3572	1707	3519

1898	6764	1779	6781	1739	3572	1709	3519
1920	6762	1788	6779	1757	3564	1725	3519
1957	6782	1867	6801	1763	3553	1743	3505
1998	6787	1831	6804	1849	3552	1797	3538
1998	6789	1831	6806	1849	3552	1797	3538
2009	6803	1850	6828	1806	3590	1747	3536
2011	6805	1852	6830	1808	3592	1749	3538
2011	6805	1852	6830	1808	3592	1749	3538
1995	6810	1863	6835	1831	3605	1772	3554
2009	6798	1845	6823	1813	3583	1754	3539
2043	6812	1871	6839	1821	3565	1766	3513
2044	6813	1872	6840	1822	3566	1767	3514
1864	6783	1771	6795	1725	3575	1685	3536
1865	6781	1761	6793	1719	3571	1679	3548
2017	6748	1879	6766	1878	3550	1896	3515
2019	6748	1881	6766	1880	3550	1896	3515
2009	6740	1871	6758	1867	3542	1885	3507
2008	6739	1870	6757	1869	3541	1887	3506
1994	6730	1884	6742	1889	3529	1907	3530
2008	6739	1870	6757	1869	3541	1887	3506
2009	6740	1871	6758	1870	3542	1888	3507
2023	6754	1885	6772	1884	3556	1902	3521
2010	6753	1909	6771	1910	3534	1928	3508
2027	6768	1912	6807	1920	3570	1953	3532
742	6768	2103	6775	1938	3776	1956	3731
748	6772	2118	6782	1946	3769	1972	3727
758	6785	2121	6792	1954	3788	1972	3747
735	6769	2076	6776	1929	3797	1947	3756
504	6826	1956	6833	1751	3679	1778	3666
486	6826	1955	6835	1742	3696	1769	3651
486	6826	1955	6835	1742	3696	1769	3651
484	6823	1953	6832	1740	3694	1767	3649
492	6834	1969	6841	1754	3702	1781	3663
454	6832	1931	6839	1738	3728	1765	3696
461	6832	1933	6839	1726	3722	1753	3693
602	6827	2023	6827	1788	3785	1815	3742
469	6818	1980	6830	1805	3669	1843	3658
401	6819	1943	6826	1724	3722	1739	3695
437	6841	1980	6861	1729	3751	1756	3717
406	6846	1970	6853	1747	3747	1762	3712
411	6852	1975	6859	1752	3752	1767	3717
396	6827	1978	6837	1755	3721	1778	3706
445	6833	1974	6840	1745	3707	1760	3660
434	6824	1969	6831	1740	3692	1755	3655
248	6807	1831	6814	1683	3699	1708	3674
250	6812	1841	6819	1684	3707	1709	3678
369	6823	1978	6752	1876	3807	1919	3779
378	6832	1987	6761	1885	3816	1928	3788
362	6816	1971	6745	1869	3800	1912	3772
428	6799	1882	6802	1754	3746	1765	3709
238	6803	1841	6810	1691	3709	1716	3684
240	6804	1842	6811	1692	3714	1717	3687
607	6754	2079	6784	1938	3716	1992	3706
193	6791	1943	6808	1812	3709	1861	3699
183	6784	1946	6801	1811	3708	1860	3698

203	6789	1956	6806	1821	3732	1870	3712
202	6800	1959	6817	1824	3727	1873	3711
	6818	1997	6750	1823	3732	1858	3738
172	6830	1908	6837	1760	3746	1785	3713
147	6823	1901	6830	1753	3725	1778	3703
145	6821	1899	6828	1751	3723	1776	3701
239	6807	1851	6814	1701	3701	1726	3666
226	6806	1841	6813	1687	3709	1712	3675
246	6827	1863	6834	1719	3697	1744	3709
237	6820	1856	6827	1710	3690	1735	3702
474	6787	1805	6794	1627	3704	1654	3677
474	6787	1805	6794	1627	3704	1654	3677
247	6810	1877	6817	1732	3687	1757	3683
242	6805	1872	6812	1728	3682	1753	3678
245	6808	1875	6815	1731	3685	1756	3681
1997	6690		6717	1941	3522	1919	3471
1998	6689	45	6716	1942	3523	1920	3473
1985	6678	32	6705	1929	3510	1907	3461
2130	6791	581	6810	2079	3693	2080	3643
1941	6714	390	6733	1934	3536	1931	3515
2265	6952	823	6971	2214	3768	2176	3721
3936	6984	3704	7050	3784	3417	3791	3409
6662	383	6625	241	6660	6600	6692	6597
6664	385	6627	243	6662	6602	6694	6599
6661	382	6624	240	6659	6599	6691	6596
6663	384	6626	242	6661	6601	6693	6598
6705	273	6678	135	6721	6740	6753	6736
6715	262	6671	124	6712	6727	6744	6724
6728	266	6671	128	6714	6737	6746	6734
6736	200	6703	20	6752	6775	6784	6772
6750	216	6717		6766	6789	6798	6786
6729	255	6700	76	6747	6754	6779	6763
6776	258	6745	98	6784	6831	6816	6828
6728	184	6687	46	6736	6755	6768	6752
6758	214	6717	76	6766	6784	6798	6781
6729	191	6670	63	6719	6746	6751	6743
6726	186	6689	50	6738	6761	6770	6758
6734	190	6693	52	6742	6761	6774	6758
6936	431	6848	389	6881	6959	6901	6945
6931	426	6843	384	6876	6954	6896	6940
6884	426	6817	379	6845	6920	6866	6908
6839	198	6694	261	6759	6769	6777	6766
6947	352	6822	401	6863	6900	6885	6879
6825	21	6697	217	6748	6750	6767	6736
6819	15	6691	211	6742	6744	6761	6730
6818		6690	216	6741	6749	6760	6735
6788	122	6664	318	6713	6730	6730	6717
6810	293	6734	489	6755	6780	6778	6765
3732	6905	3474	6944	3618	4169	3543	4140
3721	6895	3463	6934	3607	4158	3532	4129
7679	7889	7518	7898	7560	7501	7573	7490
7679	7889	7518	7898	7560	7501	7573	7490
7678	7888	7517	7897	7559	7500	7572	7489
7677	7887	7516	7896	7558	7499	7571	7488
7679	7889	7518	7898	7560	7501	7573	7490

7677	7889	7516	7898	7558	7499	7571	7488
7679	7889	7518	7898	7560	7501	7573	7490
7677	7887	7516	7896	7558	7499	7571	7488
7680	7890	7519	7899	7561	7502	7574	7491
7677	7887	7516	7896	7558	7499	7571	7488
7676	7886	7515	7895	7557	7498	7570	7487
7677	7887	7516	7896	7558	7499	7571	7488
7678	7888	7517	7897	7559	7500	7572	7489
7678	7888	7517	7897	7559	7500	7572	7489
7751	7926	7659	7940	7694	7579	7695	7564
7755	7930	7661	7944	7698	7583	7699	7568
7755	7930	7663	7944	7698	7583	7699	7568
7758	7933	7666	7947	7701	7586	7702	7571
7711	7936	7618	7946	7649	7517	7654	7498
7752	7927	7660	7941	7695	7580	7696	7565
7741	7921	7651	7935	7686	7573	7687	7558
7739	7920	7649	7934	7684	7571	7685	7556
7744	7924	7654	7938	7689	7576	7690	7561
7739	7919	7649	7933	7684	7571	7685	7556
7741	7921	7651	7935	7686	7572	7687	7557
7746	7924	7656	7938	7690	7578	7691	7563
7617	7866	7553	7888	7549	7473	7541	7470
7696	7864	7562	7869	7596	7475	7594	7474
7696	7860	7558	7861	7594	7473	7592	7472
7822	7948	7699	7966	7722	7672	7716	7641
7822	7948	7699	7966	7722	7672	7716	7641
7827	7955	7704	7973	7727	7679	7721	7648
7865	7993	7733	8011	7764	7727	7754	7698
7821	7932	7712	7949	7742	7662	7731	7633
7652	7688	7546	7712	7613	7525	7619	7518
7655	7691	7549	7715	7616	7528	7622	7521
7660	7699	7556	7723	7621	7541	7627	7528
7667	7676	7553	7700	7611	7530	7621	7503
7633	7719	7523	7743	7592	7518	7598	7497
7641	7727	7531	7751	7600	7526	7606	7505
7632	7718	7522	7742	7591	7517	7597	7496
7708	7725	7597	7749	7654	7581	7658	7558
7710	7727	7599	7751	7656	7583	7660	7560
7714	7731	7603	7756	7660	7587	7664	7564
7727	7744	7616	7768	7673	7600	7677	7577
7630	7744	7524	7748	7610	7558	7613	7536
7631	7745	7525	7749	7611	7559	7614	7535
7631	7745	7525	7749	7611	7559	7614	7537
7632	7746	7526	7750	7612	7560	7615	7538
7630	7744	7524	7748	7610	7558	7613	7536
7632	7746	7526	7750	7612	7560	7615	7538
7677	7774	7563	7785	7604	7545	7615	7515
7676	7773	7562	7784	7603	7544	7614	7514
7675	7772	7561	7783	7602	7543	7613	7513
7674	7771	7560	7782	7601	7542	7612	7512
7676	7773	7562	7784	7603	7544	7614	7514
7675	7772	7561	7783	7602	7543	7613	7513
7652	7799	7538	7810	7581	7526	7592	7496
7678	7775	7564	7786	7605	7546	7616	7516
7746	7794	7640	7808	7700	7613	7710	7589

7737	7956	7629	7968	7688	7669	7698	7633
7740	7961	7632	7973	7691	7672	7701	7636
7738	7959	7630	7971	7689	7670	7699	7634
7735	7954	7627	7966	7686	7667	7696	7631
25021	25019	24942	25033	25028	24927	25017	24900
25020	25018	24941	25032	25027	24926	25016	24899
30994	30790	30923	30811	30936	30912	30939	30910

AZ684313	10B06797	cam_1531_1	la_1424	HICF2	HICF32	HICF112	HICF191	GN02531
7532	7530	7533	7531	6814	3689	3848	3594	7606
7469	7469	7470	7468	6779	3617	3814	3550	7544
7469	7469	7470	7468	6783	3619	3814	3550	7546
7516	7516	7517	7515	6789	3645	3832	3574	7571
7522	7522	7523	7521	6788	3646	3840	3569	7597
7489	7489	7490	7488	6790	3658	3876	3594	7552
7489	7489	7490	7488	6790	3658	3876	3594	7552
7497	7497	7498	7496	6816	3674	3869	3606	7579
7500	7500	7501	7499	6818	3677	3871	3609	7583
7449	7449	7450	7448	6798	3691	3788	3630	7536
7442	7442	7443	7441	6802	3757	3783	3653	7529
7481	7481	7482	7480	6827	3675	3875	3628	7583
7581	7581	7582	7580	6834	3794	3920	3717	7618
7581	7581	7582	7580	6834	3794	3920	3717	7618
7533	7533	7534	7532	6810	3723	3850	3648	7580
7537	7537	7538	7536	6822	3720	3847	3646	7585
7480	7480	7481	7479	6773	3615	3771	3520	7543
7513	7513	7514	7512	6762	3650	3821	3569	7565
7492	7492	7493	7491	6804	3685	3824	3632	7571
7482	7482	7483	7481	6825	3728	3861	3646	7539
7513	7513	7514	7512	6836	3749	3865	3673	7595
7502	7502	7503	7501	6769	3661	3723	3529	7546
7499	7499	7500	7498	6773	3736	3800	3628	7556
7522	7522	7523	7521	6831	3751	3839	3659	7592
7489	7489	7490	7488	6772	3659	3779	3536	7538
7534	7534	7535	7533	6804	3704	3807	3602	7599
7551	7551	7552	7550	6821	3686	3755	3593	7589
7537	7537	7538	7536	6804	3784	3855	3702	7608
7479	7479	7480	7478	6799	3773	3823	3671	7546
7497	7497	7498	7496	6774	3691	3805	3587	7558
7497	7497	7498	7496	6774	3691	3805	3587	7558
7500	7500	7501	7499	6775	3694	3807	3590	7560
7497	7497	7498	7496	6815	3709	3783	3575	7581
7542	7542	7543	7541	6815	3681	3747	3599	7583
7542	7542	7543	7541	6815	3681	3747	3599	7583
7616	7616	7617	7615	6692	2236	2018	1938	7613
7614	7614	7615	7613	6682	2205	2025	1935	7613
7615	7615	7616	7614	6687	2211	2017	1911	7614
7613	7613	7614	7612	6670	2228	2020	1948	7624
7599	7599	7600	7598	6678	2205	1995	1912	7599
7606	7606	7607	7605	6674	2217	2013	1923	7616
7607	7607	7608	7606	6673	2216	2012	1922	7615
7602	7602	7603	7601	6695	2215	2020	1914	7614
7602	7602	7603	7601	6708	2232	2009	1943	7611
7574	7574	7575	7573	6661	2265	1968	1963	7569
7533	7533	7534	7532	6683	2324	2064	2053	7568
7581	7581	7582	7580	6802	2275	2092	1950	7608
7569	7569	7570	7568	6691	2032	2027	1814	7581
7568	7568	7569	7567	6686	2027	2022	1809	7578
7574	7574	7575	7573	6692	2033	2028	1815	7584
7568	7568	7569	7567	6686	2027	2022	1809	7578
7573	7573	7574	7572	6691	2032	2027	1814	7583
7604	7604	7605	7603	6738	2095	2008	1792	7607

7618	7618	7619	7617	6852	2186	2058	1882	7645
7559	7559	7560	7558	6754	1936	1944	1790	7620
7559	7559	7560	7558	6753	1935	1943	1789	7619
7554	7554	7555	7553	6745	1928	1936	1784	7614
7556	7556	7557	7555	6748	1930	1938	1786	7616
7563	7563	7564	7562	6775	1923	1910	1738	7606
7572	7572	7573	7571	6784	1932	1919	1747	7615
7572	7572	7573	7571	6784	1932	1919	1747	7615
7528	7528	7529	7527	6750	1870	1883	1735	7586
7540	7540	7541	7539	6765	1887	1906	1756	7589
7515	7515	7516	7514	6740	1900	1874	1759	7569
7520	7520	7521	7519	6742	1862	1875	1727	7578
7559	7559	7560	7558	6752	1912	1876	1806	7612
7559	7559	7560	7558	6752	1912	1876	1806	7612
7549	7549	7550	7548	6741	1901	1865	1795	7602
7568	7568	7569	7567	6738	1927	1843	1788	7607
7541	7541	7542	7540	6808	2139	1948	1829	7580
7570	7570	7571	7569	6836	2170	1979	1860	7608
7543	7543	7544	7542	6810	2141	1950	1831	7582
7541	7541	7542	7540	6808	2139	1948	1829	7580
7580	7580	7581	7579	6853	127	2395	2087	7637
7582	7582	7583	7581	6855	129	2397	2089	7641
7576	7576	7577	7575	6849	123	2391	2083	7635
7573	7573	7574	7572	6846	120	2388	2080	7632
7584	7584	7585	7583	6857	164	2370	2090	7641
7575	7575	7576	7574	6850	128	2386	2078	7632
7575	7575	7576	7574	6848	122	2390	2082	7634
7580	7580	7581	7579	6859	137	2383	2097	7635
7575	7575	7576	7574	6848	122	2390	2082	7634
7574	7574	7575	7573	6847	121	2389	2081	7633
7571	7571	7572	7570	6846	194	2374	2092	7624
7584	7584	7585	7583	6833	203	2365	2149	7645
7567	7567	7568	7566	6852	6	2396	2090	7644
7567	7567	7568	7566	6852	6	2396	2090	7644
7567	7567	7568	7566	6852		2396	2090	7644
7656	7656	7657	7655	6818	1685	2291	2120	7698
7647	7647	7648	7646	6818	1634	2224	2028	7690
7578	7578	7579	7577	6694	1905	2000	2057	7590
7579	7579	7580	7578	6698	1885	2002	2041	7589
7579	7579	7580	7578	6698	1885	2002	2041	7589
7577	7577	7578	7576	6694	1885	1992	2045	7586
7550	7550	7551	7549	6815	1973	2076	1959	7617
7658	7658	7659	7657	6817	1946	2314	1952	7653
7695	7695	7696	7694	6800	2008	2442	2072	7699
7702	7702	7703	7701	6806	1985	2517	2103	7717
7702	7702	7703	7701	6806	1985	2517	2103	7717
7704	7704	7705	7703	6817	2066	2498	2134	7714
7708	7708	7709	7707	6814	2020	2466	2114	7719
7674	7674	7675	7673	6805	1918	2456	2117	7692
7671	7671	7672	7670	6780	1921	2457	2172	7706
7699	7699	7700	7698	6875	1870	2460	2220	7767
7698	7698	7697	7697	6886	1913	2453	2255	7760
7530	7530	7531	7529	6752	2100	1950	558	7570
7551	7551	7552	7550	6772	2120	1975	571	7588
7556	7556	7557	7555	6764	2046	1953	473	7576

7558	7558	7559	7557	6767	2048	1955	475	7578
7561	7561	7562	7560	6765	2080	1977	437	7586
7530	7530	7531	7529	6787	2094	2008	461	7566
7558	7558	7559	7557	6790	2137	2066	332	7584
7560	7560	7561	7559	6792	2137	2066	332	7586
7554	7554	7555	7553	6814	2090	2052		7605
7556	7556	7557	7555	6816	2092	2054	2	7607
7554	7554	7555	7553	6816	2092	2054	2	7605
7540	7540	7541	7539	6821	2113	2061	38	7601
7552	7552	7553	7551	6809	2087	2051	19	7605
7540	7540	7541	7539	6825	2081	2081	115	7587
7541	7541	7542	7540	6826	2082	2082	116	7588
7567	7567	7568	7566	6781	2083	1920	641	7591
7568	7568	7569	7567	6779	2073	1916	639	7592
7612	7612	7613	7611	6752	2064	2051	1918	7645
7614	7614	7615	7613	6752	2062	2053	1918	7647
7604	7604	7605	7603	6744	2055	2043	1910	7637
7603	7603	7604	7602	6743	2055	2042	1909	7636
7612	7612	7613	7611	6728	2083	2052	1937	7647
7603	7603	7604	7602	6743	2055	2042	1911	7636
7604	7604	7605	7603	6744	2056	2043	1910	7637
7616	7616	7617	7615	6758	2070	2057	1924	7651
7593	7593	7594	7592	6757	2102	2049	1953	7634
7651	7651	7652	7650	6793	2103	2046	1959	7635
7739	7739	7740	7738	6761	2372	769	2139	7681
7742	7742	7743	7741	6768	2386	785	2147	7688
7754	7754	7755	7753	6778	2390	787	2155	7696
7728	7728	7729	7727	6762	2353	768	2100	7672
7725	7725	7726	7724	6819	2229	529	1995	7681
7719	7719	7720	7718	6821	2236	498	1990	7677
7719	7719	7720	7718	6821	2236	498	1990	7677
7716	7716	7717	7715	6818	2234	496	1988	7674
7719	7719	7720	7718	6827	2244	510	1994	7681
7693	7693	7694	7692	6825	2220	514	1953	7662
7724	7724	7725	7723	6825	2210	520	1966	7685
7691	7691	7692	7690	6813	2290	659	2041	7646
7680	7680	7681	7679	6816	2261	444	1995	7664
7719	7719	7720	7718	6812	2206	450	1940	7687
7705	7705	7706	7704	6847	2229	526	1947	7692
7724	7724	7725	7723	6839	2231	485	1963	7694
7728	7728	7729	7727	6845	2236	490	1968	7696
7693	7693	7694	7692	6823	2223	489	1949	7673
7723	7723	7724	7722	6826	2233	453	1963	7685
7712	7712	7713	7711	6817	2230	442	1958	7676
7679	7679	7680	7678	6800	2187	299	1879	7649
7682	7682	7683	7681	6805	2198	308	1873	7652
7683	7683	7684	7682	6738	2396		2052	7609
7692	7692	7693	7691	6747	2405	19	2061	7618
7676	7676	7677	7675	6731	2389	13	2045	7602
7686	7686	7687	7685	6788	2258	355	1902	7645
7675	7675	7676	7674	6796	2208	314	1881	7648
7678	7678	7679	7677	6797	2208	314	1882	7651
7563	7563	7564	7562	6770	2464	624	2110	7554
7682	7682	7683	7681	6794	2319	386	1994	7640
7672	7672	7673	7671	6787	2324	389	1991	7628

7681	7681	7682	7680	6792	2335	407	2001	7639
7688	7688	7689	7687	6803	2338	402	2004	7644
7678	7678	7679	7677	6736	2308	369	2009	7632
7703	7703	7704	7702	6823	2277	398	1964	7656
7693	7693	7694	7692	6816	2270	391	1954	7644
7691	7691	7692	7690	6814	2268	389	1952	7642
7674	7674	7675	7673	6800	2191	319	1889	7645
7654	7654	7655	7653	6799	2202	305	1882	7634
7699	7699	7700	7698	6820	2204	341	1914	7669
7692	7692	7693	7691	6813	2197	332	1907	7662
7662	7662	7663	7661	6780	2109	531	1804	7635
7662	7662	7663	7661	6780	2109	531	1804	7635
7677	7677	7678	7676	6803	2249	323	1924	7650
7672	7672	7673	7671	6798	2244	318	1919	7645
7675	7675	7676	7674	6801	2247	321	1922	7648
7517	7517	7518	7516	6703	2228	1978	1850	7526
7514	7514	7515	7513	6702	2229	1979	1849	7523
7504	7504	7505	7503	6691	2216	1966	1838	7513
7600	7600	7601	7599	6796	2374	2117	2041	7575
7529	7529	7530	7528	6719	2214	1960	1876	7529
7753	7753	7754	7752	6957	2473	2278	2027	7768
7768	7768	7767	7767	7036	3783	3965	3800	7755
7905	7905	7906	7904	225	6760	6664	6726	7768
7907	7907	7908	7906	227	6762	6666	6728	7770
7904	7904	7905	7903	224	6759	6663	6725	7767
7906	7906	7907	7905	226	6761	6665	6727	7769
7874	7874	7875	7873	119	6823	6735	6792	7741
7892	7892	7893	7891	108	6812	6724	6780	7747
7903	7903	7904	7902	112	6816	6718	6788	7756
7882	7882	7883	7881		6852	6738	6814	7735
7897	7897	7898	7896	20	6866	6752	6828	7750
7886	7886	7887	7885	60	6842	6713	6809	7739
7899	7899	7900	7898	82	6880	6778	6840	7752
7875	7875	7876	7874	30	6838	6730	6806	7726
7904	7904	7905	7903	60	6868	6760	6836	7755
7868	7868	7869	7867	47	6819	6731	6793	7717
7875	7875	7876	7874	34	6834	6728	6810	7728
7879	7879	7880	7878	36	6844	6736	6812	7732
8010	8010	8011	8009	373	6970	6889	6945	7861
8005	8005	8006	8004	368	6965	6884	6940	7856
7968	7968	7969	7967	363	6936	6848	6904	7834
7904	7904	7905	7903	245	6842	6837	6831	7765
8007	8007	8008	8006	385	6955	6942	6935	7871
7893	7893	7894	7892	201	6841	6830	6810	7751
7887	7887	7888	7886	195	6835	6824	6804	7745
7888	7888	7889	7887	200	6832	6823	6803	7746
7859	7859	7860	7858	302	6808	6792	6775	7704
7766	7766	7767	7765	473	6854	6849	6818	7653
7791	7791	7790	7790	6930	3790	3747	3289	7772
7780	7780	7779	7779	6920	3779	3736	3278	7761
6	6	5	5	7883	7568	7684	7555	3426
6	6	5	5	7883	7568	7684	7555	3426
5	5	4	4	7882	7567	7683	7554	3425
5	5	4	4	7881	7566	7682	7553	3425
5	5		4	7883	7568	7684	7555	3425

5	5	6	4	7883	7566	7682	7553	3425
5	5	6	2	7883	7568	7684	7555	3425
3	3	4		7881	7566	7682	7553	3423
6	6	7	5	7884	7569	7685	7556	3426
3	3	4	2	7881	7566	7682	7553	3423
2	2	3	1	7880	7565	7681	7552	3422
3	3	4	2	7881	7566	7682	7553	3423
	4	5	3	7882	7567	7683	7554	3424
4		5	3	7882	7567	7683	7554	3424
284	284	285	283	7925	7705	7750	7696	3289
288	288	289	287	7929	7709	7754	7700	3293
288	288	289	287	7929	7709	7754	7700	3293
291	291	292	290	7932	7712	7757	7703	3296
383	383	384	382	7932	7666	7720	7651	3359
285	285	286	284	7926	7706	7751	7697	3290
261	261	262	260	7920	7699	7740	7688	3302
259	259	260	258	7919	7697	7738	7686	3300
263	263	264	262	7923	7702	7743	7691	3302
258	258	259	257	7918	7697	7738	7686	3297
258	258	259	257	7920	7699	7740	7688	3301
268	268	269	267	7923	7703	7745	7692	3301
322	322	323	321	7873	7556	7696	7571	3381
813	813	814	812	7854	7614	7702	7600	3442
878	878	879	877	7846	7618	7702	7610	3502
2044	2044	2045	2043	7949	7748	7817	7744	3464
2044	2044	2045	2043	7949	7748	7817	7744	3464
2051	2051	2052	2050	7956	7753	7822	7749	3471
2065	2065	2066	2064	7994	7783	7864	7787	3473
2146	2146	2147	2145	7932	7771	7826	7767	3441
3408	3408	3409	3407	7695	7611	7632	7620	2337
3411	3411	3412	3410	7698	7614	7635	7623	2340
3415	3415	3416	3414	7706	7623	7640	7630	2342
3432	3432	3433	3431	7683	7618	7643	7622	2380
3363	3363	3364	3362	7726	7597	7611	7594	2302
3371	3371	3372	3370	7734	7605	7619	7602	2310
3362	3362	3363	3361	7725	7596	7610	7593	2301
3414	3414	3415	3413	7732	7660	7684	7668	2303
3416	3416	3417	3415	7734	7662	7686	7670	2305
3421	3421	3422	3420	7739	7666	7690	7674	2310
3433	3433	3434	3432	7751	7679	7703	7687	2322
3424	3424	3425	3423	7733	7642	7607	7603	39
3425	3425	3426	3424	7734	7643	7608	7604	40
3425	3425	3426	3424	7734	7643	7608	7604	40
3426	3426	3427	3425	7735	7644	7609	7605	41
3424	3424	3425	3423	7733	7642	7607	7603	39
3424	3424	3425	3423	7735	7644	7609	7605	
3500	3500	3501	3499	7768	7637	7668	7613	2625
3499	3499	3500	3498	7767	7636	7667	7612	2624
3498	3498	3499	3497	7766	7635	7666	7611	2623
3497	3497	3498	3496	7765	7634	7665	7610	2622
3499	3499	3500	3498	7767	7636	7667	7612	2624
3498	3498	3499	3497	7766	7635	7666	7611	2623
3530	3530	3531	3529	7793	7612	7643	7588	2652
3501	3501	3502	3500	7769	7638	7669	7614	2626
3523	3523	3524	3522	7793	7707	7769	7700	2558

3411	3411	3412	3410	7951	7681	7744	7656	2516
3414	3414	3415	3413	7956	7684	7747	7659	2519
3412	3412	3413	3411	7954	7682	7745	7657	2517
3409	3409	3410	3408	7949	7679	7742	7654	2517
25533	25533	25534	25532	25019	24998	25036	24976	25505
25532	25532	25533	25531	25018	24997	25035	24975	25504
31137	31137	31138	31136	30797	30969	31008	31007	31227

GN03624	KCJ3858	CFSAN045100	AZ-TG-WCHI-3	IEH-NGS-ECO-00205	F283	HS115	J21
3779	3583	3589	6772	841	7532	7531	7515
3685	3533	3498	6732	191	7469	7468	7444
3685	3533	3498	6736	193	7469	7468	7444
3740	3559	3537	6747	374	7516	7515	7522
3717	3560	3510	6744	250	7522	7521	7497
3748	3583	3534	6744	195	7489	7488	7464
3748	3583	3534	6744	195	7489	7488	7464
3738	3589	3551	6767	163	7497	7496	7472
3740	3592	3554	6769	165	7500	7499	7476
3780	3611	3587	6754	304	7449	7448	7520
3786	3632	3604	6757	412	7442	7441	7513
3776	3609	3570	6780		7481	7480	7485
3865	3702	3693	6784	623	7581	7580	7606
3865	3702	3693	6784	623	7581	7580	7606
3795	3631	3603	6760	475	7533	7532	7558
3792	3628	3600	6772	474	7537	7536	7562
3711	3497	3477	6731	385	7480	7479	7451
3730	3548	3528	6718	458	7513	7512	7489
3802	3607	3571	6757	404	7492	7491	7487
3743	3613	3588	6782	538	7482	7481	7490
3825	3650	3611	6785	477	7513	7512	7522
3682	3511	3533	6719	745	7502	7501	7505
3776	3619	3601	6725	570	7499	7498	7534
3798	3644	3613	6785	499	7522	7521	7567
3711	3519	3490	6724	504	7489	7488	7470
3710	3586	3554	6758	577	7534	7533	7513
3684	3535	3472	6777	1170	7551	7550	7530
3763	3658	3599	6766	1314	7537	7536	7550
3784	3633	3585	6750	984	7479	7478	7477
3725	3561	3538	6726	842	7497	7496	7470
3725	3561	3538	6726	842	7497	7496	7470
3727	3564	3541	6727	845	7500	7499	7473
3722	3532	3540	6773	886	7497	7496	7497
3680	3535	3480	6771	1157	7542	7541	7521
3680	3535	3480	6771	1157	7542	7541	7521
1952	1877	1856	6658	3584	7616	7615	7581
1980	1874	1847	6654	3572	7614	7613	7579
1966	1862	1829	6651	3578	7615	7614	7580
1975	1891	1826	6636	3541	7613	7612	7579
1936	1855	1831	6644	3551	7599	7598	7564
1946	1876	1845	6640	3571	7606	7605	7575
1945	1875	1844	6639	3570	7607	7606	7576
1977	1869	1829	6661	3548	7602	7601	7565
1980	1888	1857	6674	3537	7602	7601	7588
1954	1912	1899	6627	3545	7574	7573	7602
2029	2004	1973	6651	3533	7533	7532	7579
2060	1910	1947	6766	3617	7581	7580	7556
1967	1763	1742	6657	3440	7569	7568	7503
1962	1758	1737	6652	3435	7568	7567	7502
1968	1764	1743	6658	3441	7574	7573	7508
1962	1758	1737	6652	3435	7568	7567	7502
1967	1763	1742	6657	3440	7573	7572	7507
1982	1696	1603	6694	3599	7604	7603	7570

2001	1768	1750	6814	3646	7618	7617	7622
1898	1762	9	6714	3571	7559	7558	7546
1897	1761		6713	3570	7559	7558	7546
1890	1756	21	6705	3565	7554	7553	7541
1892	1758	23	6708	3567	7556	7555	7543
1864	1716	364	6735	3626	7563	7562	7532
1873	1725	373	6744	3635	7572	7571	7541
1873	1725	373	6744	3635	7572	7571	7541
1837	1717	261	6710	3598	7528	7527	7515
1862	1738	296	6725	3621	7540	7539	7527
1832	1753	314	6700	3571	7515	7514	7500
1829	1709	253	6702	3590	7520	7519	7507
1824	1757	551	6712	3603	7559	7558	7549
1824	1757	551	6712	3603	7559	7558	7549
1813	1746	540	6701	3592	7549	7548	7539
1793	1763	453	6698	3603	7568	7567	7523
1888	1802	1472	6770	3595	7541	7540	7528
1919	1833	1503	6798	3626	7570	7569	7557
1890	1804	1474	6772	3597	7543	7542	7530
1888	1802	1472	6770	3595	7541	7540	7528
2337	2085	1934	6813	3741	7580	7579	7569
2339	2087	1936	6815	3744	7582	7581	7571
2333	2081	1930	6809	3738	7576	7575	7565
2330	2078	1927	6806	3735	7573	7572	7562
2320	2088	1931	6817	3746	7584	7583	7573
2328	2076	1925	6810	3735	7575	7574	7564
2332	2080	1929	6808	3737	7575	7574	7564
2341	2095	1918	6819	3744	7580	7579	7569
2332	2080	1929	6808	3737	7575	7574	7564
2331	2079	1928	6807	3736	7574	7573	7563
2331	2090	1943	6806	3725	7571	7570	7580
2323	2141	1990	6793	3730	7584	7583	7573
2338	2080	1935	6812	3675	7567	7566	7556
2338	2080	1935	6812	3675	7567	7566	7556
2338	2080	1935	6812	3675	7567	7566	7556
2246	2107	2079	6790	3758	7656	7655	7652
2183	2023	2012	6782	3762	7647	7646	7635
1986	1979	1787	6650	3588	7578	7577	7546
1980	1969	1771	6650	3587	7579	7578	7547
1980	1969	1771	6650	3587	7579	7578	7547
1972	1965	1749	6646	3588	7577	7576	7545
2027	1854	1845	6767	3580	7550	7549	7550
2281	1853	2025	6775	3657	7658	7657	7632
2409	1973	2153	6760	3705	7695	7694	7669
2478	2006	2200	6764	3700	7702	7701	7676
2478	2006	2200	6764	3700	7702	7701	7676
2469	2019	2184	6787	3688	7704	7703	7682
2433	1999	2156	6786	3678	7708	7707	7682
2425	2029	2113	6767	3719	7674	7673	7648
2426	2114	2065	6736	3792	7671	7670	7645
2410	2208	2143	6835	3720	7699	7698	7690
2407	2241	2156	6846	3720	7698	7697	7689
1872	181	1821	6728	3598	7530	7529	7569
1885	190	1832	6746	3600	7551	7550	7590
1893	74	1737	6738	3609	7556	7555	7567

1895	76	1739	6741	3609	7558	7557	7571
1917		1761	6739	3609	7561	7560	7574
1952	376	1781	6755	3605	7530	7529	7540
2032	387	1847	6758	3601	7558	7557	7571
2032	387	1847	6760	3601	7560	7559	7573
2004	437	1789	6780	3628	7554	7553	7571
2006	439	1791	6782	3630	7556	7555	7573
2006	439	1791	6782	3630	7554	7553	7571
2016	451	1814	6790	3617	7540	7539	7557
2007	442	1796	6777	3628	7552	7551	7569
2035	494	1808	6797	3608	7540	7539	7557
2036	495	1809	6798	3609	7541	7540	7558
1886	711	1697	6739	3633	7567	7566	7558
1892	707	1691	6743	3634	7568	7567	7559
1994	1816	1894	6708	3586	7612	7611	7602
1996	1818	1894	6708	3586	7614	7613	7604
1986	1810	1883	6700	3578	7604	7603	7594
1985	1809	1885	6699	3577	7603	7602	7593
1973	1837	1905	6684	3585	7612	7611	7590
1985	1811	1885	6699	3577	7603	7602	7593
1986	1810	1886	6700	3578	7604	7603	7594
2000	1824	1900	6714	3592	7616	7615	7606
2017	1849	1926	6710	3546	7593	7592	7583
2006	1861	1944	6749	3610	7651	7650	7575
751	2051	1993	6745	3838	7739	7738	7662
757	2071	2009	6752	3845	7742	7741	7667
769	2067	2009	6760	3854	7754	7753	7677
746	2030	1984	6744	3863	7728	7727	7651
515	1894	1822	6803	3757	7725	7724	7648
492	1889	1813	6801	3760	7719	7718	7642
492	1889	1813	6801	3760	7719	7718	7642
490	1887	1811	6798	3758	7716	7715	7639
498	1893	1825	6807	3758	7719	7718	7642
487	1870	1809	6806	3756	7693	7692	7616
470	1865	1797	6811	3784	7724	7723	7647
610	1952	1859	6826	3846	7691	7690	7614
438	1922	1888	6802	3741	7680	7679	7685
412	1867	1783	6798	3790	7719	7718	7644
454	1898	1798	6827	3792	7705	7704	7645
429	1890	1806	6819	3792	7724	7723	7649
434	1895	1811	6825	3797	7728	7727	7653
439	1888	1822	6803	3768	7693	7692	7624
449	1890	1804	6806	3769	7723	7722	7648
440	1885	1799	6795	3758	7712	7711	7637
263	1794	1732	6782	3777	7679	7678	7596
264	1794	1733	6785	3778	7682	7681	7599
402	1977	1943	6724	3875	7683	7682	7696
411	1986	1952	6733	3884	7692	7691	7705
395	1970	1936	6717	3868	7676	7675	7689
435	1829	1807	6763	3801	7686	7685	7624
258	1794	1740	6780	3770	7675	7674	7592
260	1795	1741	6781	3773	7678	7677	7595
595	2023	2019	6786	3801	7563	7562	7556
42	1909	1885	6780	3767	7682	7681	7678
19	1904	1884	6771	3757	7672	7671	7668

37	1914	1894	6776	3770	7681	7680	7677
	1917	1897	6787	3776	7688	7687	7684
202	1920	1882	6715	3759	7678	7677	7617
176	1859	1809	6807	3787	7703	7702	7620
151	1849	1802	6800	3768	7693	7692	7610
149	1847	1800	6798	3766	7691	7690	7608
260	1802	1750	6780	3774	7674	7673	7591
263	1795	1736	6780	3749	7654	7653	7572
289	1825	1768	6806	3763	7699	7698	7616
280	1818	1759	6799	3756	7692	7691	7609
493	1688	1674	6754	3774	7662	7661	7579
493	1688	1674	6754	3774	7662	7661	7579
261	1833	1781	6781	3760	7677	7676	7594
256	1828	1777	6776	3755	7672	7671	7589
259	1831	1780	6779	3758	7675	7674	7592
1959	1788	1931	6671	3514	7517	7516	7553
1960	1789	1932	6670	3516	7514	7513	7550
1947	1776	1919	6659	3504	7504	7503	7540
2098	2015	2065	6758	3682	7600	7599	7636
1921	1824	1937	6687	3541	7529	7528	7565
2229	2015	2208	6923	3752	7753	7752	7789
3888	3838	3786	6992	3396	7768	7767	7797
6729	6675	6653	211	6652	7905	7904	7896
6731	6677	6655	213	6654	7907	7906	7898
6728	6674	6652	210	6651	7904	7903	7895
6730	6676	6654	212	6653	7906	7905	7897
6797	6745	6722	26	6769	7874	7873	7865
6787	6739	6713		6780	7892	7891	7883
6797	6741	6715	22	6793	7903	7902	7894
6803	6765	6753	108	6827	7882	7881	7873
6817	6779	6767	124	6841	7897	7896	7888
6782	6760	6748	162	6832	7886	7885	7877
6843	6791	6785	150	6883	7899	7898	7890
6795	6757	6737	94	6807	7875	7874	7866
6825	6787	6767	124	6836	7904	7903	7895
6796	6744	6720	87	6798	7868	7867	7859
6793	6761	6739	94	6813	7875	7874	7866
6801	6763	6743	100	6813	7879	7878	7870
6995	6894	6884	431	6995	8010	8009	7994
6990	6889	6879	426	6990	8005	8004	7989
6933	6861	6848	431	6948	7968	7967	7980
6832	6782	6760	279	6803	7904	7903	7898
6938	6884	6868	449	6916	8007	8006	8003
6807	6769	6751	265	6799	7893	7892	7871
6801	6763	6745	259	6793	7887	7886	7865
6800	6762	6744	262	6798	7888	7887	7866
6769	6734	6714	368	6795	7859	7858	7837
6823	6779	6762	524	6800	7766	7765	7744
3680	3371	3534	6958	4226	7791	7790	7797
3669	3360	3523	6948	4215	7780	7779	7786
7689	7562	7560	7893	7482	3	5	324
7689	7562	7560	7893	7482	3	5	324
7688	7561	7559	7892	7481		4	323
7687	7560	7558	7891	7480	2	4	323
7689	7562	7560	7893	7482	4	4	323

7687	7560	7558	7893	7480	6	4	323
7689	7562	7560	7893	7482	6	4	323
7687	7560	7558	7891	7480	4	2	321
7690	7563	7561	7894	7483	7	5	324
7687	7560	7558	7891	7480	4		321
7686	7559	7557	7890	7479	3	1	320
7687	7560	7558	7891	7480	4	2	321
7688	7561	7559	7892	7481	5	3	322
7688	7561	7559	7892	7481	5	3	322
7777	7699	7698	7936	7594	285	283	262
7781	7703	7702	7940	7598	289	287	266
7781	7703	7702	7940	7598	289	287	266
7784	7706	7705	7943	7601	292	290	269
7750	7656	7655	7938	7527	384	382	349
7778	7700	7699	7937	7595	286	284	263
7767	7691	7690	7930	7588	262	260	239
7765	7689	7688	7929	7586	260	258	237
7770	7694	7693	7933	7591	264	262	241
7765	7689	7688	7928	7586	259	257	236
7767	7691	7690	7930	7587	259	257	236
7772	7696	7694	7933	7593	269	267	246
7684	7574	7546	7883	7485	323	321	
7698	7597	7598	7855	7499	814	812	720
7698	7607	7596	7841	7497	879	877	785
7842	7744	7720	7958	7671	2045	2043	2043
7842	7744	7720	7958	7671	2045	2043	2043
7847	7749	7725	7965	7678	2052	2050	2050
7879	7783	7759	8004	7728	2066	2064	2066
7845	7764	7739	7938	7670	2146	2145	2128
7665	7617	7613	7709	7562	3409	3407	3365
7668	7620	7616	7712	7565	3412	3410	3368
7673	7627	7621	7717	7572	3416	3414	3372
7680	7620	7617	7694	7547	3433	3431	3393
7646	7591	7587	7737	7535	3364	3362	3320
7654	7599	7595	7745	7543	3372	3370	3328
7645	7590	7586	7736	7534	3363	3361	3319
7719	7665	7658	7742	7606	3415	3413	3371
7721	7667	7660	7744	7608	3417	3415	3373
7725	7671	7664	7749	7612	3422	3420	3378
7738	7684	7677	7761	7625	3434	3432	3390
7642	7584	7617	7745	7581	3425	3423	3381
7643	7585	7618	7746	7580	3426	3424	3382
7643	7585	7618	7746	7582	3426	3424	3382
7644	7586	7619	7747	7583	3427	3425	3383
7642	7584	7617	7745	7581	3425	3423	3381
7644	7586	7619	7747	7583	3425	3423	3381
7679	7595	7625	7779	7549	3501	3499	3462
7678	7594	7624	7778	7548	3500	3498	3461
7677	7593	7623	7777	7547	3499	3497	3460
7676	7592	7622	7776	7546	3498	3496	3459
7678	7594	7624	7778	7548	3500	3498	3461
7677	7593	7623	7777	7547	3499	3497	3460
7654	7570	7602	7760	7530	3531	3529	3492
7680	7596	7626	7780	7550	3502	3500	3463
7760	7706	7701	7804	7615	3524	3522	3493

7728	7661	7699	7965	7671	3412	3410	3374
7731	7664	7702	7970	7674	3415	3413	3377
7729	7662	7700	7968	7672	3413	3411	3375
7726	7659	7697	7963	7669	3410	3408	3372
25034	24991	25020	25033	24930	25533	25532	25544
25033	24990	25019	25032	24929	25532	25531	25543
31009	31010	30944	30799	30917	31137	31136	31134

KO178B	KO198B	AZ-TG-WCHI-8	AZ-TG-713-2	KCJ9492	MOD1-EC5105	MOD1-EC5111
7533	7533	3762	3841	3830	3547	3520
7470	7470	3672	3807	3776	3478	3455
7470	7470	3674	3807	3776	3480	3455
7517	7517	3712	3825	3815	3522	3513
7523	7523	3713	3833	3794	3505	3492
7490	7490	3725	3869	3830	3529	3507
7490	7490	3725	3869	3830	3529	3507
7498	7498	3727	3862	3833	3531	3502
7501	7501	3730	3864	3835	3533	3504
7450	7450	3748	3781	3875	3585	3556
7443	7443	3746	3776	3876	3606	3581
7482	7482	3730	3868	3863	3572	3551
7582	7582	3832	3913	3951	3655	3636
7582	7582	3832	3913	3951	3655	3636
7534	7534	3770	3843	3881	3584	3565
7538	7538	3767	3840	3878	3580	3561
7481	7481	3662	3764	3761	3465	3456
7514	7514	3699	3814	3810	3499	3484
7493	7493	3736	3817	3858	3567	3557
7483	7483	3779	3854	3903	3624	3606
7514	7514	3792	3858	3901	3616	3607
7503	7503	3710	3716	3749	3497	3480
7500	7500	3737	3793	3851	3595	3593
7523	7523	3756	3832	3881	3613	3617
7490	7490	3662	3772	3756	3509	3496
7535	7535	3705	3800	3796	3544	3517
7552	7552	3685	3748	3737	3468	3461
7538	7538	3779	3848	3860	3612	3589
7480	7480	3764	3816	3856	3583	3564
7498	7498	3686	3798	3795	3484	3445
7498	7498	3686	3798	3795	3484	3445
7501	7501	3689	3800	3797	3486	3447
7498	7498	3704	3776	3767	3507	3479
7543	7543	3678	3740	3727	3468	3449
7543	7543	3678	3740	3727	3468	3449
7617	7617	2233	2011	2126	75	107
7615	7615	2212	2018	2131		94
7616	7616	2218	2010	2119	108	92
7614	7614	2203	2013	2130	177	151
7600	7600	2206	1988	2105	94	
7607	7607	2218	2006	2131	130	104
7608	7608	2217	2005	2130	129	103
7603	7603	2216	2013	2110	172	146
7603	7603	2233	2002	2135	180	164
7575	7575	2266	1961	2175	234	208
7534	7534	2327	2057	2247	440	424
7582	7582	2240	2085	2128	1778	1769
7570	7570	2173	2020	2037	1785	1771
7569	7569	2168	2015	2032	1780	1766
7575	7575	2174	2021	2038	1786	1772
7569	7569	2168	2015	2032	1780	1766
7574	7574	2173	2020	2037	1785	1771
7605	7605	2096	2001	1982	1769	1759

7619	7619	2287	2051	2116	1984	1968
7560	7560	1991	1937	1985	1848	1832
7560	7560	1990	1936	1984	1847	1831
7555	7555	1983	1929	1977	1840	1824
7557	7557	1985	1931	1979	1842	1826
7564	7564	1980	1903	1938	1802	1786
7573	7573	1989	1912	1947	1811	1795
7573	7573	1989	1912	1947	1811	1795
7529	7529	1927	1876	1918	1803	1787
7541	7541	1944	1899	1943	1834	1818
7516	7516	1957	1867	1943	1804	1788
7521	7521	1919	1868	1910	1795	1779
7560	7560	1969	1869	1929	1865	1857
7560	7560	1969	1869	1929	1865	1857
7550	7550	1958	1858	1916	1854	1846
7569	7569	1984	1836	1886	1821	1805
7542	7542	2074	1941	1966	1855	1840
7571	7571	2105	1972	1997	1886	1871
7544	7544	2076	1943	1968	1857	1842
7542	7542	2074	1941	1966	1855	1840
7581	7581	98	2388	2352	2190	2190
7583	7583	100	2390	2354	2192	2192
7577	7577	94	2384	2348	2186	2186
7574	7574	91	2381	2345	2183	2183
7585	7585	135	2363	2327	2193	2181
7576	7576	99	2379	2343	2181	2181
7576	7576	93	2383	2347	2185	2185
7581	7581	108	2376	2362	2194	2194
7576	7576	93	2383	2347	2185	2185
7575	7575	92	2382	2346	2184	2184
7572	7572	165	2367	2357	2197	2197
7585	7585		2358	2352	2212	2206
7568	7568	203	2389	2353	2205	2205
7568	7568	203	2389	2353	2205	2205
7568	7568	203	2389	2353	2205	2205
7657	7657	1629	2284	2292	2265	2267
7648	7648	1551	2217	2215	2227	2229
7579	7579	1900	1993	1970	1961	1941
7580	7580	1902	1995	1952	1945	1933
7580	7580	1902	1995	1952	1945	1933
7578	7578	1902	1985	1944	1948	1930
7551	7551	2029	2069	2123	2021	2033
7659	7659	1864	2307	2312	2229	2231
7696	7696	1985	2435	2430	2310	2312
7703	7703	1968	2510	2433	2363	2363
7703	7703	1968	2510	2433	2363	2363
7705	7705	2045	2491	2474	2358	2352
7709	7709	1999	2459	2440	2307	2309
7675	7675	1901	2449	2414	2352	2352
7672	7672	1935	2450	2429	2357	2351
7700	7700	1864	2453	2470	2427	2417
7699	7699	1917	2446	2449	2443	2439
7531	7531	2151	1943	2075	1861	1838
7552	7552	2181	1968	2092	1871	1847
7557	7557	2107	1946	2018	1839	1820

7559	7559	2109	1948	2022	1843	1824
7562	7562	2141	1970	2030	1874	1855
7531	7531	2153	2001	2054	1959	1940
7559	7559	2204	2059	2088	1889	1865
7561	7561	2204	2059	2088	1889	1865
7555	7555	2149	2045	2100	1935	1912
7557	7557	2151	2047	2102	1937	1914
7555	7555	2151	2047	2102	1937	1914
7541	7541	2158	2054	2123	1950	1929
7553	7553	2148	2044	2101	1928	1901
7541	7541	2144	2074	2103	1956	1933
7542	7542	2145	2075	2104	1957	1934
7568	7568	2080	1913	1953	1860	1836
7569	7569	2076	1909	1949	1850	1826
7613	7613	2154	2044	2076	1945	1927
7615	7615	2154	2046	2078	1947	1929
7605	7605	2146	2036	2068	1937	1919
7604	7604	2145	2035	2067	1936	1918
7613	7613	2173	2045	2087	1950	1932
7604	7604	2145	2035	2067	1936	1918
7605	7605	2146	2036	2068	1937	1919
7617	7617	2160	2050	2082	1951	1933
7594	7594	2176	2042	2110	1977	1949
7652	7652	2193	2039	2090	2043	2025
7740	7740	2371	762	73	2138	2112
7743	7743	2385	778	97	2144	2118
7755	7755	2389	780	85	2156	2130
7729	7729	2352	761		2131	2105
7726	7726	2218	522	359	1956	1922
7720	7720	2221	491	358	1947	1915
7720	7720	2221	491	358	1947	1915
7717	7717	2219	489	356	1945	1913
7720	7720	2229	503	364	1961	1927
7694	7694	2203	507	320	1943	1909
7725	7725	2201	513	332	1925	1891
7692	7692	2285	652	472	2016	1990
7681	7681	2236	437	523	1969	1935
7720	7720	2183	443	410	1917	1883
7706	7706	2216	519	474	1930	1898
7725	7725	2218	478	431	1944	1912
7729	7729	2223	483	436	1949	1917
7694	7694	2210	482	485	1934	1910
7724	7724	2222	446	433	1946	1924
7713	7713	2211	435	430	1945	1909
7680	7680	2184	292	539	1845	1815
7683	7683	2193	301	546	1855	1827
7684	7684	2365	13	768	2025	1995
7693	7693	2374	22	777	2034	2004
7677	7677	2358		761	2018	1988
7687	7687	2227	348	680	1946	1916
7676	7676	2191	307	568	1855	1833
7679	7679	2191	307	568	1856	1834
7564	7564	2443	617	977	2086	2060
7683	7683	2306	379	728	1959	1922
7673	7673	2309	382	733	1964	1921

7682	7682	2320	400	751	1975	1941
7689	7689	2323	395	746	1980	1936
7679	7679	2291	362	735	1952	1911
7704	7704	2262	391	650	1921	1892
7694	7694	2255	384	643	1914	1871
7692	7692	2253	382	641	1912	1869
7675	7675	2184	312	561	1847	1826
7655	7655	2193	298	550	1855	1831
7700	7700	2207	334	581	1864	1831
7693	7693	2200	325	572	1855	1822
7663	7663	2110	524	687	1801	1783
7663	7663	2110	524	687	1801	1783
7678	7678	2234	316	609	1891	1843
7673	7673	2229	311	604	1886	1838
7676	7676	2232	314	607	1889	1841
7518	7518	2238	1971	2076	1815	1803
7515	7515	2239	1972	2077	1816	1804
7505	7505	2226	1959	2064	1803	1791
7601	7601	2401	2110	2117	2053	2039
7530	7530	2229	1953	2044	1859	1846
7754	7754	2492	2271	2336	2147	2131
7769	7769	3848	3958	4033	3831	3814
7906	7906	6741	6657	6690	6598	6594
7908	7908	6743	6659	6692	6600	6596
7905	7905	6740	6656	6689	6597	6593
7907	7907	6742	6658	6691	6599	6595
7875	7875	6804	6728	6755	6663	6657
7893	7893	6793	6717	6744	6654	6644
7904	7904	6787	6711	6752	6656	6652
7883	7883	6833	6731	6762	6682	6678
7898	7898	6847	6745	6776	6696	6692
7887	7887	6821	6706	6739	6663	6657
7900	7900	6861	6771	6802	6716	6712
7876	7876	6819	6723	6754	6674	6670
7905	7905	6849	6753	6784	6703	6700
7869	7869	6800	6724	6755	6657	6653
7876	7876	6815	6721	6752	6678	6674
7880	7880	6825	6729	6760	6680	6676
8011	8011	6951	6882	6935	6849	6845
8006	8006	6946	6877	6930	6844	6840
7969	7969	6917	6841	6889	6810	6806
7905	7905	6823	6830	6795	6694	6690
8008	8008	6936	6935	6898	6820	6816
7894	7894	6822	6823	6776	6676	6672
7888	7888	6816	6817	6770	6670	6666
7889	7889	6813	6816	6769	6669	6665
7860	7860	6789	6785	6738	6646	6636
7767	7767	6837	6842	6787	6696	6688
7792	7792	3791	3740	3696	3507	3489
7781	7781	3780	3729	3685	3496	3478
		7585	7677	7729	7615	7600
		7585	7677	7729	7615	7600
3	3	7584	7676	7728	7614	7599
3	3	7583	7675	7727	7613	7598
5	5	7585	7677	7729	7615	7600

7	7	7583	7675	7727	7613	7598
7	7	7585	7677	7729	7615	7600
5	5	7583	7675	7727	7613	7598
8	8	7586	7678	7730	7616	7601
5	5	7583	7675	7727	7613	7598
4	4	7582	7674	7726	7612	7597
5	5	7583	7675	7727	7613	7598
6	6	7584	7676	7728	7614	7599
6	6	7584	7676	7728	7614	7599
286	286	7722	7743	7815	7722	7707
290	290	7726	7747	7819	7726	7711
290	290	7726	7747	7819	7726	7711
293	293	7729	7750	7822	7729	7714
385	385	7683	7713	7774	7683	7668
287	287	7723	7744	7816	7723	7708
263	263	7716	7733	7805	7715	7700
261	261	7714	7731	7803	7713	7698
265	265	7719	7736	7808	7718	7703
260	260	7714	7731	7803	7713	7698
260	260	7716	7733	7805	7715	7700
270	270	7720	7738	7810	7719	7704
324	324	7573	7689	7651	7579	7564
815	815	7633	7695	7727	7621	7608
880	880	7637	7695	7727	7621	7608
2046	2046	7773	7810	7889	7752	7742
2046	2046	7773	7810	7889	7752	7742
2053	2053	7778	7815	7894	7757	7747
2067	2067	7807	7857	7922	7792	7782
2147	2147	7791	7819	7881	7754	7740
3410	3410	7632	7625	7713	7626	7610
3413	3413	7635	7628	7716	7629	7613
3417	3417	7644	7633	7721	7634	7618
3434	3434	7639	7636	7718	7637	7621
3365	3365	7617	7604	7694	7605	7589
3373	3373	7625	7612	7702	7613	7597
3364	3364	7616	7603	7693	7604	7588
3416	3416	7692	7677	7765	7677	7661
3418	3418	7694	7679	7767	7679	7663
3423	3423	7698	7683	7771	7683	7667
3435	3435	7711	7696	7784	7696	7680
3426	3426	7643	7600	7670	7611	7597
3427	3427	7644	7601	7671	7612	7598
3427	3427	7644	7601	7671	7612	7598
3428	3428	7645	7602	7672	7613	7599
3426	3426	7643	7600	7670	7611	7597
3426	3426	7645	7602	7672	7613	7599
3502	3502	7668	7661	7718	7619	7604
3501	3501	7667	7660	7717	7618	7603
3500	3500	7666	7659	7716	7617	7602
3499	3499	7665	7658	7715	7616	7601
3501	3501	7667	7660	7717	7618	7603
3500	3500	7666	7659	7716	7617	7602
3532	3532	7643	7636	7693	7598	7583
3503	3503	7669	7662	7719	7620	7605
3525	3525	7731	7762	7807	7708	7695

3413	3413	7707	7737	7790	7694	7680
3416	3416	7710	7740	7793	7697	7683
3414	3414	7708	7738	7791	7695	7681
3411	3411	7705	7735	7788	7692	7678
25534	25534	24963	25029	25040	24984	24971
25533	25533	24962	25028	25039	24983	24970
31138	31138	30952	31001	31025	31012	30991

MOD1-EC5122	CFSAN041116	MOD1-EC5135	MOD1-EC5153	MOD1-EC5167	246164
3586	3590	3761	3532	3524	3518
3495	3499	3679	3513	3505	3486
3495	3499	3681	3511	3503	3486
3534	3538	3707	3549	3541	3523
3507	3511	3708	3529	3521	3512
3531	3535	3720	3560	3552	3543
3531	3535	3720	3560	3552	3543
3548	3552	3736	3566	3558	3542
3551	3555	3739	3568	3560	3544
3584	3588	3753	3594	3586	3535
3601	3605	3757	3619	3611	3556
3567	3571	3737	3586	3578	3533
3690	3694	3841	3672	3664	3655
3690	3694	3841	3672	3664	3655
3600	3604	3779	3607	3599	3580
3597	3601	3776	3604	3596	3580
3474	3478	3671	3495	3487	3474
3525	3529	3706	3536	3528	3526
3568	3572	3741	3601	3593	3438
3585	3589	3784	3646	3638	3562
3608	3612	3805	3654	3646	3574
3530	3534	3685	3522	3514	3434
3598	3602	3736	3599	3591	3545
3610	3614	3751	3626	3618	3569
3487	3491	3659	3515	3507	3446
3551	3555	3704	3564	3556	3519
3469	3473	3686	3513	3505	3424
3596	3600	3784	3651	3643	3570
3582	3586	3773	3623	3615	3552
3535	3539	3691	3548	3540	3446
3535	3539	3691	3548	3540	3446
3538	3542	3694	3550	3542	3448
3537	3541	3709	3572	3564	3431
3477	3481	3681	3515	3507	3418
3477	3481	3681	3515	3507	3418
1851	1857	2216	1954	1946	460
1842	1848	2185	1945	1937	440
1824	1830	2191	1925	1917	437
1821	1827	2182	1964	1956	498
1826	1832	2185	1927	1919	424
1840	1846	2197	1939	1931	423
1839	1845	2196	1938	1930	422
1824	1830	2195	1936	1928	439
1852	1858	2212	1955	1947	437
1894	1900	2245	1963	1955	407
1968	1974	2298	2037	2029	
1942	1948	2245	1973	1965	1922
1737	1743	2126	1750	1742	1869
1732	1738	2121	1745	1737	1864
1738	1744	2127	1751	1743	1870
1732	1738	2121	1745	1737	1864
1737	1743	2126	1750	1742	1869
1600	1604	2075	1931	1923	1870

1747	1751	2262	1964	1956	2021
24		1930	1895	1887	1974
23	9	1929	1894	1886	1973
16	22	1922	1887	1879	1966
	24	1924	1889	1881	1968
359	365	1915	1887	1879	1914
368	374	1924	1896	1888	1923
368	374	1924	1896	1888	1923
256	262	1868	1858	1850	1911
291	297	1885	1881	1873	1942
309	315	1898	1865	1857	1906
248	254	1860	1850	1842	1903
546	552	1910	1878	1870	1968
546	552	1910	1878	1870	1968
535	541	1899	1867	1859	1957
448	454	1925	1871	1863	1923
1469	1473	2109	1866	1858	1965
1500	1504	2140	1897	1889	1996
1471	1475	2111	1868	1860	1967
1469	1473	2109	1866	1858	1965
1929	1935	11	2122	2114	2303
1931	1937	13	2124	2116	2305
1925	1931	7	2118	2110	2299
1922	1928	4	2115	2107	2296
1926	1932	48	2109	2101	2299
1920	1926	12	2113	2105	2296
1924	1930		2117	2109	2298
1913	1919	21	2130	2122	2307
1924	1930	6	2117	2109	2298
1923	1929	5	2116	2108	2297
1938	1944	78	2089	2081	2304
1985	1991	93	2154	2146	2327
1930	1936	122	2064	2056	2324
1930	1936	122	2064	2056	2324
1930	1936	122	2064	2056	2324
2074	2080	1678	2074	2066	2325
2007	2013	1592	2037	2029	2291
1782	1788	1907	1978	1970	2067
1766	1772	1887	1958	1950	2051
1766	1772	1887	1958	1950	2051
1744	1750	1887	1964	1956	2038
1840	1846	2026	1868	1860	2166
2022	2026	1878	2199	2191	2332
2150	2154	1999	2211	2203	2413
2197	2201	1976	2236	2228	2448
2197	2201	1976	2236	2228	2448
2181	2185	2057	2301	2293	2460
2153	2157	2011	2270	2262	2412
2108	2114	1909	2164	2156	2435
2060	2066	1920	2202	2194	2444
2138	2144	1865	2244	2236	2494
2151	2157	1918	2253	2245	2503
1818	1822	2100	1859	1853	1971
1829	1833	2120	1870	1864	1981
1734	1738	2046	1806	1800	1971

1736	1740	2048	1808	1802	1975
1758	1762	2080	1816	1810	2004
1778	1782	2092	1874	1866	2077
1844	1848	2137	1885	1877	1992
1844	1848	2137	1885	1877	1992
1786	1790	2082	1918	1910	2053
1788	1792	2084	1920	1912	2055
1788	1792	2084	1920	1912	2055
1811	1815	2105	1946	1938	2072
1793	1797	2079	1923	1915	2044
1805	1809	2073	1931	1923	2072
1806	1810	2074	1932	1924	2073
1694	1698	2083	1885	1877	1987
1688	1692	2073	1879	1871	1977
1889	1895	2117		32	2037
1889	1895	2117	28	34	2039
1878	1884	2108	34	26	2029
1880	1886	2108	33	25	2028
1900	1906	2136	65	57	2042
1880	1886	2108	33	25	2028
1881	1887	2109	32		2029
1895	1901	2123	48	40	2043
1921	1927	2155	83	75	2067
1939	1945	2156	181	173	2132
1988	1994	2366	2091	2083	2254
2004	2010	2380	2100	2092	2254
2004	2010	2384	2107	2099	2272
1979	1985	2347	2076	2068	2247
1817	1823	2223	1951	1943	2070
1808	1814	2230	1944	1936	2063
1808	1814	2230	1944	1936	2063
1806	1812	2228	1942	1934	2061
1820	1826	2238	1942	1934	2075
1804	1810	2214	1927	1919	2057
1792	1798	2204	1916	1908	2039
1854	1860	2284	1987	1979	2132
1883	1889	2253	1944	1936	1957
1778	1784	2200	1914	1906	2035
1793	1799	2223	1923	1915	2036
1801	1807	2225	1937	1929	2058
1806	1812	2230	1942	1934	2063
1817	1823	2217	1932	1924	2034
1799	1805	2227	1941	1933	2066
1794	1800	2224	1934	1926	2061
1727	1733	2181	1880	1872	1969
1728	1734	2192	1883	1875	1981
1938	1944	2390	2051	2043	2064
1947	1953	2399	2060	2052	2073
1931	1937	2383	2044	2036	2057
1802	1808	2252	1959	1951	2062
1735	1741	2202	1891	1883	1981
1736	1742	2202	1891	1883	1982
2014	2020	2458	2114	2106	2102
1880	1886	2313	1982	1974	2011
1879	1885	2318	1981	1973	2012

1889	1895	2329	1991	1983	2024
1892	1898	2332	1994	1986	2029
1877	1883	2302	2017	2009	2067
1804	1810	2271	1961	1953	2046
1797	1803	2264	1954	1946	2036
1795	1801	2262	1952	1944	2034
1745	1751	2185	1900	1892	1978
1731	1737	2196	1887	1879	1983
1763	1769	2198	1916	1908	1992
1754	1760	2191	1909	1901	1983
1669	1675	2103	1804	1796	1927
1669	1675	2103	1804	1796	1927
1776	1782	2243	1930	1922	2012
1772	1778	2238	1925	1917	2007
1775	1781	2241	1928	1920	2010
1926	1932	2193	1879	1871	1904
1927	1933	2194	1880	1872	1905
1914	1920	2181	1867	1859	1892
2060	2066	2354	2125	2117	2121
1932	1938	2178	1930	1922	1915
2203	2209	2451	2103	2095	2222
3781	3787	3835	3795	3787	3758
6648	6654	6756	6666	6658	6599
6650	6656	6758	6668	6660	6601
6647	6653	6755	6665	6657	6598
6649	6655	6757	6667	6659	6600
6717	6723	6819	6722	6714	6662
6708	6714	6808	6708	6700	6651
6710	6716	6812	6716	6708	6659
6748	6754	6848	6752	6744	6683
6762	6768	6862	6766	6758	6697
6743	6749	6838	6739	6731	6670
6780	6786	6876	6790	6782	6717
6732	6738	6834	6736	6728	6675
6762	6768	6864	6766	6758	6705
6715	6721	6815	6719	6711	6658
6734	6740	6830	6738	6730	6679
6738	6744	6840	6742	6734	6681
6879	6885	6966	6914	6906	6835
6874	6880	6961	6909	6901	6830
6843	6849	6932	6872	6864	6779
6755	6761	6838	6762	6754	6692
6863	6869	6951	6884	6876	6805
6746	6752	6837	6755	6747	6684
6740	6746	6831	6749	6741	6678
6739	6745	6828	6748	6740	6677
6709	6715	6804	6725	6717	6654
6757	6763	6850	6781	6773	6700
3529	3535	3807	3557	3549	3560
3518	3524	3796	3546	3538	3549
7557	7560	7576	7613	7605	7534
7557	7560	7576	7613	7605	7534
7556	7559	7575	7612	7604	7533
7555	7558	7574	7611	7603	7532
7557	7560	7576	7613	7605	7534

7555	7558	7574	7611	7603	7532
7557	7560	7576	7613	7605	7534
7555	7558	7574	7611	7603	7532
7558	7561	7577	7614	7606	7535
7555	7558	7574	7611	7603	7532
7554	7557	7573	7610	7602	7531
7555	7558	7574	7611	7603	7532
7556	7559	7575	7612	7604	7533
7556	7559	7575	7612	7604	7533
7697	7700	7713	7711	7703	7674
7701	7704	7717	7715	7707	7678
7701	7704	7717	7715	7707	7678
7704	7707	7720	7718	7710	7681
7654	7657	7674	7676	7668	7643
7698	7701	7714	7712	7704	7675
7689	7692	7707	7703	7695	7666
7687	7690	7705	7701	7693	7664
7692	7695	7710	7706	7698	7669
7687	7690	7705	7701	7693	7664
7689	7692	7707	7703	7695	7666
7693	7696	7711	7709	7701	7670
7543	7546	7564	7602	7594	7579
7595	7598	7622	7617	7609	7575
7593	7596	7626	7613	7605	7575
7717	7722	7757	7746	7738	7708
7717	7722	7757	7746	7738	7708
7722	7727	7762	7751	7743	7712
7756	7761	7792	7786	7778	7748
7736	7742	7780	7776	7768	7713
7608	7614	7620	7647	7639	7575
7611	7617	7623	7650	7642	7578
7616	7622	7632	7655	7647	7583
7612	7617	7627	7652	7644	7582
7582	7588	7604	7621	7613	7556
7590	7596	7612	7629	7621	7564
7581	7587	7603	7620	7612	7555
7653	7659	7680	7695	7687	7624
7655	7661	7682	7697	7689	7626
7659	7665	7686	7701	7693	7630
7672	7678	7699	7714	7706	7643
7614	7618	7632	7643	7635	7566
7615	7619	7633	7644	7636	7567
7615	7619	7633	7644	7636	7567
7616	7620	7634	7645	7637	7568
7614	7618	7632	7643	7635	7566
7616	7620	7634	7645	7637	7568
7622	7626	7655	7656	7648	7564
7621	7625	7654	7655	7647	7563
7620	7624	7653	7654	7646	7562
7619	7623	7652	7653	7645	7561
7621	7625	7654	7655	7647	7563
7620	7624	7653	7654	7646	7562
7599	7603	7630	7631	7623	7543
7623	7627	7656	7657	7649	7565
7698	7702	7724	7722	7714	7653

7696	7700	7696	7690	7682	7640
7699	7703	7699	7693	7685	7643
7697	7701	7697	7691	7683	7641
7694	7698	7694	7688	7680	7638
25016	25018	24971	25041	25035	24986
25015	25017	24970	25040	25034	24985
30938	30944	30946	30983	30975	30998

ESC_DA2647AA	195745	181089	KCJK2721	MOD1-EC7010	MOD1-EC6900	KCJK4181
3784	3406	3583	3613	1073	6781	683
3690	3364	3533	3526	511	6733	784
3690	3364	3533	3526	513	6737	786
3745	3400	3559	3565	497	6749	862
3696	3377	3560	3538	558	6742	809
3743	3403	3583	3562	559	6745	806
3743	3403	3583	3562	559	6745	806
3743	3417	3589	3579	558	6770	787
3745	3421	3592	3582	560	6772	789
3785	3443	3611	3617	551	6783	912
3791	3518	3632	3632	437	6789	794
3770	3440	3609	3598	570	6793	842
3870	3542	3702	3721	630	6824	916
3870	3542	3702	3721	630	6824	916
3800	3466	3631	3631	585	6798	877
3797	3462	3628	3628	591	6810	877
3716	3332	3497	3505	535	6734	806
3735	3381	3548	3556	583	6730	856
3805	3435	3607	3599	619	6782	849
3746	3436	3613	3612	795	6794	1053
3830	3483	3652	3639	679	6812	923
3687	3431	3511	3563	564	6743	942
3781	3501	3619	3627		6746	798
3787	3527	3644	3639	376	6807	764
3712	3408	3519	3516	395	6730	658
3715	3454	3586	3580	493	6782	700
3689	3387	3543	3502	1067	6784	642
3768	3511	3664	3635	1038	6784	807
3789	3502	3624	3613	890	6777	474
3730	3438	3569	3564	798	6743	
3730	3438	3569	3564	798	6743	
3732	3441	3572	3567	801	6744	19
3727	3402	3548	3566	859	6775	310
3685	3393	3543	3508	1069	6778	625
3685	3393	3543	3508	1069	6778	625
1957	1794	1842	1812	3595	6680	3493
1975	1785	1839	1803	3595	6670	3484
1961	1767	1827	1785	3609	6675	3493
1970	1806	1856	1782	3546	6658	3418
1941	1771	1820	1787	3593	6666	3445
1941	1779	1841	1801	3605	6662	3479
1940	1778	1840	1800	3604	6661	3478
1972	1755	1834	1787	3579	6674	3485
1975	1793	1853	1813	3584	6692	3500
1949	1823	1877	1855	3558	6660	3489
2024	1869	1971	1911	3545	6678	3446
2057	1849	1912	1895	3627	6792	3544
1964		1735	1708	3501	6687	3438
1959	15	1730	1703	3496	6682	3433
1965	25	1736	1709	3502	6688	3439
1959	19	1730	1703	3496	6682	3433
1964	26	1735	1708	3501	6687	3438
1979	1447	1696	1519	3612	6719	3567

1998	1691	1735	1710	3702	6825	3637
1895	1743	1738	262	3602	6746	3539
1894	1742	1737	261	3601	6745	3538
1887	1735	1732	254	3596	6737	3533
1889	1737	1734	256	3598	6740	3535
1861	1696	1698	179	3655	6752	3600
1870	1705	1707	188	3664	6761	3609
1870	1705	1707	188	3664	6761	3609
1834	1708	1699		3627	6742	3564
1859	1729	1720	59	3650	6757	3587
1829	1745	1735	75	3611	6736	3557
1826	1700	1691	14	3619	6734	3556
1821	1739	1737	340	3632	6742	3564
1821	1739	1737	340	3632	6742	3564
1810	1728	1726	329	3621	6731	3553
1790	1681	1743	226	3628	6727	3571
1883	1616	1776	1424	3625	6803	3539
1914	1647	1807	1455	3656	6831	3570
1885	1618	1778	1426	3627	6805	3541
1883	1616	1776	1424	3625	6803	3539
2334	2131	2051	1873	3740	6836	3695
2336	2133	2053	1875	3743	6838	3698
2330	2127	2047	1869	3737	6832	3692
2327	2124	2044	1866	3734	6829	3689
2321	2114	2054	1870	3748	6840	3696
2325	2122	2042	1864	3736	6833	3689
2329	2126	2046	1868	3736	6831	3691
2338	2141	2061	1857	3743	6842	3698
2329	2126	2046	1868	3736	6831	3691
2328	2125	2045	1867	3735	6830	3690
2328	2132	2056	1882	3727	6831	3667
2320	2173	2107	1927	3737	6816	3686
2335	2032	2046	1870	3736	6835	3691
2335	2032	2046	1870	3736	6835	3691
2335	2032	2046	1870	3736	6835	3691
2243	2105	2079	2025	3771	6808	3687
2180	2088	1995	1956	3757	6808	3669
1983	1883	1949	1719	3624	6702	3582
1977	1865	1939	1703	3610	6706	3578
1977	1865	1939	1703	3610	6706	3578
1969	1851	1917	1687	3615	6702	3579
2024	1820	1840	1787	3581	6788	3556
2278	2055	1835	1962	3649	6815	3585
2406	2113	1955	2092	3699	6800	3635
2475	2102	1988	2141	3692	6806	3624
2475	2102	1988	2141	3692	6806	3624
2466	2143	2001	2136	3689	6821	3616
2430	2105	1979	2108	3672	6814	3602
2422	2107	2007	2072	3721	6805	3645
2423	2082	2094	2032	3783	6780	3714
2407	2250	2178	2105	3714	6875	3680
2404	2283	2215	2110	3714	6886	3670
1877	1795	133	1783	3594	6747	3540
1890	1804	142	1794	3612	6767	3543
1890	1735		1699	3619	6762	3569

1892	1739	28	1701	3619	6765	3569
1914	1763	74	1717	3619	6763	3561
1949	1888	412	1709	3591	6783	3551
2029	1818	423	1787	3621	6788	3549
2029	1818	423	1787	3621	6790	3549
2001	1814	473	1735	3628	6804	3587
2003	1816	475	1737	3630	6806	3589
2003	1816	475	1737	3630	6806	3589
2013	1838	487	1760	3642	6811	3602
2004	1821	478	1742	3629	6799	3580
2032	1815	530	1754	3607	6813	3562
2033	1816	531	1755	3608	6814	3563
1883	1775	739	1627	3637	6784	3572
1889	1771	735	1621	3649	6782	3568
1991	1750	1806	1858	3599	6749	3548
1993	1752	1808	1858	3599	6749	3548
1983	1741	1800	1847	3591	6741	3540
1982	1741	1799	1849	3590	6740	3539
1970	1761	1827	1869	3602	6731	3527
1982	1741	1801	1849	3590	6740	3539
1983	1742	1800	1850	3591	6741	3540
1997	1756	1814	1864	3605	6755	3554
2014	1787	1839	1890	3590	6754	3532
2003	1807	1851	1908	3620	6769	3568
756	2066	2039	1927	3826	6769	3774
762	2071	2059	1943	3828	6773	3767
774	2082	2055	1943	3842	6786	3786
751	2037	2018	1918	3851	6770	3795
520	1880	1882	1740	3759	6827	3677
497	1873	1877	1731	3744	6827	3694
497	1873	1877	1731	3744	6827	3694
495	1871	1875	1729	3742	6824	3692
503	1879	1881	1743	3756	6835	3700
492	1848	1858	1727	3789	6833	3726
475	1851	1853	1715	3786	6833	3720
615	1933	1940	1777	3837	6824	3783
443	1904	1912	1808	3765	6819	3667
417	1811	1855	1703	3790	6820	3720
459	1818	1886	1718	3804	6842	3749
434	1836	1878	1726	3807	6847	3745
439	1841	1883	1731	3812	6853	3750
436	1856	1876	1742	3787	6828	3719
454	1834	1878	1724	3751	6834	3705
445	1829	1873	1719	3750	6825	3690
268	1830	1768	1672	3771	6808	3697
269	1831	1768	1673	3773	6813	3705
407	2027	1953	1883	3800	6824	3805
416	2036	1962	1892	3809	6833	3814
400	2020	1946	1876	3793	6817	3798
440	1913	1809	1747	3800	6802	3744
255	1836	1768	1680	3781	6804	3707
257	1837	1769	1681	3784	6805	3712
600	2124	1999	1955	3828	6747	3714
47	1957	1885	1825	3759	6792	3707
24	1954	1880	1824	3760	6785	3706

	1964	1890	1834	3781	6790	3730
37	1967	1893	1837	3776	6801	3725
203	1963	1894	1822	3818	6819	3730
181	1903	1833	1749	3802	6831	3744
156	1896	1823	1742	3795	6824	3723
154	1894	1821	1740	3793	6822	3721
265	1846	1776	1690	3753	6808	3699
268	1833	1769	1676	3770	6807	3707
294	1864	1799	1708	3792	6828	3695
285	1857	1792	1699	3785	6821	3688
490	1830	1664	1618	3766	6788	3702
490	1830	1664	1618	3766	6788	3702
266	1876	1807	1721	3774	6811	3685
261	1871	1802	1717	3769	6806	3680
264	1874	1805	1720	3772	6809	3683
1956	1908	1775	1881	3545	6691	3520
1957	1909	1776	1882	3547	6690	3521
1944	1896	1763	1869	3535	6679	3508
2095	2149	2006	2030	3705	6792	3691
1918	2008	1811	1885	3580	6714	3534
2226	2239	2002	2148	3788	6953	3766
3895	3665	3830	3772	3449	6987	3415
6718	6591	6674	6658	6598	384	6599
6720	6593	6676	6660	6600	386	6601
6717	6590	6673	6657	6597	383	6598
6719	6592	6675	6659	6599	385	6600
6786	6667	6744	6719	6737	270	6739
6776	6657	6738	6710	6725	259	6726
6786	6661	6740	6712	6735	263	6736
6792	6691	6764	6750	6773	195	6774
6806	6705	6778	6764	6787	211	6788
6787	6684	6759	6745	6741	250	6753
6832	6729	6790	6782	6829	251	6830
6784	6681	6756	6734	6753	173	6754
6814	6711	6786	6764	6782	203	6783
6785	6664	6743	6717	6744	194	6745
6782	6685	6760	6736	6759	181	6760
6790	6687	6762	6740	6759	179	6760
6984	6832	6891	6881	6942	428	6958
6979	6827	6886	6876	6937	423	6953
6922	6794	6858	6845	6891	419	6919
6821	6703	6781	6757	6772	195	6768
6927	6807	6884	6865	6883	341	6899
6796	6693	6768	6748	6752	10	6749
6790	6687	6762	6742	6746		6743
6789	6686	6761	6741	6751	15	6748
6763	6673	6735	6711	6732	119	6729
6812	6713	6778	6759	6781	286	6779
3676	3508	3338	3546	4224	6908	4167
3665	3497	3327	3535	4213	6898	4156
7682	7570	7557	7529	7500	7888	7498
7682	7570	7557	7529	7500	7888	7498
7681	7569	7556	7528	7499	7887	7497
7680	7568	7555	7527	7498	7886	7496
7682	7570	7557	7529	7500	7888	7498

7680	7568	7555	7527	7498	7888	7496
7682	7570	7557	7529	7500	7888	7498
7680	7568	7555	7527	7498	7886	7496
7683	7571	7558	7530	7501	7889	7499
7680	7568	7555	7527	7498	7886	7496
7679	7567	7554	7526	7497	7885	7495
7680	7568	7555	7527	7498	7886	7496
7681	7569	7556	7528	7499	7887	7497
7681	7569	7556	7528	7499	7887	7497
7770	7644	7694	7669	7603	7925	7576
7774	7648	7698	7673	7607	7929	7580
7774	7648	7698	7673	7607	7929	7580
7777	7651	7701	7676	7610	7932	7583
7743	7603	7651	7628	7548	7935	7514
7771	7645	7695	7670	7604	7926	7577
7760	7636	7686	7661	7597	7920	7570
7758	7634	7684	7659	7595	7919	7568
7763	7639	7689	7664	7600	7923	7573
7758	7634	7684	7659	7595	7918	7568
7760	7636	7686	7661	7596	7920	7569
7765	7640	7691	7665	7602	7923	7575
7677	7503	7567	7515	7534	7865	7470
7691	7549	7592	7568	7529	7865	7472
7691	7545	7602	7566	7527	7855	7470
7835	7703	7739	7693	7685	7947	7669
7835	7703	7739	7693	7685	7947	7669
7840	7708	7744	7698	7692	7954	7676
7872	7742	7778	7731	7742	7992	7724
7838	7702	7759	7707	7669	7931	7659
7658	7564	7607	7592	7536	7687	7522
7661	7567	7610	7595	7539	7690	7525
7666	7574	7617	7600	7546	7698	7538
7673	7566	7610	7590	7521	7675	7527
7639	7548	7581	7571	7519	7718	7515
7647	7556	7589	7579	7527	7726	7523
7638	7547	7580	7570	7518	7717	7514
7712	7608	7655	7633	7580	7724	7578
7714	7610	7657	7635	7582	7726	7580
7718	7614	7661	7639	7586	7731	7584
7731	7627	7674	7652	7599	7743	7597
7637	7579	7574	7584	7554	7743	7556
7638	7580	7575	7585	7553	7744	7557
7638	7580	7575	7585	7555	7744	7557
7639	7581	7576	7586	7556	7745	7558
7637	7579	7574	7584	7554	7743	7556
7639	7581	7576	7586	7556	7745	7558
7674	7586	7589	7581	7527	7773	7542
7673	7585	7588	7580	7526	7772	7541
7672	7584	7587	7579	7525	7771	7540
7671	7583	7586	7578	7524	7770	7539
7673	7585	7588	7580	7526	7772	7541
7672	7584	7587	7579	7525	7771	7540
7649	7563	7564	7558	7508	7798	7523
7675	7587	7590	7582	7528	7774	7543
7753	7674	7695	7680	7605	7793	7611

7723	7638	7653	7664	7659	7955	7667
7726	7641	7656	7667	7662	7960	7670
7724	7639	7654	7665	7660	7958	7668
7721	7636	7651	7662	7657	7953	7665
25026	25011	24982	25005	24927	25018	24923
25025	25010	24981	25004	24926	25017	24922
30998	30971	31009	30924	30950	30787	30910

MOD1-EC6811	MOD1-EC6825	KCJK4201	CFSAN046659	MOD1-ECOR25	MOD1-ECOR34
3725	7575	683	3726	830	3759
3671	7507	784	3696	14	3697
3671	7509	786	3696		3697
3717	7532	862	3747	256	3732
3702	7554	809	3721	197	3701
3736	7513	806	3742	192	3741
3736	7513	806	3742	192	3741
3730	7548	787	3743	144	3754
3732	7554	789	3745	146	3756
3761	7500	912	3785	353	3798
3771	7510	794	3786	461	3801
3758	7548	842	3763	193	3774
3838	7609	916	3891	578	3868
3838	7609	916	3891	578	3868
3768	7559	877	3821	430	3798
3765	7565	877	3818	430	3795
3652	7526	806	3691	348	3672
3707	7556	856	3746	401	3725
3755	7561	849	3799	431	3775
3759	7547	1053	3819	599	3780
3797	7570	923	3835	486	3822
3638	7545	942	3693	724	3659
3742	7526	798	3792	513	3766
3780	7552	764	3806	495	3798
3649	7514	658	3709	432	3677
3699	7575	700	3708	552	3713
3634	7590	642	3665	1144	3659
3743	7590	807	3828	1280	3793
3742	7546	474	3773	964	3773
3692	7541		3695	786	3702
3692	7541		3695	786	3702
3694	7544	19	3697	789	3704
3664	7567	310	3702	861	3702
3624	7578	625	3659	1131	3653
3624	7578	625	3659	1131	3653
1916	7623	3493	1884	3483	1814
1945	7618	3484	1864	3480	1801
1935	7620	3493	1861	3484	1789
1938	7625	3418	1870	3445	1798
1913	7603	3445	1831	3455	1783
1939	7617	3479	1871	3471	1799
1938	7616	3478	1870	3470	1798
1918	7615	3485	1844	3469	1772
1947	7612	3500	1881	3485	1809
1983	7592	3489	1915	3503	1843
2061	7563	3446	1992	3486	1927
1998	7604	3544	1936	3540	1856
1871	7585	3438	1864	3364	1830
1866	7582	3433	1859	3359	1825
1872	7588	3439	1865	3365	1831
1866	7582	3433	1859	3359	1825
1871	7587	3438	1864	3364	1830
1825	7624	3567	1885	3525	1803

1956	7671	3637	1911	3576	1887
1812	7625	3539	1769	3499	1675
1811	7624	3538	1768	3498	1674
1804	7619	3533	1761	3493	1667
1806	7621	3535	1763	3495	1669
1758	7605	3600	1735	3554	1645
1767	7614	3609	1744	3563	1654
1767	7614	3609	1744	3563	1654
1729	7580	3564	1708	3526	1618
1754	7587	3587	1733	3549	1643
1754	7562	3557	1725	3510	1635
1721	7572	3556	1700	3518	1610
1740	7603	3564	1719	3527	1627
1740	7603	3564	1719	3527	1627
1729	7592	3553	1708	3516	1616
1697	7590	3571	1674	3527	1586
1836	7587	3539	1770	3516	1719
1867	7616	3570	1801	3547	1750
1838	7589	3541	1772	3518	1721
1836	7587	3539	1770	3516	1719
2232	7659	3695	2203	3685	2108
2235	7661	3698	2205	3688	2110
2229	7655	3692	2199	3682	2104
2226	7652	3689	2196	3679	2101
2208	7661	3696	2195	3688	2087
2224	7654	3689	2196	3679	2099
2228	7654	3691	2198	3681	2103
2227	7655	3698	2207	3688	2112
2228	7654	3691	2198	3681	2103
2227	7653	3690	2197	3680	2102
2238	7654	3667	2194	3657	2099
2219	7667	3686	2207	3674	2110
2234	7636	3691	2204	3619	2109
2234	7636	3691	2204	3619	2109
2234	7636	3691	2204	3619	2109
2185	7714	3687	2180	3688	2091
2118	7715	3669	2091	3692	1998
1833	7626	3582	1853	3524	1791
1827	7624	3578	1833	3523	1769
1827	7624	3578	1833	3523	1769
1817	7621	3579	1835	3526	1763
1970	7618	3556	1925	3494	1895
2167	7683	3585	2173	3592	2107
2285	7707	3635	2299	3640	2229
2318	7725	3624	2360	3635	2278
2318	7725	3624	2360	3635	2278
2327	7726	3616	2373	3624	2269
2293	7725	3602	2322	3612	2255
2279	7693	3645	2307	3654	2206
2290	7707	3714	2311	3726	2214
2355	7769	3680	2282	3656	2208
2332	7761	3670	2279	3656	2203
1934	7577	3540	1839	3500	1725
1949	7598	3543	1839	3502	1740
1875	7588	3569	1799	3533	1664

1879	7590	3569	1803	3533	1668
1887	7594	3561	1825	3533	1688
1926	7568	3551	1878	3531	1748
1968	7597	3549	1898	3525	1812
1968	7599	3549	1898	3525	1812
1988	7612	3587	1914	3550	1804
1990	7614	3589	1916	3552	1806
1990	7614	3589	1916	3552	1806
1998	7618	3602	1937	3539	1831
1985	7611	3580	1911	3550	1801
1991	7595	3562	1939	3530	1815
1992	7596	3563	1940	3531	1816
1798	7608	3572	1763	3562	1616
1810	7606	3568	1753	3563	1606
1942	7655	3548	1916	3511	1804
1944	7657	3548	1918	3511	1806
1934	7647	3540	1908	3503	1796
1933	7646	3539	1907	3502	1795
1953	7657	3527	1921	3492	1809
1933	7646	3539	1907	3502	1795
1934	7647	3540	1908	3503	1796
1948	7659	3554	1922	3517	1810
1935	7651	3532	1954	3471	1842
1956	7633	3568	1928	3534	1816
329	7715	3774	586	3751	692
353	7722	3767	606	3753	712
345	7732	3786	604	3767	710
356	7717	3795	581	3776	687
79	7714	3677	362	3670	498
16	7711	3694	353	3673	491
16	7711	3694	353	3673	491
	7708	3692	351	3671	489
50	7715	3700	359	3671	497
97	7713	3726	343	3669	481
88	7725	3720	317	3697	457
234	7688	3783	453	3759	570
275	7702	3667	364	3696	538
162	7724	3720	261	3703	435
225	7742	3749	329	3702	503
182	7742	3745	286	3705	460
187	7744	3750	291	3710	465
236	7725	3719	324	3703	490
127	7718	3705	304	3682	476
116	7709	3690	301	3671	471
307	7687	3697	76	3690	266
312	7693	3705	89	3691	279
496	7667	3805	341	3814	531
505	7676	3814	350	3823	540
489	7660	3798	334	3807	524
449	7675	3744	298	3726	450
316	7692	3707	105	3693	287
316	7693	3712	105	3696	287
730	7628	3714	532	3719	703
484	7671	3707	267	3676	475
477	7662	3706	272	3666	480

495	7673	3730	294	3690	490
490	7678	3725	289	3685	493
484	7676	3730	246	3674	474
394	7694	3744	183	3696	379
387	7682	3723	164	3685	372
385	7680	3721	162	3683	370
311	7685	3699	88	3684	292
313	7682	3707	91	3662	283
351	7704	3695		3696	304
342	7697	3688	37	3689	295
489	7672	3702	304	3697	
489	7672	3702	304	3697	
313	7683	3685	140	3690	342
308	7678	3680	135	3685	337
311	7681	3683	138	3688	340
1953	7562	3520	1863	3458	1805
1954	7559	3521	1864	3460	1806
1941	7549	3508	1851	3448	1793
2052	7615	3691	2005	3626	1970
1913	7555	3534	1809	3485	1747
2193	7784	3766	2134	3698	2035
3891	7776	3415	3925	3346	3935
6746	7785	6599	6746	6608	6698
6748	7787	6601	6748	6610	6700
6745	7784	6598	6745	6607	6697
6747	7786	6600	6747	6609	6699
6810	7782	6739	6817	6725	6767
6798	7778	6726	6806	6736	6754
6810	7784	6736	6810	6749	6760
6818	7767	6774	6820	6783	6780
6832	7784	6788	6834	6797	6794
6797	7769	6753	6797	6764	6767
6858	7786	6830	6860	6839	6810
6810	7760	6754	6812	6763	6772
6840	7789	6783	6841	6792	6802
6811	7751	6745	6813	6754	6769
6808	7762	6760	6810	6769	6770
6816	7766	6760	6818	6769	6778
6986	7881	6958	6990	6976	6929
6981	7876	6953	6985	6971	6924
6940	7868	6919	6944	6935	6883
6849	7793	6768	6853	6778	6809
6952	7891	6899	6958	6907	6918
6830	7776	6749	6834	6743	6794
6824	7772	6743	6828	6737	6788
6823	7773	6748	6827	6742	6787
6792	7742	6729	6796	6723	6764
6841	7667	6779	6851	6744	6811
3650	7823	4167	3682	4159	3610
3639	7810	4156	3671	4148	3599
7717	3501	7498	7700	7470	7663
7717	3501	7498	7700	7470	7663
7716	3500	7497	7699	7469	7662
7715	3500	7496	7698	7468	7661
7717	3500	7498	7700	7470	7663

7715	3500	7496	7698	7468	7661
7717	3500	7498	7700	7470	7663
7715	3498	7496	7698	7468	7661
7718	3501	7499	7701	7471	7664
7715	3498	7496	7698	7468	7661
7714	3497	7495	7697	7467	7660
7715	3498	7496	7698	7468	7661
7716	3499	7497	7699	7469	7662
7716	3499	7497	7699	7469	7662
7803	3366	7576	7790	7562	7753
7807	3370	7580	7794	7566	7757
7807	3370	7580	7794	7566	7757
7810	3373	7583	7797	7569	7760
7764	3428	7514	7751	7495	7710
7804	3367	7577	7791	7563	7754
7793	3377	7570	7780	7556	7745
7791	3375	7568	7778	7554	7743
7796	3379	7573	7783	7559	7748
7791	3374	7568	7778	7554	7743
7793	3376	7569	7780	7555	7745
7798	3376	7575	7785	7561	7750
7639	3461	7470	7616	7444	7579
7719	3592	7472	7695	7460	7660
7719	3648	7470	7695	7458	7662
7871	3377	7669	7849	7633	7811
7871	3377	7669	7849	7633	7811
7876	3385	7676	7854	7640	7816
7906	3403	7724	7886	7690	7848
7880	3357	7659	7860	7620	7826
7700	2642	7522	7697	7512	7662
7703	2645	7525	7700	7515	7665
7708	2649	7538	7705	7522	7670
7713	2655	7527	7710	7501	7677
7681	2555	7515	7676	7489	7641
7689	2563	7523	7684	7497	7649
7680	2554	7514	7675	7488	7640
7754	2579	7578	7749	7562	7711
7756	2581	7580	7751	7564	7713
7760	2586	7584	7755	7568	7717
7773	2598	7597	7768	7581	7730
7672	2624	7556	7667	7544	7633
7673	2625	7557	7668	7543	7634
7673	2625	7557	7668	7545	7634
7674	2626	7558	7669	7546	7635
7672	2624	7556	7667	7544	7633
7674	2624	7558	7669	7546	7635
7709	7	7542	7705	7510	7673
7708	6	7541	7704	7509	7672
7707	5	7540	7703	7508	7671
7706	4	7539	7702	7507	7670
7708		7541	7704	7509	7672
7707	3	7540	7703	7508	7671
7684	42	7523	7680	7491	7648
7710	12	7543	7706	7511	7674
7792	2660	7611	7787	7573	7748

7769	2598	7667	7760	7642	7731
7772	2601	7670	7763	7645	7734
7770	2599	7668	7761	7643	7732
7767	2599	7665	7758	7640	7729
25038	25523	24923	25047	24901	25029
25037	25522	24922	25046	24900	25028
31006	31233	30910	31001	30906	31008

MOD1-EC6368	MOD1-EC6936	MOD1-EC6953	MOD1-EC6831	MOD1-EC6779	MOD1-EC6577
3748	3764	3401	6924	3799	884
3662	3703	3359	6903	3753	399
3662	3703	3359	6907	3753	401
3733	3773	3395	6908	3792	441
3712	3748	3372	6912	3765	418
3750	3774	3398	6914	3807	424
3750	3774	3398	6914	3807	424
3724	3762	3412	6940	3810	413
3726	3764	3416	6942	3812	415
3788	3801	3438	6928	3850	461
3767	3789	3513	6932	3853	603
3749	3768	3435	6916	3845	458
3866	3887	3537	6857	3930	444
3866	3887	3537	6857	3930	444
3796	3817	3461	6833	3860	293
3793	3814	3457	6845	3857	283
3670	3683	3327	6877	3736	172
3723	3744	3376	6854	3799	
3753	3747	3430	6885	3838	349
3760	3766	3431	6916	3889	530
3790	3806	3478	6943	3878	369
3676	3682	3426	6861	3727	779
3770	3787	3496	6883	3828	583
3802	3817	3522	6839	3862	582
3675	3706	3403	6879	3727	589
3713	3746	3449	6922	3771	554
3644	3685	3382	6923	3724	1153
3811	3814	3506	6924	3841	1237
3728	3758	3497	6901	3843	984
3707	3719	3433	6899	3767	856
3707	3719	3433	6899	3767	856
3709	3721	3436	6900	3769	859
3703	3697	3397	6916	3745	931
3645	3673	3388	6917	3718	1149
3645	3673	3388	6917	3718	1149
1845	1925	1789	6830	2141	3495
1855	1934	1780	6820	2144	3499
1838	1920	1762	6825	2134	3503
1856	1927	1801	6808	2143	3479
1831	1910	1766	6816	2118	3484
1857	1920	1774	6820	2142	3485
1856	1919	1773	6819	2141	3484
1830	1925	1750	6814	2120	3505
1856	1934	1788	6813	2156	3526
1901	1962	1818	6796	2190	3536
1983	2034	1864	6805	2254	3526
1928	1963	1844	6911	2156	3577
1833	1856	15	6807	2071	3381
1828	1851		6802	2066	3376
1834	1857	20	6808	2072	3382
1828	1851	14	6802	2066	3376
1833	1856	21	6807	2071	3381
1854	1834	1442	6842	2021	3533

1878	1937	1686	6960	2143	3610
1737	1823	1738	6869	2010	3529
1736	1822	1737	6868	2009	3528
1729	1815	1730	6858	2002	3523
1731	1817	1732	6863	2004	3525
1703	1769	1691	6876	1963	3584
1712	1778	1700	6885	1972	3593
1712	1778	1700	6885	1972	3593
1676	1742	1703	6865	1943	3556
1701	1767	1724	6880	1968	3579
1693	1721	1740	6866	1960	3542
1668	1734	1695	6857	1935	3548
1687	1755	1734	6863	1946	3571
1687	1755	1734	6863	1946	3571
1676	1744	1723	6852	1935	3560
1642	1702	1676	6848	1903	3559
1741	1879	1611	6924	2020	3541
1772	1910	1642	6952	2051	3572
1743	1881	1613	6926	2022	3543
1741	1879	1611	6924	2020	3541
2201	2222	2126	6956	2384	3710
2203	2224	2128	6958	2387	3713
2197	2218	2122	6952	2381	3707
2194	2215	2119	6949	2378	3704
2176	2221	2109	6960	2360	3716
2192	2213	2117	6953	2376	3702
2196	2217	2121	6951	2380	3706
2205	2226	2136	6962	2385	3715
2196	2217	2121	6951	2380	3706
2195	2216	2120	6950	2379	3705
2191	2219	2127	6950	2390	3674
2193	2210	2168	6936	2385	3699
2202	2223	2027	6955	2386	3650
2202	2223	2027	6955	2386	3650
2202	2223	2027	6955	2386	3650
2150	2178	2100	6926	2335	3694
2061	2097	2083	6928	2258	3716
1829	1866	1878	6821	1992	3550
1816	1862	1860	6825	1974	3546
1816	1862	1860	6825	1974	3546
1808	1838	1846	6821	1966	3553
1904	1959	1815	6929	2159	3526
2156	2194	2050	6932	2341	3606
2284	2312	2108	6917	2459	3654
2351	2345	2097	6923	2480	3647
2351	2345	2097	6923	2480	3647
2353	2346	2138	6936	2499	3636
2319	2314	2100	6931	2465	3625
2298	2308	2102	6922	2441	3672
2299	2315	2077	6897	2458	3744
2268	2330	2245	7000	2507	3704
2265	2311	2278	7011	2486	3702
1821	1929	1790	6877	2099	3508
1837	1944	1799	6897	2118	3520
1769	1876	1730	6884	2059	3548

1773	1880	1734	6886	2063	3548
1795	1888	1758	6884	2071	3548
1844	1917	1883	6916	2101	3538
1899	1967	1813	6909	2147	3560
1899	1967	1813	6911	2147	3560
1882	1949	1809	6935	2147	3569
1884	1951	1811	6937	2149	3571
1884	1951	1811	6937	2149	3571
1879	1943	1833	6942	2170	3581
1890	1948	1816	6930	2146	3570
1916	1954	1810	6944	2146	3548
1917	1955	1811	6945	2147	3549
1730	1817	1770	6912	2012	3577
1731	1821	1766	6910	2006	3583
1887	1932	1745	6884	2100	3536
1889	1934	1747	6884	2102	3536
1879	1924	1736	6876	2092	3528
1878	1923	1736	6875	2091	3527
1892	1929	1756	6878	2107	3521
1878	1923	1736	6875	2091	3527
1879	1924	1737	6876	2092	3528
1893	1938	1751	6890	2106	3542
1895	1937	1782	6889	2106	3547
1899	1950	1802	6873	2110	3553
557	494	2061	6897	46	3797
577	494	2066	6904		3799
573	508	2077	6914	68	3815
550	485	2032	6898	97	3810
331	252	1875	6955	358	3722
315	238	1868	6955	355	3709
315	238	1868	6955	355	3709
313	236	1866	6952	353	3707
319	237	1874	6963	361	3715
276	208	1843	6961	383	3734
288	209	1846	6961	363	3733
417	353	1928	6952	503	3790
328	212	1899	6930	548	3758
226	153	1806	6948	435	3735
273	125	1813	6981	489	3751
230	94	1831	6975	460	3752
235	99	1836	6981	465	3757
270		1851	6965	494	3744
262	180	1829	6962	434	3720
259	173	1824	6953	431	3709
53	282	1825	6938	564	3724
53	285	1826	6943	573	3724
305	489	2022	6942	785	3821
314	498	2031	6951	794	3830
298	482	2015	6935	778	3814
263	484	1908	6923	699	3759
69	286	1831	6934	595	3722
69	286	1832	6935	595	3727
470	639	2119	6865	978	3811
252	435	1952	6929	739	3731
250	426	1949	6922	744	3716

268	436	1959	6927	762	3735
263	439	1962	6938	757	3730
226	396	1958	6947	748	3736
149	365	1898	6961	675	3741
142	358	1891	6954	668	3731
140	356	1889	6952	666	3729
66	296	1841	6936	588	3702
	270	1828	6937	577	3723
91	324	1859	6958	606	3746
82	315	1852	6951	597	3739
283	490	1825	6918	712	3725
283	490	1825	6918	712	3725
114	325	1871	6941	614	3721
109	320	1866	6936	609	3716
112	323	1869	6939	612	3719
1841	1978	1901	6822	2118	3508
1842	1979	1904	6821	2119	3510
1829	1966	1891	6810	2106	3498
1977	2113	2144	6923	2157	3668
1797	1954	2003	6846	2072	3538
2106	2226	2234	7084	2356	3731
3920	3911	3660	7136	4031	3407
6725	6749	6586	574	6696	6587
6727	6751	6588	576	6698	6589
6724	6748	6585	573	6695	6586
6726	6750	6587	575	6697	6588
6769	6801	6662	460	6763	6724
6780	6803	6652	449	6752	6718
6789	6817	6656	453	6758	6728
6799	6823	6686	385	6768	6762
6813	6837	6700	401	6782	6776
6776	6818	6679	441	6745	6740
6839	6863	6724	441	6808	6818
6791	6815	6676	363	6760	6742
6821	6845	6706	393	6790	6771
6792	6816	6659	384	6761	6733
6789	6813	6680	371	6758	6748
6797	6821	6682	369	6766	6748
6969	6997	6827	511	6941	6945
6964	6992	6822	506	6936	6940
6923	6933	6789	574	6891	6898
6832	6852	6698	326	6797	6701
6937	6965	6802		6904	6854
6813	6834	6688	347	6779	6736
6807	6828	6682	341	6773	6730
6806	6827	6681	352	6772	6735
6775	6799	6668	454	6741	6716
6805	6830	6708	623	6792	6767
3656	3647	3503	7021	3730	4202
3645	3636	3492	7011	3719	4191
7655	7694	7569	8008	7743	7514
7655	7694	7569	8008	7743	7514
7654	7693	7568	8007	7742	7513
7653	7692	7567	8006	7741	7512
7655	7694	7569	8008	7743	7514

7653	7692	7567	8008	7741	7512
7655	7694	7569	8008	7743	7514
7653	7692	7567	8006	7741	7512
7656	7695	7570	8009	7744	7515
7653	7692	7567	8006	7741	7512
7652	7691	7566	8005	7740	7511
7653	7692	7567	8006	7741	7512
7654	7693	7568	8007	7742	7513
7654	7693	7568	8007	7742	7513
7745	7792	7643	8049	7831	7593
7749	7796	7647	8053	7835	7597
7749	7796	7647	8053	7835	7597
7752	7799	7650	8056	7838	7600
7706	7753	7600	8052	7790	7528
7746	7793	7644	8050	7832	7594
7735	7782	7635	8044	7821	7587
7733	7780	7633	8043	7819	7585
7738	7785	7638	8047	7824	7590
7733	7780	7633	8042	7819	7585
7735	7782	7635	8044	7821	7586
7740	7787	7639	8047	7826	7592
7572	7624	7502	8003	7667	7489
7653	7681	7548	7893	7735	7517
7653	7681	7544	7883	7735	7515
7804	7853	7700	8072	7907	7674
7804	7853	7700	8072	7907	7674
7809	7858	7705	8081	7912	7681
7841	7895	7739	8114	7944	7731
7815	7861	7699	8052	7897	7673
7659	7697	7561	7825	7727	7543
7662	7700	7564	7828	7730	7546
7667	7705	7571	7836	7735	7553
7672	7708	7563	7811	7732	7540
7638	7678	7545	7856	7708	7526
7646	7686	7553	7864	7716	7534
7637	7677	7544	7855	7707	7525
7711	7751	7605	7863	7779	7583
7713	7753	7607	7865	7781	7585
7717	7757	7611	7870	7785	7589
7730	7770	7624	7882	7798	7602
7632	7671	7576	7869	7686	7563
7633	7672	7577	7870	7687	7562
7633	7672	7577	7870	7687	7564
7634	7673	7578	7871	7688	7565
7632	7671	7576	7869	7686	7563
7634	7673	7578	7871	7688	7565
7683	7726	7583	7892	7723	7557
7682	7725	7582	7891	7722	7556
7681	7724	7581	7890	7721	7555
7680	7723	7580	7889	7720	7554
7682	7725	7582	7891	7722	7556
7681	7724	7581	7890	7721	7555
7658	7701	7560	7917	7698	7538
7684	7727	7584	7893	7724	7558
7747	7789	7671	7908	7821	7606

7739	7779	7635	8070	7802	7667
7742	7782	7638	8075	7805	7670
7740	7780	7636	8073	7803	7668
7737	7777	7633	8068	7800	7665
25028	25047	25010	25056	25037	24912
25027	25046	25009	25055	25036	24911
31004	31008	30968	30813	31030	30912

MOD1-EC5070	MOD1-EC6716	MOD1-EC6835	MOD1-EC6847	MOD1-EC6868	MOD1-EC6870
3744	4181	3593	3588	7576	3793
3692	4150	3516	3528	7508	3726
3692	4148	3516	3526	7510	3726
3747	4175	3559	3558	7533	3771
3706	4156	3528	3533	7555	3752
3730	4178	3552	3561	7514	3788
3730	4178	3552	3561	7514	3788
3747	4189	3569	3583	7549	3781
3750	4193	3572	3582	7555	3783
3784	4230	3612	3587	7501	3812
3762	4217	3627	3624	7511	3807
3762	4215	3592	3588	7549	3801
3860	4279	3711	3709	7610	3894
3860	4279	3711	3709	7610	3894
3803	4214	3621	3638	7560	3824
3800	4211	3618	3635	7566	3821
3675	4158	3509	3514	7527	3706
3716	4191	3560	3553	7557	3759
3791	4216	3591	3601	7562	3803
3806	4258	3622	3619	7548	3819
3817	4256	3663	3657	7571	3812
3722	4154	3553	3550	7546	3698
3757	4213	3621	3615	7527	3800
3770	4223	3635	3639	7553	3834
3671	4129	3514	3516	7515	3709
3687	4153	3572	3577	7576	3740
3641	4179	3514	3565	7591	3702
3740	4185	3639	3700	7591	3824
3739	4203	3625	3636	7547	3794
3669	4156	3553	3579	7542	3744
3669	4156	3553	3579	7542	3744
3673	4158	3557	3582	7545	3746
3668	4144	3566	3604	7568	3740
3639	4175	3520	3565	7579	3694
3639	4175	3520	3565	7579	3694
2236	3501	1863	1956	7624	1939
2227	3496	1854	1948	7619	1946
2231	3486	1844	1936	7621	1938
2190	3468	1841	1975	7626	1941
2229	3478	1846	1930	7604	1916
2233	3478	1856	1944	7618	1940
2232	3477	1853	1943	7617	1939
2225	3458	1842	1926	7616	1921
2236	3492	1870	1966	7613	1962
2241	3467	1900	1986	7593	1972
2291	3549	1957	2038	7564	2062
2138	3551	1942	1924	7605	2011
2088	3497	1728	1851	7586	1913
2083	3492	1723	1846	7583	1908
2089	3498	1729	1852	7589	1914
2083	3492	1723	1846	7583	1908
2088	3497	1728	1851	7588	1913
1993	3418	1504	1787	7625	1916

2151	3530	1777	2071	7672	1968
2013	3524	541	1750	7626	1808
2012	3523	540	1749	7625	1807
2005	3516	533	1742	7620	1800
2007	3518	535	1744	7622	1802
2003	3523	462	1758	7606	1756
2012	3532	471	1767	7615	1765
2012	3532	471	1767	7615	1765
1956	3535	329	1687	7581	1747
1973	3556	366	1706	7588	1770
1949	3553	372	1726	7563	1776
1948	3527	321	1679	7573	1739
1978	3607	27	1729	7604	1754
1978	3607	27	1729	7604	1754
1967	3597		1718	7593	1743
2015	3578	307	1700	7591	1727
1864	3551	1503	1889	7588	1808
1895	3582	1534	1920	7617	1839
1866	3553	1505	1891	7590	1810
1864	3551	1503	1889	7588	1808
1597	3801	1904	1892	7660	2257
1599	3803	1906	1894	7662	2259
1593	3797	1900	1888	7656	2253
1590	3794	1897	1885	7653	2250
1596	3788	1901	1889	7662	2232
1588	3792	1895	1883	7655	2248
1592	3796	1899	1887	7655	2252
1607	3803	1888	1874	7656	2245
1592	3796	1899	1887	7655	2252
1591	3795	1898	1886	7654	2251
1522	3778	1917	1881	7655	2264
1551	3780	1958	1902	7668	2227
1634	3779	1901	1885	7637	2258
1634	3779	1901	1885	7637	2258
1634	3779	1901	1885	7637	2258
303	3662	2052	1941	7715	2197
	3685	1967	1871	7716	2118
1907	3638	1750	98	7627	1882
1889	3626	1734	74	7625	1884
1889	3626	1734	74	7625	1884
1871	3609	1718		7622	1874
1902	3630	1836	1333	7619	1980
1550	3676	1962	2048	7684	2202
1588	3677	2106	2169	7708	2330
1585	3673	2155	2162	7726	2415
1585	3673	2155	2162	7726	2415
1638	3644	2150	2185	7727	2392
1566	3643	2122	2124	7726	2364
1467	3677	2069	2104	7694	2354
1692	3700	2040	2145	7708	2342
1668	3802	2134	2016	7770	2340
1721	3806	2139	2018	7762	2333
2047	3350	1798	1997	7578	1860
2060	3365	1809	2010	7599	1885
1995	3327	1726	1917	7589	1809

1999	3329	1728	1919	7591	1811
2023	3360	1746	1965	7595	1829
2048	3323	1752	2047	7569	1866
2028	3342	1838	1973	7598	1916
2028	3341	1838	1973	7600	1916
2028	3278	1795	2045	7613	1902
2030	3280	1797	2047	7615	1904
2030	3280	1797	2047	7615	1904
2055	3287	1820	2071	7619	1911
2031	3275	1802	2044	7612	1901
2057	3250	1810	2034	7596	1931
2058	3251	1811	2035	7597	1932
1989	3373	1714	1835	7609	1788
1981	3367	1708	1827	7607	1784
2037	3546	1867	1964	7656	1959
2037	3548	1869	1966	7658	1961
2029	3538	1856	1956	7648	1951
2028	3537	1858	1955	7647	1950
2028	3535	1878	1979	7658	1976
2028	3537	1858	1955	7647	1950
2029	3538	1859	1956	7648	1951
2043	3552	1873	1970	7660	1965
2077	3574	1899	2001	7652	1957
2063	3561	1909	2034	7634	1971
2244	3718	1927	1951	7716	681
2258	3719	1935	1966	7723	699
2260	3734	1943	1969	7733	699
2215	3685	1916	1944	7718	680
2115	3648	1740	1830	7715	482
2120	3641	1731	1819	7712	451
2120	3641	1731	1819	7712	451
2118	3639	1729	1817	7709	449
2124	3657	1743	1835	7716	463
2094	3619	1727	1822	7714	467
2096	3623	1715	1807	7726	473
2189	3659	1777	1883	7689	549
2125	3625	1794	1880	7703	490
2102	3615	1713	1807	7725	403
2130	3616	1718	1826	7743	479
2125	3636	1736	1834	7743	438
2130	3641	1741	1839	7745	443
2097	3636	1744	1838	7726	484
2125	3640	1734	1828	7719	406
2120	3633	1729	1823	7710	395
2051	3635	1672	1797	7688	256
2055	3644	1673	1805	7694	265
2224	3736	1865	1992	7668	355
2233	3745	1874	2001	7677	364
2217	3729	1858	1985	7661	348
2118	3691	1743	1874	7676	
2068	3639	1680	1813	7693	271
2068	3642	1681	1814	7694	271
2325	3736	1927	2091	7629	653
2169	3655	1801	1956	7672	419
2170	3656	1800	1959	7663	422

2180	3665	1810	1969	7674	440
2183	3669	1813	1972	7679	435
2191	3721	1812	1956	7677	428
2136	3692	1749	1888	7695	355
2129	3685	1742	1881	7683	348
2127	3683	1740	1879	7681	346
2075	3653	1690	1817	7686	276
2061	3645	1676	1808	7683	263
2091	3671	1708	1835	7705	298
2082	3666	1699	1826	7698	289
1998	3599	1616	1763	7673	450
1998	3599	1616	1763	7673	450
2105	3667	1721	1861	7684	280
2100	3663	1717	1856	7679	275
2103	3666	1720	1859	7682	278
2020	3463	1930	1874	7563	1882
2021	3464	1931	1875	7560	1883
2008	3451	1918	1862	7550	1870
2208	3678	2068	2065	7616	2013
2023	3518	1923	1902	7556	1856
2233	3744	2203	2170	7785	2174
3944	4438	3774	3852	7777	3923
6734	6886	6649	6608	7786	6714
6736	6888	6651	6610	7788	6716
6733	6885	6648	6607	7785	6713
6735	6887	6650	6609	7787	6715
6795	6955	6710	6658	7783	6774
6782	6948	6701	6646	7779	6763
6786	6948	6703	6652	7785	6757
6818	6920	6741	6694	7768	6788
6832	6934	6755	6708	7785	6802
6813	6925	6736	6689	7770	6763
6858	6938	6773	6724	7787	6830
6808	6910	6725	6678	7761	6782
6838	6940	6755	6708	7790	6812
6789	6909	6708	6661	7752	6770
6806	6906	6727	6676	7763	6778
6814	6916	6731	6684	7767	6788
6955	7053	6870	6839	7882	6947
6950	7048	6865	6834	7877	6942
6915	7007	6834	6801	7869	6916
6797	6930	6748	6701	7794	6800
6928	7011	6852	6821	7892	6923
6814	6904	6737	6708	7777	6808
6808	6898	6731	6702	7773	6802
6807	6895	6730	6701	7774	6799
6779	6897	6702	6675	7743	6798
6837	6949	6744	6722	7668	6853
3696	25	3608	3620	7824	3702
3685		3597	3609	7811	3691
7648	7781	7550	7578	3502	7687
7648	7781	7550	7578	3502	7687
7647	7780	7549	7577	3501	7686
7646	7779	7548	7576	3501	7685
7648	7779	7550	7578	3501	7687

7646	7779	7548	7576	3501	7685
7648	7781	7550	7578	3501	7687
7646	7779	7548	7576	3499	7685
7649	7782	7551	7579	3502	7688
7646	7779	7548	7576	3499	7685
7645	7778	7547	7575	3498	7684
7646	7779	7548	7576	3499	7685
7647	7780	7549	7577	3500	7686
7647	7780	7549	7577	3500	7686
7772	7864	7684	7690	3367	7769
7776	7868	7688	7694	3371	7773
7776	7867	7688	7694	3371	7773
7779	7870	7691	7697	3374	7776
7727	7836	7639	7651	3429	7730
7773	7865	7685	7691	3368	7770
7764	7859	7676	7682	3378	7759
7762	7857	7674	7680	3376	7757
7767	7862	7679	7685	3380	7762
7762	7857	7674	7680	3375	7757
7764	7859	7676	7682	3377	7759
7768	7863	7680	7686	3377	7764
7635	7786	7539	7545	3462	7624
7667	7765	7586	7594	3593	7684
7667	7773	7584	7596	3649	7684
7804	7883	7712	7745	3378	7832
7804	7883	7712	7745	3378	7832
7809	7888	7717	7750	3386	7837
7844	7927	7754	7777	3404	7869
7826	7919	7732	7769	3358	7842
7685	7753	7605	7616	2643	7668
7688	7756	7608	7619	2646	7671
7695	7766	7613	7624	2650	7676
7699	7761	7603	7616	2656	7679
7664	7745	7584	7596	2556	7647
7672	7753	7592	7604	2564	7655
7663	7744	7583	7595	2555	7646
7728	7812	7646	7670	2580	7718
7730	7814	7648	7672	2582	7720
7734	7818	7652	7676	2587	7724
7747	7831	7665	7689	2599	7737
7688	7759	7600	7585	2625	7643
7689	7760	7601	7586	2626	7644
7689	7760	7601	7586	2626	7644
7690	7761	7602	7587	2627	7645
7688	7759	7600	7585	2625	7643
7690	7761	7602	7586	2625	7645
7716	7811	7593	7622		7676
7715	7810	7592	7621	7	7675
7714	7809	7591	7620	6	7674
7713	7808	7590	7619	5	7673
7715	7810	7592	7621	7	7675
7714	7809	7591	7620	6	7674
7691	7840	7570	7597	43	7651
7717	7812	7594	7623	13	7677
7792	7852	7690	7685	2661	7771

7775	7882	7678	7697	2599	7745
7778	7884	7681	7700	2602	7748
7776	7883	7679	7698	2600	7746
7773	7880	7676	7696	2600	7743
24987	25018	25015	24983	25524	25027
24986	25017	25014	24982	25523	25026
30955	30966	30922	30961	31234	31023

MOD1-EC6885	MOD1-EC6891	MOD1-EC6897	MOD1-EC6802	MOD1-EC6938	MOD1-EC6943
3759	7554	902	836	3590	3760
3697	7495	428	195	3563	3666
3697	7497	430	197	3563	3666
3744	7531	462	314	3592	3726
3733	7553	447		3577	3704
3761	7518	441	173	3599	3733
3761	7518	441	173	3599	3733
3762	7540	440	200	3618	3719
3764	7544	442	202	3621	3721
3800	7494	470	383	3640	3761
3805	7504	606	515	3662	3767
3784	7543	475	250	3634	3757
3880	7596	195	593	3727	3851
3880	7596	195	593	3727	3851
3810	7547		447	3656	3779
3807	7552	40	445	3654	3778
3688	7516	261	361	3536	3697
3733	7534	293	418	3583	3716
3785	7535	315	467	3617	3786
3790	7519	516	616	3615	3730
3830	7550	412	523	3684	3811
3678	7528	793	739	3564	3668
3786	7527	585	558	3649	3760
3818	7556	419	496	3669	3781
3693	7505	614	451	3548	3698
3727	7571	583	567	3608	3691
3662	7578	1178	1150	3554	3670
3783	7573	1263	1292	3694	3749
3776	7520	1000	1004	3645	3770
3720	7523	877	809	3568	3706
3720	7523	877	809	3568	3706
3722	7526	880	812	3571	3708
3692	7564	947	889	3571	3703
3646	7570	1172	1148	3544	3666
3646	7570	1172	1148	3544	3666
1914	7614	3576	3513	1859	1939
1925	7613	3584	3505	1850	1964
1915	7613	3592	3507	1842	1953
1916	7618	3566	3473	1859	1962
1891	7597	3565	3492	1826	1921
1917	7615	3576	3497	1844	1933
1916	7614	3575	3496	1843	1932
1896	7611	3583	3495	1837	1964
1935	7607	3581	3502	1866	1967
1961	7566	3586	3529	1900	1941
2039	7564	3580	3512	1977	2012
1974	7606	3652	3558	1873	2041
1851	7556	3466	3377	1771	1954
1846	7553	3461	3372	1766	1949
1852	7559	3467	3378	1772	1955
1846	7553	3461	3372	1766	1949
1851	7558	3466	3377	1771	1954
1805	7610	3616	3539	1669	1969

1930	7660	3689	3588	1848	1988
1798	7596	3604	3511	1692	1885
1797	7595	3603	3510	1691	1884
1790	7588	3598	3504	1686	1877
1792	7590	3600	3507	1688	1879
1744	7597	3659	3566	1670	1851
1753	7606	3667	3575	1679	1860
1753	7606	3668	3575	1679	1860
1715	7579	3631	3538	1621	1824
1740	7588	3654	3561	1642	1849
1740	7561	3615	3522	1646	1819
1707	7571	3623	3530	1613	1816
1726	7600	3632	3539	1719	1811
1726	7600	3632	3539	1719	1811
1715	7592	3621	3528	1708	1800
1683	7595	3634	3539	1667	1780
1814	7583	3616	3535	1619	1875
1845	7612	3647	3566	1650	1906
1816	7585	3618	3537	1621	1877
1814	7583	3616	3535	1619	1875
2209	7617	3783	3712	2078	2323
2211	7619	3786	3715	2080	2325
2205	7613	3780	3709	2074	2319
2202	7610	3777	3706	2071	2316
2184	7619	3789	3700	2071	2307
2200	7612	3781	3704	2069	2314
2204	7612	3779	3708	2073	2318
2213	7613	3786	3715	2088	2327
2204	7612	3779	3708	2073	2318
2203	7611	3778	3707	2072	2317
2214	7608	3757	3684	2111	2317
2201	7625	3770	3713	2076	2309
2210	7605	3723	3646	2073	2324
2210	7605	3723	3646	2073	2324
2210	7605	3723	3646	2073	2324
2163	7671	3785	3710	2077	2233
2096	7672	3803	3706	1981	2170
1823	7609	3639	3558	1827	1973
1815	7608	3637	3540	1815	1967
1815	7608	3637	3540	1815	1967
1807	7604	3638	3533	1827	1959
1966	7609	3631	3529	1810	2014
2165	7641	3681	3621	1896	2267
2283	7680	3729	3668	2000	2395
2308	7690	3724	3663	2019	2464
2308	7690	3724	3663	2019	2464
2311	7695	3717	3637	2054	2456
2277	7697	3706	3642	2028	2419
2265	7655	3749	3684	2055	2411
2286	7663	3815	3755	2191	2412
2345	7739	3765	3685	2127	2396
2326	7731	3765	3685	2136	2393
1914	7581	3598	3545	836	1858
1927	7602	3612	3565	851	1868
1853	7589	3631	3560	735	1880

1857	7591	3631	3560	737	1882
1865	7599	3631	3560	707	1904
1916	7563	3609	3542	717	1939
1946	7591	3637	3581	603	2013
1946	7593	3637	3581	603	2013
1966	7602	3648	3569	639	1991
1968	7604	3650	3571	641	1993
1968	7602	3650	3571	641	1993
1983	7598	3660	3592	660	2003
1961	7602	3649	3568	628	1994
1967	7581	3629	3542	698	2022
1968	7582	3630	3543	699	2023
1792	7613	3646	3583	28	1873
1786	7614	3656	3577		1879
1916	7629	3607	3529	1879	1981
1918	7631	3607	3529	1881	1983
1908	7621	3599	3521	1871	1973
1907	7620	3598	3520	1870	1972
1927	7633	3600	3510	1898	1960
1907	7620	3598	3520	1870	1972
1908	7621	3599	3521	1871	1973
1922	7633	3613	3535	1885	1987
1940	7618	3588	3527	1909	2004
1930	7613	3626	3552	1921	1993
339	7711	3858	3763	1986	738
363	7716	3860	3765	2006	744
355	7726	3872	3785	2004	756
332	7702	3881	3794	1949	733
95	7694	3781	3706	1809	502
90	7692	3770	3704	1812	479
90	7692	3770	3704	1812	479
88	7689	3768	3702	1810	477
87	7696	3774	3714	1820	485
68	7675	3809	3739	1778	474
	7700	3810	3733	1786	457
202	7656	3865	3782	1881	597
247	7670	3794	3724	1869	425
134	7698	3806	3731	1786	399
198	7701	3826	3755	1841	441
155	7703	3827	3758	1811	416
160	7705	3832	3763	1816	421
209	7686	3817	3748	1821	426
183	7696	3777	3709	1817	436
172	7687	3762	3704	1808	427
273	7663	3797	3704	1715	250
280	7666	3797	3712	1727	251
520	7619	3850	3840	1916	389
529	7628	3859	3849	1925	398
513	7612	3843	3833	1909	382
473	7655	3824	3752	1784	422
290	7664	3797	3718	1729	245
290	7665	3802	3721	1730	247
705	7550	3857	3773	1998	582
462	7650	3788	3699	1878	23
457	7638	3779	3704	1879	

475	7647	3800	3696	1889	24
470	7654	3795	3717	1892	19
461	7641	3819	3762	1865	183
374	7670	3818	3739	1806	157
367	7658	3808	3723	1799	132
365	7656	3806	3721	1797	130
299	7659	3781	3720	1739	246
288	7646	3796	3712	1731	250
317	7684	3821	3721	1753	272
308	7677	3814	3714	1746	263
457	7649	3798	3701	1606	480
457	7649	3798	3701	1606	480
339	7662	3786	3711	1775	248
334	7657	3781	3706	1770	243
337	7660	3784	3709	1773	246
1933	7531	3573	3458	1761	1946
1934	7528	3575	3460	1762	1947
1921	7519	3563	3448	1749	1934
2058	7598	3717	3632	1992	2082
1893	7521	3591	3495	1823	1902
2173	7763	3789	3708	2010	2214
3914	7713	3417	3420	3877	3871
6753	7778	6635	6613	6687	6713
6755	7780	6637	6615	6689	6715
6752	7777	6634	6612	6686	6712
6754	7779	6636	6614	6688	6714
6818	7739	6772	6755	6756	6781
6811	7745	6760	6744	6743	6771
6819	7754	6772	6748	6751	6781
6825	7734	6810	6788	6779	6787
6839	7751	6824	6802	6793	6801
6806	7738	6791	6781	6776	6766
6865	7749	6866	6844	6803	6827
6817	7727	6790	6768	6771	6779
6847	7756	6819	6797	6801	6809
6818	7718	6781	6759	6758	6780
6815	7729	6796	6774	6769	6777
6823	7733	6796	6774	6777	6785
6995	7843	6985	6981	6914	6979
6990	7838	6980	6976	6909	6974
6949	7822	6938	6940	6880	6917
6858	7757	6811	6783	6798	6816
6961	7864	6833	6912	6910	6922
6839	7732	6804	6748	6788	6791
6833	7726	6798	6742	6782	6785
6832	7727	6803	6747	6781	6784
6801	7692	6784	6734	6753	6753
6844	7623	6831	6780	6803	6807
3634	7766	4225	4167	3378	3667
3623	7753	4214	4156	3367	3656
7725	3373	7534	7523	7569	7673
7725	3373	7534	7523	7569	7673
7724	3372	7533	7522	7568	7672
7723	3372	7532	7521	7567	7671
7725	3372	7534	7523	7569	7673

7723	3370	7532	7521	7567	7671
7725	3372	7534	7523	7569	7673
7723	3370	7532	7521	7567	7671
7726	3373	7535	7524	7570	7674
7723	3370	7532	7521	7567	7671
7722	3369	7531	7520	7566	7670
7723	3370	7532	7521	7567	7671
7724	3371	7533	7522	7568	7672
7724	3371	7533	7522	7568	7672
7811	3240	7611	7615	7710	7761
7815	3244	7615	7619	7714	7765
7815	3244	7615	7619	7714	7765
7818	3247	7618	7622	7717	7768
7772	3309	7552	7548	7667	7734
7812	3241	7612	7616	7711	7762
7801	3241	7605	7609	7703	7751
7799	3239	7603	7607	7701	7749
7804	3243	7608	7612	7706	7754
7799	3238	7603	7607	7701	7749
7801	3240	7604	7608	7703	7751
7806	3240	7610	7614	7708	7756
7647	3328	7558	7497	7559	7668
7727	3416	7455	7511	7623	7682
7727	3457	7453	7509	7637	7682
7879	3352	7686	7682	7778	7826
7879	3352	7686	7682	7778	7826
7884	3360	7695	7689	7783	7831
7914	3378	7745	7739	7812	7863
7888	3334	7689	7683	7787	7829
7711	292	7558	7558	7633	7649
7714	295	7561	7561	7636	7652
7719	283	7568	7568	7641	7657
7724	510	7555	7547	7633	7664
7692	20	7539	7545	7606	7630
7700		7547	7553	7614	7638
7691	19	7538	7544	7605	7629
7765	650	7598	7616	7679	7703
7765	652	7600	7618	7681	7705
7771	657	7604	7622	7685	7709
7784	669	7617	7635	7698	7722
7683	2312	7578	7595	7590	7626
7684	2313	7577	7594	7591	7627
7684	2313	7579	7596	7591	7627
7685	2314	7580	7597	7592	7628
7683	2312	7578	7595	7590	7626
7685	2310	7580	7597	7592	7628
7726	2564	7560	7555	7607	7663
7725	2563	7559	7554	7606	7662
7724	2562	7558	7553	7605	7661
7723	2561	7557	7552	7604	7660
7725	2563	7559	7554	7606	7662
7724	2562	7558	7553	7605	7661
7701	2594	7541	7536	7582	7638
7727	2565	7561	7556	7608	7664
7801	2544	7623	7632	7715	7744

7782	2364	7676	7679	7680	7712
7785	2367	7679	7682	7683	7715
7783	2365	7677	7680	7681	7713
7780	2364	7674	7677	7678	7710
25058	25523	24917	24904	25029	25017
25057	25522	24916	24903	25028	25016
31016	31206	30927	30890	31001	30995

MOD1-EC6946	MOD1-EC6966	MOD1-EC6978	MOD1-EC6529	MOD1-EC6332	MOD1-EC6385
7574	834	834	7545	3523	3583
7506	192	192	7486	3504	3533
7508	192	192	7488	3502	3533
7531	320	320	7522	3540	3559
7553	173	173	7544	3520	3560
7512			7509	3551	3583
7512			7509	3551	3583
7547	141	141	7531	3557	3589
7553	143	143	7535	3559	3592
7499	328	328	7485	3585	3611
7509	458	458	7495	3610	3632
7547	195	195	7534	3577	3609
7608	599	599	7587	3663	3702
7608	599	599	7587	3663	3702
7558	441	441	7538	3598	3631
7564	451	451	7543	3595	3628
7525	367	367	7507	3486	3497
7555	424	424	7525	3527	3548
7560	449	449	7526	3592	3607
7546	576	576	7510	3637	3613
7569	529	529	7541	3645	3652
7544	741	741	7519	3513	3511
7525	559	559	7518	3590	3619
7551	507	507	7547	3617	3644
7513	474	474	7496	3506	3519
7574	565	565	7562	3555	3586
7589	1157	1157	7569	3504	3543
7589	1295	1295	7564	3642	3664
7545	1009	1009	7511	3614	3624
7540	806	806	7514	3539	3569
7540	806	806	7514	3539	3569
7543	809	809	7517	3541	3572
7566	890	890	7555	3563	3548
7577	1153	1153	7561	3506	3543
7577	1153	1153	7561	3506	3543
7622	3543	3543	7605	1945	1846
7617	3529	3529	7604	1936	1843
7619	3535	3535	7604	1916	1831
7624	3501	3501	7609	1955	1860
7602	3507	3507	7588	1918	1824
7616	3525	3525	7606	1930	1845
7615	3524	3524	7605	1929	1844
7614	3523	3523	7602	1927	1838
7611	3538	3538	7598	1946	1857
7591	3557	3557	7557	1954	1881
7562	3543	3543	7555	2028	1975
7603	3576	3576	7597	1964	1916
7584	3403	3403	7547	1741	1739
7581	3398	3398	7544	1736	1734
7587	3404	3404	7550	1742	1740
7581	3398	3398	7544	1736	1734
7586	3403	3403	7549	1741	1739
7623	3561	3561	7601	1922	1700

7670	3616	3616	7651	1955	1737
7624	3535	3535	7587	1886	1740
7623	3534	3534	7586	1885	1739
7618	3529	3529	7579	1878	1734
7620	3531	3531	7581	1880	1736
7604	3590	3590	7588	1878	1700
7613	3599	3599	7597	1887	1709
7613	3599	3599	7597	1887	1709
7579	3562	3562	7570	1849	1701
7586	3585	3585	7579	1872	1722
7561	3546	3546	7552	1856	1737
7571	3554	3554	7562	1841	1693
7602	3563	3563	7591	1869	1739
7602	3563	3563	7591	1869	1739
7591	3552	3552	7583	1858	1728
7589	3563	3563	7586	1862	1745
7586	3558	3558	7574	1857	1778
7615	3589	3589	7603	1888	1809
7588	3560	3560	7576	1859	1780
7586	3558	3558	7574	1857	1778
7658	3724	3724	7608	2113	2053
7660	3727	3727	7610	2115	2055
7654	3721	3721	7604	2109	2049
7651	3718	3718	7601	2106	2046
7660	3722	3722	7610	2100	2056
7653	3718	3718	7603	2104	2044
7653	3720	3720	7603	2108	2048
7654	3727	3727	7604	2121	2063
7653	3720	3720	7603	2108	2048
7652	3719	3719	7602	2107	2047
7653	3696	3696	7599	2080	2058
7666	3725	3725	7616	2145	2109
7635	3658	3658	7596	2055	2048
7635	3658	3658	7596	2055	2048
7635	3658	3658	7596	2055	2048
7713	3734	3734	7662	2065	2083
7714	3730	3730	7663	2028	1999
7625	3578	3578	7600	1969	1951
7623	3562	3562	7599	1949	1941
7623	3562	3562	7599	1949	1941
7620	3561	3561	7595	1955	1919
7617	3541	3541	7600	1859	1842
7682	3639	3639	7632	2190	1837
7706	3687	3687	7671	2202	1957
7724	3678	3678	7681	2227	1990
7724	3678	3678	7681	2227	1990
7725	3666	3666	7686	2292	2003
7724	3657	3657	7688	2261	1983
7692	3695	3695	7646	2155	2009
7706	3770	3770	7654	2193	2096
7768	3698	3698	7730	2235	2180
7760	3698	3698	7722	2244	2217
7576	3572	3572	7572	1852	135
7597	3582	3582	7593	1863	144
7587	3583	3583	7580	1799	28

7589	3583	3583	7582	1801	
7593	3583	3583	7590	1809	76
7567	3579	3579	7554	1865	414
7596	3586	3586	7582	1876	425
7598	3586	3586	7584	1876	425
7611	3594	3594	7593	1909	475
7613	3596	3596	7595	1911	477
7613	3596	3596	7593	1911	477
7617	3617	3617	7589	1937	489
7610	3591	3591	7593	1914	480
7594	3569	3569	7572	1922	532
7595	3570	3570	7573	1923	533
7607	3605	3605	7604	1876	741
7605	3599	3599	7605	1870	737
7654	3560	3560	7620	33	1808
7656	3560	3560	7622	35	1810
7646	3552	3552	7612	11	1802
7645	3551	3551	7611		1801
7656	3541	3541	7624	58	1829
7645	3551	3551	7611	26	1803
7646	3552	3552	7612	25	1802
7658	3566	3566	7624	39	1816
7650	3556	3556	7609	76	1841
7632	3583	3583	7604	174	1853
7714	3805	3805	7702	2082	2043
7721	3807	3807	7707	2091	2063
7731	3821	3821	7717	2098	2059
7716	3830	3830	7693	2067	2022
7713	3738	3738	7685	1942	1886
7710	3738	3738	7683	1935	1881
7710	3738	3738	7683	1935	1881
7707	3736	3736	7680	1933	1879
7714	3746	3746	7687	1933	1885
7712	3771	3771	7666	1918	1862
7724	3761	3761	7691	1907	1857
7687	3816	3816	7647	1978	1944
7701	3760	3760	7661	1935	1914
7723	3767	3767	7689	1905	1859
7741	3787	3787	7692	1914	1890
7741	3790	3790	7694	1928	1882
7743	3795	3795	7696	1933	1887
7724	3774	3774	7677	1923	1880
7717	3745	3745	7687	1932	1882
7708	3738	3738	7678	1925	1877
7686	3738	3738	7654	1871	1772
7692	3746	3746	7657	1874	1772
7666	3876	3876	7610	2042	1955
7675	3885	3885	7619	2051	1964
7659	3869	3869	7603	2035	1948
7674	3788	3788	7646	1950	1811
7691	3754	3754	7655	1882	1772
7692	3757	3757	7656	1882	1773
7627	3807	3807	7541	2105	2001
7670	3732	3732	7641	1973	1887
7661	3733	3733	7629	1972	1882

7672	3743	3743	7638	1982	1892
7677	3748	3748	7645	1985	1895
7675	3765	3765	7632	2008	1898
7693	3771	3771	7661	1952	1837
7681	3752	3752	7649	1945	1827
7679	3750	3750	7647	1943	1825
7684	3747	3747	7650	1891	1780
7681	3750	3750	7637	1878	1773
7703	3742	3742	7675	1907	1803
7696	3735	3735	7668	1900	1796
7671	3741	3741	7640	1795	1668
7671	3741	3741	7640	1795	1668
7682	3740	3740	7653	1921	1811
7677	3735	3735	7648	1916	1806
7680	3738	3738	7651	1919	1809
7561	3484	3484	7522	1870	1779
7558	3486	3486	7519	1871	1780
7548	3474	3474	7510	1858	1767
7614	3654	3654	7589	2116	2010
7554	3507	3507	7512	1921	1815
7783	3722	3722	7754	2094	2006
7775	3429	3429	7705	3786	3830
7784	6615	6615	7769	6657	6677
7786	6617	6617	7771	6659	6679
7783	6614	6614	7768	6656	6676
7785	6616	6616	7770	6658	6678
7781	6755	6755	7730	6713	6747
7777	6744	6744	7736	6699	6741
7783	6750	6750	7745	6707	6743
7766	6790	6790	7725	6743	6767
7783	6804	6804	7742	6757	6781
7768	6797	6797	7729	6730	6762
7785	6846	6846	7740	6781	6793
7759	6770	6770	7718	6727	6759
7788	6799	6799	7747	6757	6789
7750	6761	6761	7709	6710	6746
7761	6776	6776	7720	6729	6763
7765	6776	6776	7724	6733	6765
7880	6983	6983	7834	6905	6896
7875	6978	6978	7829	6900	6891
7867	6942	6942	7813	6863	6863
7792	6785	6785	7748	6753	6784
7890	6914	6914	7855	6875	6886
7775	6751	6751	7723	6746	6771
7771	6745	6745	7717	6740	6765
7772	6750	6750	7718	6739	6764
7741	6730	6730	7683	6716	6738
7666	6779	6779	7614	6772	6781
7822	4189	4189	7757	3548	3340
7809	4178	4178	7744	3537	3329
3500	7490	7490	3364	7604	7559
3500	7490	7490	3364	7604	7559
3499	7489	7489	3363	7603	7558
3499	7488	7488	3363	7602	7557
3499	7490	7490	3363	7604	7559

3499	7488	7488	3361	7602	7557
3499	7490	7490	3363	7604	7559
3497	7488	7488	3361	7602	7557
3500	7491	7491	3364	7605	7560
3497	7488	7488	3361	7602	7557
3496	7487	7487	3360	7601	7556
3497	7488	7488	3361	7602	7557
3498	7489	7489	3362	7603	7558
3498	7489	7489	3362	7603	7558
3365	7582	7582	3231	7702	7696
3369	7586	7586	3235	7706	7700
3369	7586	7586	3235	7706	7700
3372	7589	7589	3238	7709	7703
3427	7515	7515	3300	7667	7653
3366	7583	7583	3232	7703	7697
3376	7576	7576	3232	7694	7688
3374	7574	7574	3230	7692	7686
3378	7579	7579	3234	7697	7691
3373	7574	7574	3229	7692	7686
3375	7575	7575	3231	7694	7688
3375	7581	7581	3231	7700	7693
3460	7464	7464	3319	7593	7571
3591	7474	7474	3407	7608	7594
3647	7472	7472	3448	7604	7604
3376	7647	7647	3343	7737	7741
3376	7647	7647	3343	7737	7741
3384	7654	7654	3351	7742	7746
3402	7704	7704	3369	7777	7780
3356	7650	7650	3325	7767	7761
2641	7537	7537	283	7638	7609
2644	7540	7540	286	7641	7612
2648	7547	7547	274	7646	7619
2654	7526	7526	501	7643	7612
2554	7510	7510	11	7612	7583
2562	7518	7518	19	7620	7591
2553	7509	7509		7611	7582
2578	7579	7579	641	7686	7657
2580	7581	7581	643	7688	7659
2585	7585	7585	648	7692	7663
2597	7598	7598	660	7705	7676
2623	7550	7550	2303	7634	7576
2624	7549	7549	2304	7635	7577
2624	7551	7551	2304	7635	7577
2625	7552	7552	2305	7636	7578
2623	7550	7550	2303	7634	7576
2623	7552	7552	2301	7636	7578
6	7514	7514	2555	7647	7591
5	7513	7513	2554	7646	7590
4	7512	7512	2553	7645	7589
3	7511	7511	2552	7644	7588
3	7513	7513	2554	7646	7590
	7512	7512	2553	7645	7589
41	7495	7495	2585	7622	7566
11	7515	7515	2556	7648	7592
2659	7589	7589	2535	7713	7697

2597	7636	7636	2355	7681	7655
2600	7639	7639	2358	7684	7658
2598	7637	7637	2356	7682	7656
2598	7634	7634	2355	7679	7653
25522	24925	24925	25515	25034	24985
25521	24924	24924	25514	25033	24984
31230	30900	30900	31198	30975	31010

MOD1-EC5700	MOD1-EC5706	MOD1-EC5440	MOD1-EC5737	MOD1-EC5722	MOD1-EC3793
993	1047	3605	3406	3765	3675
1142	459	3518	3364	3669	3612
1144	461	3518	3364	3669	3612
1186	533	3557	3400	3754	3642
1150	515	3530	3377	3739	3642
1157	458	3554	3403	3771	3657
1157	458	3554	3403	3771	3657
1150	393	3571	3417	3739	3667
1152	395	3574	3421	3741	3669
1233	191	3609	3443	3801	3696
1116		3624	3518	3782	3692
1170	412	3590	3440	3756	3678
1207	653	3713	3542	3879	3761
1207	653	3713	3542	3879	3761
1178	606	3623	3466	3809	3706
1168	606	3620	3462	3806	3703
1076	536	3497	3332	3689	3574
1153	603	3548	3381	3734	3625
1145	590	3591	3435	3762	3682
1326	734	3604	3436	3767	3724
1162	640	3631	3483	3807	3730
1179	681	3555	3431	3683	3638
1067	437	3619	3501	3789	3672
1084	357	3631	3527	3823	3698
977	456	3508	3408	3696	3594
1006	533	3572	3454	3732	3623
	1116	3494	3387	3667	3615
559	1217	3627	3511	3788	3712
699	858	3605	3502	3754	3678
642	794	3556	3438	3726	3602
642	794	3556	3438	3726	3602
645	797	3559	3441	3728	3606
564	853	3558	3402	3698	3603
62	1115	3500	3393	3652	3607
62	1115	3500	3393	3652	3607
3482	3614	1804	1794	1925	2334
3468	3606	1795	1785	1943	2307
3470	3618	1777	1767	1928	2320
3415	3540	1774	1806	1934	2283
3461	3581	1779	1771	1909	2309
3478	3611	1793	1779	1935	2321
3477	3610	1792	1778	1934	2320
3456	3595	1779	1755	1914	2313
3411	3603	1805	1793	1946	2338
3450	3581	1847	1823	1979	2335
3424	3556	1903	1869	2057	2412
3536	3618	1887	1849	1984	2384
3387	3518	1700	26	1848	2105
3382	3513	1695	21	1843	2100
3388	3519	1701	27	1849	2106
3382	3513	1695	21	1843	2100
3387	3518	1700		1848	2105
3516	3640	1511	1447	1795	2026

3597	3721	1702	1691	1934	2122
3473	3605	254	1743	1810	2157
3472	3604	253	1742	1809	2156
3467	3599	246	1735	1802	2151
3469	3601	248	1737	1804	2153
3542	3660	171	1696	1756	2133
3551	3669	180	1705	1765	2142
3551	3669	180	1705	1765	2142
3502	3632	14	1708	1727	2108
3525	3655	51	1729	1752	2125
3464	3612	67	1745	1752	2137
3494	3624		1700	1719	2100
3525	3638	332	1739	1738	2133
3525	3638	332	1739	1738	2133
3514	3627	321	1728	1727	2122
3519	3633	218	1681	1695	2146
3508	3635	1416	1616	1801	2008
3539	3666	1447	1647	1832	2039
3510	3637	1418	1618	1803	2010
3508	3635	1416	1616	1801	2008
3690	3761	1865	2131	2219	2016
3693	3764	1867	2133	2221	2018
3687	3758	1861	2127	2215	2012
3684	3755	1858	2124	2212	2009
3694	3762	1862	2114	2194	2049
3682	3755	1856	2122	2210	2017
3686	3757	1860	2126	2214	2011
3693	3764	1849	2141	2229	2000
3686	3757	1860	2126	2214	2011
3685	3756	1859	2125	2213	2010
3660	3753	1874	2132	2224	1965
3685	3746	1919	2173	2203	1999
3686	3757	1862	2032	2220	2020
3686	3757	1862	2032	2220	2020
3686	3757	1862	2032	2220	2020
3667	3768	2017	2105	2161	1435
3641	3762	1948	2088	2094	1566
3572	3622	1711	1883	1831	2148
3558	3621	1695	1865	1830	2136
3558	3621	1695	1865	1830	2136
3565	3624	1679	1851	1822	2124
3489	3626	1779	1820	1973	1957
3586	3666	1954	2055	2170	419
3640	3720	2084	2113	2288	174
3635	3717	2133	2102	2303	203
3635	3717	2133	2102	2303	203
3642	3704	2128	2143	2327	156
3615	3692	2100	2105	2293	
3638	3738	2064	2107	2276	321
3697	3802	2024	2082	2295	728
3643	3746	2097	2250	2354	1704
3633	3746	2102	2283	2333	1747
3552	3605	1775	1795	1916	2033
3562	3606	1786	1804	1932	2053
3543	3632	1691	1735	1858	1979

3543	3632	1693	1739	1862	1983
3535	3632	1709	1763	1870	1999
3562	3617	1701	1888	1899	2058
3542	3630	1779	1818	1936	2061
3542	3630	1779	1818	1936	2061
3593	3653	1727	1814	1953	2114
3595	3655	1729	1816	1955	2116
3595	3655	1729	1816	1955	2116
3615	3625	1752	1838	1942	2127
3602	3655	1734	1821	1953	2109
3580	3633	1746	1815	1959	2091
3581	3634	1747	1816	1960	2092
3556	3661	1619	1775	1777	2038
3554	3662	1613	1771	1778	2028
3513	3619	1850	1750	1927	2270
3513	3619	1850	1752	1929	2272
3505	3611	1839	1741	1919	2262
3504	3610	1841	1741	1918	2261
3512	3622	1861	1761	1938	2281
3504	3610	1841	1741	1918	2261
3505	3611	1842	1742	1919	2262
3519	3625	1856	1756	1933	2276
3508	3578	1882	1787	1923	2298
3530	3642	1900	1807	1941	2324
3712	3851	1919	2066	359	2449
3724	3853	1935	2071	383	2465
3728	3867	1935	2082	375	2467
3737	3876	1910	2037	320	2440
3627	3778	1732	1880	101	2300
3636	3773	1723	1873	99	2295
3636	3773	1723	1873	99	2295
3634	3771	1721	1871	97	2293
3642	3779	1735	1879	95	2309
3667	3782	1719	1848		2293
3662	3805	1707	1851	68	2277
3707	3859	1769	1933	223	2376
3605	3759	1800	1904	265	2340
3662	3801	1695	1811	152	2291
3698	3804	1710	1818	197	2316
3683	3807	1718	1836	154	2316
3688	3812	1723	1841	159	2321
3685	3789	1734	1856	208	2314
3631	3778	1716	1834	182	2316
3630	3771	1711	1829	171	2311
3645	3792	1664	1830	299	2307
3639	3793	1665	1831	301	2318
3755	3783	1875	2027	514	2466
3764	3792	1884	2036	523	2475
3748	3776	1868	2020	507	2459
3702	3807	1739	1913	467	2364
3645	3795	1672	1836	311	2320
3650	3798	1673	1837	311	2320
3664	3806	1947	2124	699	2573
3673	3771	1817	1957	484	2416
3670	3767	1816	1954	474	2419

3689	3791	1826	1964	492	2430
3684	3786	1829	1967	487	2433
3722	3742	1814	1963	454	2436
3673	3802	1741	1903	391	2389
3663	3781	1734	1896	384	2382
3661	3779	1732	1894	382	2380
3631	3785	1682	1846	316	2309
3644	3767	1668	1833	276	2319
3665	3786	1700	1864	343	2322
3658	3779	1691	1857	334	2317
3659	3801	1610	1830	481	2255
3659	3801	1610	1830	481	2255
3646	3775	1713	1876	352	2363
3641	3770	1709	1871	347	2358
3644	3773	1712	1874	350	2361
3498	3563	1873	1908	1931	2289
3499	3565	1874	1909	1932	2290
3486	3553	1861	1896	1919	2277
3648	3747	2022	2149	2049	2427
3514	3599	1877	2008	1907	2282
3723	3815	2140	2239	2190	2538
3453	3474	3764	3665	3913	3820
6646	6627	6650	6591	6753	6720
6648	6629	6652	6593	6755	6722
6645	6626	6649	6590	6752	6719
6647	6628	6651	6592	6754	6721
6785	6745	6711	6667	6795	6793
6777	6757	6702	6657	6806	6786
6783	6762	6704	6661	6819	6786
6821	6802	6742	6691	6825	6814
6835	6816	6756	6705	6839	6830
6793	6769	6737	6684	6806	6806
6877	6858	6774	6729	6865	6856
6801	6782	6726	6681	6817	6806
6831	6811	6756	6711	6847	6836
6790	6773	6709	6664	6818	6789
6805	6788	6728	6685	6815	6804
6807	6788	6732	6687	6823	6812
6978	6973	6873	6832	6995	6959
6973	6968	6868	6827	6990	6954
6943	6922	6837	6794	6949	6925
6803	6826	6749	6703	6858	6821
6923	6932	6857	6807	6961	6931
6790	6795	6740	6693	6839	6820
6784	6789	6734	6687	6833	6814
6787	6794	6733	6686	6832	6813
6763	6775	6703	6673	6801	6789
6812	6801	6751	6713	6821	6857
4190	4228	3538	3508	3630	3654
4179	4217	3527	3497	3619	3643
7552	7443	7521	7574	7694	7709
7552	7443	7521	7574	7694	7709
7551	7442	7520	7573	7693	7708
7550	7441	7519	7572	7692	7707
7552	7443	7521	7574	7694	7709

7550	7441	7519	7572	7692	7707
7552	7443	7521	7574	7694	7709
7550	7441	7519	7572	7692	7707
7553	7444	7522	7575	7695	7710
7550	7441	7519	7572	7692	7707
7549	7440	7518	7571	7691	7706
7550	7441	7519	7572	7692	7707
7551	7442	7520	7573	7693	7708
7551	7442	7520	7573	7693	7708
7634	7555	7661	7648	7780	7805
7638	7559	7665	7652	7784	7809
7638	7559	7665	7652	7784	7809
7641	7562	7668	7655	7787	7812
7575	7501	7620	7605	7741	7760
7635	7556	7662	7649	7781	7806
7626	7549	7653	7640	7770	7797
7624	7547	7651	7638	7768	7795
7629	7552	7656	7643	7773	7800
7624	7547	7651	7638	7768	7795
7625	7548	7653	7640	7770	7797
7631	7554	7657	7644	7775	7803
7530	7513	7507	7507	7616	7682
7536	7486	7560	7553	7696	7709
7536	7484	7558	7549	7696	7713
7717	7634	7685	7705	7848	7847
7717	7634	7685	7705	7848	7847
7724	7641	7690	7710	7853	7852
7768	7690	7723	7744	7883	7885
7713	7634	7699	7704	7857	7864
7582	7521	7584	7566	7686	7712
7585	7524	7587	7569	7689	7715
7593	7531	7592	7576	7694	7722
7584	7506	7582	7568	7699	7727
7570	7496	7563	7550	7667	7689
7578	7504	7571	7558	7675	7697
7569	7495	7562	7549	7666	7688
7638	7560	7625	7610	7740	7756
7640	7562	7627	7612	7742	7758
7644	7566	7631	7616	7746	7762
7657	7579	7644	7629	7759	7775
7587	7528	7576	7581	7660	7717
7588	7527	7577	7582	7661	7718
7588	7529	7577	7582	7661	7718
7589	7530	7578	7583	7662	7719
7587	7528	7576	7581	7660	7717
7589	7529	7578	7583	7662	7719
7591	7511	7573	7588	7714	7726
7590	7510	7572	7587	7713	7725
7589	7509	7571	7586	7712	7724
7588	7508	7570	7585	7711	7723
7590	7510	7572	7587	7713	7725
7589	7509	7571	7586	7712	7724
7572	7492	7550	7565	7689	7707
7592	7512	7574	7589	7715	7727
7646	7586	7672	7676	7774	7813

7706	7649	7656	7640	7773	7796
7709	7652	7659	7643	7776	7799
7707	7650	7657	7641	7774	7797
7704	7647	7654	7638	7771	7794
24946	24940	24998	25015	25042	24984
24945	24939	24997	25014	25041	24983
30918	30935	30920	30973	31011	30966

MOD1-EC54	MOD1-EC5522	MOD1-EC5194	MOD1-EC5196	Ecol_583	MOD1-EC3564	18.1-R1
7605	3808	7574	7573	3759	3572	6785
7554	3759	7506	7505	3677	3500	6721
7554	3759	7508	7507	3679	3500	6725
7597	3800	7531	7530	3705	3550	6754
7607	3782	7553	7552	3706	3545	6755
7574	3816	7512	7511	3718	3572	6755
7574	3816	7512	7511	3718	3572	6755
7582	3816	7547	7546	3734	3556	6757
7586	3818	7553	7552	3737	3559	6759
7554	3855	7499	7498	3751	3587	6764
7547	3859	7509	7508	3755	3605	6745
7586	3846	7547	7546	3735	3598	6769
7651	3935	7608	7607	3839	3671	6796
7651	3935	7608	7607	3839	3671	6796
7603	3865	7558	7557	3777	3598	6772
7607	3862	7564	7563	3774	3597	6784
7569	3741	7525	7524	3669	3491	6741
7585	3790	7555	7554	3704	3508	6724
7578	3841	7560	7559	3739	3611	6745
7567	3850	7546	7545	3782	3642	6770
7600	3862	7569	7568	3803	3616	6773
7585	3736	7544	7543	3683	3494	6731
7595	3837	7525	7524	3734	3594	6737
7610	3869	7551	7550	3749	3621	6795
7556	3742	7513	7512	3657	3539	6736
7611	3772	7574	7573	3702	3548	6768
7624	3707	7589	7588	3684	3552	6785
7622	3841	7589	7588	3782	3611	6782
7554	3834	7545	7544	3771	3629	6740
7568	3783	7540	7539	3689	3540	6739
7568	3783	7540	7539	3689	3540	6739
7571	3785	7543	7542	3692	3543	6740
7593	3757	7566	7565	3707	3530	6786
7615	3713	7577	7576	3679	3546	6780
7615	3713	7577	7576	3679	3546	6780
7715	2009	7622	7621	2214	1866	6668
7713	2016	7617	7616	2183	1861	6663
7714	1994	7619	7618	2189	1857	6663
7712	2015	7624	7623	2180	1880	6649
7698	1990	7602	7601	2183	1838	6657
7709	2016	7616	7615	2195	1835	6653
7710	2015	7615	7614	2194	1834	6652
7709	1995	7614	7613	2193	1863	6674
7712	2016	7611	7610	2210	1871	6688
7685	2060	7591	7590	2243	1886	6640
7664	2132	7562	7561	2296	1971	6662
7694	2073	7603	7602	2243	1950	6779
7634	1933	7584	7583	2124	1795	6667
7633	1928	7581	7580	2119	1790	6662
7639	1934	7587	7586	2125	1796	6668
7633	1928	7581	7580	2119	1790	6662
7638	1933	7586	7585	2124	1795	6667
7693	1879	7623	7622	2073	1768	6705

7745	2015	7670	7669	2260	1789	6825
7690	1860	7624	7623	1928	1822	6723
7688	1859	7623	7622	1927	1821	6722
7685	1852	7618	7617	1920	1816	6714
7687	1854	7620	7619	1922	1818	6717
7676	1806	7604	7603	1913	1782	6744
7685	1815	7613	7612	1922	1791	6753
7685	1815	7613	7612	1922	1791	6753
7659	1777	7579	7578	1866	1783	6719
7671	1802	7586	7585	1883	1804	6734
7644	1802	7561	7560	1896	1784	6709
7651	1769	7571	7570	1858	1775	6711
7684	1788	7602	7601	1908	1809	6721
7684	1788	7602	7601	1908	1809	6721
7674	1777	7591	7590	1897	1798	6710
7667	1745	7589	7588	1923	1817	6707
7664	1915	7586	7585	2107	1846	6782
7693	1946	7615	7614	2138	1877	6810
7666	1917	7588	7587	2109	1848	6784
7664	1915	7586	7585	2107	1846	6782
7710	2289	7658	7657	9	2105	6824
7712	2291	7660	7659	11	2107	6826
7706	2285	7654	7653	5	2101	6820
7703	2282	7651	7650		2098	6817
7714	2264	7660	7659	46	2114	6828
7705	2280	7653	7652	10	2104	6821
7705	2284	7653	7652	4	2100	6819
7710	2293	7654	7653	19	2115	6830
7705	2284	7653	7652	4	2100	6819
7704	2283	7652	7651	3	2099	6818
7707	2294	7653	7652	76	2098	6817
7714	2285	7666	7665	91	2151	6804
7697	2290	7635	7634	120	2100	6823
7697	2290	7635	7634	120	2100	6823
7697	2290	7635	7634	120	2100	6823
7765	2264	7713	7712	1676	2137	6797
7762	2189	7714	7713	1590	2047	6795
7681	1907	7625	7624	1905	1995	6661
7682	1891	7623	7622	1885	1995	6662
7682	1891	7623	7622	1885	1995	6662
7680	1883	7620	7619	1885	1997	6658
7681	2045	7617	7616	2024	1892	6781
7745	2246	7682	7681	1876	1887	6791
7782	2364	7706	7705	1997	2007	6776
7789	2391	7724	7723	1974	2042	6780
7789	2391	7724	7723	1974	2042	6780
7793	2408	7725	7724	2055	2073	6794
7795	2376	7724	7723	2009	2033	6793
7761	2348	7692	7691	1907	2065	6779
7756	2360	7706	7705	1918	2148	6752
7837	2423	7768	7767	1863	2208	6851
7836	2404	7760	7759	1916	2245	6862
7670	2001	7576	7575	2098		6735
7691	2014	7597	7596	2118	48	6752
7684	1940	7587	7586	2044	133	6744

7686	1944	7589	7588	2046	135	6747
7689	1952	7593	7592	2078	181	6745
7660	1999	7567	7566	2090	497	6763
7686	2037	7596	7595	2135	515	6769
7688	2037	7598	7597	2135	515	6771
7686	2041	7611	7610	2080	558	6792
7688	2043	7613	7612	2082	560	6794
7686	2043	7613	7612	2082	560	6794
7672	2073	7617	7616	2103	569	6778
7684	2056	7610	7609	2077	561	6790
7672	2058	7594	7593	2071	613	6806
7673	2059	7595	7594	2072	614	6807
7700	1883	7607	7606	2081	840	6755
7701	1881	7605	7604	2071	836	6756
7701	1987	7654	7653	2115	1859	6722
7703	1989	7656	7655	2115	1861	6722
7693	1979	7646	7645	2106	1853	6714
7692	1978	7645	7644	2106	1852	6713
7701	1998	7656	7655	2134	1844	6698
7692	1978	7645	7644	2106	1854	6713
7693	1979	7646	7645	2107	1853	6714
7705	1993	7658	7657	2121	1867	6728
7682	2022	7650	7649	2153	1887	6702
7674	2001	7632	7631	2154	1895	6763
7814	479	7714	7713	2364	2094	6756
7819	503	7721	7720	2378	2099	6763
7829	495	7731	7730	2382	2112	6771
7803	472	7716	7715	2345	2075	6755
7800	249	7713	7712	2221	1939	6812
7794	236	7710	7709	2228	1936	6813
7794	236	7710	7709	2228	1936	6813
7791	234	7707	7706	2226	1934	6810
7794	238	7714	7713	2236	1942	6817
7768	223	7712	7711	2212	1916	6795
7799	202	7724	7723	2202	1914	6818
7770		7687	7686	2282	2001	6836
7773	389	7701	7700	2251	1910	6813
7796	276	7723	7722	2198	1906	6809
7797	342	7741	7740	2221	1926	6825
7801	299	7741	7740	2223	1937	6817
7805	304	7743	7742	2228	1942	6823
7780	353	7724	7723	2215	1929	6801
7800	315	7717	7716	2225	1939	6816
7789	312	7708	7707	2222	1934	6807
7758	411	7686	7685	2179	1819	6795
7761	406	7692	7691	2190	1825	6797
7738	659	7666	7665	2388	1950	6735
7747	668	7675	7674	2397	1959	6744
7731	652	7659	7658	2381	1943	6728
7757	549	7674	7673	2250	1860	6774
7754	426	7691	7690	2200	1831	6788
7757	426	7692	7691	2200	1832	6789
7632	678	7627	7626	2456	2005	6776
7759	600	7670	7669	2311	1857	6791
7749	597	7661	7660	2316	1858	6781

7758	615	7672	7671	2327	1877	6786
7765	610	7677	7676	2330	1872	6797
7739	602	7675	7674	2300	1883	6705
7782	514	7693	7692	2269	1887	6817
7772	507	7681	7680	2262	1877	6810
7770	505	7679	7678	2260	1875	6808
7753	433	7684	7683	2183	1817	6792
7733	417	7681	7680	2194	1821	6769
7778	453	7703	7702	2196	1839	6817
7771	444	7696	7695	2189	1832	6810
7743	570	7671	7670	2101	1725	6767
7743	570	7671	7670	2101	1725	6767
7756	479	7682	7681	2241	1860	6793
7751	474	7677	7676	2236	1855	6788
7754	477	7680	7679	2239	1858	6791
7649	2023	7561	7560	2191	1849	6678
7646	2024	7558	7557	2192	1850	6677
7636	2011	7548	7547	2179	1837	6666
7732	2156	7614	7613	2352	2072	6774
7661	1995	7554	7553	2176	1876	6698
7885	2271	7783	7782	2449	2076	6939
7859	3979	7775	7774	3833	3820	7004
7939	6805	7784	7783	6754	6662	222
7941	6807	7786	7785	6756	6664	224
7938	6804	7783	7782	6753	6661	221
7940	6806	7785	7784	6755	6663	223
7910	6836	7781	7780	6817	6735	
7928	6826	7777	7776	6806	6728	26
7939	6834	7783	7782	6810	6726	37
7918	6813	7766	7765	6846	6752	119
7933	6827	7783	7782	6860	6766	135
7922	6790	7768	7767	6836	6724	175
7929	6833	7785	7784	6874	6778	161
7911	6807	7759	7758	6832	6744	105
7940	6837	7788	7787	6862	6774	135
7904	6807	7750	7749	6813	6731	98
7911	6805	7761	7760	6828	6748	105
7915	6813	7765	7764	6838	6750	111
8035	6948	7880	7879	6964	6900	442
8030	6943	7875	7874	6959	6895	437
8019	6919	7867	7866	6930	6851	442
7957	6845	7792	7791	6836	6771	290
8042	6952	7890	7889	6949	6877	460
7924	6830	7775	7774	6835	6753	276
7918	6824	7771	7770	6829	6747	270
7919	6827	7772	7771	6826	6746	273
7888	6796	7741	7740	6802	6717	379
7797	6842	7666	7665	6848	6764	512
7868	3670	7822	7821	3805	3361	6965
7857	3659	7809	7808	3794	3350	6955
260	7692	3500	3499	7574	7531	7875
260	7692	3500	3499	7574	7531	7875
259	7691	3499	3498	7573	7530	7874
259	7690	3499	3498	7572	7529	7873
259	7692	3499	3498	7574	7531	7875

259	7690	3499	3498	7572	7529	7875
259	7692	3499	3498	7574	7531	7875
257	7690	3497	3496	7572	7529	7873
260	7693	3500	3499	7575	7532	7876
257	7690	3497	3496	7572	7529	7873
256	7689	3496	3495	7571	7528	7872
257	7690	3497	3496	7572	7529	7873
258	7691	3498	3497	7573	7530	7874
258	7691	3498	3497	7573	7530	7874
74	7775	3365	3364	7711	7680	7918
78	7779	3369	3368	7715	7684	7922
78	7779	3369	3368	7715	7684	7922
81	7782	3372	3371	7718	7687	7925
175	7736	3427	3426	7672	7637	7920
75	7776	3366	3365	7712	7681	7919
17	7772	3376	3375	7705	7672	7912
15	7770	3374	3373	7703	7670	7911
19	7775	3378	3377	7708	7675	7915
	7770	3373	3372	7703	7670	7910
18	7772	3375	3374	7705	7672	7912
26	7777	3375	3374	7709	7677	7915
236	7614	3460	3459	7562	7569	7865
772	7683	3591	3590	7620	7585	7841
843	7690	3647	3646	7624	7595	7827
1879	7847	3376	3375	7755	7733	7944
1879	7847	3376	3375	7755	7733	7944
1886	7852	3384	3383	7760	7738	7951
1902	7879	3402	3401	7790	7776	7990
2012	7847	3356	3355	7778	7749	7924
3275	7664	2641	2640	7618	7599	7703
3278	7667	2644	2643	7621	7602	7706
3282	7675	2648	2647	7630	7609	7711
3305	7680	2654	2653	7625	7604	7688
3230	7648	2554	2553	7602	7573	7731
3238	7656	2562	2561	7610	7581	7739
3229	7647	2553	2552	7601	7572	7730
3277	7716	2578	2577	7678	7647	7736
3279	7718	2580	2579	7680	7649	7738
3284	7723	2585	2584	7684	7653	7743
3296	7735	2597	2596	7697	7666	7755
3297	7644	2623	2622	7630	7568	7739
3298	7645	2624	2623	7631	7569	7740
3298	7645	2624	2623	7631	7569	7740
3299	7646	2625	2624	7632	7570	7741
3297	7644	2623	2622	7630	7568	7739
3297	7646	2623	2622	7632	7570	7741
3375	7689	6	5	7653	7578	7783
3374	7688	5	4	7652	7577	7782
3373	7687		1	7651	7576	7781
3372	7686	1		7650	7575	7780
3374	7688	5	4	7652	7577	7782
3373	7687	4	3	7651	7576	7781
3405	7714	41	40	7628	7553	7764
3376	7690	11	10	7654	7579	7784
3383	7770	2659	2658	7722	7688	7794

3287	7746	2597	2596	7694	7644	7973
3290	7749	2600	2599	7697	7647	7978
3288	7747	2598	2597	7695	7645	7976
3285	7744	2598	2597	7692	7642	7971
25534	25044	25522	25521	24967	24972	25031
25533	25043	25521	25520	24966	24971	25030
31162	30999	31232	31231	30944	31006	30807

MOD1-EC3605	KCJK1916	KCJK1866	MOD1-EC707	MOD1-EC1626	MOD1-EC1634
871	3689	3689	7575	3719	3761
962	3617	3617	7507	3689	3696
964	3619	3619	7509	3689	3696
908	3645	3645	7532	3740	3742
1004	3646	3646	7554	3714	3721
1009	3658	3658	7513	3735	3757
1009	3658	3658	7513	3735	3757
971	3674	3674	7548	3736	3755
973	3677	3677	7554	3738	3757
999	3691	3691	7500	3778	3792
858	3757	3757	7510	3779	3798
984	3675	3675	7548	3756	3773
1029	3794	3794	7609	3884	3872
1029	3794	3794	7609	3884	3872
1000	3723	3723	7559	3814	3802
990	3720	3720	7565	3811	3799
904	3615	3615	7526	3684	3676
984	3650	3650	7556	3739	3727
969	3685	3685	7561	3792	3781
1136	3728	3728	7547	3812	3788
985	3749	3749	7570	3828	3821
1007	3661	3661	7545	3686	3676
890	3736	3736	7526	3785	3784
857	3751	3751	7552	3799	3806
878	3659	3659	7514	3702	3687
901	3704	3704	7575	3701	3723
699	3686	3686	7590	3658	3650
858	3784	3784	7590	3821	3815
	3773	3773	7546	3766	3753
474	3691	3691	7541	3688	3712
474	3691	3691	7541	3688	3712
477	3694	3694	7544	3690	3714
548	3709	3709	7567	3695	3708
692	3681	3681	7578	3652	3650
692	3681	3681	7578	3652	3650
3584	2236	2236	7623	1875	1853
3583	2205	2205	7618	1855	1856
3584	2211	2211	7620	1852	1840
3518	2228	2228	7625	1861	1849
3564	2205	2205	7603	1822	1834
3581	2217	2217	7617	1862	1850
3580	2216	2216	7616	1861	1849
3558	2215	2215	7615	1835	1823
3561	2232	2232	7612	1872	1862
3562	2265	2265	7592	1906	1894
3552	2324	2324	7563	1983	1982
3643	2275	2275	7604	1929	1935
3502	2032	2032	7585	1857	1837
3497	2027	2027	7582	1852	1832
3503	2033	2033	7588	1858	1838
3497	2027	2027	7582	1852	1832
3502	2032	2032	7587	1857	1837
3635	2095	2095	7624	1876	1860

3708	2186	2186	7671	1902	1886
3586	1936	1936	7625	1760	1742
3585	1935	1935	7624	1759	1741
3580	1928	1928	7619	1752	1734
3582	1930	1930	7621	1754	1736
3649	1923	1923	7605	1726	1708
3658	1932	1932	7614	1735	1717
3658	1932	1932	7614	1735	1717
3613	1870	1870	7580	1699	1681
3636	1887	1887	7587	1724	1706
3603	1900	1900	7562	1716	1698
3605	1862	1862	7572	1691	1673
3636	1912	1912	7603	1710	1692
3636	1912	1912	7603	1710	1692
3625	1901	1901	7592	1699	1681
3632	1927	1927	7590	1665	1647
3604	2139	2139	7587	1763	1746
3635	2170	2170	7616	1794	1777
3606	2141	2141	7589	1765	1748
3604	2139	2139	7587	1763	1746
3777	127	127	7659	2196	2207
3780	129	129	7661	2198	2209
3774	123	123	7655	2192	2203
3771	120	120	7652	2189	2200
3781	164	164	7661	2188	2186
3769	128	128	7654	2189	2198
3773	122	122	7654	2191	2202
3780	137	137	7655	2200	2211
3773	122	122	7654	2191	2202
3772	121	121	7653	2190	2201
3753	194	194	7654	2187	2198
3764	203	203	7667	2200	2191
3773			7636	2197	2208
3773			7636	2197	2208
3773	6	6	7636	2197	2208
3755	1685	1685	7714	2171	2151
3739	1634	1634	7715	2082	2068
3630	1905	1905	7626	1844	1824
3629	1885	1885	7624	1824	1820
3629	1885	1885	7624	1824	1820
3636	1885	1885	7621	1826	1814
3601	1973	1973	7618	1916	1912
3656	1946	1946	7683	2166	2166
3708	2008	2008	7707	2294	2294
3699	1985	1985	7725	2355	2359
3699	1985	1985	7725	2355	2359
3695	2066	2066	7726	2368	2354
3678	2020	2020	7725	2317	2320
3716	1918	1918	7693	2302	2304
3791	1921	1921	7707	2304	2307
3714	1870	1870	7769	2275	2276
3706	1913	1913	7761	2272	2273
3629	2100	2100	7577	1832	1832
3644	2120	2120	7598	1832	1845
3624	2046	2046	7588	1792	1769

3624	2048	2048	7590	1796	1773
3633	2080	2080	7594	1818	1795
3629	2094	2094	7568	1871	1846
3645	2137	2137	7597	1891	1898
3645	2137	2137	7599	1891	1898
3671	2090	2090	7612	1907	1882
3673	2092	2092	7614	1909	1884
3673	2092	2092	7614	1909	1884
3657	2113	2113	7618	1930	1902
3671	2087	2087	7611	1904	1889
3653	2081	2081	7595	1932	1921
3654	2082	2082	7596	1933	1922
3644	2083	2083	7608	1756	1732
3645	2073	2073	7606	1746	1730
3623	2064	2064	7655	1909	1891
3623	2062	2062	7657	1911	1893
3615	2055	2055	7647	1901	1883
3614	2055	2055	7646	1900	1882
3620	2083	2083	7657	1914	1896
3614	2055	2055	7646	1900	1882
3615	2056	2056	7647	1901	1883
3629	2070	2070	7659	1915	1897
3580	2102	2102	7651	1947	1914
3642	2103	2103	7633	1921	1903
3831	2372	2372	7715	577	575
3843	2386	2386	7722	597	595
3847	2390	2390	7732	595	591
3856	2353	2353	7717	572	568
3738	2229	2229	7714	353	337
3744	2236	2236	7711	344	318
3744	2236	2236	7711	344	318
3742	2234	2234	7708	342	316
3752	2244	2244	7715	350	326
3754	2220	2220	7713	334	311
3776	2210	2210	7725	308	290
3834	2290	2290	7688	444	426
3751	2261	2261	7702	355	335
3777	2206	2206	7724	252	232
3797	2229	2229	7742	320	299
3782	2231	2231	7742	277	256
3787	2236	2236	7744	282	261
3758	2223	2223	7725	315	286
3757	2233	2233	7718	295	277
3746	2230	2230	7709	292	266
3751	2187	2187	7687	67	67
3752	2198	2198	7693	80	64
3823	2396	2396	7667	332	314
3832	2405	2405	7676	341	323
3816	2389	2389	7660	325	307
3794	2258	2258	7675	289	271
3748	2208	2208	7692	96	20
3753	2208	2208	7693	96	
3780	2464	2464	7628	523	496
3780	2319	2319	7671	258	258
3770	2324	2324	7662	263	247

3789	2335	2335	7673	285	257
3784	2338	2338	7678	280	260
3782	2308	2308	7676	237	240
3774	2277	2277	7694	174	146
3764	2270	2270	7682	155	139
3762	2268	2268	7680	153	137
3736	2191	2191	7685	79	75
3728	2202	2202	7682	82	69
3773	2204	2204	7704	37	105
3766	2197	2197	7697		96
3773	2109	2109	7672	295	287
3773	2109	2109	7672	295	287
3739	2249	2249	7683	131	109
3734	2244	2244	7678	126	104
3737	2247	2247	7681	129	107
3594	2228	2228	7562	1856	1842
3595	2229	2229	7559	1857	1843
3582	2216	2216	7549	1844	1830
3763	2374	2374	7615	1998	1987
3621	2214	2214	7555	1802	1804
3832	2473	2473	7784	2127	2110
3509	3783	3783	7776	3918	3927
6624	6760	6760	7785	6739	6723
6626	6762	6762	7787	6741	6725
6623	6759	6759	7784	6738	6722
6625	6761	6761	7786	6740	6724
6740	6823	6823	7782	6810	6789
6750	6812	6812	7778	6799	6781
6763	6816	6816	7784	6803	6789
6799	6852	6852	7767	6813	6797
6813	6866	6866	7784	6827	6811
6775	6842	6842	7769	6790	6786
6855	6880	6880	7786	6853	6837
6779	6838	6838	7760	6805	6789
6808	6868	6868	7789	6834	6819
6770	6819	6819	7751	6806	6790
6785	6834	6834	7762	6803	6787
6785	6844	6844	7766	6811	6795
6958	6970	6970	7881	6983	6966
6953	6965	6965	7876	6978	6961
6913	6936	6936	7868	6937	6920
6794	6842	6842	7793	6846	6830
6901	6955	6955	7891	6951	6935
6783	6841	6841	7776	6827	6811
6777	6835	6835	7772	6821	6805
6782	6832	6832	7773	6820	6804
6764	6808	6808	7742	6789	6779
6785	6854	6854	7667	6844	6825
4214	3790	3790	7823	3677	3653
4203	3779	3779	7810	3666	3642
7480	7568	7568	3501	7693	7679
7480	7568	7568	3501	7693	7679
7479	7567	7567	3500	7692	7678
7478	7566	7566	3500	7691	7677
7480	7568	7568	3500	7693	7679

7478	7566	7566	3500	7691	7677
7480	7568	7568	3500	7693	7679
7478	7566	7566	3498	7691	7677
7481	7569	7569	3501	7694	7680
7478	7566	7566	3498	7691	7677
7477	7565	7565	3497	7690	7676
7478	7566	7566	3498	7691	7677
7479	7567	7567	3499	7692	7678
7479	7567	7567	3499	7692	7678
7562	7705	7705	3366	7783	7769
7566	7709	7709	3370	7787	7773
7566	7709	7709	3370	7787	7773
7569	7712	7712	3373	7790	7776
7511	7666	7666	3428	7744	7730
7563	7706	7706	3367	7784	7770
7556	7699	7699	3377	7773	7759
7554	7697	7697	3375	7771	7757
7559	7702	7702	3379	7776	7762
7554	7697	7697	3374	7771	7757
7555	7699	7699	3376	7773	7759
7561	7703	7703	3376	7778	7764
7477	7556	7556	3461	7609	7595
7448	7614	7614	3592	7688	7681
7446	7618	7618	3648	7688	7681
7647	7748	7748	3377	7842	7832
7647	7748	7748	3377	7842	7832
7653	7753	7753	3385	7847	7837
7702	7783	7783	3403	7879	7869
7644	7771	7771	3357	7853	7841
7525	7611	7611	2642	7690	7678
7528	7614	7614	2645	7693	7681
7535	7623	7623	2649	7698	7686
7522	7618	7618	2655	7703	7691
7512	7597	7597	2555	7669	7657
7520	7605	7605	2563	7677	7665
7511	7596	7596	2554	7668	7656
7573	7660	7660	2579	7742	7732
7575	7662	7662	2581	7744	7734
7579	7666	7666	2586	7748	7738
7592	7679	7679	2598	7761	7751
7544	7642	7642	2624	7660	7649
7545	7643	7643	2625	7661	7650
7545	7643	7643	2625	7661	7650
7546	7644	7644	2626	7662	7651
7544	7642	7642	2624	7660	7649
7546	7644	7644	2624	7662	7651
7547	7637	7637	7	7698	7694
7546	7636	7636		7697	7693
7545	7635	7635	5	7696	7692
7544	7634	7634	4	7695	7691
7546	7636	7636	6	7697	7693
7545	7635	7635	5	7696	7692
7528	7612	7612	42	7673	7669
7548	7638	7638	12	7699	7695
7588	7707	7707	2660	7782	7770

7667	7681	7681	2598	7755	7744
7670	7684	7684	2601	7758	7747
7668	7682	7682	2599	7756	7745
7665	7679	7679	2599	7753	7742
24880	24998	24998	25523	25040	25038
24879	24997	24997	25522	25039	25037
30915	30969	30969	31233	30996	31009

MOD1-EC3584	CDPHFDLB-F1602032-004B	MOD1-EC1638	29618	149438	E123	287552	140183
3401	833	902	7606	3584	3605	3573	3538
3359	144	428	7544	3493	3526	3530	3519
3359	146	430	7546	3493	3524	3530	3517
3395	322	452	7571	3532	3563	3547	3555
3372	202	445	7597	3504	3558	3542	3535
3398	143	451	7552	3529	3578	3569	3566
3398	143	451	7552	3529	3578	3569	3566
3412	60	440	7579	3546	3583	3588	3572
3416		442	7583	3549	3582	3591	3574
3438	302	470	7537	3582	3590	3609	3600
3513	395	606	7530	3599	3622	3633	3625
3435	165	474	7583	3565	3588	3608	3592
3537	590	191	7618	3688	3702	3698	3678
3537	590	191	7618	3688	3702	3698	3678
3461	442	40	7580	3598	3639	3629	3613
3457	442		7585	3595	3628	3627	3610
3327	348	251	7543	3472	3509	3501	3501
3376	415	283	7565	3523	3550	3548	3542
3430	435	341	7571	3566	3609	3612	3607
3431	555	514	7539	3583	3636	3626	3652
3478	483	402	7595	3606	3649	3653	3660
3426	736	783	7546	3528	3549	3508	3528
3496	560	591	7556	3596	3624	3607	3605
3522	503	413	7592	3608	3644	3638	3632
3403	490	611	7538	3485	3543	3513	3521
3449	551	583	7599	3549	3590	3585	3570
3382	1152	1168	7589	3467	3572	3580	3519
3506	1292	1253	7608	3594	3699	3681	3657
3497	973	990	7546	3580	3630	3653	3629
3433	789	877	7558	3533	3582	3562	3554
3433	789	877	7558	3533	3582	3562	3554
3436	792	880	7560	3536	3585	3565	3556
3397	873	947	7581	3535	3607	3550	3578
3388	1149	1162	7583	3475	3564	3572	3521
3388	1149	1162	7583	3475	3564	3572	3521
1789	3539	3574	7613	1849	1977	1963	1960
1780	3533	3580	7613	1840	1961	1956	1951
1762	3541	3582	7614	1822	1955	1946	1931
1801	3506	3555	7624	1819	1982	1969	1970
1766	3504	3561	7599	1824	1941	1933	1933
1774	3530	3565	7616	1838	1951	1944	1945
1773	3529	3564	7615	1837	1950	1943	1944
1750	3528	3572	7614	1822	1933	1933	1942
1788	3542	3571	7611	1850	1981	1970	1961
1818	3562	3576	7569	1892	1993	1984	1969
1864	3544	3580	7568	1966	2067	2072	2043
1844	3597	3655	7608	1937	1968	1957	1978
19	3421	3462	7581	1735	1883	1815	1756
14	3416	3457	7578	1730	1878	1810	1751
20	3422	3463	7584	1736	1884	1816	1757
	3416	3457	7578	1730	1878	1810	1751
21	3421	3462	7583	1735	1883	1815	1756
1442	3582	3613	7607	1598	1829	1819	1937

1686	3630	3687	7645	1745	2100	1893	1970
1738	3555	3601	7620	22	1788	1809	1901
1737	3554	3600	7619	21	1787	1808	1900
1730	3549	3595	7614		1780	1803	1893
1732	3551	3597	7616	16	1782	1805	1895
1691	3611	3656	7606	357	1790	1757	1893
1700	3620	3664	7615	366	1799	1766	1902
1700	3620	3665	7615	366	1799	1766	1902
1703	3582	3628	7586	254	1719	1754	1864
1724	3605	3651	7589	289	1738	1775	1887
1740	3566	3612	7569	307	1758	1778	1871
1695	3574	3620	7578	246	1711	1746	1856
1734	3583	3629	7612	544	1761	1821	1884
1734	3583	3629	7612	544	1761	1821	1884
1723	3572	3618	7602	533	1750	1810	1873
1676	3583	3631	7607	446	1732	1807	1877
1611	3578	3613	7580	1467	1927	1878	1872
1642	3609	3644	7608	1498	1958	1909	1903
1613	3580	3615	7582	1469	1929	1880	1874
1611	3578	3613	7580	1467	1927	1878	1872
2126	3743	3780	7637	1927	1912	2078	2128
2128	3746	3783	7641	1929	1914	2080	2130
2122	3740	3777	7635	1923	1908	2074	2124
2119	3737	3774	7632	1920	1905	2071	2121
2109	3744	3785	7641	1924	1913	2081	2115
2117	3737	3772	7632	1918	1903	2069	2119
2121	3739	3776	7634	1922	1907	2073	2123
2136	3746	3783	7635	1911	1896	2088	2136
2121	3739	3776	7634	1922	1907	2073	2123
2120	3738	3775	7633	1921	1906	2072	2122
2127	3715	3754	7624	1936	1901	2083	2095
2168	3730	3767	7645	1983	1900	2144	2160
2027	3677	3720	7644	1928	1905	2081	2070
2027	3677	3720	7644	1928	1905	2081	2070
2027	3677	3720	7644	1928	1905	2081	2070
2100	3746	3782	7698	2072	1971	2145	2080
2083	3750	3800	7690	2005	1907	2057	2043
1878	3582	3628	7591	1780		2050	1984
1860	3579	3626	7590	1764	32	2030	1964
1860	3579	3626	7590	1764	34	2030	1964
1846	3582	3635	7587	1742	98	2034	1970
1815	3561	3620	7618	1838	1369	1978	1874
2050	3649	3678	7653	2020	2072	1965	2205
2108	3697	3726	7699	2148	2193	2085	2217
2097	3692	3721	7717	2195	2190	2094	2242
2097	3692	3721	7717	2195	2190	2094	2242
2138	3681	3714	7714	2179	2225	2111	2307
2100	3669	3703	7719	2151	2148	2091	2276
2102	3711	3746	7692	2106	2136	2108	2170
2077	3783	3812	7706	2058	2165	2161	2208
2245	3712	3763	7767	2136	2048	2261	2250
2278	3712	3763	7760	2149	2048	2296	2259
1790	3559	3597	7570	1816	1995	613	1867
1799	3561	3609	7588	1827	2016	628	1878
1730	3592	3628	7576	1732	1949	530	1814

1734	3592	3628	7578	1734	1951	532	1816
1758	3592	3628	7586	1756	1979	494	1824
1883	3584	3607	7566	1776	2065	506	1880
1813	3586	3637	7584	1842	1967	397	1891
1813	3586	3637	7586	1842	1967	397	1891
1809	3609	3646	7605	1784	2057	115	1924
1811	3611	3648	7607	1786	2059	117	1926
1811	3611	3648	7605	1786	2059	117	1926
1833	3585	3658	7601	1809	2068	138	1952
1816	3611	3647	7605	1791	2054	110	1929
1810	3591	3627	7587	1803	2050		1937
1811	3592	3628	7588	1804	2051	17	1938
1770	3620	3644	7591	1692	1831	708	1891
1766	3621	3654	7592	1686	1827	698	1885
1745	3568	3604	7645	1887	1978	1931	48
1747	3568	3604	7647	1887	1980	1931	50
1736	3560	3596	7637	1876	1970	1923	40
1736	3559	3595	7636	1878	1969	1922	39
1756	3549	3597	7647	1898	1993	1950	73
1736	3559	3595	7636	1878	1969	1924	41
1737	3560	3596	7637	1879	1970	1923	40
1751	3574	3610	7651	1893	1984	1937	
1782	3527	3585	7634	1919	1992	1967	91
1802	3591	3623	7635	1937	2048	1970	189
2061	3810	3855	7681	1986	1977	2140	2097
2066	3812	3857	7688	2002	1992	2146	2106
2077	3826	3869	7696	2002	1995	2158	2113
2032	3835	3878	7672	1977	1970	2103	2082
1875	3737	3778	7681	1815	1844	1994	1957
1868	3734	3767	7677	1806	1835	1993	1950
1868	3734	3767	7677	1806	1835	1993	1950
1866	3732	3765	7674	1804	1833	1991	1948
1874	3738	3771	7681	1818	1849	2001	1948
1843	3741	3806	7662	1802	1831	1959	1933
1846	3764	3807	7685	1790	1823	1967	1922
1928	3818	3862	7646	1852	1907	2058	1993
1899	3757	3791	7664	1881	1882	1998	1950
1806	3764	3803	7687	1776	1809	1943	1920
1813	3763	3823	7692	1791	1838	1952	1929
1831	3766	3824	7694	1799	1846	1968	1943
1836	3771	3829	7696	1804	1851	1973	1948
1851	3764	3814	7673	1815	1866	1954	1938
1829	3739	3774	7685	1797	1850	1972	1947
1824	3732	3759	7676	1792	1835	1965	1940
1825	3749	3794	7649	1725	1825	1902	1886
1826	3750	3794	7652	1726	1831	1914	1889
2022	3871	3847	7609	1936	2000	2081	2057
2031	3880	3856	7618	1945	2009	2090	2066
2015	3864	3840	7602	1929	1993	2074	2050
1908	3783	3821	7645	1800	1882	1931	1965
1831	3754	3794	7648	1733	1823	1920	1897
1832	3757	3799	7651	1734	1824	1921	1897
2119	3766	3854	7555	2012	2100	2140	2120
1952	3725	3787	7640	1878	1976	2021	1988
1949	3721	3778	7628	1877	1973	2022	1987

1959	3745	3797	7639	1887	1983	2032	1997
1962	3740	3792	7644	1890	1986	2035	2000
1958	3726	3817	7632	1875	1959	2043	2023
1898	3761	3817	7656	1802	1902	1995	1967
1891	3740	3807	7644	1795	1895	1985	1960
1889	3738	3805	7642	1793	1893	1983	1958
1841	3744	3770	7645	1743	1825	1918	1906
1828	3726	3793	7634	1729	1829	1916	1893
1859	3745	3818	7669	1761	1853	1939	1922
1852	3738	3811	7662	1752	1844	1932	1915
1825	3756	3795	7635	1667	1791	1815	1810
1825	3756	3795	7635	1667	1791	1815	1810
1871	3730	3783	7650	1774	1875	1959	1936
1866	3725	3778	7645	1770	1870	1954	1931
1869	3728	3781	7648	1773	1873	1957	1934
1903	3502	3570	7526	1924	1901	1871	1885
1904	3504	3572	7523	1925	1902	1870	1886
1891	3492	3560	7513	1912	1889	1859	1873
2144	3670	3718	7575	2058	2094	2074	2131
2003	3529	3596	7529	1930	1937	1905	1936
2234	3740	3789	7770	2201	2194	2048	2109
3660	3407	3417	7755	3779	3859	3759	3801
6586	6643	6647	7767	6645	6608	6737	6672
6588	6645	6649	7769	6647	6610	6739	6674
6585	6642	6646	7766	6644	6607	6736	6671
6587	6644	6648	7768	6646	6609	6738	6673
6662	6759	6784	7741	6714	6661	6806	6728
6652	6769	6772	7747	6705	6650	6797	6714
6656	6778	6784	7756	6707	6654	6801	6722
6686	6818	6822	7735	6745	6694	6825	6758
6700	6832	6836	7750	6759	6708	6839	6772
6679	6785	6798	7739	6740	6686	6822	6745
6724	6874	6878	7752	6777	6724	6851	6796
6676	6798	6802	7726	6729	6678	6817	6742
6706	6827	6831	7755	6759	6708	6847	6772
6659	6789	6793	7717	6712	6661	6804	6725
6680	6804	6808	7728	6731	6676	6821	6744
6682	6804	6808	7732	6735	6684	6823	6748
6827	7011	6997	7861	6874	6839	6954	6920
6822	7006	6992	7856	6869	6834	6949	6915
6789	6970	6950	7834	6840	6801	6913	6878
6698	6813	6823	7765	6750	6701	6840	6768
6802	6942	6845	7871	6858	6821	6944	6890
6688	6778	6816	7751	6743	6708	6819	6761
6682	6772	6810	7745	6737	6702	6813	6755
6681	6777	6815	7746	6736	6701	6812	6754
6668	6758	6796	7704	6706	6679	6784	6731
6708	6780	6843	7653	6754	6725	6832	6785
3503	4204	4222	7772	3527	3649	3261	3563
3492	4193	4211	7761	3516	3638	3250	3552
7569	7501	7538	3428	7555	7579	7541	7617
7569	7501	7538	3428	7555	7579	7541	7617
7568	7500	7537	3427	7554	7578	7540	7616
7567	7499	7536	3427	7553	7577	7539	7615
7569	7501	7538	3427	7555	7579	7541	7617

7567	7499	7536	3427	7553	7577	7539	7615
7569	7501	7538	3427	7555	7579	7541	7617
7567	7499	7536	3425	7553	7577	7539	7615
7570	7502	7539	3428	7556	7580	7542	7618
7567	7499	7536	3425	7553	7577	7539	7615
7566	7498	7535	3424	7552	7576	7538	7614
7567	7499	7536	3425	7553	7577	7539	7615
7568	7500	7537	3426	7554	7578	7540	7616
7568	7500	7537	3426	7554	7578	7540	7616
7643	7594	7615	3291	7695	7691	7682	7715
7647	7598	7619	3295	7699	7695	7686	7719
7647	7598	7619	3295	7699	7695	7686	7719
7650	7601	7622	3298	7702	7698	7689	7722
7600	7527	7556	3361	7652	7652	7637	7680
7644	7595	7616	3292	7696	7692	7683	7716
7635	7588	7609	3304	7687	7683	7674	7707
7633	7586	7607	3302	7685	7681	7672	7705
7638	7591	7612	3304	7690	7686	7677	7710
7633	7586	7607	3299	7685	7681	7672	7705
7635	7587	7608	3303	7687	7683	7674	7707
7639	7593	7614	3303	7691	7687	7678	7713
7502	7476	7562	3383	7541	7546	7557	7606
7548	7490	7461	3444	7593	7599	7584	7621
7544	7488	7459	3504	7591	7601	7594	7617
7700	7665	7690	3466	7715	7748	7730	7750
7700	7665	7690	3466	7715	7748	7730	7750
7705	7672	7699	3473	7720	7753	7735	7755
7739	7722	7749	3475	7754	7780	7773	7790
7699	7664	7693	3445	7734	7775	7751	7780
7561	7563	7563	2341	7606	7621	7597	7651
7564	7566	7566	2344	7609	7624	7600	7654
7571	7573	7573	2346	7614	7629	7607	7659
7563	7552	7560	2384	7610	7621	7599	7656
7545	7536	7544	2306	7580	7601	7573	7625
7553	7544	7552	2314	7588	7609	7581	7633
7544	7535	7543	2305	7579	7600	7572	7624
7605	7607	7602	2306	7651	7677	7646	7699
7607	7609	7604	2308	7653	7679	7648	7701
7611	7613	7608	2313	7657	7683	7652	7705
7624	7626	7621	2325	7670	7696	7665	7718
7576	7581	7583	6	7612	7589	7585	7649
7577	7580	7582	7	7613	7590	7586	7650
7577	7582	7584	7	7613	7590	7586	7650
7578	7583	7585		7614	7591	7587	7651
7576	7581	7583	6	7612	7589	7585	7649
7578	7583	7585	41	7614	7590	7587	7651
7583	7555	7566	2627	7620	7627	7596	7660
7582	7554	7565	2626	7619	7626	7595	7659
7581	7553	7564	2625	7618	7625	7594	7658
7580	7552	7563	2624	7617	7624	7593	7657
7582	7554	7565	2626	7619	7626	7595	7659
7581	7553	7564	2625	7618	7625	7594	7658
7560	7536	7547	2654	7597	7602	7571	7635
7584	7556	7567	2628	7621	7628	7597	7661
7671	7613	7627	2559	7696	7690	7682	7728

7635	7681	7680	2516	7694	7705	7641	7694
7638	7684	7683	2519	7697	7708	7644	7697
7636	7682	7681	2517	7695	7706	7642	7695
7633	7679	7678	2517	7692	7704	7639	7692
25010	24941	24927	25499	25015	24974	24966	25042
25009	24940	24926	25498	25014	24973	24965	25041
30966	30931	30935	31227	30935	30950	31004	30986

93272	182138	272422	E2026_9	MOD1-EC3605	MOD1-EC3330	CFSAN044415	MOD1-EC5432
3586	3584	3615	6802	871	3524	3713	7546
3518	3516	3547	6774	962	3505	3654	7487
3518	3516	3547	6778	964	3503	3654	7489
3562	3560	3591	6782	908	3541	3689	7523
3537	3535	3566	6783	1004	3521	3684	7545
3560	3558	3589	6785	1009	3552	3695	7510
3560	3558	3589	6785	1009	3552	3695	7510
3576	3574	3605	6811	971	3558	3709	7532
3580	3578	3609	6813	973	3560	3711	7536
3615	3613	3644	6822	999	3586	3741	7486
3637	3635	3666	6826	858	3611	3738	7496
3597	3595	3626	6803	984	3578	3719	7535
3708	3706	3737	6835	1029	3664	3804	7588
3708	3706	3737	6835	1029	3664	3804	7588
3618	3616	3647	6811	1000	3599	3749	7539
3615	3613	3644	6823	990	3596	3746	7544
3496	3494	3525	6770	904	3487	3621	7508
3543	3541	3572	6701	984	3528	3672	7526
3589	3587	3618	6804	969	3593	3716	7527
3614	3612	3643	6816	1136	3638	3756	7511
3627	3625	3656	6847	985	3646	3778	7542
3535	3533	3564	6771	1007	3514	3678	7520
3627	3625	3656	6772	890	3591	3721	7519
3644	3642	3673	6828	857	3618	3737	7548
3501	3499	3530	6766	878	3507	3633	7497
3572	3570	3601	6785	901	3556	3650	7563
3510	3508	3539	6803	699	3505	3638	7570
3663	3661	3692	6805	858	3643	3739	7565
3606	3604	3635	6794		3615	3716	7512
3541	3539	3570	6768	474	3540	3645	7515
3541	3539	3570	6768	474	3540	3645	7515
3544	3542	3573	6769	477	3542	3649	7518
3558	3556	3587	6794	548	3564	3640	7556
3512	3510	3541	6797	692	3507	3640	7562
3512	3510	3541	6797	692	3507	3640	7562
1864	1862	1893	6704	3584	1946	2377	7606
1857	1855	1886	6694	3583	1937	2352	7605
1841	1839	1870	6699	3584	1917	2348	7605
1886	1884	1915	6682	3518	1956	2326	7610
1842	1840	1871	6690	3564	1919	2352	7589
1855	1853	1884	6686	3581	1931	2364	7607
1854	1852	1883	6685	3580	1930	2363	7606
1843	1841	1872	6698	3558	1928	2356	7603
1869	1867	1898	6701	3561	1947	2371	7599
1908	1906	1937	6648	3562	1955	2378	7558
1967	1965	1996	6692	3552	2029	2435	7556
1819	1817	1848	6799	3643	1965	2326	7598
1618	1616	1647	6703	3502	1741	2107	7548
1613	1611	1642	6698	3497	1736	2102	7545
1619	1617	1648	6704	3503	1742	2108	7551
1613	1611	1642	6698	3497	1736	2102	7545
1618	1616	1647	6703	3502	1741	2107	7550
1657	1655	1686	6730	3635	1923	2044	7602

1840	1838	1869	6840	3708	1955	2174	7652
1475	1473	1504	6761	3586	1884	2114	7588
1474	1472	1503	6760	3585	1883	2113	7587
1469	1467	1498	6750	3580	1876	2106	7580
1471	1469	1500	6755	3582	1878	2108	7582
1423	1421	1452	6768	3649	1876	2103	7589
1432	1430	1461	6777	3658	1885	2112	7598
1432	1430	1461	6777	3658	1885	2112	7598
1426	1424	1455	6757	3613	1847	2072	7571
1457	1455	1486	6772	3636	1870	2089	7580
1467	1465	1496	6738	3603	1854	2101	7553
1418	1416	1447	6749	3605	1839	2064	7563
1516	1514	1545	6759	3636	1867	2080	7592
1516	1514	1545	6759	3636	1867	2080	7592
1505	1503	1534	6748	3625	1856	2069	7584
1514	1512	1543	6742	3632	1860	2108	7587
6		35	6814	3604	1858	2057	7575
37	35		6842	3635	1889	2088	7604
	6	37	6816	3606	1860	2059	7577
6	4	35	6814	3604	1858	2057	7575
2116	2114	2145	6843	3777	2113	1914	7609
2118	2116	2147	6845	3780	2115	1916	7611
2112	2110	2141	6839	3774	2109	1910	7605
2109	2107	2138	6836	3771	2106	1907	7602
2091	2089	2120	6847	3781	2100	1939	7611
2107	2105	2136	6840	3769	2104	1915	7604
2111	2109	2140	6838	3773	2108	1909	7604
2126	2124	2155	6849	3780	2121	1898	7605
2111	2109	2140	6838	3773	2108	1909	7604
2110	2108	2139	6837	3772	2107	1908	7603
2115	2113	2144	6826	3753	2080	1863	7600
2076	2074	2105	6823	3764	2146	1901	7617
2141	2139	2170	6842	3773	2055	1918	7597
2141	2139	2170	6842	3773	2055	1918	7597
2141	2139	2170	6842	3773	2055	1918	7597
1934	1932	1963	6799	3755	2066	1368	7663
1866	1864	1895	6797	3739	2029	1467	7664
1929	1927	1958	6701	3630	1970	2136	7601
1911	1909	1940	6705	3629	1950	2120	7600
1911	1909	1940	6705	3629	1950	2120	7600
1891	1889	1920	6701	3636	1956	2104	7596
1864	1862	1893	6808	3601	1860	1902	7601
1894	1892	1923	6822	3656	2191	534	7633
2015	2013	2044	6807	3708	2203	301	7672
2056	2054	2085	6813	3699	2228	322	7682
2056	2054	2085	6813	3699	2228	322	7682
2077	2075	2106	6816	3695	2293	353	7687
2010	2008	2039	6821	3678	2262	321	7689
2059	2057	2088	6812	3716	2154		7647
2150	2148	2179	6785	3791	2194	762	7655
2147	2145	2176	6873	3714	2236	1609	7731
2150	2148	2179	6884	3706	2245	1652	7723
1848	1846	1877	6771	3629	1853	2065	7573
1857	1855	1886	6791	3644	1864	2079	7594
1778	1776	1807	6781	3624	1800	2007	7581

1780	1778	1809	6784	3624	1802	2009	7583
1804	1802	1833	6782	3633	1810	2029	7591
1747	1745	1776	6804	3629	1866	2083	7555
1829	1827	1858	6807	3645	1877	2094	7583
1829	1827	1858	6809	3645	1877	2094	7585
1831	1829	1860	6831	3671	1910	2117	7594
1833	1831	1862	6833	3673	1912	2119	7596
1833	1831	1862	6833	3673	1912	2119	7594
1859	1857	1888	6838	3657	1938	2143	7590
1838	1836	1867	6826	3671	1915	2124	7594
1880	1878	1909	6840	3653	1923	2108	7573
1881	1879	1910	6841	3654	1924	2109	7574
1625	1623	1654	6800	3644	1877	2059	7605
1621	1619	1650	6798	3645	1871	2055	7606
1868	1866	1897	6762	3623	34	2164	7621
1870	1868	1899	6762	3623	36	2166	7623
1860	1858	1889	6754	3615		2154	7613
1859	1857	1888	6753	3614	11	2155	7612
1879	1877	1908	6738	3620	59	2175	7625
1859	1857	1888	6753	3614	27	2155	7612
1860	1858	1889	6754	3615	26	2156	7613
1874	1872	1903	6768	3629	40	2170	7625
1905	1903	1934	6767	3580	77	2197	7610
1922	1920	1951	6768	3642	175	2218	7605
2007	2005	2036	6794	3831	2083	2425	7703
2022	2020	2051	6797	3843	2092	2441	7708
2023	2021	2052	6811	3847	2099	2441	7718
1968	1966	1997	6795	3856	2068	2414	7694
1845	1843	1874	6852	3738	1943	2290	7686
1840	1838	1869	6852	3744	1936	2281	7684
1840	1838	1869	6852	3744	1936	2281	7684
1838	1836	1867	6849	3742	1934	2279	7681
1844	1842	1873	6860	3752	1934	2291	7688
1803	1801	1832	6858	3754	1919	2276	7667
1816	1814	1845	6858	3776	1908	2265	7692
1917	1915	1946	6845	3834	1979	2348	7648
1923	1921	1952	6839	3751	1936	2328	7662
1830	1828	1859	6845	3777	1906	2279	7690
1852	1850	1881	6876	3797	1915	2300	7693
1849	1847	1878	6872	3782	1929	2300	7695
1854	1852	1883	6878	3787	1934	2305	7697
1881	1879	1910	6852	3758	1924	2308	7678
1849	1847	1878	6859	3757	1933	2298	7688
1844	1842	1873	6850	3746	1926	2293	7679
1737	1735	1766	6833	3751	1872	2291	7655
1738	1736	1767	6838	3752	1875	2292	7658
1950	1948	1979	6837	3823	2043	2456	7611
1959	1957	1988	6846	3832	2052	2465	7620
1943	1941	1972	6830	3816	2036	2449	7604
1810	1808	1839	6800	3794	1951	2354	7647
1747	1745	1776	6829	3748	1883	2304	7656
1748	1746	1777	6830	3753	1883	2304	7657
2049	2047	2078	6759	3780	2106	2556	7542
1880	1878	1909	6823	3780	1974	2414	7642
1877	1875	1906	6816	3770	1973	2411	7630

1885	1883	1914	6821	3789	1983	2422	7639
1890	1888	1919	6832	3784	1986	2425	7646
1871	1869	1900	6839	3782	2009	2425	7633
1810	1808	1839	6856	3774	1953	2381	7662
1800	1798	1829	6849	3764	1946	2374	7650
1798	1796	1827	6847	3762	1944	2372	7648
1749	1747	1778	6831	3736	1892	2291	7651
1743	1741	1772	6832	3728	1879	2298	7638
1772	1770	1801	6853	3773	1908	2307	7676
1765	1763	1794	6846	3766	1901	2302	7669
1721	1719	1750	6809	3773	1796	2206	7641
1721	1719	1750	6809	3773	1796	2206	7641
1782	1780	1811	6836	3739	1922	2353	7654
1777	1775	1806	6831	3734	1917	2348	7649
1780	1778	1809	6834	3737	1920	2351	7652
1807	1805	1836	6694	3594	1871	2297	7523
1808	1806	1837	6693	3595	1872	2298	7520
1795	1793	1824	6682	3582	1859	2285	7511
1966	1964	1995	6795	3763	2117	2397	7590
1809	1807	1838	6718	3621	1922	2254	7513
2088	2086	2117	6954	3832	2095	2428	7755
3814	3812	3843	6985	3509	3787	3818	7706
6714	6712	6740	422	6624	6658	6703	7770
6716	6714	6742	424	6626	6660	6705	7772
6713	6711	6739	421	6623	6657	6702	7769
6715	6713	6741	423	6625	6659	6704	7771
6784	6782	6810	290	6740	6714	6779	7731
6772	6770	6798	279	6750	6700	6767	7737
6780	6778	6806	283	6763	6708	6773	7746
6810	6808	6836	245	6799	6744	6805	7726
6824	6822	6850	261	6813	6758	6821	7743
6801	6799	6827	301	6775	6731	6797	7730
6848	6846	6874	299	6855	6782	6847	7741
6802	6800	6828	225	6779	6728	6797	7719
6832	6830	6858	255	6808	6758	6827	7748
6785	6783	6811	209	6770	6711	6780	7710
6800	6798	6826	223	6785	6730	6795	7721
6808	6806	6834	231	6785	6734	6803	7725
6946	6944	6972	424	6958	6906	6950	7835
6941	6939	6967	419	6953	6901	6945	7830
6916	6914	6942	432	6913	6864	6916	7814
6816	6814	6842		6794	6754	6812	7749
6926	6924	6952	326	6901	6876	6922	7856
6811	6809	6837	201	6783	6747	6811	7724
6805	6803	6831	195	6777	6741	6805	7718
6804	6802	6830	198	6782	6740	6804	7719
6778	6776	6804	280	6764	6717	6780	7684
6819	6817	6845	449	6785	6773	6843	7615
3564	3562	3593	6940	4214	3549	3688	7758
3553	3551	3582	6930	4203	3538	3677	7745
7544	7542	7571	7905	7480	7605	7675	3365
7544	7542	7571	7905	7480	7605	7675	3365
7543	7541	7570	7904	7479	7604	7674	3364
7542	7540	7569	7903	7478	7603	7673	3364
7544	7542	7571	7905	7480	7605	7675	3364

7542	7540	7569	7905	7478	7603	7673	3362
7544	7542	7571	7905	7480	7605	7675	3364
7542	7540	7569	7903	7478	7603	7673	3362
7545	7543	7572	7906	7481	7606	7676	3365
7542	7540	7569	7903	7478	7603	7673	3362
7541	7539	7568	7902	7477	7602	7672	3361
7542	7540	7569	7903	7478	7603	7673	3362
7543	7541	7570	7904	7479	7604	7674	3363
7543	7541	7570	7904	7479	7604	7674	3363
7677	7675	7704	7962	7562	7703	7771	3232
7681	7679	7708	7966	7566	7707	7775	3236
7681	7679	7708	7966	7566	7707	7775	3236
7684	7682	7711	7969	7569	7710	7778	3239
7628	7626	7655	7965	7511	7668	7726	3301
7678	7676	7705	7963	7563	7704	7772	3233
7668	7666	7695	7959	7556	7695	7763	3233
7666	7664	7693	7958	7554	7693	7761	3231
7671	7669	7698	7962	7559	7698	7766	3235
7666	7664	7693	7957	7554	7693	7761	3230
7668	7666	7695	7961	7555	7695	7763	3232
7674	7672	7701	7962	7561	7701	7769	3232
7530	7528	7557	7898	7477	7594	7648	3320
7602	7600	7629	7862	7448	7609	7675	3408
7602	7600	7629	7854	7446	7605	7679	3449
7735	7733	7760	7978	7647	7738	7793	3344
7735	7733	7760	7978	7647	7738	7793	3344
7740	7738	7765	7985	7653	7743	7798	3352
7768	7766	7793	8025	7702	7778	7831	3370
7726	7724	7751	7961	7644	7768	7815	3326
7598	7596	7625	7718	7525	7639	7670	284
7601	7599	7628	7721	7528	7642	7673	287
7608	7606	7635	7729	7535	7647	7680	275
7602	7600	7629	7706	7522	7644	7687	502
7577	7575	7604	7749	7512	7613	7647	
7585	7583	7612	7757	7520	7621	7655	20
7576	7574	7603	7748	7511	7612	7646	11
7647	7645	7674	7755	7573	7687	7714	642
7649	7647	7676	7757	7575	7689	7716	644
7653	7651	7680	7761	7579	7693	7720	649
7666	7664	7693	7774	7592	7706	7733	661
7580	7578	7606	7763	7544	7635	7690	2304
7581	7579	7607	7764	7545	7636	7691	2305
7581	7579	7607	7764	7545	7636	7691	2305
7582	7580	7608	7765	7546	7637	7692	2306
7580	7578	7606	7763	7544	7635	7690	2304
7582	7580	7608	7765	7546	7637	7692	2302
7590	7588	7617	7794	7547	7648	7694	2556
7589	7587	7616	7793	7546	7647	7693	2555
7588	7586	7615	7792	7545	7646	7692	2554
7587	7585	7614	7791	7544	7645	7691	2553
7589	7587	7616	7793	7546	7647	7693	2555
7588	7586	7615	7792	7545	7646	7692	2554
7565	7563	7592	7819	7528	7623	7675	2586
7591	7589	7618	7795	7548	7649	7695	2557
7669	7667	7696	7824	7588	7714	7783	2536

7664	7662	7691	7984	7667	7682	7754	2356
7667	7665	7694	7989	7670	7685	7757	2359
7665	7663	7692	7987	7668	7683	7755	2357
7662	7660	7689	7982	7665	7680	7752	2356
24980	24978	25006	25028	24880	25035	24967	25516
24979	24977	25005	25027	24879	25034	24966	25515
30948	30946	30973	30779	30915	30974	30947	31199

UMB12_01.1uot	RR1	UMB08_01.1uog	MOD1-EC3102	NC_P10-04	NC_P19-11	NC_STEC173
7634	1017	3618	7694	3762	3768	3636
7579	550	3527	7645	3680	3686	3549
7581	552	3527	7645	3682	3688	3549
7615	631	3566	7668	3708	3714	3588
7635	567	3539	7682	3709	3715	3561
7598	565	3563	7639	3721	3727	3585
7598	565	3563	7639	3721	3727	3585
7622	549	3580	7682	3737	3743	3602
7626	551	3583	7684	3740	3746	3605
7577	649	3618	7629	3754	3760	3640
7579	533	3633	7652	3758	3764	3655
7625	577	3603	7674	3738	3744	3621
7660	622	3724	7725	3842	3848	3744
7660	622	3724	7725	3842	3848	3744
7617	583	3634	7679	3780	3786	3654
7621	583	3631	7683	3777	3783	3651
7586	518	3508	7646	3672	3678	3528
7602	554	3559	7670	3707	3713	3579
7605	589	3600	7683	3742	3748	3622
7587	776	3635	7653	3785	3791	3635
7622	632	3660	7702	3806	3812	3660
7602	694	3562	7650	3686	3692	3585
7599	493	3628	7662	3737	3743	3650
7630	526	3640	7685	3752	3758	3662
7577	462	3521	7636	3660	3666	3539
7645		3583	7702	3705	3711	3603
7657	1006	3519	7709	3687	3693	3525
7653	1064	3644	7720	3785	3791	3658
7592	901	3632	7670	3774	3780	3636
7597	700	3571	7670	3692	3698	3587
7597	700	3571	7670	3692	3698	3587
7600	703	3574	7672	3695	3701	3590
7638	800	3571	7696	3710	3716	3589
7649	1014	3525	7705	3682	3688	3531
7649	1014	3525	7705	3682	3688	3531
7695	3555	1830	7692	2217	2223	1843
7696	3544	1821	7697	2186	2192	1834
7694	3543	1803	7696	2192	2198	1816
7703	3490	1800	7699	2183	2189	1813
7680	3517	1805	7683	2186	2192	1818
7696	3545	1815	7696	2198	2204	1832
7695	3544	1812	7695	2197	2203	1831
7692	3545	1799	7692	2196	2202	1818
7688	3540	1835	7687	2213	2219	1844
7649	3556	1869	7667	2246	2252	1886
7643	3519	1923	7643	2299	2305	1942
7692	3578	1855	7696	2246	2252	1924
7627	3454	1681	7641	2127	2133	1729
7624	3449	1676	7638	2122	2128	1724
7630	3455	1682	7644	2128	2134	1730
7624	3449	1676	7638	2122	2128	1724
7629	3454	1681	7643	2127	2133	1729
7684	3573	1535	7695	2076	2082	1540

7728	3666	1770	7733	2263	2269	1735
7678	3555	454	7703	1931	1937	297
7677	3554	453	7702	1930	1936	296
7670	3549	446	7697	1923	1929	289
7672	3551	448	7699	1925	1931	291
7668	3615	328	7692	1916	1922	216
7677	3624	337	7701	1925	1931	225
7677	3624	337	7701	1925	1931	225
7652	3580	226	7667	1869	1875	59
7661	3603	263	7678	1886	1892	
7634	3570	259	7651	1899	1905	112
7644	3572	218	7659	1861	1867	51
7673	3583	318	7691	1911	1917	377
7673	3583	318	7691	1911	1917	377
7665	3572	307	7681	1900	1906	366
7668	3583		7689	1926	1932	263
7664	3570	1512	7665	2110	2116	1455
7693	3601	1543	7694	2141	2147	1486
7666	3572	1514	7667	2112	2118	1457
7664	3570	1512	7665	2110	2116	1455
7704	3708	1930	7704	12	18	1890
7706	3711	1932	7706	14		1892
7700	3705	1926	7700		14	1886
7697	3702	1923	7697	5	11	1883
7706	3711	1927	7708	49	55	1887
7699	3702	1921	7699	13	19	1881
7699	3704	1925	7699	7	13	1885
7700	3711	1914	7700	22	28	1874
7699	3704	1925	7699	7	13	1885
7698	3703	1924	7698	6	12	1884
7695	3688	1939	7699	79	85	1899
7711	3705	1984	7710	94	100	1944
7679	3704	1927	7684	123	129	1887
7679	3704	1927	7684	123	129	1887
7679	3704	1927	7684	123	129	1887
7745	3705	2086	7776	1679	1685	2042
7747	3687	2015	7778	1593	1599	1973
7696	3590	1732	7708	1908	1914	1738
7693	3574	1716	7704	1888	1894	1722
7693	3574	1716	7704	1888	1894	1722
7689	3577	1700	7700	1888	1894	1706
7698	3566	1814	7703	2027	2033	1806
7721	3598	1992	7732	1879	1885	1979
7760	3648	2128	7775	2000	2006	2109
7768	3645	2179	7789	1977	1983	2158
7768	3645	2179	7789	1977	1983	2158
7767	3635	2182	7792	2058	2064	2153
7775	3623	2146	7799	2012	2018	2125
7733	3650	2108	7757	1910	1916	2089
7743	3738	2070	7781	1921	1927	2049
7811	3682	2137	7821	1866	1872	2122
7810	3682	2142	7818	1919	1925	2127
7666	3548	1817	7647	2101	2107	1804
7687	3544	1828	7667	2121	2127	1815
7674	3586	1743	7656	2047	2053	1720

7676	3586	1745	7658	2049	2055	1722
7684	3586	1763	7664	2081	2087	1738
7649	3586	1755	7628	2093	2099	1730
7678	3576	1843	7654	2138	2144	1808
7680	3576	1843	7656	2138	2144	1808
7687	3602	1788	7659	2083	2089	1756
7689	3604	1790	7661	2085	2091	1758
7687	3604	1790	7659	2085	2091	1758
7685	3628	1813	7671	2106	2112	1781
7689	3611	1795	7662	2080	2086	1763
7665	3585	1807	7644	2074	2080	1775
7666	3586	1808	7645	2075	2081	1776
7697	3610	1673	7679	2084	2090	1648
7698	3608	1667	7683	2074	2080	1642
7714	3564	1871	7693	2118	2124	1881
7716	3564	1873	7695	2118	2124	1881
7706	3556	1860	7685	2109	2115	1870
7705	3555	1862	7684	2109	2115	1872
7718	3545	1882	7697	2137	2143	1892
7705	3555	1862	7684	2109	2115	1872
7706	3556	1863	7685	2110	2116	1873
7718	3570	1877	7697	2124	2130	1887
7705	3561	1903	7700	2156	2162	1913
7698	3572	1888	7677	2157	2163	1931
7793	3769	1895	7798	2367	2373	1952
7798	3771	1903	7805	2381	2387	1968
7808	3787	1911	7815	2385	2391	1968
7784	3796	1886	7793	2348	2354	1943
7778	3706	1708	7784	2224	2230	1765
7776	3701	1699	7775	2231	2237	1756
7776	3701	1699	7775	2231	2237	1756
7773	3699	1697	7772	2229	2235	1754
7780	3707	1711	7779	2239	2245	1768
7759	3732	1695	7776	2215	2221	1752
7784	3727	1683	7785	2205	2211	1740
7735	3772	1745	7749	2285	2291	1802
7754	3708	1776	7757	2254	2260	1833
7782	3731	1671	7783	2201	2207	1728
7785	3759	1710	7797	2224	2230	1743
7787	3754	1694	7799	2226	2232	1751
7789	3759	1699	7801	2231	2237	1756
7770	3746	1702	7782	2218	2224	1767
7780	3706	1692	7782	2228	2234	1749
7771	3699	1687	7773	2225	2231	1744
7747	3710	1638	7743	2182	2188	1697
7750	3708	1639	7745	2193	2199	1698
7703	3807	1843	7747	2391	2397	1906
7712	3816	1852	7756	2400	2406	1915
7696	3800	1836	7740	2384	2390	1899
7737	3740	1727	7748	2253	2259	1770
7750	3718	1646	7746	2203	2209	1705
7751	3723	1647	7747	2203	2209	1706
7630	3809	1929	7655	2459	2465	1980
7734	3696	1781	7730	2314	2320	1850
7722	3691	1780	7715	2319	2325	1849

7731	3715	1790	7726	2330	2336	1859
7738	3710	1793	7731	2333	2339	1862
7727	3718	1778	7740	2303	2309	1847
7754	3742	1715	7744	2272	2278	1774
7742	3721	1708	7732	2265	2271	1767
7740	3719	1706	7730	2263	2269	1765
7743	3708	1656	7737	2186	2192	1715
7730	3713	1642	7742	2197	2203	1701
7768	3708	1674	7763	2199	2205	1733
7761	3701	1665	7758	2192	2198	1724
7730	3713	1586	7734	2104	2110	1643
7730	3713	1586	7734	2104	2110	1643
7748	3710	1687	7746	2244	2250	1746
7743	3705	1683	7741	2239	2245	1742
7746	3708	1686	7744	2242	2248	1745
7616	3545	1913	7632	2194	2200	1916
7613	3547	1914	7629	2195	2201	1917
7604	3535	1901	7619	2182	2188	1904
7695	3712	2078	7675	2355	2361	2065
7618	3561	1933	7631	2179	2185	1920
7857	3784	2196	7850	2452	2458	2183
7795	3411	3761	7807	3836	3842	3791
7795	6629	6646	7986	6757	6763	6673
7797	6631	6648	7988	6759	6765	6675
7794	6628	6645	7985	6756	6762	6672
7796	6630	6647	7987	6758	6764	6674
7755	6768	6707	7978	6820	6826	6734
7761	6758	6698	7970	6809	6815	6725
7768	6764	6700	7976	6813	6819	6727
7751	6804	6738	7956	6849	6855	6765
7768	6818	6752	7973	6863	6869	6779
7755	6781	6733	7960	6839	6845	6760
7766	6860	6770	7973	6877	6883	6797
7744	6784	6722	7949	6835	6841	6749
7773	6813	6752	7978	6865	6871	6779
7735	6775	6705	7938	6816	6822	6732
7746	6790	6724	7951	6831	6837	6751
7750	6790	6728	7955	6841	6847	6755
7875	6983	6864	8072	6967	6973	6896
7870	6978	6859	8067	6962	6968	6891
7852	6945	6832	8051	6933	6939	6860
7774	6785	6742	7989	6839	6845	6772
7882	6922	6848	8075	6952	6958	6880
7749	6788	6733	7966	6838	6844	6763
7743	6782	6727	7960	6832	6838	6757
7744	6787	6726	7961	6829	6835	6756
7705	6768	6698	7923	6805	6811	6726
7636	6817	6746	7853	6851	6857	6774
7844	4164	3589	7897	3808	3814	3567
7831	4153	3578	7884	3797	3803	3556
3435	7535	7569	3416	7577	7583	7541
3435	7535	7569	3416	7577	7583	7541
3434	7534	7568	3415	7576	7582	7540
3434	7533	7567	3415	7575	7581	7539
3434	7535	7569	3415	7577	7583	7541

3432	7533	7567	3413	7575	7581	7539
3434	7535	7569	3415	7577	7583	7541
3432	7533	7567	3413	7575	7581	7539
3435	7536	7570	3416	7578	7584	7542
3432	7533	7567	3413	7575	7581	7539
3431	7532	7566	3412	7574	7580	7538
3432	7533	7567	3413	7575	7581	7539
3433	7534	7568	3414	7576	7582	7540
3433	7534	7568	3414	7576	7582	7540
3300	7619	7677	3294	7714	7720	7681
3304	7623	7681	3298	7718	7724	7685
3304	7623	7681	3298	7718	7724	7685
3307	7626	7684	3301	7721	7727	7688
3367	7559	7636	3360	7675	7681	7640
3301	7620	7678	3295	7715	7721	7682
3299	7613	7669	3293	7708	7714	7673
3297	7611	7667	3291	7706	7712	7671
3301	7616	7672	3295	7711	7717	7676
3296	7611	7667	3290	7706	7712	7671
3298	7612	7669	3292	7708	7714	7673
3298	7618	7673	3294	7712	7718	7677
3390	7513	7523	3377	7565	7571	7527
3493	7534	7584	3483	7623	7629	7580
3517	7532	7582	3526	7627	7633	7578
3401	7693	7706	3318	7758	7764	7706
3401	7693	7706	3318	7758	7764	7706
3409	7700	7711	3326	7763	7769	7711
3417	7752	7748	3322	7793	7799	7744
3327	7687	7720	3340	7781	7787	7719
651	7580	7608	2462	7621	7627	7601
654	7583	7611	2465	7624	7630	7604
644	7590	7616	2467	7633	7639	7609
822	7577	7610	2469	7628	7634	7599
661	7563	7587	2359	7605	7611	7580
669	7571	7595	2367	7613	7619	7588
660	7562	7586	2358	7604	7610	7579
35	7626	7649	2451	7681	7687	7642
37	7628	7651	2453	7683	7689	7644
42	7632	7655	2458	7687	7693	7648
	7645	7668	2470	7700	7706	7661
2323	7597	7605	2517	7633	7639	7587
2324	7596	7606	2518	7634	7640	7588
2324	7598	7606	2518	7634	7640	7588
2325	7599	7607	2519	7635	7641	7589
2323	7597	7605	2517	7633	7639	7587
2322	7599	7607	2519	7635	7641	7589
2599	7576	7591	2602	7656	7662	7588
2598	7575	7590	2601	7655	7661	7587
2597	7574	7589	2600	7654	7660	7586
2596	7573	7588	2599	7653	7659	7585
2598	7575	7590	2601	7655	7661	7587
2597	7574	7589	2600	7654	7660	7586
2629	7557	7568	2631	7631	7637	7565
2600	7577	7592	2603	7657	7663	7589
2620	7663	7696	2615	7725	7731	7691

2467	7699	7686	23	7697	7703	7675
2470	7702	7689		7700	7706	7678
2468	7700	7687	28	7698	7704	7676
2467	7697	7684	43	7695	7701	7673
25566	24921	25009	25572	24972	24976	25017
25565	24920	25008	25571	24971	24975	25016
31219	30913	30937	31239	30947	30951	30937

NC_STEC228	CFSAN061770	MS7925	OH-17-6342	FSIS1703155	KPPUTH06	NC_STEC242
3731	6870	3577	6769	3740	3737	3707
3671	6835	3502	6719	3688	3685	3640
3671	6839	3502	6723	3688	3685	3640
3721	6845	3563	6735	3737	3734	3679
3704	6844	3565	6734	3709	3706	3668
3738	6846	3582	6730	3738	3735	3687
3738	6846	3582	6730	3738	3735	3687
3730	6872	3558	6756	3726	3723	3695
3732	6874	3561	6758	3728	3725	3697
3765	6854	3585	6769	3765	3762	3721
3771	6858	3606	6775	3773	3770	3720
3758	6883	3600	6795	3758	3755	3705
3832	6890	3683	6810	3854	3851	3784
3832	6890	3683	6810	3854	3851	3784
3762	6866	3612	6784	3784	3781	3729
3759	6878	3609	6796	3781	3778	3726
3654	6829	3503	6720	3668	3665	3605
3709	6818	3520	6716	3719	3716	3654
3756	6860	3628	6781	3784	3781	3702
3758	6879	3658	6793	3785	3782	3750
3797	6894	3631	6796	3813	3810	3762
3646	6825	3506	6729	3660	3657	3660
3750	6829	3612	6732	3772	3769	3699
3782	6887	3626	6799	3798	3795	3721
3655	6828	3555	6717	3681	3678	3613
3699	6860	3544	6768	3708	3705	3648
3630	6877	3562	6763	3644	3641	3640
3757	6862	3623	6698	3789	3786	3733
3746	6855	3644	6764	3737	3734	3708
3690	6830	3543	6729	3683	3680	3635
3690	6830	3543	6729	3683	3680	3635
3692	6831	3546	6730	3685	3682	3639
3658	6871	3533	6761	3690	3689	3630
3622	6871	3562	6757	3634	3631	3638
3622	6871	3562	6757	3634	3631	3638
1920	6726	1881	6650	1864	1861	2327
1945	6716	1871	6646	1889	1886	2310
1929	6721	1864	6651	1873	1870	2309
1934	6704	1893	6634	1880	1877	2286
1909	6712	1847	6636	1841	1838	2312
1935	6708	1848	6638	1881	1878	2324
1934	6707	1847	6637	1880	1877	2323
1914	6729	1876	6650	1856	1853	2316
1945	6742	1886	6668	1895	1892	2323
1979	6695	1899	6636	1927	1924	2338
2061	6717	1981	6654	2010	2007	2413
1992	6836	1962	6767	1972	1969	2386
1829	6729	1804	6673	1874	1871	2113
1824	6724	1799	6668	1869	1866	2108
1830	6730	1805	6674	1875	1872	2114
1824	6724	1799	6668	1869	1866	2108
1829	6729	1804	6673	1874	1871	2113
1829	6768	1779	6690	1907	1904	2050

1960	6894	1804	6796	1925	1922	2161
1800	6786	1833	6715	1781	1778	2154
1799	6785	1832	6714	1780	1777	2153
1792	6777	1827	6706	1773	1770	2148
1794	6780	1829	6709	1775	1772	2150
1746	6807	1793	6721	1747	1744	2111
1755	6816	1802	6730	1756	1753	2120
1755	6816	1802	6730	1756	1753	2120
1719	6782	1794	6711	1720	1717	2092
1744	6797	1815	6726	1745	1742	2109
1746	6772	1795	6705	1737	1734	2121
1711	6774	1786	6703	1712	1709	2084
1740	6784	1820	6713	1731	1728	2117
1740	6784	1820	6713	1731	1728	2117
1729	6773	1809	6702	1720	1717	2106
1687	6770	1828	6698	1686	1683	2128
1842	6846	1855	6776	1778	1775	2013
1873	6874	1886	6804	1809	1806	2044
1844	6848	1857	6778	1780	1777	2015
1842	6846	1855	6776	1778	1775	2013
2228	6881	2125	6809	2246	2243	2004
2231	6883	2127	6811	2248	2245	2006
2225	6877	2121	6805	2242	2239	2000
2222	6874	2118	6802	2239	2236	1997
2204	6885	2129	6811	2229	2226	2029
2220	6878	2118	6806	2237	2234	2005
2224	6876	2120	6804	2241	2238	1999
2229	6887	2135	6815	2248	2245	1988
2224	6876	2120	6804	2241	2238	1999
2223	6875	2119	6803	2240	2237	1998
2234	6874	2118	6804	2237	2234	1953
2211	6861	2181	6789	2232	2229	1985
2230	6880	2120	6808	2247	2244	2008
2230	6880	2120	6808	2247	2244	2008
2230	6880	2120	6808	2247	2244	2008
2187	6858	2150	6779	2188	2185	1489
2120	6858	2060	6779	2103	2100	1588
1835	6724	2016	6679	1873	1870	2193
1833	6728	2006	6683	1869	1866	2181
1833	6728	2006	6683	1869	1866	2181
1823	6724	2010	6675	1859	1856	2169
1974	6855	1901	6761	1935	1932	1957
2179	6857	1907	6790	2207	2204	245
2297	6842	2027	6775	2335	2332	
2326	6848	2060	6781	2396	2393	151
2326	6848	2060	6781	2396	2393	151
2345	6859	2088	6796	2395	2392	214
2311	6856	2053	6789	2361	2358	174
2293	6847	2079	6780	2351	2348	301
2296	6812	2166	6757	2346	2343	710
2349	6909	2226	6846	2313	2310	1709
2326	6920	2263	6857	2306	2303	1764
1934	6778	48	6717	1858	1855	2007
1947	6798		6737	1871	1868	2027
1873	6790	142	6735	1805	1802	1955

1877	6793	144	6738	1809	1806	1957
1885	6791	190	6734	1831	1828	1973
1922	6813	508	6754	1863	1860	2022
1964	6816	511	6759	1930	1927	2053
1964	6818	511	6761	1930	1927	2053
1958	6840	571	6775	1922	1919	2072
1960	6842	573	6777	1924	1921	2074
1960	6842	573	6777	1924	1921	2074
1970	6847	585	6782	1934	1931	2092
1959	6835	576	6770	1923	1920	2079
1965	6851	628	6784	1957	1954	2085
1966	6852	629	6785	1958	1955	2086
1798	6805	855	6755	1763	1760	1996
1808	6803	851	6753	1773	1770	2000
1934	6790	1870	6725	1928	1925	2211
1936	6790	1872	6725	1930	1927	2213
1926	6782	1864	6717	1920	1917	2203
1925	6781	1863	6716	1919	1916	2202
1945	6766	1855	6707	1931	1928	2222
1925	6781	1865	6716	1919	1916	2202
1926	6782	1864	6717	1920	1917	2203
1940	6796	1878	6731	1934	1931	2217
1925	6795	1903	6730	1927	1924	2240
1946	6831	1906	6745	1940	1937	2265
411	6801	2113	6737	592	589	2443
431	6808	2118	6741	612	609	2459
425	6818	2129	6754	606	603	2457
430	6802	2092	6738	607	604	2430
155	6859	1956	6795	352	349	2300
118	6861	1951	6795	313	310	2287
118	6861	1951	6795	313	310	2287
116	6858	1949	6792	311	308	2285
122	6867	1955	6803	317	314	2299
171	6865	1932	6801	350	347	2288
172	6865	1927	6801	337	334	2283
312	6833	2014	6796	477	474	2364
217	6856	1933	6787	382	379	2336
106	6852	1929	6788	279	276	2287
160	6887	1941	6810	336	333	2304
117	6879	1952	6815	293	290	2304
122	6885	1957	6821	298	295	2309
173	6863	1944	6799	323	320	2312
33	6866	1952	6802	270	267	2302
	6857	1947	6793	259	256	2297
255	6840	1836	6776	112	109	2283
256	6845	1836	6781	113	110	2284
442	6778	1975	6792	321	318	2442
451	6787	1984	6801	330	327	2451
435	6771	1968	6785	314	311	2435
395	6830	1885	6798	278	275	2330
266	6836	1844	6778	107	104	2294
266	6837	1845	6779	107	104	2294
682	6780	2021	6719	541	538	2558
436	6834	1873	6760	257	254	2398
427	6827	1868	6753	246	243	2395

445	6832	1890	6763	264	261	2406
440	6843	1885	6769	259	256	2409
434	6776	1884	6788	245	242	2413
344	6863	1897	6799	161	158	2365
337	6856	1887	6792	140	137	2358
335	6854	1885	6790	138	135	2356
263	6840	1831	6776	105	102	2273
259	6839	1837	6775	112	109	2284
301	6860	1839	6796	138	135	2299
292	6853	1832	6789	129	126	2294
471	6810	1740	6764	340	337	2229
471	6810	1740	6764	340	337	2229
261	6843	1873	6779	14	11	2337
256	6838	1868	6774	9		2332
259	6841	1871	6777		9	2335
1969	6745	1864	6664	1875	1872	2279
1970	6744	1865	6663	1876	1873	2280
1957	6733	1852	6652	1863	1860	2267
2082	6838	2088	6767	2006	2003	2394
1945	6761	1886	6690	1833	1830	2254
2221	6999	2086	6926	2147	2144	2494
3896	7088	3820	6962	3922	3919	3848
6743	272	6682	491	6727	6724	6720
6745	274	6684	493	6729	6726	6722
6742	271	6681	490	6726	6723	6719
6744	273	6683	492	6728	6725	6721
6807	161	6752	379	6791	6788	6776
6795	150	6746	368	6779	6776	6760
6807	154	6748	372	6793	6790	6768
6817	82	6772	302	6801	6798	6800
6831	98	6786	318	6815	6812	6816
6796	138	6749	326	6780	6777	6792
6857		6798	362	6841	6838	6842
6809	86	6764	286	6793	6790	6792
6839	116	6794	316	6823	6820	6822
6810	103	6751	301	6794	6791	6775
6807	90	6768	288	6791	6788	6790
6815	92	6770	292	6799	6796	6798
6987	378	6920	535	6973	6970	6945
6982	373	6915	530	6968	6965	6940
6941	364	6871	498	6927	6924	6911
6850	299	6791	280	6834	6831	6807
6953	441	6897	454	6939	6936	6917
6831	257	6773	125	6815	6812	6806
6825	251	6767	119	6809	6806	6800
6824	258	6766	122	6808	6805	6799
6793	362	6737		6777	6774	6775
6843	533	6781	287	6825	6822	6842
3644	6948	3376	6907	3677	3674	3688
3633	6938	3365	6897	3666	3663	3677
7713	7900	7552	7860	7676	7673	7696
7713	7900	7552	7860	7676	7673	7696
7712	7899	7551	7859	7675	7672	7695
7711	7898	7550	7858	7674	7671	7694
7713	7900	7552	7860	7676	7673	7696

7711	7900	7550	7860	7674	7671	7694
7713	7900	7552	7860	7676	7673	7696
7711	7898	7550	7858	7674	7671	7694
7714	7901	7553	7861	7677	7674	7697
7711	7898	7550	7858	7674	7671	7694
7710	7897	7549	7857	7673	7670	7693
7711	7898	7550	7858	7674	7671	7694
7712	7899	7551	7859	7675	7672	7695
7712	7899	7551	7859	7675	7672	7695
7801	7939	7701	7897	7766	7763	7792
7805	7943	7705	7901	7770	7767	7796
7805	7943	7705	7901	7770	7767	7796
7808	7946	7708	7904	7773	7770	7799
7762	7945	7658	7907	7727	7724	7747
7802	7940	7702	7898	7767	7764	7793
7791	7931	7693	7890	7756	7753	7784
7789	7930	7691	7889	7754	7751	7782
7794	7934	7696	7893	7759	7756	7787
7789	7929	7691	7888	7754	7751	7782
7791	7931	7693	7890	7756	7753	7784
7796	7934	7698	7893	7761	7758	7790
7637	7890	7590	7837	7592	7589	7669
7714	7871	7601	7835	7678	7675	7696
7714	7871	7611	7829	7678	7675	7696
7864	7966	7752	7921	7829	7826	7828
7864	7966	7752	7921	7829	7826	7828
7869	7973	7757	7928	7834	7831	7833
7902	8009	7795	7966	7866	7863	7866
7874	7949	7770	7899	7840	7837	7842
7698	7710	7620	7653	7672	7669	7697
7701	7713	7623	7656	7675	7672	7700
7706	7721	7630	7664	7680	7677	7707
7711	7698	7625	7641	7685	7682	7712
7679	7741	7594	7684	7652	7649	7672
7687	7749	7602	7692	7660	7657	7680
7678	7740	7593	7683	7651	7648	7671
7752	7747	7668	7686	7727	7724	7741
7754	7749	7670	7688	7729	7726	7743
7758	7754	7674	7693	7733	7730	7747
7771	7766	7687	7705	7746	7743	7760
7674	7750	7586	7702	7646	7643	7697
7675	7751	7587	7703	7647	7644	7698
7675	7751	7587	7703	7647	7644	7698
7676	7752	7588	7704	7648	7645	7699
7674	7750	7586	7702	7646	7643	7697
7676	7752	7588	7704	7648	7645	7699
7710	7787	7599	7743	7682	7679	7708
7709	7786	7598	7742	7681	7678	7707
7708	7785	7597	7741	7680	7677	7706
7707	7784	7596	7740	7679	7676	7705
7709	7786	7598	7742	7681	7678	7707
7708	7785	7597	7741	7680	7677	7706
7685	7812	7574	7768	7657	7654	7689
7711	7788	7600	7744	7683	7680	7709
7793	7808	7709	7754	7767	7764	7791

7770	7968	7664	7918	7741	7738	7772
7773	7973	7667	7923	7744	7741	7775
7771	7971	7665	7921	7742	7739	7773
7768	7966	7662	7916	7739	7736	7770
25043	25028	24981	25010	25034	25033	24969
25042	25027	24980	25009	25033	25032	24968
30994	30804	31020	30787	30991	30988	30967

AZ-TG98487	KTE187	KTE141	KTE126	UMEA
3604	7604	7605	7691	3555
3527	7542	7541	7642	3470
3527	7544	7543	7642	3470
3570	7569	7568	7665	3529
3539	7595	7594	7679	3496
3563	7550	7549	7636	3524
3563	7550	7549	7636	3524
3580	7577	7576	7679	3527
3583	7581	7580	7681	3529
3623	7535	7534	7626	3585
3638	7528	7527	7649	3610
3603	7581	7580	7671	3570
3722	7616	7615	7722	3640
3722	7616	7615	7722	3640
3632	7578	7577	7676	3575
3629	7583	7582	7680	3564
3520	7541	7540	7643	3460
3571	7563	7562	7667	3484
3602	7569	7568	7680	3573
3633	7537	7536	7650	3619
3674	7593	7592	7699	3615
3564	7544	7543	7647	3490
3632	7554	7553	7659	3604
3646	7590	7589	7682	3617
3525	7536	7535	7633	3518
3583	7597	7596	7699	3544
3525	7587	7588	7706	3477
3650	7606	7607	7717	3587
3636	7544	7545	7667	3580
3564	7556	7557	7667	3478
3564	7556	7557	7667	3478
3568	7558	7559	7669	3480
3577	7579	7580	7693	3497
3531	7581	7582	7702	3465
3531	7581	7582	7702	3465
1874	7611	7612	7689	148
1865	7611	7612	7694	129
1855	7612	7613	7693	117
1852	7622	7623	7696	164
1857	7597	7598	7680	103
1867	7614	7615	7693	5
1864	7613	7614	7692	
1853	7612	7613	7689	139
1881	7609	7610	7684	177
1911	7567	7568	7664	205
1968	7566	7567	7640	422
1953	7606	7607	7693	1780
1739	7579	7580	7638	1778
1734	7576	7577	7635	1773
1740	7582	7583	7641	1779
1734	7576	7577	7635	1773
1739	7581	7582	7640	1778
1515	7605	7606	7692	1768

1788	7643	7644	7730	1975
552	7618	7619	7700	1845
551	7617	7618	7699	1844
544	7612	7613	7694	1837
546	7614	7615	7696	1839
473	7604	7605	7689	1799
482	7613	7614	7698	1808
482	7613	7614	7698	1808
340	7584	7585	7664	1800
377	7587	7588	7675	1831
383	7567	7568	7648	1791
332	7576	7577	7656	1792
	7610	7611	7688	1864
	7610	7611	7688	1864
27	7600	7601	7678	1853
318	7605	7606	7686	1812
1514	7578	7579	7662	1852
1545	7606	7607	7691	1883
1516	7580	7581	7664	1854
1514	7578	7579	7662	1852
1915	7635	7636	7701	2201
1917	7639	7640	7703	2203
1911	7633	7634	7697	2197
1908	7630	7631	7694	2194
1912	7639	7640	7705	2198
1906	7630	7631	7696	2192
1910	7632	7633	7696	2196
1899	7633	7634	7697	2205
1910	7632	7633	7696	2196
1909	7631	7632	7695	2195
1928	7622	7623	7696	2208
1969	7643	7644	7707	2217
1912	7642	7643	7681	2216
1912	7642	7643	7681	2216
1912	7642	7643	7681	2216
2063	7696	7697	7773	2270
1978	7688	7689	7775	2232
1761	7589	7590	7705	1950
1745	7588	7589	7701	1942
1745	7588	7589	7701	1942
1729	7585	7586	7697	1943
1847	7616	7617	7700	2036
1973	7651	7652	7729	2242
2117	7697	7698	7772	2323
2166	7715	7716	7786	2374
2166	7715	7716	7786	2374
2161	7712	7713	7789	2365
2133	7717	7718	7796	2320
2080	7690	7691	7754	2363
2051	7704	7705	7779	2362
2145	7765	7766	7818	2418
2150	7758	7759	7815	2440
1809	7568	7569	7644	1834
1820	7586	7587	7664	1847
1737	7574	7575	7653	1840

1739	7576	7577	7655	1844
1757	7584	7585	7661	1875
1763	7564	7565	7625	1948
1849	7582	7583	7651	1891
1849	7584	7585	7653	1891
1806	7603	7604	7656	1922
1808	7605	7606	7658	1924
1808	7603	7604	7656	1924
1831	7599	7600	7668	1939
1813	7603	7604	7659	1911
1821	7585	7586	7641	1943
1822	7586	7587	7642	1944
1725	7589	7590	7676	1853
1719	7590	7591	7680	1843
1878	7643	7644	7690	1938
1880	7645	7646	7692	1940
1867	7635	7636	7682	1930
1869	7634	7635	7681	1929
1889	7645	7646	7694	1927
1869	7634	7635	7681	1929
1870	7635	7636	7682	1930
1884	7649	7650	7694	1944
1910	7632	7633	7697	1960
1920	7633	7634	7674	2033
1938	7679	7680	7795	2137
1946	7686	7687	7802	2141
1954	7694	7695	7812	2155
1929	7670	7671	7790	2130
1751	7679	7680	7781	1947
1742	7675	7676	7772	1940
1742	7675	7676	7772	1940
1740	7672	7673	7769	1938
1754	7679	7680	7776	1952
1738	7660	7661	7773	1934
1726	7683	7684	7782	1916
1788	7644	7645	7746	2015
1805	7662	7663	7754	1954
1724	7685	7686	7780	1908
1729	7690	7691	7794	1913
1747	7692	7693	7796	1937
1752	7694	7695	7798	1942
1755	7671	7672	7779	1919
1745	7683	7684	7779	1949
1740	7674	7675	7770	1934
1683	7647	7648	7740	1840
1684	7650	7651	7742	1852
1876	7607	7608	7744	2012
1885	7616	7617	7753	2021
1869	7600	7601	7737	2005
1754	7643	7644	7745	1939
1691	7646	7647	7743	1848
1692	7649	7650	7744	1849
1938	7553	7554	7652	2077
1812	7638	7639	7727	1933
1811	7626	7627	7712	1932

1821	7637	7638	7723	1940
1824	7642	7643	7728	1945
1823	7630	7631	7737	1932
1760	7654	7655	7741	1919
1753	7642	7643	7729	1912
1751	7640	7641	7727	1910
1701	7643	7644	7734	1852
1687	7632	7633	7739	1856
1719	7667	7668	7760	1870
1710	7660	7661	7755	1861
1627	7633	7634	7731	1798
1627	7633	7634	7731	1798
1732	7648	7649	7743	1882
1728	7643	7644	7738	1877
1731	7646	7647	7741	1880
1941	7524	7525	7629	1820
1942	7521	7522	7626	1821
1929	7511	7512	7616	1808
2079	7573	7574	7672	2058
1934	7527	7528	7628	1867
2214	7768	7769	7847	2152
3784	7753	7754	7804	3835
6660	7765	7766	7981	6589
6662	7767	7768	7983	6591
6659	7764	7765	7980	6588
6661	7766	7767	7982	6590
6721	7739	7740	7973	6652
6712	7745	7746	7965	6639
6714	7754	7755	7971	6647
6752	7733	7734	7951	6673
6766	7748	7749	7968	6687
6747	7737	7738	7955	6663
6784	7750	7751	7968	6707
6736	7724	7725	7944	6665
6766	7753	7754	7973	6695
6719	7715	7716	7933	6648
6738	7726	7727	7946	6669
6742	7730	7731	7950	6671
6881	7859	7860	8067	6849
6876	7854	7855	8062	6844
6845	7832	7833	8046	6797
6759	7763	7764	7984	6685
6863	7869	7870	8070	6819
6748	7749	7750	7961	6667
6742	7743	7744	7955	6661
6741	7744	7745	7956	6660
6713	7702	7703	7918	6637
6755	7651	7652	7848	6683
3618	7770	7771	7895	3488
3607	7759	7760	7882	3477
7560	3426	3427	3413	7608
7560	3426	3427	3413	7608
7559	3425	3426	3412	7607
7558	3425	3426	3412	7606
7560	3425	3426	3412	7608

7558	3425	3426	3410	7606
7560	3425	3426	3412	7608
7558	3423	3424	3410	7606
7561	3426	3427	3413	7609
7558	3423	3424	3410	7606
7557	3422	3423	3409	7605
7558	3423	3424	3410	7606
7559	3424	3425	3411	7607
7559	3424	3425	3411	7607
7694	3289	3290	3291	7719
7698	3293	3294	3295	7723
7698	3293	3294	3295	7723
7701	3296	3297	3298	7726
7649	3359	3360	3357	7680
7695	3290	3291	3292	7720
7686	3302	3303	3290	7712
7684	3300	3301	3288	7710
7689	3302	3303	3292	7715
7684	3297	3298	3287	7710
7686	3301	3302	3289	7712
7690	3301	3302	3291	7716
7549	3381	3382	3374	7576
7596	3442	3443	3480	7625
7594	3502	3503	3523	7625
7722	3464	3465	3315	7752
7722	3464	3465	3315	7752
7727	3471	3472	3323	7757
7764	3473	3474	3319	7794
7742	3443	3444	3337	7753
7613	2339	2340	2459	7627
7616	2342	2343	2462	7630
7621	2344	2345	2464	7635
7611	2382	2383	2466	7636
7592	2304	2305	2356	7606
7600	2312	2313	2364	7614
7591	2303	2304	2355	7605
7654	2304	2305	2448	7676
7656	2306	2307	2450	7678
7660	2311	2312	2455	7682
7673	2323	2324	2467	7695
7610		1	2514	7613
7611	1		2515	7614
7611	3	4	2515	7614
7612	6	7	2516	7615
7610	4	5	2514	7613
7612	39	40	2516	7615
7604	2625	2626	2599	7617
7603	2624	2625	2598	7616
7602	2623	2624	2597	7615
7601	2622	2623	2596	7614
7603	2624	2625	2598	7616
7602	2623	2624	2597	7615
7581	2652	2653	2628	7596
7605	2626	2627	2600	7618
7700	2557	2558	2612	7705

7688	2514	2515		7692
7691	2517	2518	23	7695
7689	2515	2516	25	7693
7686	2515	2516	40	7690
25028	25497	25498	25570	24987
25027	25496	25497	25569	24986
30936	31225	31226	31237	31012

	C6466	C6468	C6473	C6518	C6842	C6847	C6894	C6895	C6949
**C6466**		3593	3613	3404	809	24961	3597	3554	3588
**C6468**	3593		2067	3853	3505	24975	1813	1969	1946
**C6473**	3613	2067		3810	3520	24976	1819	1948	1977
**C6518**	3404	3853	3810		3352	25018	3763	3823	3788
**C6842**	809	3505	3520	3352		24901	3484	3469	3540
**C6847**	24961	24975	24976	25018	24901		25051	24987	24974
**C6894**	3597	1813	1819	3763	3484	25051		1778	1902
**C6895**	3554	1969	1948	3823	3469	24987	1778		1798
**C6949**	3588	1946	1977	3788	3540	24974	1902	1798	
**C6950**	24960	24974	24975	25017	24900	1	25050	24986	24973
**C7030**	3740	1805	1904	3917	3671	25038	1849	1844	1914
**C7031**	3745	2028	2246	3908	3659	24986	2284	2442	2333
**C7032**	7604	7588	7599	7753	7541	25497	7605	7611	7606
**C7136**	3512	1875	1851	3692	3445	24932	1906	1812	1762
**C7143**	3553	1347	1985	3818	3484	24983	1902	2034	1983
**C7145**	6979	6843	6952	7176	6934	25057	6866	6859	6932
**C7148**	3565	1742	1784	3765	3485	24999	1528	1813	1894
**C7257**	3692	2174	2127	3837	3604	24973	2065	2380	2397
**C7259_L33**	3692	2174	2127	3837	3604	24973	2065	2380	2397
**C7277**	3658	2086	1905	3847	3555	25036	1618	1993	2016
**C7279**	4192	3637	3298	4449	4169	25029	3429	3512	3562
**C7328**	3760	1886	2104	3834	3670	24969	2074	2215	2244
**C7347**	7646	7689	7690	7800	7587	25557	7705	7709	7678
**C7349**	3584	1909	1857	3812	3494	24978	1655	1862	1817
**C7369**	3737	1845	2006	3900	3662	25050	1831	1934	2008
**C7382**	3742	1871	1936	3924	3670	25038	1908	1866	1974
**C7570**	7556	7620	7618	7733	7522	25479	7613	7638	7637
**C7962**	3549	2028	1987	3807	3514	25051	1953	2052	1975
**C7963**	7692	7702	7669	7805	7644	25569	7693	7690	7694
**C7963**	7692	7702	7669	7805	7644	25569	7693	7690	7694
**C7968**	3407	1866	1839	3666	3333	25016	1448	1795	1850
**C7969**	30934	30957	31009	31054	30907	27325	30973	31010	30961
**C7970**	7607	7684	7674	7861	7571	25535	7695	7717	7696
**C7971**	7754	7777	7775	7964	7705	25610	7812	7793	7798
**C7973**	987	3558	3612	3441	1078	24938	3518	3474	3530
**C7974**	987	3558	3612	3441	1078	24938	3518	3474	3530
**C7975**	7577	7626	7620	7778	7528	25525	7626	7625	7606
**C7979**	7703	7772	7755	7931	7650	25585	7778	7757	7765
**C8124**	3753	1964	2007	3876	3704	25019	1970	1952	2036
**KCh007**	3727	1829	2000	3893	3654	25040	1827	1918	2000
**HE-MDREc53**	3761	1887	2105	3835	3671	24971	2075	2216	2245
**KFu023**	1064	3633	3630	3177	450	24932	3606	3611	3669
**KFu031**	7509	7596	7588	7769	7466	25510	7607	7624	7620
**KMi011**	6787	6712	6817	6993	6740	25023	6725	6686	6798
**Kmi017**	6800	6682	6813	7022	6753	25015	6722	6684	6794
**KMi024**	1209	3541	3543	3448	711	24914	3558	3493	3546
**KFu021**	3857	2011	2070	3974	3780	25044	2017	2027	2101
**Kmi017**	6829	6712	6843	7051	6782	25045	6752	6713	6824
**KMi019**	3738	2186	2047	3946	3680	25174	2179	2147	1935
**KTa003**	7507	7598	7598	7769	7464	25514	7609	7624	7620
**JSWP001**	6778	6656	6795	7006	6733	25040	6698	6670	6772
**JSWP014**	7531	7578	7539	7767	7479	25532	7603	7615	7580
**JSWP021**	3765	1892	2110	3840	3675	24974	2079	2221	2250
**1657**	3684	2078	2065	3808	3615	24944	2147	2050	1998

**5051**	3499	1965	1967	3809	3467	24980	1791	195	1816
**VREC0418**	3544	1919	1903	3714	3487	24961	1988	1875	1778
**VREC0426**	3658	1783	1772	3791	3542	25016	1533	1820	1919
**VREC0506**	3523	1949	1939	3788	3486	25032	1922	1945	1964
**VREC0559**	7701	7745	7732	7925	7648	25595	7770	7757	7755
**C-0863N0015**	7557	7600	7594	7756	7508	25545	7600	7603	7580
**AMA940**	3563	1957	353	3761	3513	24978	1776	1911	1947
**703**	3556	1943	1940	3836	3461	24988	1769	149	1781
**998**	3536	1985	1980	3771	3474	24960	1800	253	1850
**1599**	3520	1974	1965	3805	3451	24958	1836	196	1810
**VRES0107**	3563	1957	353	3760	3513	24975	1776	1911	1947
**EC-F86E-R-141010**	6791	6688	6806	7013	6737	25018	6733	6671	6795
**412049521**	7544	7653	7637	7834	7512	25546	7660	7685	7662
**VREC0504**	3532	1960	1946	3793	3495	25041	1933	1956	1975
**EC362**	3759	1883	2101	3833	3667	24969	2071	2212	2243
**MS2481**	7530	7577	7538	7766	7478	25531	7602	7614	7579
**2010031282**	7531	7578	7539	7767	7479	25532	7603	7615	7580
**SCK02-43**	3735	2028	2281	3903	3659	25012	2307	2458	2351
**SCP21-24**	3593	10	2067	3853	3505	24973	1813	1969	1946
**SCP29-34**	3531	1973	1965	3812	3490	25036	1942	1959	1958
**ERS1340929**	7564	7623	7619	7721	7530	25521	7625	7630	7619
**ERS1340998**	3768	1876	2120	3832	3678	24967	2076	2225	2258
**ERS1341034**	3773	2099	2084	3995	3735	25035	2111	2074	2206
**SCK53-23**	7708	7750	7737	7929	7655	25600	7775	7762	7760
**SCP24-18**	7689	7700	7666	7802	7641	25572	7690	7687	7691
**E.**	6821	6726	6804	7022	6773	25033	6748	6701	6825
**509sc**	3536	1925	1931	3810	3456	24988	1767	191	1792
**ME160327**	3596	2043	40	3802	3522	24978	1794	1940	1952
**AM_LREC-61**	7610	7687	7677	7864	7574	25539	7698	7720	7699
**AM_LREC-98**	7534	7581	7542	7770	7482	25535	7606	7618	7583
**AM_LREC-15**	7614	7693	7683	7867	7578	25553	7706	7725	7706
**AM_LREC-63**	7613	7692	7682	7866	7577	25552	7705	7724	7705
**AM_LREC-128**	7617	7696	7686	7870	7581	25555	7709	7728	7709
**AM_LREC-109**	7606	7684	7674	7860	7570	25535	7695	7717	7696
**ERS1724554**	3685	2213	2147	3829	3591	24978	2082	2367	2445
**EDZFRVQ5**	7605	7589	7600	7754	7542	25498	7606	7612	7607
**ME160633**	3803	1959	2162	4030	3734	25029	2013	2135	2147
**ME160685**	7615	7674	7666	7776	7567	25549	7665	7676	7673
**1508493**	3768	1893	2113	3836	3681	24972	2071	2202	2246
**ERS1801995**	3748	1813	1905	3921	3671	25042	1850	1848	1925
**ERS1812824**	3765	1887	1964	3899	3680	25037	1929	1887	1990
**20151201**	3787	1834	1941	3895	3704	25059	1821	1911	2001
**MT66C.C1**	3591	2040	40	3797	3523	24979	1799	1937	1947
**DTU2017-812-PR**	3787	1847	1962	3902	3710	25061	1841	1930	2000
**DTU2017-821-PRJ1111**	7617	7696	7686	7870	7581	25553	7709	7728	7709
**PA20B**	6649	6614	6735	6930	6600	25061	6660	6610	6722
**1351**	6806	6680	6817	7026	6759	25017	6718	6688	6798
**2011-70-34-3**	3736	1809	1907	3904	3651	25033	1869	1844	1933
**2011-70-41-2**	859	3549	3589	3376	378	24918	3562	3536	3598
**DTU2011_26**	3525	1888	1862	3705	3457	24941	1919	1825	1775
**2011-70-219-2**	729	3603	3590	3410	852	24932	3535	3516	3571
**HVH**	7605	7682	7672	7859	7569	25532	7693	7715	7694
**ESC0167**	1040	3630	3675	3515	536	24931	3638	3628	3641
**ESC0211**	6974	6838	6947	7171	6929	25052	6861	6854	6927
**ESC0198**	3759	1769	1831	3935	3672	25029	1803	1814	1856

**AZ-TG60445**	7612	7688	7678	7866	7576	25538	7701	7721	7700
**AZ-TG60412**	3782	1842	1957	3897	3705	25056	1836	1925	1995
**2-316-03_S3_C3**	3720	1888	2006	3867	3707	25037	1886	1960	2017
**7-233-03_S3_**	904	3584	3642	3399	410	24943	3617	3576	3623
**2-316-03_S1_C2**	6938	6805	6911	7141	6877	25043	6832	6820	6916
**2-460-02_S1_C2**	7531	7578	7539	7767	7479	25532	7603	7615	7580
**AZ-TG73171**	7620	7699	7689	7873	7584	25558	7712	7731	7712
**AZ-TG73251**	7571	7628	7626	7728	7537	25525	7630	7635	7626
**AZ-TG73331**	3720	1838	2008	3905	3663	25045	1828	1943	2001
**AZ-TG73319**	3588	1823	659	3865	3532	25027	1675	1847	1883
**AZ-TG73315**	7701	7745	7732	7925	7648	25595	7770	7757	7755
**AZ-TG73343**	3748	1961	2133	3923	3657	24964	2067	2272	2235
**AZ-TG73651**	3736	1838	1977	3901	3661	25045	1834	1929	1997
**AZ-TG73483**	3786	1896	1976	3910	3692	25045	1940	1896	1999
**blood-09-1294**	6646	6611	6732	6927	6597	25057	6657	6607	6719
**blood-10-1009**	908	3606	3616	3372	271	24917	3582	3570	3609
**upec-128**	7617	7676	7668	7778	7569	25551	7667	7678	7675
**upec-203**	3767	1889	1966	3903	3682	25040	1933	1889	1992
**upec-205**	3721	1881	2115	3828	3627	24970	2065	2228	2237
**upec-3**	3596	2043	40	3802	3522	24978	1794	1940	1952
**upec-33**	3649	1774	1763	3782	3533	25008	1524	1811	1911
**AZ-TG71327**	3727	1829	2000	3893	3654	25040	1827	1918	2000
**AZ-TG71195**	3526	1993	1941	3790	3482	25028	1967	1973	2011
**AZ-TG71423**	3574	2031	139	3760	3502	24968	1820	1964	1958
**NA**	6806	6688	6819	7028	6759	25023	6728	6690	6800
**12_ECOL**	7621	7680	7672	7782	7573	25555	7671	7682	7679
**184_ECOL**	7533	7580	7541	7769	7481	25534	7605	7617	7582
**283_ECOL**	6647	6612	6733	6928	6598	25059	6658	6608	6720
**775_SBOY**	6791	6665	6800	7003	6744	25010	6705	6667	6777
**966_ECOL**	6648	6613	6734	6929	6599	25058	6659	6609	6721
**C260_92**	828	3525	3539	3344	346	24897	3525	3481	3540
**IHD45_5**	3821	1977	2178	4046	3752	25050	2029	2151	2165
**IHD717_3**	3761	1815	1951	3920	3686	25056	1815	1908	1970
**IHD717_9**	3671	2060	1972	3836	3557	24959	1947	2246	2275
**IHD717_16**	3787	2149	2196	3805	3695	24971	2043	2374	2440
**IHD813_9**	1050	3700	3729	3526	412	24952	3689	3649	3725
**IHD813_16**	1050	3700	3729	3526	412	24952	3689	3649	3725
**AZ_TG76998**	7561	7620	7616	7718	7527	25519	7622	7627	7616
**H124600634**	3556	1935	1941	3840	3473	24989	1757	127	1781
**H134240608**	944	3648	3645	3410	296	24920	3646	3623	3691
**AZ-TG71191**	3583	2057	473	3780	3491	24986	1768	1950	1981
**2012C-3377**	831	3580	3582	3405	346	24938	3578	3537	3595
**2013C-4350**	3756	1819	1901	3924	3671	25041	1860	1852	1934
**MGH108**	1243	3693	3718	3526	1205	24889	3636	3606	3643
**AZ-TG71543**	3801	1958	1995	3936	3734	25021	1989	1958	2018
**AZ-TG71539**	6786	6705	6810	6984	6739	25019	6718	6679	6791
**AZ-TG71555**	3524	1887	1863	3704	3455	24942	1918	1824	1774
**SEQ895**	6828	6712	6835	7050	6787	25033	6752	6706	6816
**AZ_TG78596**	3604	1745	1831	3784	3520	25028	1515	1874	1953
**JEONG-9567**	686	3581	3605	3417	809	24927	3569	3495	3547
**KCJ1232**	3658	1783	1772	3791	3542	25017	1533	1820	1920
**FSIS**	957	3527	3554	3409	538	24900	3515	3491	3542
**AZ684313**	7532	7579	7540	7768	7480	25533	7604	7616	7581
**10B06797**	7530	7579	7540	7768	7480	25533	7604	7616	7581
**cam_1531_1**	7533	7580	7541	7767	7481	25534	7605	7617	7582

**Ia_1424**	7531	7578	7539	7767	7479	25532	7603	7615	7580
**HICF2**	6814	6698	6821	7036	6773	25019	6738	6692	6802
**HICF32**	3689	1885	2113	3783	3615	24998	2095	2236	2275
**HICF112**	3848	2002	2061	3965	3771	25036	2008	2018	2092
**HICF191**	3594	2041	38	3800	3520	24976	1792	1938	1950
**GN02531**	7606	7589	7601	7755	7543	25505	7607	7613	7608
**GN03624**	3779	1980	2016	3888	3711	25034	1982	1952	2060
**KCJ3858**	3583	1969	451	3838	3497	24991	1696	1877	1910
**CFSAN045100**	3589	1771	1814	3786	3477	25020	1603	1856	1947
**AZ-TG-WCHI-3**	6772	6650	6790	6992	6731	25033	6694	6658	6766
**IEH-NGS-ECO-00205**	841	3587	3617	3396	385	24930	3599	3584	3617
**F283**	7532	7579	7540	7768	7480	25533	7604	7616	7581
**HS115**	7531	7578	7539	7767	7479	25532	7603	7615	7580
**J21**	7515	7547	7557	7797	7451	25544	7570	7581	7556
**KO178B**	7533	7580	7541	7769	7481	25534	7605	7617	7582
**KO198B**	7533	7580	7541	7769	7481	25534	7605	7617	7582
**AZ-TG-WCHI-8**	3762	1902	2158	3848	3662	24963	2096	2233	2240
**AZ-TG-713-2**	3841	1995	2054	3958	3764	25029	2001	2011	2085
**KCJ9492**	3830	1952	2123	4033	3761	25040	1982	2126	2128
**MOD1-EC5105**	3547	1945	1950	3831	3465	24984	1769	75	1778
**MOD1-EC5111**	3520	1933	1929	3814	3456	24971	1759	107	1769
**MOD1-EC5122**	3586	1766	1811	3781	3474	25016	1600	1851	1942
**CFSAN041116**	3590	1772	1815	3787	3478	25018	1604	1857	1948
**MOD1-EC5135**	3761	1887	2105	3835	3671	24971	2075	2216	2245
**MOD1-EC5153**	3532	1958	1946	3795	3495	25041	1931	1954	1973
**MOD1-EC5167**	3524	1950	1938	3787	3487	25035	1923	1946	1965
**246164**	3518	2051	2072	3758	3474	24986	1870	460	1922
**ESC_DA2647AA**	3784	1977	2013	3895	3716	25026	1979	1957	2057
**195745**	3406	1865	1838	3665	3332	25011	1447	1794	1849
**181089**	3583	1939	487	3830	3497	24982	1696	1842	1912
**KCJK2721**	3613	1703	1760	3772	3505	25005	1519	1812	1895
**MOD1-EC7010**	1073	3610	3642	3449	535	24927	3612	3595	3627
**MOD1-EC6900**	6781	6706	6811	6987	6734	25018	6719	6680	6792
**KCJK4181**	683	3578	3602	3415	806	24923	3567	3493	3544
**MOD1-EC6811**	3725	1827	1998	3891	3652	25038	1825	1916	1998
**MOD1-EC6825**	7575	7624	7618	7776	7526	25523	7624	7623	7604
**KCJK4201**	683	3578	3602	3415	806	24923	3567	3493	3544
**CFSAN046659**	3726	1833	1937	3925	3691	25047	1885	1884	1936
**MOD1-ECOR25**	830	3523	3539	3346	348	24901	3525	3483	3540
**MOD1-ECOR34**	3759	1769	1831	3935	3672	25029	1803	1814	1856
**MOD1-EC6368**	3748	1816	1879	3920	3670	25028	1854	1845	1928
**MOD1-EC6936**	3764	1862	1943	3911	3683	25047	1834	1925	1963
**MOD1-EC6953**	3401	1860	1833	3660	3327	25010	1442	1789	1844
**MOD1-EC6831**	6924	6825	6942	7136	6877	25056	6842	6830	6911
**MOD1-EC6779**	3799	1974	2170	4031	3736	25037	2021	2141	2156
**MOD1-EC6577**	884	3546	3581	3407	172	24912	3533	3495	3577
**MOD1-EC5070**	3744	1889	2055	3944	3675	24987	1993	2236	2138
**MOD1-EC6716**	4181	3626	3287	4438	4158	25018	3418	3501	3551
**MOD1-EC6835**	3593	1734	1820	3774	3509	25015	1504	1863	1942
**MOD1-EC6847**	3588	74	2071	3852	3514	24983	1787	1956	1924
**MOD1-EC6868**	7576	7625	7619	7777	7527	25524	7625	7624	7605
**MOD1-EC6870**	3793	1884	1911	3923	3706	25027	1916	1939	2011
**MOD1-EC6885**	3759	1815	1983	3914	3688	25058	1805	1914	1974
**MOD1-EC6891**	7554	7608	7598	7713	7516	25523	7610	7614	7606
**MOD1-EC6897**	902	3637	3660	3417	261	24917	3616	3576	3652

**MOD1-EC6802**	836	3540	3592	3420	361	24904	3539	3513	3558
**MOD1-EC6938**	3590	1815	660	3877	3536	25029	1669	1859	1873
**MOD1-EC6943**	3760	1967	2003	3871	3697	25017	1969	1939	2041
**MOD1-EC6946**	7574	7623	7617	7775	7525	25522	7623	7622	7603
**MOD1-EC6966**	834	3562	3617	3429	367	24925	3561	3543	3576
**MOD1-EC6978**	834	3562	3617	3429	367	24925	3561	3543	3576
**MOD1-EC6529**	7545	7599	7589	7705	7507	25515	7601	7605	7597
**MOD1-EC6332**	3523	1949	1937	3786	3486	25034	1922	1945	1964
**MOD1-EC6385**	3583	1941	489	3830	3497	24985	1700	1846	1916
**MOD1-EC5700**	993	3558	3615	3453	1076	24946	3516	3482	3536
**MOD1-EC5706**	1047	3621	3625	3474	536	24940	3640	3614	3618
**MOD1-EC5440**	3605	1695	1752	3764	3497	24998	1511	1804	1887
**MOD1-EC5737**	3406	1865	1838	3665	3332	25015	1447	1794	1849
**MOD1-EC5722**	3765	1830	1942	3913	3689	25042	1795	1925	1984
**MOD1-EC3793**	3675	2136	2127	3820	3574	24984	2026	2334	2384
**MOD1-EC54**	7605	7682	7672	7859	7569	25534	7693	7715	7694
**MOD1-EC5522**	3808	1891	2073	3979	3741	25044	1879	2009	2073
**MOD1-EC5194**	7574	7623	7617	7775	7525	25522	7623	7622	7603
**MOD1-EC5196**	7573	7622	7616	7774	7524	25521	7622	7621	7602
**Ecol_583**	3759	1885	2103	3833	3669	24967	2073	2214	2243
**MOD1-EC3564**	3572	1995	569	3820	3491	24972	1768	1866	1950
**18.1-R1**	6785	6662	6778	7004	6741	25031	6705	6668	6779
**KCJK1916**	3689	1885	2113	3783	3615	24998	2095	2236	2275
**KCJK1866**	3689	1885	2113	3783	3615	24998	2095	2236	2275
**MOD1-EC707**	7575	7624	7618	7776	7526	25523	7624	7623	7604
**MOD1-EC1626**	3719	1824	1930	3918	3684	25040	1876	1875	1929
**MOD1-EC1634**	3761	1820	1902	3927	3676	25038	1860	1853	1935
**MOD1-EC3584**	3401	1860	1833	3660	3327	25010	1442	1789	1844
**CDPHFDLB-F1602032-004B**	833	3579	3585	3407	348	24941	3582	3539	3597
**MOD1-EC1638**	902	3626	3658	3417	251	24927	3613	3574	3655
**29618**	7606	7590	7601	7755	7543	25499	7607	7613	7608
**149438**	3584	1764	1809	3779	3472	25015	1598	1849	1937
**E123**	3605	34	2068	3859	3509	24974	1829	1977	1968
**287552**	3573	2030	138	3759	3501	24966	1819	1963	1957
**140183**	3538	1964	1952	3801	3501	25042	1937	1960	1978
**93272**	3586	1911	1859	3814	3496	24980	1657	1864	1819
**182138**	3584	1909	1857	3812	3494	24978	1655	1862	1817
**272422**	3615	1940	1888	3843	3525	25006	1686	1893	1848
**E2026_9**	6802	6705	6838	6985	6770	25028	6730	6704	6799
**MOD1-EC3605**	871	3629	3657	3509	904	24880	3635	3584	3643
**MOD1-EC3330**	3524	1950	1938	3787	3487	25035	1923	1946	1965
**CFSAN044415**	3713	2120	2143	3818	3621	24967	2044	2377	2326
**MOD1-EC5432**	7546	7600	7590	7706	7508	25516	7602	7606	7598
**UMB12_01.1uot**	7634	7693	7685	7795	7586	25566	7684	7695	7692
**RR1**	1017	3574	3628	3411	518	24921	3573	3555	3578
**UMB08_01.1uog**	3618	1716	1813	3761	3508	25009	1535	1830	1855
**MOD1-EC3102**	7694	7704	7671	7807	7646	25572	7695	7692	7696
**NC_P10-04**	3762	1888	2106	3836	3672	24972	2076	2217	2246
**NC_P19-11**	3768	1894	2112	3842	3678	24976	2082	2223	2252
**NC_STEC173**	3636	1722	1781	3791	3528	25017	1540	1843	1924
**NC_STEC228**	3731	1833	1970	3896	3654	25043	1829	1920	1992
**CFSAN061770**	6870	6728	6847	7088	6829	25028	6768	6726	6836
**MS7925**	3577	2006	585	3820	3503	24981	1779	1881	1962
**OH-17-6342**	6769	6683	6782	6962	6720	25010	6690	6650	6767
**FSIS1703155**	3740	1869	1934	3922	3668	25034	1907	1864	1972

**KPPUTH06**	3737	1866	1931	3919	3665	25033	1904	1861	1969
**NC_STEC242**	3707	2181	2092	3848	3605	24969	2050	2327	2386
**AZ-TG98487**	3604	1745	1831	3784	3520	25028	1515	1874	1953
**KTE187**	7604	7588	7599	7753	7541	25497	7605	7611	7606
**KTE141**	7605	7589	7600	7754	7540	25498	7606	7612	7607
**KTE126**	7691	7701	7668	7804	7643	25570	7692	7689	7693
**UMEA**	3555	1942	1939	3835	3460	24987	1768	148	1780

C6950	C7030	C7031	C7032	C7136	C7143	C7145	C7148	C7257	C7259_L33	C7277	C7279
24960	3740	3745	7604	3512	3553	6979	3565	3692	3692	3658	4192
24974	1805	2028	7588	1875	1347	6843	1742	2174	2174	2086	3637
24975	1904	2246	7599	1851	1985	6952	1784	2127	2127	1905	3298
25017	3917	3908	7753	3692	3818	7176	3765	3837	3837	3847	4449
24900	3671	3659	7541	3445	3484	6934	3485	3604	3604	3555	4169
1	25038	24986	25497	24932	24983	25057	24999	24973	24973	25036	25029
25050	1849	2284	7605	1906	1902	6866	1528	2065	2065	1618	3429
24986	1844	2442	7611	1812	2034	6859	1813	2380	2380	1993	3512
24973	1914	2333	7606	1762	1983	6932	1894	2397	2397	2016	3562
	25037	24985	25496	24931	24982	25056	24998	24972	24972	25035	25028
25037		2263	7647	1819	1897	6970	1689	2344	2344	1875	3646
24985	2263		7765	2360	1956	7011	2110	1702	1702	2250	3813
25496	7647	7765		7511	7616	7859	7567	7715	7715	7643	7770
24931	1819	2360	7511		1892	6836	1906	2310	2310	2045	3462
24982	1897	1956	7616	1892		6960	1783	1996	1996	1984	3641
25056	6970	7011	7859	6836	6960		6880	6951	6951	7013	7063
24998	1689	2110	7567	1906	1783	6880		2170	2170	1717	3564
24972	2344	1702	7715	2310	1996	6951	2170			2164	3684
24972	2344	1702	7715	2310	1996	6951	2170			2164	3684
25035	1875	2250	7643	2045	1984	7013	1717	2164	2164		3541
25028	3646	3813	7770	3462	3641	7063	3564	3684	3684	3541	
24968	2180	1864	7631	2180	2025	6965	1897	1975	1975	2261	3806
25556	7769	7850	2557	7627	7714	7897	7664	7810	7810	7737	7865
24977	1735	2145	7578	1793	1862	6944	1465	2054	2054	1838	3562
25049	315	2369	7679	1957	1980	6997	1768	2326	2326	1958	3668
25037	114	2315	7648	1865	1937	6975	1738	2398	2398	1927	3678
25478	7689	7764	2382	7541	7629	7804	7572	7720	7720	7673	7774
25050	1892	2291	7633	1900	1936	6896	1913	2290	2290	2009	3572
25568	7741	7819	2515	7617	7701	8070	7649	7787	7787	7731	7896
25568	7741	7819	2515	7617	7701	8070	7649	7787	7787	7731	7896
25015	1831	2251	7582	1897	1821	6833	1746	2103	2103	1692	3509
27324	31004	30941	31225	30908	30950	30795	30919	30961	30961	31031	30974
25534	7760	7839	3302	7638	7683	8037	7646	7791	7791	7747	7870
25609	7866	7927	3473	7720	7775	8100	7722	7872	7872	7857	7938
24937	3637	3639	7581	3480	3487	6972	3470	3631	3631	3597	4186
24937	3637	3639	7581	3480	3487	6972	3470	3631	3631	3597	4186
25524	7689	7771	2626	7551	7620	7883	7564	7727	7727	7673	7825
25584	7840	7903	3443	7699	7751	8050	7694	7851	7851	7825	7930
25018	247	2393	7638	1931	2017	6986	1820	2461	2461	1987	3666
25039	309	2357	7675	1943	1972	6989	1756	2320	2320	1958	3652
24970	2181	1865	7632	2181	2026	6966	1898	1976	1976	2262	3807
24931	3783	3796	7537	3540	3596	6977	3623	3739	3739	3706	4269
25509	7678	7754	3442	7549	7589	7998	7551	7703	7703	7649	7776
25022	6814	6881	7749	6685	6794	434	6742	6812	6812	6831	6914
25014	6792	6869	7724	6675	6799	391	6724	6798	6798	6836	6920
24913	3671	3677	7544	3433	3525	6941	3558	3657	3657	3644	4165
25043	308	2469	7616	1975	2085	6898	1883	2526	2526	2067	3756
25044	6822	6899	7753	6705	6829	421	6754	6828	6828	6866	6950
25173	2103	2566	7768	811	2287	7092	2185	2537	2537	2391	3755
25513	7678	7758	3502	7545	7593	7992	7549	7707	7707	7651	7784
25039	6786	6845	7754	6659	6779	435	6702	6774	6774	6816	6958
25531	7678	7698	3425	7503	7549	8009	7514	7701	7701	7617	7790
24973	2186	1869	7635	2186	2031	6971	1903	1981	1981	2266	3812
24943	1974	2489	7573	569	2118	6931	2067	2445	2445	2272	3689

24979	1853	2425	7609	1805	2049	6851	1780	2382	2382	1988	3503
24960	1787	2397	7527	378	1981	6854	1922	2299	2299	2103	3529
25015	1708	2173	7613	1907	1861	6901	239	2175	2175	1747	3543
25031	1871	2235	7634	1858	1859	6905	1856	2227	2227	1955	3548
25594	7829	7891	3464	7686	7744	8062	7680	7835	7835	7815	7894
25544	7663	7745	2652	7525	7594	7907	7540	7707	7707	7651	7853
24977	1889	2216	7582	1819	1907	6919	1823	2076	2076	1856	3353
24987	1841	2419	7614	1809	2037	6850	1792	2375	2375	1976	3489
24959	1885	2445	7567	1819	2090	6817	1828	2389	2389	1986	3478
24957	1840	2391	7622	1837	2051	6837	1783	2337	2337	2007	3479
24974	1889	2218	7584	1819	1907	6921	1823	2076	2076	1856	3352
25017	6777	6867	7737	6688	6807	429	6735	6798	6798	6845	6935
25545	7731	7804	3359	7605	7648	8051	7613	7754	7754	7712	7847
25040	1882	2246	7645	1869	1870	6914	1865	2238	2238	1966	3559
24968	2177	1871	7630	2177	2022	6968	1894	1982	1982	2258	3803
25530	7677	7697	3422	7502	7548	8008	7513	7700	7700	7616	7789
25531	7678	7698	3423	7503	7549	8009	7514	7701	7701	7617	7790
25011	2260	160	7758	2372	1965	7022	2115	1757	1757	2260	3817
24972	1805	2028	7588	1875	1347	6843	1742	2174	2174	2086	3637
25035	1885	2237	7645	1872	1865	6914	1868	2247	2247	1969	3546
25520	7679	7763	2342	7537	7633	7807	7577	7708	7708	7673	7769
24966	2190	1870	7633	2188	2035	6977	1887	1989	1989	2277	3814
25034	494	2527	7553	2067	2144	6881	1938	2611	2611	2117	3747
25599	7834	7896	3471	7691	7749	8069	7685	7840	7840	7820	7899
25571	7738	7816	2515	7614	7698	8065	7646	7784	7784	7728	7893
25032	6829	6885	7651	6722	6800	702	6753	6844	6844	6844	6959
24987	1814	2419	7612	1771	2051	6846	1803	2367	2367	1952	3469
24977	1881	2222	7603	1840	1961	6947	1761	2105	2105	1884	3291
25538	7763	7842	3302	7641	7686	8040	7649	7794	7794	7750	7873
25534	7681	7701	3426	7506	7552	8012	7517	7704	7704	7620	7793
25552	7771	7848	3290	7647	7692	8046	7655	7800	7800	7758	7876
25551	7770	7847	3289	7646	7691	8045	7654	7799	7799	7757	7875
25554	7774	7851	3293	7650	7695	8049	7658	7803	7803	7761	7878
25534	7760	7839	3301	7638	7683	8037	7646	7791	7791	7747	7870
24977	2341	1778	7712	2317	2015	6962	2161	237	237	2205	3655
25497	7648	7766	5	7512	7617	7860	7568	7716	7716	7644	7771
25028	544	2491	7679	2091	2146	6934	1952	2464	2464	2137	3729
25548	7728	7792	2304	7585	7679	7856	7615	7749	7749	7709	7825
24971	2161	1895	7639	2181	2052	6975	1900	2006	2006	2262	3799
25041	33	2266	7650	1829	1902	6975	1690	2347	2347	1878	3655
25036	140	2338	7640	1887	1961	6984	1757	2425	2425	1941	3694
25058	285	2346	7690	1968	1987	7015	1746	2337	2337	1929	3627
24978	1874	2225	7603	1833	1962	6940	1766	2108	2108	1887	3286
25060	247	2359	7694	1963	1998	7015	1758	2342	2342	1964	3652
25552	7774	7851	3293	7648	7695	8049	7658	7803	7803	7761	7879
25060	6728	6775	7767	6615	6727	571	6650	6712	6712	6764	6898
25016	6790	6873	7726	6677	6801	391	6726	6796	6796	6838	6916
25032	64	2255	7643	1839	1893	6970	1707	2336	2336	1893	3664
24917	3725	3707	7569	3488	3516	6972	3551	3672	3672	3617	4186
24940	1832	2373	7521	33	1905	6847	1919	2323	2323	2058	3475
24931	3693	3706	7579	3517	3549	6972	3527	3625	3625	3622	4155
25531	7758	7837	3300	7636	7681	8036	7644	7789	7789	7745	7868
24930	3801	3739	7590	3562	3632	7002	3623	3714	3714	3719	4234
25051	6965	7006	7854	6831	6955	23	6875	6946	6946	7008	7058
25028	266	2208	7633	1793	1895	6929	1635	2278	2278	1887	3610

25537	7765	7843	3301	7643	7687	8040	7650	7797	7797	7751	7874
25055	242	2354	7692	1958	1993	7009	1753	2337	2337	1959	3647
25036	320	2382	7662	1968	2023	6984	1793	2367	2367	1975	3636
24942	3789	3747	7535	3523	3587	6969	3597	3720	3720	3674	4241
25042	6924	6969	7832	6805	6924	150	6834	6917	6917	6967	7017
25531	7678	7698	3425	7503	7549	8011	7514	7701	7701	7617	7790
25557	7777	7854	3296	7653	7698	8052	7661	7806	7806	7764	7881
25524	7684	7768	2344	7544	7640	7815	7582	7715	7715	7680	7779
25044	318	2376	7679	1944	1991	6989	1765	2325	2325	1961	3659
25026	1725	2123	7589	1759	1808	6916	1652	2015	2015	1854	3384
25594	7829	7891	3464	7686	7744	8062	7680	7835	7835	7815	7894
24963	2144	1599	7696	2108	1865	6955	2032	1462	1462	2226	3673
25044	262	2354	7683	1962	1979	6996	1751	2331	2331	1965	3651
25044	149	2347	7654	1896	1970	6993	1766	2434	2434	1954	3703
25056	6725	6772	7764	6612	6724	568	6647	6709	6709	6761	6895
24916	3776	3746	7569	3547	3602	6930	3598	3697	3697	3681	4227
25550	7730	7794	2306	7587	7681	7858	7617	7751	7751	7711	7827
25039	142	2340	7642	1889	1963	6986	1759	2427	2427	1947	3696
24969	2177	1837	7622	2129	2010	6966	1898	1930	1930	2194	3789
24977	1881	2222	7605	1840	1961	6947	1761	2105	2105	1884	3291
25007	1699	2164	7604	1898	1852	6892	230	2166	2166	1738	3534
25039	309	2357	7675	1943	1972	6989	1756	2320	2320	1958	3652
25027	1916	2275	7632	1897	1899	6919	1897	2271	2271	2002	3585
24967	1903	2262	7586	1860	1979	6955	1779	2095	2095	1894	3262
25022	6798	6875	7730	6681	6805	397	6730	6804	6804	6842	6926
25554	7734	7798	2311	7591	7685	7863	7621	7755	7755	7715	7831
25533	7680	7700	3425	7505	7551	8011	7516	7703	7703	7619	7792
25058	6726	6773	7765	6613	6725	569	6648	6710	6710	6762	6896
25009	6793	6852	7715	6658	6782	392	6707	6781	6781	6819	6919
25057	6727	6774	7766	6614	6726	570	6649	6711	6711	6763	6897
24896	3690	3656	7542	3450	3492	6972	3510	3635	3635	3576	4161
25049	562	2505	7694	2109	2160	6951	1968	2478	2478	2153	3745
25055	217	2335	7685	1931	1974	6982	1728	2318	2318	1930	3626
24958	2155	1854	7651	2168	1963	6960	1991	396	396	2089	3687
24970	2292	1741	7704	2370	2021	6913	2061	745	745	2154	3711
24951	3867	3820	7616	3637	3694	7009	3705	3779	3779	3759	4290
24951	3867	3820	7616	3637	3694	7009	3705	3779	3779	3759	4290
25518	7676	7760	2339	7534	7630	7804	7574	7705	7705	7670	7766
24988	1833	2417	7612	1805	2033	6854	1786	2362	2362	1970	3497
24919	3815	3782	7593	3579	3640	6988	3617	3759	3759	3705	4267
24985	1847	2193	7564	1855	1903	6920	1733	2051	2051	1858	3334
24937	3747	3709	7577	3489	3561	7009	3563	3690	3690	3631	4200
25040	67	2275	7646	1829	1911	6966	1697	2359	2359	1885	3650
24888	3804	3748	7606	3623	3638	6995	3623	3724	3724	3712	4196
25020	248	2385	7630	1985	2016	6936	1813	2478	2478	2017	3732
25018	6807	6874	7744	6678	6787	431	6735	6805	6805	6824	6905
24941	1831	2372	7524	32	1904	6848	1918	2322	2322	2057	3474
25032	6814	6891	7748	6705	6829	389	6754	6822	6822	6866	6944
25027	1683	2145	7610	1929	1847	6881	383	2166	2166	1788	3618
24926	3699	3683	7558	3510	3559	6959	3560	3628	3628	3640	4169
25016	1708	2173	7613	1907	1861	6901	239	2175	2175	1747	3543
24899	3674	3653	7536	3461	3533	6945	3506	3606	3606	3610	4140
25532	7679	7699	3424	7504	7550	8010	7515	7702	7702	7618	7791
25532	7679	7699	3424	7504	7550	8010	7515	7702	7702	7618	7791
25533	7680	7700	3425	7505	7551	8011	7516	7703	7703	7619	7790

25531	7678	7698	3423	7503	7549	8009	7514	7701	7701	7617	7790
25018	6800	6875	7733	6691	6815	373	6740	6806	6806	6852	6930
24997	2187	1870	7642	2216	1973	6970	1900	1985	1985	2186	3790
25035	299	2460	7607	1966	2076	6889	1874	2517	2517	2058	3747
24975	1879	2220	7603	1838	1959	6945	1759	2103	2103	1882	3289
25504	7649	7767	39	7513	7617	7861	7569	7717	7717	7645	7772
25033	263	2410	7642	1947	2027	6995	1832	2478	2478	2001	3680
24990	1794	2208	7584	1776	1854	6894	1753	2006	2006	1768	3371
25019	1732	2143	7617	1919	1845	6884	314	2200	2200	1750	3534
25032	6782	6835	7745	6659	6767	431	6700	6764	6764	6814	6958
24929	3777	3720	7581	3504	3580	6995	3571	3700	3700	3646	4226
25532	7679	7699	3425	7504	7550	8010	7515	7702	7702	7618	7791
25531	7678	7698	3423	7503	7549	8009	7514	7701	7701	7617	7790
25543	7596	7690	3381	7540	7550	7994	7500	7676	7676	7622	7797
25533	7680	7700	3426	7505	7551	8011	7516	7703	7703	7619	7792
25533	7680	7700	3426	7505	7551	8011	7516	7703	7703	7619	7792
24962	2184	1864	7643	2226	2029	6951	1957	1968	1968	2287	3791
25028	292	2453	7600	1959	2069	6882	1867	2510	2510	2051	3740
25039	539	2470	7670	2064	2123	6935	1943	2433	2433	2116	3696
24983	1845	2427	7611	1803	2021	6849	1804	2363	2363	1984	3507
24970	1815	2417	7597	1791	2033	6845	1788	2363	2363	1968	3489
25015	1727	2138	7614	1914	1840	6879	309	2197	2197	1747	3529
25017	1733	2144	7618	1920	1846	6885	315	2201	2201	1751	3535
24970	2181	1865	7632	2181	2026	6966	1898	1976	1976	2262	3807
25040	1880	2244	7643	1867	1868	6914	1865	2236	2236	1964	3557
25034	1872	2236	7635	1859	1860	6906	1857	2228	2228	1956	3549
24985	1969	2494	7566	1892	2166	6835	1906	2448	2448	2021	3560
25025	268	2407	7637	1944	2024	6984	1829	2475	2475	1998	3676
25010	1830	2250	7579	1896	1820	6832	1745	2102	2102	1691	3508
24981	1768	2178	7574	1763	1840	6891	1735	1988	1988	1735	3338
25004	1672	2105	7584	1869	1787	6881	75	2141	2141	1710	3546
24926	3771	3714	7554	3535	3581	6942	3611	3692	3692	3702	4224
25017	6808	6875	7743	6679	6788	428	6736	6806	6806	6825	6908
24922	3697	3680	7556	3508	3556	6958	3557	3624	3624	3637	4167
25037	307	2355	7672	1941	1970	6986	1754	2318	2318	1956	3650
25522	7687	7769	2624	7549	7618	7881	7562	7725	7725	7671	7823
24922	3697	3680	7556	3508	3556	6958	3557	3624	3624	3637	4167
25046	76	2282	7667	1851	1925	6990	1725	2360	2360	1911	3682
24900	3690	3656	7544	3448	3494	6976	3510	3635	3635	3576	4159
25028	266	2208	7633	1793	1895	6929	1635	2278	2278	1887	3610
25027	53	2268	7632	1829	1904	6969	1693	2351	2351	1878	3656
25046	282	2330	7671	1966	1959	6997	1721	2345	2345	1937	3647
25009	1825	2245	7576	1891	1815	6827	1740	2097	2097	1686	3503
25055	6938	7000	7869	6810	6929	511	6866	6923	6923	6960	7021
25036	564	2507	7686	2106	2159	6941	1960	2480	2480	2143	3730
24911	3724	3704	7563	3498	3526	6945	3542	3647	3647	3610	4202
24986	2051	1668	7688	2008	1902	6955	1949	1585	1585	2151	3696
25017	3635	3802	7759	3451	3630	7053	3553	3673	3673	3530	25
25014	1672	2134	7600	1918	1836	6870	372	2155	2155	1777	3608
24982	1797	2016	7585	1862	1333	6839	1726	2162	2162	2071	3620
25523	7688	7770	2625	7550	7619	7882	7563	7726	7726	7672	7824
25026	256	2340	7643	1870	1980	6947	1776	2415	2415	1968	3702
25057	273	2345	7683	1921	1966	6995	1740	2308	2308	1930	3634
25522	7663	7739	2312	7519	7609	7843	7561	7690	7690	7660	7766
24916	3797	3765	7578	3563	3631	6985	3615	3724	3724	3689	4225

24903	3704	3685	7595	3448	3529	6981	3522	3663	3663	3588	4167
25028	1715	2127	7590	1749	1810	6914	1646	2019	2019	1848	3378
25016	250	2396	7626	1934	2014	6979	1819	2464	2464	1988	3667
25521	7686	7768	2623	7548	7617	7880	7561	7724	7724	7670	7822
24924	3738	3698	7550	3474	3541	6983	3546	3678	3678	3616	4189
24924	3738	3698	7550	3474	3541	6983	3546	3678	3678	3616	4189
25514	7654	7730	2303	7510	7600	7834	7552	7681	7681	7651	7757
25033	1871	2235	7634	1858	1859	6905	1856	2227	2227	1955	3548
24984	1772	2180	7576	1767	1842	6896	1737	1990	1990	1737	3340
24945	3645	3643	7587	3486	3489	6978	3464	3635	3635	3597	4190
24939	3792	3746	7528	3553	3626	6973	3612	3717	3717	3721	4228
24997	1664	2097	7576	1861	1779	6873	67	2133	2133	1702	3538
25014	1830	2250	7581	1896	1820	6832	1745	2102	2102	1691	3508
25041	299	2354	7660	1919	1973	6995	1752	2303	2303	1934	3630
24983	2307	1704	7717	2277	1957	6959	2137	203	203	2122	3654
25533	7758	7837	3297	7636	7681	8035	7644	7789	7789	7745	7868
25043	411	2423	7644	2011	2045	6948	1802	2391	2391	2015	3670
25521	7686	7768	2623	7548	7617	7880	7561	7724	7724	7670	7822
25520	7685	7767	2622	7547	7616	7879	7560	7723	7723	7669	7821
24966	2179	1863	7630	2179	2024	6964	1896	1974	1974	2260	3805
24971	1819	2208	7568	1837	1892	6900	1784	2042	2042	1789	3361
25030	6795	6851	7739	6666	6781	442	6709	6780	6780	6825	6965
24997	2187	1870	7642	2216	1973	6970	1900	1985	1985	2186	3790
24997	2187	1870	7642	2216	1973	6970	1900	1985	1985	2186	3790
25522	7687	7769	2624	7549	7618	7881	7562	7725	7725	7671	7823
25039	67	2275	7660	1844	1916	6983	1716	2355	2355	1902	3677
25037	67	2276	7649	1830	1912	6966	1698	2359	2359	1886	3653
25009	1825	2245	7576	1891	1815	6827	1740	2097	2097	1686	3503
24940	3749	3712	7581	3492	3561	7011	3566	3692	3692	3630	4204
24926	3794	3763	7583	3560	3620	6997	3612	3721	3721	3687	4222
25498	7649	7767	6	7513	7618	7861	7569	7717	7717	7645	7772
25014	1725	2136	7612	1912	1838	6874	307	2195	2195	1745	3527
24973	1825	2048	7589	1889	1369	6839	1758	2190	2190	2100	3649
24965	1902	2261	7585	1859	1978	6954	1778	2094	2094	1893	3261
25041	1886	2250	7649	1873	1874	6920	1871	2242	2242	1970	3563
24979	1737	2147	7580	1795	1864	6946	1467	2056	2056	1840	3564
24977	1735	2145	7578	1793	1862	6944	1465	2054	2054	1838	3562
25005	1766	2176	7606	1824	1893	6972	1496	2085	2085	1869	3593
25027	6833	6873	7763	6682	6808	424	6738	6813	6813	6840	6940
24879	3751	3714	7544	3582	3601	6958	3603	3699	3699	3708	4214
25034	1872	2236	7635	1859	1860	6906	1854	2228	2228	1955	3549
24966	2291	1609	7690	2285	1902	6950	2101	322	322	2174	3688
25515	7655	7731	2304	7511	7601	7835	7553	7682	7682	7652	7758
25565	7747	7811	2323	7604	7698	7875	7634	7768	7768	7728	7844
24920	3710	3682	7597	3535	3566	6983	3570	3645	3645	3666	4164
25008	1638	2137	7605	1901	1814	6864	259	2179	2179	1770	3589
25571	7743	7821	2517	7619	7703	8072	7651	7789	7789	7733	7897
24971	2182	1866	7633	2182	2027	6967	1899	1977	1977	2263	3808
24975	2188	1872	7639	2188	2033	6973	1905	1983	1983	2269	3814
25016	1697	2122	7587	1904	1806	6896	112	2158	2158	1735	3567
25042	255	2349	7674	1957	1974	6987	1746	2326	2326	1960	3644
25027	6840	6909	7750	6733	6855	378	6772	6848	6848	6894	6948
24980	1836	2226	7586	1852	1901	6920	1795	2060	2060	1804	3376
25009	6776	6846	7702	6652	6761	535	6705	6781	6781	6796	6907
25033	112	2313	7646	1863	1935	6973	1737	2396	2396	1925	3677

25032	109	2310	7643	1860	1932	6970	1734	2393	2393	1922	3674
24968	2283	1709	7697	2267	1957	6945	2121	151	151	2161	3688
25027	1683	2145	7610	1929	1847	6881	383	2166	2166	1788	3618
25496	7647	7765	4	7511	7616	7859	7567	7715	7715	7643	7770
25497	7648	7766	5	7512	7617	7860	7568	7716	7716	7644	7771
25569	7740	7818	2514	7616	7700	8067	7648	7786	7786	7730	7895
24986	1840	2418	7613	1808	2036	6849	1791	2374	2374	1975	3488

C7328	C7347	C7349	C7369	C7382	C7570	C7962	C7963	C7963	C7968	C7969	C7970
3760	7646	3584	3737	3742	7556	3549	7692	7692	3407	30934	7607
1886	7689	1909	1845	1871	7620	2028	7702	7702	1866	30957	7684
2104	7690	1857	2006	1936	7618	1987	7669	7669	1839	31009	7674
3834	7800	3812	3900	3924	7733	3807	7805	7805	3666	31054	7861
3670	7587	3494	3662	3670	7522	3514	7644	7644	3333	30907	7571
24969	25557	24978	25050	25038	25479	25051	25569	25569	25016	27325	25535
2074	7705	1655	1831	1908	7613	1953	7693	7693	1448	30973	7695
2215	7709	1862	1934	1866	7638	2052	7690	7690	1795	31010	7717
2244	7678	1817	2008	1974	7637	1975	7694	7694	1850	30961	7696
24968	25556	24977	25049	25037	25478	25050	25568	25568	25015	27324	25534
2180	7769	1735	315	114	7689	1892	7741	7741	1831	31004	7760
1864	7850	2145	2369	2315	7764	2291	7819	7819	2251	30941	7839
7631	2557	7578	7679	7648	2382	7633	2515	2515	7582	31225	3302
2180	7627	1793	1957	1865	7541	1900	7617	7617	1897	30908	7638
2025	7714	1862	1980	1937	7629	1936	7701	7701	1821	30950	7683
6965	7897	6944	6997	6975	7804	6896	8070	8070	6833	30795	8037
1897	7664	1465	1768	1738	7572	1913	7649	7649	1746	30919	7646
1975	7810	2054	2326	2398	7720	2290	7787	7787	2103	30961	7791
1975	7810	2054	2326	2398	7720	2290	7787	7787	2103	30961	7791
2261	7737	1838	1958	1927	7673	2009	7731	7731	1692	31031	7747
3806	7865	3562	3668	3678	7774	3572	7896	7896	3509	30974	7870
	7723	2108	2237	2242	7626	2155	7696	7696	2126	30945	7706
7723		7667	7799	7769	2683	7708	2613	2613	7677	31220	3386
2108	7667		1842	1780	7600	1920	7663	7663	1617	30946	7666
2237	7799	1842		319	7720	1956	7777	7777	1880	31008	7796
2242	7769	1780	319		7687	1942	7744	7744	1877	30995	7758
7626	2683	7600	7720	7687		7636	2467	2467	7569	31187	3308
2155	7708	1920	1956	1942	7636		7675	7675	1808	30999	7676
7696	2613	7663	7777	7744	2467	7675			7642	31234	3291
7696	2613	7663	7777	7744	2467	7675			7642	31234	3291
2126	7677	1617	1880	1877	7569	1808	7642	7642		30972	7641
30945	31220	30946	31008	30995	31187	30999	31234	31234	30972		31164
7706	3386	7666	7796	7758	3308	7676	3291	3291	7641	31164	
7791	3495	7766	7911	7868	3478	7767	3320	3320	7745	31290	1905
3680	7638	3510	3626	3636	7576	3532	7703	7703	3394	30908	7617
3680	7638	3510	3626	3636	7576	3532	7703	7703	3394	30908	7617
7655	2662	7589	7717	7685	2657	7635	2601	2601	7590	31233	3379
7779	3489	7724	7883	7842	3419	7755	3338	3338	7705	31215	2015
2312	7756	1878	496	259	7676	1994	7728	7728	1958	30996	7761
2229	7795	1838	52	315	7716	1958	7773	7773	1874	31009	7796
5	7724	2109	2238	2243	7627	2156	7697	7697	2127	30945	7707
3783	7582	3612	3764	3787	7531	3664	7651	7651	3437	30933	7569
7621	3620	7600	7724	7680	3507	7624	3481	3481	7554	31149	775
6836	7799	6809	6841	6817	7681	6775	7964	7964	6694	30792	7926
6833	7788	6800	6819	6795	7676	6777	7947	7947	6682	30795	7913
3684	7611	3533	3650	3662	7522	3544	7648	7648	3432	30914	7587
2398	7778	1957	519	332	7652	2055	7754	7754	2037	31016	7749
6863	7817	6830	6849	6825	7705	6807	7976	7976	6712	30825	7942
2450	7856	2086	2199	2149	7793	2138	7848	7848	2240	31149	7887
7625	3672	7600	7724	7680	3536	7620	3524	3524	7550	31159	846
6811	7815	6778	6821	6795	7703	6757	7974	7974	6662	30809	7941
7573	3524	7540	7718	7676	3433	7650	3413	3413	7573	31136	262
10	7729	2114	2242	2248	7632	2161	7702	7702	2132	30948	7712
2353	7702	1964	2064	2008	7620	2158	7673	7673	2150	30933	7734

2211	7702	1867	1963	1897	7627	2051	7685	7685	1794	31008	7714
2177	7636	1807	1929	1835	7551	1963	7629	7629	2009	30936	7663
1923	7710	1430	1781	1757	7621	1953	7699	7699	1706	30936	7687
2107	7713	1857	1933	1921	7643	174	7682	7682	1742	30977	7694
7756	3478	7733	7876	7831	3454	7727	3316	3316	7706	31273	1882
7629	2691	7563	7691	7659	2686	7609	2629	2629	7566	31248	3408
2136	7701	1827	1976	1932	7611	1926	7652	7652	1819	31001	7688
2196	7706	1853	1953	1883	7637	2034	7694	7694	1780	31013	7711
2244	7678	1906	1997	1929	7586	2062	7665	7665	1824	30999	7687
2181	7712	1884	1952	1882	7644	2062	7697	7697	1807	30987	7714
2136	7703	1827	1976	1932	7613	1928	7654	7654	1819	30997	7690
6837	7797	6799	6808	6782	7687	6780	7958	7958	6685	30810	7924
7673	3478	7626	7767	7729	3377	7649	3358	3358	7606	31166	182
2116	7724	1868	1944	1932	7654	183	7693	7693	1753	30983	7705
11	7724	2105	2234	2239	7627	2152	7697	7697	2123	30944	7707
7572	3521	7539	7717	7675	3430	7649	3410	3410	7572	31135	259
7573	3522	7540	7718	7676	3431	7650	3411	3411	7573	31136	260
1917	7843	2148	2346	2308	7755	2300	7816	7816	2284	30934	7838
1886	7689	1909	1845	1871	7620	2028	7702	7702	1866	30956	7684
2135	7726	1877	1953	1933	7658	198	7695	7695	1762	30975	7703
7622	2643	7599	7710	7677	409	7634	2463	2463	7570	31216	3281
20	7725	2124	2243	2250	7628	2169	7698	7698	2142	30948	7712
2457	7669	2047	736	543	7570	2139	7653	7653	2125	30989	7634
7761	3486	7738	7881	7836	3462	7732	3324	3324	7711	31276	1889
7693	2613	7660	7774	7741	2467	7672	41	41	7639	31235	3288
6849	7683	6817	6843	6827	7572	6801	7851	7851	6714	30819	7799
2194	7702	1841	1932	1858	7633	2022	7690	7690	1756	31007	7711
2083	7700	1831	1996	1926	7622	1961	7657	7657	1817	31007	7688
7709	3388	7669	7799	7761	3310	7679	3293	3293	7644	31163	20
7576	3525	7543	7721	7679	3434	7653	3414	3414	7576	31139	263
7713	3404	7676	7807	7769	3310	7685	3293	3293	7650	31177	82
7712	3403	7675	7806	7768	3309	7684	3292	3292	7649	31177	81
7716	3407	7679	7810	7772	3313	7688	3296	3296	7653	31182	85
7706	3387	7666	7796	7758	3307	7676	3290	3290	7641	31163	21
2056	7812	2075	2343	2397	7725	2355	7790	7790	2144	30968	7795
7632	2558	7579	7680	7649	2383	7634	2516	2516	7583	31226	3303
2365	7814	2005	337	594	7727	2105	7796	7796	2067	31016	7816
7679	2601	7645	7761	7729	803	7679	2449	2449	7611	31203	3280
47	7733	2089	2218	2231	7634	2148	7706	7706	2115	30944	7716
2191	7772	1736	316	115	7692	1895	7743	7743	1832	31010	7763
2261	7760	1796	393	140	7680	1964	7728	7728	1895	30996	7772
2222	7807	1850	226	338	7725	1954	7795	7795	1819	31016	7799
2078	7700	1836	1995	1925	7622	1964	7660	7660	1822	31002	7686
2229	7809	1852	188	300	7729	1954	7799	7799	1842	31018	7807
7716	3407	7679	7810	7772	3313	7688	3296	3296	7653	31182	85
6757	7819	6714	6757	6731	7729	6709	7986	7986	6594	30821	7943
6829	7788	6798	6817	6793	7678	6779	7949	7949	6686	30797	7913
2184	7764	1747	323	107	7685	1912	7735	7735	1847	31007	7755
3706	7599	3560	3729	3739	7529	3568	7666	7666	3401	30939	7599
2193	7637	1806	1970	1878	7550	1913	7627	7627	1910	30914	7648
3708	7647	3556	3672	3694	7564	3592	7694	7694	3403	30928	7595
7704	3384	7664	7794	7756	3306	7674	3289	3289	7639	31161	8
3750	7636	3642	3790	3800	7550	3647	7683	7683	3528	30954	7612
6960	7892	6939	6992	6970	7799	6891	8065	8065	6828	30788	8032
2102	7748	1719	497	342	7677	1816	7732	7732	1831	31008	7745

7710	3385	7672	7801	7763	3307	7682	3292	3292	7645	31166	29
2224	7807	1847	183	295	7727	1949	7797	7797	1837	31010	7803
2252	7781	1921	283	384	7692	1965	7755	7755	1905	31001	7775
3752	7584	3613	3771	3767	7498	3617	7627	7627	3444	30930	7556
6931	7879	6914	6951	6929	7779	6885	8049	8049	6795	30780	8021
7573	3522	7540	7718	7676	3431	7650	3411	3411	7573	31136	262
7719	3410	7682	7813	7775	3316	7691	3299	3299	7656	31185	88
7631	2647	7606	7715	7682	397	7639	2465	2465	7577	31213	3285
2222	7799	1843	81	354	7718	1965	7782	7782	1881	31011	7802
2082	7714	1623	1810	1765	7632	1927	7677	7677	1776	31002	7702
7756	3478	7733	7876	7831	3454	7727	3316	3316	7706	31273	1882
1677	7795	1932	2191	2190	7704	2100	7774	7774	2106	30950	7767
2226	7802	1847	133	272	7720	1953	7780	7780	1835	31005	7802
2270	7774	1808	402	163	7694	1973	7742	7742	1904	31012	7784
6754	7816	6711	6754	6728	7726	6706	7983	7983	6591	30817	7940
3740	7604	3587	3760	3786	7539	3603	7681	7681	3436	30921	7580
7681	2603	7647	7763	7731	805	7681	2451	2451	7613	31207	3282
2263	7762	1798	395	142	7682	1966	7730	7730	1897	31001	7774
77	7724	2113	2248	2239	7623	2130	7697	7697	2133	30938	7709
2083	7702	1831	1996	1926	7624	1961	7659	7659	1817	31007	7690
1914	7701	1421	1772	1748	7612	1944	7690	7690	1697	30928	7678
2229	7795	1838	52	315	7716	1958	7773	7773	1874	31009	7796
2154	7707	1903	1935	1929	7641	224	7698	7698	1788	30973	7684
2073	7683	1879	2002	1960	7600	1971	7643	7643	1817	31007	7675
6839	7792	6806	6825	6801	7682	6783	7953	7953	6688	30801	7917
7685	2608	7651	7767	7735	810	7685	2456	2456	7617	31209	3287
7575	3524	7542	7720	7678	3433	7652	3413	3413	7575	31138	262
6755	7817	6712	6755	6729	7727	6707	7984	7984	6592	30819	7941
6814	7780	6783	6820	6796	7667	6760	7936	7936	6665	30791	7906
6756	7818	6713	6756	6730	7728	6708	7985	7985	6593	30820	7942
3678	7573	3516	3671	3690	7499	3536	7643	7643	3365	30906	7556
2383	7829	2021	351	608	7742	2121	7813	7813	2083	31031	7831
2199	7804	1828	170	281	7722	1926	7781	7781	1812	31012	7798
1877	7753	1892	2181	2209	7665	2253	7730	7730	2056	30967	7747
1919	7787	2148	2304	2347	7696	2256	7780	7780	2083	30948	7758
3840	7671	3706	3844	3856	7604	3691	7723	7723	3543	30934	7653
3840	7671	3706	3844	3856	7604	3691	7723	7723	3543	30934	7653
7619	2640	7596	7707	7674	406	7631	2460	2460	7567	31211	3278
2190	7708	1839	1945	1875	7637	2023	7694	7694	1768	31017	7716
3804	7618	3625	3805	3815	7554	3668	7700	7700	3484	30969	7602
2091	7672	1745	1932	1865	7577	1913	7626	7626	1889	30986	7662
3735	7607	3574	3736	3728	7546	3589	7680	7680	3418	30927	7584
2201	7767	1745	326	109	7690	1903	7744	7744	1837	31009	7756
3783	7650	3661	3753	3791	7583	3662	7718	7718	3512	30941	7624
2301	7746	1869	492	247	7667	2027	7738	7738	1964	30994	7741
6827	7794	6802	6834	6810	7676	6768	7959	7959	6687	30790	7921
2192	7640	1805	1969	1877	7553	1912	7630	7630	1909	30923	7651
6861	7808	6822	6841	6817	7700	6807	7971	7971	6706	30811	7935
1909	7700	1514	1754	1732	7611	1920	7689	7689	1740	30936	7686
3693	7613	3542	3702	3687	7530	3570	7670	7670	3442	30912	7573
1923	7710	1430	1781	1757	7621	1953	7699	7699	1706	30939	7687
3658	7589	3499	3663	3683	7503	3532	7634	7634	3409	30910	7558
7574	3523	7541	7719	7677	3432	7651	3412	3412	7574	31137	261
7574	3523	7541	7719	7677	3432	7651	3412	3412	7574	31137	261
7575	3524	7542	7720	7678	3433	7652	3413	3413	7575	31138	262

7573	3522	7540	7718	7676	3431	7650	3411	3411	7573	31136	260
6847	7793	6808	6827	6803	7683	6793	7954	7954	6692	30797	7920
121	7707	2139	2244	2249	7618	2103	7682	7682	2033	30969	7699
2389	7769	1948	510	323	7643	2046	7745	7745	2028	31008	7740
2081	7700	1829	1994	1924	7622	1959	7657	7657	1815	31007	7688
7633	2558	7580	7681	7650	2380	7635	2517	2517	7584	31227	3302
2331	7760	1888	498	261	7680	2006	7729	7729	1968	31009	7767
2079	7706	1802	1893	1833	7620	1861	7662	7662	1764	31010	7691
1928	7701	1472	1825	1781	7617	1944	7700	7700	1743	30944	7690
6807	7804	6770	6807	6781	7694	6749	7968	7968	6658	30799	7930
3736	7615	3595	3758	3760	7547	3610	7672	7672	3441	30917	7588
7574	3524	7541	7719	7677	3433	7651	3413	3413	7574	31137	262
7573	3522	7540	7718	7676	3431	7650	3411	3411	7573	31136	260
7563	3493	7528	7642	7594	3393	7575	3375	3375	7508	31134	239
7575	3525	7542	7720	7678	3434	7652	3414	3414	7575	31138	263
7575	3525	7542	7720	7678	3434	7652	3414	3414	7575	31138	263
92	7731	2074	2229	2234	7639	2193	7708	7708	2174	30952	7716
2382	7762	1941	503	316	7636	2039	7738	7738	2021	31001	7733
2346	7807	1966	364	609	7718	2090	7791	7791	2038	31025	7805
2184	7708	1855	1961	1891	7637	2043	7695	7695	1786	31012	7715
2184	7695	1840	1927	1843	7621	2025	7681	7681	1772	30991	7700
1923	7698	1469	1820	1776	7612	1939	7697	7697	1738	30938	7689
1929	7702	1473	1826	1782	7617	1945	7701	7701	1744	30944	7692
5	7724	2109	2238	2243	7627	2156	7697	7697	2127	30946	7707
2116	7722	1866	1942	1930	7652	181	7691	7691	1751	30983	7703
2108	7714	1858	1934	1922	7644	173	7683	7683	1743	30975	7695
2297	7653	1965	2075	2012	7582	2132	7641	7641	1870	30998	7666
2328	7753	1883	503	266	7673	2003	7724	7724	1965	30998	7760
2125	7674	1616	1879	1876	7566	1807	7639	7639	25	30971	7636
2045	7695	1776	1881	1807	7610	1851	7654	7654	1736	31009	7686
1867	7680	1424	1743	1721	7590	1908	7665	7665	1709	30924	7661
3735	7605	3625	3756	3774	7521	3620	7660	7660	3502	30950	7597
6830	7793	6803	6835	6811	7675	6769	7958	7958	6688	30787	7920
3690	7611	3539	3700	3685	7527	3568	7668	7668	3439	30910	7570
2227	7792	1836	50	313	7713	1956	7770	7770	1872	31006	7793
7653	2660	7587	7715	7683	2655	7633	2599	2599	7588	31233	3377
3690	7611	3539	3700	3685	7527	3568	7668	7668	3439	30910	7570
2197	7787	1770	359	140	7710	1928	7761	7761	1865	31001	7780
3680	7573	3516	3671	3690	7501	3534	7643	7643	3365	30906	7556
2102	7748	1719	497	342	7677	1816	7732	7732	1831	31008	7745
2195	7747	1741	319	114	7672	1899	7740	7740	1834	31004	7735
2216	7789	1879	237	325	7708	1950	7780	7780	1857	31008	7782
2120	7671	1611	1874	1871	7563	1802	7636	7636	20	30968	7635
6950	7908	6924	6963	6941	7811	6873	8073	8073	6808	30813	8044
2379	7821	2020	361	614	7732	2110	7803	7803	2072	31030	7821
3705	7606	3541	3715	3721	7540	3553	7668	7668	3382	30912	7587
1591	7792	1864	2124	2105	7699	2063	7776	7776	2089	30955	7764
3795	7852	3551	3657	3667	7761	3561	7883	7883	3498	30966	7859
1898	7690	1503	1743	1721	7603	1909	7679	7679	1729	30922	7676
1886	7685	1889	1835	1861	7616	2034	7698	7698	1852	30961	7682
7654	2661	7588	7716	7684	2656	7634	2600	2600	7589	31234	3378
2251	7771	1808	463	280	7679	1971	7746	7746	1914	31023	7759
2203	7801	1814	87	339	7724	1930	7783	7783	1852	31016	7801
7611	2544	7583	7696	7662	510	7613	2365	2365	7559	31206	3241
3778	7623	3616	3774	3786	7555	3626	7677	7677	3467	30927	7605

3707	7632	3535	3714	3711	7547	3552	7680	7680	3378	30890	7609
2072	7715	1619	1820	1775	7633	1921	7681	7681	1772	31001	7703
2317	7744	1875	485	248	7664	1993	7713	7713	1955	30995	7751
7652	2659	7586	7714	7682	2654	7632	2598	2598	7587	31230	3376
3719	7589	3558	3746	3740	7526	3583	7637	7637	3404	30900	7576
3719	7589	3558	3746	3740	7526	3583	7637	7637	3404	30900	7576
7602	2535	7574	7687	7653	501	7604	2356	2356	7550	31198	3232
2107	7713	1857	1933	1921	7643	174	7682	7682	1742	30975	7694
2047	7697	1778	1885	1811	7612	1853	7656	7656	1740	31010	7688
3685	7646	3508	3642	3646	7584	3530	7707	7707	3388	30918	7626
3756	7586	3635	3779	3775	7506	3642	7650	7650	3519	30935	7549
1859	7672	1416	1735	1713	7582	1900	7657	7657	1701	30920	7653
2125	7676	1616	1879	1876	7568	1807	7641	7641	27	30973	7640
2213	7774	1801	95	352	7699	1941	7774	7774	1849	31011	7770
2010	7813	2008	2309	2363	7727	2324	7797	7797	2106	30966	7797
7704	3383	7664	7794	7756	3305	7674	3288	3288	7639	31162	17
2283	7770	1915	238	479	7680	2001	7747	7747	1934	30999	7772
7652	2659	7586	7714	7682	2654	7632	2598	2598	7587	31232	3376
7651	2658	7585	7713	7681	2653	7631	2597	2597	7586	31231	3375
3	7722	2107	2236	2241	7625	2154	7695	7695	2125	30944	7705
2099	7688	1846	1942	1860	7604	1895	7645	7645	1796	31006	7672
6818	7794	6782	6817	6793	7688	6763	7976	7976	6668	30807	7912
121	7707	2139	2244	2249	7618	2103	7682	7682	2033	30969	7699
121	7707	2139	2244	2249	7618	2103	7682	7682	2033	30969	7699
7653	2660	7587	7715	7683	2655	7633	2599	2599	7588	31233	3377
2190	7782	1763	350	131	7703	1921	7756	7756	1858	30996	7773
2201	7770	1746	326	109	7691	1903	7745	7745	1838	31009	7759
2120	7671	1611	1874	1871	7563	1802	7636	7636	20	30966	7635
3738	7613	3578	3738	3730	7552	3591	7682	7682	3422	30931	7588
3775	7627	3613	3771	3783	7560	3623	7681	7681	3463	30935	7609
7633	2559	7580	7681	7650	2384	7635	2517	2517	7584	31227	3304
1921	7696	1467	1818	1774	7610	1937	7695	7695	1736	30935	7687
1906	7690	1927	1849	1875	7621	2048	7706	7706	1884	30950	7683
2072	7682	1878	2001	1959	7599	1970	7642	7642	1816	31004	7674
2122	7728	1872	1948	1936	7656	189	7695	7695	1757	30986	7707
2110	7669	6	1844	1782	7602	1922	7665	7665	1619	30948	7668
2108	7667	4	1842	1780	7600	1920	7663	7663	1617	30946	7666
2139	7696	35	1873	1811	7629	1951	7692	7692	1648	30973	7695
6837	7824	6814	6860	6836	7706	6768	7987	7987	6704	30779	7959
3772	7588	3604	3752	3739	7522	3642	7668	7668	3503	30915	7556
2107	7714	1858	1934	1922	7644	175	7683	7683	1742	30974	7695
1908	7783	2057	2291	2353	7687	2218	7755	7755	2108	30947	7763
7603	2536	7575	7688	7654	502	7605	2357	2357	7551	31199	3233
7698	2620	7664	7780	7748	822	7698	2468	2468	7630	31219	3299
3703	7663	3570	3707	3710	7577	3572	7700	7700	3455	30913	7613
1924	7696	1512	1711	1687	7610	1888	7687	7687	1682	30937	7669
7698	2615	7665	7779	7746	2469	7677	28	28	7644	31239	3293
6	7725	2110	2239	2244	7628	2157	7698	7698	2128	30947	7708
12	7731	2116	2245	2250	7634	2163	7704	7704	2134	30951	7714
1884	7691	1455	1768	1746	7599	1931	7676	7676	1730	30937	7673
2223	7793	1842	122	261	7711	1946	7771	7771	1830	30994	7791
6875	7808	6846	6867	6843	7698	6831	7971	7971	6730	30804	7931
2119	7709	1855	1955	1873	7625	1906	7665	7665	1805	31020	7693
6803	7754	6776	6803	6779	7641	6745	7921	7921	6674	30787	7890
2240	7767	1778	317	14	7685	1940	7742	7742	1875	30991	7756

2237	7764	1775	314	11	7682	1937	7739	7739	1872	30988	7753
1998	7791	2013	2299	2337	7712	2265	7773	7773	2114	30967	7784
1909	7700	1514	1754	1732	7611	1920	7689	7689	1740	30936	7686
7631	2557	7578	7679	7648	2382	7633	2515	2515	7582	31225	3302
7632	2558	7579	7680	7649	2383	7634	2516	2516	7583	31226	3303
7695	2612	7662	7776	7743	2466	7674	25	25	7641	31237	3290
2195	7705	1852	1952	1882	7636	2033	7693	7693	1779	31012	7712

C7971	C7973	C7974	C7975	C7979	C8124	KCh007	HE-MDREc53	KFu023	KFu031	KMi011
7754	987	987	7577	7703	3753	3727	3761	1064	7509	6787
7777	3558	3558	7626	7772	1964	1829	1887	3633	7596	6712
7775	3612	3612	7620	7755	2007	2000	2105	3630	7588	6817
7964	3441	3441	7778	7931	3876	3893	3835	3177	7769	6993
7705	1078	1078	7528	7650	3704	3654	3671	450	7466	6740
25610	24938	24938	25525	25585	25019	25040	24971	24932	25510	25023
7812	3518	3518	7626	7778	1970	1827	2075	3606	7607	6725
7793	3474	3474	7625	7757	1952	1918	2216	3611	7624	6686
7798	3530	3530	7606	7765	2036	2000	2245	3669	7620	6798
25609	24937	24937	25524	25584	25018	25039	24970	24931	25509	25022
7866	3637	3637	7689	7840	247	309	2181	3783	7678	6814
7927	3639	3639	7771	7903	2393	2357	1865	3796	7754	6881
3473	7581	7581	2626	3443	7638	7675	7632	7537	3442	7749
7720	3480	3480	7551	7699	1931	1943	2181	3540	7549	6685
7775	3487	3487	7620	7751	2017	1972	2026	3596	7589	6794
8100	6972	6972	7883	8050	6986	6989	6966	6977	7998	434
7722	3470	3470	7564	7694	1820	1756	1898	3623	7551	6742
7872	3631	3631	7727	7851	2461	2320	1976	3739	7703	6812
7872	3631	3631	7727	7851	2461	2320	1976	3739	7703	6812
7857	3597	3597	7673	7825	1987	1958	2262	3706	7649	6831
7938	4186	4186	7825	7930	3666	3652	3807	4269	7776	6914
7791	3680	3680	7655	7779	2312	2229	5	3783	7621	6836
3495	7638	7638	2662	3489	7756	7795	7724	7582	3620	7799
7766	3510	3510	7589	7724	1878	1838	2109	3612	7600	6809
7911	3626	3626	7717	7883	496	52	2238	3764	7724	6841
7868	3636	3636	7685	7842	259	315	2243	3787	7680	6817
3478	7576	7576	2657	3419	7676	7716	7627	7531	3507	7681
7767	3532	3532	7635	7755	1994	1958	2156	3664	7624	6775
3320	7703	7703	2601	3338	7728	7773	7697	7651	3481	7964
3320	7703	7703	2601	3338	7728	7773	7697	7651	3481	7964
7745	3394	3394	7590	7705	1958	1874	2127	3437	7554	6694
31290	30908	30908	31233	31215	30996	31009	30945	30933	31149	30792
1905	7617	7617	3379	2015	7761	7796	7707	7569	775	7926
	7759	7759	3405	1924	7875	7909	7792	7706	2192	7998
7759			7580	7706	3669	3626	3681	1319	7529	6784
7759			7580	7706	3669	3626	3681	1319	7529	6784
3405	7580	7580		3359	7673	7713	7656	7549	3594	7778
1924	7706	7706	3359		7837	7883	7780	7655	2245	7937
7875	3669	3669	7673	7837		486	2313	3738	7690	6798
7909	3626	3626	7713	7883	486		2230	3761	7722	6833
7792	3681	3681	7656	7780	2313	2230		3784	7622	6837
7706	1319	1319	7549	7655	3738	3761	3784		7463	6800
2192	7529	7529	3594	2245	7690	7722	7622	7463		7871
7998	6784	6784	7778	7937	6798	6833	6837	6800	7871	
7987	6795	6795	7762	7923	6786	6813	6834	6805	7835	179
7719	1167	1167	7547	7661	3677	3640	3685	977	7480	6749
7873	3756	3756	7678	7835	395	507	2399	3870	7711	6839
8015	6825	6825	7791	7952	6816	6843	6864	6834	7864	209
7963	3723	3723	7786	7948	2221	2195	2451	3769	7802	6959
2249	7529	7529	3650	2296	7690	7722	7626	7461	98	7861
8013	6781	6781	7786	7947	6780	6813	6812	6795	7864	269
2066	7541	7541	3502	2146	7681	7718	7574	7481	814	7892
7797	3685	3685	7661	7785	2318	2234	11	3788	7627	6842
7807	3644	3644	7617	7753	2081	2054	2354	3698	7651	6798

7790	3413	3413	7614	7753	1966	1949	2212	3586	7611	6698
7731	3510	3510	7557	7720	1899	1915	2178	3579	7578	6721
7754	3557	3557	7616	7731	1861	1769	1924	3652	7594	6767
7777	3506	3506	7648	7767	1973	1935	2108	3637	7608	6746
217	7708	7708	3379	1917	7834	7874	7757	7653	2113	7953
3434	7560	7560	44	3388	7647	7687	7630	7527	3623	7802
7787	3536	3536	7599	7762	2018	1970	2137	3635	7596	6794
7793	3466	3466	7619	7754	1934	1941	2197	3620	7624	6668
7772	3438	3438	7594	7737	1942	1985	2245	3590	7597	6666
7798	3403	3403	7627	7753	1963	1940	2182	3598	7623	6664
7789	3536	3536	7601	7764	2018	1970	2137	3635	7598	6796
7996	6793	6793	7771	7934	6767	6800	6838	6826	7858	256
2013	7566	7566	3430	2108	7744	7767	7674	7526	874	7941
7788	3515	3515	7659	7778	1984	1946	2117	3646	7619	6755
7790	3677	3677	7656	7780	2309	2226	12	3780	7620	6839
2063	7540	7540	3499	2144	7680	7717	7573	7480	811	7891
2064	7541	7541	3500	2145	7681	7718	7574	7481	812	7892
7929	3629	3629	7763	7905	2390	2334	1918	3796	7757	6892
7777	3558	3558	7626	7772	1964	1829	1887	3633	7596	6712
7788	3514	3514	7659	7774	1961	1955	2136	3643	7619	6737
3443	7578	7578	2647	3391	7664	7706	7623	7541	3454	7696
7797	3688	3688	7657	7785	2322	2229	21	3791	7627	6848
7779	3661	3661	7630	7747	586	732	2458	3822	7577	6753
224	7715	7715	3387	1924	7839	7879	7762	7660	2122	7960
3320	7700	7700	2601	3335	7725	7770	7694	7648	3478	7959
7869	6801	6801	7669	7808	6817	6845	6850	6802	7744	292
7797	3444	3444	7617	7756	1965	1920	2195	3608	7622	6680
7787	3601	3601	7616	7767	1996	1992	2084	3648	7600	6812
1907	7620	7620	3381	2017	7764	7799	7710	7572	777	7929
2067	7544	7544	3503	2148	7684	7721	7577	7484	815	7895
1929	7626	7626	3369	2027	7772	7807	7714	7576	784	7932
1928	7625	7625	3368	2026	7771	7806	7713	7575	783	7931
1932	7629	7629	3372	2030	7775	7810	7717	7579	787	7935
1904	7616	7616	3378	2014	7761	7796	7707	7568	774	7926
7885	3634	3634	7728	7859	2453	2329	2057	3725	7705	6827
3474	7582	7582	2627	3444	7639	7676	7633	7538	3443	7750
7933	3706	3706	7717	7890	733	331	2366	3878	7738	6775
3399	7630	7630	2581	3308	7715	7757	7680	7568	3474	7730
7801	3689	3689	7663	7789	2302	2210	48	3764	7629	6846
7869	3645	3645	7695	7843	254	314	2192	3784	7681	6819
7876	3657	3657	7682	7844	153	387	2262	3810	7692	6828
7914	3686	3686	7744	7884	450	227	2223	3780	7722	6848
7789	3592	3592	7613	7769	1993	1987	2079	3646	7602	6805
7914	3676	3676	7746	7884	430	189	2230	3771	7726	6859
1932	7629	7629	3372	2030	7775	7810	7717	7579	787	7935
8015	6642	6642	7789	7949	6722	6751	6758	6660	7887	392
7989	6799	6799	7764	7925	6784	6811	6830	6811	7833	187
7859	3627	3627	7687	7835	247	313	2185	3794	7673	6814
7731	1184	1184	7534	7663	3727	3719	3707	676	7502	6755
7728	3493	3493	7561	7707	1944	1956	2194	3552	7559	6696
7759	548	548	7569	7686	3704	3666	3709	1092	7511	6781
1903	7615	7615	3377	2013	7759	7794	7705	7567	773	7925
7753	1082	1082	7554	7690	3784	3782	3751	803	7446	6813
8095	6967	6967	7878	8045	6981	6984	6961	6972	7993	429
7848	3653	3653	7674	7826	475	491	2103	3780	7660	6794

1914	7622	7622	3378	2018	7766	7801	7711	7574	784	7929
7909	3671	3671	7744	7882	425	184	2225	3766	7722	6853
7889	3595	3595	7704	7857	428	277	2253	3774	7687	6825
7693	1231	1231	7502	7646	3782	3763	3753	629	7491	6789
8085	6937	6937	7870	8029	6924	6943	6932	6932	7954	425
2066	7541	7541	3502	2147	7681	7718	7574	7481	814	7894
1935	7632	7632	3375	2033	7778	7813	7720	7582	790	7938
3447	7585	7585	2651	3393	7669	7711	7632	7548	3458	7704
7915	3603	3603	7716	7887	505	81	2223	3763	7728	6833
7811	3548	3548	7610	7786	1876	1800	2083	3614	7622	6790
217	7708	7708	3379	1917	7834	7874	7757	7653	2113	7953
7844	3663	3663	7716	7826	2234	2187	1678	3785	7674	6814
7911	3633	3633	7720	7883	439	129	2227	3767	7723	6840
7888	3669	3669	7696	7856	180	396	2271	3821	7704	6837
8012	6639	6639	7786	7946	6719	6748	6755	6657	7884	389
7719	1138	1138	7563	7664	3794	3757	3741	378	7499	6788
3401	7632	7632	2583	3310	7717	7759	7682	7570	3476	7732
7878	3659	3659	7684	7846	155	389	2264	3814	7694	6830
7790	3655	3655	7656	7776	2312	2240	78	3787	7612	6837
7789	3601	3601	7616	7769	1996	1992	2084	3648	7602	6812
7745	3548	3548	7607	7722	1852	1760	1915	3644	7585	6758
7909	3626	3626	7713	7883	486	2	2230	3761	7722	6833
7771	3501	3501	7653	7757	2012	1937	2155	3605	7602	6760
7772	3573	3573	7598	7752	2022	1994	2074	3627	7585	6820
7993	6801	6801	7768	7929	6792	6819	6840	6811	7839	185
3406	7636	7636	2588	3315	7721	7763	7686	7573	3481	7737
2066	7543	7543	3502	2147	7683	7720	7576	7483	814	7894
8013	6640	6640	7787	7947	6720	6749	6756	6658	7885	390
7980	6784	6784	7753	7916	6787	6814	6815	6792	7826	200
8014	6641	6641	7788	7948	6721	6750	6757	6659	7886	391
7688	1129	1129	7509	7620	3676	3673	3679	597	7460	6739
7948	3718	3718	7734	7905	751	347	2384	3894	7753	6792
7909	3652	3652	7726	7880	402	164	2200	3790	7721	6826
7834	3584	3584	7685	7812	2270	2169	1878	3702	7658	6821
7850	3693	3693	7709	7837	2409	2292	1920	3828	7680	6786
7795	1201	1201	7611	7737	3859	3840	3841	675	7507	6830
7795	1201	1201	7611	7737	3859	3840	3841	675	7507	6830
3440	7575	7575	2644	3388	7661	7703	7620	7538	3451	7693
7796	3476	3476	7622	7752	1956	1937	2191	3621	7621	6681
7742	1161	1161	7572	7688	3815	3799	3805	582	7520	6818
7763	3560	3560	7570	7735	1944	1928	2092	3611	7571	6789
7716	1147	1147	7550	7660	3723	3732	3736	553	7486	6776
7866	3645	3645	7694	7840	256	318	2202	3785	7678	6810
7785	547	547	7592	7720	3754	3745	3784	1453	7550	6790
7865	3713	3713	7678	7821	193	486	2302	3836	7696	6825
7993	6781	6781	7775	7932	6791	6826	6828	6797	7864	21
7733	3492	3492	7564	7712	1943	1955	2193	3550	7562	6697
8011	6829	6829	7786	7949	6808	6835	6862	6839	7869	217
7764	3531	3531	7605	7742	1812	1742	1910	3633	7596	6748
7727	628	628	7546	7662	3709	3696	3694	1056	7475	6750
7754	3557	3557	7616	7731	1861	1769	1924	3653	7594	6767
7698	975	975	7516	7633	3699	3651	3659	758	7474	6736
2065	7542	7542	3501	2146	7682	7719	7575	7482	813	7893
2065	7542	7542	3501	2146	7682	7719	7575	7482	813	7893
2066	7543	7543	3502	2147	7683	7720	7576	7483	814	7894

2064	7541	7541	3500	2145	7681	7718	7574	7481	812	7892
7994	6815	6815	7769	7932	6794	6821	6848	6825	7854	201
7783	3681	3681	7638	7771	2319	2236	122	3728	7614	6841
7864	3747	3747	7669	7826	386	498	2390	3861	7702	6830
7787	3599	3599	7614	7767	1994	1990	2082	3646	7600	6810
3473	7583	7583	2626	3441	7640	7677	7634	7539	3442	7751
7879	3680	3680	7680	7845	42	492	2332	3743	7698	6807
7783	3535	3535	7596	7764	1909	1889	2080	3613	7597	6769
7759	3480	3480	7626	7739	1885	1813	1929	3588	7598	6751
8004	6771	6771	7780	7938	6780	6801	6808	6782	7855	265
7728	1157	1157	7550	7670	3767	3760	3737	538	7499	6799
2066	7542	7542	3502	2146	7682	7719	7575	7482	814	7893
2064	7541	7541	3500	2145	7681	7718	7574	7481	812	7892
2066	7521	7521	3463	2128	7678	7642	7564	7490	720	7871
2067	7543	7543	3503	2147	7683	7720	7576	7483	815	7894
2067	7543	7543	3503	2147	7683	7720	7576	7483	815	7894
7807	3678	3678	7669	7791	2306	2221	93	3779	7633	6822
7857	3740	3740	7662	7819	379	491	2383	3854	7695	6823
7922	3727	3727	7719	7881	728	358	2347	3903	7727	6776
7792	3468	3468	7620	7754	1959	1947	2185	3624	7621	6676
7782	3449	3449	7605	7740	1922	1915	2185	3606	7608	6672
7756	3477	3477	7623	7736	1880	1808	1924	3585	7595	6746
7761	3481	3481	7627	7742	1886	1814	1930	3589	7598	6752
7792	3681	3681	7656	7780	2313	2230	6	3784	7622	6837
7786	3515	3515	7657	7776	1982	1944	2117	3646	7617	6755
7778	3507	3507	7649	7768	1974	1936	2109	3638	7609	6747
7748	3418	3418	7565	7713	2011	2063	2298	3562	7575	6684
7872	3685	3685	7675	7838	47	497	2329	3746	7691	6796
7742	3393	3393	7587	7702	1957	1873	2126	3436	7549	6693
7778	3543	3543	7590	7759	1885	1877	2046	3613	7592	6768
7731	3508	3508	7582	7707	1825	1731	1868	3612	7568	6748
7742	1069	1069	7528	7669	3759	3744	3736	795	7529	6752
7992	6778	6778	7774	7931	6792	6827	6831	6794	7865	10
7724	625	625	7543	7659	3707	3694	3691	1053	7472	6749
7906	3624	3624	7710	7880	484	16	2228	3759	7719	6830
3403	7578	7578	12	3357	7671	7711	7654	7547	3592	7776
7724	625	625	7543	7659	3707	3694	3691	1053	7472	6749
7886	3659	3659	7706	7860	267	353	2198	3819	7695	6834
7690	1131	1131	7511	7620	3676	3673	3681	599	7460	6743
7848	3653	3653	7674	7826	475	491	2103	3780	7660	6794
7841	3645	3645	7684	7815	252	315	2196	3760	7653	6813
7895	3673	3673	7727	7861	435	238	2217	3766	7681	6834
7739	3388	3388	7584	7699	1952	1868	2121	3431	7548	6688
8114	6917	6917	7893	8052	6929	6955	6951	6916	7893	347
7944	3718	3718	7724	7897	739	355	2380	3889	7735	6779
7731	1149	1149	7558	7673	3731	3709	3706	530	7517	6736
7844	3639	3639	7717	7826	2169	2120	1592	3806	7667	6814
7927	4175	4175	7812	7919	3655	3641	3796	4258	7765	6904
7754	3520	3520	7594	7732	1801	1731	1899	3622	7586	6737
7777	3565	3565	7623	7769	1956	1819	1887	3619	7594	6708
3404	7579	7579	13	3358	7672	7712	7655	7548	3593	7777
7869	3694	3694	7677	7842	419	451	2252	3819	7684	6808
7914	3646	3646	7727	7888	462	90	2204	3790	7727	6839
3378	7570	7570	2565	3334	7650	7692	7612	7519	3416	7732
7745	1172	1172	7561	7689	3788	3770	3779	516	7455	6804

7739	1148	1148	7556	7683	3699	3704	3708	616	7511	6748
7812	3544	3544	7608	7787	1878	1812	2073	3615	7623	6788
7863	3666	3666	7664	7829	23	479	2318	3730	7682	6791
3402	7577	7577	11	3356	7670	7710	7653	7546	3591	7775
7704	1153	1153	7515	7650	3732	3738	3720	576	7474	6751
7704	1153	1153	7515	7650	3732	3738	3720	576	7474	6751
3369	7561	7561	2556	3325	7641	7683	7603	7510	3407	7723
7777	3506	3506	7648	7767	1973	1935	2108	3637	7608	6746
7780	3543	3543	7592	7761	1887	1881	2048	3613	7594	6771
7768	62	62	7592	7713	3673	3636	3686	1326	7536	6790
7690	1115	1115	7512	7634	3771	3773	3757	734	7486	6795
7723	3500	3500	7574	7699	1817	1723	1860	3604	7560	6740
7744	3393	3393	7589	7704	1957	1873	2126	3436	7553	6693
7883	3652	3652	7715	7857	484	99	2214	3767	7696	6839
7885	3607	3607	7727	7864	2416	2295	2011	3724	7709	6820
1902	7615	7615	3376	2012	7759	7794	7705	7567	772	7924
7879	3713	3713	7690	7847	600	236	2284	3850	7683	6830
3402	7577	7577	11	3356	7670	7710	7653	7546	3591	7775
3401	7576	7576	10	3355	7669	7709	7652	7545	3590	7774
7790	3679	3679	7654	7778	2311	2228	4	3782	7620	6835
7776	3546	3546	7579	7749	1857	1936	2100	3642	7585	6753
7990	6780	6780	7784	7924	6791	6813	6819	6770	7841	276
7783	3681	3681	7638	7771	2319	2236	122	3728	7614	6841
7783	3681	3681	7638	7771	2319	2236	122	3728	7614	6841
3403	7578	7578	12	3357	7671	7711	7654	7547	3592	7776
7879	3652	3652	7699	7853	258	344	2191	3812	7688	6827
7869	3650	3650	7695	7841	258	318	2202	3788	7681	6811
7739	3388	3388	7584	7699	1952	1868	2121	3431	7548	6688
7722	1149	1149	7556	7664	3725	3734	3739	555	7490	6778
7749	1162	1162	7567	7693	3787	3767	3776	514	7461	6816
3475	7583	7583	2628	3445	7640	7677	7634	7539	3444	7751
7754	3475	3475	7621	7734	1878	1806	1922	3583	7593	6743
7780	3564	3564	7628	7775	1976	1835	1907	3636	7599	6708
7773	3572	3572	7597	7751	2021	1993	2073	3626	7584	6819
7790	3521	3521	7661	7780	1988	1950	2123	3652	7621	6761
7768	3512	3512	7591	7726	1880	1840	2111	3614	7602	6811
7766	3510	3510	7589	7724	1878	1838	2109	3612	7600	6809
7793	3541	3541	7618	7751	1909	1869	2140	3643	7629	6837
8025	6797	6797	7795	7961	6823	6852	6838	6816	7862	201
7702	692	692	7548	7644	3780	3744	3773	1136	7448	6783
7778	3507	3507	7649	7768	1974	1936	2108	3638	7609	6747
7831	3640	3640	7695	7815	2414	2281	1909	3756	7675	6811
3370	7562	7562	2557	3326	7642	7684	7604	7511	3408	7724
3417	7649	7649	2600	3327	7734	7776	7699	7587	3493	7749
7752	1014	1014	7577	7687	3696	3701	3704	776	7534	6788
7748	3525	3525	7592	7720	1781	1699	1925	3635	7584	6733
3322	7705	7705	2603	3340	7730	7775	7699	7653	3483	7966
7793	3682	3682	7657	7781	2314	2231	7	3785	7623	6838
7799	3688	3688	7663	7787	2320	2237	13	3791	7629	6844
7744	3531	3531	7589	7719	1850	1756	1885	3635	7580	6763
7902	3622	3622	7711	7874	436	118	2224	3758	7714	6831
8009	6871	6871	7788	7949	6834	6861	6876	6879	7871	257
7795	3562	3562	7600	7770	1873	1951	2120	3658	7601	6773
7966	6757	6757	7744	7899	6760	6795	6804	6793	7835	125
7866	3634	3634	7683	7840	257	313	2241	3785	7678	6815

7863	3631	3631	7680	7837	254	310	2238	3782	7675	6812
7866	3638	3638	7709	7842	2398	2287	1999	3750	7696	6806
7764	3531	3531	7605	7742	1812	1742	1910	3633	7596	6748
3473	7581	7581	2626	3443	7638	7675	7632	7537	3442	7749
3474	7582	7582	2627	3444	7639	7676	7633	7536	3443	7750
3319	7702	7702	2600	3337	7727	7772	7696	7650	3480	7961
7794	3465	3465	7618	7753	1933	1940	2196	3619	7625	6667

Kmi017	KMi024	KFu021	Kmi017	KMi019	KTa003	JSWP001	JSWP014	JSWP021	1657
6800	1209	3857	6829	3738	7507	6778	7531	3765	3684
6682	3541	2011	6712	2186	7598	6656	7578	1892	2078
6813	3543	2070	6843	2047	7598	6795	7539	2110	2065
7022	3448	3974	7051	3946	7769	7006	7767	3840	3808
6753	711	3780	6782	3680	7464	6733	7479	3675	3615
25015	24914	25044	25045	25174	25514	25040	25532	24974	24944
6722	3558	2017	6752	2179	7609	6698	7603	2079	2147
6684	3493	2027	6713	2147	7624	6670	7615	2221	2050
6794	3546	2101	6824	1935	7620	6772	7580	2250	1998
25014	24913	25043	25044	25173	25513	25039	25531	24973	24943
6792	3671	308	6822	2103	7678	6786	7678	2186	1974
6869	3677	2469	6899	2566	7758	6845	7698	1869	2489
7724	7544	7616	7753	7768	3502	7754	3425	7635	7573
6675	3433	1975	6705	811	7545	6659	7503	2186	569
6799	3525	2085	6829	2287	7593	6779	7549	2031	2118
391	6941	6898	421	7092	7992	435	8009	6971	6931
6724	3558	1883	6754	2185	7549	6702	7514	1903	2067
6798	3657	2526	6828	2537	7707	6774	7701	1981	2445
6798	3657	2526	6828	2537	7707	6774	7701	1981	2445
6836	3644	2067	6866	2391	7651	6816	7617	2266	2272
6920	4165	3756	6950	3755	7784	6958	7790	3812	3689
6833	3684	2398	6863	2450	7625	6811	7573	10	2353
7788	7611	7778	7817	7856	3672	7815	3524	7729	7702
6800	3533	1957	6830	2086	7600	6778	7540	2114	1964
6819	3650	519	6849	2199	7724	6821	7718	2242	2064
6795	3662	332	6825	2149	7680	6795	7676	2248	2008
7676	7522	7652	7705	7793	3536	7703	3433	7632	7620
6777	3544	2055	6807	2138	7620	6757	7650	2161	2158
7947	7648	7754	7976	7848	3524	7974	3413	7702	7673
7947	7648	7754	7976	7848	3524	7974	3413	7702	7673
6682	3432	2037	6712	2240	7550	6662	7573	2132	2150
30795	30914	31016	30825	31149	31159	30809	31136	30948	30933
7913	7587	7749	7942	7887	846	7941	262	7712	7734
7987	7719	7873	8015	7963	2249	8013	2066	7797	7807
6795	1167	3756	6825	3723	7529	6781	7541	3685	3644
6795	1167	3756	6825	3723	7529	6781	7541	3685	3644
7762	7547	7678	7791	7786	3650	7786	3502	7661	7617
7923	7661	7835	7952	7948	2296	7947	2146	7785	7753
6786	3677	395	6816	2221	7690	6780	7681	2318	2081
6813	3640	507	6843	2195	7722	6813	7718	2234	2054
6834	3685	2399	6864	2451	7626	6812	7574	11	2354
6805	977	3870	6834	3769	7461	6795	7481	3788	3698
7835	7480	7711	7864	7802	98	7864	814	7627	7651
179	6749	6839	209	6959	7861	269	7892	6842	6798
	6749	6739	30	6949	7825	98	7874	6839	6788
6749		3732	6779	3706	7478	6733	7501	3689	3628
6739	3732		6769	2287	7711	6727	7691	2404	2126
30	6779	6769		6979	7854	128	7903	6869	6818
6949	3706	2287	6979		7798	6931	7752	2456	777
7825	7478	7711	7854	7798		7850	879	7631	7647
98	6733	6727	128	6931	7850		7902	6817	6770
7874	7501	7691	7903	7752	879	7902		7579	7599
6839	3689	2404	6869	2456	7631	6817	7579		2359
6788	3628	2126	6818	777	7647	6770	7599	2359	

6700	3488	2018	6730	2136	7611	6680	7601	2217	2041
6711	3487	1969	6741	596	7574	6693	7528	2183	330
6768	3600	1928	6798	2176	7592	6746	7571	1929	2080
6727	3515	2051	6757	2094	7604	6707	7602	2113	2116
7940	7661	7826	7968	7927	2173	7967	2045	7762	7771
7786	7527	7652	7815	7760	3679	7766	3531	7635	7591
6782	3517	2075	6812	2041	7606	6766	7557	2142	2037
6666	3491	2022	6696	2153	7624	6648	7605	2202	2059
6653	3469	1977	6683	2163	7597	6635	7573	2250	2069
6662	3466	2029	6692	2174	7623	6644	7612	2187	2080
6784	3517	2075	6814	2041	7608	6768	7559	2142	2037
86	6745	6722	116	6954	7850	162	7885	6843	6788
7924	7535	7729	7953	7859	941	7949	384	7679	7705
6736	3522	2062	6766	2105	7615	6716	7613	2122	2127
6836	3681	2395	6866	2447	7624	6814	7574	17	2350
7873	7500	7690	7902	7751	876	7901	3	7578	7598
7874	7501	7691	7903	7752	877	7902	4	7579	7599
6880	3673	2462	6910	2595	7761	6856	7697	1922	2491
6682	3541	2011	6712	2186	7598	6656	7578	1892	2078
6712	3527	2061	6742	2108	7615	6692	7611	2141	2130
7691	7540	7644	7720	7781	3495	7721	3412	7628	7620
6845	3692	2392	6875	2458	7631	6823	7579	26	2349
6754	3715	633	6784	2358	7581	6791	7562	2463	2241
7947	7668	7831	7975	7932	2182	7974	2052	7767	7776
7942	7645	7751	7971	7845	3521	7969	3410	7699	7670
455	6774	6858	485	6989	7734	537	7765	6855	6829
6687	3487	2029	6717	2115	7622	6669	7601	2200	2021
6808	3531	2063	6838	2029	7610	6790	7553	2089	2043
7916	7590	7752	7945	7890	848	7944	264	7715	7737
7877	7504	7694	7906	7755	880	7905	7	7582	7602
7919	7594	7760	7948	7896	851	7948	286	7719	7743
7918	7593	7759	7947	7895	850	7947	285	7718	7742
7922	7597	7763	7951	7899	854	7951	289	7722	7746
7913	7586	7749	7942	7887	845	7941	259	7712	7734
6809	3656	2507	6839	2595	7709	6787	7703	2062	2475
7725	7545	7617	7754	7769	3503	7755	3426	7636	7574
6753	3728	778	6783	2341	7738	6751	7738	2370	2142
7725	7583	7693	7754	7838	3498	7749	3415	7685	7676
6843	3695	2379	6873	2446	7633	6821	7583	53	2358
6797	3675	317	6827	2103	7681	6793	7681	2197	1976
6806	3686	398	6836	2167	7692	6808	7690	2267	2035
6839	3698	535	6869	2222	7722	6841	7704	2228	2113
6801	3526	2060	6831	2032	7612	6785	7551	2084	2046
6837	3707	499	6867	2217	7726	6839	7727	2235	2108
7922	7597	7763	7951	7897	854	7951	289	7722	7744
221	6596	6675	251	6879	7889	217	7906	6763	6718
16	6755	6737	46	6953	7829	98	7874	6835	6792
6792	3651	328	6822	2108	7673	6790	7673	2190	1970
6769	702	3841	6798	3743	7500	6753	7515	3711	3670
6686	3446	1988	6716	824	7555	6670	7513	2199	582
6795	957	3792	6825	3770	7511	6783	7496	3713	3690
7912	7585	7747	7941	7885	844	7940	260	7710	7732
6811	625	3848	6840	3827	7444	6795	7521	3755	3749
386	6936	6893	416	7087	7987	430	8004	6966	6926
6772	3659	540	6802	2035	7662	6760	7661	2108	1970

7916	7592	7754	7945	7891	855	7944	269	7716	7739
6831	3702	494	6861	2212	7722	6833	7723	2230	2103
6808	3650	453	6838	2230	7687	6806	7679	2258	2123
6778	758	3797	6807	3764	7489	6764	7448	3757	3696
377	6892	6857	407	7059	7947	435	7967	6937	6900
7876	7501	7691	7905	7752	879	7904	6	7579	7599
7925	7600	7766	7954	7902	857	7954	292	7725	7749
7699	7547	7649	7728	7788	3499	7726	3416	7637	7627
6811	3649	538	6841	2216	7728	6813	7724	2227	2075
6773	3558	1929	6803	2002	7636	6753	7566	2088	1980
7940	7661	7826	7968	7927	2173	7967	2045	7762	7771
6808	3732	2300	6838	2355	7674	6788	7655	1683	2330
6818	3657	462	6848	2228	7723	6816	7722	2231	2087
6815	3698	407	6845	2176	7704	6817	7702	2276	2044
218	6593	6672	248	6876	7886	214	7903	6760	6715
6784	780	3833	6813	3774	7497	6770	7491	3745	3710
7727	7585	7695	7756	7840	3500	7751	3417	7687	7678
6808	3688	400	6838	2169	7694	6810	7692	2269	2037
6832	3681	2383	6862	2399	7616	6810	7570	83	2302
6808	3531	2063	6838	2029	7612	6790	7555	2089	2043
6759	3591	1919	6789	2167	7583	6737	7562	1920	2071
6813	3640	507	6843	2195	7722	6813	7718	2234	2054
6741	3507	2058	6771	2132	7598	6723	7592	2160	2150
6818	3509	2091	6848	2049	7595	6802	7540	2079	2075
14	6755	6745	44	6955	7829	104	7878	6845	6794
7732	7589	7699	7761	7844	3505	7756	3422	7691	7682
7876	7503	7693	7905	7754	879	7904	6	7581	7601
219	6594	6673	249	6877	7887	215	7904	6761	6716
29	6740	6740	59	6932	7820	91	7867	6820	6771
220	6595	6674	250	6878	7888	216	7905	6762	6717
6759	722	3823	6788	3700	7458	6745	7468	3683	3628
6770	3740	796	6800	2359	7753	6768	7753	2388	2158
6804	3686	459	6834	2193	7721	6802	7718	2205	2086
6807	3610	2323	6837	2397	7658	6783	7657	1883	2271
6772	3750	2466	6802	2587	7686	6746	7670	1925	2435
6814	838	3929	6843	3874	7505	6796	7580	3845	3803
6814	838	3929	6843	3874	7505	6796	7580	3845	3803
7688	7537	7641	7717	7778	3492	7718	3409	7625	7617
6679	3503	2026	6709	2137	7621	6659	7614	2196	2043
6818	859	3874	6847	3819	7518	6790	7512	3809	3752
6779	3489	2017	6809	2086	7581	6761	7529	2097	2084
6796	734	3878	6825	3737	7484	6776	7496	3740	3667
6788	3671	323	6818	2109	7678	6788	7674	2207	1986
6786	1209	3864	6816	3843	7548	6780	7536	3788	3758
6728	3716	378	6758	2265	7696	6728	7677	2307	2130
184	6748	6832	214	6952	7860	266	7887	6833	6791
6687	3443	1987	6717	823	7558	6671	7516	2198	581
46	6783	6761	76	6971	7861	128	7896	6867	6810
6736	3564	1885	6766	2214	7594	6714	7558	1915	2079
6755	944	3816	6784	3768	7473	6737	7499	3698	3693
6768	3600	1928	6798	2176	7592	6746	7571	1929	2080
6752	628	3788	6781	3721	7472	6734	7488	3663	3643
7875	7502	7692	7904	7753	878	7903	5	7580	7600
7875	7502	7692	7904	7753	878	7903	5	7580	7600
7876	7503	7693	7905	7754	879	7904	4	7581	7601

7874	7501	7691	7903	7752	877	7902	4	7579	7599
30	6769	6747	60	6957	7846	112	7881	6853	6796
6838	3661	2405	6868	2473	7618	6816	7566	127	2374
6730	3723	19	6760	2278	7702	6718	7682	2395	2117
6806	3529	2061	6836	2027	7610	6788	7553	2087	2041
7726	7546	7618	7755	7768	3502	7756	3425	7637	7575
6795	3682	411	6825	2229	7698	6797	7687	2337	2098
6757	3511	1986	6787	2015	7607	6741	7560	2085	2015
6737	3533	1952	6767	2208	7596	6715	7558	1934	2065
94	6719	6733	124	6923	7841	22	7891	6813	6758
6807	745	3884	6836	3752	7497	6793	7480	3741	3682
7875	7502	7692	7904	7753	879	7903	2	7580	7600
7874	7501	7691	7903	7752	877	7902	4	7579	7599
7866	7505	7705	7895	7789	785	7894	323	7569	7636
7876	7503	7693	7905	7754	880	7904	3	7581	7601
7876	7503	7693	7905	7754	880	7904	3	7581	7601
6819	3710	2374	6849	2492	7637	6787	7583	98	2401
6723	3716	22	6753	2271	7695	6711	7675	2388	2110
6754	3749	777	6784	2336	7727	6752	7727	2352	2117
6674	3497	2034	6703	2147	7621	6656	7613	2190	2053
6670	3480	2004	6700	2131	7608	6652	7598	2190	2039
6732	3530	1947	6762	2203	7593	6710	7555	1929	2060
6738	3534	1953	6768	2209	7596	6716	7558	1935	2066
6834	3685	2399	6864	2451	7626	6812	7574	11	2354
6736	3522	2060	6766	2103	7613	6716	7611	2122	2125
6728	3514	2052	6758	2095	7605	6708	7603	2114	2117
6675	3434	2073	6705	2222	7575	6659	7532	2303	2121
6784	3687	416	6814	2226	7691	6786	7680	2334	2095
6681	3431	2036	6711	2239	7545	6661	7568	2131	2149
6756	3511	1962	6786	2002	7602	6740	7555	2051	2006
6734	3563	1892	6764	2148	7566	6712	7527	1873	2030
6753	564	3809	6782	3788	7527	6735	7498	3740	3705
173	6743	6833	203	6953	7855	263	7886	6836	6792
6754	942	3814	6783	3766	7470	6736	7496	3695	3691
6810	3638	505	6840	2193	7719	6810	7715	2232	2052
7760	7545	7676	7789	7784	3648	7784	3500	7659	7615
6754	942	3814	6783	3766	7470	6736	7496	3695	3691
6812	3693	350	6841	2134	7695	6810	7698	2203	2005
6763	724	3823	6792	3698	7458	6749	7468	3685	3626
6772	3659	540	6802	2035	7662	6760	7661	2108	1970
6791	3676	314	6821	2106	7653	6789	7653	2201	1977
6815	3682	498	6845	2226	7681	6817	7692	2222	2113
6676	3426	2031	6706	2234	7544	6656	7567	2126	2144
363	6861	6951	393	7084	7883	453	8006	6956	6923
6760	3727	794	6790	2356	7735	6758	7741	2384	2157
6742	779	3830	6771	3731	7515	6728	7512	3710	3668
6808	3722	2233	6838	2233	7667	6786	7646	1597	2208
6910	4154	3745	6940	3744	7773	6948	7779	3801	3678
6725	3553	1874	6755	2203	7584	6703	7548	1904	2068
6678	3550	2001	6708	2170	7596	6652	7576	1892	2065
7761	7546	7677	7790	7785	3649	7785	3501	7660	7616
6782	3698	364	6812	2174	7684	6757	7685	2257	2013
6817	3678	529	6847	2173	7727	6819	7723	2209	2058
7727	7528	7628	7756	7763	3457	7754	3372	7617	7598
6790	793	3859	6819	3789	7453	6772	7532	3783	3717

6768	739	3849	6797	3708	7509	6748	7521	3712	3632
6771	3564	1925	6801	2010	7637	6751	7567	2078	1992
6779	3668	398	6809	2214	7682	6781	7671	2323	2082
7759	7544	7675	7788	7783	3647	7783	3499	7658	7614
6770	741	3885	6799	3722	7472	6750	7488	3724	3654
6770	741	3885	6799	3722	7472	6750	7488	3724	3654
7718	7519	7619	7747	7754	3448	7745	3363	7608	7589
6727	3513	2051	6757	2094	7604	6707	7602	2113	2116
6759	3511	1964	6789	2006	7604	6743	7557	2053	2010
6801	1179	3764	6831	3723	7536	6783	7550	3690	3648
6782	681	3792	6811	3815	7484	6762	7441	3761	3747
6726	3555	1884	6756	2140	7558	6704	7519	1865	2022
6681	3431	2036	6711	2239	7549	6661	7572	2131	2149
6817	3683	523	6847	2190	7696	6819	7692	2219	2049
6806	3638	2475	6836	2538	7713	6786	7707	2016	2427
7911	7585	7747	7940	7885	843	7939	259	7710	7732
6807	3736	668	6837	2271	7690	6834	7690	2289	2156
7759	7544	7675	7788	7783	3647	7783	3499	7658	7614
7758	7543	7674	7787	7782	3646	7782	3498	7657	7613
6832	3683	2397	6862	2449	7624	6810	7572	9	2352
6744	3494	1959	6774	2076	7595	6726	7529	2105	2072
105	6731	6744	135	6939	7827	37	7873	6824	6774
6838	3661	2405	6868	2473	7618	6816	7566	127	2374
6838	3661	2405	6868	2473	7618	6816	7566	127	2374
7760	7545	7676	7789	7784	3648	7784	3500	7659	7615
6805	3686	341	6834	2127	7688	6803	7691	2196	1998
6789	3676	323	6819	2110	7681	6789	7677	2207	1987
6676	3426	2031	6706	2234	7544	6656	7567	2126	2144
6798	736	3880	6827	3740	7488	6778	7499	3743	3670
6802	783	3856	6831	3789	7459	6784	7536	3780	3718
7726	7546	7618	7755	7770	3504	7756	3427	7637	7575
6729	3528	1945	6759	2201	7591	6707	7553	1927	2058
6678	3549	2009	6708	2194	7601	6654	7577	1912	2094
6817	3508	2090	6847	2048	7594	6801	7539	2078	2074
6742	3528	2066	6772	2109	7617	6722	7615	2128	2131
6802	3535	1959	6832	2088	7602	6780	7542	2116	1966
6800	3533	1957	6830	2086	7600	6778	7540	2114	1964
6828	3564	1988	6858	2117	7629	6806	7569	2145	1995
225	6771	6846	255	6954	7854	283	7903	6843	6795
6779	1007	3832	6808	3832	7446	6763	7478	3777	3763
6728	3514	2052	6758	2095	7605	6708	7603	2113	2117
6797	3678	2465	6827	2428	7679	6773	7673	1914	2397
7719	7520	7620	7748	7755	3449	7746	3364	7609	7590
7744	7602	7712	7773	7857	3517	7768	3434	7704	7695
6784	694	3816	6813	3784	7532	6764	7533	3708	3712
6722	3562	1852	6752	2196	7582	6700	7567	1930	2078
7949	7650	7756	7978	7850	3526	7976	3415	7704	7675
6835	3686	2400	6865	2452	7627	6813	7575	12	2355
6841	3692	2406	6871	2458	7633	6819	7581	18	2361
6749	3585	1915	6779	2183	7578	6727	7539	1890	2065
6809	3646	451	6839	2221	7714	6807	7711	2228	2082
86	6825	6787	116	6999	7871	154	7898	6881	6838
6764	3506	1984	6794	2086	7611	6748	7550	2125	2088
286	6729	6801	316	6926	7829	372	7858	6809	6767
6793	3660	330	6823	2147	7678	6793	7674	2246	2006

6790	3657	327	6820	2144	7675	6790	7671	2243	2003
6792	3660	2451	6822	2494	7696	6768	7694	2004	2394
6736	3564	1885	6766	2214	7594	6714	7558	1915	2079
7724	7544	7616	7753	7768	3502	7754	3425	7635	7573
7725	7543	7617	7754	7769	3503	7755	3426	7636	7574
7944	7647	7753	7973	7847	3523	7971	3412	7701	7672
6665	3490	2021	6695	2152	7625	6647	7606	2201	2058

5051	VREC0418	VREC0426	VREC0506	VREC0559	C-0863N0015	AMA940	703	998
3499	3544	3658	3523	7701	7557	3563	3556	3536
1965	1919	1783	1949	7745	7600	1957	1943	1985
1967	1903	1772	1939	7732	7594	353	1940	1980
3809	3714	3791	3788	7925	7756	3761	3836	3771
3467	3487	3542	3486	7648	7508	3513	3461	3474
24980	24961	25016	25032	25595	25545	24978	24988	24960
1791	1988	1533	1922	7770	7600	1776	1769	1800
195	1875	1820	1945	7757	7603	1911	149	253
1816	1778	1919	1964	7755	7580	1947	1781	1850
24979	24960	25015	25031	25594	25544	24977	24987	24959
1853	1787	1708	1871	7829	7663	1889	1841	1885
2425	2397	2173	2235	7891	7745	2216	2419	2445
7609	7527	7613	7634	3464	2652	7582	7614	7567
1805	378	1907	1858	7686	7525	1819	1809	1819
2049	1981	1861	1859	7744	7594	1907	2037	2090
6851	6854	6901	6905	8062	7907	6919	6850	6817
1780	1922	239	1856	7680	7540	1823	1792	1828
2382	2299	2175	2227	7835	7707	2076	2375	2389
2382	2299	2175	2227	7835	7707	2076	2375	2389
1988	2103	1747	1955	7815	7651	1856	1976	1986
3503	3529	3543	3548	7894	7853	3353	3489	3478
2211	2177	1923	2107	7756	7629	2136	2196	2244
7702	7636	7710	7713	3478	2691	7701	7706	7678
1867	1807	1430	1857	7733	7563	1827	1853	1906
1963	1929	1781	1933	7876	7691	1976	1953	1997
1897	1835	1757	1921	7831	7659	1932	1883	1929
7627	7551	7621	7643	3454	2686	7611	7637	7586
2051	1963	1953	174	7727	7609	1926	2034	2062
7685	7629	7699	7682	3316	2629	7652	7694	7665
7685	7629	7699	7682	3316	2629	7652	7694	7665
1794	2009	1706	1742	7706	7566	1819	1780	1824
31008	30936	30936	30977	31273	31248	31001	31013	30999
7714	7663	7687	7694	1882	3408	7688	7711	7687
7790	7731	7754	7777	217	3434	7787	7793	7772
3413	3510	3557	3506	7708	7560	3536	3466	3438
3413	3510	3557	3506	7708	7560	3536	3466	3438
7614	7557	7616	7648	3379	44	7599	7619	7594
7753	7720	7731	7767	1917	3388	7762	7754	7737
1966	1899	1861	1973	7834	7647	2018	1934	1942
1949	1915	1769	1935	7874	7687	1970	1941	1985
2212	2178	1924	2108	7757	7630	2137	2197	2245
3586	3579	3652	3637	7653	7527	3635	3620	3590
7611	7578	7594	7608	2113	3623	7596	7624	7597
6698	6721	6767	6746	7953	7802	6794	6668	6666
6700	6711	6768	6727	7940	7786	6782	6666	6653
3488	3487	3600	3515	7661	7527	3517	3491	3469
2018	1969	1928	2051	7826	7652	2075	2022	1977
6730	6741	6798	6757	7968	7815	6812	6696	6683
2136	596	2176	2094	7927	7760	2041	2153	2163
7611	7574	7592	7604	2173	3679	7606	7624	7597
6680	6693	6746	6707	7967	7766	6766	6648	6635
7601	7528	7571	7602	2045	3531	7557	7605	7573
2217	2183	1929	2113	7762	7635	2142	2202	2250
2041	330	2080	2116	7771	7591	2037	2059	2069

	1858	1821	1946	7754	7592	1912	178	198
1858		1931	1921	7695	7531	1865	1868	1878
1821	1931		1887	7716	7592	1797	1809	1863
1946	1921	1887		7737	7622	1878	1930	1954
7754	7695	7716	7737		3408	7748	7751	7730
7592	7531	7592	7622	3408		7573	7597	7572
1912	1865	1797	1878	7748	7573		1892	1928
178	1868	1809	1930	7751	7597	1892		206
198	1878	1863	1954	7730	7572	1928	206	
241	1901	1790	1955	7758	7605	1907	165	283
1912	1865	1797	1878	7750	7575	20	1892	1928
6696	6714	6779	6730	7951	7795	6787	6664	6651
7682	7634	7654	7667	1995	3458	7651	7679	7663
1957	1932	1896	35	7748	7633	1887	1941	1965
2208	2176	1920	2104	7755	7630	2135	2193	2241
7600	7527	7570	7601	2042	3528	7556	7604	7572
7601	7528	7571	7602	2043	3529	7557	7605	7573
2447	2399	2194	2244	7893	7737	2251	2441	2467
1965	1919	1783	1949	7745	7600	1957	1943	1985
1960	1935	1907	48	7746	7633	1904	1928	1962
7623	7543	7622	7641	3417	2675	7612	7631	7582
2221	2185	1913	2121	7762	7631	2152	2206	2254
2055	2061	1992	2105	7746	7654	2133	2078	2058
7759	7700	7721	7742	15	3416	7753	7756	7735
7682	7626	7696	7679	3316	2629	7649	7691	7662
6717	6752	6778	6772	7824	7693	6801	6684	6682
178	1830	1795	1927	7755	7595	1885	140	206
1945	1878	1749	1913	7744	7590	334	1925	1965
7717	7666	7690	7697	1884	3410	7691	7714	7690
7604	7531	7574	7605	2046	3532	7560	7608	7576
7722	7672	7696	7703	1911	3397	7697	7719	7695
7721	7671	7695	7702	1910	3396	7696	7718	7694
7725	7675	7699	7706	1914	3400	7700	7722	7698
7714	7663	7687	7694	1881	3407	7688	7711	7687
2383	2333	2140	2292	7847	7708	2124	2366	2376
7610	7528	7614	7635	3465	2653	7583	7615	7568
2142	2057	1956	2082	7900	7691	2127	2138	2182
7669	7599	7658	7686	3383	2610	7659	7677	7630
2214	2187	1926	2100	7766	7637	2162	2199	2247
1855	1793	1709	1874	7832	7669	1895	1853	1897
1917	1855	1776	1943	7843	7656	1956	1911	1955
1936	1956	1756	1914	7872	7718	1977	1914	1962
1948	1877	1754	1916	7746	7587	327	1912	1952
1951	1951	1767	1933	7876	7720	1974	1943	1987
7725	7673	7699	7706	1914	3400	7700	7722	7698
6626	6641	6694	6659	7968	7769	6704	6592	6579
6704	6715	6770	6729	7942	7788	6786	6670	6657
1853	1802	1726	1891	7822	7661	1888	1853	1897
3531	3532	3600	3540	7674	7514	3565	3530	3527
1818	391	1920	1871	7694	7535	1830	1822	1832
3455	3545	3611	3563	7690	7549	3524	3498	3464
7712	7661	7685	7692	1880	3406	7686	7709	7685
3595	3606	3676	3617	7694	7534	3641	3618	3585
6846	6849	6896	6900	8057	7902	6914	6845	6812
1809	1747	1654	1795	7811	7648	1812	1799	1843

7718	7668	7691	7700	1891	3407	7693	7715	7691
1946	1946	1762	1928	7871	7718	1969	1938	1982
1925	1960	1843	1935	7851	7678	2003	1955	1923
3575	3560	3654	3585	7641	7482	3606	3586	3556
6805	6823	6866	6863	8043	7894	6886	6798	6761
7601	7528	7571	7602	2045	3531	7557	7605	7573
7728	7678	7702	7709	1917	3403	7703	7725	7701
7628	7550	7627	7646	3421	2680	7619	7636	7587
1964	1924	1778	1942	7880	7690	1975	1948	1992
1866	1829	1685	1876	7777	7584	607	1854	1910
7754	7695	7716	7737	2	3408	7748	7751	7730
2280	2145	2055	2065	7804	7690	2128	2271	2283
1950	1950	1760	1932	7873	7694	1973	1950	1994
1926	1864	1785	1952	7855	7670	1968	1920	1964
6623	6638	6691	6656	7965	7766	6701	6589	6576
3572	3575	3635	3592	7664	7543	3614	3574	3558
7671	7601	7660	7688	3385	2612	7661	7679	7632
1919	1857	1778	1945	7845	7658	1958	1913	1957
2214	2126	1938	2080	7755	7630	2147	2209	2215
1945	1878	1749	1913	7746	7590	334	1925	1965
1812	1922	21	1878	7707	7583	1788	1800	1854
1949	1915	1769	1935	7874	7687	1970	1941	1985
1990	1964	1928	76	7731	7627	1913	1961	1985
1971	1906	1767	1925	7729	7572	398	1945	1985
6706	6717	6774	6733	7946	7792	6788	6672	6659
7675	7605	7664	7692	3390	2617	7665	7683	7636
7603	7530	7573	7604	2045	3531	7559	7607	7575
6624	6639	6692	6657	7966	7767	6702	6590	6577
6683	6694	6751	6710	7933	7777	6769	6649	6636
6625	6640	6693	6658	7967	7768	6703	6591	6578
3485	3487	3563	3504	7631	7489	3525	3471	3503
2160	2073	1972	2098	7915	7708	2143	2156	2200
1921	1923	1739	1905	7871	7700	1948	1909	1953
2242	2131	1990	2190	7796	7665	1933	2243	2257
2370	2296	2058	2195	7812	7683	2175	2363	2377
3646	3677	3757	3663	7736	7591	3710	3641	3651
3646	3677	3757	3663	7736	7591	3710	3641	3651
7620	7540	7619	7638	3414	2672	7609	7628	7579
162	1854	1793	1916	7756	7600	1884	118	224
3604	3621	3676	3645	7685	7552	3667	3616	3607
1971	1913	1743	1867	7720	7544	465	1949	1998
3540	3526	3616	3557	7659	7530	3583	3528	3560
1861	1803	1716	1882	7829	7668	1897	1849	1893
3613	3640	3684	3642	7730	7572	3657	3588	3565
1978	1941	1858	2008	7822	7652	1998	1933	2000
6691	6714	6760	6739	7948	7799	6787	6661	6659
1817	390	1919	1870	7699	7538	1831	1821	1831
6722	6733	6798	6757	7966	7810	6804	6688	6675
1881	1934	482	1869	7722	7581	1849	1867	1911
3502	3536	3612	3541	7672	7526	3552	3481	3491
1821	1931	34	1887	7716	7592	1797	1809	1863
3478	3515	3553	3506	7641	7496	3538	3519	3509
7602	7529	7572	7603	2044	3530	7558	7606	7574
7602	7529	7572	7603	2044	3530	7558	7606	7574
7603	7530	7573	7604	2045	3531	7559	7607	7575

7601	7528	7571	7602	2043	3529	7557	7605	7573
6708	6719	6784	6743	7949	7793	6790	6674	6661
2232	2214	1932	2055	7748	7612	2137	2217	2265
2009	1960	1919	2042	7817	7643	2066	2013	1968
1943	1876	1747	1911	7744	7588	332	1923	1963
7611	7529	7615	7636	3464	2652	7584	7616	7569
1980	1921	1873	1985	7842	7654	2032	1946	1954
1888	1824	1725	1811	7744	7570	387	1876	1912
1857	1937	373	1885	7720	7602	1847	1845	1899
6674	6687	6744	6699	7958	7760	6758	6640	6627
3537	3541	3635	3577	7671	7530	3601	3571	3545
7602	7529	7572	7603	2045	3531	7558	7606	7574
7601	7528	7571	7602	2043	3529	7557	7605	7573
7588	7565	7541	7593	2043	3492	7571	7575	7602
7603	7530	7573	7604	2046	3532	7559	7607	7575
7603	7530	7573	7604	2046	3532	7559	7607	7575
2233	2229	1989	2145	7773	7643	2204	2218	2266
2002	1953	1912	2035	7810	7636	2059	2006	1961
2135	2044	1947	2067	7889	7693	2088	2131	2175
180	1859	1811	1936	7752	7598	1889	130	234
164	1846	1795	1918	7742	7583	1865	104	208
1852	1932	368	1880	7717	7599	1844	1840	1894
1858	1938	374	1886	7722	7603	1848	1846	1900
2212	2178	1924	2108	7757	7630	2137	2197	2245
1955	1930	1896	33	7746	7631	1885	1939	1963
1947	1922	1888	25	7738	7623	1877	1931	1955
437	1915	1923	2028	7708	7543	1992	423	407
1975	1918	1870	1982	7835	7649	2029	1941	1949
1793	2008	1705	1741	7703	7563	1818	1779	1823
1853	1811	1707	1801	7739	7564	423	1841	1877
1813	1885	188	1849	7693	7558	1787	1801	1855
3584	3580	3664	3590	7685	7508	3621	3605	3558
6692	6714	6761	6740	7947	7798	6788	6662	6660
3500	3534	3609	3539	7669	7523	3549	3479	3489
1947	1913	1767	1933	7871	7684	1968	1939	1983
7612	7555	7614	7646	3377	42	7597	7617	7592
3500	3534	3609	3539	7669	7523	3549	3479	3489
1881	1809	1744	1907	7849	7680	1898	1871	1915
3485	3485	3563	3502	7633	7491	3525	3471	3503
1809	1747	1654	1795	7811	7648	1812	1799	1843
1856	1797	1712	1878	7804	7658	1899	1857	1901
1934	1954	1778	1923	7853	7701	1967	1920	1962
1788	2003	1700	1736	7700	7560	1813	1774	1818
6813	6846	6885	6875	8072	7917	6909	6820	6796
2156	2072	1972	2091	7907	7698	2147	2142	2190
3526	3538	3593	3527	7674	7538	3560	3485	3536
2236	2023	2012	2028	7804	7691	2028	2233	2241
3492	3518	3532	3537	7883	7840	3342	3478	3467
1870	1923	471	1858	7712	7570	1838	1856	1900
1966	1902	1767	1955	7745	7597	1973	1944	1986
7613	7556	7615	7647	3378	43	7598	7618	7593
1962	1856	1765	1950	7832	7651	1916	1940	1972
1935	1893	1753	1907	7879	7701	1946	1917	1961
7607	7521	7606	7620	3352	2594	7591	7615	7566
3581	3591	3667	3598	7686	7541	3637	3576	3586

3502	3495	3575	3520	7682	7536	3581	3497	3529
1866	1823	1679	1870	7778	7582	603	1844	1900
1967	1902	1860	1972	7826	7638	2013	1933	1941
7611	7554	7613	7645	3376	41	7596	7616	7591
3538	3507	3599	3551	7647	7495	3586	3525	3557
3538	3507	3599	3551	7647	7495	3586	3525	3557
7598	7512	7597	7611	3343	2585	7582	7606	7557
1946	1921	1887	26	7737	7622	1876	1930	1954
1857	1815	1709	1803	7741	7566	425	1845	1881
3411	3514	3551	3504	7717	7572	3542	3478	3450
3603	3599	3669	3610	7634	7492	3630	3611	3581
1805	1877	180	1841	7685	7550	1779	1793	1847
1793	2008	1705	1741	7705	7565	1818	1779	1823
1946	1907	1765	1918	7848	7689	1936	1935	1979
2338	2282	2142	2261	7847	7707	2061	2321	2335
7712	7661	7685	7692	1879	3405	7686	7709	7685
2016	1995	1815	1978	7847	7714	2037	2016	2060
7611	7554	7613	7645	3376	41	7596	7616	7591
7610	7553	7612	7644	3375	40	7595	7615	7590
2210	2176	1922	2106	7755	7628	2135	2195	2243
1871	1876	1791	1854	7733	7553	515	1835	1886
6688	6698	6753	6713	7944	7764	6769	6653	6640
2232	2214	1932	2055	7748	7612	2137	2217	2265
2232	2214	1932	2055	7748	7612	2137	2217	2265
7612	7555	7614	7646	3377	42	7597	7617	7592
1872	1802	1735	1900	7842	7673	1891	1862	1906
1862	1804	1717	1882	7832	7669	1898	1850	1894
1788	2003	1700	1736	7700	7560	1813	1774	1818
3542	3529	3620	3559	7665	7536	3586	3530	3562
3571	3596	3664	3595	7690	7547	3637	3565	3576
7611	7529	7615	7636	3466	2654	7584	7616	7569
1850	1930	366	1878	7715	7597	1842	1838	1892
1981	1937	1799	1969	7748	7602	1967	1951	1993
1970	1905	1766	1924	7730	7571	397	1944	1984
1961	1936	1902	41	7750	7635	1891	1945	1969
1869	1809	1432	1859	7735	7565	1829	1855	1908
1867	1807	1430	1857	7733	7563	1827	1853	1906
1898	1838	1461	1888	7760	7592	1858	1884	1937
6701	6718	6777	6753	7978	7819	6807	6686	6648
3561	3621	3658	3614	7647	7528	3645	3581	3562
1947	1922	1885	27	7738	7623	1877	1931	1955
2371	2254	2112	2155	7793	7675	2094	2364	2378
7599	7513	7598	7612	3344	2586	7583	7607	7558
7688	7618	7677	7705	3401	2629	7678	7696	7649
3540	3561	3624	3555	7693	7557	3576	3545	3556
1835	1933	337	1862	7706	7568	1843	1815	1869
7687	7631	7701	7684	3318	2631	7654	7696	7667
2213	2179	1925	2109	7758	7631	2138	2198	2246
2219	2185	1931	2115	7764	7637	2144	2204	2252
1844	1920	225	1872	7706	7565	1808	1832	1886
1945	1945	1755	1925	7864	7685	1964	1935	1979
6742	6761	6816	6781	7966	7812	6816	6708	6695
1886	1886	1802	1865	7752	7574	511	1848	1899
6668	6690	6730	6716	7921	7768	6759	6638	6636
1895	1833	1756	1919	7829	7657	1930	1881	1927

1892	1830	1753	1916	7826	7654	1927	1878	1924
2323	2254	2120	2202	7828	7689	2053	2324	2338
1881	1934	482	1869	7722	7581	1849	1867	1911
7609	7527	7613	7634	3464	2652	7582	7614	7567
7610	7528	7614	7635	3465	2653	7583	7615	7568
7684	7628	7698	7681	3315	2628	7651	7693	7664
177	1867	1808	1929	7752	7596	1891	5	205

1599	VRES0107	EC-F86E-R-141010	412049521	VREC0504	EC362	MS2481	2010031282
3520	3563	6791	7544	3532	3759	7530	7531
1974	1957	6688	7653	1960	1883	7577	7578
1965	353	6806	7637	1946	2101	7538	7539
3805	3760	7013	7834	3793	3833	7766	7767
3451	3513	6737	7512	3495	3667	7478	7479
24958	24975	25018	25546	25041	24969	25531	25532
1836	1776	6733	7660	1933	2071	7602	7603
196	1911	6671	7685	1956	2212	7614	7615
1810	1947	6795	7662	1975	2243	7579	7580
24957	24974	25017	25545	25040	24968	25530	25531
1840	1889	6777	7731	1882	2177	7677	7678
2391	2218	6867	7804	2246	1871	7697	7698
7622	7584	7737	3359	7645	7630	3422	3423
1837	1819	6688	7605	1869	2177	7502	7503
2051	1907	6807	7648	1870	2022	7548	7549
6837	6921	429	8051	6914	6968	8008	8009
1783	1823	6735	7613	1865	1894	7513	7514
2337	2076	6798	7754	2238	1982	7700	7701
2337	2076	6798	7754	2238	1982	7700	7701
2007	1856	6845	7712	1966	2258	7616	7617
3479	3352	6935	7847	3559	3803	7789	7790
2181	2136	6837	7673	2116	11	7572	7573
7712	7703	7797	3478	7724	7724	3521	3522
1884	1827	6799	7626	1868	2105	7539	7540
1952	1976	6808	7767	1944	2234	7717	7718
1882	1932	6782	7729	1932	2239	7675	7676
7644	7613	7687	3377	7654	7627	3430	3431
2062	1928	6780	7649	183	2152	7649	7650
7697	7654	7958	3358	7693	7697	3410	3411
7697	7654	7958	3358	7693	7697	3410	3411
1807	1819	6685	7606	1753	2123	7572	7573
30987	30997	30810	31166	30983	30944	31135	31136
7714	7690	7924	182	7705	7707	259	260
7798	7789	7996	2013	7788	7790	2063	2064
3403	3536	6793	7566	3515	3677	7540	7541
3403	3536	6793	7566	3515	3677	7540	7541
7627	7601	7771	3430	7659	7656	3499	3500
7753	7764	7934	2108	7778	7780	2144	2145
1963	2018	6767	7744	1984	2309	7680	7681
1940	1970	6800	7767	1946	2226	7717	7718
2182	2137	6838	7674	2117	12	7573	7574
3598	3635	6826	7526	3646	3780	7480	7481
7623	7598	7858	874	7619	7620	811	812
6664	6796	256	7941	6755	6839	7891	7892
6662	6784	86	7924	6736	6836	7873	7874
3466	3517	6745	7535	3522	3681	7500	7501
2029	2075	6722	7729	2062	2395	7690	7691
6692	6814	116	7953	6766	6866	7902	7903
2174	2041	6954	7859	2105	2447	7751	7752
7623	7608	7850	941	7615	7624	876	877
6644	6768	162	7949	6716	6814	7901	7902
7612	7559	7885	384	7613	7574	3	4
2187	2142	6843	7679	2122	17	7578	7579
2080	2037	6788	7705	2127	2350	7598	7599

241	1912	6696	7682	1957	2208	7600	7601
1901	1865	6714	7634	1932	2176	7527	7528
1790	1797	6779	7654	1896	1920	7570	7571
1955	1878	6730	7667	35	2104	7601	7602
7758	7750	7951	1995	7748	7755	2042	2043
7605	7575	7795	3458	7633	7630	3528	3529
1907	20	6787	7651	1887	2135	7556	7557
165	1892	6664	7679	1941	2193	7604	7605
283	1928	6651	7663	1965	2241	7572	7573
	1907	6660	7682	1966	2178	7611	7612
1907		6789	7653	1887	2135	7558	7559
6660	6789		7935	6739	6842	7884	7885
7682	7653	7935		7678	7674	381	382
1966	1887	6739	7678		2113	7612	7613
2178	2135	6842	7674	2113		7573	7574
7611	7558	7884	381	7612	7573		1
7612	7559	7885	382	7613	7574	1	
2413	2253	6878	7803	2255	1924	7696	7697
1974	1957	6688	7653	1960	1883	7577	7578
1969	1904	6715	7676	67	2132	7610	7611
7636	7614	7702	3345	7652	7623	3409	3410
2191	2152	6849	7679	2130	27	7578	7579
2085	2133	6737	7647	2116	2454	7561	7562
7763	7755	7958	2002	7753	7760	2049	2050
7694	7651	7953	3355	7690	7694	3407	3408
6680	6803	528	7816	6781	6852	7764	7765
215	1885	6685	7679	1938	2191	7600	7601
1950	334	6811	7651	1920	2080	7552	7553
7717	7693	7927	184	7708	7710	261	262
7615	7562	7888	385	7616	7577	4	5
7722	7699	7930	112	7714	7714	283	284
7721	7698	7929	107	7713	7713	282	283
7725	7702	7933	109	7717	7717	286	287
7714	7690	7924	183	7705	7707	256	257
2328	2124	6814	7758	2303	2053	7702	7703
7623	7585	7738	3360	7646	7631	3423	3424
2137	2127	6738	7785	2093	2362	7737	7738
7684	7661	7736	3348	7697	7680	3412	3413
2184	2162	6848	7683	2109	44	7582	7583
1852	1895	6782	7734	1885	2188	7680	7681
1910	1956	6793	7743	1954	2258	7689	7690
1923	1977	6824	7770	1925	2219	7703	7704
1937	327	6806	7649	1923	2075	7550	7551
1942	1974	6822	7778	1944	2226	7726	7727
7725	7702	7933	111	7717	7717	286	287
6588	6706	283	7953	6668	6760	7905	7906
6666	6788	90	7924	6738	6832	7873	7874
1852	1888	6774	7726	1902	2181	7672	7673
3496	3565	6761	7552	3549	3703	7514	7515
1850	1830	6699	7615	1882	2190	7512	7513
3441	3524	6794	7538	3572	3707	7495	7496
7712	7688	7923	180	7703	7705	257	258
3561	3641	6815	7559	3626	3749	7520	7521
6832	6916	424	8046	6909	6963	8003	8004
1798	1812	6767	7710	1806	2099	7660	7661

7718	7695	7927	191	7711	7711	266	267
1937	1969	6816	7774	1939	2221	7722	7723
1960	2003	6795	7746	1946	2249	7678	7679
3558	3606	6777	7508	3594	3751	7447	7448
6798	6888	419	8034	6872	6934	7966	7967
7612	7559	7887	384	7613	7574	3	4
7728	7705	7936	112	7720	7720	289	290
7641	7621	7710	3353	7657	7632	3413	3414
1947	1975	6800	7773	1953	2219	7723	7724
1869	607	6778	7666	1887	2079	7565	7566
7758	7750	7951	1995	7748	7755	2042	2043
2255	2128	6813	7730	2074	1674	7654	7655
1949	1973	6801	7773	1943	2223	7721	7722
1919	1968	6802	7755	1963	2267	7701	7702
6585	6703	280	7950	6665	6757	7902	7903
3553	3614	6807	7527	3601	3743	7490	7491
7686	7663	7738	3350	7699	7682	3414	3415
1912	1958	6795	7745	1956	2260	7691	7692
2194	2147	6836	7676	2089	84	7569	7570
1950	334	6811	7653	1920	2080	7554	7555
1781	1788	6770	7645	1887	1911	7561	7562
1940	1970	6800	7767	1946	2226	7717	7718
1986	1913	6746	7657	85	2151	7591	7592
1970	398	6823	7638	1932	2070	7539	7540
6668	6790	92	7928	6742	6842	7877	7878
7690	7667	7743	3355	7703	7686	3419	3420
7614	7561	7887	384	7615	7576	3	4
6586	6704	281	7951	6666	6758	7903	7904
6645	6771	103	7911	6719	6817	7866	7867
6587	6705	282	7952	6667	6759	7904	7905
3445	3525	6760	7495	3513	3677	7467	7468
2155	2143	6755	7800	2109	2380	7752	7753
1908	1948	6791	7769	1916	2196	7717	7718
2230	1933	6807	7710	2201	1884	7656	7657
2329	2175	6772	7727	2204	1926	7669	7670
3640	3710	6810	7600	3672	3837	7579	7580
3640	3710	6810	7600	3672	3837	7579	7580
7633	7611	7699	3342	7649	7620	3406	3407
167	1884	6675	7684	1927	2187	7613	7614
3602	3667	6800	7548	3654	3801	7511	7512
1976	465	6782	7631	1876	2088	7528	7529
3504	3583	6783	7523	3566	3734	7495	7496
1848	1897	6785	7727	1893	2198	7673	7674
3543	3657	6780	7577	3651	3780	7535	7536
1952	1998	6729	7711	2019	2300	7676	7677
6657	6789	255	7936	6748	6830	7886	7887
1849	1831	6700	7618	1881	2189	7515	7516
6684	6806	76	7946	6766	6864	7895	7896
1852	1849	6747	7649	1880	1906	7557	7558
3420	3552	6754	7517	3550	3692	7498	7499
1790	1797	6779	7654	1896	1920	7570	7571
3446	3538	6763	7498	3515	3655	7487	7488
7613	7560	7886	383	7614	7575	2	3
7613	7560	7886	383	7614	7575	2	3
7614	7561	7887	384	7615	7576	3	4

7612	7559	7885	382	7613	7574	1	2
6670	6792	60	7932	6752	6850	7880	7881
2228	2137	6842	7666	2062	128	7565	7566
2020	2066	6713	7720	2053	2386	7681	7682
1948	332	6809	7651	1918	2078	7552	7553
7624	7586	7739	3359	7647	7632	3422	3423
1975	2032	6782	7750	1996	2328	7686	7687
1891	387	6760	7656	1818	2076	7559	7560
1826	1847	6748	7655	1894	1925	7557	7558
6636	6760	162	7938	6708	6810	7890	7891
3541	3601	6832	7527	3586	3735	7479	7480
7613	7560	7886	384	7614	7575	3	4
7612	7559	7885	382	7613	7574	1	2
7579	7573	7877	349	7604	7564	320	321
7614	7561	7887	385	7615	7576	4	5
7614	7561	7887	385	7615	7576	4	5
2203	2204	6821	7683	2154	99	7582	7583
2013	2059	6706	7713	2046	2379	7674	7675
2130	2088	6739	7774	2078	2343	7726	7727
177	1889	6663	7683	1947	2181	7612	7613
151	1865	6657	7668	1929	2181	7597	7598
1821	1844	6743	7654	1889	1920	7554	7555
1827	1848	6749	7657	1895	1926	7557	7558
2182	2137	6838	7674	2117	12	7573	7574
1964	1885	6739	7676	28	2113	7610	7611
1956	1877	6731	7668	34	2105	7602	7603
498	1992	6670	7643	2039	2296	7531	7532
1970	2029	6787	7743	1993	2325	7679	7680
1806	1818	6684	7603	1752	2122	7567	7568
1856	423	6759	7651	1808	2042	7554	7555
1782	1787	6745	7628	1858	1864	7526	7527
3546	3621	6741	7548	3599	3736	7497	7498
6658	6790	250	7935	6749	6833	7885	7886
3418	3549	6753	7514	3548	3689	7495	7496
1938	1968	6797	7764	1944	2224	7714	7715
7625	7599	7769	3428	7657	7654	3497	3498
3418	3549	6753	7514	3548	3689	7495	7496
1870	1898	6797	7751	1918	2196	7697	7698
3445	3525	6764	7495	3511	3679	7467	7468
1798	1812	6767	7710	1806	2099	7660	7661
1856	1899	6776	7706	1889	2192	7652	7653
1927	1967	6818	7753	1934	2213	7691	7692
1801	1813	6679	7600	1747	2117	7566	7567
6808	6911	441	8052	6884	6953	8005	8006
2143	2147	6745	7790	2102	2376	7740	7741
3479	3560	6740	7528	3536	3702	7511	7512
2190	2028	6813	7727	2037	1588	7645	7646
3468	3341	6925	7836	3548	3792	7778	7779
1841	1838	6736	7639	1869	1895	7547	7548
1975	1973	6689	7651	1966	1883	7575	7576
7626	7600	7770	3429	7658	7655	3498	3499
1941	1916	6763	7730	1961	2248	7684	7685
1916	1946	6806	7772	1918	2200	7722	7723
7618	7593	7738	3309	7631	7612	3369	3370
3566	3637	6791	7552	3607	3781	7531	7532

3473	3581	6781	7548	3529	3704	7520	7521
1859	603	6776	7667	1881	2069	7566	7567
1962	2013	6766	7734	1983	2314	7670	7671
7624	7598	7768	3427	7656	7653	3496	3497
3501	3586	6797	7515	3560	3718	7487	7488
3501	3586	6797	7515	3560	3718	7487	7488
7609	7584	7729	3300	7622	7603	3360	3361
1955	1876	6730	7667	35	2104	7601	7602
1860	425	6762	7653	1810	2044	7556	7557
3415	3542	6793	7575	3513	3682	7549	7550
3540	3630	6769	7501	3619	3755	7440	7441
1774	1779	6737	7620	1850	1856	7518	7519
1806	1818	6684	7605	1752	2122	7571	7572
1934	1936	6806	7741	1929	2210	7691	7692
2283	2061	6806	7760	2272	2017	7706	7707
7712	7688	7922	175	7703	7705	256	257
2015	2037	6790	7736	1989	2280	7689	7690
7624	7598	7768	3427	7656	7653	3496	3497
7623	7597	7767	3426	7655	7652	3495	3496
2180	2135	6836	7672	2115	10	7571	7572
1880	515	6724	7637	1861	2104	7528	7529
6649	6771	175	7920	6722	6821	7872	7873
2228	2137	6842	7666	2062	128	7565	7566
2228	2137	6842	7666	2062	128	7565	7566
7625	7599	7769	3428	7657	7654	3497	3498
1861	1891	6790	7744	1911	2189	7690	7691
1849	1898	6786	7730	1893	2198	7676	7677
1801	1813	6679	7600	1747	2117	7566	7567
3506	3586	6785	7527	3568	3737	7498	7499
3555	3637	6798	7556	3604	3772	7535	7536
7624	7586	7739	3361	7647	7632	3424	3425
1819	1842	6740	7652	1887	1918	7552	7553
1982	1967	6686	7652	1980	1903	7576	7577
1969	397	6822	7637	1931	2069	7538	7539
1970	1891	6745	7680	50	2119	7614	7615
1886	1829	6801	7628	1870	2107	7541	7542
1884	1827	6799	7626	1868	2105	7539	7540
1915	1858	6827	7655	1899	2136	7568	7569
6682	6809	301	7965	6762	6840	7902	7903
3518	3645	6775	7511	3623	3769	7477	7478
1956	1877	6731	7668	36	2104	7602	7603
2326	2094	6797	7726	2166	1915	7672	7673
7610	7585	7730	3301	7623	7604	3361	3362
7703	7680	7755	3367	7716	7699	3431	3432
3490	3576	6781	7559	3564	3702	7532	7533
1800	1843	6733	7636	1873	1921	7566	7567
7699	7656	7960	3360	7695	7699	3412	3413
2183	2138	6839	7675	2118	13	7574	7575
2189	2144	6845	7681	2124	19	7580	7581
1813	1808	6760	7640	1881	1881	7538	7539
1934	1964	6796	7762	1936	2220	7710	7711
6704	6818	138	7945	6790	6878	7897	7898
1893	511	6749	7658	1872	2118	7549	7550
6634	6761	326	7907	6725	6806	7857	7858
1880	1930	6780	7727	1930	2237	7673	7674

1877	1927	6777	7724	1927	2234	7670	7671
2286	2053	6792	7747	2213	2005	7693	7694
1852	1849	6747	7649	1880	1906	7557	7558
7622	7584	7737	3359	7645	7630	3422	3423
7623	7585	7738	3360	7646	7631	3423	3424
7696	7653	7955	3357	7692	7696	3409	3410
164	1891	6663	7680	1940	2192	7605	7606

SCK02-43	SCP21-24	SCP29-34	ERS1340929	ERS1340998	ERS1341034	SCK53-23	SCP24-18
3735	3593	3531	7564	3768	3773	7708	7689
2028	10	1973	7623	1876	2099	7750	7700
2281	2067	1965	7619	2120	2084	7737	7666
3903	3853	3812	7721	3832	3995	7929	7802
3659	3505	3490	7530	3678	3735	7655	7641
25012	24973	25036	25521	24967	25035	25600	25572
2307	1813	1942	7625	2076	2111	7775	7690
2458	1969	1959	7630	2225	2074	7762	7687
2351	1946	1958	7619	2258	2206	7760	7691
25011	24972	25035	25520	24966	25034	25599	25571
2260	1805	1885	7679	2190	494	7834	7738
160	2028	2237	7763	1870	2527	7896	7816
7758	7588	7645	2342	7633	7553	3471	2515
2372	1875	1872	7537	2188	2067	7691	7614
1965	1347	1865	7633	2035	2144	7749	7698
7022	6843	6914	7807	6977	6881	8069	8065
2115	1742	1868	7577	1887	1938	7685	7646
1757	2174	2247	7708	1989	2611	7840	7784
1757	2174	2247	7708	1989	2611	7840	7784
2260	2086	1969	7673	2277	2117	7820	7728
3817	3637	3546	7769	3814	3747	7899	7893
1917	1886	2135	7622	20	2457	7761	7693
7843	7689	7726	2643	7725	7669	3486	2613
2148	1909	1877	7599	2124	2047	7738	7660
2346	1845	1953	7710	2243	736	7881	7774
2308	1871	1933	7677	2250	543	7836	7741
7755	7620	7658	409	7628	7570	3462	2467
2300	2028	198	7634	2169	2139	7732	7672
7816	7702	7695	2463	7698	7653	3324	41
7816	7702	7695	2463	7698	7653	3324	41
2284	1866	1762	7570	2142	2125	7711	7639
30934	30956	30975	31216	30948	30989	31276	31235
7838	7684	7703	3281	7712	7634	1889	3288
7929	7777	7788	3443	7797	7779	224	3320
3629	3558	3514	7578	3688	3661	7715	7700
3629	3558	3514	7578	3688	3661	7715	7700
7763	7626	7659	2647	7657	7630	3387	2601
7905	7772	7774	3391	7785	7747	1924	3335
2390	1964	1961	7664	2322	586	7839	7725
2334	1829	1955	7706	2229	732	7879	7770
1918	1887	2136	7623	21	2458	7762	7694
3796	3633	3643	7541	3791	3822	7660	7648
7757	7596	7619	3454	7627	7577	2122	3478
6892	6712	6737	7696	6848	6753	7960	7959
6880	6682	6712	7691	6845	6754	7947	7942
3673	3541	3527	7540	3692	3715	7668	7645
2462	2011	2061	7644	2392	633	7831	7751
6910	6712	6742	7720	6875	6784	7975	7971
2595	2186	2108	7781	2458	2358	7932	7845
7761	7598	7615	3495	7631	7581	2182	3521
6856	6656	6692	7721	6823	6791	7974	7969
7697	7578	7611	3412	7579	7562	2052	3410
1922	1892	2141	7628	26	2463	7767	7699
2491	2078	2130	7620	2349	2241	7776	7670

2447	1965	1960	7623	2221	2055	7759	7682
2399	1919	1935	7543	2185	2061	7700	7626
2194	1783	1907	7622	1913	1992	7721	7696
2244	1949	48	7641	2121	2105	7742	7679
7893	7745	7746	3417	7762	7746	15	3316
7737	7600	7633	2675	7631	7654	3416	2629
2251	1957	1904	7612	2152	2133	7753	7649
2441	1943	1928	7631	2206	2078	7756	7691
2467	1985	1962	7582	2254	2058	7735	7662
2413	1974	1969	7636	2191	2085	7763	7694
2253	1957	1904	7614	2152	2133	7755	7651
6878	6688	6715	7702	6849	6737	7958	7953
7803	7653	7676	3345	7679	7647	2002	3355
2255	1960	67	7652	2130	2116	7753	7690
1924	1883	2132	7623	27	2454	7760	7694
7696	7577	7610	3409	7578	7561	2049	3407
7697	7578	7611	3410	7579	7562	2050	3408
	2028	2246	7755	1923	2524	7898	7813
2028		1973	7623	1876	2099	7750	7700
2246	1973		7654	2149	2113	7751	7692
7755	7623	7654		7624	7563	3425	2462
1923	1876	2149	7624		2467	7767	7695
2524	2099	2113	7563	2467		7751	7650
7898	7750	7751	3425	7767	7751		3324
7813	7700	7692	2462	7695	7650	3324	
6896	6726	6763	7587	6861	6742	7831	7846
2441	1925	1937	7627	2204	2082	7760	7687
2257	2043	1939	7623	2099	2112	7749	7654
7841	7687	7706	3283	7715	7637	1891	3290
7700	7581	7614	3413	7582	7565	2053	3411
7847	7693	7712	3277	7719	7685	1918	3290
7846	7692	7711	3276	7718	7684	1917	3289
7850	7696	7715	3280	7722	7688	1921	3293
7838	7684	7703	3280	7712	7634	1888	3287
1817	2213	2314	7713	2046	2601	7852	7787
7759	7589	7646	2343	7634	7554	3472	2516
2470	1959	2102	7725	2371	984	7905	7793
7791	7674	7699	635	7681	7611	3391	2448
1948	1893	2128	7630	63	2438	7771	7703
2263	1813	1888	7682	2201	496	7837	7740
2335	1887	1957	7670	2271	569	7848	7725
2327	1834	1934	7715	2232	659	7877	7792
2260	2040	1942	7623	2094	2112	7751	7657
2340	1847	1953	7719	2239	658	7881	7796
7850	7696	7715	3280	7722	7688	1921	3293
6786	6614	6644	7747	6769	6860	7975	7981
6884	6680	6714	7693	6841	6754	7949	7944
2250	1809	1905	7675	2194	503	7827	7732
3707	3549	3546	7545	3714	3791	7681	7663
2385	1888	1885	7546	2201	2080	7699	7624
3696	3603	3573	7572	3716	3698	7697	7691
7836	7682	7701	3279	7710	7632	1887	3286
3739	3630	3629	7568	3758	3859	7703	7680
7017	6838	6909	7802	6972	6876	8064	8060
2203	1769	1809	7665	2112	703	7816	7729

7842	7688	7709	3280	7716	7639	1898	3289
2335	1842	1948	7717	2234	653	7876	7794
2363	1888	1951	7684	2262	572	7856	7752
3747	3584	3597	7516	3760	3840	7648	7624
6980	6805	6874	7786	6943	6836	8050	8044
7697	7578	7611	3410	7579	7562	2052	3408
7853	7699	7718	3283	7725	7691	1924	3296
7760	7628	7659	62	7633	7571	3429	2464
2355	1838	1962	7708	2234	748	7885	7779
2132	1823	1904	7635	2098	1997	7782	7674
7893	7745	7746	3417	7762	7746	15	3316
1654	1961	2073	7693	1693	2396	7809	7771
2331	1838	1952	7710	2232	689	7878	7777
2344	1896	1966	7684	2280	578	7860	7739
6783	6611	6641	7744	6766	6857	7972	7978
3746	3606	3604	7549	3748	3758	7671	7678
7793	7676	7701	637	7683	7613	3393	2450
2337	1889	1959	7672	2273	571	7850	7727
1890	1881	2094	7619	93	2434	7760	7694
2257	2043	1939	7625	2099	2112	7751	7656
2185	1774	1898	7613	1904	1983	7712	7687
2334	1829	1955	7706	2229	732	7879	7770
2284	1993	108	7639	2166	2111	7736	7695
2297	2031	1951	7601	2089	2141	7734	7640
6886	6688	6718	7697	6851	6760	7953	7948
7797	7680	7705	642	7687	7618	3398	2455
7699	7580	7613	3412	7581	7564	2052	3410
6784	6612	6642	7745	6767	6858	7973	7979
6863	6665	6695	7682	6826	6751	7940	7931
6785	6613	6643	7746	6768	6859	7974	7980
3656	3525	3494	7513	3686	3719	7638	7640
2484	1977	2118	7740	2389	1000	7920	7810
2316	1815	1925	7712	2209	647	7876	7778
1909	2060	2210	7653	1867	2430	7801	7727
1786	2149	2213	7681	1909	2551	7817	7777
3820	3700	3665	7610	3848	3927	7745	7720
3820	3700	3665	7610	3848	3927	7745	7720
7752	7620	7651	11	7621	7560	3422	2459
2439	1935	1930	7629	2200	2077	7761	7691
3782	3648	3657	7566	3812	3815	7692	7697
2200	2057	1889	7577	2107	2060	7725	7623
3709	3580	3547	7562	3743	3764	7666	7677
2272	1819	1896	7680	2211	496	7834	7741
3738	3693	3634	7581	3791	3884	7737	7715
2382	1958	1994	7655	2311	607	7827	7735
6885	6705	6730	7691	6839	6754	7955	7954
2384	1887	1884	7549	2200	2079	7704	7627
6902	6712	6742	7715	6873	6784	7973	7966
2150	1745	1889	7616	1899	1938	7727	7686
3673	3581	3529	7528	3701	3716	7679	7667
2194	1783	1907	7622	1913	1992	7721	7696
3653	3527	3530	7521	3666	3706	7648	7631
7698	7579	7612	3411	7580	7563	2051	3409
7698	7579	7612	3411	7580	7563	2051	3409
7697	7580	7613	3412	7581	7564	2052	3410

7697	7578	7611	3410	7579	7562	2050	3408
6886	6698	6728	7698	6859	6770	7956	7949
1913	1885	2083	7614	137	2464	7753	7679
2453	2002	2052	7635	2383	624	7822	7742
2255	2041	1937	7623	2097	2110	7749	7654
7760	7589	7647	2340	7635	7554	3471	2517
2407	1980	1973	7668	2341	595	7847	7726
2241	1969	1837	7620	2095	2023	7749	7659
2156	1771	1905	7616	1918	2019	7725	7697
6846	6650	6684	7712	6819	6786	7965	7963
3720	3587	3585	7565	3744	3801	7678	7669
7698	7579	7612	3412	7580	7563	2052	3410
7697	7578	7611	3410	7579	7562	2050	3408
7689	7547	7590	3368	7569	7556	2050	3372
7699	7580	7613	3413	7581	7564	2053	3411
7699	7580	7613	3413	7581	7564	2053	3411
1917	1902	2173	7635	108	2443	7778	7705
2446	1995	2045	7628	2376	617	7815	7735
2449	1952	2087	7716	2362	977	7894	7788
2443	1945	1950	7629	2194	2086	7757	7692
2439	1933	1932	7613	2194	2060	7747	7678
2151	1766	1900	7611	1913	2014	7722	7694
2157	1772	1906	7617	1919	2020	7727	7698
1918	1887	2136	7623	21	2458	7762	7694
2253	1958	65	7650	2130	2114	7751	7688
2245	1950	57	7642	2122	2106	7743	7680
2503	2051	2042	7578	2307	2102	7712	7638
2404	1977	1970	7661	2338	600	7840	7721
2283	1865	1761	7567	2141	2124	7708	7636
2215	1939	1827	7610	2061	1999	7744	7651
2110	1703	1869	7595	1857	1955	7698	7662
3714	3610	3602	7539	3743	3828	7692	7657
6886	6706	6731	7690	6842	6747	7954	7953
3670	3578	3527	7525	3698	3714	7676	7665
2332	1827	1953	7703	2227	730	7876	7767
7761	7624	7657	2645	7655	7628	3385	2599
3670	3578	3527	7525	3698	3714	7676	7665
2279	1833	1921	7700	2207	532	7854	7758
3656	3523	3492	7515	3688	3719	7640	7640
2203	1769	1809	7665	2112	703	7816	7729
2265	1816	1892	7662	2205	470	7809	7737
2311	1862	1929	7700	2226	639	7858	7777
2278	1860	1756	7564	2136	2119	7705	7633
7011	6825	6878	7828	6962	6865	8081	8068
2486	1974	2107	7730	2385	978	7912	7800
3702	3546	3521	7546	3715	3811	7681	7665
1721	1889	2028	7688	1607	2325	7809	7773
3806	3626	3535	7756	3803	3736	7888	7880
2139	1734	1878	7608	1888	1927	7717	7676
2018	74	1979	7619	1874	2091	7750	7696
7762	7625	7658	2646	7656	7629	3386	2600
2333	1884	1976	7671	2245	653	7837	7743
2326	1815	1927	7714	2213	705	7884	7780
7731	7608	7633	295	7613	7550	3360	2364
3765	3637	3600	7561	3786	3857	7695	7674

3685	3540	3510	7561	3715	3773	7689	7677
2136	1815	1898	7636	2088	1998	7783	7678
2393	1967	1960	7652	2327	582	7831	7710
7760	7623	7656	2644	7654	7627	3384	2598
3698	3562	3541	7540	3727	3807	7654	7634
3698	3562	3541	7540	3727	3807	7654	7634
7722	7599	7624	286	7604	7541	3351	2355
2244	1949	58	7641	2121	2105	7742	7679
2217	1941	1829	7612	2063	2001	7746	7653
3633	3558	3512	7585	3693	3664	7724	7704
3746	3621	3622	7524	3764	3806	7641	7647
2102	1695	1861	7587	1849	1947	7690	7654
2283	1865	1761	7569	2141	2124	7710	7638
2333	1830	1938	7689	2229	699	7853	7771
1747	2136	2281	7715	2000	2573	7852	7794
7836	7682	7701	3278	7710	7632	1886	3285
2404	1891	1998	7667	2293	678	7852	7744
7760	7623	7656	2644	7654	7627	3384	2598
7759	7622	7655	2643	7653	7626	3383	2597
1916	1885	2134	7621	19	2456	7760	7692
2245	1995	1844	7602	2115	2005	7738	7642
6862	6662	6698	7706	6830	6776	7951	7971
1913	1885	2083	7614	137	2464	7753	7679
1913	1885	2083	7614	137	2464	7753	7679
7761	7624	7657	2645	7655	7628	3385	2599
2272	1824	1914	7693	2200	523	7847	7753
2273	1820	1896	7681	2211	496	7837	7742
2278	1860	1756	7564	2136	2119	7705	7633
3712	3579	3549	7566	3746	3766	7672	7679
3763	3626	3597	7566	3783	3854	7699	7678
7760	7590	7647	2344	7635	7555	3473	2517
2149	1764	1898	7609	1911	2012	7720	7692
2048	32	1993	7624	1896	2100	7753	7704
2296	2030	1950	7600	2088	2140	7735	7639
2259	1964	73	7654	2136	2120	7755	7692
2150	1911	1879	7601	2126	2049	7740	7662
2148	1909	1877	7599	2124	2047	7738	7660
2179	1940	1908	7628	2155	2078	7765	7689
6884	6705	6738	7721	6849	6759	7985	7982
3706	3629	3620	7528	3780	3780	7653	7665
2245	1950	59	7642	2121	2106	7743	7680
1652	2120	2175	7673	1898	2556	7798	7752
7723	7600	7625	287	7605	7542	3352	2356
7810	7693	7718	654	7700	7630	3409	2467
3682	3574	3545	7583	3711	3809	7700	7697
2142	1716	1882	7611	1914	1929	7711	7684
7818	7704	7697	2465	7700	7655	3326	43
1919	1888	2137	7624	22	2459	7763	7695
1925	1894	2143	7630	28	2465	7769	7701
2127	1722	1892	7604	1874	1980	7711	7673
2326	1833	1945	7701	2229	682	7869	7768
6920	6728	6766	7713	6887	6780	7973	7966
2263	2006	1855	7623	2135	2021	7757	7662
6857	6683	6707	7656	6815	6719	7928	7916
2306	1869	1931	7675	2248	541	7834	7739

2303	1866	1928	7672	2245	538	7831	7736
1764	2181	2222	7700	1988	2558	7833	7770
2150	1745	1889	7616	1899	1938	7727	7686
7758	7588	7645	2342	7633	7553	3471	2515
7759	7589	7646	2343	7634	7554	3472	2516
7815	7701	7694	2462	7697	7652	3323	40
2440	1942	1927	7630	2205	2077	7757	7690

E.	509sc	ME160327	AM_LREC-61	AM_LREC-98	AM_LREC-15	AM_LREC-63	AM_LREC-128
6821	3536	3596	7610	7534	7614	7613	7617
6726	1925	2043	7687	7581	7693	7692	7696
6804	1931	40	7677	7542	7683	7682	7686
7022	3810	3802	7864	7770	7867	7866	7870
6773	3456	3522	7574	7482	7578	7577	7581
25033	24988	24978	25539	25535	25553	25552	25555
6748	1767	1794	7698	7606	7706	7705	7709
6701	191	1940	7720	7618	7725	7724	7728
6825	1792	1952	7699	7583	7706	7705	7709
25032	24987	24977	25538	25534	25552	25551	25554
6829	1814	1881	7763	7681	7771	7770	7774
6885	2419	2222	7842	7701	7848	7847	7851
7651	7612	7603	3302	3426	3290	3289	3293
6722	1771	1840	7641	7506	7647	7646	7650
6800	2051	1961	7686	7552	7692	7691	7695
702	6846	6947	8040	8012	8046	8045	8049
6753	1803	1761	7649	7517	7655	7654	7658
6844	2367	2105	7794	7704	7800	7799	7803
6844	2367	2105	7794	7704	7800	7799	7803
6844	1952	1884	7750	7620	7758	7757	7761
6959	3469	3291	7873	7793	7876	7875	7878
6849	2194	2083	7709	7576	7713	7712	7716
7683	7702	7700	3388	3525	3404	3403	3407
6817	1841	1831	7669	7543	7676	7675	7679
6843	1932	1996	7799	7721	7807	7806	7810
6827	1858	1926	7761	7679	7769	7768	7772
7572	7633	7622	3310	3434	3310	3309	3313
6801	2022	1961	7679	7653	7685	7684	7688
7851	7690	7657	3293	3414	3293	3292	3296
7851	7690	7657	3293	3414	3293	3292	3296
6714	1756	1817	7644	7576	7650	7649	7653
30819	31007	31007	31163	31139	31177	31177	31182
7799	7711	7688	20	263	82	81	85
7869	7797	7787	1907	2067	1929	1928	1932
6801	3444	3601	7620	7544	7626	7625	7629
6801	3444	3601	7620	7544	7626	7625	7629
7669	7617	7616	3381	3503	3369	3368	3372
7808	7756	7767	2017	2148	2027	2026	2030
6817	1965	1996	7764	7684	7772	7771	7775
6845	1920	1992	7799	7721	7807	7806	7810
6850	2195	2084	7710	7577	7714	7713	7717
6802	3608	3648	7572	7484	7576	7575	7579
7744	7622	7600	777	815	784	783	787
292	6680	6812	7929	7895	7932	7931	7935
455	6687	6808	7916	7877	7919	7918	7922
6774	3487	3531	7590	7504	7594	7593	7597
6858	2029	2063	7752	7694	7760	7759	7763
485	6717	6838	7945	7906	7948	7947	7951
6989	2115	2029	7890	7755	7896	7895	7899
7734	7622	7610	848	880	851	850	854
537	6669	6790	7944	7905	7948	7947	7951
7765	7601	7553	264	7	286	285	289
6855	2200	2089	7715	7582	7719	7718	7722
6829	2021	2043	7737	7602	7743	7742	7746

6717	178	1945	7717	7604	7722	7721	7725
6752	1830	1878	7666	7531	7672	7671	7675
6778	1795	1749	7690	7574	7696	7695	7699
6772	1927	1913	7697	7605	7703	7702	7706
7824	7755	7744	1884	2046	1911	1910	1914
7693	7595	7590	3410	3532	3397	3396	3400
6801	1885	334	7691	7560	7697	7696	7700
6684	140	1925	7714	7608	7719	7718	7722
6682	206	1965	7690	7576	7695	7694	7698
6680	215	1950	7717	7615	7722	7721	7725
6803	1885	334	7693	7562	7699	7698	7702
528	6685	6811	7927	7888	7930	7929	7933
7816	7679	7651	184	385	112	107	109
6781	1938	1920	7708	7616	7714	7713	7717
6852	2191	2080	7710	7577	7714	7713	7717
7764	7600	7552	261	4	283	282	286
7765	7601	7553	262	5	284	283	287
6896	2441	2257	7841	7700	7847	7846	7850
6726	1925	2043	7687	7581	7693	7692	7696
6763	1937	1939	7706	7614	7712	7711	7715
7587	7627	7623	3283	3413	3277	3276	3280
6861	2204	2099	7715	7582	7719	7718	7722
6742	2082	2112	7637	7565	7685	7684	7688
7831	7760	7749	1891	2053	1918	1917	1921
7846	7687	7654	3290	3411	3290	3289	3293
	6696	6820	7802	7768	7805	7804	7808
6696		1916	7714	7604	7719	7718	7722
6820	1916		7691	7556	7697	7696	7700
7802	7714	7691		265	84	83	87
7768	7604	7556	265		287	286	290
7805	7719	7697	84	287		11	15
7804	7718	7696	83	286	11		10
7808	7722	7700	87	290	15	10	
7799	7711	7688	23	260	83	82	86
6864	2362	2136	7798	7706	7804	7803	7807
7652	7613	7604	3303	3427	3291	3290	3294
6788	2117	2141	7819	7741	7827	7826	7830
7617	7673	7668	3282	3416	3282	3281	3285
6859	2197	2092	7719	7586	7723	7722	7726
6831	1826	1875	7766	7684	7774	7773	7777
6844	1884	1954	7775	7693	7783	7782	7786
6844	1892	1949	7802	7707	7810	7809	7813
6816	1903	21	7689	7554	7695	7694	7698
6855	1922	1970	7810	7730	7818	7817	7821
7808	7722	7700	87	290	15	8	14
664	6613	6730	7946	7909	7954	7953	7957
459	6691	6812	7916	7877	7919	7918	7922
6828	1826	1891	7758	7676	7766	7765	7769
6785	3509	3576	7602	7518	7606	7605	7609
6733	1784	1851	7651	7516	7657	7656	7660
6811	3482	3577	7598	7499	7604	7603	7607
7798	7709	7686	18	261	80	79	83
6842	3589	3661	7615	7524	7619	7618	7622
697	6841	6942	8035	8007	8041	8040	8044
6811	1772	1806	7748	7664	7754	7753	7757

7802	7715	7692	31	270	91	90	94
6849	1917	1965	7806	7726	7814	7813	7817
6844	1962	1997	7778	7682	7786	7785	7789
6820	3570	3632	7559	7451	7563	7562	7566
675	6802	6906	8024	7970	8029	8028	8032
7767	7601	7553	264	7	286	285	289
7811	7725	7703	90	293	18	13	11
7595	7632	7630	3287	3417	3285	3284	3288
6840	1927	1997	7805	7727	7813	7812	7816
6802	1847	643	7705	7569	7710	7709	7713
7824	7755	7744	1884	2046	1911	1910	1914
6841	2273	2122	7770	7658	7776	7775	7779
6852	1929	1965	7805	7725	7813	7812	7816
6853	1893	1966	7787	7705	7795	7794	7798
661	6610	6727	7943	7906	7951	7950	7954
6790	3584	3634	7583	7494	7587	7586	7590
7619	7675	7670	3284	3418	3284	3283	3287
6846	1886	1956	7777	7695	7785	7784	7788
6850	2187	2094	7712	7573	7716	7715	7719
6820	1916	4	7693	7558	7699	7698	7702
6769	1786	1740	7681	7565	7687	7686	7690
6845	1920	1992	7799	7721	7807	7806	7810
6759	1958	1955	7687	7595	7693	7692	7696
6833	1934	118	7678	7543	7684	7683	7687
461	6693	6814	7920	7881	7923	7922	7926
7624	7679	7674	3289	3423	3289	3288	3292
7767	7603	7555	264	7	286	285	289
662	6611	6728	7944	7907	7952	7951	7955
470	6670	6795	7909	7870	7910	7909	7913
663	6612	6729	7945	7908	7953	7952	7956
6740	3469	3552	7559	7471	7563	7562	7566
6803	2135	2157	7834	7756	7842	7841	7845
6845	1888	1942	7801	7721	7809	7808	7812
6837	2235	1954	7750	7660	7756	7755	7759
6814	2355	2174	7761	7673	7769	7768	7772
6857	3648	3719	7656	7583	7660	7659	7663
6857	3648	3719	7656	7583	7660	7659	7663
7584	7624	7620	3280	3410	3274	3273	3277
6694	160	1913	7719	7617	7724	7723	7727
6812	3593	3675	7605	7515	7609	7608	7612
6797	1944	463	7665	7532	7671	7670	7674
6778	3526	3608	7587	7499	7591	7590	7594
6824	1822	1883	7759	7677	7767	7766	7770
6795	3596	3704	7627	7539	7631	7630	7634
6810	1952	2011	7744	7680	7752	7751	7755
293	6673	6805	7924	7890	7927	7926	7930
6734	1783	1852	7654	7519	7660	7659	7663
489	6709	6830	7938	7899	7941	7940	7944
6755	1853	1808	7689	7561	7695	7694	7698
6780	3487	3592	7576	7502	7580	7579	7583
6778	1795	1749	7690	7574	7696	7695	7699
6765	3503	3538	7561	7491	7565	7564	7568
7766	7602	7554	263	6	285	284	288
7766	7602	7554	263	6	285	284	288
7767	7603	7555	264	7	286	285	289

7765	7601	7553	262	5	284	283	287
473	6695	6816	7923	7884	7926	7925	7929
6854	2215	2092	7702	7569	7706	7705	7709
6849	2020	2054	7743	7685	7751	7750	7754
6818	1914	2	7691	7556	7697	7696	7700
7653	7614	7605	3302	3426	3290	3289	3293
6823	1977	2006	7770	7690	7778	7777	7781
6779	1869	439	7694	7563	7700	7699	7703
6762	1829	1791	7693	7561	7699	7698	7702
524	6661	6782	7933	7894	7937	7936	7940
6800	3548	3630	7591	7483	7595	7594	7598
7766	7602	7554	264	7	286	285	289
7765	7601	7553	262	5	284	283	287
7744	7565	7571	241	324	263	262	266
7767	7603	7555	265	8	287	286	290
7767	7603	7555	265	8	287	286	290
6837	2216	2151	7719	7586	7723	7722	7726
6842	2013	2047	7736	7678	7744	7743	7747
6787	2110	2102	7808	7730	7816	7815	7819
6696	172	1937	7718	7616	7723	7722	7726
6688	146	1914	7703	7601	7708	7707	7711
6757	1824	1788	7692	7558	7698	7697	7701
6763	1830	1792	7695	7561	7701	7700	7704
6850	2195	2084	7710	7577	7714	7713	7717
6781	1936	1920	7706	7614	7712	7711	7715
6773	1928	1912	7698	7606	7704	7703	7707
6700	439	2055	7669	7535	7675	7674	7678
6812	1972	2003	7763	7683	7771	7770	7774
6713	1755	1816	7639	7571	7645	7644	7648
6778	1834	475	7689	7558	7695	7694	7698
6759	1787	1737	7664	7530	7670	7669	7673
6781	3579	3630	7600	7501	7604	7603	7607
286	6674	6806	7923	7889	7926	7925	7929
6779	3485	3589	7573	7499	7577	7576	7580
6841	1918	1990	7796	7718	7804	7803	7807
7667	7615	7614	3379	3501	3367	3366	3370
6779	3485	3589	7573	7499	7577	7576	7580
6851	1844	1916	7783	7701	7791	7790	7794
6744	3469	3552	7559	7471	7563	7562	7566
6811	1772	1806	7748	7664	7754	7753	7757
6805	1830	1884	7738	7656	7746	7745	7749
6830	1925	1951	7785	7695	7793	7792	7796
6708	1750	1811	7638	7570	7644	7643	7647
623	6814	6937	8047	8009	8050	8049	8053
6792	2120	2149	7824	7744	7832	7831	7835
6767	3505	3571	7590	7515	7594	7593	7597
6837	2225	2030	7767	7649	7773	7772	7776
6949	3458	3280	7862	7782	7865	7864	7867
6744	1842	1797	7679	7551	7685	7684	7688
6722	1926	2047	7685	7579	7691	7690	7694
7668	7616	7615	3380	3502	3368	3367	3371
6853	1921	1904	7762	7688	7770	7769	7773
6844	1896	1968	7804	7726	7812	7811	7815
7623	7611	7602	3243	3373	3241	3240	3244
6831	3583	3650	7608	7535	7612	7611	7615

6780	3495	3571	7612	7524	7616	7615	7619
6803	1837	641	7706	7570	7711	7710	7714
6807	1964	1993	7754	7674	7762	7761	7765
7666	7614	7613	3378	3500	3366	3365	3369
6779	3523	3596	7579	7491	7583	7582	7586
6779	3523	3596	7579	7491	7583	7582	7586
7614	7602	7593	3234	3364	3232	3231	3235
6772	1927	1911	7697	7605	7703	7702	7706
6781	1838	477	7691	7560	7697	7696	7700
6812	3456	3595	7629	7553	7635	7634	7638
6801	3595	3655	7552	7444	7556	7555	7559
6751	1779	1729	7656	7522	7662	7661	7665
6713	1755	1816	7643	7575	7649	7648	7652
6821	1914	1955	7773	7695	7781	7780	7784
6857	2313	2116	7800	7710	7806	7805	7809
7797	7709	7686	19	260	75	74	78
6842	1995	2043	7775	7693	7776	7775	7779
7666	7614	7613	3378	3500	3366	3365	3369
7665	7613	7612	3377	3499	3365	3364	3368
6848	2193	2082	7708	7575	7712	7711	7715
6764	1863	560	7675	7532	7681	7680	7684
512	6674	6794	7915	7876	7919	7918	7922
6854	2215	2092	7702	7569	7706	7705	7709
6854	2215	2092	7702	7569	7706	7705	7709
7667	7615	7614	3379	3501	3367	3366	3370
6844	1835	1909	7776	7694	7784	7783	7787
6825	1823	1884	7762	7680	7770	7769	7773
6708	1750	1811	7638	7570	7644	7643	7647
6780	3528	3611	7591	7502	7595	7594	7598
6843	3572	3648	7612	7539	7616	7615	7619
7653	7614	7605	3304	3428	3292	3291	3295
6754	1822	1786	7690	7556	7696	7695	7699
6725	1933	2059	7686	7580	7692	7691	7695
6832	1933	117	7677	7542	7683	7682	7686
6785	1942	1926	7710	7618	7716	7715	7719
6819	1843	1833	7671	7545	7678	7677	7681
6817	1841	1831	7669	7543	7676	7675	7679
6845	1872	1862	7698	7572	7705	7704	7708
449	6698	6833	7962	7906	7963	7962	7966
6785	3558	3673	7559	7481	7563	7562	7566
6773	1928	1912	7698	7606	7704	7703	7707
6843	2356	2119	7766	7676	7772	7771	7775
7615	7603	7594	3235	3365	3233	3232	3236
7636	7692	7687	3301	3435	3301	3300	3304
6817	3545	3604	7616	7536	7620	7619	7623
6746	1799	1790	7672	7570	7678	7677	7681
7853	7692	7659	3295	3416	3295	3294	3298
6851	2196	2085	7711	7578	7715	7714	7718
6857	2202	2091	7717	7584	7721	7720	7724
6774	1818	1758	7676	7542	7682	7681	7685
6843	1914	1960	7794	7714	7802	7801	7805
533	6729	6842	7934	7901	7940	7939	7943
6781	1876	573	7696	7553	7702	7701	7705
287	6650	6777	7893	7861	7898	7897	7901
6825	1856	1924	7759	7677	7767	7766	7770

6822	1853	1921	7756	7674	7764	7763	7767
6842	2316	2074	7787	7697	7793	7792	7796
6755	1853	1808	7689	7561	7695	7694	7698
7651	7612	7603	3302	3426	3290	3289	3293
7652	7613	7604	3303	3427	3291	3290	3294
7848	7689	7656	3292	3413	3292	3291	3295
6683	139	1924	7715	7609	7720	7719	7723

AM_LREC-109	ERS1724554	EDZFRVQ5	ME160633	ME160685	1508493	ERS1801995
7606	3685	7605	3803	7615	3768	3748
7684	2213	7589	1959	7674	1893	1813
7674	2147	7600	2162	7666	2113	1905
7860	3829	7754	4030	7776	3836	3921
7570	3591	7542	3734	7567	3681	3671
25535	24978	25498	25029	25549	24972	25042
7695	2082	7606	2013	7665	2071	1850
7717	2367	7612	2135	7676	2202	1848
7696	2445	7607	2147	7673	2246	1925
25534	24977	25497	25028	25548	24971	25041
7760	2341	7648	544	7728	2161	33
7839	1778	7766	2491	7792	1895	2266
3301	7712	5	7679	2304	7639	7650
7638	2317	7512	2091	7585	2181	1829
7683	2015	7617	2146	7679	2052	1902
8037	6962	7860	6934	7856	6975	6975
7646	2161	7568	1952	7615	1900	1690
7791	237	7716	2464	7749	2006	2347
7791	237	7716	2464	7749	2006	2347
7747	2205	7644	2137	7709	2262	1878
7870	3655	7771	3729	7825	3799	3655
7706	2056	7632	2365	7679	47	2191
3387	7812	2558	7814	2601	7733	7772
7666	2075	7579	2005	7645	2089	1736
7796	2343	7680	337	7761	2218	316
7758	2397	7649	594	7729	2231	115
3307	7725	2383	7727	803	7634	7692
7676	2355	7634	2105	7679	2148	1895
3290	7790	2516	7796	2449	7706	7743
3290	7790	2516	7796	2449	7706	7743
7641	2144	7583	2067	7611	2115	1832
31163	30968	31226	31016	31203	30944	31010
21	7795	3303	7816	3280	7716	7763
1904	7885	3474	7933	3399	7801	7869
7616	3634	7582	3706	7630	3689	3645
7616	3634	7582	3706	7630	3689	3645
3378	7728	2627	7717	2581	7663	7695
2014	7859	3444	7890	3308	7789	7843
7761	2453	7639	733	7715	2302	254
7796	2329	7676	331	7757	2210	314
7707	2057	7633	2366	7680	48	2192
7568	3725	7538	3878	7568	3764	3784
774	7705	3443	7738	3474	7629	7681
7926	6827	7750	6775	7730	6846	6819
7913	6809	7725	6753	7725	6843	6797
7586	3656	7545	3728	7583	3695	3675
7749	2507	7617	778	7693	2379	317
7942	6839	7754	6783	7754	6873	6827
7887	2595	7769	2341	7838	2446	2103
845	7709	3503	7738	3498	7633	7681
7941	6787	7755	6751	7749	6821	6793
259	7703	3426	7738	3415	7583	7681
7712	2062	7636	2370	7685	53	2197
7734	2475	7574	2142	7676	2358	1976

7714	2383	7610	2142	7669	2214	1855
7663	2333	7528	2057	7599	2187	1793
7687	2140	7614	1956	7658	1926	1709
7694	2292	7635	2082	7686	2100	1874
1881	7847	3465	7900	3383	7766	7832
3407	7708	2653	7691	2610	7637	7669
7688	2124	7583	2127	7659	2162	1895
7711	2366	7615	2138	7677	2199	1853
7687	2376	7568	2182	7630	2247	1897
7714	2328	7623	2137	7684	2184	1852
7690	2124	7585	2127	7661	2162	1895
7924	6814	7738	6738	7736	6848	6782
183	7758	3360	7785	3348	7683	7734
7705	2303	7646	2093	7697	2109	1885
7707	2053	7631	2362	7680	44	2188
256	7702	3423	7737	3412	7582	7680
257	7703	3424	7738	3413	7583	7681
7838	1817	7759	2470	7791	1948	2263
7684	2213	7589	1959	7674	1893	1813
7703	2314	7646	2102	7699	2128	1888
3280	7713	2343	7725	635	7630	7682
7712	2046	7634	2371	7681	63	2201
7634	2601	7554	984	7611	2438	496
1888	7852	3472	7905	3391	7771	7837
3287	7787	2516	7793	2448	7703	7740
7799	6864	7652	6788	7617	6859	6831
7711	2362	7613	2117	7673	2197	1826
7688	2136	7604	2141	7668	2092	1875
23	7798	3303	7819	3282	7719	7766
260	7706	3427	7741	3416	7586	7684
83	7804	3291	7827	3282	7723	7774
82	7803	3290	7826	3281	7722	7773
86	7807	3294	7830	3285	7726	7777
	7795	3302	7816	3279	7716	7763
7795		7713	2483	7748	2063	2352
3302	7713		7680	2305	7640	7651
7816	2483	7680		7774	2346	553
3279	7748	2305	7774		7687	7731
7716	2063	7640	2346	7687		2172
7763	2352	7651	553	7731	2172	
7772	2415	7641	646	7721	2251	141
7799	2350	7691	483	7766	2203	288
7686	2129	7604	2138	7670	2087	1886
7807	2355	7695	445	7770	2210	250
86	7807	3294	7830	3285	7726	7777
7943	6723	7768	6691	7778	6767	6733
7913	6807	7727	6751	7727	6839	6795
7755	2363	7644	568	7724	2185	69
7598	3657	7570	3790	7596	3719	3733
7648	2330	7522	2104	7594	2194	1842
7594	3613	7580	3746	7619	3714	3701
19	7793	3301	7814	3278	7714	7761
7611	3716	7591	3860	7611	3766	3803
8032	6957	7855	6929	7851	6970	6970
7745	2269	7634	692	7711	2087	279

20	7801	3302	7821	3279	7720	7768
7803	2350	7693	440	7768	2205	245
7775	2368	7663	528	7735	2233	327
7555	3708	7536	3848	7558	3758	3789
8021	6928	7833	6888	7833	6941	6929
259	7703	3426	7738	3413	7583	7681
89	7810	3297	7833	3288	7729	7780
3284	7720	2345	7730	625	7639	7687
7802	2334	7680	334	7759	2203	327
7702	2064	7590	1992	7678	2081	1729
1881	7847	3465	7900	3383	7766	7832
7767	1407	7697	2321	7726	1682	2148
7802	2350	7684	414	7761	2207	261
7784	2424	7655	655	7735	2260	150
7940	6720	7765	6688	7775	6764	6730
7579	3701	7570	3833	7586	3741	3777
3281	7750	2307	7776	18	7689	7733
7774	2417	7643	648	7723	2253	143
7709	2005	7623	2376	7676	120	2188
7690	2136	7606	2141	7670	2092	1875
7678	2131	7605	1947	7649	1917	1700
7796	2329	7676	331	7757	2210	314
7684	2329	7633	2097	7686	2147	1918
7675	2112	7587	2141	7647	2082	1915
7917	6815	7731	6759	7731	6849	6803
3286	7754	2312	7780	23	7693	7737
259	7705	3426	7740	3415	7585	7683
7941	6721	7766	6689	7776	6765	6731
7906	6792	7716	6754	7716	6824	6798
7942	6722	7767	6690	7777	6766	6732
7555	3624	7543	3751	7560	3686	3691
7831	2501	7695	44	7789	2364	569
7798	2325	7686	415	7763	2180	224
7747	459	7652	2325	7702	1908	2156
7758	774	7705	2442	7724	1950	2295
7652	3772	7617	3928	7641	3851	3867
7652	3772	7617	3928	7641	3851	3867
3277	7710	2340	7722	632	7627	7679
7716	2365	7613	2128	7675	2193	1831
7601	3745	7594	3876	7603	3815	3816
7662	2078	7565	2095	7630	2100	1845
7583	3679	7578	3808	7603	3741	3748
7756	2354	7647	575	7731	2186	64
7623	3739	7607	3839	7634	3792	3808
7741	2485	7631	742	7708	2295	250
7921	6820	7745	6768	7725	6837	6812
7651	2329	7525	2103	7597	2193	1841
7935	6833	7749	6775	7749	6871	6819
7686	2161	7611	1938	7654	1912	1684
7572	3620	7559	3776	7581	3699	3707
7687	2140	7614	1956	7658	1926	1709
7557	3595	7537	3731	7558	3633	3678
258	7704	3425	7739	3414	7584	7682
258	7704	3425	7739	3414	7584	7682
259	7705	3426	7740	3415	7585	7683

257	7703	3424	7738	3413	7583	7681
7920	6817	7734	6761	7732	6857	6805
7699	2066	7643	2372	7660	164	2198
7740	2498	7608	769	7684	2370	308
7688	2134	7604	2139	7668	2090	1873
3301	7714	40	7681	2303	7641	7652
7767	2469	7643	751	7719	2320	264
7691	2019	7585	2051	7665	2088	1794
7690	2184	7618	1993	7658	1931	1733
7930	6787	7746	6745	7742	6817	6785
7587	3688	7582	3838	7606	3746	3778
259	7704	3426	7739	3415	7584	7682
257	7703	3424	7738	3413	7583	7681
236	7682	3382	7662	3371	7573	7599
260	7705	3427	7740	3416	7585	7683
260	7705	3427	7740	3416	7585	7683
7716	2045	7644	2371	7692	135	2193
7733	2491	7601	762	7677	2363	301
7805	2474	7671	73	7765	2327	546
7715	2358	7612	2138	7677	2193	1855
7700	2352	7598	2112	7661	2181	1827
7689	2181	7615	1988	7653	1926	1728
7692	2185	7619	1994	7659	1932	1734
7707	2057	7633	2366	7680	48	2192
7703	2301	7644	2091	7695	2109	1883
7695	2293	7636	2083	7687	2101	1875
7666	2460	7567	2254	7624	2299	1981
7760	2466	7638	756	7712	2321	269
7636	2143	7580	2066	7608	2114	1831
7686	2001	7575	2039	7655	2054	1768
7661	2136	7585	1927	7633	1870	1673
7596	3689	7555	3826	7580	3748	3773
7920	6821	7744	6769	7724	6840	6813
7569	3616	7557	3774	7578	3696	3705
7793	2327	7673	329	7754	2208	312
3376	7726	2625	7715	2579	7661	7693
7569	3616	7557	3774	7578	3696	3705
7780	2373	7668	586	7749	2195	89
7555	3624	7545	3751	7562	3688	3691
7745	2269	7634	692	7711	2087	279
7735	2353	7633	557	7711	2176	53
7782	2346	7672	494	7751	2221	285
7635	2138	7577	2061	7605	2109	1826
8044	6936	7870	6897	7863	6960	6943
7821	2499	7687	46	7779	2360	573
7586	3636	7564	3797	7583	3716	3724
7764	1638	7689	2244	7728	1596	2055
7859	3644	7760	3718	7812	3788	3644
7676	2150	7601	1927	7646	1901	1673
7682	2185	7586	1951	7670	1889	1805
3377	7727	2626	7716	2580	7662	7694
7759	2392	7644	681	7718	2232	265
7801	2311	7684	339	7765	2184	280
3240	7695	2313	7711	650	7619	7666
7604	3717	7579	3858	7598	3789	3797

7608	3637	7596	3763	7616	3700	3712
7703	2054	7591	1986	7679	2071	1727
7751	2456	7627	738	7703	2307	251
3375	7725	2624	7714	2578	7660	7692
7575	3666	7551	3805	7579	3722	3746
7575	3666	7551	3805	7579	3722	3746
3231	7686	2304	7702	641	7610	7657
7694	2292	7635	2082	7686	2100	1874
7688	2003	7577	2043	7657	2056	1772
7625	3642	7588	3712	7638	3694	3639
7548	3704	7529	3851	7560	3762	3793
7653	2128	7577	1919	7625	1862	1665
7640	2143	7582	2066	7610	2114	1831
7770	2327	7661	359	7740	2194	301
7797	156	7718	2449	7756	2049	2318
18	7793	3298	7814	3277	7714	7761
7772	2408	7645	479	7716	2264	406
3375	7725	2624	7714	2578	7660	7692
3374	7724	2623	7713	2577	7659	7691
7705	2055	7631	2364	7678	46	2190
7672	2073	7569	2094	7647	2114	1825
7912	6794	7740	6756	7736	6828	6797
7699	2066	7643	2372	7660	164	2198
7699	2066	7643	2372	7660	164	2198
3376	7726	2625	7715	2579	7661	7693
7773	2368	7661	577	7742	2188	80
7759	2354	7650	575	7732	2186	64
7635	2138	7577	2061	7605	2109	1826
7587	3681	7582	3810	7607	3744	3750
7608	3714	7584	3855	7602	3785	3794
3303	7714	7	7681	2306	7641	7652
7687	2179	7613	1986	7651	1924	1726
7683	2225	7590	1977	7677	1913	1831
7674	2111	7586	2140	7646	2081	1914
7707	2307	7650	2097	7699	2115	1889
7668	2077	7581	2007	7647	2091	1738
7666	2075	7579	2005	7645	2089	1736
7695	2106	7607	2036	7674	2120	1767
7961	6816	7764	6794	7755	6847	6838
7555	3695	7545	3831	7573	3781	3752
7695	2293	7636	2083	7687	2100	1875
7763	353	7691	2425	7714	1939	2292
3232	7687	2305	7703	642	7611	7658
3298	7767	2324	7793	35	7706	7750
7612	3635	7598	3769	7626	3711	3708
7669	2182	7606	1895	7649	1927	1639
3292	7792	2518	7798	2451	7708	7745
7708	2058	7634	2367	7681	49	2193
7714	2064	7640	2373	7687	55	2199
7673	2153	7588	1952	7642	1887	1698
7791	2345	7675	411	7752	2204	256
7931	6859	7751	6801	7747	6885	6845
7693	2088	7587	2113	7668	2129	1836
7890	6796	7703	6737	7686	6811	6781
7756	2395	7647	592	7727	2229	113

7753	2392	7644	589	7724	2226	110
7784	214	7698	2443	7741	2029	2284
7686	2161	7611	1938	7654	1912	1684
3301	7712	3	7679	2304	7639	7650
3302	7713	4	7680	2305	7640	7651
3289	7789	2515	7795	2448	7705	7742
7712	2365	7614	2137	7676	2198	1852

ERS1812824	20151201	MT66C.C1	DTU2017-812-PR	DTU2017-821-PRJ1111	PA20B	1351
3765	3787	3591	3787	7617	6649	6806
1887	1834	2040	1847	7696	6614	6680
1964	1941	40	1962	7686	6735	6817
3899	3895	3797	3902	7870	6930	7026
3680	3704	3523	3710	7581	6600	6759
25037	25059	24979	25061	25553	25061	25017
1929	1821	1799	1841	7709	6660	6718
1887	1911	1937	1930	7728	6610	6688
1990	2001	1947	2000	7709	6722	6798
25036	25058	24978	25060	25552	25060	25016
140	285	1874	247	7774	6728	6790
2338	2346	2225	2359	7851	6775	6873
7640	7690	7603	7694	3293	7767	7726
1887	1968	1833	1963	7648	6615	6677
1961	1987	1962	1998	7695	6727	6801
6984	7015	6940	7015	8049	571	391
1757	1746	1766	1758	7658	6650	6726
2425	2337	2108	2342	7803	6712	6796
2425	2337	2108	2342	7803	6712	6796
1941	1929	1887	1964	7761	6764	6838
3694	3627	3286	3652	7879	6898	6916
2261	2222	2078	2229	7716	6757	6829
7760	7807	7700	7809	3407	7819	7788
1796	1850	1836	1852	7679	6714	6798
393	226	1995	188	7810	6757	6817
140	338	1925	300	7772	6731	6793
7680	7725	7622	7729	3313	7729	7678
1964	1954	1964	1954	7688	6709	6779
7728	7795	7660	7799	3296	7986	7949
7728	7795	7660	7799	3296	7986	7949
1895	1819	1822	1842	7653	6594	6686
30996	31016	31002	31018	31182	30821	30797
7772	7799	7686	7807	85	7943	7913
7876	7914	7789	7914	1932	8015	7989
3657	3686	3592	3676	7629	6642	6799
3657	3686	3592	3676	7629	6642	6799
7682	7744	7613	7746	3372	7789	7764
7844	7884	7769	7884	2030	7949	7925
153	450	1993	430	7775	6722	6784
387	227	1987	189	7810	6751	6811
2262	2223	2079	2230	7717	6758	6830
3810	3780	3646	3771	7579	6660	6811
7692	7722	7602	7726	787	7887	7833
6828	6848	6805	6859	7935	392	187
6806	6839	6801	6837	7922	221	16
3686	3698	3526	3707	7597	6596	6755
398	535	2060	499	7763	6675	6737
6836	6869	6831	6867	7951	251	46
2167	2222	2032	2217	7897	6879	6953
7692	7722	7612	7726	854	7889	7829
6808	6841	6785	6839	7951	217	98
7690	7704	7551	7727	289	7906	7874
2267	2228	2084	2235	7722	6763	6835
2035	2113	2046	2108	7744	6718	6792

1917	1936	1948	1951	7725	6626	6704
1855	1956	1877	1951	7673	6641	6715
1776	1756	1754	1767	7699	6694	6770
1943	1914	1916	1933	7706	6659	6729
7843	7872	7746	7876	1914	7968	7942
7656	7718	7587	7720	3400	7769	7788
1956	1977	327	1974	7700	6704	6786
1911	1914	1912	1943	7722	6592	6670
1955	1962	1952	1987	7698	6579	6657
1910	1923	1937	1942	7725	6588	6666
1956	1977	327	1974	7702	6706	6788
6793	6824	6806	6822	7933	283	90
7743	7770	7649	7778	111	7953	7924
1954	1925	1923	1944	7717	6668	6738
2258	2219	2075	2226	7717	6760	6832
7689	7703	7550	7726	286	7905	7873
7690	7704	7551	7727	287	7906	7874
2335	2327	2260	2340	7850	6786	6884
1887	1834	2040	1847	7696	6614	6680
1957	1934	1942	1953	7715	6644	6714
7670	7715	7623	7719	3280	7747	7693
2271	2232	2094	2239	7722	6769	6841
569	659	2112	658	7688	6860	6754
7848	7877	7751	7881	1921	7975	7949
7725	7792	7657	7796	3293	7981	7944
6844	6844	6816	6855	7808	664	459
1884	1892	1903	1922	7722	6613	6691
1954	1949	21	1970	7700	6730	6812
7775	7802	7689	7810	87	7946	7916
7693	7707	7554	7730	290	7909	7877
7783	7810	7695	7818	15	7954	7919
7782	7809	7694	7817	8	7953	7918
7786	7813	7698	7821	14	7957	7922
7772	7799	7686	7807	86	7943	7913
2415	2350	2129	2355	7807	6723	6807
7641	7691	7604	7695	3294	7768	7727
646	483	2138	445	7830	6691	6751
7721	7766	7670	7770	3285	7778	7727
2251	2203	2087	2210	7726	6767	6839
141	288	1886	250	7777	6733	6795
	367	1955	329	7786	6742	6804
367		1946	86	7813	6775	6837
1955	1946		1967	7698	6723	6805
329	86	1967		7821	6773	6835
7786	7813	7698	7821		7957	7922
6742	6775	6723	6773	7957		225
6804	6837	6805	6835	7922	225	
136	301	1892	263	7769	6728	6790
3736	3768	3569	3776	7609	6616	6775
1900	1981	1844	1976	7658	6626	6688
3712	3709	3568	3718	7607	6648	6801
7770	7797	7684	7805	83	7942	7912
3802	3836	3660	3844	7622	6658	6817
6979	7010	6935	7010	8044	566	386
370	503	1801	465	7757	6700	6770

7777	7804	7690	7812	94	7946	7916
324	69	1962	43	7817	6767	6829
410	253	1992	215	7789	6744	6806
3773	3816	3633	3824	7566	6625	6784
6938	6959	6899	6969	8032	553	381
7690	7704	7551	7727	289	7908	7876
7789	7816	7701	7824	17	7960	7925
7675	7720	7630	7724	3288	7752	7701
410	241	1988	203	7816	6749	6809
1791	1837	638	1812	7713	6691	6771
7843	7872	7746	7876	1914	7968	7942
2208	2197	2121	2197	7779	6736	6806
344	167	1968	129	7816	6754	6816
43	376	1967	338	7798	6751	6813
6739	6772	6720	6770	7954	5	222
3800	3769	3632	3768	7590	6631	6790
7723	7768	7672	7772	3287	7780	7729
18	369	1957	331	7788	6744	6806
2258	2233	2089	2240	7719	6756	6828
1954	1949	21	1970	7702	6730	6812
1767	1747	1745	1758	7690	6685	6761
387	227	1987	189	7810	6751	6811
1975	1928	1953	1947	7696	6673	6743
1984	1953	111	1974	7687	6740	6822
6812	6845	6807	6843	7926	227	22
7727	7772	7676	7776	3292	7785	7734
7692	7706	7553	7729	289	7908	7876
6740	6773	6721	6771	7955	6	223
6807	6840	6788	6838	7913	224	33
6741	6774	6722	6772	7956	7	224
3683	3702	3550	3710	7566	6606	6765
664	497	2156	459	7845	6708	6768
307	142	1937	104	7812	6740	6802
2228	2186	1959	2191	7759	6737	6805
2373	2307	2177	2312	7772	6696	6770
3878	3896	3718	3902	7663	6661	6820
3878	3896	3718	3902	7663	6661	6820
7667	7712	7620	7716	3277	7744	7690
1901	1916	1920	1935	7727	6605	6683
3829	3831	3675	3837	7612	6663	6822
1898	1925	468	1938	7674	6699	6783
3736	3761	3608	3769	7594	6643	6802
135	299	1888	261	7770	6724	6786
3818	3815	3699	3823	7634	6631	6790
145	437	2009	411	7755	6664	6726
6821	6841	6798	6852	7930	385	186
1899	1980	1845	1975	7661	6627	6689
6828	6861	6823	6859	7944	243	50
1751	1729	1813	1752	7698	6662	6738
3723	3751	3583	3752	7583	6602	6761
1776	1756	1754	1767	7699	6694	6770
3701	3717	3539	3717	7568	6599	6758
7691	7705	7552	7728	288	7907	7875
7691	7705	7552	7728	288	7907	7875
7692	7706	7553	7729	289	7908	7876

7690	7704	7551	7727	287	7906	7874
6814	6847	6809	6845	7929	227	34
2268	2229	2087	2236	7709	6762	6834
389	526	2051	490	7754	6666	6728
1952	1947	19	1968	7700	6728	6810
7642	7692	7605	7696	3293	7770	7728
149	454	2007	434	7781	6731	6793
1847	1898	442	1895	7703	6677	6761
1800	1798	1796	1811	7702	6655	6739
6798	6827	6777	6825	7940	213	94
3766	3792	3628	3797	7598	6654	6813
7691	7705	7552	7728	289	7907	7875
7690	7704	7551	7727	287	7906	7874
7608	7645	7569	7653	266	7898	7866
7692	7706	7553	7729	290	7908	7876
7692	7706	7553	7729	290	7908	7876
2253	2216	2148	2223	7726	6743	6815
382	519	2044	483	7747	6659	6721
641	474	2101	436	7819	6692	6752
1912	1930	1928	1949	7726	6600	6678
1869	1898	1901	1917	7711	6596	6674
1795	1793	1793	1806	7701	6650	6734
1801	1799	1797	1812	7704	6656	6740
2262	2223	2079	2230	7717	6758	6830
1952	1923	1923	1942	7715	6668	6738
1944	1915	1915	1934	7707	6660	6730
2034	2036	2044	2063	7678	6601	6679
154	459	2004	439	7774	6720	6782
1894	1818	1821	1841	7648	6593	6685
1821	1886	478	1883	7698	6676	6760
1740	1718	1742	1731	7673	6660	6736
3793	3804	3629	3812	7607	6600	6759
6822	6842	6799	6853	7929	386	181
3721	3749	3580	3750	7580	6601	6760
385	225	1985	187	7807	6748	6808
7680	7742	7611	7744	3370	7787	7762
3721	3749	3580	3750	7580	6601	6760
162	329	1911	291	7794	6748	6810
3683	3702	3550	3710	7566	6610	6769
370	503	1801	465	7757	6700	6770
140	273	1890	235	7749	6727	6789
356	125	1948	99	7796	6751	6813
1889	1813	1816	1836	7647	6588	6680
6952	6981	6930	6981	8053	576	371
666	489	2146	465	7835	6698	6758
3729	3751	3570	3757	7597	6589	6748
2127	2130	2031	2130	7776	6736	6806
3683	3616	3275	3641	7868	6888	6906
1740	1718	1802	1741	7688	6651	6727
1879	1826	2044	1839	7694	6610	6676
7681	7743	7612	7745	3371	7788	7763
346	479	1901	443	7773	6716	6778
365	198	1961	160	7815	6755	6815
7656	7701	7602	7705	3244	7780	7729
3806	3826	3649	3832	7615	6637	6796

3721	3755	3568	3763	7619	6615	6774
1797	1841	628	1816	7714	6689	6769
130	441	1994	421	7765	6715	6777
7679	7741	7610	7743	3369	7786	7761
3750	3787	3591	3795	7586	6617	6776
3750	3787	3591	3795	7586	6617	6776
7647	7692	7593	7696	3235	7771	7720
1943	1914	1914	1933	7706	6659	6729
1825	1890	480	1887	7700	6679	6763
3661	3698	3602	3688	7638	6648	6805
3779	3804	3655	3812	7559	6629	6788
1732	1710	1734	1723	7665	6652	6728
1894	1818	1821	1841	7652	6593	6685
382	197	1953	159	7784	6755	6815
2380	2316	2109	2321	7809	6722	6804
7770	7797	7684	7805	78	7941	7911
505	342	2056	304	7779	6807	6805
7679	7741	7610	7743	3369	7786	7761
7678	7740	7609	7742	3368	7785	7760
2260	2221	2077	2228	7715	6756	6828
1875	1926	561	1942	7684	6664	6748
6808	6825	6790	6823	7922	224	105
2268	2229	2087	2236	7709	6762	6834
2268	2229	2087	2236	7709	6762	6834
7680	7742	7611	7744	3370	7787	7762
153	320	1904	282	7787	6741	6803
137	299	1889	261	7773	6725	6787
1889	1813	1816	1836	7647	6588	6680
3738	3763	3611	3771	7598	6645	6804
3805	3823	3647	3829	7619	6649	6808
7642	7692	7605	7696	3295	7769	7728
1793	1791	1791	1804	7699	6647	6731
1893	1838	2054	1851	7695	6610	6676
1983	1952	110	1973	7686	6739	6821
1958	1929	1929	1948	7719	6674	6744
1798	1852	1838	1854	7681	6716	6800
1796	1850	1836	1852	7679	6714	6798
1827	1881	1867	1883	7708	6742	6826
6847	6876	6826	6878	7966	424	223
3762	3797	3671	3787	7566	6626	6785
1944	1915	1915	1934	7707	6660	6730
2372	2300	2124	2305	7775	6705	6795
7648	7693	7594	7697	3236	7772	7721
7740	7785	7689	7789	3304	7797	7746
3719	3759	3611	3759	7623	6631	6790
1706	1710	1795	1699	7681	6648	6724
7730	7797	7662	7801	3298	7988	7951
2263	2224	2080	2231	7718	6759	6831
2269	2230	2086	2237	7724	6765	6837
1765	1743	1763	1756	7685	6675	6751
335	160	1959	122	7805	6745	6807
6854	6887	6835	6885	7943	274	90
1885	1941	576	1957	7705	6684	6768
6790	6810	6770	6821	7901	493	288
138	336	1923	298	7770	6729	6791

135	333	1920	295	7767	6726	6788
2356	2304	2079	2309	7796	6722	6790
1751	1729	1813	1752	7698	6662	6738
7640	7690	7603	7694	3293	7767	7726
7641	7691	7604	7695	3294	7768	7727
7727	7794	7659	7798	3295	7983	7946
1910	1913	1911	1942	7723	6591	6669

2011-70-34-3	2011-70-41-2	DTU2011_26	2011-70-219-2	HVH	ESC0167	ESC0211	ESC0198
3736	859	3525	729	7605	1040	6974	3759
1809	3549	1888	3603	7682	3630	6838	1769
1907	3589	1862	3590	7672	3675	6947	1831
3904	3376	3705	3410	7859	3515	7171	3935
3651	378	3457	852	7569	536	6929	3672
25033	24918	24941	24932	25532	24931	25052	25029
1869	3562	1919	3535	7693	3638	6861	1803
1844	3536	1825	3516	7715	3628	6854	1814
1933	3598	1775	3571	7694	3641	6927	1856
25032	24917	24940	24931	25531	24930	25051	25028
64	3725	1832	3693	7758	3801	6965	266
2255	3707	2373	3706	7837	3739	7006	2208
7643	7569	7521	7579	3300	7590	7854	7633
1839	3488	33	3517	7636	3562	6831	1793
1893	3516	1905	3549	7681	3632	6955	1895
6970	6972	6847	6972	8036	7002	23	6929
1707	3551	1919	3527	7644	3623	6875	1635
2336	3672	2323	3625	7789	3714	6946	2278
2336	3672	2323	3625	7789	3714	6946	2278
1893	3617	2058	3622	7745	3719	7008	1887
3664	4186	3475	4155	7868	4234	7058	3610
2184	3706	2193	3708	7704	3750	6960	2102
7764	7599	7637	7647	3384	7636	7892	7748
1747	3560	1806	3556	7664	3642	6939	1719
323	3729	1970	3672	7794	3790	6992	497
107	3739	1878	3694	7756	3800	6970	342
7685	7529	7550	7564	3306	7550	7799	7677
1912	3568	1913	3592	7674	3647	6891	1816
7735	7666	7627	7694	3289	7683	8065	7732
7735	7666	7627	7694	3289	7683	8065	7732
1847	3401	1910	3403	7639	3528	6828	1831
31007	30939	30914	30928	31161	30954	30788	31008
7755	7599	7648	7595	8	7612	8032	7745
7859	7731	7728	7759	1903	7753	8095	7848
3627	1184	3493	548	7615	1082	6967	3653
3627	1184	3493	548	7615	1082	6967	3653
7687	7534	7561	7569	3377	7554	7878	7674
7835	7663	7707	7686	2013	7690	8045	7826
247	3727	1944	3704	7759	3784	6981	475
313	3719	1956	3666	7794	3782	6984	491
2185	3707	2194	3709	7705	3751	6961	2103
3794	676	3552	1092	7567	803	6972	3780
7673	7502	7559	7511	773	7446	7993	7660
6814	6755	6696	6781	7925	6813	429	6794
6792	6769	6686	6795	7912	6811	386	6772
3651	702	3446	957	7585	625	6936	3659
328	3841	1988	3792	7747	3848	6893	540
6822	6798	6716	6825	7941	6840	416	6802
2108	3743	824	3770	7885	3827	7087	2035
7673	7500	7555	7511	844	7444	7987	7662
6790	6753	6670	6783	7940	6795	430	6760
7673	7515	7513	7496	260	7521	8004	7661
2190	3711	2199	3713	7710	3755	6966	2108
1970	3670	582	3690	7732	3749	6926	1970

1853	3531	1818	3455	7712	3595	6846	1809
1802	3532	391	3545	7661	3606	6849	1747
1726	3600	1920	3611	7685	3676	6896	1654
1891	3540	1871	3563	7692	3617	6900	1795
7822	7674	7694	7690	1880	7694	8057	7811
7661	7514	7535	7549	3406	7534	7902	7648
1888	3565	1830	3524	7686	3641	6914	1812
1853	3530	1822	3498	7709	3618	6845	1799
1897	3527	1832	3464	7685	3585	6812	1843
1852	3496	1850	3441	7712	3561	6832	1798
1888	3565	1830	3524	7688	3641	6916	1812
6774	6761	6699	6794	7923	6815	424	6767
7726	7552	7615	7538	180	7559	8046	7710
1902	3549	1882	3572	7703	3626	6909	1806
2181	3703	2190	3707	7705	3749	6963	2099
7672	7514	7512	7495	257	7520	8003	7660
7673	7515	7513	7496	258	7521	8004	7661
2250	3707	2385	3696	7836	3739	7017	2203
1809	3549	1888	3603	7682	3630	6838	1769
1905	3546	1885	3573	7701	3629	6909	1809
7675	7545	7546	7572	3279	7568	7802	7665
2194	3714	2201	3716	7710	3758	6972	2112
503	3791	2080	3698	7632	3859	6876	703
7827	7681	7699	7697	1887	7703	8064	7816
7732	7663	7624	7691	3286	7680	8060	7729
6828	6785	6733	6811	7798	6842	697	6811
1826	3509	1784	3482	7709	3589	6841	1772
1891	3576	1851	3577	7686	3661	6942	1806
7758	7602	7651	7598	18	7615	8035	7748
7676	7518	7516	7499	261	7524	8007	7664
7766	7606	7657	7604	80	7619	8041	7754
7765	7605	7656	7603	79	7618	8040	7753
7769	7609	7660	7607	83	7622	8044	7757
7755	7598	7648	7594	19	7611	8032	7745
2363	3657	2330	3613	7793	3716	6957	2269
7644	7570	7522	7580	3301	7591	7855	7634
568	3790	2104	3746	7814	3860	6929	692
7724	7596	7594	7619	3278	7611	7851	7711
2185	3719	2194	3714	7714	3766	6970	2087
69	3733	1842	3701	7761	3803	6970	279
136	3736	1900	3712	7770	3802	6979	370
301	3768	1981	3709	7797	3836	7010	503
1892	3569	1844	3568	7684	3660	6935	1801
263	3776	1976	3718	7805	3844	7010	465
7769	7609	7658	7607	83	7622	8044	7757
6728	6616	6626	6648	7942	6658	566	6700
6790	6775	6688	6801	7912	6817	386	6770
	3715	1852	3692	7753	3780	6965	292
3715		3500	938	7597	499	6967	3732
1852	3500		3530	7646	3574	6842	1806
3692	938	3530		7593	806	6967	3702
7753	7597	7646	7593		7610	8031	7743
3780	499	3574	806	7610		6997	3798
6965	6967	6842	6967	8031	6997		6924
292	3732	1806	3702	7743	3798	6924	

7760	7604	7653	7600	27	7617	8035	7750
258	3771	1971	3713	7801	3839	7004	460
344	3750	1981	3627	7773	3790	6979	538
3777	394	3535	971	7554	484	6964	3798
6924	6933	6816	6926	8020	6951	137	6883
7673	7515	7513	7496	260	7521	8006	7661
7772	7612	7663	7610	86	7625	8047	7760
7680	7552	7553	7579	3283	7575	7810	7670
340	3721	1957	3649	7800	3789	6984	498
1727	3598	1772	3575	7700	3663	6911	1616
7822	7674	7694	7690	1880	7694	8057	7811
2164	3747	2121	3682	7765	3784	6950	2091
268	3726	1975	3673	7800	3785	6991	476
148	3748	1909	3724	7782	3814	6988	379
6725	6613	6623	6645	7939	6655	563	6697
3775	499	3559	901	7578	633	6925	3775
7726	7598	7596	7621	3280	7613	7853	7713
138	3738	1902	3714	7772	3804	6981	372
2181	3685	2142	3681	7707	3751	6961	2099
1891	3576	1851	3577	7688	3661	6942	1806
1717	3591	1911	3602	7676	3667	6887	1645
313	3719	1956	3666	7794	3782	6984	491
1929	3543	1910	3556	7682	3619	6914	1842
1919	3548	1871	3551	7673	3639	6950	1816
6798	6775	6692	6801	7916	6817	392	6778
7730	7602	7600	7624	3285	7617	7858	7717
7675	7517	7515	7498	260	7523	8006	7663
6726	6614	6624	6646	7940	6656	564	6698
6793	6760	6669	6782	7905	6802	387	6769
6727	6615	6625	6647	7941	6657	565	6699
3684	254	3462	859	7554	493	6967	3697
584	3806	2122	3758	7829	3872	6946	710
241	3750	1944	3688	7796	3820	6977	435
2145	3631	2181	3580	7745	3671	6955	2107
2284	3763	2383	3719	7756	3807	6908	2214
3843	600	3649	986	7651	452	7004	3868
3843	600	3649	986	7651	452	7004	3868
7672	7542	7543	7569	3276	7565	7799	7662
1839	3532	1818	3516	7714	3622	6849	1789
3798	526	3591	973	7600	655	6983	3822
1843	3547	1868	3541	7660	3628	6915	1748
3742	320	3501	871	7582	501	7004	3754
75	3737	1842	3703	7754	3801	6961	287
3782	1281	3636	812	7622	1179	6990	3793
239	3781	1998	3740	7739	3811	6931	474
6807	6754	6689	6778	7920	6812	426	6787
1851	3498	45	3529	7649	3572	6843	1805
6814	6803	6716	6829	7934	6845	384	6794
1701	3570	1942	3577	7684	3646	6876	1627
3701	865	3523	313	7571	767	6954	3704
1726	3600	1920	3611	7685	3676	6896	1654
3666	469	3473	722	7556	395	6940	3677
7674	7516	7514	7497	259	7522	8005	7662
7674	7516	7514	7497	259	7522	8005	7662
7675	7517	7515	7498	260	7523	8006	7663

7673	7515	7513	7496	258	7521	8004	7661
6800	6789	6702	6815	7919	6831	368	6780
2191	3645	2229	3709	7697	3751	6965	2109
319	3832	1979	3783	7738	3839	6884	531
1889	3574	1849	3575	7686	3659	6940	1804
7645	7571	7523	7581	3300	7592	7856	7635
260	3740	1960	3722	7765	3798	6990	493
1802	3559	1789	3532	7689	3644	6889	1688
1750	3537	1932	3540	7688	3613	6879	1674
6780	6747	6670	6773	7929	6785	426	6754
3774	374	3516	886	7586	499	6990	3774
7674	7516	7514	7497	260	7522	8005	7662
7673	7515	7513	7496	258	7521	8004	7661
7591	7522	7550	7497	237	7567	7989	7579
7675	7517	7515	7498	261	7523	8006	7663
7675	7517	7515	7498	261	7523	8006	7663
2184	3712	2239	3704	7714	3756	6946	2110
312	3825	1972	3776	7731	3832	6877	524
561	3815	2077	3767	7803	3881	6930	687
1847	3522	1816	3507	7713	3613	6844	1801
1826	3513	1804	3479	7698	3617	6840	1783
1745	3534	1927	3537	7687	3610	6874	1669
1751	3538	1933	3541	7690	3614	6880	1675
2185	3707	2194	3709	7705	3751	6961	2103
1900	3549	1880	3572	7701	3626	6909	1804
1892	3541	1872	3564	7693	3618	6901	1796
1978	3523	1905	3431	7664	3569	6830	1927
265	3745	1957	3727	7758	3787	6979	490
1846	3400	1909	3402	7634	3527	6827	1830
1776	3559	1776	3548	7684	3644	6886	1664
1690	3565	1882	3566	7659	3639	6876	1618
3753	497	3547	859	7595	376	6937	3766
6808	6749	6690	6775	7919	6807	423	6788
3699	862	3521	310	7568	764	6953	3702
311	3717	1954	3664	7791	3780	6981	489
7685	7532	7559	7567	3375	7552	7876	7672
3699	862	3521	310	7568	764	6953	3702
88	3747	1864	3702	7778	3806	6985	304
3684	256	3460	861	7554	495	6971	3697
292	3732	1806	3702	7743	3798	6924	
66	3733	1842	3703	7733	3802	6964	283
296	3773	1979	3697	7780	3817	6992	490
1841	3395	1904	3397	7633	3522	6822	1825
6936	6908	6821	6916	8043	6839	506	6918
588	3792	2119	3745	7819	3862	6936	712
3702	441	3510	931	7585	582	6940	3725
2075	3747	2021	3668	7762	3770	6950	1998
3653	4175	3464	4144	7857	4223	7048	3599
1690	3559	1931	3566	7674	3635	6865	1616
1817	3558	1875	3604	7680	3639	6834	1763
7686	7533	7560	7568	3376	7553	7877	7673
276	3771	1883	3740	7757	3834	6942	450
299	3744	1934	3692	7799	3818	6990	457
7659	7531	7528	7564	3239	7556	7838	7649
3781	462	3575	947	7603	419	6980	3798

3720	314	3460	889	7607	496	6976	3701
1739	3592	1762	3571	7701	3669	6909	1606
246	3726	1947	3703	7749	3781	6974	480
7684	7531	7558	7566	3374	7551	7875	7671
3747	320	3486	890	7574	507	6978	3741
3747	320	3486	890	7574	507	6978	3741
7650	7522	7519	7555	3230	7547	7829	7640
1891	3540	1871	3563	7692	3617	6900	1795
1780	3559	1780	3548	7686	3644	6891	1668
3631	1186	3499	564	7624	1084	6973	3659
3785	533	3565	853	7547	357	6968	3801
1682	3557	1874	3558	7651	3631	6868	1610
1846	3400	1909	3402	7638	3527	6827	1830
316	3754	1932	3698	7768	3823	6990	481
2309	3642	2290	3603	7795	3698	6954	2255
7753	7597	7646	7593	15	7610	8030	7743
433	3800	2024	3757	7770	3869	6943	570
7684	7531	7558	7566	3374	7551	7875	7671
7683	7530	7557	7565	3373	7550	7874	7670
2183	3705	2192	3707	7703	3749	6959	2101
1817	3550	1850	3530	7670	3621	6895	1725
6792	6754	6677	6786	7911	6795	437	6767
2191	3645	2229	3709	7697	3751	6965	2109
2191	3645	2229	3709	7697	3751	6965	2109
7685	7532	7559	7567	3375	7552	7876	7672
79	3740	1857	3695	7771	3799	6978	295
75	3742	1843	3708	7757	3806	6961	287
1841	3395	1904	3397	7633	3522	6822	1825
3744	322	3504	873	7586	503	7006	3756
3770	452	3572	947	7607	413	6992	3795
7645	7571	7523	7581	3302	7592	7856	7635
1743	3532	1925	3535	7685	3608	6869	1667
1825	3563	1902	3607	7681	3644	6834	1791
1918	3547	1870	3550	7672	3638	6949	1815
1906	3555	1886	3578	7705	3632	6915	1810
1749	3562	1808	3558	7666	3644	6941	1721
1747	3560	1806	3556	7664	3642	6939	1719
1778	3591	1837	3587	7693	3673	6967	1750
6831	6782	6693	6794	7958	6828	419	6809
3736	908	3595	548	7554	857	6953	3773
1892	3541	1872	3564	7693	3618	6901	1796
2291	3689	2298	3640	7761	3737	6945	2206
7651	7523	7520	7556	3231	7548	7830	7641
7743	7615	7613	7638	3297	7630	7870	7730
3708	631	3547	800	7611	526	6978	3713
1656	3566	1914	3571	7667	3640	6859	1586
7737	7668	7629	7696	3291	7685	8067	7734
2186	3708	2195	3710	7706	3752	6962	2104
2192	3714	2201	3716	7712	3758	6968	2110
1715	3588	1917	3589	7671	3662	6891	1643
263	3721	1970	3658	7789	3782	6982	471
6840	6845	6744	6871	7930	6887	373	6810
1831	3563	1865	3533	7691	3626	6915	1740
6776	6735	6663	6761	7889	6799	530	6764
105	3737	1876	3690	7754	3798	6968	340

102	3734	1873	3689	7751	3795	6965	337
2273	3679	2280	3630	7782	3721	6940	2229
1701	3570	1942	3577	7684	3646	6876	1627
7643	7569	7521	7579	3300	7590	7854	7633
7644	7568	7522	7580	3301	7589	7855	7634
7734	7665	7626	7693	3288	7682	8062	7731
1852	3529	1821	3497	7710	3617	6844	1798

AZ-TG60445	AZ-TG60412	2-316-03_S3_C3	7-233-03_S3_	2-316-03_S1_C2	2-460-02_S1_C2
7612	3782	3720	904	6938	7531
7688	1842	1888	3584	6805	7578
7678	1957	2006	3642	6911	7539
7866	3897	3867	3399	7141	7767
7576	3705	3707	410	6877	7479
25538	25056	25037	24943	25043	25532
7701	1836	1886	3617	6832	7603
7721	1925	1960	3576	6820	7615
7700	1995	2017	3623	6916	7580
25537	25055	25036	24942	25042	25531
7765	242	320	3789	6924	7678
7843	2354	2382	3747	6969	7698
3301	7692	7662	7535	7832	3425
7643	1958	1968	3523	6805	7503
7687	1993	2023	3587	6924	7549
8040	7009	6984	6969	150	8011
7650	1753	1793	3597	6834	7514
7797	2337	2367	3720	6917	7701
7797	2337	2367	3720	6917	7701
7751	1959	1975	3674	6967	7617
7874	3647	3636	4241	7017	7790
7710	2224	2252	3752	6931	7573
3385	7807	7781	7584	7879	3522
7672	1847	1921	3613	6914	7540
7801	183	283	3771	6951	7718
7763	295	384	3767	6929	7676
3307	7727	7692	7498	7779	3431
7682	1949	1965	3617	6885	7650
3292	7797	7755	7627	8049	3411
3292	7797	7755	7627	8049	3411
7645	1837	1905	3444	6795	7573
31166	31010	31001	30930	30780	31136
29	7803	7775	7556	8021	262
1914	7909	7889	7693	8085	2066
7622	3671	3595	1231	6937	7541
7622	3671	3595	1231	6937	7541
3378	7744	7704	7502	7870	3502
2018	7882	7857	7646	8029	2147
7766	425	428	3782	6924	7681
7801	184	277	3763	6943	7718
7711	2225	2253	3753	6932	7574
7574	3766	3774	629	6932	7481
784	7722	7687	7491	7954	814
7929	6853	6825	6789	425	7894
7916	6831	6808	6778	377	7876
7592	3702	3650	758	6892	7501
7754	494	453	3797	6857	7691
7945	6861	6838	6807	407	7905
7891	2212	2230	3764	7059	7752
855	7722	7687	7489	7947	879
7944	6833	6806	6764	435	7904
269	7723	7679	7448	7967	6
7716	2230	2258	3757	6937	7579
7739	2103	2123	3696	6900	7599

7718	1946	1925	3575	6805	7601
7668	1946	1960	3560	6823	7528
7691	1762	1843	3654	6866	7571
7700	1928	1935	3585	6863	7602
1891	7871	7851	7641	8043	2045
3407	7718	7678	7482	7894	3531
7693	1969	2003	3606	6886	7557
7715	1938	1955	3586	6798	7605
7691	1982	1923	3556	6761	7573
7718	1937	1960	3558	6798	7612
7695	1969	2003	3606	6888	7559
7927	6816	6795	6777	419	7887
191	7774	7746	7508	8034	384
7711	1939	1946	3594	6872	7613
7711	2221	2249	3751	6934	7574
266	7722	7678	7447	7966	3
267	7723	7679	7448	7967	4
7842	2335	2363	3747	6980	7697
7688	1842	1888	3584	6805	7578
7709	1948	1951	3597	6874	7611
3280	7717	7684	7516	7786	3410
7716	2234	2262	3760	6943	7579
7639	653	572	3840	6836	7562
1898	7876	7856	7648	8050	2052
3289	7794	7752	7624	8044	3408
7802	6849	6844	6820	675	7767
7715	1917	1962	3570	6802	7601
7692	1965	1997	3632	6906	7553
31	7806	7778	7559	8024	264
270	7726	7682	7451	7970	7
91	7814	7786	7563	8029	286
90	7813	7785	7562	8028	285
94	7817	7789	7566	8032	289
20	7803	7775	7555	8021	259
7801	2350	2368	3708	6928	7703
3302	7693	7663	7536	7833	3426
7821	440	528	3848	6888	7738
3279	7768	7735	7558	7833	3413
7720	2205	2233	3758	6941	7583
7768	245	327	3789	6929	7681
7777	324	410	3773	6938	7690
7804	69	253	3816	6959	7704
7690	1962	1992	3633	6899	7551
7812	43	215	3824	6969	7727
94	7817	7789	7566	8032	289
7946	6767	6744	6625	553	7908
7916	6829	6806	6784	381	7876
7760	258	344	3777	6924	7673
7604	3771	3750	394	6933	7515
7653	1971	1981	3535	6816	7513
7600	3713	3627	971	6926	7496
27	7801	7773	7554	8020	260
7617	3839	3790	484	6951	7521
8035	7004	6979	6964	137	8006
7750	460	538	3798	6883	7661

	7808	7780	7561	8024	269
7808		210	3819	6963	7723
7780	210		3760	6926	7679
7561	3819	3760		6918	7448
8024	6963	6926	6918		7969
269	7723	7679	7448	7969	
97	7820	7792	7569	8035	292
3284	7722	7689	7523	7794	3414
7807	198	290	3775	6943	7724
7707	1807	1873	3634	6882	7566
1891	7871	7851	7641	8043	2045
7771	2192	2198	3772	6915	7655
7807	124	232	3774	6950	7722
7789	333	419	3796	6947	7702
7943	6764	6741	6622	550	7905
7585	3763	3710	485	6919	7491
3281	7770	7737	7560	7835	3415
7779	326	412	3775	6940	7692
7713	2235	2245	3749	6930	7570
7694	1965	1997	3632	6906	7555
7682	1753	1834	3645	6857	7562
7801	184	277	3763	6943	7718
7690	1942	1962	3581	6877	7592
7679	1969	1999	3610	6914	7540
7920	6837	6814	6784	383	7880
3286	7774	7741	7564	7840	3420
269	7725	7681	7450	7969	6
7944	6765	6742	6623	551	7906
7909	6832	6809	6769	390	7869
7945	6766	6743	6624	552	7907
7561	3705	3696	351	6931	7468
7836	454	546	3866	6905	7753
7803	99	165	3802	6936	7718
7753	2186	2218	3669	6926	7657
7762	2307	2337	3803	6877	7670
7658	3897	3864	618	6962	7580
7658	3897	3864	618	6962	7580
3277	7714	7681	7513	7783	3407
7720	1930	1961	3592	6815	7614
7605	3832	3759	546	6950	7512
7667	1933	1979	3594	6879	7529
7589	3764	3755	300	6968	7496
7761	256	335	3789	6920	7674
7629	3818	3783	1328	6936	7536
7746	406	469	3776	6884	7677
7924	6846	6818	6788	426	7889
7656	1970	1980	3533	6817	7516
7938	6853	6830	6812	379	7898
7690	1747	1805	3623	6845	7558
7578	3747	3669	915	6920	7499
7691	1762	1843	3654	6866	7571
7563	3712	3658	567	6908	7488
268	7724	7680	7449	7968	5
268	7724	7680	7449	7968	5
269	7725	7681	7450	7969	6

267	7723	7679	7448	7967	4
7923	6839	6816	6798	363	7883
7703	2231	2261	3691	6936	7566
7745	485	444	3788	6848	7682
7692	1963	1995	3630	6904	7553
3301	7694	7664	7536	7834	3425
7772	429	438	3780	6933	7687
7696	1890	1922	3611	6861	7560
7694	1806	1888	3587	6848	7558
7933	6819	6802	6754	431	7893
7593	3792	3741	304	6948	7480
269	7724	7680	7449	7968	6
267	7723	7679	7448	7967	4
246	7649	7685	7520	7980	323
270	7725	7681	7450	7969	7
270	7725	7681	7450	7969	7
7720	2218	2236	3748	6917	7583
7738	478	437	3781	6841	7675
7810	431	523	3875	6889	7727
7719	1944	1969	3585	6810	7613
7704	1912	1935	3556	6806	7598
7693	1801	1883	3584	6843	7555
7696	1807	1889	3588	6849	7558
7711	2225	2253	3753	6932	7574
7709	1937	1944	3594	6872	7611
7701	1929	1936	3586	6864	7603
7670	2058	1957	3535	6779	7532
7765	434	443	3785	6922	7680
7640	1836	1904	3443	6794	7568
7691	1878	1912	3611	6858	7555
7665	1726	1808	3617	6845	7527
7602	3807	3765	551	6891	7498
7923	6847	6819	6783	419	7888
7575	3745	3667	912	6919	7496
7798	182	275	3761	6940	7715
3376	7742	7702	7500	7868	3500
7575	3745	3667	912	6919	7496
7785	286	364	3785	6944	7698
7561	3705	3696	353	6935	7468
7750	460	538	3798	6883	7661
7740	230	328	3788	6923	7653
7787	94	212	3801	6933	7692
7639	1831	1899	3438	6789	7567
8047	6975	6930	6928	574	8008
7826	460	548	3850	6891	7741
7592	3752	3758	461	6898	7512
7768	2125	2125	3784	6915	7646
7863	3636	3625	4230	7007	7779
7680	1736	1794	3612	6834	7548
7686	1834	1880	3587	6801	7576
3377	7743	7703	7501	7869	3501
7764	438	490	3812	6916	7685
7806	155	247	3800	6949	7723
3240	7703	7670	7494	7822	3370
7610	3827	3794	470	6938	7532

7614	3758	3724	383	6940	7521
7708	1811	1869	3640	6880	7567
7756	416	425	3761	6917	7671
3375	7741	7701	7499	7867	3499
7581	3790	3760	328	6942	7488
7581	3790	3760	328	6942	7488
3231	7694	7661	7485	7813	3361
7700	1928	1935	3585	6863	7602
7693	1882	1914	3611	6863	7557
7631	3683	3605	1233	6943	7550
7554	3807	3759	191	6922	7441
7657	1718	1800	3609	6837	7519
7644	1836	1904	3443	6794	7572
7775	154	265	3801	6949	7692
7803	2316	2340	3696	6925	7707
26	7801	7773	7554	8019	259
7777	299	389	3855	6919	7690
3375	7741	7701	7499	7867	3499
3374	7740	7700	7498	7866	3498
7709	2223	2251	3751	6930	7572
7677	1937	1910	3587	6851	7529
7915	6817	6813	6764	442	7875
7703	2231	2261	3691	6936	7566
7703	2231	2261	3691	6936	7566
3376	7742	7702	7500	7868	3500
7778	277	355	3778	6937	7691
7764	256	335	3792	6920	7677
7639	1831	1899	3438	6789	7567
7593	3766	3757	302	6970	7499
7614	3824	3791	470	6950	7536
3303	7694	7664	7537	7834	3427
7691	1799	1881	3582	6840	7553
7687	1846	1882	3590	6801	7577
7678	1968	1998	3609	6913	7539
7713	1943	1950	3600	6878	7615
7674	1849	1923	3615	6916	7542
7672	1847	1921	3613	6914	7540
7701	1878	1952	3644	6942	7569
7962	6872	6839	6822	432	7905
7561	3782	3751	999	6913	7478
7701	1929	1936	3586	6864	7603
7769	2300	2328	3741	6916	7673
3232	7695	7662	7486	7814	3362
3298	7787	7754	7577	7852	3432
7618	3754	3708	649	6945	7533
7673	1694	1776	3618	6832	7567
3294	7799	7757	7629	8051	3413
7712	2226	2254	3754	6933	7575
7718	2232	2260	3760	6939	7581
7677	1751	1833	3640	6860	7539
7796	117	217	3765	6941	7711
7934	6879	6856	6854	364	7900
7698	1952	1933	3585	6871	7550
7893	6815	6787	6769	498	7860
7761	293	382	3765	6927	7674

7758	290	379	3762	6924	7671
7790	2304	2336	3721	6911	7694
7690	1747	1805	3623	6845	7558
3301	7692	7662	7535	7832	3425
3302	7693	7663	7534	7833	3426
3291	7796	7754	7626	8046	3410
7716	1937	1954	3585	6797	7606

AZ-TG73171	AZ-TG73251	AZ-TG73331	AZ-TG73319	AZ-TG73315	AZ-TG73343	AZ-TG73651
7620	7571	3720	3588	7701	3748	3736
7699	7628	1838	1823	7745	1961	1838
7689	7626	2008	659	7732	2133	1977
7873	7728	3905	3865	7925	3923	3901
7584	7537	3663	3532	7648	3657	3661
25558	25525	25045	25027	25595	24964	25045
7712	7630	1828	1675	7770	2067	1834
7731	7635	1943	1847	7757	2272	1929
7712	7626	2001	1883	7755	2235	1997
25557	25524	25044	25026	25594	24963	25044
7777	7684	318	1725	7829	2144	262
7854	7768	2376	2123	7891	1599	2354
3296	2344	7679	7589	3464	7696	7683
7653	7544	1944	1759	7686	2108	1962
7698	7640	1991	1808	7744	1865	1979
8052	7815	6989	6916	8062	6955	6996
7661	7582	1765	1652	7680	2032	1751
7806	7715	2325	2015	7835	1462	2331
7806	7715	2325	2015	7835	1462	2331
7764	7680	1961	1854	7815	2226	1965
7881	7779	3659	3384	7894	3673	3651
7719	7631	2222	2082	7756	1677	2226
3410	2647	7799	7714	3478	7795	7802
7682	7606	1843	1623	7733	1932	1847
7813	7715	81	1810	7876	2191	133
7775	7682	354	1765	7831	2190	272
3316	397	7718	7632	3454	7704	7720
7691	7639	1965	1927	7727	2100	1953
3299	2465	7782	7677	3316	7774	7780
3299	2465	7782	7677	3316	7774	7780
7656	7577	1881	1776	7706	2106	1835
31185	31213	31011	31002	31273	30950	31005
88	3285	7802	7702	1882	7767	7802
1935	3447	7915	7811	217	7844	7911
7632	7585	3603	3548	7708	3663	3633
7632	7585	3603	3548	7708	3663	3633
3375	2651	7716	7610	3379	7716	7720
2033	3393	7887	7786	1917	7826	7883
7778	7669	505	1876	7834	2234	439
7813	7711	81	1800	7874	2187	129
7720	7632	2223	2083	7757	1678	2227
7582	7548	3763	3614	7653	3785	3767
790	3458	7728	7622	2113	7674	7723
7938	7704	6833	6790	7953	6814	6840
7925	7699	6811	6773	7940	6808	6818
7600	7547	3649	3558	7661	3732	3657
7766	7649	538	1929	7826	2300	462
7954	7728	6841	6803	7968	6838	6848
7902	7788	2216	2002	7927	2355	2228
857	3499	7728	7636	2173	7674	7723
7954	7726	6813	6753	7967	6788	6816
292	3416	7724	7566	2045	7655	7722
7725	7637	2227	2088	7762	1683	2231
7749	7627	2075	1980	7771	2330	2087

7728	7628	1964	1866	7754	2280	1950
7678	7550	1924	1829	7695	2145	1950
7702	7627	1778	1685	7716	2055	1760
7709	7646	1942	1876	7737	2065	1932
1917	3421	7880	7777	2	7804	7873
3403	2680	7690	7584	3408	7690	7694
7703	7619	1975	607	7748	2128	1973
7725	7636	1948	1854	7751	2271	1950
7701	7587	1992	1910	7730	2283	1994
7728	7641	1947	1869	7758	2255	1949
7705	7621	1975	607	7750	2128	1973
7936	7710	6800	6778	7951	6813	6801
112	3353	7773	7666	1995	7730	7773
7720	7657	1953	1887	7748	2074	1943
7720	7632	2219	2079	7755	1674	2223
289	3413	7723	7565	2042	7654	7721
290	3414	7724	7566	2043	7655	7722
7853	7760	2355	2132	7893	1654	2331
7699	7628	1838	1823	7745	1961	1838
7718	7659	1962	1904	7746	2073	1952
3283	62	7708	7635	3417	7693	7710
7725	7633	2234	2098	7762	1693	2232
7691	7571	748	1997	7746	2396	689
1924	3429	7885	7782	15	7809	7878
3296	2464	7779	7674	3316	7771	7777
7811	7595	6840	6802	7824	6841	6852
7725	7632	1927	1847	7755	2273	1929
7703	7630	1997	643	7744	2122	1965
90	3287	7805	7705	1884	7770	7805
293	3417	7727	7569	2046	7658	7725
18	3285	7813	7710	1911	7776	7813
13	3284	7812	7709	1910	7775	7812
11	3288	7816	7713	1914	7779	7816
89	3284	7802	7702	1881	7767	7802
7810	7720	2334	2064	7847	1407	2350
3297	2345	7680	7590	3465	7697	7684
7833	7730	334	1992	7900	2321	414
3288	625	7759	7678	3383	7726	7761
7729	7639	2203	2081	7766	1682	2207
7780	7687	327	1729	7832	2148	261
7789	7675	410	1791	7843	2208	344
7816	7720	241	1837	7872	2197	167
7701	7630	1988	638	7746	2121	1968
7824	7724	203	1812	7876	2197	129
17	3288	7816	7713	1914	7779	7816
7960	7752	6749	6691	7968	6736	6754
7925	7701	6809	6771	7942	6806	6816
7772	7680	340	1727	7822	2164	268
7612	7552	3721	3598	7674	3747	3726
7663	7553	1957	1772	7694	2121	1975
7610	7579	3649	3575	7690	3682	3673
86	3283	7800	7700	1880	7765	7800
7625	7575	3789	3663	7694	3784	3785
8047	7810	6984	6911	8057	6950	6991
7760	7670	498	1616	7811	2091	476

97	3284	7807	7707	1891	7771	7807
7820	7722	198	1807	7871	2192	124
7792	7689	290	1873	7851	2198	232
7569	7523	3775	3634	7641	3772	3774
8035	7794	6943	6882	8043	6915	6950
292	3414	7724	7566	2045	7655	7722
	3291	7819	7716	1917	7782	7819
3291		7713	7640	3421	7700	7715
7819	7713		1813	7880	2182	166
7716	7640	1813		7777	2085	1807
1917	3421	7880	7777		7804	7873
7782	7700	2182	2085	7804		2192
7819	7715	166	1807	7873	2192	
7801	7689	419	1800	7855	2217	353
7957	7749	6746	6688	7965	6733	6751
7593	7556	3758	3616	7664	3769	3765
3290	627	7761	7680	3385	7728	7763
7791	7677	412	1793	7845	2210	346
7722	7628	2233	2121	7755	1626	2237
7705	7632	1997	643	7746	2122	1965
7693	7618	1769	1676	7707	2046	1751
7813	7711	81	1800	7874	2187	129
7699	7644	1945	1908	7731	2108	1942
7690	7608	1995	709	7729	2146	1973
7929	7705	6817	6779	7946	6814	6824
3295	632	7765	7684	3390	7732	7767
292	3416	7726	7568	2045	7657	7724
7958	7750	6747	6689	7966	6734	6752
7916	7690	6812	6760	7933	6789	6819
7959	7751	6748	6690	7967	6735	6753
7569	7520	3670	3562	7631	3688	3682
7848	7745	346	2008	7915	2337	428
7815	7717	177	1790	7871	2169	121
7762	7660	2182	1892	7796	1570	2184
7775	7688	2309	2187	7812	1627	2301
7666	7617	3851	3717	7736	3848	3847
7666	7617	3851	3717	7736	3848	3847
3280	59	7705	7632	3414	7690	7707
7730	7634	1948	1846	7756	2275	1934
7615	7573	3803	3683	7685	3795	3804
7677	7584	1941	709	7720	2136	1927
7597	7569	3735	3617	7659	3743	3737
7773	7685	337	1731	7829	2151	277
7637	7588	3742	3684	7730	3762	3772
7758	7660	504	1864	7822	2262	445
7933	7699	6826	6783	7948	6807	6833
7666	7556	1956	1771	7699	2120	1974
7947	7723	6833	6795	7966	6832	6840
7701	7621	1751	1725	7722	2063	1745
7586	7541	3679	3575	7672	3691	3707
7702	7627	1778	1685	7716	2055	1760
7571	7528	3666	3536	7641	3685	3660
291	3415	7725	7567	2044	7656	7723
291	3415	7725	7567	2044	7656	7723
292	3416	7726	7568	2045	7657	7724

290	3414	7724	7566	2043	7655	7722
7932	7706	6819	6781	7949	6818	6826
7712	7623	2229	2083	7748	1685	2233
7757	7640	529	1920	7817	2291	453
7703	7630	1995	641	7744	2120	1963
3296	2342	7681	7591	3464	7698	7685
7784	7673	515	1886	7842	2246	449
7706	7627	1894	711	7744	2107	1890
7705	7621	1822	1697	7720	2079	1804
7943	7717	6803	6739	7958	6790	6806
7601	7572	3757	3633	7671	3758	3769
292	3416	7725	7567	2045	7656	7723
290	3414	7724	7566	2043	7655	7722
269	3372	7648	7558	2043	7652	7648
293	3417	7726	7568	2046	7657	7724
293	3417	7726	7568	2046	7657	7724
7729	7644	2218	2080	7773	1629	2222
7750	7633	522	1913	7810	2284	446
7822	7721	359	1953	7889	2292	433
7729	7634	1956	1860	7752	2265	1946
7714	7618	1922	1836	7742	2267	1924
7704	7616	1817	1694	7717	2074	1799
7707	7622	1823	1698	7722	2080	1805
7720	7632	2223	2083	7757	1678	2227
7718	7655	1951	1885	7746	2074	1941
7710	7647	1943	1877	7738	2066	1933
7681	7583	2070	1987	7708	2325	2066
7777	7666	520	1883	7835	2243	454
7651	7574	1880	1775	7703	2105	1834
7701	7617	1882	739	7739	2079	1878
7676	7600	1740	1627	7693	2025	1724
7610	7546	3759	3637	7685	3771	3751
7932	7698	6827	6784	7947	6808	6834
7583	7538	3677	3572	7669	3687	3705
7810	7708	79	1798	7871	2185	127
3373	2649	7714	7608	3377	7714	7718
7583	7538	3677	3572	7669	3687	3705
7797	7705	362	1763	7849	2180	304
7569	7522	3670	3562	7633	3688	3682
7760	7670	498	1616	7811	2091	476
7752	7667	331	1730	7804	2150	262
7799	7705	252	1817	7853	2178	180
7650	7571	1875	1770	7700	2100	1829
8056	7836	6955	6912	8072	6926	6962
7838	7735	358	2012	7907	2335	434
7600	7553	3722	3577	7674	3694	3720
7779	7695	2115	1989	7804	303	2125
7870	7766	3648	3373	7883	3662	3640
7691	7613	1740	1714	7712	2052	1734
7697	7624	1830	1835	7745	1941	1828
3374	2650	7715	7609	3378	7715	7719
7776	7676	482	1788	7832	2197	406
7818	7719	95	1792	7879	2163	183
3247	283	7694	7613	3352	7671	7696
7618	7568	3781	3646	7686	3785	3777

7622	7568	3706	3583	7682	3710	3709
7717	7641	1809	28	7778	2077	1817
7768	7657	502	1873	7826	2233	436
3372	2648	7713	7607	3376	7713	7717
7589	7547	3738	3605	7647	3734	3745
7589	7547	3738	3605	7647	3734	3745
3238	274	7685	7604	3343	7662	7687
7709	7646	1942	1876	7737	2065	1932
7703	7619	1886	741	7741	2083	1882
7641	7593	3627	3556	7717	3667	3631
7562	7531	3778	3661	7634	3768	3778
7668	7592	1732	1619	7685	2017	1716
7655	7576	1880	1775	7705	2105	1834
7787	7694	101	1777	7848	2161	182
7812	7722	2300	2038	7847	1435	2316
81	3282	7800	7700	1879	7765	7800
7782	7675	249	1883	7847	2264	315
3372	2648	7713	7607	3376	7713	7717
3371	2647	7712	7606	3375	7712	7716
7718	7630	2221	2081	7755	1676	2225
7687	7609	1939	840	7733	2137	1939
7925	7711	6812	6755	7944	6797	6816
7712	7623	2229	2083	7748	1685	2233
7712	7623	2229	2083	7748	1685	2233
3373	2649	7714	7608	3377	7714	7718
7790	7698	353	1756	7842	2171	295
7776	7686	337	1732	7832	2151	277
7650	7571	1875	1770	7700	2100	1829
7601	7573	3737	3620	7665	3746	3739
7622	7573	3778	3644	7690	3782	3774
3298	2346	7681	7591	3466	7698	7685
7702	7614	1815	1692	7715	2072	1797
7698	7629	1844	1831	7748	1971	1850
7689	7607	1994	708	7730	2145	1972
7722	7659	1957	1891	7750	2080	1947
7684	7608	1845	1625	7735	1934	1849
7682	7606	1843	1623	7733	1932	1847
7711	7635	1874	1654	7760	1963	1878
7969	7729	6852	6800	7978	6799	6859
7569	7535	3738	3644	7647	3755	3757
7710	7647	1943	1877	7738	2066	1933
7778	7680	2290	2059	7793	1368	2298
3239	275	7686	7605	3344	7663	7688
3307	644	7778	7697	3401	7745	7780
7626	7590	3706	3610	7693	3705	3706
7684	7616	1708	1673	7706	2086	1692
3301	2467	7784	7679	3318	7776	7782
7721	7633	2224	2084	7758	1679	2228
7727	7639	2230	2090	7764	1685	2234
7688	7609	1765	1648	7706	2042	1749
7808	7706	155	1798	7864	2187	33
7946	7721	6859	6805	7966	6858	6866
7708	7630	1956	855	7752	2150	1952
7904	7664	6795	6755	7921	6779	6802
7773	7680	352	1763	7829	2188	270

7770	7677	349	1760	7826	2185	267
7799	7707	2300	1996	7828	1489	2302
7701	7621	1751	1725	7722	2063	1745
3296	2344	7679	7589	3464	7696	7683
3297	2345	7680	7590	3465	7697	7684
3298	2464	7781	7676	3315	7773	7779
7726	7635	1947	1853	7752	2270	1949

AZ-TG73483	blood-09-1294	blood-10-1009	upec-128	upec-203	upec-205	upec-3	upec-33
3786	6646	908	7617	3767	3721	3596	3649
1896	6611	3606	7676	1889	1881	2043	1774
1976	6732	3616	7668	1966	2115	40	1763
3910	6927	3372	7778	3903	3828	3802	3782
3692	6597	271	7569	3682	3627	3522	3533
25045	25057	24917	25551	25040	24970	24978	25008
1940	6657	3582	7667	1933	2065	1794	1524
1896	6607	3570	7678	1889	2228	1940	1811
1999	6719	3609	7675	1992	2237	1952	1911
25044	25056	24916	25550	25039	24969	24977	25007
149	6725	3776	7730	142	2177	1881	1699
2347	6772	3746	7794	2340	1837	2222	2164
7654	7764	7569	2306	7642	7622	7605	7604
1896	6612	3547	7587	1889	2129	1840	1898
1970	6724	3602	7681	1963	2010	1961	1852
6993	568	6930	7858	6986	6966	6947	6892
1766	6647	3598	7617	1759	1898	1761	230
2434	6709	3697	7751	2427	1930	2105	2166
2434	6709	3697	7751	2427	1930	2105	2166
1954	6761	3681	7711	1947	2194	1884	1738
3703	6895	4227	7827	3696	3789	3291	3534
2270	6754	3740	7681	2263	77	2083	1914
7774	7816	7604	2603	7762	7724	7702	7701
1808	6711	3587	7647	1798	2113	1831	1421
402	6754	3760	7763	395	2248	1996	1772
163	6728	3786	7731	142	2239	1926	1748
7694	7726	7539	805	7682	7623	7624	7612
1973	6706	3603	7681	1966	2130	1961	1944
7742	7983	7681	2451	7730	7697	7659	7690
7742	7983	7681	2451	7730	7697	7659	7690
1904	6591	3436	7613	1897	2133	1817	1697
31012	30817	30921	31207	31001	30938	31007	30928
7784	7940	7580	3282	7774	7709	7690	7678
7888	8012	7719	3401	7878	7790	7789	7745
3669	6639	1138	7632	3659	3655	3601	3548
3669	6639	1138	7632	3659	3655	3601	3548
7696	7786	7563	2583	7684	7656	7616	7607
7856	7946	7664	3310	7846	7776	7769	7722
180	6719	3794	7717	155	2312	1996	1852
396	6748	3757	7759	389	2240	1992	1760
2271	6755	3741	7682	2264	78	2084	1915
3821	6657	378	7570	3814	3787	3648	3644
7704	7884	7499	3476	7694	7612	7602	7585
6837	389	6788	7732	6830	6837	6812	6758
6815	218	6784	7727	6808	6832	6808	6759
3698	6593	780	7585	3688	3681	3531	3591
407	6672	3833	7695	400	2383	2063	1919
6845	248	6813	7756	6838	6862	6838	6789
2176	6876	3774	7840	2169	2399	2029	2167
7704	7886	7497	3500	7694	7616	7612	7583
6817	214	6770	7751	6810	6810	6790	6737
7702	7903	7491	3417	7692	7570	7555	7562
2276	6760	3745	7687	2269	83	2089	1920
2044	6715	3710	7678	2037	2302	2043	2071

1926	6623	3572	7671	1919	2214	1945	1812
1864	6638	3575	7601	1857	2126	1878	1922
1785	6691	3635	7660	1778	1938	1749	21
1952	6656	3592	7688	1945	2080	1913	1878
7855	7965	7664	3385	7845	7755	7746	7707
7670	7766	7543	2612	7658	7630	7590	7583
1968	6701	3614	7661	1958	2147	334	1788
1920	6589	3574	7679	1913	2209	1925	1800
1964	6576	3558	7632	1957	2215	1965	1854
1919	6585	3553	7686	1912	2194	1950	1781
1968	6703	3614	7663	1958	2147	334	1788
6802	280	6807	7738	6795	6836	6811	6770
7755	7950	7527	3350	7745	7676	7653	7645
1963	6665	3601	7699	1956	2089	1920	1887
2267	6757	3743	7682	2260	84	2080	1911
7701	7902	7490	3414	7691	7569	7554	7561
7702	7903	7491	3415	7692	7570	7555	7562
2344	6783	3746	7793	2337	1890	2257	2185
1896	6611	3606	7676	1889	1881	2043	1774
1966	6641	3604	7701	1959	2094	1939	1898
7684	7744	7549	637	7672	7619	7625	7613
2280	6766	3748	7683	2273	93	2099	1904
578	6857	3758	7613	571	2434	2112	1983
7860	7972	7671	3393	7850	7760	7751	7712
7739	7978	7678	2450	7727	7694	7656	7687
6853	661	6790	7619	6846	6850	6820	6769
1893	6610	3584	7675	1886	2187	1916	1786
1966	6727	3634	7670	1956	2094	4	1740
7787	7943	7583	3284	7777	7712	7693	7681
7705	7906	7494	3418	7695	7573	7558	7565
7795	7951	7587	3284	7785	7716	7699	7687
7794	7950	7586	3283	7784	7715	7698	7686
7798	7954	7590	3287	7788	7719	7702	7690
7784	7940	7579	3281	7774	7709	7690	7678
2424	6720	3701	7750	2417	2005	2136	2131
7655	7765	7570	2307	7643	7623	7606	7605
655	6688	3833	7776	648	2376	2141	1947
7735	7775	7586	18	7723	7676	7670	7649
2260	6764	3741	7689	2253	120	2092	1917
150	6730	3777	7733	143	2188	1875	1700
43	6739	3800	7723	18	2258	1954	1767
376	6772	3769	7768	369	2233	1949	1747
1967	6720	3632	7672	1957	2089	21	1745
338	6770	3768	7772	331	2240	1970	1758
7798	7954	7590	3287	7788	7719	7702	7690
6751	5	6631	7780	6744	6756	6730	6685
6813	222	6790	7729	6806	6828	6812	6761
148	6725	3775	7726	138	2181	1891	1717
3748	6613	499	7598	3738	3685	3576	3591
1909	6623	3559	7596	1902	2142	1851	1911
3724	6645	901	7621	3714	3681	3577	3602
7782	7939	7578	3280	7772	7707	7688	7676
3814	6655	633	7613	3804	3751	3661	3667
6988	563	6925	7853	6981	6961	6942	6887
379	6697	3775	7713	372	2099	1806	1645

7789	7943	7585	3281	7779	7713	7694	7682
333	6764	3763	7770	326	2235	1965	1753
419	6741	3710	7737	412	2245	1997	1834
3796	6622	485	7560	3775	3749	3632	3645
6947	550	6919	7835	6940	6930	6906	6857
7702	7905	7491	3415	7692	7570	7555	7562
7801	7957	7593	3290	7791	7722	7705	7693
7689	7749	7556	627	7677	7628	7632	7618
419	6746	3758	7761	412	2233	1997	1769
1800	6688	3616	7680	1793	2121	643	1676
7855	7965	7664	3385	7845	7755	7746	7707
2217	6733	3769	7728	2210	1626	2122	2046
353	6751	3765	7763	346	2237	1965	1751
	6748	3809	7737	37	2267	1966	1776
6748		6628	7777	6741	6753	6727	6682
3809	6628		7588	3802	3747	3634	3627
7737	7777	7588		7725	7678	7672	7651
37	6741	3802	7725		2260	1956	1769
2267	6753	3747	7678	2260		2094	1929
1966	6727	3634	7672	1956	2094		1740
1776	6682	3627	7651	1769	1929	1740	
396	6748	3757	7759	389	2240	1992	1760
1984	6670	3560	7688	1977	2127	1955	1919
1996	6737	3613	7649	1986	2084	118	1758
6821	224	6790	7733	6814	6838	6814	6765
7741	7782	7592	25	7729	7682	7676	7655
7704	7905	7493	3417	7694	7572	7557	7564
6749	3	6629	7778	6742	6754	6728	6683
6816	221	6775	7718	6809	6813	6795	6742
6750	4	6630	7779	6743	6755	6729	6684
3696	6603	429	7562	3685	3655	3552	3554
673	6705	3849	7791	666	2394	2157	1963
316	6737	3787	7765	309	2210	1942	1730
2237	6734	3654	7704	2230	1832	1954	1981
2382	6693	3788	7726	2375	1874	2174	2049
3890	6658	502	7643	3880	3819	3719	3749
3890	6658	502	7643	3880	3819	3719	3749
7681	7741	7546	634	7669	7616	7622	7610
1910	6602	3576	7677	1903	2203	1913	1784
3841	6660	387	7605	3831	3789	3675	3667
1910	6696	3593	7632	1900	2124	463	1734
3759	6640	433	7605	3738	3712	3608	3607
144	6721	3776	7733	137	2198	1883	1707
3830	6628	1295	7636	3820	3763	3704	3675
172	6661	3795	7710	147	2322	2011	1849
6830	382	6787	7727	6823	6828	6805	6751
1908	6624	3557	7599	1901	2141	1852	1910
6837	240	6818	7751	6830	6860	6830	6789
1760	6659	3602	7656	1753	1928	1808	473
3746	6599	852	7583	3725	3670	3592	3603
1785	6691	3636	7660	1778	1938	1749	25
3713	6596	565	7560	3703	3659	3538	3544
7703	7904	7492	3416	7693	7571	7556	7563
7703	7904	7492	3416	7693	7571	7556	7563
7704	7905	7493	3417	7694	7572	7557	7564

7702	7903	7491	3415	7692	7570	7555	7562
6823	224	6804	7734	6816	6846	6816	6775
2277	6759	3685	7662	2270	194	2092	1923
398	6663	3824	7686	391	2374	2054	1910
1964	6725	3632	7670	1954	2092	2	1738
7656	7767	7571	2305	7644	7624	7607	7606
176	6728	3802	7721	151	2331	2006	1864
1859	6674	3607	7667	1849	2090	439	1716
1809	6652	3571	7660	1802	1943	1791	364
6807	210	6757	7744	6800	6806	6782	6735
3787	6651	404	7608	3768	3725	3630	3626
7703	7904	7492	3417	7693	7571	7556	7563
7702	7903	7491	3415	7692	7570	7555	7562
7620	7895	7487	3373	7610	7580	7573	7532
7704	7905	7493	3418	7694	7572	7557	7564
7704	7905	7493	3418	7694	7572	7557	7564
2262	6740	3736	7694	2255	165	2151	1980
391	6656	3817	7679	384	2367	2047	1903
650	6689	3858	7767	643	2357	2102	1938
1921	6597	3567	7679	1914	2197	1937	1802
1892	6593	3557	7663	1871	2197	1914	1786
1804	6647	3568	7655	1797	1938	1788	359
1810	6653	3572	7661	1803	1944	1792	365
2271	6755	3741	7682	2264	78	2084	1915
1961	6665	3601	7697	1954	2089	1920	1887
1953	6657	3593	7689	1946	2081	1912	1879
2046	6598	3438	7626	2036	2304	2055	1914
181	6717	3805	7714	156	2328	2003	1861
1903	6590	3435	7610	1896	2132	1816	1696
1833	6673	3607	7657	1823	2056	475	1698
1749	6657	3599	7635	1742	1882	1737	179
3802	6597	619	7582	3795	3727	3630	3655
6831	383	6782	7726	6824	6831	6806	6752
3744	6598	849	7580	3723	3667	3589	3600
394	6745	3755	7756	387	2238	1990	1758
7694	7784	7561	2581	7682	7654	7614	7605
3744	6598	849	7580	3723	3667	3589	3600
183	6745	3799	7751	164	2194	1916	1735
3696	6607	431	7564	3685	3657	3552	3554
379	6697	3775	7713	372	2099	1806	1645
149	6724	3753	7713	142	2191	1884	1703
365	6748	3747	7753	358	2219	1951	1769
1898	6585	3430	7607	1891	2127	1811	1691
6961	573	6885	7865	6954	6950	6937	6876
675	6695	3838	7781	668	2390	2149	1963
3741	6586	349	7585	3731	3674	3571	3584
2136	6733	3791	7730	2129	1522	2030	2003
3692	6885	4216	7814	3685	3778	3280	3523
1749	6648	3591	7648	1742	1917	1797	462
1888	6607	3601	7672	1881	1881	2047	1758
7695	7785	7562	2582	7683	7655	7615	7606
355	6713	3803	7720	348	2264	1904	1756
374	6752	3785	7765	367	2214	1968	1744
7670	7777	7535	652	7658	7608	7604	7597
3818	6634	315	7600	3808	3757	3650	3659

3739	6612	467	7618	3723	3684	3571	3566
1806	6686	3617	7681	1799	2111	641	1670
157	6712	3786	7705	132	2317	1993	1851
7693	7783	7560	2580	7681	7653	7613	7604
3771	6614	449	7581	3752	3696	3596	3590
3771	6614	449	7581	3752	3696	3596	3590
7661	7768	7526	643	7649	7599	7595	7588
1952	6656	3592	7688	1945	2080	1911	1878
1837	6676	3607	7659	1827	2058	477	1700
3673	6645	1145	7640	3663	3660	3595	3542
3802	6626	590	7562	3781	3753	3655	3660
1741	6649	3591	7627	1734	1874	1729	171
1903	6590	3435	7612	1896	2132	1816	1696
391	6752	3762	7742	384	2224	1955	1756
2389	6719	3682	7758	2382	1965	2116	2133
7782	7938	7578	3279	7772	7707	7688	7676
514	6804	3841	7718	507	2294	2043	1806
7693	7783	7560	2580	7681	7653	7613	7604
7692	7782	7559	2579	7680	7652	7612	7603
2269	6753	3739	7680	2262	76	2082	1913
1887	6661	3611	7649	1877	2098	560	1782
6817	221	6745	7738	6810	6817	6794	6744
2277	6759	3685	7662	2270	194	2092	1923
2277	6759	3685	7662	2270	194	2092	1923
7694	7784	7561	2581	7682	7654	7614	7605
174	6738	3792	7744	155	2187	1909	1726
146	6722	3781	7734	139	2198	1884	1708
1898	6585	3430	7607	1891	2127	1811	1691
3761	6642	435	7609	3740	3715	3611	3611
3817	6646	341	7604	3807	3754	3648	3656
7656	7766	7571	2308	7644	7624	7607	7606
1802	6644	3566	7653	1795	1936	1786	357
1902	6607	3609	7679	1895	1901	2059	1790
1995	6736	3612	7648	1985	2083	117	1757
1967	6671	3607	7701	1960	2095	1926	1893
1810	6713	3589	7649	1800	2115	1833	1423
1808	6711	3587	7647	1798	2113	1831	1421
1839	6739	3618	7676	1829	2144	1862	1452
6856	421	6804	7757	6849	6826	6833	6768
3774	6623	969	7575	3764	3753	3673	3649
1953	6657	3593	7689	1946	2080	1912	1876
2381	6702	3716	7716	2374	1863	2119	2103
7662	7769	7527	644	7650	7600	7596	7589
7754	7794	7605	37	7742	7695	7689	7668
3742	6628	589	7628	3721	3688	3604	3615
1715	6645	3600	7651	1708	1939	1790	328
7744	7985	7683	2453	7732	7699	7661	7692
2272	6756	3742	7683	2265	79	2085	1916
2278	6762	3748	7689	2271	85	2091	1922
1774	6672	3622	7644	1767	1899	1758	216
344	6742	3756	7754	337	2234	1960	1746
6863	271	6860	7749	6856	6874	6842	6807
1897	6681	3628	7670	1887	2118	573	1793
6799	490	6781	7688	6792	6804	6777	6721
161	6726	3784	7729	140	2237	1924	1747

158	6723	3781	7726	137	2234	1921	1744
2365	6719	3702	7743	2358	1953	2074	2111
1760	6659	3602	7656	1753	1928	1808	473
7654	7764	7569	2306	7642	7622	7605	7604
7655	7765	7568	2307	7643	7623	7606	7605
7741	7980	7680	2450	7729	7696	7658	7689
1919	6588	3573	7678	1912	2208	1924	1799

AZ-TG71327	AZ-TG71195	AZ-TG71423	NA	12_ECOL	184_ECOL	283_ECOL	775_SBOY
3727	3526	3574	6806	7621	7533	6647	6791
1829	1993	2031	6688	7680	7580	6612	6665
2000	1941	139	6819	7672	7541	6733	6800
3893	3790	3760	7028	7782	7769	6928	7003
3654	3482	3502	6759	7573	7481	6598	6744
25040	25028	24968	25023	25555	25534	25059	25010
1827	1967	1820	6728	7671	7605	6658	6705
1918	1973	1964	6690	7682	7617	6608	6667
2000	2011	1958	6800	7679	7582	6720	6777
25039	25027	24967	25022	25554	25533	25058	25009
309	1916	1903	6798	7734	7680	6726	6793
2357	2275	2262	6875	7798	7700	6773	6852
7675	7632	7586	7730	2311	3425	7765	7715
1943	1897	1860	6681	7591	7505	6613	6658
1972	1899	1979	6805	7685	7551	6725	6782
6989	6919	6955	397	7863	8011	569	392
1756	1897	1779	6730	7621	7516	6648	6707
2320	2271	2095	6804	7755	7703	6710	6781
2320	2271	2095	6804	7755	7703	6710	6781
1958	2002	1894	6842	7715	7619	6762	6819
3652	3585	3262	6926	7831	7792	6896	6919
2229	2154	2073	6839	7685	7575	6755	6814
7795	7707	7683	7792	2608	3524	7817	7780
1838	1903	1879	6806	7651	7542	6712	6783
52	1935	2002	6825	7767	7720	6755	6820
315	1929	1960	6801	7735	7678	6729	6796
7716	7641	7600	7682	810	3433	7727	7667
1958	224	1971	6783	7685	7652	6707	6760
7773	7698	7643	7953	2456	3413	7984	7936
7773	7698	7643	7953	2456	3413	7984	7936
1874	1788	1817	6688	7617	7575	6592	6665
31009	30973	31007	30801	31209	31138	30819	30791
7796	7684	7675	7917	3287	262	7941	7906
7909	7771	7772	7993	3406	2066	8013	7980
3626	3501	3573	6801	7636	7543	6640	6784
3626	3501	3573	6801	7636	7543	6640	6784
7713	7653	7598	7768	2588	3502	7787	7753
7883	7757	7752	7929	3315	2147	7947	7916
486	2012	2022	6792	7721	7683	6720	6787
2	1937	1994	6819	7763	7720	6749	6814
2230	2155	2074	6840	7686	7576	6756	6815
3761	3605	3627	6811	7573	7483	6658	6792
7722	7602	7585	7839	3481	814	7885	7826
6833	6760	6820	185	7737	7894	390	200
6813	6741	6818	14	7732	7876	219	29
3640	3507	3509	6755	7589	7503	6594	6740
507	2058	2091	6745	7699	7693	6673	6740
6843	6771	6848	44	7761	7905	249	59
2195	2132	2049	6955	7844	7754	6877	6932
7722	7598	7595	7829	3505	879	7887	7820
6813	6723	6802	104	7756	7904	215	91
7718	7592	7540	7878	3422	6	7904	7867
2234	2160	2079	6845	7691	7581	6761	6820
2054	2150	2075	6794	7682	7601	6716	6771

1949	1990	1971	6706	7675	7603	6624	6683
1915	1964	1906	6717	7605	7530	6639	6694
1769	1928	1767	6774	7664	7573	6692	6751
1935	76	1925	6733	7692	7604	6657	6710
7874	7731	7729	7946	3390	2045	7966	7933
7687	7627	7572	7792	2617	3531	7767	7777
1970	1913	398	6788	7665	7559	6702	6769
1941	1961	1945	6672	7683	7607	6590	6649
1985	1985	1985	6659	7636	7575	6577	6636
1940	1986	1970	6668	7690	7614	6586	6645
1970	1913	398	6790	7667	7561	6704	6771
6800	6746	6823	92	7743	7887	281	103
7767	7657	7638	7928	3355	384	7951	7911
1946	85	1932	6742	7703	7615	6666	6719
2226	2151	2070	6842	7686	7576	6758	6817
7717	7591	7539	7877	3419	3	7903	7866
7718	7592	7540	7878	3420	4	7904	7867
2334	2284	2297	6886	7797	7699	6784	6863
1829	1993	2031	6688	7680	7580	6612	6665
1955	108	1951	6718	7705	7613	6642	6695
7706	7639	7601	7697	642	3412	7745	7682
2229	2166	2089	6851	7687	7581	6767	6826
732	2111	2141	6760	7618	7564	6858	6751
7879	7736	7734	7953	3398	2052	7973	7940
7770	7695	7640	7948	2455	3410	7979	7931
6845	6759	6833	461	7624	7767	662	470
1920	1958	1934	6693	7679	7603	6611	6670
1992	1955	118	6814	7674	7555	6728	6795
7799	7687	7678	7920	3289	264	7944	7909
7721	7595	7543	7881	3423	7	7907	7870
7807	7693	7684	7923	3289	286	7952	7910
7806	7692	7683	7922	3288	285	7951	7909
7810	7696	7687	7926	3292	289	7955	7913
7796	7684	7675	7917	3286	259	7941	7906
2329	2329	2112	6815	7754	7705	6721	6792
7676	7633	7587	7731	2312	3426	7766	7716
331	2097	2141	6759	7780	7740	6689	6754
7757	7686	7647	7731	23	3415	7776	7716
2210	2147	2082	6849	7693	7585	6765	6824
314	1918	1915	6803	7737	7683	6731	6798
387	1975	1984	6812	7727	7692	6740	6807
227	1928	1953	6845	7772	7706	6773	6840
1987	1953	111	6807	7676	7553	6721	6788
189	1947	1974	6843	7776	7729	6771	6838
7810	7696	7687	7926	3292	289	7955	7913
6751	6673	6740	227	7785	7908	6	224
6811	6743	6822	22	7734	7876	223	33
313	1929	1919	6798	7730	7675	6726	6793
3719	3543	3548	6775	7602	7517	6614	6760
1956	1910	1871	6692	7600	7515	6624	6669
3666	3556	3551	6801	7624	7498	6646	6782
7794	7682	7673	7916	3285	260	7940	7905
3782	3619	3639	6817	7617	7523	6656	6802
6984	6914	6950	392	7858	8006	564	387
491	1842	1816	6778	7717	7663	6698	6769

7801	7690	7679	7920	3286	269	7944	7909
184	1942	1969	6837	7774	7725	6765	6832
277	1962	1999	6814	7741	7681	6742	6809
3763	3581	3610	6784	7564	7450	6623	6769
6943	6877	6914	383	7840	7969	551	390
7718	7592	7540	7880	3420	6	7906	7869
7813	7699	7690	7929	3295	292	7958	7916
7711	7644	7608	7705	632	3416	7750	7690
81	1945	1995	6817	7765	7726	6747	6812
1800	1908	709	6779	7684	7568	6689	6760
7874	7731	7729	7946	3390	2045	7966	7933
2187	2108	2146	6814	7732	7657	6734	6789
129	1942	1973	6824	7767	7724	6752	6819
396	1984	1996	6821	7741	7704	6749	6816
6748	6670	6737	224	7782	7905	3	221
3757	3560	3613	6790	7592	7493	6629	6775
7759	7688	7649	7733	25	3417	7778	7718
389	1977	1986	6814	7729	7694	6742	6809
2240	2127	2084	6838	7682	7572	6754	6813
1992	1955	118	6814	7676	7557	6728	6795
1760	1919	1758	6765	7655	7564	6683	6742
	1937	1994	6819	7763	7720	6749	6814
1937		1968	6747	7692	7594	6671	6724
1994	1968		6824	7653	7542	6738	6805
6819	6747	6824		7738	7880	225	35
7763	7692	7653	7738		3422	7783	7722
7720	7594	7542	7880	3422		7906	7869
6749	6671	6738	225	7783	7906		222
6814	6724	6805	35	7722	7869	222	
6750	6672	6739	226	7784	7907	5	223
3673	3473	3531	6765	7566	7470	6604	6750
347	2113	2159	6776	7795	7755	6706	6771
164	1932	1944	6810	7769	7720	6738	6805
2169	2228	1966	6813	7708	7659	6735	6790
2292	2233	2162	6778	7730	7672	6694	6755
3840	3653	3699	6820	7647	7582	6659	6805
3840	3653	3699	6820	7647	7582	6659	6805
7703	7636	7598	7694	639	3409	7742	7679
1937	1960	1947	6685	7681	7616	6603	6662
3799	3613	3654	6824	7609	7514	6661	6811
1928	1901	507	6785	7636	7531	6697	6766
3732	3525	3589	6802	7609	7498	6641	6787
318	1914	1921	6794	7737	7676	6722	6789
3745	3634	3682	6792	7640	7538	6629	6793
486	2010	2044	6734	7714	7679	6662	6729
6826	6753	6813	190	7731	7889	383	191
1955	1909	1872	6693	7603	7518	6625	6670
6835	6771	6840	52	7756	7898	241	63
1742	1910	1822	6742	7660	7560	6660	6719
3696	3534	3566	6761	7587	7501	6600	6746
1769	1928	1767	6774	7664	7573	6692	6751
3651	3508	3514	6758	7564	7490	6597	6743
7719	7593	7541	7879	3421	5	7905	7868
7719	7593	7541	7879	3421	5	7905	7868
7720	7594	7542	7880	3422	6	7906	7869

7718	7592	7540	7878	3420	2	7904	7867
6821	6757	6826	36	7739	7883	225	47
2236	2102	2082	6844	7666	7568	6760	6819
498	2049	2082	6736	7690	7684	6664	6731
1990	1953	116	6812	7674	7555	6726	6793
7677	7634	7588	7732	2310	3425	7768	7717
492	2017	2036	6801	7725	7689	6729	6796
1889	1849	495	6763	7671	7562	6675	6744
1813	1926	1809	6743	7664	7560	6653	6720
6801	6710	6798	100	7749	7893	211	87
3760	3546	3609	6813	7612	7482	6652	6798
7719	7593	7541	7879	3422	6	7905	7868
7718	7592	7540	7878	3420	4	7904	7867
7642	7583	7558	7870	3378	323	7896	7859
7720	7594	7542	7880	3423	7	7906	7869
7720	7594	7542	7880	3423	7	7906	7869
2221	2176	2145	6825	7698	7585	6741	6800
491	2042	2075	6729	7683	7677	6657	6724
358	2110	2104	6760	7771	7729	6690	6755
1947	1977	1957	6680	7683	7615	6598	6657
1915	1949	1934	6676	7667	7600	6594	6653
1808	1921	1806	6738	7659	7557	6648	6715
1814	1927	1810	6744	7665	7560	6654	6721
2230	2155	2074	6840	7686	7576	6756	6815
1944	83	1932	6742	7701	7613	6666	6719
1936	75	1924	6734	7693	7605	6658	6711
2063	2067	2073	6681	7630	7534	6599	6658
497	2014	2033	6790	7718	7682	6718	6785
1873	1787	1816	6687	7614	7570	6591	6664
1877	1839	531	6762	7661	7557	6674	6743
1731	1890	1755	6740	7639	7529	6658	6717
3744	3590	3608	6759	7586	7500	6598	6744
6827	6754	6814	179	7731	7888	384	194
3694	3532	3563	6760	7584	7498	6599	6745
16	1935	1992	6816	7760	7717	6746	6811
7711	7651	7596	7766	2586	3500	7785	7751
3694	3532	3563	6760	7584	7498	6599	6745
353	1954	1940	6818	7755	7700	6746	6813
3673	3471	3531	6769	7568	7470	6608	6754
491	1842	1816	6778	7717	7663	6698	6769
315	1895	1917	6797	7717	7655	6725	6792
238	1937	1955	6821	7757	7694	6749	6816
1868	1782	1811	6682	7611	7569	6586	6659
6955	6889	6945	369	7870	8008	574	384
355	2106	2147	6766	7785	7743	6696	6761
3709	3547	3549	6748	7589	7514	6587	6733
2120	2077	2058	6814	7734	7648	6734	6789
3641	3574	3251	6916	7818	7781	6886	6909
1731	1899	1811	6731	7652	7550	6649	6708
1819	2001	2035	6684	7676	7578	6608	6661
7712	7652	7597	7767	2587	3501	7786	7752
451	1957	1932	6788	7724	7687	6714	6770
90	1940	1968	6823	7771	7725	6753	6818
7692	7618	7582	7733	657	3372	7778	7718
3770	3588	3630	6796	7604	7534	6635	6781

3704	3527	3543	6774	7622	7523	6613	6759
1812	1909	699	6777	7685	7569	6687	6758
479	2004	2023	6785	7709	7673	6713	6780
7710	7650	7595	7765	2585	3499	7784	7750
3738	3556	3570	6776	7585	7490	6615	6761
3738	3556	3570	6776	7585	7490	6615	6761
7683	7609	7573	7724	648	3363	7769	7709
1935	76	1923	6733	7692	7604	6657	6710
1881	1841	533	6765	7663	7559	6677	6746
3636	3508	3581	6807	7644	7552	6646	6790
3773	3578	3634	6788	7566	7443	6627	6773
1723	1882	1747	6732	7631	7521	6650	6709
1873	1787	1816	6687	7616	7574	6591	6664
99	1923	1960	6823	7746	7694	6753	6818
2295	2298	2092	6812	7762	7709	6720	6789
7794	7682	7673	7915	3284	259	7939	7904
236	2022	2059	6813	7723	7692	6805	6807
7710	7650	7595	7765	2585	3499	7784	7750
7709	7649	7594	7764	2584	3498	7783	7749
2228	2153	2072	6838	7684	7574	6754	6813
1936	1887	614	6750	7653	7531	6662	6731
6813	6702	6807	111	7743	7875	222	98
2236	2102	2082	6844	7666	7568	6760	6819
2236	2102	2082	6844	7666	7568	6760	6819
7711	7651	7596	7766	2586	3500	7785	7751
344	1947	1933	6811	7748	7693	6739	6806
318	1914	1922	6795	7738	7679	6723	6790
1868	1782	1811	6682	7611	7569	6586	6659
3734	3527	3592	6804	7613	7501	6643	6789
3767	3585	3628	6808	7608	7538	6647	6793
7677	7634	7588	7732	2313	3427	7767	7717
1806	1919	1804	6735	7657	7555	6645	6712
1835	1992	2051	6684	7683	7579	6608	6661
1993	1967	17	6823	7652	7541	6737	6804
1950	91	1938	6748	7705	7617	6672	6725
1840	1905	1881	6808	7653	7544	6714	6785
1838	1903	1879	6806	7651	7542	6712	6783
1869	1934	1910	6834	7680	7571	6740	6811
6852	6767	6841	231	7761	7905	422	209
3744	3580	3654	6785	7579	7480	6624	6770
1936	77	1924	6734	7693	7605	6658	6711
2281	2197	2109	6803	7720	7675	6703	6780
7684	7610	7574	7725	649	3364	7770	7710
7776	7705	7666	7750	42	3434	7795	7735
3701	3561	3586	6790	7632	7535	6629	6775
1699	1903	1808	6728	7655	7569	6646	6705
7775	7700	7645	7955	2458	3415	7986	7938
2231	2156	2075	6841	7687	7577	6757	6816
2237	2162	2081	6847	7693	7583	6763	6822
1756	1913	1776	6755	7648	7541	6673	6732
118	1925	1966	6815	7758	7713	6743	6810
6861	6795	6852	92	7754	7900	272	103
1951	1903	629	6770	7674	7552	6682	6751
6795	6730	6785	292	7693	7860	491	301
313	1927	1958	6799	7733	7676	6727	6794

310	1924	1955	6796	7730	7673	6724	6791
2287	2240	2086	6798	7747	7696	6720	6775
1742	1910	1822	6742	7660	7560	6660	6719
7675	7632	7586	7730	2311	3425	7765	7715
7676	7633	7587	7731	2312	3426	7766	7716
7772	7697	7642	7950	2455	3412	7981	7933
1940	1960	1944	6671	7682	7608	6589	6648

966_ECOL	C260_92	IHD45_5	IHD717_3	IHD717_9	IHD717_16	IHD813_9	IHD813_16
6648	828	3821	3761	3671	3787	1050	1050
6613	3525	1977	1815	2060	2149	3700	3700
6734	3539	2178	1951	1972	2196	3729	3729
6929	3344	4046	3920	3836	3805	3526	3526
6599	346	3752	3686	3557	3695	412	412
25058	24897	25050	25056	24959	24971	24952	24952
6659	3525	2029	1815	1947	2043	3689	3689
6609	3481	2151	1908	2246	2374	3649	3649
6721	3540	2165	1970	2275	2440	3725	3725
25057	24896	25049	25055	24958	24970	24951	24951
6727	3690	562	217	2155	2292	3867	3867
6774	3656	2505	2335	1854	1741	3820	3820
7766	7542	7694	7685	7651	7704	7616	7616
6614	3450	2109	1931	2168	2370	3637	3637
6726	3492	2160	1974	1963	2021	3694	3694
570	6972	6951	6982	6960	6913	7009	7009
6649	3510	1968	1728	1991	2061	3705	3705
6711	3635	2478	2318	396	745	3779	3779
6711	3635	2478	2318	396	745	3779	3779
6763	3576	2153	1930	2089	2154	3759	3759
6897	4161	3745	3626	3687	3711	4290	4290
6756	3678	2383	2199	1877	1919	3840	3840
7818	7573	7829	7804	7753	7787	7671	7671
6713	3516	2021	1828	1892	2148	3706	3706
6756	3671	351	170	2181	2304	3844	3844
6730	3690	608	281	2209	2347	3856	3856
7728	7499	7742	7722	7665	7696	7604	7604
6708	3536	2121	1926	2253	2256	3691	3691
7985	7643	7813	7781	7730	7780	7723	7723
7985	7643	7813	7781	7730	7780	7723	7723
6593	3365	2083	1812	2056	2083	3543	3543
30820	30906	31031	31012	30967	30948	30934	30934
7942	7556	7831	7798	7747	7758	7653	7653
8014	7688	7948	7909	7834	7850	7795	7795
6641	1129	3718	3652	3584	3693	1201	1201
6641	1129	3718	3652	3584	3693	1201	1201
7788	7509	7734	7726	7685	7709	7611	7611
7948	7620	7905	7880	7812	7837	7737	7737
6721	3676	751	402	2270	2409	3859	3859
6750	3673	347	164	2169	2292	3840	3840
6757	3679	2384	2200	1878	1920	3841	3841
6659	597	3894	3790	3702	3828	675	675
7886	7460	7753	7721	7658	7680	7507	7507
391	6739	6792	6826	6821	6786	6830	6830
220	6759	6770	6804	6807	6772	6814	6814
6595	722	3740	3686	3610	3750	838	838
6674	3823	796	459	2323	2466	3929	3929
250	6788	6800	6834	6837	6802	6843	6843
6878	3700	2359	2193	2397	2587	3874	3874
7888	7458	7753	7721	7658	7686	7505	7505
216	6745	6768	6802	6783	6746	6796	6796
7905	7468	7753	7718	7657	7670	7580	7580
6762	3683	2388	2205	1883	1925	3845	3845
6717	3628	2158	2086	2271	2435	3803	3803

6625	3485	2160	1921	2242	2370	3646	3646
6640	3487	2073	1923	2131	2296	3677	3677
6693	3563	1972	1739	1990	2058	3757	3757
6658	3504	2098	1905	2190	2195	3663	3663
7967	7631	7915	7871	7796	7812	7736	7736
7768	7489	7708	7700	7665	7683	7591	7591
6703	3525	2143	1948	1933	2175	3710	3710
6591	3471	2156	1909	2243	2363	3641	3641
6578	3503	2200	1953	2257	2377	3651	3651
6587	3445	2155	1908	2230	2329	3640	3640
6705	3525	2143	1948	1933	2175	3710	3710
282	6760	6755	6791	6807	6772	6810	6810
7952	7495	7800	7769	7710	7727	7600	7600
6667	3513	2109	1916	2201	2204	3672	3672
6759	3677	2380	2196	1884	1926	3837	3837
7904	7467	7752	7717	7656	7669	7579	7579
7905	7468	7753	7718	7657	7670	7580	7580
6785	3656	2484	2316	1909	1786	3820	3820
6613	3525	1977	1815	2060	2149	3700	3700
6643	3494	2118	1925	2210	2213	3665	3665
7746	7513	7740	7712	7653	7681	7610	7610
6768	3686	2389	2209	1867	1909	3848	3848
6859	3719	1000	647	2430	2551	3927	3927
7974	7638	7920	7876	7801	7817	7745	7745
7980	7640	7810	7778	7727	7777	7720	7720
663	6740	6803	6845	6837	6814	6857	6857
6612	3469	2135	1888	2235	2355	3648	3648
6729	3552	2157	1942	1954	2174	3719	3719
7945	7559	7834	7801	7750	7761	7656	7656
7908	7471	7756	7721	7660	7673	7583	7583
7953	7563	7842	7809	7756	7769	7660	7660
7952	7562	7841	7808	7755	7768	7659	7659
7956	7566	7845	7812	7759	7772	7663	7663
7942	7555	7831	7798	7747	7758	7652	7652
6722	3624	2501	2325	459	774	3772	3772
7767	7543	7695	7686	7652	7705	7617	7617
6690	3751	44	415	2325	2442	3928	3928
7777	7560	7789	7763	7702	7724	7641	7641
6766	3686	2364	2180	1908	1950	3851	3851
6732	3691	569	224	2156	2295	3867	3867
6741	3683	664	307	2228	2373	3878	3878
6774	3702	497	142	2186	2307	3896	3896
6722	3550	2156	1937	1959	2177	3718	3718
6772	3710	459	104	2191	2312	3902	3902
7956	7566	7845	7812	7759	7772	7663	7663
7	6606	6708	6740	6737	6696	6661	6661
224	6765	6768	6802	6805	6770	6820	6820
6727	3684	584	241	2145	2284	3843	3843
6615	254	3806	3750	3631	3763	600	600
6625	3462	2122	1944	2181	2383	3649	3649
6647	859	3758	3688	3580	3719	986	986
7941	7554	7829	7796	7745	7756	7651	7651
6657	493	3872	3820	3671	3807	452	452
565	6967	6946	6977	6955	6908	7004	7004
6699	3697	710	435	2107	2214	3868	3868

7945	7561	7836	7803	7753	7762	7658	7658
6766	3705	454	99	2186	2307	3897	3897
6743	3696	546	165	2218	2337	3864	3864
6624	351	3866	3802	3669	3803	618	618
552	6931	6905	6936	6926	6877	6962	6962
7907	7468	7753	7718	7657	7670	7580	7580
7959	7569	7848	7815	7762	7775	7666	7666
7751	7520	7745	7717	7660	7688	7617	7617
6748	3670	346	177	2182	2309	3851	3851
6690	3562	2008	1790	1892	2187	3717	3717
7967	7631	7915	7871	7796	7812	7736	7736
6735	3688	2337	2169	1570	1627	3848	3848
6753	3682	428	121	2184	2301	3847	3847
6750	3696	673	316	2237	2382	3890	3890
4	6603	6705	6737	6734	6693	6658	6658
6630	429	3849	3787	3654	3788	502	502
7779	7562	7791	7765	7704	7726	7643	7643
6743	3685	666	309	2230	2375	3880	3880
6755	3655	2394	2210	1832	1874	3819	3819
6729	3552	2157	1942	1954	2174	3719	3719
6684	3554	1963	1730	1981	2049	3749	3749
6750	3673	347	164	2169	2292	3840	3840
6672	3473	2113	1932	2228	2233	3653	3653
6739	3531	2159	1944	1966	2162	3699	3699
226	6765	6776	6810	6813	6778	6820	6820
7784	7566	7795	7769	7708	7730	7647	7647
7907	7470	7755	7720	7659	7672	7582	7582
5	6604	6706	6738	6735	6694	6659	6659
223	6750	6771	6805	6790	6755	6805	6805
	6605	6707	6739	6736	6695	6660	6660
6605		3767	3703	3592	3726	576	576
6707	3767		433	2339	2456	3942	3942
6739	3703	433		2169	2288	3876	3876
6736	3592	2339	2169		897	3736	3736
6695	3726	2456	2288	897		3872	3872
6660	576	3942	3876	3736	3872		
6660	576	3942	3876	3736	3872		
7743	7510	7737	7709	7650	7678	7607	7607
6604	3484	2144	1909	2228	2350	3657	3657
6662	484	3892	3826	3714	3835	563	563
6698	3531	2109	1914	1902	2161	3680	3680
6642	142	3824	3762	3647	3781	588	588
6723	3693	591	232	2166	2307	3867	3867
6630	1278	3851	3799	3683	3764	1292	1292
6663	3674	758	401	2285	2426	3891	3891
384	6738	6785	6819	6814	6779	6829	6829
6626	3460	2121	1943	2180	2382	3647	3647
242	6793	6792	6826	6831	6796	6848	6848
6661	3527	1954	1724	1973	2051	3722	3722
6601	787	3788	3722	3589	3718	919	919
6693	3563	1972	1739	1990	2058	3758	3758
6598	430	3747	3695	3563	3703	651	651
7906	7469	7754	7719	7658	7671	7581	7581
7906	7469	7754	7719	7658	7671	7581	7581
7907	7470	7755	7720	7659	7672	7582	7582

7905	7468	7753	7718	7657	7670	7580	7580
226	6779	6778	6812	6817	6780	6834	6834
6761	3617	2390	2206	1946	1921	3794	3794
6665	3814	787	450	2314	2457	3920	3920
6727	3550	2155	1940	1952	2172	3717	3717
7769	7544	7696	7687	7653	7706	7618	7618
6730	3685	769	412	2281	2426	3865	3865
6676	3533	2067	1867	1853	2114	3702	3702
6654	3498	2009	1783	2025	2065	3693	3693
212	6732	6760	6798	6775	6736	6784	6784
6653	191	3854	3790	3657	3792	623	623
7906	7469	7754	7719	7658	7671	7581	7581
7905	7468	7753	7718	7657	7670	7580	7580
7897	7444	7677	7644	7632	7645	7606	7606
7907	7470	7755	7720	7659	7672	7582	7582
7907	7470	7755	7720	7659	7672	7582	7582
6742	3672	2389	2183	1864	1935	3832	3832
6658	3807	780	443	2307	2450	3913	3913
6691	3776	85	410	2312	2429	3951	3951
6599	3478	2156	1917	2229	2357	3655	3655
6595	3455	2130	1883	2231	2351	3636	3636
6649	3495	2004	1778	2022	2060	3690	3690
6655	3499	2010	1784	2026	2066	3694	3694
6757	3679	2384	2200	1878	1920	3841	3841
6667	3513	2107	1914	2199	2202	3672	3672
6659	3505	2099	1906	2191	2194	3664	3664
6600	3486	2272	2035	2332	2444	3655	3655
6719	3690	774	417	2278	2423	3870	3870
6592	3364	2082	1811	2055	2082	3542	3542
6675	3533	2055	1855	1835	2094	3702	3702
6659	3526	1943	1703	1962	2032	3721	3721
6599	511	3842	3790	3649	3783	630	630
385	6733	6786	6820	6815	6780	6824	6824
6600	784	3786	3720	3585	3714	916	916
6747	3671	345	162	2167	2290	3838	3838
7786	7507	7732	7724	7683	7707	7609	7609
6600	784	3786	3720	3585	3714	916	916
6747	3696	604	261	2173	2311	3891	3891
6609	14	3767	3703	3592	3726	578	578
6699	3697	710	435	2107	2214	3868	3868
6726	3662	573	226	2156	2299	3866	3866
6750	3703	508	153	2194	2315	3887	3887
6587	3359	2077	1806	2050	2077	3537	3537
575	6903	6914	6948	6932	6897	6857	6857
6697	3753	68	435	2341	2458	3930	3930
6588	399	3815	3735	3606	3744	444	444
6735	3692	2260	2102	1550	1692	3860	3860
6887	4150	3734	3615	3676	3700	4279	4279
6650	3516	1943	1713	1962	2040	3711	3711
6609	3528	1969	1807	2048	2145	3709	3709
7787	7508	7733	7725	7684	7708	7610	7610
6715	3726	699	403	2202	2342	3894	3894
6754	3697	355	134	2165	2286	3880	3880
7779	7495	7726	7698	7641	7663	7596	7596
6636	428	3872	3806	3681	3815	195	195

6614	195	3785	3731	3621	3755	593	593
6688	3563	2004	1786	1896	2191	3727	3727
6714	3666	756	399	2267	2412	3851	3851
7785	7506	7731	7723	7682	7706	7608	7608
6616	192	3821	3767	3639	3770	599	599
6616	192	3821	3767	3639	3770	599	599
7770	7486	7717	7689	7632	7654	7587	7587
6658	3504	2098	1905	2190	2193	3663	3663
6678	3533	2059	1859	1837	2096	3702	3702
6647	1142	3728	3662	3586	3697	1207	1207
6628	459	3867	3801	3666	3802	653	653
6651	3518	1935	1695	1954	2024	3713	3713
6592	3364	2082	1811	2055	2082	3542	3542
6754	3669	375	152	2170	2295	3879	3879
6721	3612	2467	2291	419	728	3761	3761
7940	7554	7829	7796	7745	7756	7651	7651
6806	3759	495	276	2246	2360	3935	3935
7785	7506	7731	7723	7682	7706	7608	7608
7784	7505	7730	7722	7681	7705	7607	7607
6755	3677	2382	2198	1876	1918	3839	3839
6663	3500	2112	1906	1887	2148	3671	3671
223	6721	6771	6809	6791	6752	6796	6796
6761	3617	2390	2206	1946	1921	3794	3794
6761	3617	2390	2206	1946	1921	3794	3794
7786	7507	7732	7724	7683	7707	7609	7609
6740	3689	595	252	2166	2304	3884	3884
6724	3696	591	232	2166	2307	3872	3872
6587	3359	2077	1806	2050	2077	3537	3537
6644	144	3826	3764	3649	3783	590	590
6648	428	3869	3803	3678	3812	191	191
7768	7544	7696	7687	7653	7706	7618	7618
6646	3493	2002	1776	2020	2058	3688	3688
6609	3526	1995	1809	2072	2165	3702	3702
6738	3530	2158	1943	1965	2161	3698	3698
6673	3519	2113	1920	2205	2208	3678	3678
6715	3518	2023	1830	1894	2150	3708	3708
6713	3516	2021	1828	1892	2148	3706	3706
6741	3547	2052	1859	1923	2179	3737	3737
423	6774	6811	6845	6822	6785	6835	6835
6625	962	3847	3777	3656	3791	1029	1029
6659	3505	2099	1906	2191	2194	3664	3664
6704	3654	2441	2279	534	762	3804	3804
7771	7487	7718	7690	7633	7655	7588	7588
7796	7579	7808	7782	7721	7743	7660	7660
6630	550	3787	3731	3598	3738	622	622
6647	3527	1911	1671	1992	2070	3724	3724
7987	7645	7815	7783	7732	7781	7725	7725
6758	3680	2385	2201	1879	1921	3842	3842
6764	3686	2391	2207	1885	1927	3848	3848
6674	3549	1968	1728	1979	2049	3744	3744
6744	3671	425	106	2179	2296	3832	3832
273	6835	6818	6852	6857	6812	6890	6890
6683	3502	2129	1929	1907	2166	3683	3683
492	6719	6754	6788	6790	6757	6810	6810
6728	3688	606	279	2207	2346	3854	3854

6725	3685	603	276	2204	2343	3851	3851
6721	3640	2457	2287	245	710	3784	3784
6661	3527	1954	1724	1973	2051	3722	3722
7766	7542	7694	7685	7651	7704	7616	7616
7767	7541	7695	7686	7652	7705	7615	7615
7982	7642	7812	7780	7729	7779	7722	7722
6590	3470	2155	1908	2242	2362	3640	3640

AZ_TG76998	H124600634	H134240608	AZ-TG71191	2012C-3377	2013C-4350	MGH108
7561	3556	944	3583	831	3756	1243
7620	1935	3648	2057	3580	1819	3693
7616	1941	3645	473	3582	1901	3718
7718	3840	3410	3780	3405	3924	3526
7527	3473	296	3491	346	3671	1205
25519	24989	24920	24986	24938	25041	24889
7622	1757	3646	1768	3578	1860	3636
7627	127	3623	1950	3537	1852	3606
7616	1781	3691	1981	3595	1934	3643
25518	24988	24919	24985	24937	25040	24888
7676	1833	3815	1847	3747	67	3804
7760	2417	3782	2193	3709	2275	3748
2339	7612	7593	7564	7577	7646	7606
7534	1805	3579	1855	3489	1829	3623
7630	2033	3640	1903	3561	1911	3638
7804	6854	6988	6920	7009	6966	6995
7574	1786	3617	1733	3563	1697	3623
7705	2362	3759	2051	3690	2359	3724
7705	2362	3759	2051	3690	2359	3724
7670	1970	3705	1858	3631	1885	3712
7766	3497	4267	3334	4200	3650	4196
7619	2190	3804	2091	3735	2201	3783
2640	7708	7618	7672	7607	7767	7650
7596	1839	3625	1745	3574	1745	3661
7707	1945	3805	1932	3736	326	3753
7674	1875	3815	1865	3728	109	3791
406	7637	7554	7577	7546	7690	7583
7631	2023	3668	1913	3589	1903	3662
2460	7694	7700	7626	7680	7744	7718
2460	7694	7700	7626	7680	7744	7718
7567	1768	3484	1889	3418	1837	3512
31211	31017	30969	30986	30927	31009	30941
3278	7716	7602	7662	7584	7756	7624
3440	7796	7742	7763	7716	7866	7785
7575	3476	1161	3560	1147	3645	547
7575	3476	1161	3560	1147	3645	547
2644	7622	7572	7570	7550	7694	7592
3388	7752	7688	7735	7660	7840	7720
7661	1956	3815	1944	3723	256	3754
7703	1937	3799	1928	3732	318	3745
7620	2191	3805	2092	3736	2202	3784
7538	3621	582	3611	553	3785	1453
3451	7621	7520	7571	7486	7678	7550
7693	6681	6818	6789	6776	6810	6790
7688	6679	6818	6779	6796	6788	6786
7537	3503	859	3489	734	3671	1209
7641	2026	3874	2017	3878	323	3864
7717	6709	6847	6809	6825	6818	6816
7778	2137	3819	2086	3737	2109	3843
3492	7621	7518	7581	7484	7678	7548
7718	6659	6790	6761	6776	6788	6780
3409	7614	7512	7529	7496	7674	7536
7625	2196	3809	2097	3740	2207	3788
7617	2043	3752	2084	3667	1986	3758

7620	162	3604	1971	3540	1861	3613
7540	1854	3621	1913	3526	1803	3640
7619	1793	3676	1743	3616	1716	3684
7638	1916	3645	1867	3557	1882	3642
3414	7756	7685	7720	7659	7829	7730
2672	7600	7552	7544	7530	7668	7572
7609	1884	3667	465	3583	1897	3657
7628	118	3616	1949	3528	1849	3588
7579	224	3607	1998	3560	1893	3565
7633	167	3602	1976	3504	1848	3543
7611	1884	3667	465	3583	1897	3657
7699	6675	6800	6782	6783	6785	6780
3342	7684	7548	7631	7523	7727	7577
7649	1927	3654	1876	3566	1893	3651
7620	2187	3801	2088	3734	2198	3780
3406	7613	7511	7528	7495	7673	7535
3407	7614	7512	7529	7496	7674	7536
7752	2439	3782	2200	3709	2272	3738
7620	1935	3648	2057	3580	1819	3693
7651	1930	3657	1889	3547	1896	3634
11	7629	7566	7577	7562	7680	7581
7621	2200	3812	2107	3743	2211	3791
7560	2077	3815	2060	3764	496	3884
3422	7761	7692	7725	7666	7834	7737
2459	7691	7697	7623	7677	7741	7715
7584	6694	6812	6797	6778	6824	6795
7624	160	3593	1944	3526	1822	3596
7620	1913	3675	463	3608	1883	3704
3280	7719	7605	7665	7587	7759	7627
3410	7617	7515	7532	7499	7677	7539
3274	7724	7609	7671	7591	7767	7631
3273	7723	7608	7670	7590	7766	7630
3277	7727	7612	7674	7594	7770	7634
3277	7716	7601	7662	7583	7756	7623
7710	2365	3745	2078	3679	2354	3739
2340	7613	7594	7565	7578	7647	7607
7722	2128	3876	2095	3808	575	3839
632	7675	7603	7630	7603	7731	7634
7627	2193	3815	2100	3741	2186	3792
7679	1831	3816	1845	3748	64	3808
7667	1901	3829	1898	3736	135	3818
7712	1916	3831	1925	3761	299	3815
7620	1920	3675	468	3608	1888	3699
7716	1935	3837	1938	3769	261	3823
3277	7727	7612	7674	7594	7770	7634
7744	6605	6663	6699	6643	6724	6631
7690	6683	6822	6783	6802	6786	6790
7672	1839	3798	1843	3742	75	3782
7542	3532	526	3547	320	3737	1281
7543	1818	3591	1868	3501	1842	3636
7569	3516	973	3541	871	3703	812
3276	7714	7600	7660	7582	7754	7622
7565	3622	655	3628	501	3801	1179
7799	6849	6983	6915	7004	6961	6990
7662	1789	3822	1748	3754	287	3793

3277	7720	7605	7667	7589	7761	7629
7714	1930	3832	1933	3764	256	3818
7681	1961	3759	1979	3755	335	3783
7513	3592	546	3594	300	3789	1328
7783	6815	6950	6879	6968	6920	6936
3407	7614	7512	7529	7496	7674	7536
3280	7730	7615	7677	7597	7773	7637
59	7634	7573	7584	7569	7685	7588
7705	1948	3803	1941	3735	337	3742
7632	1846	3683	709	3617	1731	3684
3414	7756	7685	7720	7659	7829	7730
7690	2275	3795	2136	3743	2151	3762
7707	1934	3804	1927	3737	277	3772
7681	1910	3841	1910	3759	144	3830
7741	6602	6660	6696	6640	6721	6628
7546	3576	387	3593	433	3776	1295
634	7677	7605	7632	7605	7733	7636
7669	1903	3831	1900	3738	137	3820
7616	2203	3789	2124	3712	2198	3763
7622	1913	3675	463	3608	1883	3704
7610	1784	3667	1734	3607	1707	3675
7703	1937	3799	1928	3732	318	3745
7636	1960	3613	1901	3525	1914	3634
7598	1947	3654	507	3589	1921	3682
7694	6685	6824	6785	6802	6794	6792
639	7681	7609	7636	7609	7737	7640
3409	7616	7514	7531	7498	7676	7538
7742	6603	6661	6697	6641	6722	6629
7679	6662	6811	6766	6787	6789	6793
7743	6604	6662	6698	6642	6723	6630
7510	3484	484	3531	142	3693	1278
7737	2144	3892	2109	3824	591	3851
7709	1909	3826	1914	3762	232	3799
7650	2228	3714	1902	3647	2166	3683
7678	2350	3835	2161	3781	2307	3764
7607	3657	563	3680	588	3867	1292
7607	3657	563	3680	588	3867	1292
	7626	7561	7574	7559	7677	7578
7626		3625	1947	3539	1839	3614
7561	3625		3640	481	3816	1282
7574	1947	3640		3581	1845	3642
7559	3539	481	3581		3752	1290
7677	1839	3816	1845	3752		3810
7578	3614	1282	3642	1290	3810	
7652	1946	3819	1957	3724	238	3831
7688	6674	6815	6782	6775	6803	6787
7546	1817	3589	1867	3499	1841	3635
7712	6701	6850	6801	6830	6810	6818
7613	1855	3674	1763	3580	1691	3650
7525	3495	926	3553	790	3709	810
7619	1793	3676	1743	3616	1716	3684
7518	3507	667	3505	488	3684	1155
3408	7615	7513	7530	7497	7675	7537
3408	7615	7513	7530	7497	7675	7537
3409	7616	7514	7531	7498	7676	7538

3407	7614	7512	7529	7496	7674	7536
7695	6687	6836	6787	6816	6796	6804
7611	2211	3749	2094	3674	2208	3784
7632	2017	3865	2008	3869	314	3855
7620	1911	3673	461	3606	1881	3702
2337	7614	7595	7566	7579	7648	7608
7665	1966	3825	1952	3738	258	3763
7617	1862	3650	376	3589	1794	3658
7613	1829	3611	1781	3551	1740	3599
7709	6651	6785	6755	6767	6780	6766
7562	3578	477	3605	163	3770	1314
3409	7615	7513	7530	7497	7675	7537
3407	7614	7512	7529	7496	7674	7536
3365	7580	7522	7540	7472	7592	7550
3410	7616	7514	7531	7498	7676	7538
3410	7616	7514	7531	7498	7676	7538
7632	2218	3792	2153	3727	2191	3779
7625	2010	3858	2001	3862	307	3848
7713	2119	3901	2054	3833	568	3860
7626	108	3616	1959	3531	1855	3612
7610	92	3607	1940	3502	1833	3589
7608	1824	3608	1778	3548	1735	3596
7614	1830	3612	1782	3552	1741	3600
7620	2191	3805	2092	3736	2202	3784
7647	1925	3654	1874	3566	1891	3651
7639	1917	3646	1866	3558	1883	3643
7575	437	3574	2077	3542	1981	3570
7658	1961	3830	1949	3743	255	3768
7564	1767	3483	1888	3417	1836	3511
7607	1827	3652	412	3589	1768	3664
7592	1785	3639	1709	3579	1680	3635
7536	3609	679	3591	558	3781	1038
7687	6675	6812	6783	6770	6804	6784
7522	3493	923	3551	787	3707	807
7700	1935	3797	1926	3730	316	3743
2642	7620	7570	7568	7548	7692	7590
7522	3493	923	3551	787	3707	807
7697	1861	3835	1878	3743	105	3828
7512	3484	486	3531	144	3693	1280
7662	1789	3822	1748	3754	287	3793
7659	1838	3790	1844	3724	69	3811
7697	1920	3806	1917	3762	286	3814
7561	1762	3478	1883	3412	1831	3506
7825	6825	6943	6916	6940	6934	6924
7727	2134	3878	2101	3810	595	3841
7543	3503	369	3538	413	3722	1237
7685	2231	3817	2048	3747	2068	3740
7753	3486	4256	3323	4189	3639	4185
7605	1844	3663	1752	3569	1680	3639
7616	1936	3657	2047	3583	1813	3700
2643	7621	7571	7569	7549	7693	7591
7668	1938	3812	1866	3781	271	3824
7711	1915	3830	1916	3762	290	3783
292	7613	7550	7563	7540	7664	7573
7558	3592	412	3609	440	3797	1263

7558	3507	523	3542	200	3718	1292
7633	1842	3684	717	3618	1729	3694
7649	1953	3811	1939	3719	245	3749
2641	7619	7569	7567	7547	7691	7589
7537	3535	529	3579	141	3754	1295
7537	3535	529	3579	141	3754	1295
283	7604	7541	7554	7531	7655	7564
7638	1916	3645	1865	3557	1882	3642
7609	1831	3652	414	3589	1772	3664
7582	3470	1162	3562	1150	3645	559
7521	3618	640	3617	393	3795	1217
7584	1777	3631	1701	3571	1672	3627
7566	1767	3483	1888	3417	1836	3511
7686	1928	3807	1899	3739	311	3788
7712	2320	3730	2058	3667	2320	3712
3275	7714	7600	7660	7582	7754	7622
7664	1994	3862	1999	3816	426	3841
2641	7619	7569	7567	7547	7691	7589
2640	7618	7568	7566	7546	7690	7588
7618	2189	3803	2090	3734	2200	3782
7599	1857	3616	497	3556	1831	3611
7703	6663	6773	6763	6757	6788	6782
7611	2211	3749	2094	3674	2208	3784
7611	2211	3749	2094	3674	2208	3784
2642	7620	7570	7568	7548	7692	7590
7690	1852	3828	1871	3736	96	3821
7678	1840	3821	1846	3755	20	3815
7561	1762	3478	1883	3412	1831	3506
7563	3541	483	3584	60	3754	1292
7563	3582	402	3607	440	3794	1253
2341	7614	7595	7566	7579	7648	7608
7606	1822	3606	1776	3546	1733	3594
7621	1955	3649	2065	3583	1823	3699
7597	1946	3653	506	3588	1920	3681
7651	1931	3660	1880	3572	1897	3657
7598	1841	3627	1747	3576	1747	3663
7596	1839	3625	1745	3574	1745	3661
7625	1870	3656	1776	3605	1776	3692
7718	6699	6847	6804	6811	6829	6805
7525	3584	985	3629	971	3748	858
7639	1917	3646	1866	3558	1883	3643
7670	2348	3778	2083	3709	2304	3739
284	7605	7542	7555	7532	7656	7565
651	7694	7622	7649	7622	7750	7653
7580	3543	632	3586	549	3718	1064
7608	1803	3660	1755	3580	1646	3644
2462	7696	7702	7628	7682	7746	7720
7621	2192	3806	2093	3737	2203	3785
7627	2198	3812	2099	3743	2209	3791
7601	1816	3660	1730	3602	1705	3658
7698	1929	3797	1922	3730	266	3757
7710	6721	6894	6813	6872	6836	6862
7620	1864	3631	508	3558	1844	3623
7653	6651	6796	6754	6756	6778	6698
7672	1873	3813	1863	3726	107	3789

7669	1870	3810	1860	3723	104	3786
7697	2309	3762	2022	3695	2294	3733
7613	1855	3674	1763	3580	1691	3650
2339	7612	7593	7564	7577	7646	7606
2340	7613	7592	7565	7576	7647	7607
2459	7693	7699	7625	7679	7743	7717
7627	117	3615	1948	3527	1848	3587

AZ-TG71543	AZ-TG71539	AZ-TG71555	SEQ895	AZ_TG78596	JEONG-9567	KCJ1232	FSIS
3801	6786	3524	6828	3604	686	3658	957
1958	6705	1887	6712	1745	3581	1783	3527
1995	6810	1863	6835	1831	3605	1772	3554
3936	6984	3704	7050	3784	3417	3791	3409
3734	6739	3455	6787	3520	809	3542	538
25021	25019	24942	25033	25028	24927	25017	24900
1989	6718	1918	6752	1515	3569	1533	3515
1958	6679	1824	6706	1874	3495	1820	3491
2018	6791	1774	6816	1953	3547	1920	3542
25020	25018	24941	25032	25027	24926	25016	24899
248	6807	1831	6814	1683	3699	1708	3674
2385	6874	2372	6891	2145	3683	2173	3653
7630	7744	7524	7748	7610	7558	7613	7536
1985	6678	32	6705	1929	3510	1907	3461
2016	6787	1904	6829	1847	3559	1861	3533
6936	431	6848	389	6881	6959	6901	6945
1813	6735	1918	6754	383	3560	239	3506
2478	6805	2322	6822	2166	3628	2175	3606
2478	6805	2322	6822	2166	3628	2175	3606
2017	6824	2057	6866	1788	3640	1747	3610
3732	6905	3474	6944	3618	4169	3543	4140
2301	6827	2192	6861	1909	3693	1923	3658
7746	7794	7640	7808	7700	7613	7710	7589
1869	6802	1805	6822	1514	3542	1430	3499
492	6834	1969	6841	1754	3702	1781	3663
247	6810	1877	6817	1732	3687	1757	3683
7667	7676	7553	7700	7611	7530	7621	7503
2027	6768	1912	6807	1920	3570	1953	3532
7738	7959	7630	7971	7689	7670	7699	7634
7738	7959	7630	7971	7689	7670	7699	7634
1964	6687	1909	6706	1740	3442	1706	3409
30994	30790	30923	30811	30936	30912	30939	30910
7741	7921	7651	7935	7686	7573	7687	7558
7865	7993	7733	8011	7764	7727	7754	7698
3713	6781	3492	6829	3531	628	3557	975
3713	6781	3492	6829	3531	628	3557	975
7678	7775	7564	7786	7605	7546	7616	7516
7821	7932	7712	7949	7742	7662	7731	7633
193	6791	1943	6808	1812	3709	1861	3699
486	6826	1955	6835	1742	3696	1769	3651
2302	6828	2193	6862	1910	3694	1924	3659
3836	6797	3550	6839	3633	1056	3653	758
7696	7864	7562	7869	7596	7475	7594	7474
6825	21	6697	217	6748	6750	6767	6736
6728	184	6687	46	6736	6755	6768	6752
3716	6748	3443	6783	3564	944	3600	628
378	6832	1987	6761	1885	3816	1928	3788
6758	214	6717	76	6766	6784	6798	6781
2265	6952	823	6971	2214	3768	2176	3721
7696	7860	7558	7861	7594	7473	7592	7472
6728	266	6671	128	6714	6737	6746	6734
7677	7887	7516	7896	7558	7499	7571	7488
2307	6833	2198	6867	1915	3698	1929	3663
2130	6791	581	6810	2079	3693	2080	3643

1978	6691	1817	6722	1881	3502	1821	3478
1941	6714	390	6733	1934	3536	1931	3515
1858	6760	1919	6798	482	3612	34	3553
2008	6739	1870	6757	1869	3541	1887	3506
7822	7948	7699	7966	7722	7672	7716	7641
7652	7799	7538	7810	7581	7526	7592	7496
1998	6787	1831	6804	1849	3552	1797	3538
1933	6661	1821	6688	1867	3481	1809	3519
2000	6659	1831	6675	1911	3491	1863	3509
1952	6657	1849	6684	1852	3420	1790	3446
1998	6789	1831	6806	1849	3552	1797	3538
6729	255	6700	76	6747	6754	6779	6763
7711	7936	7618	7946	7649	7517	7654	7498
2019	6748	1881	6766	1880	3550	1896	3515
2300	6830	2189	6864	1906	3692	1920	3655
7676	7886	7515	7895	7557	7498	7570	7487
7677	7887	7516	7896	7558	7499	7571	7488
2382	6885	2384	6902	2150	3673	2194	3653
1958	6705	1887	6712	1745	3581	1783	3527
1994	6730	1884	6742	1889	3529	1907	3530
7655	7691	7549	7715	7616	7528	7622	7521
2311	6839	2200	6873	1899	3701	1913	3666
607	6754	2079	6784	1938	3716	1992	3706
7827	7955	7704	7973	7727	7679	7721	7648
7735	7954	7627	7966	7686	7667	7696	7631
6810	293	6734	489	6755	6780	6778	6765
1952	6673	1783	6709	1853	3487	1795	3503
2011	6805	1852	6830	1808	3592	1749	3538
7744	7924	7654	7938	7689	7576	7690	7561
7680	7890	7519	7899	7561	7502	7574	7491
7752	7927	7660	7941	7695	7580	7696	7565
7751	7926	7659	7940	7694	7579	7695	7564
7755	7930	7663	7944	7698	7583	7699	7568
7741	7921	7651	7935	7686	7572	7687	7557
2485	6820	2329	6833	2161	3620	2140	3595
7631	7745	7525	7749	7611	7559	7614	7537
742	6768	2103	6775	1938	3776	1956	3731
7708	7725	7597	7749	7654	7581	7658	7558
2295	6837	2193	6871	1912	3699	1926	3633
250	6812	1841	6819	1684	3707	1709	3678
145	6821	1899	6828	1751	3723	1776	3701
437	6841	1980	6861	1729	3751	1756	3717
2009	6798	1845	6823	1813	3583	1754	3539
411	6852	1975	6859	1752	3752	1767	3717
7755	7930	7661	7944	7698	7583	7699	7568
6664	385	6627	243	6662	6602	6694	6599
6726	186	6689	50	6738	6761	6770	6758
239	6807	1851	6814	1701	3701	1726	3666
3781	6754	3498	6803	3570	865	3600	469
1998	6689	45	6716	1942	3523	1920	3473
3740	6778	3529	6829	3577	313	3611	722
7739	7920	7649	7934	7684	7571	7685	7556
3811	6812	3572	6845	3646	767	3676	395
6931	426	6843	384	6876	6954	6896	6940
474	6787	1805	6794	1627	3704	1654	3677

7746	7924	7656	7938	7690	7578	7691	7563
406	6846	1970	6853	1747	3747	1762	3712
469	6818	1980	6830	1805	3669	1843	3658
3776	6788	3533	6812	3623	915	3654	567
6884	426	6817	379	6845	6920	6866	6908
7677	7889	7516	7898	7558	7499	7571	7488
7758	7933	7666	7947	7701	7586	7702	7571
7660	7699	7556	7723	7621	7541	7627	7528
504	6826	1956	6833	1751	3679	1778	3666
1864	6783	1771	6795	1725	3575	1685	3536
7822	7948	7699	7966	7722	7672	7716	7641
2262	6807	2120	6832	2063	3691	2055	3685
445	6833	1974	6840	1745	3707	1760	3660
172	6830	1908	6837	1760	3746	1785	3713
6661	382	6624	240	6659	6599	6691	6596
3795	6787	3557	6818	3602	852	3636	565
7710	7727	7599	7751	7656	7583	7660	7560
147	6823	1901	6830	1753	3725	1778	3703
2322	6828	2141	6860	1928	3670	1938	3659
2011	6805	1852	6830	1808	3592	1749	3538
1849	6751	1910	6789	473	3603	25	3544
486	6826	1955	6835	1742	3696	1769	3651
2010	6753	1909	6771	1910	3534	1928	3508
2044	6813	1872	6840	1822	3566	1767	3514
6734	190	6693	52	6742	6761	6774	6758
7714	7731	7603	7756	7660	7587	7664	7564
7679	7889	7518	7898	7560	7501	7573	7490
6662	383	6625	241	6660	6600	6692	6597
6729	191	6670	63	6719	6746	6751	6743
6663	384	6626	242	6661	6601	6693	6598
3674	6738	3460	6793	3527	787	3563	430
758	6785	2121	6792	1954	3788	1972	3747
401	6819	1943	6826	1724	3722	1739	3695
2285	6814	2180	6831	1973	3589	1990	3563
2426	6779	2382	6796	2051	3718	2058	3703
3891	6829	3647	6848	3722	919	3758	651
3891	6829	3647	6848	3722	919	3758	651
7652	7688	7546	7712	7613	7525	7619	7518
1946	6674	1817	6701	1855	3495	1793	3507
3819	6815	3589	6850	3674	926	3676	667
1957	6782	1867	6801	1763	3553	1743	3505
3724	6775	3499	6830	3580	790	3616	488
238	6803	1841	6810	1691	3709	1716	3684
3831	6787	3635	6818	3650	810	3684	1155
	6818	1997	6750	1823	3732	1858	3738
6818		6690	216	6741	6749	6760	6735
1997	6690		6717	1941	3522	1919	3471
6750	216	6717		6766	6789	6798	6786
1823	6741	1941	6766		3568	482	3525
3732	6749	3522	6789	3568		3612	661
1858	6760	1919	6798	482	3612		3553
3738	6735	3471	6786	3525	661	3553	
7678	7888	7517	7897	7559	7500	7572	7489
7678	7888	7517	7897	7559	7500	7572	7489
7679	7889	7518	7898	7560	7501	7573	7490

7677	7887	7516	7896	7558	7499	7571	7488
6736	200	6703	20	6752	6775	6784	6772
2308	6832	2228	6866	1912	3694	1932	3659
369	6823	1978	6752	1876	3807	1919	3779
2009	6803	1850	6828	1806	3590	1747	3536
7632	7746	7526	7750	7612	7560	7615	7538
202	6800	1959	6817	1824	3727	1873	3711
1920	6762	1788	6779	1757	3564	1725	3519
1882	6744	1931	6767	551	3541	373	3490
6715	262	6671	124	6712	6727	6744	6724
3759	6798	3514	6841	3603	845	3635	504
7678	7888	7517	7897	7559	7500	7572	7489
7677	7887	7516	7896	7558	7499	7571	7488
7617	7866	7553	7888	7549	7473	7541	7470
7679	7889	7518	7898	7560	7501	7573	7490
7679	7889	7518	7898	7560	7501	7573	7490
2291	6813	2238	6847	1969	3689	1989	3662
362	6816	1971	6745	1869	3800	1912	3772
735	6769	2076	6776	1929	3797	1947	3756
1952	6669	1815	6696	1865	3486	1811	3509
1911	6665	1803	6692	1857	3447	1795	3496
1877	6739	1926	6762	546	3538	368	3487
1883	6745	1932	6768	552	3542	374	3491
2302	6828	2193	6862	1910	3694	1924	3659
2017	6748	1879	6766	1878	3550	1896	3515
2009	6740	1871	6758	1870	3542	1888	3507
2067	6677	1904	6697	1968	3448	1923	3446
203	6789	1956	6806	1821	3732	1870	3712
1963	6686	1908	6705	1739	3441	1705	3408
1894	6761	1775	6778	1737	3572	1707	3519
1822	6741	1881	6764	340	3567	188	3516
3818	6751	3545	6787	3632	801	3664	395
6819	15	6691	211	6742	6744	6761	6730
3730	6748	3520	6788	3564	19	3609	658
484	6823	1953	6832	1740	3694	1767	3649
7676	7773	7562	7784	7603	7544	7614	7514
3730	6748	3520	6788	3564	19	3609	658
246	6827	1863	6834	1719	3697	1744	3709
3674	6742	3458	6797	3527	789	3563	432
474	6787	1805	6794	1627	3704	1654	3677
226	6806	1841	6813	1687	3709	1712	3675
396	6827	1978	6837	1755	3721	1778	3706
1958	6681	1901	6700	1734	3436	1700	3403
6947	352	6822	401	6863	6900	6885	6879
748	6772	2118	6782	1946	3769	1972	3727
3736	6735	3508	6776	3571	859	3593	589
2191	6807	2020	6832	1978	3673	2012	3671
3721	6895	3463	6934	3607	4158	3532	4129
1812	6730	1930	6755	27	3557	471	3514
1956	6701	1874	6708	1729	3582	1767	3516
7677	7774	7563	7785	7604	7545	7615	7515
428	6799	1882	6802	1754	3746	1765	3709
461	6832	1933	6839	1726	3722	1753	3693
7641	7727	7531	7751	7600	7526	7606	7505
3819	6803	3573	6824	3632	880	3668	614

3762	6747	3458	6802	3539	812	3575	451
1865	6781	1761	6793	1719	3571	1679	3548
183	6784	1946	6801	1811	3708	1860	3698
7675	7772	7561	7783	7602	7543	7613	7513
3765	6750	3484	6804	3563	809	3599	474
3765	6750	3484	6804	3563	809	3599	474
7632	7718	7522	7742	7591	7517	7597	7496
2008	6739	1870	6757	1869	3541	1887	3506
1898	6764	1779	6781	1739	3572	1709	3519
3722	6787	3498	6835	3525	645	3551	977
3742	6794	3563	6816	3638	797	3669	456
1814	6733	1873	6756	332	3559	180	3508
1963	6686	1908	6705	1739	3441	1705	3408
454	6832	1931	6839	1738	3728	1765	3696
2436	6813	2289	6830	2133	3606	2142	3594
7739	7919	7649	7933	7684	7571	7685	7556
602	6827	2023	6827	1788	3785	1815	3742
7675	7772	7561	7783	7602	7543	7613	7513
7674	7771	7560	7782	7601	7542	7612	7512
2300	6826	2191	6860	1908	3692	1922	3657
1883	6746	1849	6766	1809	3543	1791	3539
6705	273	6678	135	6721	6740	6753	6736
2308	6832	2228	6866	1912	3694	1932	3659
2308	6832	2228	6866	1912	3694	1932	3659
7676	7773	7562	7784	7603	7544	7614	7514
237	6820	1856	6827	1710	3690	1735	3702
240	6804	1842	6811	1692	3714	1717	3687
1958	6681	1903	6700	1734	3436	1700	3403
3726	6777	3502	6832	3583	792	3620	490
3817	6815	3570	6836	3629	880	3665	611
7632	7746	7526	7750	7612	7560	7615	7538
1875	6736	1924	6759	544	3536	366	3485
1959	6701	1901	6708	1761	3585	1799	3543
2043	6812	1871	6839	1821	3565	1766	3513
2023	6754	1885	6772	1884	3556	1902	3521
1871	6804	1807	6824	1516	3544	1432	3501
1869	6802	1805	6822	1514	3542	1430	3499
1900	6830	1836	6850	1545	3573	1461	3530
6839	198	6694	261	6759	6769	6777	6766
3782	6782	3594	6813	3636	477	3658	878
2009	6740	1871	6758	1867	3542	1885	3507
2425	6804	2297	6821	2080	3649	2112	3633
7633	7719	7523	7743	7592	7518	7598	7497
7727	7744	7616	7768	7673	7600	7677	7577
3718	6787	3545	6818	3583	703	3624	462
1778	6726	1913	6752	318	3574	337	3521
7740	7961	7632	7973	7691	7672	7701	7636
2303	6829	2194	6863	1911	3695	1925	3660
2309	6835	2200	6869	1917	3701	1931	3666
1847	6756	1916	6779	377	3590	225	3539
434	6824	1969	6831	1740	3692	1755	3655
6776	258	6745	98	6784	6831	6816	6828
1884	6766	1864	6786	1820	3546	1802	3555
6788	122	6664	318	6713	6730	6730	6717
245	6808	1875	6815	1731	3685	1756	3681

242	6805	1872	6812	1728	3682	1753	3678
2413	6799	2279	6816	2117	3639	2120	3613
1823	6741	1941	6766		3568	482	3525
7630	7744	7524	7748	7610	7558	7613	7536
7631	7745	7525	7749	7611	7559	7614	7535
7737	7956	7629	7968	7688	7669	7698	7633
1932	6660	1820	6687	1864	3480	1808	3518

AZ684313	10B06797	cam_1531_1	la_1424	HICF2	HICF32	HICF112	HICF191	GN02531
7532	7530	7533	7531	6814	3689	3848	3594	7606
7579	7579	7580	7578	6698	1885	2002	2041	7589
7540	7540	7541	7539	6821	2113	2061	38	7601
7768	7768	7767	7767	7036	3783	3965	3800	7755
7480	7480	7481	7479	6773	3615	3771	3520	7543
25533	25533	25534	25532	25019	24998	25036	24976	25505
7604	7604	7605	7603	6738	2095	2008	1792	7607
7616	7616	7617	7615	6692	2236	2018	1938	7613
7581	7581	7582	7580	6802	2275	2092	1950	7608
25532	25532	25533	25531	25018	24997	25035	24975	25504
7679	7679	7680	7678	6800	2187	299	1879	7649
7699	7699	7700	7698	6875	1870	2460	2220	7767
3424	3424	3425	3423	7733	7642	7607	7603	39
7504	7504	7505	7503	6691	2216	1966	1838	7513
7550	7550	7551	7549	6815	1973	2076	1959	7617
8010	8010	8011	8009	373	6970	6889	6945	7861
7515	7515	7516	7514	6740	1900	1874	1759	7569
7702	7702	7703	7701	6806	1985	2517	2103	7717
7702	7702	7703	7701	6806	1985	2517	2103	7717
7618	7618	7619	7617	6852	2186	2058	1882	7645
7791	7791	7790	7790	6930	3790	3747	3289	7772
7574	7574	7575	7573	6847	121	2389	2081	7633
3523	3523	3524	3522	7793	7707	7769	7700	2558
7541	7541	7542	7540	6808	2139	1948	1829	7580
7719	7719	7720	7718	6827	2244	510	1994	7681
7677	7677	7678	7676	6803	2249	323	1924	7650
3432	3432	3433	3431	7683	7618	7643	7622	2380
7651	7651	7652	7650	6793	2103	2046	1959	7635
3412	3412	3413	3411	7954	7682	7745	7657	2517
3412	3412	3413	3411	7954	7682	7745	7657	2517
7574	7574	7575	7573	6692	2033	2028	1815	7584
31137	31137	31138	31136	30797	30969	31008	31007	31227
261	261	262	260	7920	7699	7740	7688	3302
2065	2065	2066	2064	7994	7783	7864	7787	3473
7542	7542	7543	7541	6815	3681	3747	3599	7583
7542	7542	7543	7541	6815	3681	3747	3599	7583
3501	3501	3502	3500	7769	7638	7669	7614	2626
2146	2146	2147	2145	7932	7771	7826	7767	3441
7682	7682	7683	7681	6794	2319	386	1994	7640
7719	7719	7720	7718	6821	2236	498	1990	7677
7575	7575	7576	7574	6848	122	2390	2082	7634
7482	7482	7483	7481	6825	3728	3861	3646	7539
813	813	814	812	7854	7614	7702	7600	3442
7893	7893	7894	7892	201	6841	6830	6810	7751
7875	7875	7876	7874	30	6838	6730	6806	7726
7502	7502	7503	7501	6769	3661	3723	3529	7546
7692	7692	7693	7691	6747	2405	19	2061	7618
7904	7904	7905	7903	60	6868	6760	6836	7755
7753	7753	7754	7752	6957	2473	2278	2027	7768
878	878	879	877	7846	7618	7702	7610	3502
7903	7903	7904	7902	112	6816	6718	6788	7756
5	5	4	4	7881	7566	7682	7553	3425
7580	7580	7581	7579	6853	127	2395	2087	7637
7600	7600	7601	7599	6796	2374	2117	2041	7575

7602	7602	7603	7601	6708	2232	2009	1943	7611
7529	7529	7530	7528	6719	2214	1960	1876	7529
7572	7572	7573	7571	6784	1932	1919	1747	7615
7603	7603	7604	7602	6743	2055	2042	1911	7636
2044	2044	2045	2043	7949	7748	7817	7744	3464
3530	3530	3531	3529	7793	7612	7643	7588	2652
7558	7558	7559	7557	6790	2137	2066	332	7584
7606	7606	7607	7605	6674	2217	2013	1923	7616
7574	7574	7575	7573	6661	2265	1968	1963	7569
7613	7613	7614	7612	6670	2228	2020	1948	7624
7560	7560	7561	7559	6792	2137	2066	332	7586
7886	7886	7887	7885	60	6842	6713	6809	7739
383	383	384	382	7932	7666	7720	7651	3359
7614	7614	7615	7613	6752	2062	2053	1918	7647
7575	7575	7576	7574	6850	128	2386	2078	7632
2	2	3	1	7880	7565	7681	7552	3422
3	3	4	2	7881	7566	7682	7553	3423
7698	7698	7697	7697	6886	1913	2453	2255	7760
7579	7579	7580	7578	6698	1885	2002	2041	7589
7612	7612	7613	7611	6728	2083	2052	1937	7647
3411	3411	3412	3410	7698	7614	7635	7623	2340
7580	7580	7581	7579	6859	137	2383	2097	7635
7563	7563	7564	7562	6770	2464	624	2110	7554
2051	2051	2052	2050	7956	7753	7822	7749	3471
3409	3409	3410	3408	7949	7679	7742	7654	2517
7766	7766	7767	7765	473	6854	6849	6818	7653
7602	7602	7603	7601	6695	2215	2020	1914	7614
7554	7554	7555	7553	6816	2092	2054	2	7605
263	263	264	262	7923	7702	7743	7691	3302
6	6	7	5	7884	7569	7685	7556	3426
285	285	286	284	7926	7706	7751	7697	3290
284	284	285	283	7925	7705	7750	7696	3289
288	288	289	287	7929	7709	7754	7700	3293
258	258	259	257	7920	7699	7740	7688	3301
7704	7704	7705	7703	6817	2066	2498	2134	7714
3425	3425	3426	3424	7734	7643	7608	7604	40
7739	7739	7740	7738	6761	2372	769	2139	7681
3414	3414	3415	3413	7732	7660	7684	7668	2303
7584	7584	7585	7583	6857	164	2370	2090	7641
7682	7682	7683	7681	6805	2198	308	1873	7652
7691	7691	7692	7690	6814	2268	389	1952	7642
7705	7705	7706	7704	6847	2229	526	1947	7692
7552	7552	7553	7551	6809	2087	2051	19	7605
7728	7728	7729	7727	6845	2236	490	1968	7696
288	288	289	287	7929	7709	7754	7700	3293
7907	7907	7908	7906	227	6762	6666	6728	7770
7875	7875	7876	7874	34	6834	6728	6810	7728
7674	7674	7675	7673	6800	2191	319	1889	7645
7516	7516	7517	7515	6789	3645	3832	3574	7571
7514	7514	7515	7513	6702	2229	1979	1849	7523
7497	7497	7498	7496	6815	3709	3783	3575	7581
259	259	260	258	7919	7697	7738	7686	3300
7522	7522	7523	7521	6831	3751	3839	3659	7592
8005	8005	8006	8004	368	6965	6884	6940	7856
7662	7662	7663	7661	6780	2109	531	1804	7635

268	268	269	267	7923	7703	7745	7692	3301
7724	7724	7725	7723	6839	2231	485	1963	7694
7680	7680	7681	7679	6816	2261	444	1995	7664
7449	7449	7450	7448	6798	3691	3788	3630	7536
7968	7968	7969	7967	363	6936	6848	6904	7834
5	5	6	4	7883	7566	7682	7553	3425
291	291	292	290	7932	7712	7757	7703	3296
3415	3415	3416	3414	7706	7623	7640	7630	2342
7725	7725	7726	7724	6819	2229	529	1995	7681
7567	7567	7568	7566	6781	2083	1920	641	7591
2044	2044	2045	2043	7949	7748	7817	7744	3464
7656	7656	7657	7655	6818	1685	2291	2120	7698
7723	7723	7724	7722	6826	2233	453	1963	7685
7703	7703	7704	7702	6823	2277	398	1964	7656
7904	7904	7905	7903	224	6759	6663	6725	7767
7492	7492	7493	7491	6804	3685	3824	3632	7571
3416	3416	3417	3415	7734	7662	7686	7670	2305
7693	7693	7694	7692	6816	2270	391	1954	7644
7571	7571	7572	7570	6846	194	2374	2092	7624
7556	7556	7557	7555	6816	2092	2054	2	7607
7563	7563	7564	7562	6775	1923	1910	1738	7606
7719	7719	7720	7718	6821	2236	498	1990	7677
7593	7593	7594	7592	6757	2102	2049	1953	7634
7541	7541	7542	7540	6826	2082	2082	116	7588
7879	7879	7880	7878	36	6844	6736	6812	7732
3421	3421	3422	3420	7739	7666	7690	7674	2310
5	5	6	2	7883	7568	7684	7555	3425
7905	7905	7906	7904	225	6760	6664	6726	7768
7868	7868	7869	7867	47	6819	6731	6793	7717
7906	7906	7907	7905	226	6761	6665	6727	7769
7469	7469	7470	7468	6779	3617	3814	3550	7544
7754	7754	7755	7753	6778	2390	787	2155	7696
7719	7719	7720	7718	6812	2206	450	1940	7687
7658	7658	7659	7657	6817	1946	2314	1952	7653
7671	7671	7672	7670	6780	1921	2457	2172	7706
7581	7581	7582	7580	6834	3794	3920	3717	7618
7581	7581	7582	7580	6834	3794	3920	3717	7618
3408	3408	3409	3407	7695	7611	7632	7620	2337
7615	7615	7616	7614	6687	2211	2017	1911	7614
7513	7513	7514	7512	6836	3749	3865	3673	7595
7530	7530	7531	7529	6787	2094	2008	461	7566
7497	7497	7498	7496	6816	3674	3869	3606	7579
7675	7675	7676	7674	6796	2208	314	1881	7648
7537	7537	7538	7536	6804	3784	3855	3702	7608
7678	7678	7679	7677	6736	2308	369	2009	7632
7888	7888	7889	7887	200	6832	6823	6803	7746
7517	7517	7518	7516	6703	2228	1978	1850	7526
7897	7897	7898	7896	20	6866	6752	6828	7750
7559	7559	7560	7558	6752	1912	1876	1806	7612
7500	7500	7501	7499	6775	3694	3807	3590	7560
7572	7572	7573	7571	6784	1932	1919	1747	7615
7489	7489	7490	7488	6772	3659	3779	3536	7538
	4	5	3	7882	7567	7683	7554	3424
4		5	3	7882	7567	7683	7554	3424
5	5		4	7883	7568	7684	7555	3425

3	3	4		7881	7566	7682	7553	3423
7882	7882	7883	7881		6852	6738	6814	7735
7567	7567	7568	7566	6852		2396	2090	7644
7683	7683	7684	7682	6738	2396		2052	7609
7554	7554	7555	7553	6814	2090	2052		7605
3424	3424	3425	3423	7735	7644	7609	7605	
7688	7688	7689	7687	6803	2338	402	2004	7644
7561	7561	7562	7560	6765	2080	1977	437	7586
7559	7559	7560	7558	6753	1935	1943	1789	7619
7892	7892	7893	7891	108	6812	6724	6780	7747
7481	7481	7482	7480	6827	3675	3875	3628	7583
5	5	4	4	7882	7567	7683	7554	3425
3	3	4	2	7881	7566	7682	7553	3423
322	322	323	321	7873	7556	7696	7571	3381
6	6	5	5	7883	7568	7684	7555	3426
6	6	5	5	7883	7568	7684	7555	3426
7584	7584	7585	7583	6833	203	2365	2149	7645
7676	7676	7677	7675	6731	2389	13	2045	7602
7728	7728	7729	7727	6762	2353	768	2100	7672
7614	7614	7615	7613	6682	2205	2025	1935	7613
7599	7599	7600	7598	6678	2205	1995	1912	7599
7556	7556	7557	7555	6748	1930	1938	1786	7616
7559	7559	7560	7558	6754	1936	1944	1790	7620
7575	7575	7576	7574	6848	122	2390	2082	7634
7612	7612	7613	7611	6752	2064	2051	1918	7645
7604	7604	7605	7603	6744	2056	2043	1910	7637
7533	7533	7534	7532	6683	2324	2064	2053	7568
7681	7681	7682	7680	6792	2335	407	2001	7639
7569	7569	7570	7568	6691	2032	2027	1814	7581
7556	7556	7557	7555	6764	2046	1953	473	7576
7528	7528	7529	7527	6750	1870	1883	1735	7586
7499	7499	7500	7498	6773	3736	3800	3628	7556
7887	7887	7888	7886	195	6835	6824	6804	7745
7497	7497	7498	7496	6774	3691	3805	3587	7558
7716	7716	7717	7715	6818	2234	496	1988	7674
3499	3499	3500	3498	7767	7636	7667	7612	2624
7497	7497	7498	7496	6774	3691	3805	3587	7558
7699	7699	7700	7698	6820	2204	341	1914	7669
7469	7469	7470	7468	6783	3619	3814	3550	7546
7662	7662	7663	7661	6780	2109	531	1804	7635
7654	7654	7655	7653	6799	2202	305	1882	7634
7693	7693	7694	7692	6823	2223	489	1949	7673
7568	7568	7569	7567	6686	2027	2022	1809	7578
8007	8007	8008	8006	385	6955	6942	6935	7871
7742	7742	7743	7741	6768	2386	785	2147	7688
7513	7513	7514	7512	6762	3650	3821	3569	7565
7647	7647	7648	7646	6818	1634	2224	2028	7690
7780	7780	7779	7779	6920	3779	3736	3278	7761
7549	7549	7550	7548	6741	1901	1865	1795	7602
7577	7577	7578	7576	6694	1885	1992	2045	7586
3500	3500	3501	3499	7768	7637	7668	7613	2625
7686	7686	7687	7685	6788	2258	355	1902	7645
7724	7724	7725	7723	6825	2210	520	1966	7685
3371	3371	3372	3370	7734	7605	7619	7602	2310
7533	7533	7534	7532	6810	3723	3850	3648	7580

7522	7522	7523	7521	6788	3646	3840	3569	7597
7568	7568	7569	7567	6779	2073	1916	639	7592
7672	7672	7673	7671	6787	2324	389	1991	7628
3498	3498	3499	3497	7766	7635	7666	7611	2623
7489	7489	7490	7488	6790	3658	3876	3594	7552
7489	7489	7490	7488	6790	3658	3876	3594	7552
3362	3362	3363	3361	7725	7596	7610	7593	2301
7603	7603	7604	7602	6743	2055	2042	1909	7636
7558	7558	7559	7557	6767	2048	1955	475	7578
7551	7551	7552	7550	6821	3686	3755	3593	7589
7442	7442	7443	7441	6802	3757	3783	3653	7529
7520	7520	7521	7519	6742	1862	1875	1727	7578
7573	7573	7574	7572	6691	2032	2027	1814	7583
7693	7693	7694	7692	6825	2220	514	1953	7662
7708	7708	7709	7707	6814	2020	2466	2114	7719
258	258	259	257	7918	7697	7738	7686	3297
7691	7691	7692	7690	6813	2290	659	2041	7646
3498	3498	3499	3497	7766	7635	7666	7611	2623
3497	3497	3498	3496	7765	7634	7665	7610	2622
7573	7573	7574	7572	6846	120	2388	2080	7632
7530	7530	7531	7529	6752	2100	1950	558	7570
7874	7874	7875	7873	119	6823	6735	6792	7741
7567	7567	7568	7566	6852	6	2396	2090	7644
7567	7567	7568	7566	6852	6	2396	2090	7644
3499	3499	3500	3498	7767	7636	7667	7612	2624
7692	7692	7693	7691	6813	2197	332	1907	7662
7678	7678	7679	7677	6797	2208	314	1882	7651
7568	7568	7569	7567	6686	2027	2022	1809	7578
7500	7500	7501	7499	6818	3677	3871	3609	7583
7537	7537	7538	7536	6822	3720	3847	3646	7585
3426	3426	3427	3425	7735	7644	7609	7605	41
7554	7554	7555	7553	6745	1928	1936	1784	7614
7578	7578	7579	7577	6694	1905	2000	2057	7590
7540	7540	7541	7539	6825	2081	2081	115	7587
7616	7616	7617	7615	6758	2070	2057	1924	7651
7543	7543	7544	7542	6810	2141	1950	1831	7582
7541	7541	7542	7540	6808	2139	1948	1829	7580
7570	7570	7571	7569	6836	2170	1979	1860	7608
7904	7904	7905	7903	245	6842	6837	6831	7765
7479	7479	7480	7478	6799	3773	3823	3671	7546
7604	7604	7605	7603	6744	2055	2043	1910	7637
7674	7674	7675	7673	6805	1918	2456	2117	7692
3363	3363	3364	3362	7726	7597	7611	7594	2302
3433	3433	3434	3432	7751	7679	7703	7687	2322
7534	7534	7535	7533	6804	3704	3807	3602	7599
7568	7568	7569	7567	6738	1927	1843	1788	7607
3414	3414	3415	3413	7956	7684	7747	7659	2519
7576	7576	7577	7575	6849	123	2391	2083	7635
7582	7582	7583	7581	6855	129	2397	2089	7641
7540	7540	7541	7539	6765	1887	1906	1756	7589
7712	7712	7713	7711	6817	2230	442	1958	7676
7899	7899	7900	7898	82	6880	6778	6840	7752
7551	7551	7552	7550	6772	2120	1975	571	7588
7859	7859	7860	7858	302	6808	6792	6775	7704
7675	7675	7676	7674	6801	2247	321	1922	7648

7672	7672	7673	7671	6798	2244	318	1919	7645
7695	7695	7696	7694	6800	2008	2442	2072	7699
7559	7559	7560	7558	6752	1912	1876	1806	7612
3424	3424	3425	3423	7733	7642	7607	7603	39
3425	3425	3426	3424	7734	7643	7608	7604	40
3411	3411	3412	3410	7951	7681	7744	7656	2516
7607	7607	7608	7606	6673	2216	2012	1922	7615

GN03624	KCJ3858	CFSAN045100	AZ-TG-WCHI-3	IEH-NGS-ECO-00205	F283	HS115	J21
3779	3583	3589	6772	841	7532	7531	7515
1980	1969	1771	6650	3587	7579	7578	7547
2016	451	1814	6790	3617	7540	7539	7557
3888	3838	3786	6992	3396	7768	7767	7797
3711	3497	3477	6731	385	7480	7479	7451
25034	24991	25020	25033	24930	25533	25532	25544
1982	1696	1603	6694	3599	7604	7603	7570
1952	1877	1856	6658	3584	7616	7615	7581
2060	1910	1947	6766	3617	7581	7580	7556
25033	24990	25019	25032	24929	25532	25531	25543
263	1794	1732	6782	3777	7679	7678	7596
2410	2208	2143	6835	3720	7699	7698	7690
7642	7584	7617	7745	7581	3425	3423	3381
1947	1776	1919	6659	3504	7504	7503	7540
2027	1854	1845	6767	3580	7550	7549	7550
6995	6894	6884	431	6995	8010	8009	7994
1832	1753	314	6700	3571	7515	7514	7500
2478	2006	2200	6764	3700	7702	7701	7676
2478	2006	2200	6764	3700	7702	7701	7676
2001	1768	1750	6814	3646	7618	7617	7622
3680	3371	3534	6958	4226	7791	7790	7797
2331	2079	1928	6807	3736	7574	7573	7563
7760	7706	7701	7804	7615	3524	3522	3493
1888	1802	1472	6770	3595	7541	7540	7528
498	1893	1825	6807	3758	7719	7718	7642
261	1833	1781	6781	3760	7677	7676	7594
7680	7620	7617	7694	7547	3433	3431	3393
2006	1861	1944	6749	3610	7651	7650	7575
7729	7662	7700	7968	7672	3413	3411	3375
7729	7662	7700	7968	7672	3413	3411	3375
1968	1764	1743	6658	3441	7574	7573	7508
31009	31010	30944	30799	30917	31137	31136	31134
7767	7691	7690	7930	7588	262	260	239
7879	7783	7759	8004	7728	2066	2064	2066
3680	3535	3480	6771	1157	7542	7541	7521
3680	3535	3480	6771	1157	7542	7541	7521
7680	7596	7626	7780	7550	3502	3500	3463
7845	7764	7739	7938	7670	2146	2145	2128
42	1909	1885	6780	3767	7682	7681	7678
492	1889	1813	6801	3760	7719	7718	7642
2332	2080	1929	6808	3737	7575	7574	7564
3743	3613	3588	6782	538	7482	7481	7490
7698	7597	7598	7855	7499	814	812	720
6807	6769	6751	265	6799	7893	7892	7871
6795	6757	6737	94	6807	7875	7874	7866
3682	3511	3533	6719	745	7502	7501	7505
411	1986	1952	6733	3884	7692	7691	7705
6825	6787	6767	124	6836	7904	7903	7895
2229	2015	2208	6923	3752	7753	7752	7789
7698	7607	7596	7841	7497	879	877	785
6797	6741	6715	22	6793	7903	7902	7894
7687	7560	7558	7891	7480	2	4	323
2337	2085	1934	6813	3741	7580	7579	7569
2098	2015	2065	6758	3682	7600	7599	7636

1980	1888	1857	6674	3537	7602	7601	7588
1921	1824	1937	6687	3541	7529	7528	7565
1873	1725	373	6744	3635	7572	7571	7541
1985	1811	1885	6699	3577	7603	7602	7593
7842	7744	7720	7958	7671	2045	2043	2043
7654	7570	7602	7760	7530	3531	3529	3492
2032	387	1847	6758	3601	7558	7557	7571
1946	1876	1845	6640	3571	7606	7605	7575
1954	1912	1899	6627	3545	7574	7573	7602
1975	1891	1826	6636	3541	7613	7612	7579
2032	387	1847	6760	3601	7560	7559	7573
6782	6760	6748	162	6832	7886	7885	7877
7750	7656	7655	7938	7527	384	382	349
1996	1818	1894	6708	3586	7614	7613	7604
2328	2076	1925	6810	3735	7575	7574	7564
7686	7559	7557	7890	7479	3	1	320
7687	7560	7558	7891	7480	4	2	321
2407	2241	2156	6846	3720	7698	7697	7689
1980	1969	1771	6650	3587	7579	7578	7547
1973	1837	1905	6684	3585	7612	7611	7590
7668	7620	7616	7712	7565	3412	3410	3368
2341	2095	1918	6819	3744	7580	7579	7569
595	2023	2019	6786	3801	7563	7562	7556
7847	7749	7725	7965	7678	2052	2050	2050
7726	7659	7697	7963	7669	3410	3408	3372
6823	6779	6762	524	6800	7766	7765	7744
1977	1869	1829	6661	3548	7602	7601	7565
2006	439	1791	6782	3630	7554	7553	7571
7770	7694	7693	7933	7591	264	262	241
7690	7563	7561	7894	7483	7	5	324
7778	7700	7699	7937	7595	286	284	263
7777	7699	7698	7936	7594	285	283	262
7781	7703	7702	7940	7598	289	287	266
7767	7691	7690	7930	7587	259	257	236
2469	2019	2184	6787	3688	7704	7703	7682
7643	7585	7618	7746	7582	3426	3424	3382
751	2051	1993	6745	3838	7739	7738	7662
7719	7665	7658	7742	7606	3415	3413	3371
2320	2088	1931	6817	3746	7584	7583	7573
264	1794	1733	6785	3778	7682	7681	7599
149	1847	1800	6798	3766	7691	7690	7608
454	1898	1798	6827	3792	7705	7704	7645
2007	442	1796	6777	3628	7552	7551	7569
434	1895	1811	6825	3797	7728	7727	7653
7781	7703	7702	7940	7598	289	287	266
6731	6677	6655	213	6654	7907	7906	7898
6793	6761	6739	94	6813	7875	7874	7866
260	1802	1750	6780	3774	7674	7673	7591
3740	3559	3537	6747	374	7516	7515	7522
1960	1789	1932	6670	3516	7514	7513	7550
3722	3532	3540	6773	886	7497	7496	7497
7765	7689	7688	7929	7586	260	258	237
3798	3644	3613	6785	499	7522	7521	7567
6990	6889	6879	426	6990	8005	8004	7989
493	1688	1674	6754	3774	7662	7661	7579

7772	7696	7694	7933	7593	269	267	246
429	1890	1806	6819	3792	7724	7723	7649
438	1922	1888	6802	3741	7680	7679	7685
3780	3611	3587	6754	304	7449	7448	7520
6933	6861	6848	431	6948	7968	7967	7980
7687	7560	7558	7893	7480	6	4	323
7784	7706	7705	7943	7601	292	290	269
7673	7627	7621	7717	7572	3416	3414	3372
515	1894	1822	6803	3757	7725	7724	7648
1886	711	1697	6739	3633	7567	7566	7558
7842	7744	7720	7958	7671	2045	2043	2043
2246	2107	2079	6790	3758	7656	7655	7652
449	1890	1804	6806	3769	7723	7722	7648
176	1859	1809	6807	3787	7703	7702	7620
6728	6674	6652	210	6651	7904	7903	7895
3802	3607	3571	6757	404	7492	7491	7487
7721	7667	7660	7744	7608	3417	3415	3373
151	1849	1802	6800	3768	7693	7692	7610
2331	2090	1943	6806	3725	7571	7570	7580
2006	439	1791	6782	3630	7556	7555	7573
1864	1716	364	6735	3626	7563	7562	7532
492	1889	1813	6801	3760	7719	7718	7642
2017	1849	1926	6710	3546	7593	7592	7583
2036	495	1809	6798	3609	7541	7540	7558
6801	6763	6743	100	6813	7879	7878	7870
7725	7671	7664	7749	7612	3422	3420	3378
7689	7562	7560	7893	7482	6	4	323
6729	6675	6653	211	6652	7905	7904	7896
6796	6744	6720	87	6798	7868	7867	7859
6730	6676	6654	212	6653	7906	7905	7897
3685	3533	3498	6732	191	7469	7468	7444
769	2067	2009	6760	3854	7754	7753	7677
412	1867	1783	6798	3790	7719	7718	7644
2281	1853	2025	6775	3657	7658	7657	7632
2426	2114	2065	6736	3792	7671	7670	7645
3865	3702	3693	6784	623	7581	7580	7606
3865	3702	3693	6784	623	7581	7580	7606
7665	7617	7613	7709	7562	3409	3407	3365
1966	1862	1829	6651	3578	7615	7614	7580
3825	3650	3611	6785	477	7513	7512	7522
1952	376	1781	6755	3605	7530	7529	7540
3738	3589	3551	6767	163	7497	7496	7472
258	1794	1740	6780	3770	7675	7674	7592
3763	3658	3599	6766	1314	7537	7536	7550
202	1920	1882	6715	3759	7678	7677	7617
6800	6762	6744	262	6798	7888	7887	7866
1959	1788	1931	6671	3514	7517	7516	7553
6817	6779	6767	124	6841	7897	7896	7888
1824	1757	551	6712	3603	7559	7558	7549
3727	3564	3541	6727	845	7500	7499	7473
1873	1725	373	6744	3635	7572	7571	7541
3711	3519	3490	6724	504	7489	7488	7470
7688	7561	7559	7892	7481	5	3	322
7688	7561	7559	7892	7481	5	3	322
7689	7562	7560	7893	7482	4	4	323

7687	7560	7558	7891	7480	4	2	321
6803	6765	6753	108	6827	7882	7881	7873
2338	2080	1935	6812	3675	7567	7566	7556
402	1977	1943	6724	3875	7683	7682	7696
2004	437	1789	6780	3628	7554	7553	7571
7644	7586	7619	7747	7583	3425	3423	3381
	1917	1897	6787	3776	7688	7687	7684
1917		1761	6739	3609	7561	7560	7574
1897	1761		6713	3570	7559	7558	7546
6787	6739	6713		6780	7892	7891	7883
3776	3609	3570	6780		7481	7480	7485
7688	7561	7559	7892	7481		4	323
7687	7560	7558	7891	7480	4		321
7684	7574	7546	7883	7485	323	321	
7689	7562	7560	7893	7482	3	5	324
7689	7562	7560	7893	7482	3	5	324
2323	2141	1990	6793	3730	7584	7583	7573
395	1970	1936	6717	3868	7676	7675	7689
746	2030	1984	6744	3863	7728	7727	7651
1980	1874	1847	6654	3572	7614	7613	7579
1936	1855	1831	6644	3551	7599	7598	7564
1892	1758	23	6708	3567	7556	7555	7543
1898	1762	9	6714	3571	7559	7558	7546
2332	2080	1929	6808	3737	7575	7574	7564
1994	1816	1894	6708	3586	7612	7611	7602
1986	1810	1886	6700	3578	7604	7603	7594
2029	2004	1973	6651	3533	7533	7532	7579
37	1914	1894	6776	3770	7681	7680	7677
1967	1763	1742	6657	3440	7569	7568	7503
1893	74	1737	6738	3609	7556	7555	7567
1837	1717	261	6710	3598	7528	7527	7515
3776	3619	3601	6725	570	7499	7498	7534
6801	6763	6745	259	6793	7887	7886	7865
3725	3561	3538	6726	842	7497	7496	7470
490	1887	1811	6798	3758	7716	7715	7639
7678	7594	7624	7778	7548	3500	3498	3461
3725	3561	3538	6726	842	7497	7496	7470
289	1825	1768	6806	3763	7699	7698	7616
3685	3533	3498	6736	193	7469	7468	7444
493	1688	1674	6754	3774	7662	7661	7579
263	1795	1736	6780	3749	7654	7653	7572
439	1888	1822	6803	3768	7693	7692	7624
1962	1758	1737	6652	3435	7568	7567	7502
6938	6884	6868	449	6916	8007	8006	8003
757	2071	2009	6752	3845	7742	7741	7667
3730	3548	3528	6718	458	7513	7512	7489
2183	2023	2012	6782	3762	7647	7646	7635
3669	3360	3523	6948	4215	7780	7779	7786
1813	1746	540	6701	3592	7549	7548	7539
1972	1965	1749	6646	3588	7577	7576	7545
7679	7595	7625	7779	7549	3501	3499	3462
435	1829	1807	6763	3801	7686	7685	7624
470	1865	1797	6811	3784	7724	7723	7647
7654	7599	7595	7745	7543	3372	3370	3328
3795	3631	3603	6760	475	7533	7532	7558

3717	3560	3510	6744	250	7522	7521	7497
1892	707	1691	6743	3634	7568	7567	7559
19	1904	1884	6771	3757	7672	7671	7668
7677	7593	7623	7777	7547	3499	3497	3460
3748	3583	3534	6744	195	7489	7488	7464
3748	3583	3534	6744	195	7489	7488	7464
7645	7590	7586	7736	7534	3363	3361	3319
1985	1809	1885	6699	3577	7603	7602	7593
1895	76	1739	6741	3609	7558	7557	7571
3684	3535	3472	6777	1170	7551	7550	7530
3786	3632	3604	6757	412	7442	7441	7513
1829	1709	253	6702	3590	7520	7519	7507
1967	1763	1742	6657	3440	7573	7572	7507
487	1870	1809	6806	3756	7693	7692	7616
2433	1999	2156	6786	3678	7708	7707	7682
7765	7689	7688	7928	7586	259	257	236
610	1952	1859	6826	3846	7691	7690	7614
7677	7593	7623	7777	7547	3499	3497	3460
7676	7592	7622	7776	7546	3498	3496	3459
2330	2078	1927	6806	3735	7573	7572	7562
1872	181	1821	6728	3598	7530	7529	7569
6797	6745	6722	26	6769	7874	7873	7865
2338	2080	1935	6812	3675	7567	7566	7556
2338	2080	1935	6812	3675	7567	7566	7556
7678	7594	7624	7778	7548	3500	3498	3461
280	1818	1759	6799	3756	7692	7691	7609
260	1795	1741	6781	3773	7678	7677	7595
1962	1758	1737	6652	3435	7568	7567	7502
3740	3592	3554	6769	165	7500	7499	7476
3792	3628	3600	6772	474	7537	7536	7562
7644	7586	7619	7747	7583	3427	3425	3383
1890	1756	21	6705	3565	7554	7553	7541
1986	1979	1787	6650	3588	7578	7577	7546
2035	494	1808	6797	3608	7540	7539	7557
2000	1824	1900	6714	3592	7616	7615	7606
1890	1804	1474	6772	3597	7543	7542	7530
1888	1802	1472	6770	3595	7541	7540	7528
1919	1833	1503	6798	3626	7570	7569	7557
6832	6782	6760	279	6803	7904	7903	7898
3784	3633	3585	6750	984	7479	7478	7477
1986	1810	1883	6700	3578	7604	7603	7594
2425	2029	2113	6767	3719	7674	7673	7648
7646	7591	7587	7737	7535	3364	3362	3320
7738	7684	7677	7761	7625	3434	3432	3390
3710	3586	3554	6758	577	7534	7533	7513
1793	1763	453	6698	3603	7568	7567	7523
7731	7664	7702	7970	7674	3415	3413	3377
2333	2081	1930	6809	3738	7576	7575	7565
2339	2087	1936	6815	3744	7582	7581	7571
1862	1738	296	6725	3621	7540	7539	7527
440	1885	1799	6795	3758	7712	7711	7637
6843	6791	6785	150	6883	7899	7898	7890
1885	190	1832	6746	3600	7551	7550	7590
6769	6734	6714	368	6795	7859	7858	7837
259	1831	1780	6779	3758	7675	7674	7592

256	1828	1777	6776	3755	7672	7671	7589
2409	1973	2153	6760	3705	7695	7694	7669
1824	1757	551	6712	3603	7559	7558	7549
7642	7584	7617	7745	7581	3425	3423	3381
7643	7585	7618	7746	7580	3426	3424	3382
7728	7661	7699	7965	7671	3412	3410	3374
1945	1875	1844	6639	3570	7607	7606	7576

KO178B	KO198B	AZ-TG-WCHI-8	AZ-TG-713-2	KCJ9492	MOD1-EC5105	MOD1-EC5111
7533	7533	3762	3841	3830	3547	3520
7580	7580	1902	1995	1952	1945	1933
7541	7541	2158	2054	2123	1950	1929
7769	7769	3848	3958	4033	3831	3814
7481	7481	3662	3764	3761	3465	3456
25534	25534	24963	25029	25040	24984	24971
7605	7605	2096	2001	1982	1769	1759
7617	7617	2233	2011	2126	75	107
7582	7582	2240	2085	2128	1778	1769
25533	25533	24962	25028	25039	24983	24970
7680	7680	2184	292	539	1845	1815
7700	7700	1864	2453	2470	2427	2417
3426	3426	7643	7600	7670	7611	7597
7505	7505	2226	1959	2064	1803	1791
7551	7551	2029	2069	2123	2021	2033
8011	8011	6951	6882	6935	6849	6845
7516	7516	1957	1867	1943	1804	1788
7703	7703	1968	2510	2433	2363	2363
7703	7703	1968	2510	2433	2363	2363
7619	7619	2287	2051	2116	1984	1968
7792	7792	3791	3740	3696	3507	3489
7575	7575	92	2382	2346	2184	2184
3525	3525	7731	7762	7807	7708	7695
7542	7542	2074	1941	1966	1855	1840
7720	7720	2229	503	364	1961	1927
7678	7678	2234	316	609	1891	1843
3434	3434	7639	7636	7718	7637	7621
7652	7652	2193	2039	2090	2043	2025
3414	3414	7708	7738	7791	7695	7681
3414	3414	7708	7738	7791	7695	7681
7575	7575	2174	2021	2038	1786	1772
31138	31138	30952	31001	31025	31012	30991
263	263	7716	7733	7805	7715	7700
2067	2067	7807	7857	7922	7792	7782
7543	7543	3678	3740	3727	3468	3449
7543	7543	3678	3740	3727	3468	3449
3503	3503	7669	7662	7719	7620	7605
2147	2147	7791	7819	7881	7754	7740
7683	7683	2306	379	728	1959	1922
7720	7720	2221	491	358	1947	1915
7576	7576	93	2383	2347	2185	2185
7483	7483	3779	3854	3903	3624	3606
815	815	7633	7695	7727	7621	7608
7894	7894	6822	6823	6776	6676	6672
7876	7876	6819	6723	6754	6674	6670
7503	7503	3710	3716	3749	3497	3480
7693	7693	2374	22	777	2034	2004
7905	7905	6849	6753	6784	6703	6700
7754	7754	2492	2271	2336	2147	2131
880	880	7637	7695	7727	7621	7608
7904	7904	6787	6711	6752	6656	6652
3	3	7583	7675	7727	7613	7598
7581	7581	98	2388	2352	2190	2190
7601	7601	2401	2110	2117	2053	2039

7603	7603	2233	2002	2135	180	164
7530	7530	2229	1953	2044	1859	1846
7573	7573	1989	1912	1947	1811	1795
7604	7604	2145	2035	2067	1936	1918
2046	2046	7773	7810	7889	7752	7742
3532	3532	7643	7636	7693	7598	7583
7559	7559	2204	2059	2088	1889	1865
7607	7607	2218	2006	2131	130	104
7575	7575	2266	1961	2175	234	208
7614	7614	2203	2013	2130	177	151
7561	7561	2204	2059	2088	1889	1865
7887	7887	6821	6706	6739	6663	6657
385	385	7683	7713	7774	7683	7668
7615	7615	2154	2046	2078	1947	1929
7576	7576	99	2379	2343	2181	2181
4	4	7582	7674	7726	7612	7597
5	5	7583	7675	7727	7613	7598
7699	7699	1917	2446	2449	2443	2439
7580	7580	1902	1995	1952	1945	1933
7613	7613	2173	2045	2087	1950	1932
3413	3413	7635	7628	7716	7629	7613
7581	7581	108	2376	2362	2194	2194
7564	7564	2443	617	977	2086	2060
2053	2053	7778	7815	7894	7757	7747
3411	3411	7705	7735	7788	7692	7678
7767	7767	6837	6842	6787	6696	6688
7603	7603	2216	2013	2110	172	146
7555	7555	2151	2047	2102	1937	1914
265	265	7719	7736	7808	7718	7703
8	8	7586	7678	7730	7616	7601
287	287	7723	7744	7816	7723	7708
286	286	7722	7743	7815	7722	7707
290	290	7726	7747	7819	7726	7711
260	260	7716	7733	7805	7715	7700
7705	7705	2045	2491	2474	2358	2352
3427	3427	7644	7601	7671	7612	7598
7740	7740	2371	762	73	2138	2112
3416	3416	7692	7677	7765	7677	7661
7585	7585	135	2363	2327	2193	2181
7683	7683	2193	301	546	1855	1827
7692	7692	2253	382	641	1912	1869
7706	7706	2216	519	474	1930	1898
7553	7553	2148	2044	2101	1928	1901
7729	7729	2223	483	436	1949	1917
290	290	7726	7747	7819	7726	7711
7908	7908	6743	6659	6692	6600	6596
7876	7876	6815	6721	6752	6678	6674
7675	7675	2184	312	561	1847	1826
7517	7517	3712	3825	3815	3522	3513
7515	7515	2239	1972	2077	1816	1804
7498	7498	3704	3776	3767	3507	3479
261	261	7714	7731	7803	7713	7698
7523	7523	3756	3832	3881	3613	3617
8006	8006	6946	6877	6930	6844	6840
7663	7663	2110	524	687	1801	1783

270	270	7720	7738	7810	7719	7704
7725	7725	2218	478	431	1944	1912
7681	7681	2236	437	523	1969	1935
7450	7450	3748	3781	3875	3585	3556
7969	7969	6917	6841	6889	6810	6806
7	7	7583	7675	7727	7613	7598
293	293	7729	7750	7822	7729	7714
3417	3417	7644	7633	7721	7634	7618
7726	7726	2218	522	359	1956	1922
7568	7568	2080	1913	1953	1860	1836
2046	2046	7773	7810	7889	7752	7742
7657	7657	1629	2284	2292	2265	2267
7724	7724	2222	446	433	1946	1924
7704	7704	2262	391	650	1921	1892
7905	7905	6740	6656	6689	6597	6593
7493	7493	3736	3817	3858	3567	3557
3418	3418	7694	7679	7767	7679	7663
7694	7694	2255	384	643	1914	1871
7572	7572	165	2367	2357	2197	2197
7557	7557	2151	2047	2102	1937	1914
7564	7564	1980	1903	1938	1802	1786
7720	7720	2221	491	358	1947	1915
7594	7594	2176	2042	2110	1977	1949
7542	7542	2145	2075	2104	1957	1934
7880	7880	6825	6729	6760	6680	6676
3423	3423	7698	7683	7771	7683	7667
7	7	7585	7677	7729	7615	7600
7906	7906	6741	6657	6690	6598	6594
7869	7869	6800	6724	6755	6657	6653
7907	7907	6742	6658	6691	6599	6595
7470	7470	3672	3807	3776	3478	3455
7755	7755	2389	780	85	2156	2130
7720	7720	2183	443	410	1917	1883
7659	7659	1864	2307	2312	2229	2231
7672	7672	1935	2450	2429	2357	2351
7582	7582	3832	3913	3951	3655	3636
7582	7582	3832	3913	3951	3655	3636
3410	3410	7632	7625	7713	7626	7610
7616	7616	2218	2010	2119	108	92
7514	7514	3792	3858	3901	3616	3607
7531	7531	2153	2001	2054	1959	1940
7498	7498	3727	3862	3833	3531	3502
7676	7676	2191	307	568	1855	1833
7538	7538	3779	3848	3860	3612	3589
7679	7679	2291	362	735	1952	1911
7889	7889	6813	6816	6769	6669	6665
7518	7518	2238	1971	2076	1815	1803
7898	7898	6847	6745	6776	6696	6692
7560	7560	1969	1869	1929	1865	1857
7501	7501	3689	3800	3797	3486	3447
7573	7573	1989	1912	1947	1811	1795
7490	7490	3662	3772	3756	3509	3496
6	6	7584	7676	7728	7614	7599
6	6	7584	7676	7728	7614	7599
5	5	7585	7677	7729	7615	7600

5	5	7583	7675	7727	7613	7598
7883	7883	6833	6731	6762	6682	6678
7568	7568	203	2389	2353	2205	2205
7684	7684	2365	13	768	2025	1995
7555	7555	2149	2045	2100	1935	1912
3426	3426	7645	7602	7672	7613	7599
7689	7689	2323	395	746	1980	1936
7562	7562	2141	1970	2030	1874	1855
7560	7560	1990	1936	1984	1847	1831
7893	7893	6793	6717	6744	6654	6644
7482	7482	3730	3868	3863	3572	3551
3	3	7584	7676	7728	7614	7599
5	5	7583	7675	7727	7613	7598
324	324	7573	7689	7651	7579	7564
		7585	7677	7729	7615	7600
		7585	7677	7729	7615	7600
7585	7585		2358	2352	2212	2206
7677	7677	2358		761	2018	1988
7729	7729	2352	761		2131	2105
7615	7615	2212	2018	2131		94
7600	7600	2206	1988	2105	94	
7557	7557	1985	1931	1979	1842	1826
7560	7560	1991	1937	1985	1848	1832
7576	7576	93	2383	2347	2185	2185
7613	7613	2154	2044	2076	1945	1927
7605	7605	2146	2036	2068	1937	1919
7534	7534	2327	2057	2247	440	424
7682	7682	2320	400	751	1975	1941
7570	7570	2173	2020	2037	1785	1771
7557	7557	2107	1946	2018	1839	1820
7529	7529	1927	1876	1918	1803	1787
7500	7500	3737	3793	3851	3595	3593
7888	7888	6816	6817	6770	6670	6666
7498	7498	3686	3798	3795	3484	3445
7717	7717	2219	489	356	1945	1913
3501	3501	7667	7660	7717	7618	7603
7498	7498	3686	3798	3795	3484	3445
7700	7700	2207	334	581	1864	1831
7470	7470	3674	3807	3776	3480	3455
7663	7663	2110	524	687	1801	1783
7655	7655	2193	298	550	1855	1831
7694	7694	2210	482	485	1934	1910
7569	7569	2168	2015	2032	1780	1766
8008	8008	6936	6935	6898	6820	6816
7743	7743	2385	778	97	2144	2118
7514	7514	3699	3814	3810	3499	3484
7648	7648	1551	2217	2215	2227	2229
7781	7781	3780	3729	3685	3496	3478
7550	7550	1958	1858	1916	1854	1846
7578	7578	1902	1985	1944	1948	1930
3502	3502	7668	7661	7718	7619	7604
7687	7687	2227	348	680	1946	1916
7725	7725	2201	513	332	1925	1891
3373	3373	7625	7612	7702	7613	7597
7534	7534	3770	3843	3881	3584	3565

7523	7523	3713	3833	3794	3505	3492
7569	7569	2076	1909	1949	1850	1826
7673	7673	2309	382	733	1964	1921
3500	3500	7666	7659	7716	7617	7602
7490	7490	3725	3869	3830	3529	3507
7490	7490	3725	3869	3830	3529	3507
3364	3364	7616	7603	7693	7604	7588
7604	7604	2145	2035	2067	1936	1918
7559	7559	2109	1948	2022	1843	1824
7552	7552	3685	3748	3737	3468	3461
7443	7443	3746	3776	3876	3606	3581
7521	7521	1919	1868	1910	1795	1779
7574	7574	2173	2020	2037	1785	1771
7694	7694	2203	507	320	1943	1909
7709	7709	1999	2459	2440	2307	2309
260	260	7714	7731	7803	7713	7698
7692	7692	2285	652	472	2016	1990
3500	3500	7666	7659	7716	7617	7602
3499	3499	7665	7658	7715	7616	7601
7574	7574	91	2381	2345	2183	2183
7531	7531	2151	1943	2075	1861	1838
7875	7875	6804	6728	6755	6663	6657
7568	7568	203	2389	2353	2205	2205
7568	7568	203	2389	2353	2205	2205
3501	3501	7667	7660	7717	7618	7603
7693	7693	2200	325	572	1855	1822
7679	7679	2191	307	568	1856	1834
7569	7569	2168	2015	2032	1780	1766
7501	7501	3730	3864	3835	3533	3504
7538	7538	3767	3840	3878	3580	3561
3428	3428	7645	7602	7672	7613	7599
7555	7555	1983	1929	1977	1840	1824
7579	7579	1900	1993	1970	1961	1941
7541	7541	2144	2074	2103	1956	1933
7617	7617	2160	2050	2082	1951	1933
7544	7544	2076	1943	1968	1857	1842
7542	7542	2074	1941	1966	1855	1840
7571	7571	2105	1972	1997	1886	1871
7905	7905	6823	6830	6795	6694	6690
7480	7480	3764	3816	3856	3583	3564
7605	7605	2146	2036	2068	1937	1919
7675	7675	1901	2449	2414	2352	2352
3365	3365	7617	7604	7694	7605	7589
3435	3435	7711	7696	7784	7696	7680
7535	7535	3705	3800	3796	3544	3517
7569	7569	1984	1836	1886	1821	1805
3416	3416	7710	7740	7793	7697	7683
7577	7577	94	2384	2348	2186	2186
7583	7583	100	2390	2354	2192	2192
7541	7541	1944	1899	1943	1834	1818
7713	7713	2211	435	430	1945	1909
7900	7900	6861	6771	6802	6716	6712
7552	7552	2181	1968	2092	1871	1847
7860	7860	6789	6785	6738	6646	6636
7676	7676	2232	314	607	1889	1841

7673	7673	2229	311	604	1886	1838
7696	7696	1985	2435	2430	2310	2312
7560	7560	1969	1869	1929	1865	1857
3426	3426	7643	7600	7670	7611	7597
3427	3427	7644	7601	7671	7612	7598
3413	3413	7707	7737	7790	7694	7680
7608	7608	2217	2005	2130	129	103

MOD1-EC5122	CFSAN041116	MOD1-EC5135	MOD1-EC5153	MOD1-EC5167	246164
3586	3590	3761	3532	3524	3518
1766	1772	1887	1958	1950	2051
1811	1815	2105	1946	1938	2072
3781	3787	3835	3795	3787	3758
3474	3478	3671	3495	3487	3474
25016	25018	24971	25041	25035	24986
1600	1604	2075	1931	1923	1870
1851	1857	2216	1954	1946	460
1942	1948	2245	1973	1965	1922
25015	25017	24970	25040	25034	24985
1727	1733	2181	1880	1872	1969
2138	2144	1865	2244	2236	2494
7614	7618	7632	7643	7635	7566
1914	1920	2181	1867	1859	1892
1840	1846	2026	1868	1860	2166
6879	6885	6966	6914	6906	6835
309	315	1898	1865	1857	1906
2197	2201	1976	2236	2228	2448
2197	2201	1976	2236	2228	2448
1747	1751	2262	1964	1956	2021
3529	3535	3807	3557	3549	3560
1923	1929	5	2116	2108	2297
7698	7702	7724	7722	7714	7653
1469	1473	2109	1866	1858	1965
1820	1826	2238	1942	1934	2075
1776	1782	2243	1930	1922	2012
7612	7617	7627	7652	7644	7582
1939	1945	2156	181	173	2132
7697	7701	7697	7691	7683	7641
7697	7701	7697	7691	7683	7641
1738	1744	2127	1751	1743	1870
30938	30944	30946	30983	30975	30998
7689	7692	7707	7703	7695	7666
7756	7761	7792	7786	7778	7748
3477	3481	3681	3515	3507	3418
3477	3481	3681	3515	3507	3418
7623	7627	7656	7657	7649	7565
7736	7742	7780	7776	7768	7713
1880	1886	2313	1982	1974	2011
1808	1814	2230	1944	1936	2063
1924	1930	6	2117	2109	2298
3585	3589	3784	3646	3638	3562
7595	7598	7622	7617	7609	7575
6746	6752	6837	6755	6747	6684
6732	6738	6834	6736	6728	6675
3530	3534	3685	3522	3514	3434
1947	1953	2399	2060	2052	2073
6762	6768	6864	6766	6758	6705
2203	2209	2451	2103	2095	2222
7593	7596	7626	7613	7605	7575
6710	6716	6812	6716	6708	6659
7555	7558	7574	7611	7603	7532
1929	1935	11	2122	2114	2303
2060	2066	2354	2125	2117	2121

1852	1858	2212	1955	1947	437
1932	1938	2178	1930	1922	1915
368	374	1924	1896	1888	1923
1880	1886	2108	33	25	2028
7717	7722	7757	7746	7738	7708
7599	7603	7630	7631	7623	7543
1844	1848	2137	1885	1877	1992
1840	1846	2197	1939	1931	423
1894	1900	2245	1963	1955	407
1821	1827	2182	1964	1956	498
1844	1848	2137	1885	1877	1992
6743	6749	6838	6739	6731	6670
7654	7657	7674	7676	7668	7643
1889	1895	2117	28	34	2039
1920	1926	12	2113	2105	2296
7554	7557	7573	7610	7602	7531
7555	7558	7574	7611	7603	7532
2151	2157	1918	2253	2245	2503
1766	1772	1887	1958	1950	2051
1900	1906	2136	65	57	2042
7611	7617	7623	7650	7642	7578
1913	1919	21	2130	2122	2307
2014	2020	2458	2114	2106	2102
7722	7727	7762	7751	7743	7712
7694	7698	7694	7688	7680	7638
6757	6763	6850	6781	6773	6700
1824	1830	2195	1936	1928	439
1788	1792	2084	1920	1912	2055
7692	7695	7710	7706	7698	7669
7558	7561	7577	7614	7606	7535
7698	7701	7714	7712	7704	7675
7697	7700	7713	7711	7703	7674
7701	7704	7717	7715	7707	7678
7689	7692	7707	7703	7695	7666
2181	2185	2057	2301	2293	2460
7615	7619	7633	7644	7636	7567
1988	1994	2366	2091	2083	2254
7653	7659	7680	7695	7687	7624
1926	1932	48	2109	2101	2299
1728	1734	2192	1883	1875	1981
1795	1801	2262	1952	1944	2034
1793	1799	2223	1923	1915	2036
1793	1797	2079	1923	1915	2044
1806	1812	2230	1942	1934	2063
7701	7704	7717	7715	7707	7678
6650	6656	6758	6668	6660	6601
6734	6740	6830	6738	6730	6679
1745	1751	2185	1900	1892	1978
3534	3538	3707	3549	3541	3523
1927	1933	2194	1880	1872	1905
3537	3541	3709	3572	3564	3431
7687	7690	7705	7701	7693	7664
3610	3614	3751	3626	3618	3569
6874	6880	6961	6909	6901	6830
1669	1675	2103	1804	1796	1927

7693	7696	7711	7709	7701	7670
1801	1807	2225	1937	1929	2058
1883	1889	2253	1944	1936	1957
3584	3588	3753	3594	3586	3535
6843	6849	6932	6872	6864	6779
7555	7558	7574	7611	7603	7532
7704	7707	7720	7718	7710	7681
7616	7622	7632	7655	7647	7583
1817	1823	2223	1951	1943	2070
1694	1698	2083	1885	1877	1987
7717	7722	7757	7746	7738	7708
2074	2080	1678	2074	2066	2325
1799	1805	2227	1941	1933	2066
1804	1810	2271	1961	1953	2046
6647	6653	6755	6665	6657	6598
3568	3572	3741	3601	3593	3438
7655	7661	7682	7697	7689	7626
1797	1803	2264	1954	1946	2036
1938	1944	78	2089	2081	2304
1788	1792	2084	1920	1912	2055
359	365	1915	1887	1879	1914
1808	1814	2230	1944	1936	2063
1921	1927	2155	83	75	2067
1806	1810	2074	1932	1924	2073
6738	6744	6840	6742	6734	6681
7659	7665	7686	7701	7693	7630
7557	7560	7576	7613	7605	7534
6648	6654	6756	6666	6658	6599
6715	6721	6815	6719	6711	6658
6649	6655	6757	6667	6659	6600
3495	3499	3679	3513	3505	3486
2004	2010	2384	2107	2099	2272
1778	1784	2200	1914	1906	2035
2022	2026	1878	2199	2191	2332
2060	2066	1920	2202	2194	2444
3690	3694	3841	3672	3664	3655
3690	3694	3841	3672	3664	3655
7608	7614	7620	7647	7639	7575
1824	1830	2191	1925	1917	437
3608	3612	3805	3654	3646	3574
1778	1782	2092	1874	1866	2077
3548	3552	3736	3566	3558	3542
1735	1741	2202	1891	1883	1981
3596	3600	3784	3651	3643	3570
1877	1883	2302	2017	2009	2067
6739	6745	6828	6748	6740	6677
1926	1932	2193	1879	1871	1904
6762	6768	6862	6766	6758	6697
546	552	1910	1878	1870	1968
3538	3542	3694	3550	3542	3448
368	374	1924	1896	1888	1923
3487	3491	3659	3515	3507	3446
7556	7559	7575	7612	7604	7533
7556	7559	7575	7612	7604	7533
7557	7560	7576	7613	7605	7534

7555	7558	7574	7611	7603	7532
6748	6754	6848	6752	6744	6683
1930	1936	122	2064	2056	2324
1938	1944	2390	2051	2043	2064
1786	1790	2082	1918	1910	2053
7616	7620	7634	7645	7637	7568
1892	1898	2332	1994	1986	2029
1758	1762	2080	1816	1810	2004
23	9	1929	1894	1886	1973
6708	6714	6808	6708	6700	6651
3567	3571	3737	3586	3578	3533
7556	7559	7575	7612	7604	7533
7555	7558	7574	7611	7603	7532
7543	7546	7564	7602	7594	7579
7557	7560	7576	7613	7605	7534
7557	7560	7576	7613	7605	7534
1985	1991	93	2154	2146	2327
1931	1937	2383	2044	2036	2057
1979	1985	2347	2076	2068	2247
1842	1848	2185	1945	1937	440
1826	1832	2185	1927	1919	424
	24	1924	1889	1881	1968
24		1930	1895	1887	1974
1924	1930		2117	2109	2298
1889	1895	2117		32	2037
1881	1887	2109	32		2029
1968	1974	2298	2037	2029	
1889	1895	2329	1991	1983	2024
1737	1743	2126	1750	1742	1869
1734	1738	2046	1806	1800	1971
256	262	1868	1858	1850	1911
3598	3602	3736	3599	3591	3545
6740	6746	6831	6749	6741	6678
3535	3539	3691	3548	3540	3446
1806	1812	2228	1942	1934	2061
7621	7625	7654	7655	7647	7563
3535	3539	3691	3548	3540	3446
1763	1769	2198	1916	1908	1992
3495	3499	3681	3511	3503	3486
1669	1675	2103	1804	1796	1927
1731	1737	2196	1887	1879	1983
1817	1823	2217	1932	1924	2034
1732	1738	2121	1745	1737	1864
6863	6869	6951	6884	6876	6805
2004	2010	2380	2100	2092	2254
3525	3529	3706	3536	3528	3526
2007	2013	1592	2037	2029	2291
3518	3524	3796	3546	3538	3549
535	541	1899	1867	1859	1957
1744	1750	1887	1964	1956	2038
7622	7626	7655	7656	7648	7564
1802	1808	2252	1959	1951	2062
1792	1798	2204	1916	1908	2039
7590	7596	7612	7629	7621	7564
3600	3604	3779	3607	3599	3580

3507	3511	3708	3529	3521	3512
1688	1692	2073	1879	1871	1977
1879	1885	2318	1981	1973	2012
7620	7624	7653	7654	7646	7562
3531	3535	3720	3560	3552	3543
3531	3535	3720	3560	3552	3543
7581	7587	7603	7620	7612	7555
1880	1886	2108	33	25	2028
1736	1740	2048	1808	1802	1975
3469	3473	3686	3513	3505	3424
3601	3605	3757	3619	3611	3556
248	254	1860	1850	1842	1903
1737	1743	2126	1750	1742	1869
1804	1810	2214	1927	1919	2057
2153	2157	2011	2270	2262	2412
7687	7690	7705	7701	7693	7664
1854	1860	2284	1987	1979	2132
7620	7624	7653	7654	7646	7562
7619	7623	7652	7653	7645	7561
1922	1928	4	2115	2107	2296
1818	1822	2100	1859	1853	1971
6717	6723	6819	6722	6714	6662
1930	1936	122	2064	2056	2324
1930	1936	122	2064	2056	2324
7621	7625	7654	7655	7647	7563
1754	1760	2191	1909	1901	1983
1736	1742	2202	1891	1883	1982
1732	1738	2121	1745	1737	1864
3551	3555	3739	3568	3560	3544
3597	3601	3776	3604	3596	3580
7616	7620	7634	7645	7637	7568
16	22	1922	1887	1879	1966
1782	1788	1907	1978	1970	2067
1805	1809	2073	1931	1923	2072
1895	1901	2123	48	40	2043
1471	1475	2111	1868	1860	1967
1469	1473	2109	1866	1858	1965
1500	1504	2140	1897	1889	1996
6755	6761	6838	6762	6754	6692
3582	3586	3773	3623	3615	3552
1878	1884	2108	34	26	2029
2108	2114	1909	2164	2156	2435
7582	7588	7604	7621	7613	7556
7672	7678	7699	7714	7706	7643
3551	3555	3704	3564	3556	3519
448	454	1925	1871	1863	1923
7699	7703	7699	7693	7685	7643
1925	1931	7	2118	2110	2299
1931	1937	13	2124	2116	2305
291	297	1885	1881	1873	1942
1794	1800	2224	1934	1926	2061
6780	6786	6876	6790	6782	6717
1829	1833	2120	1870	1864	1981
6709	6715	6804	6725	6717	6654
1775	1781	2241	1928	1920	2010

1772	1778	2238	1925	1917	2007
2150	2154	1999	2211	2203	2413
546	552	1910	1878	1870	1968
7614	7618	7632	7643	7635	7566
7615	7619	7633	7644	7636	7567
7696	7700	7696	7690	7682	7640
1839	1845	2196	1938	1930	422

ESC_DA2647AA	195745	181089	KCJK2721	MOD1-EC7010	MOD1-EC6900	KCJK4181
3784	3406	3583	3613	1073	6781	683
1977	1865	1939	1703	3610	6706	3578
2013	1838	487	1760	3642	6811	3602
3895	3665	3830	3772	3449	6987	3415
3716	3332	3497	3505	535	6734	806
25026	25011	24982	25005	24927	25018	24923
1979	1447	1696	1519	3612	6719	3567
1957	1794	1842	1812	3595	6680	3493
2057	1849	1912	1895	3627	6792	3544
25025	25010	24981	25004	24926	25017	24922
268	1830	1768	1672	3771	6808	3697
2407	2250	2178	2105	3714	6875	3680
7637	7579	7574	7584	7554	7743	7556
1944	1896	1763	1869	3535	6679	3508
2024	1820	1840	1787	3581	6788	3556
6984	6832	6891	6881	6942	428	6958
1829	1745	1735	75	3611	6736	3557
2475	2102	1988	2141	3692	6806	3624
2475	2102	1988	2141	3692	6806	3624
1998	1691	1735	1710	3702	6825	3637
3676	3508	3338	3546	4224	6908	4167
2328	2125	2045	1867	3735	6830	3690
7753	7674	7695	7680	7605	7793	7611
1883	1616	1776	1424	3625	6803	3539
503	1879	1881	1743	3756	6835	3700
266	1876	1807	1721	3774	6811	3685
7673	7566	7610	7590	7521	7675	7527
2003	1807	1851	1908	3620	6769	3568
7724	7639	7654	7665	7660	7958	7668
7724	7639	7654	7665	7660	7958	7668
1965	25	1736	1709	3502	6688	3439
30998	30971	31009	30924	30950	30787	30910
7760	7636	7686	7661	7597	7920	7570
7872	7742	7778	7731	7742	7992	7724
3685	3393	3543	3508	1069	6778	625
3685	3393	3543	3508	1069	6778	625
7675	7587	7590	7582	7528	7774	7543
7838	7702	7759	7707	7669	7931	7659
47	1957	1885	1825	3759	6792	3707
497	1873	1877	1731	3744	6827	3694
2329	2126	2046	1868	3736	6831	3691
3746	3436	3613	3612	795	6794	1053
7691	7549	7592	7568	7529	7865	7472
6796	6693	6768	6748	6752	10	6749
6784	6681	6756	6734	6753	173	6754
3687	3431	3511	3563	564	6743	942
416	2036	1962	1892	3809	6833	3814
6814	6711	6786	6764	6782	203	6783
2226	2239	2002	2148	3788	6953	3766
7691	7545	7602	7566	7527	7855	7470
6786	6661	6740	6712	6735	263	6736
7680	7568	7555	7527	7498	7886	7496
2334	2131	2051	1873	3740	6836	3695
2095	2149	2006	2030	3705	6792	3691

1975	1793	1853	1813	3584	6692	3500
1918	2008	1811	1885	3580	6714	3534
1870	1705	1707	188	3664	6761	3609
1982	1741	1801	1849	3590	6740	3539
7835	7703	7739	7693	7685	7947	7669
7649	7563	7564	7558	7508	7798	7523
2029	1818	423	1787	3621	6788	3549
1941	1779	1841	1801	3605	6662	3479
1949	1823	1877	1855	3558	6660	3489
1970	1806	1856	1782	3546	6658	3418
2029	1818	423	1787	3621	6790	3549
6787	6684	6759	6745	6741	250	6753
7743	7603	7651	7628	7548	7935	7514
1993	1752	1808	1858	3599	6749	3548
2325	2122	2042	1864	3736	6833	3689
7679	7567	7554	7526	7497	7885	7495
7680	7568	7555	7527	7498	7886	7496
2404	2283	2215	2110	3714	6886	3670
1977	1865	1939	1703	3610	6706	3578
1970	1761	1827	1869	3602	6731	3527
7661	7567	7610	7595	7539	7690	7525
2338	2141	2061	1857	3743	6842	3698
600	2124	1999	1955	3828	6747	3714
7840	7708	7744	7698	7692	7954	7676
7721	7636	7651	7662	7657	7953	7665
6812	6713	6778	6759	6781	286	6779
1972	1755	1834	1787	3579	6674	3485
2003	1816	475	1737	3630	6806	3589
7763	7639	7689	7664	7600	7923	7573
7683	7571	7558	7530	7501	7889	7499
7771	7645	7695	7670	7604	7926	7577
7770	7644	7694	7669	7603	7925	7576
7774	7648	7698	7673	7607	7929	7580
7760	7636	7686	7661	7596	7920	7569
2466	2143	2001	2136	3689	6821	3616
7638	7580	7575	7585	7555	7744	7557
756	2066	2039	1927	3826	6769	3774
7712	7608	7655	7633	7580	7724	7578
2321	2114	2054	1870	3748	6840	3696
269	1831	1768	1673	3773	6813	3705
154	1894	1821	1740	3793	6822	3721
459	1818	1886	1718	3804	6842	3749
2004	1821	478	1742	3629	6799	3580
439	1841	1883	1731	3812	6853	3750
7774	7648	7698	7673	7607	7929	7580
6720	6593	6676	6660	6600	386	6601
6782	6685	6760	6736	6759	181	6760
265	1846	1776	1690	3753	6808	3699
3745	3400	3559	3565	497	6749	862
1957	1909	1776	1882	3547	6690	3521
3727	3402	3548	3566	859	6775	310
7758	7634	7684	7659	7595	7919	7568
3787	3527	3644	3639	376	6807	764
6979	6827	6886	6876	6937	423	6953
490	1830	1664	1618	3766	6788	3702

7765	7640	7691	7665	7602	7923	7575
434	1836	1878	1726	3807	6847	3745
443	1904	1912	1808	3765	6819	3667
3785	3443	3611	3617	551	6783	912
6922	6794	6858	6845	6891	419	6919
7680	7568	7555	7527	7498	7888	7496
7777	7651	7701	7676	7610	7932	7583
7666	7574	7617	7600	7546	7698	7538
520	1880	1882	1740	3759	6827	3677
1883	1775	739	1627	3637	6784	3572
7835	7703	7739	7693	7685	7947	7669
2243	2105	2079	2025	3771	6808	3687
454	1834	1878	1724	3751	6834	3705
181	1903	1833	1749	3802	6831	3744
6717	6590	6673	6657	6597	383	6598
3805	3435	3607	3599	619	6782	849
7714	7610	7657	7635	7582	7726	7580
156	1896	1823	1742	3795	6824	3723
2328	2132	2056	1882	3727	6831	3667
2003	1816	475	1737	3630	6806	3589
1861	1696	1698	179	3655	6752	3600
497	1873	1877	1731	3744	6827	3694
2014	1787	1839	1890	3590	6754	3532
2033	1816	531	1755	3608	6814	3563
6790	6687	6762	6740	6759	179	6760
7718	7614	7661	7639	7586	7731	7584
7682	7570	7557	7529	7500	7888	7498
6718	6591	6674	6658	6598	384	6599
6785	6664	6743	6717	6744	194	6745
6719	6592	6675	6659	6599	385	6600
3690	3364	3533	3526	511	6733	784
774	2082	2055	1943	3842	6786	3786
417	1811	1855	1703	3790	6820	3720
2278	2055	1835	1962	3649	6815	3585
2423	2082	2094	2032	3783	6780	3714
3870	3542	3702	3721	630	6824	916
3870	3542	3702	3721	630	6824	916
7658	7564	7607	7592	7536	7687	7522
1961	1767	1827	1785	3609	6675	3493
3830	3483	3652	3639	679	6812	923
1949	1888	412	1709	3591	6783	3551
3743	3417	3589	3579	558	6770	787
255	1836	1768	1680	3781	6804	3707
3768	3511	3664	3635	1038	6784	807
203	1963	1894	1822	3818	6819	3730
6789	6686	6761	6741	6751	15	6748
1956	1908	1775	1881	3545	6691	3520
6806	6705	6778	6764	6787	211	6788
1821	1739	1737	340	3632	6742	3564
3732	3441	3572	3567	801	6744	19
1870	1705	1707	188	3664	6761	3609
3712	3408	3519	3516	395	6730	658
7681	7569	7556	7528	7499	7887	7497
7681	7569	7556	7528	7499	7887	7497
7682	7570	7557	7529	7500	7888	7498

7680	7568	7555	7527	7498	7886	7496
6792	6691	6764	6750	6773	195	6774
2335	2032	2046	1870	3736	6835	3691
407	2027	1953	1883	3800	6824	3805
2001	1814	473	1735	3628	6804	3587
7639	7581	7576	7586	7556	7745	7558
37	1967	1893	1837	3776	6801	3725
1914	1763	74	1717	3619	6763	3561
1894	1742	1737	261	3601	6745	3538
6776	6657	6738	6710	6725	259	6726
3770	3440	3609	3598	570	6793	842
7681	7569	7556	7528	7499	7887	7497
7680	7568	7555	7527	7498	7886	7496
7677	7503	7567	7515	7534	7865	7470
7682	7570	7557	7529	7500	7888	7498
7682	7570	7557	7529	7500	7888	7498
2320	2173	2107	1927	3737	6816	3686
400	2020	1946	1876	3793	6817	3798
751	2037	2018	1918	3851	6770	3795
1975	1785	1839	1803	3595	6670	3484
1941	1771	1820	1787	3593	6666	3445
1889	1737	1734	256	3598	6740	3535
1895	1743	1738	262	3602	6746	3539
2329	2126	2046	1868	3736	6831	3691
1991	1750	1806	1858	3599	6749	3548
1983	1742	1800	1850	3591	6741	3540
2024	1869	1971	1911	3545	6678	3446
	1964	1890	1834	3781	6790	3730
1964		1735	1708	3501	6687	3438
1890	1735		1699	3619	6762	3569
1834	1708	1699		3627	6742	3564
3781	3501	3619	3627		6746	798
6790	6687	6762	6742	6746		6743
3730	3438	3569	3564	798	6743	
495	1871	1875	1729	3742	6824	3692
7673	7585	7588	7580	7526	7772	7541
3730	3438	3569	3564	798	6743	
294	1864	1799	1708	3792	6828	3695
3690	3364	3533	3526	513	6737	786
490	1830	1664	1618	3766	6788	3702
268	1833	1769	1676	3770	6807	3707
436	1856	1876	1742	3787	6828	3719
1959	15	1730	1703	3496	6682	3433
6927	6807	6884	6865	6883	341	6899
762	2071	2059	1943	3828	6773	3767
3735	3381	3548	3556	583	6730	856
2180	2088	1995	1956	3757	6808	3669
3665	3497	3327	3535	4213	6898	4156
1810	1728	1726	329	3621	6731	3553
1969	1851	1917	1687	3615	6702	3579
7674	7586	7589	7581	7527	7773	7542
440	1913	1809	1747	3800	6802	3744
475	1851	1853	1715	3786	6833	3720
7647	7556	7589	7579	7527	7726	7523
3800	3466	3631	3631	585	6798	877

3696	3377	3560	3538	558	6742	809
1889	1771	735	1621	3649	6782	3568
24	1954	1880	1824	3760	6785	3706
7672	7584	7587	7579	7525	7771	7540
3743	3403	3583	3562	559	6745	806
3743	3403	3583	3562	559	6745	806
7638	7547	7580	7570	7518	7717	7514
1982	1741	1799	1849	3590	6740	3539
1892	1739	28	1701	3619	6765	3569
3689	3387	3543	3502	1067	6784	642
3791	3518	3632	3632	437	6789	794
1826	1700	1691	14	3619	6734	3556
1964	26	1735	1708	3501	6687	3438
492	1848	1858	1727	3789	6833	3726
2430	2105	1979	2108	3672	6814	3602
7758	7634	7684	7659	7595	7918	7568
615	1933	1940	1777	3837	6824	3783
7672	7584	7587	7579	7525	7771	7540
7671	7583	7586	7578	7524	7770	7539
2327	2124	2044	1866	3734	6829	3689
1877	1795	133	1783	3594	6747	3540
6786	6667	6744	6719	6737	270	6739
2335	2032	2046	1870	3736	6835	3691
2335	2032	2046	1870	3736	6835	3691
7673	7585	7588	7580	7526	7772	7541
285	1857	1792	1699	3785	6821	3688
257	1837	1769	1681	3784	6805	3712
1959	19	1730	1703	3496	6682	3433
3745	3421	3592	3582	560	6772	789
3797	3462	3628	3628	591	6810	877
7639	7581	7576	7586	7556	7745	7558
1887	1735	1732	254	3596	6737	3533
1983	1883	1949	1719	3624	6702	3582
2032	1815	530	1754	3607	6813	3562
1997	1756	1814	1864	3605	6755	3554
1885	1618	1778	1426	3627	6805	3541
1883	1616	1776	1424	3625	6803	3539
1914	1647	1807	1455	3656	6831	3570
6821	6703	6781	6757	6772	195	6768
3789	3502	3624	3613	890	6777	474
1983	1741	1800	1847	3591	6741	3540
2422	2107	2007	2072	3721	6805	3645
7639	7548	7581	7571	7519	7718	7515
7731	7627	7674	7652	7599	7743	7597
3715	3454	3586	3580	493	6782	700
1790	1681	1743	226	3628	6727	3571
7726	7641	7656	7667	7662	7960	7670
2330	2127	2047	1869	3737	6832	3692
2336	2133	2053	1875	3743	6838	3698
1859	1729	1720	59	3650	6757	3587
445	1829	1873	1719	3750	6825	3690
6832	6729	6790	6782	6829	251	6830
1890	1804	142	1794	3612	6767	3543
6763	6673	6735	6711	6732	119	6729
264	1874	1805	1720	3772	6809	3683

261	1871	1802	1717	3769	6806	3680
2406	2113	1955	2092	3699	6800	3635
1821	1739	1737	340	3632	6742	3564
7637	7579	7574	7584	7554	7743	7556
7638	7580	7575	7585	7553	7744	7557
7723	7638	7653	7664	7659	7955	7667
1940	1778	1840	1800	3604	6661	3478

MOD1-EC6811	MOD1-EC6825	KCJK4201	CFSAN046659	MOD1-ECOR25	MOD1-ECOR34
3725	7575	683	3726	830	3759
1827	7624	3578	1833	3523	1769
1998	7618	3602	1937	3539	1831
3891	7776	3415	3925	3346	3935
3652	7526	806	3691	348	3672
25038	25523	24923	25047	24901	25029
1825	7624	3567	1885	3525	1803
1916	7623	3493	1884	3483	1814
1998	7604	3544	1936	3540	1856
25037	25522	24922	25046	24900	25028
307	7687	3697	76	3690	266
2355	7769	3680	2282	3656	2208
7672	2624	7556	7667	7544	7633
1941	7549	3508	1851	3448	1793
1970	7618	3556	1925	3494	1895
6986	7881	6958	6990	6976	6929
1754	7562	3557	1725	3510	1635
2318	7725	3624	2360	3635	2278
2318	7725	3624	2360	3635	2278
1956	7671	3637	1911	3576	1887
3650	7823	4167	3682	4159	3610
2227	7653	3690	2197	3680	2102
7792	2660	7611	7787	7573	7748
1836	7587	3539	1770	3516	1719
50	7715	3700	359	3671	497
313	7683	3685	140	3690	342
7713	2655	7527	7710	7501	7677
1956	7633	3568	1928	3534	1816
7770	2599	7668	7761	7643	7732
7770	2599	7668	7761	7643	7732
1872	7588	3439	1865	3365	1831
31006	31233	30910	31001	30906	31008
7793	3377	7570	7780	7556	7745
7906	3403	7724	7886	7690	7848
3624	7578	625	3659	1131	3653
3624	7578	625	3659	1131	3653
7710	12	7543	7706	7511	7674
7880	3357	7659	7860	7620	7826
484	7671	3707	267	3676	475
16	7711	3694	353	3673	491
2228	7654	3691	2198	3681	2103
3759	7547	1053	3819	599	3780
7719	3592	7472	7695	7460	7660
6830	7776	6749	6834	6743	6794
6810	7760	6754	6812	6763	6772
3638	7545	942	3693	724	3659
505	7676	3814	350	3823	540
6840	7789	6783	6841	6792	6802
2193	7784	3766	2134	3698	2035
7719	3648	7470	7695	7458	7662
6810	7784	6736	6810	6749	6760
7715	3500	7496	7698	7468	7661
2232	7659	3695	2203	3685	2108
2052	7615	3691	2005	3626	1970

1947	7612	3500	1881	3485	1809
1913	7555	3534	1809	3485	1747
1767	7614	3609	1744	3563	1654
1933	7646	3539	1907	3502	1795
7871	3377	7669	7849	7633	7811
7684	42	7523	7680	7491	7648
1968	7597	3549	1898	3525	1812
1939	7617	3479	1871	3471	1799
1983	7592	3489	1915	3503	1843
1938	7625	3418	1870	3445	1798
1968	7599	3549	1898	3525	1812
6797	7769	6753	6797	6764	6767
7764	3428	7514	7751	7495	7710
1944	7657	3548	1918	3511	1806
2224	7654	3689	2196	3679	2099
7714	3497	7495	7697	7467	7660
7715	3498	7496	7698	7468	7661
2332	7761	3670	2279	3656	2203
1827	7624	3578	1833	3523	1769
1953	7657	3527	1921	3492	1809
7703	2645	7525	7700	7515	7665
2227	7655	3698	2207	3688	2112
730	7628	3714	532	3719	703
7876	3385	7676	7854	7640	7816
7767	2599	7665	7758	7640	7729
6841	7667	6779	6851	6744	6811
1918	7615	3485	1844	3469	1772
1990	7614	3589	1916	3552	1806
7796	3379	7573	7783	7559	7748
7718	3501	7499	7701	7471	7664
7804	3367	7577	7791	7563	7754
7803	3366	7576	7790	7562	7753
7807	3370	7580	7794	7566	7757
7793	3376	7569	7780	7555	7745
2327	7726	3616	2373	3624	2269
7673	2625	7557	7668	7545	7634
329	7715	3774	586	3751	692
7754	2579	7578	7749	7562	7711
2208	7661	3696	2195	3688	2087
312	7693	3705	89	3691	279
385	7680	3721	162	3683	370
225	7742	3749	329	3702	503
1985	7611	3580	1911	3550	1801
187	7744	3750	291	3710	465
7807	3370	7580	7794	7566	7757
6748	7787	6601	6748	6610	6700
6808	7762	6760	6810	6769	6770
311	7685	3699	88	3684	292
3717	7532	862	3747	256	3732
1954	7559	3521	1864	3460	1806
3664	7567	310	3702	861	3702
7791	3375	7568	7778	7554	7743
3780	7552	764	3806	495	3798
6981	7876	6953	6985	6971	6924
489	7672	3702	304	3697	

7798	3376	7575	7785	7561	7750
182	7742	3745	286	3705	460
275	7702	3667	364	3696	538
3761	7500	912	3785	353	3798
6940	7868	6919	6944	6935	6883
7715	3500	7496	7698	7468	7661
7810	3373	7583	7797	7569	7760
7708	2649	7538	7705	7522	7670
79	7714	3677	362	3670	498
1798	7608	3572	1763	3562	1616
7871	3377	7669	7849	7633	7811
2185	7714	3687	2180	3688	2091
127	7718	3705	304	3682	476
394	7694	3744	183	3696	379
6745	7784	6598	6745	6607	6697
3755	7561	849	3799	431	3775
7756	2581	7580	7751	7564	7713
387	7682	3723	164	3685	372
2238	7654	3667	2194	3657	2099
1990	7614	3589	1916	3552	1806
1758	7605	3600	1735	3554	1645
16	7711	3694	353	3673	491
1935	7651	3532	1954	3471	1842
1992	7596	3563	1940	3531	1816
6816	7766	6760	6818	6769	6778
7760	2586	7584	7755	7568	7717
7717	3500	7498	7700	7470	7663
6746	7785	6599	6746	6608	6698
6811	7751	6745	6813	6754	6769
6747	7786	6600	6747	6609	6699
3671	7507	784	3696	14	3697
345	7732	3786	604	3767	710
162	7724	3720	261	3703	435
2167	7683	3585	2173	3592	2107
2290	7707	3714	2311	3726	2214
3838	7609	916	3891	578	3868
3838	7609	916	3891	578	3868
7700	2642	7522	7697	7512	7662
1935	7620	3493	1861	3484	1789
3797	7570	923	3835	486	3822
1926	7568	3551	1878	3531	1748
3730	7548	787	3743	144	3754
316	7692	3707	105	3693	287
3743	7590	807	3828	1280	3793
484	7676	3730	246	3674	474
6823	7773	6748	6827	6742	6787
1953	7562	3520	1863	3458	1805
6832	7784	6788	6834	6797	6794
1740	7603	3564	1719	3527	1627
3694	7544	19	3697	789	3704
1767	7614	3609	1744	3563	1654
3649	7514	658	3709	432	3677
7716	3499	7497	7699	7469	7662
7716	3499	7497	7699	7469	7662
7717	3500	7498	7700	7470	7663

7715	3498	7496	7698	7468	7661
6818	7767	6774	6820	6783	6780
2234	7636	3691	2204	3619	2109
496	7667	3805	341	3814	531
1988	7612	3587	1914	3550	1804
7674	2624	7558	7669	7546	7635
490	7678	3725	289	3685	493
1887	7594	3561	1825	3533	1688
1811	7624	3538	1768	3498	1674
6798	7778	6726	6806	6736	6754
3758	7548	842	3763	193	3774
7716	3500	7497	7699	7469	7662
7715	3498	7496	7698	7468	7661
7639	3461	7470	7616	7444	7579
7717	3501	7498	7700	7470	7663
7717	3501	7498	7700	7470	7663
2219	7667	3686	2207	3674	2110
489	7660	3798	334	3807	524
356	7717	3795	581	3776	687
1945	7618	3484	1864	3480	1801
1913	7603	3445	1831	3455	1783
1806	7621	3535	1763	3495	1669
1812	7625	3539	1769	3499	1675
2228	7654	3691	2198	3681	2103
1942	7655	3548	1916	3511	1804
1934	7647	3540	1908	3503	1796
2061	7563	3446	1992	3486	1927
495	7673	3730	294	3690	490
1871	7585	3438	1864	3364	1830
1875	7588	3569	1799	3533	1664
1729	7580	3564	1708	3526	1618
3742	7526	798	3792	513	3766
6824	7772	6743	6828	6737	6788
3692	7541		3695	786	3702
	7708	3692	351	3671	489
7708		7541	7704	7509	7672
3692	7541		3695	786	3702
351	7704	3695		3696	304
3671	7509	786	3696		3697
489	7672	3702	304	3697	
313	7682	3707	91	3662	283
236	7725	3719	324	3703	490
1866	7582	3433	1859	3359	1825
6952	7891	6899	6958	6907	6918
353	7722	3767	606	3753	712
3707	7556	856	3746	401	3725
2118	7715	3669	2091	3692	1998
3639	7810	4156	3671	4148	3599
1729	7592	3553	1708	3516	1616
1817	7621	3579	1835	3526	1763
7709	7	7542	7705	7510	7673
449	7675	3744	298	3726	450
88	7725	3720	317	3697	457
7689	2563	7523	7684	7497	7649
3768	7559	877	3821	430	3798

3702	7554	809	3721	197	3701
1810	7606	3568	1753	3563	1606
477	7662	3706	272	3666	480
7707	3	7540	7703	7508	7671
3736	7513	806	3742	192	3741
3736	7513	806	3742	192	3741
7680	2554	7514	7675	7488	7640
1933	7646	3539	1907	3502	1795
1879	7590	3569	1803	3533	1668
3634	7590	642	3665	1144	3659
3771	7510	794	3786	461	3801
1721	7572	3556	1700	3518	1610
1871	7587	3438	1864	3364	1830
97	7713	3726	343	3669	481
2293	7725	3602	2322	3612	2255
7791	3374	7568	7778	7554	7743
234	7688	3783	453	3759	570
7707	5	7540	7703	7508	7671
7706	4	7539	7702	7507	7670
2226	7652	3689	2196	3679	2101
1934	7577	3540	1839	3500	1725
6810	7782	6739	6817	6725	6767
2234	7636	3691	2204	3619	2109
2234	7636	3691	2204	3619	2109
7708	6	7541	7704	7509	7672
342	7697	3688	37	3689	295
316	7693	3712	105	3696	287
1866	7582	3433	1859	3359	1825
3732	7554	789	3745	146	3756
3765	7565	877	3818	430	3795
7674	2626	7558	7669	7546	7635
1804	7619	3533	1761	3493	1667
1833	7626	3582	1853	3524	1791
1991	7595	3562	1939	3530	1815
1948	7659	3554	1922	3517	1810
1838	7589	3541	1772	3518	1721
1836	7587	3539	1770	3516	1719
1867	7616	3570	1801	3547	1750
6849	7793	6768	6853	6778	6809
3742	7546	474	3773	964	3773
1934	7647	3540	1908	3503	1796
2279	7693	3645	2307	3654	2206
7681	2555	7515	7676	7489	7641
7773	2598	7597	7768	7581	7730
3699	7575	700	3708	552	3713
1697	7590	3571	1674	3527	1586
7772	2601	7670	7763	7645	7734
2229	7655	3692	2199	3682	2104
2235	7661	3698	2205	3688	2110
1754	7587	3587	1733	3549	1643
116	7709	3690	301	3671	471
6858	7786	6830	6860	6839	6810
1949	7598	3543	1839	3502	1740
6792	7742	6729	6796	6723	6764
311	7681	3683	138	3688	340

308	7678	3680	135	3685	337
2285	7707	3635	2299	3640	2229
1740	7603	3564	1719	3527	1627
7672	2624	7556	7667	7544	7633
7673	2625	7557	7668	7543	7634
7769	2598	7667	7760	7642	7731
1938	7616	3478	1870	3470	1798

MOD1-EC6368	MOD1-EC6936	MOD1-EC6953	MOD1-EC6831	MOD1-EC6779	MOD1-EC6577
3748	3764	3401	6924	3799	884
1816	1862	1860	6825	1974	3546
1879	1943	1833	6942	2170	3581
3920	3911	3660	7136	4031	3407
3670	3683	3327	6877	3736	172
25028	25047	25010	25056	25037	24912
1854	1834	1442	6842	2021	3533
1845	1925	1789	6830	2141	3495
1928	1963	1844	6911	2156	3577
25027	25046	25009	25055	25036	24911
53	282	1825	6938	564	3724
2268	2330	2245	7000	2507	3704
7632	7671	7576	7869	7686	7563
1829	1966	1891	6810	2106	3498
1904	1959	1815	6929	2159	3526
6969	6997	6827	511	6941	6945
1693	1721	1740	6866	1960	3542
2351	2345	2097	6923	2480	3647
2351	2345	2097	6923	2480	3647
1878	1937	1686	6960	2143	3610
3656	3647	3503	7021	3730	4202
2195	2216	2120	6950	2379	3705
7747	7789	7671	7908	7821	7606
1741	1879	1611	6924	2020	3541
319	237	1874	6963	361	3715
114	325	1871	6941	614	3721
7672	7708	7563	7811	7732	7540
1899	1950	1802	6873	2110	3553
7740	7780	7636	8073	7803	7668
7740	7780	7636	8073	7803	7668
1834	1857	20	6808	2072	3382
31004	31008	30968	30813	31030	30912
7735	7782	7635	8044	7821	7587
7841	7895	7739	8114	7944	7731
3645	3673	3388	6917	3718	1149
3645	3673	3388	6917	3718	1149
7684	7727	7584	7893	7724	7558
7815	7861	7699	8052	7897	7673
252	435	1952	6929	739	3731
315	238	1868	6955	355	3709
2196	2217	2121	6951	2380	3706
3760	3766	3431	6916	3889	530
7653	7681	7548	7893	7735	7517
6813	6834	6688	347	6779	6736
6791	6815	6676	363	6760	6742
3676	3682	3426	6861	3727	779
314	498	2031	6951	794	3830
6821	6845	6706	393	6790	6771
2106	2226	2234	7084	2356	3731
7653	7681	7544	7883	7735	7515
6789	6817	6656	453	6758	6728
7653	7692	7567	8006	7741	7512
2201	2222	2126	6956	2384	3710
1977	2113	2144	6923	2157	3668

1856	1934	1788	6813	2156	3526
1797	1954	2003	6846	2072	3538
1712	1778	1700	6885	1972	3593
1878	1923	1736	6875	2091	3527
7804	7853	7700	8072	7907	7674
7658	7701	7560	7917	7698	7538
1899	1967	1813	6909	2147	3560
1857	1920	1774	6820	2142	3485
1901	1962	1818	6796	2190	3536
1856	1927	1801	6808	2143	3479
1899	1967	1813	6911	2147	3560
6776	6818	6679	441	6745	6740
7706	7753	7600	8052	7790	7528
1889	1934	1747	6884	2102	3536
2192	2213	2117	6953	2376	3702
7652	7691	7566	8005	7740	7511
7653	7692	7567	8006	7741	7512
2265	2311	2278	7011	2486	3702
1816	1862	1860	6825	1974	3546
1892	1929	1756	6878	2107	3521
7662	7700	7564	7828	7730	7546
2205	2226	2136	6962	2385	3715
470	639	2119	6865	978	3811
7809	7858	7705	8081	7912	7681
7737	7777	7633	8068	7800	7665
6805	6830	6708	623	6792	6767
1830	1925	1750	6814	2120	3505
1884	1951	1811	6937	2149	3571
7738	7785	7638	8047	7824	7590
7656	7695	7570	8009	7744	7515
7746	7793	7644	8050	7832	7594
7745	7792	7643	8049	7831	7593
7749	7796	7647	8053	7835	7597
7735	7782	7635	8044	7821	7586
2353	2346	2138	6936	2499	3636
7633	7672	7577	7870	7687	7564
557	494	2061	6897	46	3797
7711	7751	7605	7863	7779	7583
2176	2221	2109	6960	2360	3716
53	285	1826	6943	573	3724
140	356	1889	6952	666	3729
273	125	1813	6981	489	3751
1890	1948	1816	6930	2146	3570
235	99	1836	6981	465	3757
7749	7796	7647	8053	7835	7597
6727	6751	6588	576	6698	6589
6789	6813	6680	371	6758	6748
66	296	1841	6936	588	3702
3733	3773	3395	6908	3792	441
1842	1979	1904	6821	2119	3510
3703	3697	3397	6916	3745	931
7733	7780	7633	8043	7819	7585
3802	3817	3522	6839	3862	582
6964	6992	6822	506	6936	6940
283	490	1825	6918	712	3725

7740	7787	7639	8047	7826	7592
230	94	1831	6975	460	3752
328	212	1899	6930	548	3758
3788	3801	3438	6928	3850	461
6923	6933	6789	574	6891	6898
7653	7692	7567	8008	7741	7512
7752	7799	7650	8056	7838	7600
7667	7705	7571	7836	7735	7553
331	252	1875	6955	358	3722
1730	1817	1770	6912	2012	3577
7804	7853	7700	8072	7907	7674
2150	2178	2100	6926	2335	3694
262	180	1829	6962	434	3720
149	365	1898	6961	675	3741
6724	6748	6585	573	6695	6586
3753	3747	3430	6885	3838	349
7713	7753	7607	7865	7781	7585
142	358	1891	6954	668	3731
2191	2219	2127	6950	2390	3674
1884	1951	1811	6937	2149	3571
1703	1769	1691	6876	1963	3584
315	238	1868	6955	355	3709
1895	1937	1782	6889	2106	3547
1917	1955	1811	6945	2147	3549
6797	6821	6682	369	6766	6748
7717	7757	7611	7870	7785	7589
7655	7694	7569	8008	7743	7514
6725	6749	6586	574	6696	6587
6792	6816	6659	384	6761	6733
6726	6750	6587	575	6697	6588
3662	3703	3359	6903	3753	399
573	508	2077	6914	68	3815
226	153	1806	6948	435	3735
2156	2194	2050	6932	2341	3606
2299	2315	2077	6897	2458	3744
3866	3887	3537	6857	3930	444
3866	3887	3537	6857	3930	444
7659	7697	7561	7825	7727	7543
1838	1920	1762	6825	2134	3503
3790	3806	3478	6943	3878	369
1844	1917	1883	6916	2101	3538
3724	3762	3412	6940	3810	413
69	286	1831	6934	595	3722
3811	3814	3506	6924	3841	1237
226	396	1958	6947	748	3736
6806	6827	6681	352	6772	6735
1841	1978	1901	6822	2118	3508
6813	6837	6700	401	6782	6776
1687	1755	1734	6863	1946	3571
3709	3721	3436	6900	3769	859
1712	1778	1700	6885	1972	3593
3675	3706	3403	6879	3727	589
7654	7693	7568	8007	7742	7513
7654	7693	7568	8007	7742	7513
7655	7694	7569	8008	7743	7514

7653	7692	7567	8006	7741	7512
6799	6823	6686	385	6768	6762
2202	2223	2027	6955	2386	3650
305	489	2022	6942	785	3821
1882	1949	1809	6935	2147	3569
7634	7673	7578	7871	7688	7565
263	439	1962	6938	757	3730
1795	1888	1758	6884	2071	3548
1736	1822	1737	6868	2009	3528
6780	6803	6652	449	6752	6718
3749	3768	3435	6916	3845	458
7654	7693	7568	8007	7742	7513
7653	7692	7567	8006	7741	7512
7572	7624	7502	8003	7667	7489
7655	7694	7569	8008	7743	7514
7655	7694	7569	8008	7743	7514
2193	2210	2168	6936	2385	3699
298	482	2015	6935	778	3814
550	485	2032	6898	97	3810
1855	1934	1780	6820	2144	3499
1831	1910	1766	6816	2118	3484
1731	1817	1732	6863	2004	3525
1737	1823	1738	6869	2010	3529
2196	2217	2121	6951	2380	3706
1887	1932	1745	6884	2100	3536
1879	1924	1737	6876	2092	3528
1983	2034	1864	6805	2254	3526
268	436	1959	6927	762	3735
1833	1856	15	6807	2071	3381
1769	1876	1730	6884	2059	3548
1676	1742	1703	6865	1943	3556
3770	3787	3496	6883	3828	583
6807	6828	6682	341	6773	6730
3707	3719	3433	6899	3767	856
313	236	1866	6952	353	3707
7682	7725	7582	7891	7722	7556
3707	3719	3433	6899	3767	856
91	324	1859	6958	606	3746
3662	3703	3359	6907	3753	401
283	490	1825	6918	712	3725
	270	1828	6937	577	3723
270		1851	6965	494	3744
1828	1851		6802	2066	3376
6937	6965	6802		6904	6854
577	494	2066	6904		3799
3723	3744	3376	6854	3799	
2061	2097	2083	6928	2258	3716
3645	3636	3492	7011	3719	4191
1676	1744	1723	6852	1935	3560
1808	1838	1846	6821	1966	3553
7683	7726	7583	7892	7723	7557
263	484	1908	6923	699	3759
288	209	1846	6961	363	3733
7646	7686	7553	7864	7716	7534
3796	3817	3461	6833	3860	293

3712	3748	3372	6912	3765	418
1731	1821	1766	6910	2006	3583
250	426	1949	6922	744	3716
7681	7724	7581	7890	7721	7555
3750	3774	3398	6914	3807	424
3750	3774	3398	6914	3807	424
7637	7677	7544	7855	7707	7525
1878	1923	1736	6875	2091	3527
1773	1880	1734	6886	2063	3548
3644	3685	3382	6923	3724	1153
3767	3789	3513	6932	3853	603
1668	1734	1695	6857	1935	3548
1833	1856	21	6807	2071	3381
276	208	1843	6961	383	3734
2319	2314	2100	6931	2465	3625
7733	7780	7633	8042	7819	7585
417	353	1928	6952	503	3790
7681	7724	7581	7890	7721	7555
7680	7723	7580	7889	7720	7554
2194	2215	2119	6949	2378	3704
1821	1929	1790	6877	2099	3508
6769	6801	6662	460	6763	6724
2202	2223	2027	6955	2386	3650
2202	2223	2027	6955	2386	3650
7682	7725	7582	7891	7722	7556
82	315	1852	6951	597	3739
69	286	1832	6935	595	3727
1828	1851	14	6802	2066	3376
3726	3764	3416	6942	3812	415
3793	3814	3457	6845	3857	283
7634	7673	7578	7871	7688	7565
1729	1815	1730	6858	2002	3523
1829	1866	1878	6821	1992	3550
1916	1954	1810	6944	2146	3548
1893	1938	1751	6890	2106	3542
1743	1881	1613	6926	2022	3543
1741	1879	1611	6924	2020	3541
1772	1910	1642	6952	2051	3572
6832	6852	6698	326	6797	6701
3728	3758	3497	6901	3843	984
1879	1924	1736	6876	2092	3528
2298	2308	2102	6922	2441	3672
7638	7678	7545	7856	7708	7526
7730	7770	7624	7882	7798	7602
3713	3746	3449	6922	3771	554
1642	1702	1676	6848	1903	3559
7742	7782	7638	8075	7805	7670
2197	2218	2122	6952	2381	3707
2203	2224	2128	6958	2387	3713
1701	1767	1724	6880	1968	3579
259	173	1824	6953	431	3709
6839	6863	6724	441	6808	6818
1837	1944	1799	6897	2118	3520
6775	6799	6668	454	6741	6716
112	323	1869	6939	612	3719

109	320	1866	6936	609	3716
2284	2312	2108	6917	2459	3654
1687	1755	1734	6863	1946	3571
7632	7671	7576	7869	7686	7563
7633	7672	7577	7870	7687	7562
7739	7779	7635	8070	7802	7667
1856	1919	1773	6819	2141	3484

MOD1-EC5070	MOD1-EC6716	MOD1-EC6835	MOD1-EC6847	MOD1-EC6868	MOD1-EC6870
3744	4181	3593	3588	7576	3793
1889	3626	1734	74	7625	1884
2055	3287	1820	2071	7619	1911
3944	4438	3774	3852	7777	3923
3675	4158	3509	3514	7527	3706
24987	25018	25015	24983	25524	25027
1993	3418	1504	1787	7625	1916
2236	3501	1863	1956	7624	1939
2138	3551	1942	1924	7605	2011
24986	25017	25014	24982	25523	25026
2051	3635	1672	1797	7688	256
1668	3802	2134	2016	7770	2340
7688	7759	7600	7585	2625	7643
2008	3451	1918	1862	7550	1870
1902	3630	1836	1333	7619	1980
6955	7053	6870	6839	7882	6947
1949	3553	372	1726	7563	1776
1585	3673	2155	2162	7726	2415
1585	3673	2155	2162	7726	2415
2151	3530	1777	2071	7672	1968
3696	25	3608	3620	7824	3702
1591	3795	1898	1886	7654	2251
7792	7852	7690	7685	2661	7771
1864	3551	1503	1889	7588	1808
2124	3657	1743	1835	7716	463
2105	3667	1721	1861	7684	280
7699	7761	7603	7616	2656	7679
2063	3561	1909	2034	7634	1971
7776	7883	7679	7698	2600	7746
7776	7883	7679	7698	2600	7746
2089	3498	1729	1852	7589	1914
30955	30966	30922	30961	31234	31023
7764	7859	7676	7682	3378	7759
7844	7927	7754	7777	3404	7869
3639	4175	3520	3565	7579	3694
3639	4175	3520	3565	7579	3694
7717	7812	7594	7623	13	7677
7826	7919	7732	7769	3358	7842
2169	3655	1801	1956	7672	419
2120	3641	1731	1819	7712	451
1592	3796	1899	1887	7655	2252
3806	4258	3622	3619	7548	3819
7667	7765	7586	7594	3593	7684
6814	6904	6737	6708	7777	6808
6808	6910	6725	6678	7761	6782
3722	4154	3553	3550	7546	3698
2233	3745	1874	2001	7677	364
6838	6940	6755	6708	7790	6812
2233	3744	2203	2170	7785	2174
7667	7773	7584	7596	3649	7684
6786	6948	6703	6652	7785	6757
7646	7779	7548	7576	3501	7685
1597	3801	1904	1892	7660	2257
2208	3678	2068	2065	7616	2013

2236	3492	1870	1966	7613	1962
2023	3518	1923	1902	7556	1856
2012	3532	471	1767	7615	1765
2028	3537	1858	1955	7647	1950
7804	7883	7712	7745	3378	7832
7691	7840	7570	7597	43	7651
2028	3342	1838	1973	7598	1916
2233	3478	1856	1944	7618	1940
2241	3467	1900	1986	7593	1972
2190	3468	1841	1975	7626	1941
2028	3341	1838	1973	7600	1916
6813	6925	6736	6689	7770	6763
7727	7836	7639	7651	3429	7730
2037	3548	1869	1966	7658	1961
1588	3792	1895	1883	7655	2248
7645	7778	7547	7575	3498	7684
7646	7779	7548	7576	3499	7685
1721	3806	2139	2018	7762	2333
1889	3626	1734	74	7625	1884
2028	3535	1878	1979	7658	1976
7688	7756	7608	7619	2646	7671
1607	3803	1888	1874	7656	2245
2325	3736	1927	2091	7629	653
7809	7888	7717	7750	3386	7837
7773	7880	7676	7696	2600	7743
6837	6949	6744	6722	7668	6853
2225	3458	1842	1926	7616	1921
2030	3280	1797	2047	7615	1904
7767	7862	7679	7685	3380	7762
7649	7782	7551	7579	3502	7688
7773	7865	7685	7691	3368	7770
7772	7864	7684	7690	3367	7769
7776	7867	7688	7694	3371	7773
7764	7859	7676	7682	3377	7759
1638	3644	2150	2185	7727	2392
7689	7760	7601	7586	2626	7644
2244	3718	1927	1951	7716	681
7728	7812	7646	7670	2580	7718
1596	3788	1901	1889	7662	2232
2055	3644	1673	1805	7694	265
2127	3683	1740	1879	7681	346
2130	3616	1718	1826	7743	479
2031	3275	1802	2044	7612	1901
2130	3641	1741	1839	7745	443
7776	7868	7688	7694	3371	7773
6736	6888	6651	6610	7788	6716
6806	6906	6727	6676	7763	6778
2075	3653	1690	1817	7686	276
3747	4175	3559	3558	7533	3771
2021	3464	1931	1875	7560	1883
3668	4144	3566	3604	7568	3740
7762	7857	7674	7680	3376	7757
3770	4223	3635	3639	7553	3834
6950	7048	6865	6834	7877	6942
1998	3599	1616	1763	7673	450

7768	7863	7680	7686	3377	7764
2125	3636	1736	1834	7743	438
2125	3625	1794	1880	7703	490
3784	4230	3612	3587	7501	3812
6915	7007	6834	6801	7869	6916
7646	7779	7548	7576	3501	7685
7779	7870	7691	7697	3374	7776
7695	7766	7613	7624	2650	7676
2115	3648	1740	1830	7715	482
1989	3373	1714	1835	7609	1788
7804	7883	7712	7745	3378	7832
303	3662	2052	1941	7715	2197
2125	3640	1734	1828	7719	406
2136	3692	1749	1888	7695	355
6733	6885	6648	6607	7785	6713
3791	4216	3591	3601	7562	3803
7730	7814	7648	7672	2582	7720
2129	3685	1742	1881	7683	348
1522	3778	1917	1881	7655	2264
2030	3280	1797	2047	7615	1904
2003	3523	462	1758	7606	1756
2120	3641	1731	1819	7712	451
2077	3574	1899	2001	7652	1957
2058	3251	1811	2035	7597	1932
6814	6916	6731	6684	7767	6788
7734	7818	7652	7676	2587	7724
7648	7781	7550	7578	3501	7687
6734	6886	6649	6608	7786	6714
6789	6909	6708	6661	7752	6770
6735	6887	6650	6609	7787	6715
3692	4150	3516	3528	7508	3726
2260	3734	1943	1969	7733	699
2102	3615	1713	1807	7725	403
1550	3676	1962	2048	7684	2202
1692	3700	2040	2145	7708	2342
3860	4279	3711	3709	7610	3894
3860	4279	3711	3709	7610	3894
7685	7753	7605	7616	2643	7668
2231	3486	1844	1936	7621	1938
3817	4256	3663	3657	7571	3812
2048	3323	1752	2047	7569	1866
3747	4189	3569	3583	7549	3781
2068	3639	1680	1813	7693	271
3740	4185	3639	3700	7591	3824
2191	3721	1812	1956	7677	428
6807	6895	6730	6701	7774	6799
2020	3463	1930	1874	7563	1882
6832	6934	6755	6708	7785	6802
1978	3607	27	1729	7604	1754
3673	4158	3557	3582	7545	3746
2012	3532	471	1767	7615	1765
3671	4129	3514	3516	7515	3709
7647	7780	7549	7577	3500	7686
7647	7780	7549	7577	3500	7686
7648	7779	7550	7578	3501	7687

7646	7779	7548	7576	3499	7685
6818	6920	6741	6694	7768	6788
1634	3779	1901	1885	7637	2258
2224	3736	1865	1992	7668	355
2028	3278	1795	2045	7613	1902
7690	7761	7602	7586	2625	7645
2183	3669	1813	1972	7679	435
2023	3360	1746	1965	7595	1829
2012	3523	540	1749	7625	1807
6782	6948	6701	6646	7779	6763
3762	4215	3592	3588	7549	3801
7647	7780	7549	7577	3501	7686
7646	7779	7548	7576	3499	7685
7635	7786	7539	7545	3462	7624
7648	7781	7550	7578	3502	7687
7648	7781	7550	7578	3502	7687
1551	3780	1958	1902	7668	2227
2217	3729	1858	1985	7661	348
2215	3685	1916	1944	7718	680
2227	3496	1854	1948	7619	1946
2229	3478	1846	1930	7604	1916
2007	3518	535	1744	7622	1802
2013	3524	541	1750	7626	1808
1592	3796	1899	1887	7655	2252
2037	3546	1867	1964	7656	1959
2029	3538	1859	1956	7648	1951
2291	3549	1957	2038	7564	2062
2180	3665	1810	1969	7674	440
2088	3497	1728	1851	7586	1913
1995	3327	1726	1917	7589	1809
1956	3535	329	1687	7581	1747
3757	4213	3621	3615	7527	3800
6808	6898	6731	6702	7773	6802
3669	4156	3553	3579	7542	3744
2118	3639	1729	1817	7709	449
7715	7810	7592	7621	7	7675
3669	4156	3553	3579	7542	3744
2091	3671	1708	1835	7705	298
3692	4148	3516	3526	7510	3726
1998	3599	1616	1763	7673	450
2061	3645	1676	1808	7683	263
2097	3636	1744	1838	7726	484
2083	3492	1723	1846	7583	1908
6928	7011	6852	6821	7892	6923
2258	3719	1935	1966	7723	699
3716	4191	3560	3553	7557	3759
	3685	1967	1871	7716	2118
3685		3597	3609	7811	3691
1967	3597		1718	7593	1743
1871	3609	1718		7622	1874
7716	7811	7593	7622		7676
2118	3691	1743	1874	7676	
2096	3623	1715	1807	7726	473
7672	7753	7592	7604	2564	7655
3803	4214	3621	3638	7560	3824

3706	4156	3528	3533	7555	3752
1981	3367	1708	1827	7607	1784
2170	3656	1800	1959	7663	422
7714	7809	7591	7620	6	7674
3730	4178	3552	3561	7514	3788
3730	4178	3552	3561	7514	3788
7663	7744	7583	7595	2555	7646
2028	3537	1858	1955	7647	1950
1999	3329	1728	1919	7591	1811
3641	4179	3514	3565	7591	3702
3762	4217	3627	3624	7511	3807
1948	3527	321	1679	7573	1739
2088	3497	1728	1851	7588	1913
2094	3619	1727	1822	7714	467
1566	3643	2122	2124	7726	2364
7762	7857	7674	7680	3375	7757
2189	3659	1777	1883	7689	549
7714	7809	7591	7620	6	7674
7713	7808	7590	7619	5	7673
1590	3794	1897	1885	7653	2250
2047	3350	1798	1997	7578	1860
6795	6955	6710	6658	7783	6774
1634	3779	1901	1885	7637	2258
1634	3779	1901	1885	7637	2258
7715	7810	7592	7621	7	7675
2082	3666	1699	1826	7698	289
2068	3642	1681	1814	7694	271
2083	3492	1723	1846	7583	1908
3750	4193	3572	3582	7555	3783
3800	4211	3618	3635	7566	3821
7690	7761	7602	7587	2627	7645
2005	3516	533	1742	7620	1800
1907	3638	1750	98	7627	1882
2057	3250	1810	2034	7596	1931
2043	3552	1873	1970	7660	1965
1866	3553	1505	1891	7590	1810
1864	3551	1503	1889	7588	1808
1895	3582	1534	1920	7617	1839
6797	6930	6748	6701	7794	6800
3739	4203	3625	3636	7547	3794
2029	3538	1856	1956	7648	1951
1467	3677	2069	2104	7694	2354
7664	7745	7584	7596	2556	7647
7747	7831	7665	7689	2599	7737
3687	4153	3572	3577	7576	3740
2015	3578	307	1700	7591	1727
7778	7884	7681	7700	2602	7748
1593	3797	1900	1888	7656	2253
1599	3803	1906	1894	7662	2259
1973	3556	366	1706	7588	1770
2120	3633	1729	1823	7710	395
6858	6938	6773	6724	7787	6830
2060	3365	1809	2010	7599	1885
6779	6897	6702	6675	7743	6798
2103	3666	1720	1859	7682	278

2100	3663	1717	1856	7679	275
1588	3677	2106	2169	7708	2330
1978	3607	27	1729	7604	1754
7688	7759	7600	7585	2625	7643
7689	7760	7601	7586	2626	7644
7775	7882	7678	7697	2599	7745
2232	3477	1853	1943	7617	1939

MOD1-EC6885	MOD1-EC6891	MOD1-EC6897	MOD1-EC6802	MOD1-EC6938	MOD1-EC6943
3759	7554	902	836	3590	3760
1815	7608	3637	3540	1815	1967
1983	7598	3660	3592	660	2003
3914	7713	3417	3420	3877	3871
3688	7516	261	361	3536	3697
25058	25523	24917	24904	25029	25017
1805	7610	3616	3539	1669	1969
1914	7614	3576	3513	1859	1939
1974	7606	3652	3558	1873	2041
25057	25522	24916	24903	25028	25016
273	7663	3797	3704	1715	250
2345	7739	3765	3685	2127	2396
7683	2312	7578	7595	7590	7626
1921	7519	3563	3448	1749	1934
1966	7609	3631	3529	1810	2014
6995	7843	6985	6981	6914	6979
1740	7561	3615	3522	1646	1819
2308	7690	3724	3663	2019	2464
2308	7690	3724	3663	2019	2464
1930	7660	3689	3588	1848	1988
3634	7766	4225	4167	3378	3667
2203	7611	3778	3707	2072	2317
7801	2544	7623	7632	7715	7744
1814	7583	3616	3535	1619	1875
87	7696	3774	3714	1820	485
339	7662	3786	3711	1775	248
7724	510	7555	7547	7633	7664
1930	7613	3626	3552	1921	1993
7783	2365	7677	7680	7681	7713
7783	2365	7677	7680	7681	7713
1852	7559	3467	3378	1772	1955
31016	31206	30927	30890	31001	30995
7801	3241	7605	7609	7703	7751
7914	3378	7745	7739	7812	7863
3646	7570	1172	1148	3544	3666
3646	7570	1172	1148	3544	3666
7727	2565	7561	7556	7608	7664
7888	3334	7689	7683	7787	7829
462	7650	3788	3699	1878	23
90	7692	3770	3704	1812	479
2204	7612	3779	3708	2073	2318
3790	7519	516	616	3615	3730
7727	3416	7455	7511	7623	7682
6839	7732	6804	6748	6788	6791
6817	7727	6790	6768	6771	6779
3678	7528	793	739	3564	3668
529	7628	3859	3849	1925	398
6847	7756	6819	6797	6801	6809
2173	7763	3789	3708	2010	2214
7727	3457	7453	7509	7637	7682
6819	7754	6772	6748	6751	6781
7723	3372	7532	7521	7567	7671
2209	7617	3783	3712	2078	2323
2058	7598	3717	3632	1992	2082

1935	7607	3581	3502	1866	1967
1893	7521	3591	3495	1823	1902
1753	7606	3667	3575	1679	1860
1907	7620	3598	3520	1870	1972
7879	3352	7686	7682	7778	7826
7701	2594	7541	7536	7582	7638
1946	7591	3637	3581	603	2013
1917	7615	3576	3497	1844	1933
1961	7566	3586	3529	1900	1941
1916	7618	3566	3473	1859	1962
1946	7593	3637	3581	603	2013
6806	7738	6791	6781	6776	6766
7772	3309	7552	7548	7667	7734
1918	7631	3607	3529	1881	1983
2200	7612	3781	3704	2069	2314
7722	3369	7531	7520	7566	7670
7723	3370	7532	7521	7567	7671
2326	7731	3765	3685	2136	2393
1815	7608	3637	3540	1815	1967
1927	7633	3600	3510	1898	1960
7714	295	7561	7561	7636	7652
2213	7613	3786	3715	2088	2327
705	7550	3857	3773	1998	582
7884	3360	7695	7689	7783	7831
7780	2364	7674	7677	7678	7710
6844	7623	6831	6780	6803	6807
1896	7611	3583	3495	1837	1964
1968	7602	3650	3571	641	1993
7804	3243	7608	7612	7706	7754
7726	3373	7535	7524	7570	7674
7812	3241	7612	7616	7711	7762
7811	3240	7611	7615	7710	7761
7815	3244	7615	7619	7714	7765
7801	3240	7604	7608	7703	7751
2311	7695	3717	3637	2054	2456
7684	2313	7579	7596	7591	7627
339	7711	3858	3763	1986	738
7765	650	7598	7616	7679	7703
2184	7619	3789	3700	2071	2307
280	7666	3797	3712	1727	251
365	7656	3806	3721	1797	130
198	7701	3826	3755	1841	441
1961	7602	3649	3568	628	1994
160	7705	3832	3763	1816	421
7815	3244	7615	7619	7714	7765
6755	7780	6637	6615	6689	6715
6815	7729	6796	6774	6769	6777
299	7659	3781	3720	1739	246
3744	7531	462	314	3592	3726
1934	7528	3575	3460	1762	1947
3692	7564	947	889	3571	3703
7799	3239	7603	7607	7701	7749
3818	7556	419	496	3669	3781
6990	7838	6980	6976	6909	6974
457	7649	3798	3701	1606	480

7806	3240	7610	7614	7708	7756
155	7703	3827	3758	1811	416
247	7670	3794	3724	1869	425
3800	7494	470	383	3640	3761
6949	7822	6938	6940	6880	6917
7723	3370	7532	7521	7567	7671
7818	3247	7618	7622	7717	7768
7719	283	7568	7568	7641	7657
95	7694	3781	3706	1809	502
1792	7613	3646	3583	28	1873
7879	3352	7686	7682	7778	7826
2163	7671	3785	3710	2077	2233
183	7696	3777	3709	1817	436
374	7670	3818	3739	1806	157
6752	7777	6634	6612	6686	6712
3785	7535	315	467	3617	3786
7765	652	7600	7618	7681	7705
367	7658	3808	3723	1799	132
2214	7608	3757	3684	2111	2317
1968	7604	3650	3571	641	1993
1744	7597	3659	3566	1670	1851
90	7692	3770	3704	1812	479
1940	7618	3588	3527	1909	2004
1968	7582	3630	3543	699	2023
6823	7733	6796	6774	6777	6785
7771	657	7604	7622	7685	7709
7725	3372	7534	7523	7569	7673
6753	7778	6635	6613	6687	6713
6818	7718	6781	6759	6758	6780
6754	7779	6636	6614	6688	6714
3697	7495	428	195	3563	3666
355	7726	3872	3785	2004	756
134	7698	3806	3731	1786	399
2165	7641	3681	3621	1896	2267
2286	7663	3815	3755	2191	2412
3880	7596	195	593	3727	3851
3880	7596	195	593	3727	3851
7711	292	7558	7558	7633	7649
1915	7613	3592	3507	1842	1953
3830	7550	412	523	3684	3811
1916	7563	3609	3542	717	1939
3762	7540	440	200	3618	3719
290	7664	3797	3718	1729	245
3783	7573	1263	1292	3694	3749
461	7641	3819	3762	1865	183
6832	7727	6803	6747	6781	6784
1933	7531	3573	3458	1761	1946
6839	7751	6824	6802	6793	6801
1726	7600	3632	3539	1719	1811
3722	7526	880	812	3571	3708
1753	7606	3668	3575	1679	1860
3693	7505	614	451	3548	3698
7724	3371	7533	7522	7568	7672
7724	3371	7533	7522	7568	7672
7725	3372	7534	7523	7569	7673

7723	3370	7532	7521	7567	7671
6825	7734	6810	6788	6779	6787
2210	7605	3723	3646	2073	2324
520	7619	3850	3840	1916	389
1966	7602	3648	3569	639	1991
7685	2310	7580	7597	7592	7628
470	7654	3795	3717	1892	19
1865	7599	3631	3560	707	1904
1797	7595	3603	3510	1691	1884
6811	7745	6760	6744	6743	6771
3784	7543	475	250	3634	3757
7724	3372	7533	7522	7568	7672
7723	3370	7532	7521	7567	7671
7647	3328	7558	7497	7559	7668
7725	3373	7534	7523	7569	7673
7725	3373	7534	7523	7569	7673
2201	7625	3770	3713	2076	2309
513	7612	3843	3833	1909	382
332	7702	3881	3794	1949	733
1925	7613	3584	3505	1850	1964
1891	7597	3565	3492	1826	1921
1792	7590	3600	3507	1688	1879
1798	7596	3604	3511	1692	1885
2204	7612	3779	3708	2073	2318
1916	7629	3607	3529	1879	1981
1908	7621	3599	3521	1871	1973
2039	7564	3580	3512	1977	2012
475	7647	3800	3696	1889	24
1851	7556	3466	3377	1771	1954
1853	7589	3631	3560	735	1880
1715	7579	3631	3538	1621	1824
3786	7527	585	558	3649	3760
6833	7726	6798	6742	6782	6785
3720	7523	877	809	3568	3706
88	7689	3768	3702	1810	477
7725	2563	7559	7554	7606	7662
3720	7523	877	809	3568	3706
317	7684	3821	3721	1753	272
3697	7497	430	197	3563	3666
457	7649	3798	3701	1606	480
288	7646	3796	3712	1731	250
209	7686	3817	3748	1821	426
1846	7553	3461	3372	1766	1949
6961	7864	6833	6912	6910	6922
363	7716	3860	3765	2006	744
3733	7534	293	418	3583	3716
2096	7672	3803	3706	1981	2170
3623	7753	4214	4156	3367	3656
1715	7592	3621	3528	1708	1800
1807	7604	3638	3533	1827	1959
7726	2564	7560	7555	7607	7663
473	7655	3824	3752	1784	422
	7700	3810	3733	1786	457
7700		7547	7553	7614	7638
3810	7547		447	3656	3779

3733	7553	447		3577	3704
1786	7614	3656	3577		1879
457	7638	3779	3704	1879	
7724	2562	7558	7553	7605	7661
3761	7518	441	173	3599	3733
3761	7518	441	173	3599	3733
7691	19	7538	7544	7605	7629
1907	7620	3598	3520	1870	1972
1857	7591	3631	3560	737	1882
3662	7578	1178	1150	3554	3670
3805	7504	606	515	3662	3767
1707	7571	3623	3530	1613	1816
1851	7558	3466	3377	1771	1954
68	7675	3809	3739	1778	474
2277	7697	3706	3642	2028	2419
7799	3238	7603	7607	7701	7749
202	7656	3865	3782	1881	597
7724	2562	7558	7553	7605	7661
7723	2561	7557	7552	7604	7660
2202	7610	3777	3706	2071	2316
1914	7581	3598	3545	836	1858
6818	7739	6772	6755	6756	6781
2210	7605	3723	3646	2073	2324
2210	7605	3723	3646	2073	2324
7725	2563	7559	7554	7606	7662
308	7677	3814	3714	1746	263
290	7665	3802	3721	1730	247
1846	7553	3461	3372	1766	1949
3764	7544	442	202	3621	3721
3807	7552	40	445	3654	3778
7685	2314	7580	7597	7592	7628
1790	7588	3598	3504	1686	1877
1823	7609	3639	3558	1827	1973
1967	7581	3629	3542	698	2022
1922	7633	3613	3535	1885	1987
1816	7585	3618	3537	1621	1877
1814	7583	3616	3535	1619	1875
1845	7612	3647	3566	1650	1906
6858	7757	6811	6783	6798	6816
3776	7520	1000	1004	3645	3770
1908	7621	3599	3521	1871	1973
2265	7655	3749	3684	2055	2411
7692	20	7539	7545	7606	7630
7784	669	7617	7635	7698	7722
3727	7571	583	567	3608	3691
1683	7595	3634	3539	1667	1780
7785	2367	7679	7682	7683	7715
2205	7613	3780	3709	2074	2319
2211	7619	3786	3715	2080	2325
1740	7588	3654	3561	1642	1849
172	7687	3762	3704	1808	427
6865	7749	6866	6844	6803	6827
1927	7602	3612	3565	851	1868
6801	7692	6784	6734	6753	6753
337	7660	3784	3709	1773	246

334	7657	3781	3706	1770	243
2283	7680	3729	3668	2000	2395
1726	7600	3632	3539	1719	1811
7683	2312	7578	7595	7590	7626
7684	2313	7577	7594	7591	7627
7782	2364	7676	7679	7680	7712
1916	7614	3575	3496	1843	1932

MOD1-EC6946	MOD1-EC6966	MOD1-EC6978	MOD1-EC6529	MOD1-EC6332	MOD1-EC6385
7574	834	834	7545	3523	3583
7623	3562	3562	7599	1949	1941
7617	3617	3617	7589	1937	489
7775	3429	3429	7705	3786	3830
7525	367	367	7507	3486	3497
25522	24925	24925	25515	25034	24985
7623	3561	3561	7601	1922	1700
7622	3543	3543	7605	1945	1846
7603	3576	3576	7597	1964	1916
25521	24924	24924	25514	25033	24984
7686	3738	3738	7654	1871	1772
7768	3698	3698	7730	2235	2180
2623	7550	7550	2303	7634	7576
7548	3474	3474	7510	1858	1767
7617	3541	3541	7600	1859	1842
7880	6983	6983	7834	6905	6896
7561	3546	3546	7552	1856	1737
7724	3678	3678	7681	2227	1990
7724	3678	3678	7681	2227	1990
7670	3616	3616	7651	1955	1737
7822	4189	4189	7757	3548	3340
7652	3719	3719	7602	2107	2047
2659	7589	7589	2535	7713	7697
7586	3558	3558	7574	1857	1778
7714	3746	3746	7687	1933	1885
7682	3740	3740	7653	1921	1811
2654	7526	7526	501	7643	7612
7632	3583	3583	7604	174	1853
2598	7637	7637	2356	7682	7656
2598	7637	7637	2356	7682	7656
7587	3404	3404	7550	1742	1740
31230	30900	30900	31198	30975	31010
3376	7576	7576	3232	7694	7688
3402	7704	7704	3369	7777	7780
7577	1153	1153	7561	3506	3543
7577	1153	1153	7561	3506	3543
11	7515	7515	2556	7648	7592
3356	7650	7650	3325	7767	7761
7670	3732	3732	7641	1973	1887
7710	3738	3738	7683	1935	1881
7653	3720	3720	7603	2108	2048
7546	576	576	7510	3637	3613
3591	7474	7474	3407	7608	7594
7775	6751	6751	7723	6746	6771
7759	6770	6770	7718	6727	6759
7544	741	741	7519	3513	3511
7675	3885	3885	7619	2051	1964
7788	6799	6799	7747	6757	6789
7783	3722	3722	7754	2094	2006
3647	7472	7472	3448	7604	7604
7783	6750	6750	7745	6707	6743
3499	7488	7488	3363	7602	7557
7658	3724	3724	7608	2113	2053
7614	3654	3654	7589	2116	2010

7611	3538	3538	7598	1946	1857
7554	3507	3507	7512	1921	1815
7613	3599	3599	7597	1887	1709
7645	3551	3551	7611	26	1803
3376	7647	7647	3343	7737	7741
41	7495	7495	2585	7622	7566
7596	3586	3586	7582	1876	425
7616	3525	3525	7606	1930	1845
7591	3557	3557	7557	1954	1881
7624	3501	3501	7609	1955	1860
7598	3586	3586	7584	1876	425
7768	6797	6797	7729	6730	6762
3427	7515	7515	3300	7667	7653
7656	3560	3560	7622	35	1810
7653	3718	3718	7603	2104	2044
3496	7487	7487	3360	7601	7556
3497	7488	7488	3361	7602	7557
7760	3698	3698	7722	2244	2217
7623	3562	3562	7599	1949	1941
7656	3541	3541	7624	58	1829
2644	7540	7540	286	7641	7612
7654	3727	3727	7604	2121	2063
7627	3807	3807	7541	2105	2001
3384	7654	7654	3351	7742	7746
2598	7634	7634	2355	7679	7653
7666	6779	6779	7614	6772	6781
7614	3523	3523	7602	1927	1838
7613	3596	3596	7593	1911	477
3378	7579	7579	3234	7697	7691
3500	7491	7491	3364	7605	7560
3366	7583	7583	3232	7703	7697
3365	7582	7582	3231	7702	7696
3369	7586	7586	3235	7706	7700
3375	7575	7575	3231	7694	7688
7725	3666	3666	7686	2292	2003
2624	7551	7551	2304	7635	7577
7714	3805	3805	7702	2082	2043
2578	7579	7579	641	7686	7657
7660	3722	3722	7610	2100	2056
7692	3746	3746	7657	1874	1772
7679	3750	3750	7647	1943	1825
7741	3787	3787	7692	1914	1890
7610	3591	3591	7593	1914	480
7743	3795	3795	7696	1933	1887
3369	7586	7586	3235	7706	7700
7786	6617	6617	7771	6659	6679
7761	6776	6776	7720	6729	6763
7684	3747	3747	7650	1891	1780
7531	320	320	7522	3540	3559
7558	3486	3486	7519	1871	1780
7566	890	890	7555	3563	3548
3374	7574	7574	3230	7692	7686
7551	507	507	7547	3617	3644
7875	6978	6978	7829	6900	6891
7671	3741	3741	7640	1795	1668

3375	7581	7581	3231	7700	7693
7741	3790	3790	7694	1928	1882
7701	3760	3760	7661	1935	1914
7499	328	328	7485	3585	3611
7867	6942	6942	7813	6863	6863
3499	7488	7488	3361	7602	7557
3372	7589	7589	3238	7709	7703
2648	7547	7547	274	7646	7619
7713	3738	3738	7685	1942	1886
7607	3605	3605	7604	1876	741
3376	7647	7647	3343	7737	7741
7713	3734	3734	7662	2065	2083
7717	3745	3745	7687	1932	1882
7693	3771	3771	7661	1952	1837
7783	6614	6614	7768	6656	6676
7560	449	449	7526	3592	3607
2580	7581	7581	643	7688	7659
7681	3752	3752	7649	1945	1827
7653	3696	3696	7599	2080	2058
7613	3596	3596	7595	1911	477
7604	3590	3590	7588	1878	1700
7710	3738	3738	7683	1935	1881
7650	3556	3556	7609	76	1841
7595	3570	3570	7573	1923	533
7765	6776	6776	7724	6733	6765
2585	7585	7585	648	7692	7663
3499	7490	7490	3363	7604	7559
7784	6615	6615	7769	6657	6677
7750	6761	6761	7709	6710	6746
7785	6616	6616	7770	6658	6678
7506	192	192	7486	3504	3533
7731	3821	3821	7717	2098	2059
7723	3767	3767	7689	1905	1859
7682	3639	3639	7632	2190	1837
7706	3770	3770	7654	2193	2096
7608	599	599	7587	3663	3702
7608	599	599	7587	3663	3702
2641	7537	7537	283	7638	7609
7619	3535	3535	7604	1916	1831
7569	529	529	7541	3645	3652
7567	3579	3579	7554	1865	414
7547	141	141	7531	3557	3589
7691	3754	3754	7655	1882	1772
7589	1295	1295	7564	3642	3664
7675	3765	3765	7632	2008	1898
7772	6750	6750	7718	6739	6764
7561	3484	3484	7522	1870	1779
7783	6804	6804	7742	6757	6781
7602	3563	3563	7591	1869	1739
7543	809	809	7517	3541	3572
7613	3599	3599	7597	1887	1709
7513	474	474	7496	3506	3519
3498	7489	7489	3362	7603	7558
3498	7489	7489	3362	7603	7558
3499	7490	7490	3363	7604	7559

3497	7488	7488	3361	7602	7557
7766	6790	6790	7725	6743	6767
7635	3658	3658	7596	2055	2048
7666	3876	3876	7610	2042	1955
7611	3594	3594	7593	1909	475
2623	7552	7552	2301	7636	7578
7677	3748	3748	7645	1985	1895
7593	3583	3583	7590	1809	76
7623	3534	3534	7586	1885	1739
7777	6744	6744	7736	6699	6741
7547	195	195	7534	3577	3609
3499	7489	7489	3363	7603	7558
3497	7488	7488	3361	7602	7557
3460	7464	7464	3319	7593	7571
3500	7490	7490	3364	7604	7559
3500	7490	7490	3364	7604	7559
7666	3725	3725	7616	2145	2109
7659	3869	3869	7603	2035	1948
7716	3830	3830	7693	2067	2022
7617	3529	3529	7604	1936	1843
7602	3507	3507	7588	1918	1824
7620	3531	3531	7581	1880	1736
7624	3535	3535	7587	1886	1740
7653	3720	3720	7603	2108	2048
7654	3560	3560	7620	33	1808
7646	3552	3552	7612	25	1802
7562	3543	3543	7555	2028	1975
7672	3743	3743	7638	1982	1892
7584	3403	3403	7547	1741	1739
7587	3583	3583	7580	1799	28
7579	3562	3562	7570	1849	1701
7525	559	559	7518	3590	3619
7771	6745	6745	7717	6740	6765
7540	806	806	7514	3539	3569
7707	3736	3736	7680	1933	1879
3	7513	7513	2554	7646	7590
7540	806	806	7514	3539	3569
7703	3742	3742	7675	1907	1803
7508	192	192	7488	3502	3533
7671	3741	3741	7640	1795	1668
7681	3750	3750	7637	1878	1773
7724	3774	3774	7677	1923	1880
7581	3398	3398	7544	1736	1734
7890	6914	6914	7855	6875	6886
7721	3807	3807	7707	2091	2063
7555	424	424	7525	3527	3548
7714	3730	3730	7663	2028	1999
7809	4178	4178	7744	3537	3329
7591	3552	3552	7583	1858	1728
7620	3561	3561	7595	1955	1919
6	7514	7514	2555	7647	7591
7674	3788	3788	7646	1950	1811
7724	3761	3761	7691	1907	1857
2562	7518	7518	19	7620	7591
7558	441	441	7538	3598	3631

7553	173	173	7544	3520	3560
7605	3599	3599	7605	1870	737
7661	3733	3733	7629	1972	1882
	7512	7512	2553	7645	7589
7512			7509	3551	3583
7512			7509	3551	3583
2553	7509	7509		7611	7582
7645	3551	3551	7611		1801
7589	3583	3583	7582	1801	
7589	1157	1157	7569	3504	3543
7509	458	458	7495	3610	3632
7571	3554	3554	7562	1841	1693
7586	3403	3403	7549	1741	1739
7712	3771	3771	7666	1918	1862
7724	3657	3657	7688	2261	1983
3373	7574	7574	3229	7692	7686
7687	3816	3816	7647	1978	1944
4	7512	7512	2553	7645	7589
3	7511	7511	2552	7644	7588
7651	3718	3718	7601	2106	2046
7576	3572	3572	7572	1852	135
7781	6755	6755	7730	6713	6747
7635	3658	3658	7596	2055	2048
7635	3658	3658	7596	2055	2048
5	7513	7513	2554	7646	7590
7696	3735	3735	7668	1900	1796
7692	3757	3757	7656	1882	1773
7581	3398	3398	7544	1736	1734
7553	143	143	7535	3559	3592
7564	451	451	7543	3595	3628
2625	7552	7552	2305	7636	7578
7618	3529	3529	7579	1878	1734
7625	3578	3578	7600	1969	1951
7594	3569	3569	7572	1922	532
7658	3566	3566	7624	39	1816
7588	3560	3560	7576	1859	1780
7586	3558	3558	7574	1857	1778
7615	3589	3589	7603	1888	1809
7792	6785	6785	7748	6753	6784
7545	1009	1009	7511	3614	3624
7646	3552	3552	7612	11	1802
7692	3695	3695	7646	2155	2009
2554	7510	7510	11	7612	7583
2597	7598	7598	660	7705	7676
7574	565	565	7562	3555	3586
7589	3563	3563	7586	1862	1745
2600	7639	7639	2358	7684	7658
7654	3721	3721	7604	2109	2049
7660	3727	3727	7610	2115	2055
7586	3585	3585	7579	1872	1722
7708	3738	3738	7678	1925	1877
7785	6846	6846	7740	6781	6793
7597	3582	3582	7593	1863	144
7741	6730	6730	7683	6716	6738
7680	3738	3738	7651	1919	1809

7677	3735	3735	7648	1916	1806
7706	3687	3687	7671	2202	1957
7602	3563	3563	7591	1869	1739
2623	7550	7550	2303	7634	7576
2624	7549	7549	2304	7635	7577
2597	7636	7636	2355	7681	7655
7615	3524	3524	7605	1929	1844

MOD1-EC5700	MOD1-EC5706	MOD1-EC5440	MOD1-EC5737	MOD1-EC5722	MOD1-EC3793
993	1047	3605	3406	3765	3675
3558	3621	1695	1865	1830	2136
3615	3625	1752	1838	1942	2127
3453	3474	3764	3665	3913	3820
1076	536	3497	3332	3689	3574
24946	24940	24998	25015	25042	24984
3516	3640	1511	1447	1795	2026
3482	3614	1804	1794	1925	2334
3536	3618	1887	1849	1984	2384
24945	24939	24997	25014	25041	24983
3645	3792	1664	1830	299	2307
3643	3746	2097	2250	2354	1704
7587	7528	7576	7581	7660	7717
3486	3553	1861	1896	1919	2277
3489	3626	1779	1820	1973	1957
6978	6973	6873	6832	6995	6959
3464	3612	67	1745	1752	2137
3635	3717	2133	2102	2303	203
3635	3717	2133	2102	2303	203
3597	3721	1702	1691	1934	2122
4190	4228	3538	3508	3630	3654
3685	3756	1859	2125	2213	2010
7646	7586	7672	7676	7774	7813
3508	3635	1416	1616	1801	2008
3642	3779	1735	1879	95	2309
3646	3775	1713	1876	352	2363
7584	7506	7582	7568	7699	7727
3530	3642	1900	1807	1941	2324
7707	7650	7657	7641	7774	7797
7707	7650	7657	7641	7774	7797
3388	3519	1701	27	1849	2106
30918	30935	30920	30973	31011	30966
7626	7549	7653	7640	7770	7797
7768	7690	7723	7744	7883	7885
62	1115	3500	3393	3652	3607
62	1115	3500	3393	3652	3607
7592	7512	7574	7589	7715	7727
7713	7634	7699	7704	7857	7864
3673	3771	1817	1957	484	2416
3636	3773	1723	1873	99	2295
3686	3757	1860	2126	2214	2011
1326	734	3604	3436	3767	3724
7536	7486	7560	7553	7696	7709
6790	6795	6740	6693	6839	6820
6801	6782	6726	6681	6817	6806
1179	681	3555	3431	3683	3638
3764	3792	1884	2036	523	2475
6831	6811	6756	6711	6847	6836
3723	3815	2140	2239	2190	2538
7536	7484	7558	7549	7696	7713
6783	6762	6704	6661	6819	6786
7550	7441	7519	7572	7692	7707
3690	3761	1865	2131	2219	2016
3648	3747	2022	2149	2049	2427

3411	3603	1805	1793	1946	2338
3514	3599	1877	2008	1907	2282
3551	3669	180	1705	1765	2142
3504	3610	1841	1741	1918	2261
7717	7634	7685	7705	7848	7847
7572	7492	7550	7565	7689	7707
3542	3630	1779	1818	1936	2061
3478	3611	1793	1779	1935	2321
3450	3581	1847	1823	1979	2335
3415	3540	1774	1806	1934	2283
3542	3630	1779	1818	1936	2061
6793	6769	6737	6684	6806	6806
7575	7501	7620	7605	7741	7760
3513	3619	1850	1752	1929	2272
3682	3755	1856	2122	2210	2017
7549	7440	7518	7571	7691	7706
7550	7441	7519	7572	7692	7707
3633	3746	2102	2283	2333	1747
3558	3621	1695	1865	1830	2136
3512	3622	1861	1761	1938	2281
7585	7524	7587	7569	7689	7715
3693	3764	1849	2141	2229	2000
3664	3806	1947	2124	699	2573
7724	7641	7690	7710	7853	7852
7704	7647	7654	7638	7771	7794
6812	6801	6751	6713	6821	6857
3456	3595	1779	1755	1914	2313
3595	3655	1729	1816	1955	2116
7629	7552	7656	7643	7773	7800
7553	7444	7522	7575	7695	7710
7635	7556	7662	7649	7781	7806
7634	7555	7661	7648	7780	7805
7638	7559	7665	7652	7784	7809
7625	7548	7653	7640	7770	7797
3642	3704	2128	2143	2327	156
7588	7529	7577	7582	7661	7718
3712	3851	1919	2066	359	2449
7638	7560	7625	7610	7740	7756
3694	3762	1862	2114	2194	2049
3639	3793	1665	1831	301	2318
3661	3779	1732	1894	382	2380
3698	3804	1710	1818	197	2316
3602	3655	1734	1821	1953	2109
3688	3812	1723	1841	159	2321
7638	7559	7665	7652	7784	7809
6648	6629	6652	6593	6755	6722
6805	6788	6728	6685	6815	6804
3631	3785	1682	1846	316	2309
1186	533	3557	3400	3754	3642
3499	3565	1874	1909	1932	2290
564	853	3558	3402	3698	3603
7624	7547	7651	7638	7768	7795
1084	357	3631	3527	3823	3698
6973	6968	6868	6827	6990	6954
3659	3801	1610	1830	481	2255

7631	7554	7657	7644	7775	7803
3683	3807	1718	1836	154	2316
3605	3759	1800	1904	265	2340
1233	191	3609	3443	3801	3696
6943	6922	6837	6794	6949	6925
7550	7441	7519	7572	7692	7707
7641	7562	7668	7655	7787	7812
7593	7531	7592	7576	7694	7722
3627	3778	1732	1880	101	2300
3556	3661	1619	1775	1777	2038
7717	7634	7685	7705	7848	7847
3667	3768	2017	2105	2161	1435
3631	3778	1716	1834	182	2316
3673	3802	1741	1903	391	2389
6645	6626	6649	6590	6752	6719
1145	590	3591	3435	3762	3682
7640	7562	7627	7612	7742	7758
3663	3781	1734	1896	384	2382
3660	3753	1874	2132	2224	1965
3595	3655	1729	1816	1955	2116
3542	3660	171	1696	1756	2133
3636	3773	1723	1873	99	2295
3508	3578	1882	1787	1923	2298
3581	3634	1747	1816	1960	2092
6807	6788	6732	6687	6823	6812
7644	7566	7631	7616	7746	7762
7552	7443	7521	7574	7694	7709
6646	6627	6650	6591	6753	6720
6790	6773	6709	6664	6818	6789
6647	6628	6651	6592	6754	6721
1142	459	3518	3364	3669	3612
3728	3867	1935	2082	375	2467
3662	3801	1695	1811	152	2291
3586	3666	1954	2055	2170	419
3697	3802	2024	2082	2295	728
1207	653	3713	3542	3879	3761
1207	653	3713	3542	3879	3761
7582	7521	7584	7566	7686	7712
3470	3618	1777	1767	1928	2320
1162	640	3631	3483	3807	3730
3562	3617	1701	1888	1899	2058
1150	393	3571	3417	3739	3667
3645	3795	1672	1836	311	2320
559	1217	3627	3511	3788	3712
3722	3742	1814	1963	454	2436
6787	6794	6733	6686	6832	6813
3498	3563	1873	1908	1931	2289
6835	6816	6756	6705	6839	6830
3525	3638	332	1739	1738	2133
645	797	3559	3441	3728	3606
3551	3669	180	1705	1765	2142
977	456	3508	3408	3696	3594
7551	7442	7520	7573	7693	7708
7551	7442	7520	7573	7693	7708
7552	7443	7521	7574	7694	7709

7550	7441	7519	7572	7692	7707
6821	6802	6742	6691	6825	6814
3686	3757	1862	2032	2220	2020
3755	3783	1875	2027	514	2466
3593	3653	1727	1814	1953	2114
7589	7529	7578	7583	7662	7719
3684	3786	1829	1967	487	2433
3535	3632	1709	1763	1870	1999
3472	3604	253	1742	1809	2156
6777	6757	6702	6657	6806	6786
1170	412	3590	3440	3756	3678
7551	7442	7520	7573	7693	7708
7550	7441	7519	7572	7692	7707
7530	7513	7507	7507	7616	7682
7552	7443	7521	7574	7694	7709
7552	7443	7521	7574	7694	7709
3685	3746	1919	2173	2203	1999
3748	3776	1868	2020	507	2459
3737	3876	1910	2037	320	2440
3468	3606	1795	1785	1943	2307
3461	3581	1779	1771	1909	2309
3469	3601	248	1737	1804	2153
3473	3605	254	1743	1810	2157
3686	3757	1860	2126	2214	2011
3513	3619	1850	1750	1927	2270
3505	3611	1842	1742	1919	2262
3424	3556	1903	1869	2057	2412
3689	3791	1826	1964	492	2430
3387	3518	1700	26	1848	2105
3543	3632	1691	1735	1858	1979
3502	3632	14	1708	1727	2108
1067	437	3619	3501	3789	3672
6784	6789	6734	6687	6833	6814
642	794	3556	3438	3726	3602
3634	3771	1721	1871	97	2293
7590	7510	7572	7587	7713	7725
642	794	3556	3438	3726	3602
3665	3786	1700	1864	343	2322
1144	461	3518	3364	3669	3612
3659	3801	1610	1830	481	2255
3644	3767	1668	1833	276	2319
3685	3789	1734	1856	208	2314
3382	3513	1695	21	1843	2100
6923	6932	6857	6807	6961	6931
3724	3853	1935	2071	383	2465
1153	603	3548	3381	3734	3625
3641	3762	1948	2088	2094	1566
4179	4217	3527	3497	3619	3643
3514	3627	321	1728	1727	2122
3565	3624	1679	1851	1822	2124
7591	7511	7573	7588	7714	7726
3702	3807	1739	1913	467	2364
3662	3805	1707	1851	68	2277
7578	7504	7571	7558	7675	7697
1178	606	3623	3466	3809	3706

1150	515	3530	3377	3739	3642
3554	3662	1613	1771	1778	2028
3670	3767	1816	1954	474	2419
7589	7509	7571	7586	7712	7724
1157	458	3554	3403	3771	3657
1157	458	3554	3403	3771	3657
7569	7495	7562	7549	7666	7688
3504	3610	1841	1741	1918	2261
3543	3632	1693	1739	1862	1983
	1116	3494	3387	3667	3615
1116		3624	3518	3782	3692
3494	3624		1700	1719	2100
3387	3518	1700		1848	2105
3667	3782	1719	1848		2293
3615	3692	2100	2105	2293	
7624	7547	7651	7638	7768	7795
3707	3859	1769	1933	223	2376
7589	7509	7571	7586	7712	7724
7588	7508	7570	7585	7711	7723
3684	3755	1858	2124	2212	2009
3552	3605	1775	1795	1916	2033
6785	6745	6711	6667	6795	6793
3686	3757	1862	2032	2220	2020
3686	3757	1862	2032	2220	2020
7590	7510	7572	7587	7713	7725
3658	3779	1691	1857	334	2317
3650	3798	1673	1837	311	2320
3382	3513	1695	21	1843	2100
1152	395	3574	3421	3741	3669
1168	606	3620	3462	3806	3703
7589	7530	7578	7583	7662	7719
3467	3599	246	1735	1802	2151
3572	3622	1711	1883	1831	2148
3580	3633	1746	1815	1959	2091
3519	3625	1856	1756	1933	2276
3510	3637	1418	1618	1803	2010
3508	3635	1416	1616	1801	2008
3539	3666	1447	1647	1832	2039
6803	6826	6749	6703	6858	6821
699	858	3605	3502	3754	3678
3505	3611	1839	1741	1919	2262
3638	3738	2064	2107	2276	321
7570	7496	7563	7550	7667	7689
7657	7579	7644	7629	7759	7775
1006	533	3572	3454	3732	3623
3519	3633	218	1681	1695	2146
7709	7652	7659	7643	7776	7799
3687	3758	1861	2127	2215	2012
3693	3764	1867	2133	2221	2018
3525	3655	51	1729	1752	2125
3630	3771	1711	1829	171	2311
6877	6858	6774	6729	6865	6856
3562	3606	1786	1804	1932	2053
6763	6775	6703	6673	6801	6789
3644	3773	1712	1874	350	2361

3641	3770	1709	1871	347	2358
3640	3720	2084	2113	2288	174
3525	3638	332	1739	1738	2133
7587	7528	7576	7581	7660	7717
7588	7527	7577	7582	7661	7718
7706	7649	7656	7640	7773	7796
3477	3610	1792	1778	1934	2320

MOD1-EC54	MOD1-EC5522	MOD1-EC5194	MOD1-EC5196	Ecol_583	MOD1-EC3564	18.1-R1
7605	3808	7574	7573	3759	3572	6785
7682	1891	7623	7622	1885	1995	6662
7672	2073	7617	7616	2103	569	6778
7859	3979	7775	7774	3833	3820	7004
7569	3741	7525	7524	3669	3491	6741
25534	25044	25522	25521	24967	24972	25031
7693	1879	7623	7622	2073	1768	6705
7715	2009	7622	7621	2214	1866	6668
7694	2073	7603	7602	2243	1950	6779
25533	25043	25521	25520	24966	24971	25030
7758	411	7686	7685	2179	1819	6795
7837	2423	7768	7767	1863	2208	6851
3297	7644	2623	2622	7630	7568	7739
7636	2011	7548	7547	2179	1837	6666
7681	2045	7617	7616	2024	1892	6781
8035	6948	7880	7879	6964	6900	442
7644	1802	7561	7560	1896	1784	6709
7789	2391	7724	7723	1974	2042	6780
7789	2391	7724	7723	1974	2042	6780
7745	2015	7670	7669	2260	1789	6825
7868	3670	7822	7821	3805	3361	6965
7704	2283	7652	7651	3	2099	6818
3383	7770	2659	2658	7722	7688	7794
7664	1915	7586	7585	2107	1846	6782
7794	238	7714	7713	2236	1942	6817
7756	479	7682	7681	2241	1860	6793
3305	7680	2654	2653	7625	7604	7688
7674	2001	7632	7631	2154	1895	6763
3288	7747	2598	2597	7695	7645	7976
3288	7747	2598	2597	7695	7645	7976
7639	1934	7587	7586	2125	1796	6668
31162	30999	31232	31231	30944	31006	30807
17	7772	3376	3375	7705	7672	7912
1902	7879	3402	3401	7790	7776	7990
7615	3713	7577	7576	3679	3546	6780
7615	3713	7577	7576	3679	3546	6780
3376	7690	11	10	7654	7579	7784
2012	7847	3356	3355	7778	7749	7924
7759	600	7670	7669	2311	1857	6791
7794	236	7710	7709	2228	1936	6813
7705	2284	7653	7652	4	2100	6819
7567	3850	7546	7545	3782	3642	6770
772	7683	3591	3590	7620	7585	7841
7924	6830	7775	7774	6835	6753	276
7911	6807	7759	7758	6832	6744	105
7585	3736	7544	7543	3683	3494	6731
7747	668	7675	7674	2397	1959	6744
7940	6837	7788	7787	6862	6774	135
7885	2271	7783	7782	2449	2076	6939
843	7690	3647	3646	7624	7595	7827
7939	6834	7783	7782	6810	6726	37
259	7690	3499	3498	7572	7529	7873
7710	2289	7658	7657	9	2105	6824
7732	2156	7614	7613	2352	2072	6774

7712	2016	7611	7610	2210	1871	6688
7661	1995	7554	7553	2176	1876	6698
7685	1815	7613	7612	1922	1791	6753
7692	1978	7645	7644	2106	1854	6713
1879	7847	3376	3375	7755	7733	7944
3405	7714	41	40	7628	7553	7764
7686	2037	7596	7595	2135	515	6769
7709	2016	7616	7615	2195	1835	6653
7685	2060	7591	7590	2243	1886	6640
7712	2015	7624	7623	2180	1880	6649
7688	2037	7598	7597	2135	515	6771
7922	6790	7768	7767	6836	6724	175
175	7736	3427	3426	7672	7637	7920
7703	1989	7656	7655	2115	1861	6722
7705	2280	7653	7652	10	2104	6821
256	7689	3496	3495	7571	7528	7872
257	7690	3497	3496	7572	7529	7873
7836	2404	7760	7759	1916	2245	6862
7682	1891	7623	7622	1885	1995	6662
7701	1998	7656	7655	2134	1844	6698
3278	7667	2644	2643	7621	7602	7706
7710	2293	7654	7653	19	2115	6830
7632	678	7627	7626	2456	2005	6776
1886	7852	3384	3383	7760	7738	7951
3285	7744	2598	2597	7692	7642	7971
7797	6842	7666	7665	6848	6764	512
7709	1995	7614	7613	2193	1863	6674
7686	2043	7613	7612	2082	560	6794
19	7775	3378	3377	7708	7675	7915
260	7693	3500	3499	7575	7532	7876
75	7776	3366	3365	7712	7681	7919
74	7775	3365	3364	7711	7680	7918
78	7779	3369	3368	7715	7684	7922
18	7772	3375	3374	7705	7672	7912
7793	2408	7725	7724	2055	2073	6794
3298	7645	2624	2623	7631	7569	7740
7814	479	7714	7713	2364	2094	6756
3277	7716	2578	2577	7678	7647	7736
7714	2264	7660	7659	46	2114	6828
7761	406	7692	7691	2190	1825	6797
7770	505	7679	7678	2260	1875	6808
7797	342	7741	7740	2221	1926	6825
7684	2056	7610	7609	2077	561	6790
7805	304	7743	7742	2228	1942	6823
78	7779	3369	3368	7715	7684	7922
7941	6807	7786	7785	6756	6664	224
7911	6805	7761	7760	6828	6748	105
7753	433	7684	7683	2183	1817	6792
7597	3800	7531	7530	3705	3550	6754
7646	2024	7558	7557	2192	1850	6677
7593	3757	7566	7565	3707	3530	6786
15	7770	3374	3373	7703	7670	7911
7610	3869	7551	7550	3749	3621	6795
8030	6943	7875	7874	6959	6895	437
7743	570	7671	7670	2101	1725	6767

26	7777	3375	3374	7709	7677	7915
7801	299	7741	7740	2223	1937	6817
7773	389	7701	7700	2251	1910	6813
7554	3855	7499	7498	3751	3587	6764
8019	6919	7867	7866	6930	6851	442
259	7690	3499	3498	7572	7529	7875
81	7782	3372	3371	7718	7687	7925
3282	7675	2648	2647	7630	7609	7711
7800	249	7713	7712	2221	1939	6812
7700	1883	7607	7606	2081	840	6755
1879	7847	3376	3375	7755	7733	7944
7765	2264	7713	7712	1676	2137	6797
7800	315	7717	7716	2225	1939	6816
7782	514	7693	7692	2269	1887	6817
7938	6804	7783	7782	6753	6661	221
7578	3841	7560	7559	3739	3611	6745
3279	7718	2580	2579	7680	7649	7738
7772	507	7681	7680	2262	1877	6810
7707	2294	7653	7652	76	2098	6817
7688	2043	7613	7612	2082	560	6794
7676	1806	7604	7603	1913	1782	6744
7794	236	7710	7709	2228	1936	6813
7682	2022	7650	7649	2153	1887	6702
7673	2059	7595	7594	2072	614	6807
7915	6813	7765	7764	6838	6750	111
3284	7723	2585	2584	7684	7653	7743
259	7692	3499	3498	7574	7531	7875
7939	6805	7784	7783	6754	6662	222
7904	6807	7750	7749	6813	6731	98
7940	6806	7785	7784	6755	6663	223
7554	3759	7506	7505	3677	3500	6721
7829	495	7731	7730	2382	2112	6771
7796	276	7723	7722	2198	1906	6809
7745	2246	7682	7681	1876	1887	6791
7756	2360	7706	7705	1918	2148	6752
7651	3935	7608	7607	3839	3671	6796
7651	3935	7608	7607	3839	3671	6796
3275	7664	2641	2640	7618	7599	7703
7714	1994	7619	7618	2189	1857	6663
7600	3862	7569	7568	3803	3616	6773
7660	1999	7567	7566	2090	497	6763
7582	3816	7547	7546	3734	3556	6757
7754	426	7691	7690	2200	1831	6788
7622	3841	7589	7588	3782	3611	6782
7739	602	7675	7674	2300	1883	6705
7919	6827	7772	7771	6826	6746	273
7649	2023	7561	7560	2191	1849	6678
7933	6827	7783	7782	6860	6766	135
7684	1788	7602	7601	1908	1809	6721
7571	3785	7543	7542	3692	3543	6740
7685	1815	7613	7612	1922	1791	6753
7556	3742	7513	7512	3657	3539	6736
258	7691	3498	3497	7573	7530	7874
258	7691	3498	3497	7573	7530	7874
259	7692	3499	3498	7574	7531	7875

257	7690	3497	3496	7572	7529	7873
7918	6813	7766	7765	6846	6752	119
7697	2290	7635	7634	120	2100	6823
7738	659	7666	7665	2388	1950	6735
7686	2041	7611	7610	2080	558	6792
3297	7646	2623	2622	7632	7570	7741
7765	610	7677	7676	2330	1872	6797
7689	1952	7593	7592	2078	181	6745
7688	1859	7623	7622	1927	1821	6722
7928	6826	7777	7776	6806	6728	26
7586	3846	7547	7546	3735	3598	6769
259	7691	3499	3498	7573	7530	7874
257	7690	3497	3496	7572	7529	7873
236	7614	3460	3459	7562	7569	7865
260	7692	3500	3499	7574	7531	7875
260	7692	3500	3499	7574	7531	7875
7714	2285	7666	7665	91	2151	6804
7731	652	7659	7658	2381	1943	6728
7803	472	7716	7715	2345	2075	6755
7713	2016	7617	7616	2183	1861	6663
7698	1990	7602	7601	2183	1838	6657
7687	1854	7620	7619	1922	1818	6717
7690	1860	7624	7623	1928	1822	6723
7705	2284	7653	7652	4	2100	6819
7701	1987	7654	7653	2115	1859	6722
7693	1979	7646	7645	2107	1853	6714
7664	2132	7562	7561	2296	1971	6662
7758	615	7672	7671	2327	1877	6786
7634	1933	7584	7583	2124	1795	6667
7684	1940	7587	7586	2044	133	6744
7659	1777	7579	7578	1866	1783	6719
7595	3837	7525	7524	3734	3594	6737
7918	6824	7771	7770	6829	6747	270
7568	3783	7540	7539	3689	3540	6739
7791	234	7707	7706	2226	1934	6810
3374	7688	5	4	7652	7577	7782
7568	3783	7540	7539	3689	3540	6739
7778	453	7703	7702	2196	1839	6817
7554	3759	7508	7507	3679	3500	6725
7743	570	7671	7670	2101	1725	6767
7733	417	7681	7680	2194	1821	6769
7780	353	7724	7723	2215	1929	6801
7633	1928	7581	7580	2119	1790	6662
8042	6952	7890	7889	6949	6877	460
7819	503	7721	7720	2378	2099	6763
7585	3790	7555	7554	3704	3508	6724
7762	2189	7714	7713	1590	2047	6795
7857	3659	7809	7808	3794	3350	6955
7674	1777	7591	7590	1897	1798	6710
7680	1883	7620	7619	1885	1997	6658
3375	7689	6	5	7653	7578	7783
7757	549	7674	7673	2250	1860	6774
7799	202	7724	7723	2202	1914	6818
3238	7656	2562	2561	7610	7581	7739
7603	3865	7558	7557	3777	3598	6772

7607	3782	7553	7552	3706	3545	6755
7701	1881	7605	7604	2071	836	6756
7749	597	7661	7660	2316	1858	6781
3373	7687	4	3	7651	7576	7781
7574	3816	7512	7511	3718	3572	6755
7574	3816	7512	7511	3718	3572	6755
3229	7647	2553	2552	7601	7572	7730
7692	1978	7645	7644	2106	1852	6713
7686	1944	7589	7588	2046	135	6747
7624	3707	7589	7588	3684	3552	6785
7547	3859	7509	7508	3755	3605	6745
7651	1769	7571	7570	1858	1775	6711
7638	1933	7586	7585	2124	1795	6667
7768	223	7712	7711	2212	1916	6795
7795	2376	7724	7723	2009	2033	6793
	7770	3373	3372	7703	7670	7910
7770		7687	7686	2282	2001	6836
3373	7687		1	7651	7576	7781
3372	7686	1		7650	7575	7780
7703	2282	7651	7650		2098	6817
7670	2001	7576	7575	2098		6735
7910	6836	7781	7780	6817	6735	
7697	2290	7635	7634	120	2100	6823
7697	2290	7635	7634	120	2100	6823
3374	7688	5	4	7652	7577	7782
7771	444	7696	7695	2189	1832	6810
7757	426	7692	7691	2200	1832	6789
7633	1928	7581	7580	2119	1790	6662
7586	3818	7553	7552	3737	3559	6759
7607	3862	7564	7563	3774	3597	6784
3299	7646	2625	2624	7632	7570	7741
7685	1852	7618	7617	1920	1816	6714
7681	1907	7625	7624	1905	1995	6661
7672	2058	7594	7593	2071	613	6806
7705	1993	7658	7657	2121	1867	6728
7666	1917	7588	7587	2109	1848	6784
7664	1915	7586	7585	2107	1846	6782
7693	1946	7615	7614	2138	1877	6810
7957	6845	7792	7791	6836	6771	290
7554	3834	7545	7544	3771	3629	6740
7693	1979	7646	7645	2106	1853	6714
7761	2348	7692	7691	1907	2065	6779
3230	7648	2554	2553	7602	7573	7731
3296	7735	2597	2596	7697	7666	7755
7611	3772	7574	7573	3702	3548	6768
7667	1745	7589	7588	1923	1817	6707
3290	7749	2600	2599	7697	7647	7978
7706	2285	7654	7653	5	2101	6820
7712	2291	7660	7659	11	2107	6826
7671	1802	7586	7585	1883	1804	6734
7789	312	7708	7707	2222	1934	6807
7929	6833	7785	7784	6874	6778	161
7691	2014	7597	7596	2118	48	6752
7888	6796	7741	7740	6802	6717	379
7754	477	7680	7679	2239	1858	6791

7751	474	7677	7676	2236	1855	6788
7782	2364	7706	7705	1997	2007	6776
7684	1788	7602	7601	1908	1809	6721
3297	7644	2623	2622	7630	7568	7739
3298	7645	2624	2623	7631	7569	7740
3287	7746	2597	2596	7694	7644	7973
7710	2015	7615	7614	2194	1834	6652

MOD1-EC3605	KCJK1916	KCJK1866	MOD1-EC707	MOD1-EC1626	MOD1-EC1634
871	3689	3689	7575	3719	3761
3629	1885	1885	7624	1824	1820
3657	2113	2113	7618	1930	1902
3509	3783	3783	7776	3918	3927
904	3615	3615	7526	3684	3676
24880	24998	24998	25523	25040	25038
3635	2095	2095	7624	1876	1860
3584	2236	2236	7623	1875	1853
3643	2275	2275	7604	1929	1935
24879	24997	24997	25522	25039	25037
3751	2187	2187	7687	67	67
3714	1870	1870	7769	2275	2276
7544	7642	7642	2624	7660	7649
3582	2216	2216	7549	1844	1830
3601	1973	1973	7618	1916	1912
6958	6970	6970	7881	6983	6966
3603	1900	1900	7562	1716	1698
3699	1985	1985	7725	2355	2359
3699	1985	1985	7725	2355	2359
3708	2186	2186	7671	1902	1886
4214	3790	3790	7823	3677	3653
3772	121	121	7653	2190	2201
7588	7707	7707	2660	7782	7770
3604	2139	2139	7587	1763	1746
3752	2244	2244	7715	350	326
3739	2249	2249	7683	131	109
7522	7618	7618	2655	7703	7691
3642	2103	2103	7633	1921	1903
7668	7682	7682	2599	7756	7745
7668	7682	7682	2599	7756	7745
3503	2033	2033	7588	1858	1838
30915	30969	30969	31233	30996	31009
7556	7699	7699	3377	7773	7759
7702	7783	7783	3403	7879	7869
692	3681	3681	7578	3652	3650
692	3681	3681	7578	3652	3650
7548	7638	7638	12	7699	7695
7644	7771	7771	3357	7853	7841
3780	2319	2319	7671	258	258
3744	2236	2236	7711	344	318
3773	122	122	7654	2191	2202
1136	3728	3728	7547	3812	3788
7448	7614	7614	3592	7688	7681
6783	6841	6841	7776	6827	6811
6779	6838	6838	7760	6805	6789
1007	3661	3661	7545	3686	3676
3832	2405	2405	7676	341	323
6808	6868	6868	7789	6834	6819
3832	2473	2473	7784	2127	2110
7446	7618	7618	3648	7688	7681
6763	6816	6816	7784	6803	6789
7478	7566	7566	3500	7691	7677
3777	127	127	7659	2196	2207
3763	2374	2374	7615	1998	1987

3561	2232	2232	7612	1872	1862
3621	2214	2214	7555	1802	1804
3658	1932	1932	7614	1735	1717
3614	2055	2055	7646	1900	1882
7647	7748	7748	3377	7842	7832
7528	7612	7612	42	7673	7669
3645	2137	2137	7597	1891	1898
3581	2217	2217	7617	1862	1850
3562	2265	2265	7592	1906	1894
3518	2228	2228	7625	1861	1849
3645	2137	2137	7599	1891	1898
6775	6842	6842	7769	6790	6786
7511	7666	7666	3428	7744	7730
3623	2062	2062	7657	1911	1893
3769	128	128	7654	2189	2198
7477	7565	7565	3497	7690	7676
7478	7566	7566	3498	7691	7677
3706	1913	1913	7761	2272	2273
3629	1885	1885	7624	1824	1820
3620	2083	2083	7657	1914	1896
7528	7614	7614	2645	7693	7681
3780	137	137	7655	2200	2211
3780	2464	2464	7628	523	496
7653	7753	7753	3385	7847	7837
7665	7679	7679	2599	7753	7742
6785	6854	6854	7667	6844	6825
3558	2215	2215	7615	1835	1823
3673	2092	2092	7614	1909	1884
7559	7702	7702	3379	7776	7762
7481	7569	7569	3501	7694	7680
7563	7706	7706	3367	7784	7770
7562	7705	7705	3366	7783	7769
7566	7709	7709	3370	7787	7773
7555	7699	7699	3376	7773	7759
3695	2066	2066	7726	2368	2354
7545	7643	7643	2625	7661	7650
3831	2372	2372	7715	577	575
7573	7660	7660	2579	7742	7732
3781	164	164	7661	2188	2186
3752	2198	2198	7693	80	64
3762	2268	2268	7680	153	137
3797	2229	2229	7742	320	299
3671	2087	2087	7611	1904	1889
3787	2236	2236	7744	282	261
7566	7709	7709	3370	7787	7773
6626	6762	6762	7787	6741	6725
6785	6834	6834	7762	6803	6787
3736	2191	2191	7685	79	75
908	3645	3645	7532	3740	3742
3595	2229	2229	7559	1857	1843
548	3709	3709	7567	3695	3708
7554	7697	7697	3375	7771	7757
857	3751	3751	7552	3799	3806
6953	6965	6965	7876	6978	6961
3773	2109	2109	7672	295	287

7561	7703	7703	3376	7778	7764
3782	2231	2231	7742	277	256
3751	2261	2261	7702	355	335
999	3691	3691	7500	3778	3792
6913	6936	6936	7868	6937	6920
7478	7566	7566	3500	7691	7677
7569	7712	7712	3373	7790	7776
7535	7623	7623	2649	7698	7686
3738	2229	2229	7714	353	337
3644	2083	2083	7608	1756	1732
7647	7748	7748	3377	7842	7832
3755	1685	1685	7714	2171	2151
3757	2233	2233	7718	295	277
3774	2277	2277	7694	174	146
6623	6759	6759	7784	6738	6722
969	3685	3685	7561	3792	3781
7575	7662	7662	2581	7744	7734
3764	2270	2270	7682	155	139
3753	194	194	7654	2187	2198
3673	2092	2092	7614	1909	1884
3649	1923	1923	7605	1726	1708
3744	2236	2236	7711	344	318
3580	2102	2102	7651	1947	1914
3654	2082	2082	7596	1933	1922
6785	6844	6844	7766	6811	6795
7579	7666	7666	2586	7748	7738
7480	7568	7568	3500	7693	7679
6624	6760	6760	7785	6739	6723
6770	6819	6819	7751	6806	6790
6625	6761	6761	7786	6740	6724
962	3617	3617	7507	3689	3696
3847	2390	2390	7732	595	591
3777	2206	2206	7724	252	232
3656	1946	1946	7683	2166	2166
3791	1921	1921	7707	2304	2307
1029	3794	3794	7609	3884	3872
1029	3794	3794	7609	3884	3872
7525	7611	7611	2642	7690	7678
3584	2211	2211	7620	1852	1840
985	3749	3749	7570	3828	3821
3629	2094	2094	7568	1871	1846
971	3674	3674	7548	3736	3755
3748	2208	2208	7692	96	20
858	3784	3784	7590	3821	3815
3782	2308	2308	7676	237	240
6782	6832	6832	7773	6820	6804
3594	2228	2228	7562	1856	1842
6813	6866	6866	7784	6827	6811
3636	1912	1912	7603	1710	1692
477	3694	3694	7544	3690	3714
3658	1932	1932	7614	1735	1717
878	3659	3659	7514	3702	3687
7479	7567	7567	3499	7692	7678
7479	7567	7567	3499	7692	7678
7480	7568	7568	3500	7693	7679

7478	7566	7566	3498	7691	7677
6799	6852	6852	7767	6813	6797
3773	6	6	7636	2197	2208
3823	2396	2396	7667	332	314
3671	2090	2090	7612	1907	1882
7546	7644	7644	2624	7662	7651
3784	2338	2338	7678	280	260
3633	2080	2080	7594	1818	1795
3585	1935	1935	7624	1759	1741
6750	6812	6812	7778	6799	6781
984	3675	3675	7548	3756	3773
7479	7567	7567	3500	7692	7678
7478	7566	7566	3498	7691	7677
7477	7556	7556	3461	7609	7595
7480	7568	7568	3501	7693	7679
7480	7568	7568	3501	7693	7679
3764	203	203	7667	2200	2191
3816	2389	2389	7660	325	307
3856	2353	2353	7717	572	568
3583	2205	2205	7618	1855	1856
3564	2205	2205	7603	1822	1834
3582	1930	1930	7621	1754	1736
3586	1936	1936	7625	1760	1742
3773	122	122	7654	2191	2202
3623	2064	2064	7655	1909	1891
3615	2056	2056	7647	1901	1883
3552	2324	2324	7563	1983	1982
3789	2335	2335	7673	285	257
3502	2032	2032	7585	1857	1837
3624	2046	2046	7588	1792	1769
3613	1870	1870	7580	1699	1681
890	3736	3736	7526	3785	3784
6777	6835	6835	7772	6821	6805
474	3691	3691	7541	3688	3712
3742	2234	2234	7708	342	316
7546	7636	7636	6	7697	7693
474	3691	3691	7541	3688	3712
3773	2204	2204	7704	37	105
964	3619	3619	7509	3689	3696
3773	2109	2109	7672	295	287
3728	2202	2202	7682	82	69
3758	2223	2223	7725	315	286
3497	2027	2027	7582	1852	1832
6901	6955	6955	7891	6951	6935
3843	2386	2386	7722	597	595
984	3650	3650	7556	3739	3727
3739	1634	1634	7715	2082	2068
4203	3779	3779	7810	3666	3642
3625	1901	1901	7592	1699	1681
3636	1885	1885	7621	1826	1814
7547	7637	7637	7	7698	7694
3794	2258	2258	7675	289	271
3776	2210	2210	7725	308	290
7520	7605	7605	2563	7677	7665
1000	3723	3723	7559	3814	3802

1004	3646	3646	7554	3714	3721
3645	2073	2073	7606	1746	1730
3770	2324	2324	7662	263	247
7545	7635	7635	5	7696	7692
1009	3658	3658	7513	3735	3757
1009	3658	3658	7513	3735	3757
7511	7596	7596	2554	7668	7656
3614	2055	2055	7646	1900	1882
3624	2048	2048	7590	1796	1773
699	3686	3686	7590	3658	3650
858	3757	3757	7510	3779	3798
3605	1862	1862	7572	1691	1673
3502	2032	2032	7587	1857	1837
3754	2220	2220	7713	334	311
3678	2020	2020	7725	2317	2320
7554	7697	7697	3374	7771	7757
3834	2290	2290	7688	444	426
7545	7635	7635	5	7696	7692
7544	7634	7634	4	7695	7691
3771	120	120	7652	2189	2200
3629	2100	2100	7577	1832	1832
6740	6823	6823	7782	6810	6789
3773			7636	2197	2208
3773			7636	2197	2208
7546	7636	7636		7697	7693
3766	2197	2197	7697		96
3753	2208	2208	7693	96	
3497	2027	2027	7582	1852	1832
973	3677	3677	7554	3738	3757
990	3720	3720	7565	3811	3799
7546	7644	7644	2626	7662	7651
3580	1928	1928	7619	1752	1734
3630	1905	1905	7626	1844	1824
3653	2081	2081	7595	1932	1921
3629	2070	2070	7659	1915	1897
3606	2141	2141	7589	1765	1748
3604	2139	2139	7587	1763	1746
3635	2170	2170	7616	1794	1777
6794	6842	6842	7793	6846	6830
	3773	3773	7546	3766	3753
3615	2055	2055	7647	1901	1883
3716	1918	1918	7693	2302	2304
7512	7597	7597	2555	7669	7657
7592	7679	7679	2598	7761	7751
901	3704	3704	7575	3701	3723
3632	1927	1927	7590	1665	1647
7670	7684	7684	2601	7758	7747
3774	123	123	7655	2192	2203
3780	129	129	7661	2198	2209
3636	1887	1887	7587	1724	1706
3746	2230	2230	7709	292	266
6855	6880	6880	7786	6853	6837
3644	2120	2120	7598	1832	1845
6764	6808	6808	7742	6789	6779
3737	2247	2247	7681	129	107

3734	2244	2244	7678	126	104
3708	2008	2008	7707	2294	2294
3636	1912	1912	7603	1710	1692
7544	7642	7642	2624	7660	7649
7545	7643	7643	2625	7661	7650
7667	7681	7681	2598	7755	7744
3580	2216	2216	7616	1861	1849

MOD1-EC3584	CDPHFDLB-F1602032-004B	MOD1-EC1638	29618	149438	E123	287552	140183
3401	833	902	7606	3584	3605	3573	3538
1860	3579	3626	7590	1764	34	2030	1964
1833	3585	3658	7601	1809	2068	138	1952
3660	3407	3417	7755	3779	3859	3759	3801
3327	348	251	7543	3472	3509	3501	3501
25010	24941	24927	25499	25015	24974	24966	25042
1442	3582	3613	7607	1598	1829	1819	1937
1789	3539	3574	7613	1849	1977	1963	1960
1844	3597	3655	7608	1937	1968	1957	1978
25009	24940	24926	25498	25014	24973	24965	25041
1825	3749	3794	7649	1725	1825	1902	1886
2245	3712	3763	7767	2136	2048	2261	2250
7576	7581	7583	6	7612	7589	7585	7649
1891	3492	3560	7513	1912	1889	1859	1873
1815	3561	3620	7618	1838	1369	1978	1874
6827	7011	6997	7861	6874	6839	6954	6920
1740	3566	3612	7569	307	1758	1778	1871
2097	3692	3721	7717	2195	2190	2094	2242
2097	3692	3721	7717	2195	2190	2094	2242
1686	3630	3687	7645	1745	2100	1893	1970
3503	4204	4222	7772	3527	3649	3261	3563
2120	3738	3775	7633	1921	1906	2072	2122
7671	7613	7627	2559	7696	7690	7682	7728
1611	3578	3613	7580	1467	1927	1878	1872
1874	3738	3771	7681	1818	1849	2001	1948
1871	3730	3783	7650	1774	1875	1959	1936
7563	7552	7560	2384	7610	7621	7599	7656
1802	3591	3623	7635	1937	2048	1970	189
7636	7682	7681	2517	7695	7706	7642	7695
7636	7682	7681	2517	7695	7706	7642	7695
20	3422	3463	7584	1736	1884	1816	1757
30966	30931	30935	31227	30935	30950	31004	30986
7635	7588	7609	3304	7687	7683	7674	7707
7739	7722	7749	3475	7754	7780	7773	7790
3388	1149	1162	7583	3475	3564	3572	3521
3388	1149	1162	7583	3475	3564	3572	3521
7584	7556	7567	2628	7621	7628	7597	7661
7699	7664	7693	3445	7734	7775	7751	7780
1952	3725	3787	7640	1878	1976	2021	1988
1868	3734	3767	7677	1806	1835	1993	1950
2121	3739	3776	7634	1922	1907	2073	2123
3431	555	514	7539	3583	3636	3626	3652
7548	7490	7461	3444	7593	7599	7584	7621
6688	6778	6816	7751	6743	6708	6819	6761
6676	6798	6802	7726	6729	6678	6817	6742
3426	736	783	7546	3528	3549	3508	3528
2031	3880	3856	7618	1945	2009	2090	2066
6706	6827	6831	7755	6759	6708	6847	6772
2234	3740	3789	7770	2201	2194	2048	2109
7544	7488	7459	3504	7591	7601	7594	7617
6656	6778	6784	7756	6707	6654	6801	6722
7567	7499	7536	3427	7553	7577	7539	7615
2126	3743	3780	7637	1927	1912	2078	2128
2144	3670	3718	7575	2058	2094	2074	2131

1788	3542	3571	7611	1850	1981	1970	1961
2003	3529	3596	7529	1930	1937	1905	1936
1700	3620	3664	7615	366	1799	1766	1902
1736	3559	3595	7636	1878	1969	1924	41
7700	7665	7690	3466	7715	7748	7730	7750
7560	7536	7547	2654	7597	7602	7571	7635
1813	3586	3637	7584	1842	1967	397	1891
1774	3530	3565	7616	1838	1951	1944	1945
1818	3562	3576	7569	1892	1993	1984	1969
1801	3506	3555	7624	1819	1982	1969	1970
1813	3586	3637	7586	1842	1967	397	1891
6679	6785	6798	7739	6740	6686	6822	6745
7600	7527	7556	3361	7652	7652	7637	7680
1747	3568	3604	7647	1887	1980	1931	50
2117	3737	3772	7632	1918	1903	2069	2119
7566	7498	7535	3424	7552	7576	7538	7614
7567	7499	7536	3425	7553	7577	7539	7615
2278	3712	3763	7760	2149	2048	2296	2259
1860	3579	3626	7590	1764	32	2030	1964
1756	3549	3597	7647	1898	1993	1950	73
7564	7566	7566	2344	7609	7624	7600	7654
2136	3746	3783	7635	1911	1896	2088	2136
2119	3766	3854	7555	2012	2100	2140	2120
7705	7672	7699	3473	7720	7753	7735	7755
7633	7679	7678	2517	7692	7704	7639	7692
6708	6780	6843	7653	6754	6725	6832	6785
1750	3528	3572	7614	1822	1933	1933	1942
1811	3611	3648	7605	1786	2059	117	1926
7638	7591	7612	3304	7690	7686	7677	7710
7570	7502	7539	3428	7556	7580	7542	7618
7644	7595	7616	3292	7696	7692	7683	7716
7643	7594	7615	3291	7695	7691	7682	7715
7647	7598	7619	3295	7699	7695	7686	7719
7635	7587	7608	3303	7687	7683	7674	7707
2138	3681	3714	7714	2179	2225	2111	2307
7577	7582	7584	7	7613	7590	7586	7650
2061	3810	3855	7681	1986	1977	2140	2097
7605	7607	7602	2306	7651	7677	7646	7699
2109	3744	3785	7641	1924	1913	2081	2115
1826	3750	3794	7652	1726	1831	1914	1889
1889	3738	3805	7642	1793	1893	1983	1958
1813	3763	3823	7692	1791	1838	1952	1929
1816	3611	3647	7605	1791	2054	110	1929
1836	3771	3829	7696	1804	1851	1973	1948
7647	7598	7619	3295	7699	7695	7686	7719
6588	6645	6649	7769	6647	6610	6739	6674
6680	6804	6808	7728	6731	6676	6821	6744
1841	3744	3770	7645	1743	1825	1918	1906
3395	322	452	7571	3532	3563	3547	3555
1904	3504	3572	7523	1925	1902	1870	1886
3397	873	947	7581	3535	3607	3550	3578
7633	7586	7607	3302	7685	7681	7672	7705
3522	503	413	7592	3608	3644	3638	3632
6822	7006	6992	7856	6869	6834	6949	6915
1825	3756	3795	7635	1667	1791	1815	1810

7639	7593	7614	3303	7691	7687	7678	7713
1831	3766	3824	7694	1799	1846	1968	1943
1899	3757	3791	7664	1881	1882	1998	1950
3438	302	470	7537	3582	3590	3609	3600
6789	6970	6950	7834	6840	6801	6913	6878
7567	7499	7536	3427	7553	7577	7539	7615
7650	7601	7622	3298	7702	7698	7689	7722
7571	7573	7573	2346	7614	7629	7607	7659
1875	3737	3778	7681	1815	1844	1994	1957
1770	3620	3644	7591	1692	1831	708	1891
7700	7665	7690	3466	7715	7748	7730	7750
2100	3746	3782	7698	2072	1971	2145	2080
1829	3739	3774	7685	1797	1850	1972	1947
1898	3761	3817	7656	1802	1902	1995	1967
6585	6642	6646	7766	6644	6607	6736	6671
3430	435	341	7571	3566	3609	3612	3607
7607	7609	7604	2308	7653	7679	7648	7701
1891	3740	3807	7644	1795	1895	1985	1960
2127	3715	3754	7624	1936	1901	2083	2095
1811	3611	3648	7607	1786	2059	117	1926
1691	3611	3656	7606	357	1790	1757	1893
1868	3734	3767	7677	1806	1835	1993	1950
1782	3527	3585	7634	1919	1992	1967	91
1811	3592	3628	7588	1804	2051	17	1938
6682	6804	6808	7732	6735	6684	6823	6748
7611	7613	7608	2313	7657	7683	7652	7705
7569	7501	7538	3427	7555	7579	7541	7617
6586	6643	6647	7767	6645	6608	6737	6672
6659	6789	6793	7717	6712	6661	6804	6725
6587	6644	6648	7768	6646	6609	6738	6673
3359	144	428	7544	3493	3526	3530	3519
2077	3826	3869	7696	2002	1995	2158	2113
1806	3764	3803	7687	1776	1809	1943	1920
2050	3649	3678	7653	2020	2072	1965	2205
2077	3783	3812	7706	2058	2165	2161	2208
3537	590	191	7618	3688	3702	3698	3678
3537	590	191	7618	3688	3702	3698	3678
7561	7563	7563	2341	7606	7621	7597	7651
1762	3541	3582	7614	1822	1955	1946	1931
3478	483	402	7595	3606	3649	3653	3660
1883	3584	3607	7566	1776	2065	506	1880
3412	60	440	7579	3546	3583	3588	3572
1831	3754	3794	7648	1733	1823	1920	1897
3506	1292	1253	7608	3594	3699	3681	3657
1958	3726	3817	7632	1875	1959	2043	2023
6681	6777	6815	7746	6736	6701	6812	6754
1903	3502	3570	7526	1924	1901	1871	1885
6700	6832	6836	7750	6759	6708	6839	6772
1734	3583	3629	7612	544	1761	1821	1884
3436	792	880	7560	3536	3585	3565	3556
1700	3620	3665	7615	366	1799	1766	1902
3403	490	611	7538	3485	3543	3513	3521
7568	7500	7537	3426	7554	7578	7540	7616
7568	7500	7537	3426	7554	7578	7540	7616
7569	7501	7538	3427	7555	7579	7541	7617

7567	7499	7536	3425	7553	7577	7539	7615
6686	6818	6822	7735	6745	6694	6825	6758
2027	3677	3720	7644	1928	1905	2081	2070
2022	3871	3847	7609	1936	2000	2081	2057
1809	3609	3646	7605	1784	2057	115	1924
7578	7583	7585	41	7614	7590	7587	7651
1962	3740	3792	7644	1890	1986	2035	2000
1758	3592	3628	7586	1756	1979	494	1824
1737	3554	3600	7619	21	1787	1808	1900
6652	6769	6772	7747	6705	6650	6797	6714
3435	165	474	7583	3565	3588	3608	3592
7568	7500	7537	3427	7554	7578	7540	7616
7567	7499	7536	3425	7553	7577	7539	7615
7502	7476	7562	3383	7541	7546	7557	7606
7569	7501	7538	3428	7555	7579	7541	7617
7569	7501	7538	3428	7555	7579	7541	7617
2168	3730	3767	7645	1983	1900	2144	2160
2015	3864	3840	7602	1929	1993	2074	2050
2032	3835	3878	7672	1977	1970	2103	2082
1780	3533	3580	7613	1840	1961	1956	1951
1766	3504	3561	7599	1824	1941	1933	1933
1732	3551	3597	7616	16	1782	1805	1895
1738	3555	3601	7620	22	1788	1809	1901
2121	3739	3776	7634	1922	1907	2073	2123
1745	3568	3604	7645	1887	1978	1931	48
1737	3560	3596	7637	1879	1970	1923	40
1864	3544	3580	7568	1966	2067	2072	2043
1959	3745	3797	7639	1887	1983	2032	1997
19	3421	3462	7581	1735	1883	1815	1756
1730	3592	3628	7576	1732	1949	530	1814
1703	3582	3628	7586	254	1719	1754	1864
3496	560	591	7556	3596	3624	3607	3605
6682	6772	6810	7745	6737	6702	6813	6755
3433	789	877	7558	3533	3582	3562	3554
1866	3732	3765	7674	1804	1833	1991	1948
7582	7554	7565	2626	7619	7626	7595	7659
3433	789	877	7558	3533	3582	3562	3554
1859	3745	3818	7669	1761	1853	1939	1922
3359	146	430	7546	3493	3524	3530	3517
1825	3756	3795	7635	1667	1791	1815	1810
1828	3726	3793	7634	1729	1829	1916	1893
1851	3764	3814	7673	1815	1866	1954	1938
14	3416	3457	7578	1730	1878	1810	1751
6802	6942	6845	7871	6858	6821	6944	6890
2066	3812	3857	7688	2002	1992	2146	2106
3376	415	283	7565	3523	3550	3548	3542
2083	3750	3800	7690	2005	1907	2057	2043
3492	4193	4211	7761	3516	3638	3250	3552
1723	3572	3618	7602	533	1750	1810	1873
1846	3582	3635	7587	1742	98	2034	1970
7583	7555	7566	2627	7620	7627	7596	7660
1908	3783	3821	7645	1800	1882	1931	1965
1846	3764	3807	7685	1790	1823	1967	1922
7553	7544	7552	2314	7588	7609	7581	7633
3461	442	40	7580	3598	3639	3629	3613

3372	202	445	7597	3504	3558	3542	3535
1766	3621	3654	7592	1686	1827	698	1885
1949	3721	3778	7628	1877	1973	2022	1987
7581	7553	7564	2625	7618	7625	7594	7658
3398	143	451	7552	3529	3578	3569	3566
3398	143	451	7552	3529	3578	3569	3566
7544	7535	7543	2305	7579	7600	7572	7624
1736	3559	3595	7636	1878	1969	1922	39
1734	3592	3628	7578	1734	1951	532	1816
3382	1152	1168	7589	3467	3572	3580	3519
3513	395	606	7530	3599	3622	3633	3625
1695	3574	3620	7578	246	1711	1746	1856
21	3421	3462	7583	1735	1883	1815	1756
1843	3741	3806	7662	1802	1831	1959	1933
2100	3669	3703	7719	2151	2148	2091	2276
7633	7586	7607	3299	7685	7681	7672	7705
1928	3818	3862	7646	1852	1907	2058	1993
7581	7553	7564	2625	7618	7625	7594	7658
7580	7552	7563	2624	7617	7624	7593	7657
2119	3737	3774	7632	1920	1905	2071	2121
1790	3559	3597	7570	1816	1995	613	1867
6662	6759	6784	7741	6714	6661	6806	6728
2027	3677	3720	7644	1928	1905	2081	2070
2027	3677	3720	7644	1928	1905	2081	2070
7582	7554	7565	2626	7619	7626	7595	7659
1852	3738	3811	7662	1752	1844	1932	1915
1832	3757	3799	7651	1734	1824	1921	1897
	3416	3457	7578	1730	1878	1810	1751
3416		442	7583	3549	3582	3591	3574
3457	442		7585	3595	3628	3627	3610
7578	7583	7585		7614	7591	7587	7651
1730	3549	3595	7614		1780	1803	1893
1878	3582	3628	7591	1780		2050	1984
1810	3591	3627	7587	1803	2050		1937
1751	3574	3610	7651	1893	1984	1937	
1613	3580	3615	7582	1469	1929	1880	1874
1611	3578	3613	7580	1467	1927	1878	1872
1642	3609	3644	7608	1498	1958	1909	1903
6698	6813	6823	7765	6750	6701	6840	6768
3497	973	990	7546	3580	3630	3653	3629
1736	3560	3596	7637	1876	1970	1923	40
2102	3711	3746	7692	2106	2136	2108	2170
7545	7536	7544	2306	7580	7601	7573	7625
7624	7626	7621	2325	7670	7696	7665	7718
3449	551	583	7599	3549	3590	3585	3570
1676	3583	3631	7607	446	1732	1807	1877
7638	7684	7683	2519	7697	7708	7644	7697
2122	3740	3777	7635	1923	1908	2074	2124
2128	3746	3783	7641	1929	1914	2080	2130
1724	3605	3651	7589	289	1738	1775	1887
1824	3732	3759	7676	1792	1835	1965	1940
6724	6874	6878	7752	6777	6724	6851	6796
1799	3561	3609	7588	1827	2016	628	1878
6668	6758	6796	7704	6706	6679	6784	6731
1869	3728	3781	7648	1773	1873	1957	1934

1866	3725	3778	7645	1770	1870	1954	1931
2108	3697	3726	7699	2148	2193	2085	2217
1734	3583	3629	7612	544	1761	1821	1884
7576	7581	7583	6	7612	7589	7585	7649
7577	7580	7582	7	7613	7590	7586	7650
7635	7681	7680	2516	7694	7705	7641	7694
1773	3529	3564	7615	1837	1950	1943	1944

93272	182138	272422	E2026_9	MOD1-EC3605	MOD1-EC3330	CFSAN044415	MOD1-EC5432
3586	3584	3615	6802	871	3524	3713	7546
1911	1909	1940	6705	3629	1950	2120	7600
1859	1857	1888	6838	3657	1938	2143	7590
3814	3812	3843	6985	3509	3787	3818	7706
3496	3494	3525	6770	904	3487	3621	7508
24980	24978	25006	25028	24880	25035	24967	25516
1657	1655	1686	6730	3635	1923	2044	7602
1864	1862	1893	6704	3584	1946	2377	7606
1819	1817	1848	6799	3643	1965	2326	7598
24979	24977	25005	25027	24879	25034	24966	25515
1737	1735	1766	6833	3751	1872	2291	7655
2147	2145	2176	6873	3714	2236	1609	7731
7580	7578	7606	7763	7544	7635	7690	2304
1795	1793	1824	6682	3582	1859	2285	7511
1864	1862	1893	6808	3601	1860	1902	7601
6946	6944	6972	424	6958	6906	6950	7835
1467	1465	1496	6738	3603	1854	2101	7553
2056	2054	2085	6813	3699	2228	322	7682
2056	2054	2085	6813	3699	2228	322	7682
1840	1838	1869	6840	3708	1955	2174	7652
3564	3562	3593	6940	4214	3549	3688	7758
2110	2108	2139	6837	3772	2107	1908	7603
7669	7667	7696	7824	7588	7714	7783	2536
6	4	35	6814	3604	1858	2057	7575
1844	1842	1873	6860	3752	1934	2291	7688
1782	1780	1811	6836	3739	1922	2353	7654
7602	7600	7629	7706	7522	7644	7687	502
1922	1920	1951	6768	3642	175	2218	7605
7665	7663	7692	7987	7668	7683	7755	2357
7665	7663	7692	7987	7668	7683	7755	2357
1619	1617	1648	6704	3503	1742	2108	7551
30948	30946	30973	30779	30915	30974	30947	31199
7668	7666	7695	7959	7556	7695	7763	3233
7768	7766	7793	8025	7702	7778	7831	3370
3512	3510	3541	6797	692	3507	3640	7562
3512	3510	3541	6797	692	3507	3640	7562
7591	7589	7618	7795	7548	7649	7695	2557
7726	7724	7751	7961	7644	7768	7815	3326
1880	1878	1909	6823	3780	1974	2414	7642
1840	1838	1869	6852	3744	1936	2281	7684
2111	2109	2140	6838	3773	2108	1909	7604
3614	3612	3643	6816	1136	3638	3756	7511
7602	7600	7629	7862	7448	7609	7675	3408
6811	6809	6837	201	6783	6747	6811	7724
6802	6800	6828	225	6779	6728	6797	7719
3535	3533	3564	6771	1007	3514	3678	7520
1959	1957	1988	6846	3832	2052	2465	7620
6832	6830	6858	255	6808	6758	6827	7748
2088	2086	2117	6954	3832	2095	2428	7755
7602	7600	7629	7854	7446	7605	7679	3449
6780	6778	6806	283	6763	6708	6773	7746
7542	7540	7569	7903	7478	7603	7673	3364
2116	2114	2145	6843	3777	2113	1914	7609
1966	1964	1995	6795	3763	2117	2397	7590

1869	1867	1898	6701	3561	1947	2371	7599
1809	1807	1838	6718	3621	1922	2254	7513
1432	1430	1461	6777	3658	1885	2112	7598
1859	1857	1888	6753	3614	27	2155	7612
7735	7733	7760	7978	7647	7738	7793	3344
7565	7563	7592	7819	7528	7623	7675	2586
1829	1827	1858	6807	3645	1877	2094	7583
1855	1853	1884	6686	3581	1931	2364	7607
1908	1906	1937	6648	3562	1955	2378	7558
1886	1884	1915	6682	3518	1956	2326	7610
1829	1827	1858	6809	3645	1877	2094	7585
6801	6799	6827	301	6775	6731	6797	7730
7628	7626	7655	7965	7511	7668	7726	3301
1870	1868	1899	6762	3623	36	2166	7623
2107	2105	2136	6840	3769	2104	1915	7604
7541	7539	7568	7902	7477	7602	7672	3361
7542	7540	7569	7903	7478	7603	7673	3362
2150	2148	2179	6884	3706	2245	1652	7723
1911	1909	1940	6705	3629	1950	2120	7600
1879	1877	1908	6738	3620	59	2175	7625
7601	7599	7628	7721	7528	7642	7673	287
2126	2124	2155	6849	3780	2121	1898	7605
2049	2047	2078	6759	3780	2106	2556	7542
7740	7738	7765	7985	7653	7743	7798	3352
7662	7660	7689	7982	7665	7680	7752	2356
6819	6817	6845	449	6785	6773	6843	7615
1843	1841	1872	6698	3558	1928	2356	7603
1833	1831	1862	6833	3673	1912	2119	7594
7671	7669	7698	7962	7559	7698	7766	3235
7545	7543	7572	7906	7481	7606	7676	3365
7678	7676	7705	7963	7563	7704	7772	3233
7677	7675	7704	7962	7562	7703	7771	3232
7681	7679	7708	7966	7566	7707	7775	3236
7668	7666	7695	7961	7555	7695	7763	3232
2077	2075	2106	6816	3695	2293	353	7687
7581	7579	7607	7764	7545	7636	7691	2305
2007	2005	2036	6794	3831	2083	2425	7703
7647	7645	7674	7755	7573	7687	7714	642
2091	2089	2120	6847	3781	2100	1939	7611
1738	1736	1767	6838	3752	1875	2292	7658
1798	1796	1827	6847	3762	1944	2372	7648
1852	1850	1881	6876	3797	1915	2300	7693
1838	1836	1867	6826	3671	1915	2124	7594
1854	1852	1883	6878	3787	1934	2305	7697
7681	7679	7708	7966	7566	7707	7775	3236
6716	6714	6742	424	6626	6660	6705	7772
6800	6798	6826	223	6785	6730	6795	7721
1749	1747	1778	6831	3736	1892	2291	7651
3562	3560	3591	6782	908	3541	3689	7523
1808	1806	1837	6693	3595	1872	2298	7520
3558	3556	3587	6794	548	3564	3640	7556
7666	7664	7693	7958	7554	7693	7761	3231
3644	3642	3673	6828	857	3618	3737	7548
6941	6939	6967	419	6953	6901	6945	7830
1721	1719	1750	6809	3773	1796	2206	7641

7674	7672	7701	7962	7561	7701	7769	3232
1849	1847	1878	6872	3782	1929	2300	7695
1923	1921	1952	6839	3751	1936	2328	7662
3615	3613	3644	6822	999	3586	3741	7486
6916	6914	6942	432	6913	6864	6916	7814
7542	7540	7569	7905	7478	7603	7673	3362
7684	7682	7711	7969	7569	7710	7778	3239
7608	7606	7635	7729	7535	7647	7680	275
1845	1843	1874	6852	3738	1943	2290	7686
1625	1623	1654	6800	3644	1877	2059	7605
7735	7733	7760	7978	7647	7738	7793	3344
1934	1932	1963	6799	3755	2066	1368	7663
1849	1847	1878	6859	3757	1933	2298	7688
1810	1808	1839	6856	3774	1953	2381	7662
6713	6711	6739	421	6623	6657	6702	7769
3589	3587	3618	6804	969	3593	3716	7527
7649	7647	7676	7757	7575	7689	7716	644
1800	1798	1829	6849	3764	1946	2374	7650
2115	2113	2144	6826	3753	2080	1863	7600
1833	1831	1862	6833	3673	1912	2119	7596
1423	1421	1452	6768	3649	1876	2103	7589
1840	1838	1869	6852	3744	1936	2281	7684
1905	1903	1934	6767	3580	77	2197	7610
1881	1879	1910	6841	3654	1924	2109	7574
6808	6806	6834	231	6785	6734	6803	7725
7653	7651	7680	7761	7579	7693	7720	649
7544	7542	7571	7905	7480	7605	7675	3364
6714	6712	6740	422	6624	6658	6703	7770
6785	6783	6811	209	6770	6711	6780	7710
6715	6713	6741	423	6625	6659	6704	7771
3518	3516	3547	6774	962	3505	3654	7487
2023	2021	2052	6811	3847	2099	2441	7718
1830	1828	1859	6845	3777	1906	2279	7690
1894	1892	1923	6822	3656	2191	534	7633
2150	2148	2179	6785	3791	2194	762	7655
3708	3706	3737	6835	1029	3664	3804	7588
3708	3706	3737	6835	1029	3664	3804	7588
7598	7596	7625	7718	7525	7639	7670	284
1841	1839	1870	6699	3584	1917	2348	7605
3627	3625	3656	6847	985	3646	3778	7542
1747	1745	1776	6804	3629	1866	2083	7555
3576	3574	3605	6811	971	3558	3709	7532
1747	1745	1776	6829	3748	1883	2304	7656
3663	3661	3692	6805	858	3643	3739	7565
1871	1869	1900	6839	3782	2009	2425	7633
6804	6802	6830	198	6782	6740	6804	7719
1807	1805	1836	6694	3594	1871	2297	7523
6824	6822	6850	261	6813	6758	6821	7743
1516	1514	1545	6759	3636	1867	2080	7592
3544	3542	3573	6769	477	3542	3649	7518
1432	1430	1461	6777	3658	1885	2112	7598
3501	3499	3530	6766	878	3507	3633	7497
7543	7541	7570	7904	7479	7604	7674	3363
7543	7541	7570	7904	7479	7604	7674	3363
7544	7542	7571	7905	7480	7605	7675	3364

7542	7540	7569	7903	7478	7603	7673	3362
6810	6808	6836	245	6799	6744	6805	7726
2141	2139	2170	6842	3773	2055	1918	7597
1950	1948	1979	6837	3823	2043	2456	7611
1831	1829	1860	6831	3671	1910	2117	7594
7582	7580	7608	7765	7546	7637	7692	2302
1890	1888	1919	6832	3784	1986	2425	7646
1804	1802	1833	6782	3633	1810	2029	7591
1474	1472	1503	6760	3585	1883	2113	7587
6772	6770	6798	279	6750	6700	6767	7737
3597	3595	3626	6803	984	3578	3719	7535
7543	7541	7570	7904	7479	7604	7674	3364
7542	7540	7569	7903	7478	7603	7673	3362
7530	7528	7557	7898	7477	7594	7648	3320
7544	7542	7571	7905	7480	7605	7675	3365
7544	7542	7571	7905	7480	7605	7675	3365
2076	2074	2105	6823	3764	2146	1901	7617
1943	1941	1972	6830	3816	2036	2449	7604
1968	1966	1997	6795	3856	2068	2414	7694
1857	1855	1886	6694	3583	1937	2352	7605
1842	1840	1871	6690	3564	1919	2352	7589
1471	1469	1500	6755	3582	1878	2108	7582
1475	1473	1504	6761	3586	1884	2114	7588
2111	2109	2140	6838	3773	2108	1909	7604
1868	1866	1897	6762	3623	34	2164	7621
1860	1858	1889	6754	3615	26	2156	7613
1967	1965	1996	6692	3552	2029	2435	7556
1885	1883	1914	6821	3789	1983	2422	7639
1618	1616	1647	6703	3502	1741	2107	7548
1778	1776	1807	6781	3624	1800	2007	7581
1426	1424	1455	6757	3613	1847	2072	7571
3627	3625	3656	6772	890	3591	3721	7519
6805	6803	6831	195	6777	6741	6805	7718
3541	3539	3570	6768	474	3540	3645	7515
1838	1836	1867	6849	3742	1934	2279	7681
7589	7587	7616	7793	7546	7647	7693	2555
3541	3539	3570	6768	474	3540	3645	7515
1772	1770	1801	6853	3773	1908	2307	7676
3518	3516	3547	6778	964	3503	3654	7489
1721	1719	1750	6809	3773	1796	2206	7641
1743	1741	1772	6832	3728	1879	2298	7638
1881	1879	1910	6852	3758	1924	2308	7678
1613	1611	1642	6698	3497	1736	2102	7545
6926	6924	6952	326	6901	6876	6922	7856
2022	2020	2051	6797	3843	2092	2441	7708
3543	3541	3572	6701	984	3528	3672	7526
1866	1864	1895	6797	3739	2029	1467	7664
3553	3551	3582	6930	4203	3538	3677	7745
1505	1503	1534	6748	3625	1856	2069	7584
1891	1889	1920	6701	3636	1956	2104	7596
7590	7588	7617	7794	7547	7648	7694	2556
1810	1808	1839	6800	3794	1951	2354	7647
1816	1814	1845	6858	3776	1908	2265	7692
7585	7583	7612	7757	7520	7621	7655	20
3618	3616	3647	6811	1000	3599	3749	7539

3537	3535	3566	6783	1004	3521	3684	7545
1621	1619	1650	6798	3645	1871	2055	7606
1877	1875	1906	6816	3770	1973	2411	7630
7588	7586	7615	7792	7545	7646	7692	2554
3560	3558	3589	6785	1009	3552	3695	7510
3560	3558	3589	6785	1009	3552	3695	7510
7576	7574	7603	7748	7511	7612	7646	11
1859	1857	1888	6753	3614	11	2155	7612
1780	1778	1809	6784	3624	1802	2009	7583
3510	3508	3539	6803	699	3505	3638	7570
3637	3635	3666	6826	858	3611	3738	7496
1418	1416	1447	6749	3605	1839	2064	7563
1618	1616	1647	6703	3502	1741	2107	7550
1803	1801	1832	6858	3754	1919	2276	7667
2010	2008	2039	6821	3678	2262	321	7689
7666	7664	7693	7957	7554	7693	7761	3230
1917	1915	1946	6845	3834	1979	2348	7648
7588	7586	7615	7792	7545	7646	7692	2554
7587	7585	7614	7791	7544	7645	7691	2553
2109	2107	2138	6836	3771	2106	1907	7602
1848	1846	1877	6771	3629	1853	2065	7573
6784	6782	6810	290	6740	6714	6779	7731
2141	2139	2170	6842	3773	2055	1918	7597
2141	2139	2170	6842	3773	2055	1918	7597
7589	7587	7616	7793	7546	7647	7693	2555
1765	1763	1794	6846	3766	1901	2302	7669
1748	1746	1777	6830	3753	1883	2304	7657
1613	1611	1642	6698	3497	1736	2102	7545
3580	3578	3609	6813	973	3560	3711	7536
3615	3613	3644	6823	990	3596	3746	7544
7582	7580	7608	7765	7546	7637	7692	2306
1469	1467	1498	6750	3580	1876	2106	7580
1929	1927	1958	6701	3630	1970	2136	7601
1880	1878	1909	6840	3653	1923	2108	7573
1874	1872	1903	6768	3629	40	2170	7625
	6	37	6816	3606	1860	2059	7577
6		35	6814	3604	1858	2057	7575
37	35		6842	3635	1889	2088	7604
6816	6814	6842		6794	6754	6812	7749
3606	3604	3635	6794		3615	3716	7512
1860	1858	1889	6754	3615		2154	7613
2059	2057	2088	6812	3716	2154		7647
7577	7575	7604	7749	7512	7613	7647	
7666	7664	7693	7774	7592	7706	7733	661
3572	3570	3601	6785	901	3556	3650	7563
1514	1512	1543	6742	3632	1860	2108	7587
7667	7665	7694	7989	7670	7685	7757	2359
2112	2110	2141	6839	3774	2109	1910	7605
2118	2116	2147	6845	3780	2115	1916	7611
1457	1455	1486	6772	3636	1870	2089	7580
1844	1842	1873	6850	3746	1926	2293	7679
6848	6846	6874	299	6855	6782	6847	7741
1857	1855	1886	6791	3644	1864	2079	7594
6778	6776	6804	280	6764	6717	6780	7684
1780	1778	1809	6834	3737	1920	2351	7652

1777	1775	1806	6831	3734	1917	2348	7649
2015	2013	2044	6807	3708	2203	301	7672
1516	1514	1545	6759	3636	1867	2080	7592
7580	7578	7606	7763	7544	7635	7690	2304
7581	7579	7607	7764	7545	7636	7691	2305
7664	7662	7691	7984	7667	7682	7754	2356
1854	1852	1883	6685	3580	1930	2363	7606

UMB12_01.1uot	RR1	UMB08_01.1uog	MOD1-EC3102	NC_P10-04	NC_P19-11	NC_STEC173
7634	1017	3618	7694	3762	3768	3636
7693	3574	1716	7704	1888	1894	1722
7685	3628	1813	7671	2106	2112	1781
7795	3411	3761	7807	3836	3842	3791
7586	518	3508	7646	3672	3678	3528
25566	24921	25009	25572	24972	24976	25017
7684	3573	1535	7695	2076	2082	1540
7695	3555	1830	7692	2217	2223	1843
7692	3578	1855	7696	2246	2252	1924
25565	24920	25008	25571	24971	24975	25016
7747	3710	1638	7743	2182	2188	1697
7811	3682	2137	7821	1866	1872	2122
2323	7597	7605	2517	7633	7639	7587
7604	3535	1901	7619	2182	2188	1904
7698	3566	1814	7703	2027	2033	1806
7875	6983	6864	8072	6967	6973	6896
7634	3570	259	7651	1899	1905	112
7768	3645	2179	7789	1977	1983	2158
7768	3645	2179	7789	1977	1983	2158
7728	3666	1770	7733	2263	2269	1735
7844	4164	3589	7897	3808	3814	3567
7698	3703	1924	7698	6	12	1884
2620	7663	7696	2615	7725	7731	7691
7664	3570	1512	7665	2110	2116	1455
7780	3707	1711	7779	2239	2245	1768
7748	3710	1687	7746	2244	2250	1746
822	7577	7610	2469	7628	7634	7599
7698	3572	1888	7677	2157	2163	1931
2468	7700	7687	28	7698	7704	7676
2468	7700	7687	28	7698	7704	7676
7630	3455	1682	7644	2128	2134	1730
31219	30913	30937	31239	30947	30951	30937
3299	7613	7669	3293	7708	7714	7673
3417	7752	7748	3322	7793	7799	7744
7649	1014	3525	7705	3682	3688	3531
7649	1014	3525	7705	3682	3688	3531
2600	7577	7592	2603	7657	7663	7589
3327	7687	7720	3340	7781	7787	7719
7734	3696	1781	7730	2314	2320	1850
7776	3701	1699	7775	2231	2237	1756
7699	3704	1925	7699	7	13	1885
7587	776	3635	7653	3785	3791	3635
3493	7534	7584	3483	7623	7629	7580
7749	6788	6733	7966	6838	6844	6763
7744	6784	6722	7949	6835	6841	6749
7602	694	3562	7650	3686	3692	3585
7712	3816	1852	7756	2400	2406	1915
7773	6813	6752	7978	6865	6871	6779
7857	3784	2196	7850	2452	2458	2183
3517	7532	7582	3526	7627	7633	7578
7768	6764	6700	7976	6813	6819	6727
3434	7533	7567	3415	7575	7581	7539
7704	3708	1930	7704	12	18	1890
7695	3712	2078	7675	2355	2361	2065

7688	3540	1835	7687	2213	2219	1844
7618	3561	1933	7631	2179	2185	1920
7677	3624	337	7701	1925	1931	225
7705	3555	1862	7684	2109	2115	1872
3401	7693	7706	3318	7758	7764	7706
2629	7557	7568	2631	7631	7637	7565
7678	3576	1843	7654	2138	2144	1808
7696	3545	1815	7696	2198	2204	1832
7649	3556	1869	7667	2246	2252	1886
7703	3490	1800	7699	2183	2189	1813
7680	3576	1843	7656	2138	2144	1808
7755	6781	6733	7960	6839	6845	6760
3367	7559	7636	3360	7675	7681	7640
7716	3564	1873	7695	2118	2124	1881
7699	3702	1921	7699	13	19	1881
3431	7532	7566	3412	7574	7580	7538
3432	7533	7567	3413	7575	7581	7539
7810	3682	2142	7818	1919	1925	2127
7693	3574	1716	7704	1888	1894	1722
7718	3545	1882	7697	2137	2143	1892
654	7583	7611	2465	7624	7630	7604
7700	3711	1914	7700	22	28	1874
7630	3809	1929	7655	2459	2465	1980
3409	7700	7711	3326	7763	7769	7711
2467	7697	7684	43	7695	7701	7673
7636	6817	6746	7853	6851	6857	6774
7692	3545	1799	7692	2196	2202	1818
7687	3604	1790	7659	2085	2091	1758
3301	7616	7672	3295	7711	7717	7676
3435	7536	7570	3416	7578	7584	7542
3301	7620	7678	3295	7715	7721	7682
3300	7619	7677	3294	7714	7720	7681
3304	7623	7681	3298	7718	7724	7685
3298	7612	7669	3292	7708	7714	7673
7767	3635	2182	7792	2058	2064	2153
2324	7598	7606	2518	7634	7640	7588
7793	3769	1895	7798	2367	2373	1952
35	7626	7649	2451	7681	7687	7642
7706	3711	1927	7708	49	55	1887
7750	3708	1639	7745	2193	2199	1698
7740	3719	1706	7730	2263	2269	1765
7785	3759	1710	7797	2224	2230	1743
7689	3611	1795	7662	2080	2086	1763
7789	3759	1699	7801	2231	2237	1756
3304	7623	7681	3298	7718	7724	7685
7797	6631	6648	7988	6759	6765	6675
7746	6790	6724	7951	6831	6837	6751
7743	3708	1656	7737	2186	2192	1715
7615	631	3566	7668	3708	3714	3588
7613	3547	1914	7629	2195	2201	1917
7638	800	3571	7696	3710	3716	3589
3297	7611	7667	3291	7706	7712	7671
7630	526	3640	7685	3752	3758	3662
7870	6978	6859	8067	6962	6968	6891
7730	3713	1586	7734	2104	2110	1643

3298	7618	7673	3294	7712	7718	7677
7787	3754	1694	7799	2226	2232	1751
7754	3708	1776	7757	2254	2260	1833
7577	649	3618	7629	3754	3760	3640
7852	6945	6832	8051	6933	6939	6860
3432	7533	7567	3413	7575	7581	7539
3307	7626	7684	3301	7721	7727	7688
644	7590	7616	2467	7633	7639	7609
7778	3706	1708	7784	2224	2230	1765
7697	3610	1673	7679	2084	2090	1648
3401	7693	7706	3318	7758	7764	7706
7745	3705	2086	7776	1679	1685	2042
7780	3706	1692	7782	2228	2234	1749
7754	3742	1715	7744	2272	2278	1774
7794	6628	6645	7985	6756	6762	6672
7605	589	3600	7683	3742	3748	3622
37	7628	7651	2453	7683	7689	7644
7742	3721	1708	7732	2265	2271	1767
7695	3688	1939	7699	79	85	1899
7689	3604	1790	7661	2085	2091	1758
7668	3615	328	7692	1916	1922	216
7776	3701	1699	7775	2231	2237	1756
7705	3561	1903	7700	2156	2162	1913
7666	3586	1808	7645	2075	2081	1776
7750	6790	6728	7955	6841	6847	6755
42	7632	7655	2458	7687	7693	7648
3434	7535	7569	3415	7577	7583	7541
7795	6629	6646	7986	6757	6763	6673
7735	6775	6705	7938	6816	6822	6732
7796	6630	6647	7987	6758	6764	6674
7579	550	3527	7645	3680	3686	3549
7808	3787	1911	7815	2385	2391	1968
7782	3731	1671	7783	2201	2207	1728
7721	3598	1992	7732	1879	1885	1979
7743	3738	2070	7781	1921	1927	2049
7660	622	3724	7725	3842	3848	3744
7660	622	3724	7725	3842	3848	3744
651	7580	7608	2462	7621	7627	7601
7694	3543	1803	7696	2192	2198	1816
7622	632	3660	7702	3806	3812	3660
7649	3586	1755	7628	2093	2099	1730
7622	549	3580	7682	3737	3743	3602
7750	3718	1646	7746	2203	2209	1705
7653	1064	3644	7720	3785	3791	3658
7727	3718	1778	7740	2303	2309	1847
7744	6787	6726	7961	6829	6835	6756
7616	3545	1913	7632	2194	2200	1916
7768	6818	6752	7973	6863	6869	6779
7673	3583	318	7691	1911	1917	377
7600	703	3574	7672	3695	3701	3590
7677	3624	337	7701	1925	1931	225
7577	462	3521	7636	3660	3666	3539
3433	7534	7568	3414	7576	7582	7540
3433	7534	7568	3414	7576	7582	7540
3434	7535	7569	3415	7577	7583	7541

3432	7533	7567	3413	7575	7581	7539
7751	6804	6738	7956	6849	6855	6765
7679	3704	1927	7684	123	129	1887
7703	3807	1843	7747	2391	2397	1906
7687	3602	1788	7659	2083	2089	1756
2322	7599	7607	2519	7635	7641	7589
7738	3710	1793	7731	2333	2339	1862
7684	3586	1763	7664	2081	2087	1738
7677	3554	453	7702	1930	1936	296
7761	6758	6698	7970	6809	6815	6725
7625	577	3603	7674	3738	3744	3621
3434	7534	7568	3415	7576	7582	7540
3432	7533	7567	3413	7575	7581	7539
3390	7513	7523	3377	7565	7571	7527
3435	7535	7569	3416	7577	7583	7541
3435	7535	7569	3416	7577	7583	7541
7711	3705	1984	7710	94	100	1944
7696	3800	1836	7740	2384	2390	1899
7784	3796	1886	7793	2348	2354	1943
7696	3544	1821	7697	2186	2192	1834
7680	3517	1805	7683	2186	2192	1818
7672	3551	448	7699	1925	1931	291
7678	3555	454	7703	1931	1937	297
7699	3704	1925	7699	7	13	1885
7714	3564	1871	7693	2118	2124	1881
7706	3556	1863	7685	2110	2116	1873
7643	3519	1923	7643	2299	2305	1942
7731	3715	1790	7726	2330	2336	1859
7627	3454	1681	7641	2127	2133	1729
7674	3586	1743	7656	2047	2053	1720
7652	3580	226	7667	1869	1875	59
7599	493	3628	7662	3737	3743	3650
7743	6782	6727	7960	6832	6838	6757
7597	700	3571	7670	3692	3698	3587
7773	3699	1697	7772	2229	2235	1754
2598	7575	7590	2601	7655	7661	7587
7597	700	3571	7670	3692	3698	3587
7768	3708	1674	7763	2199	2205	1733
7581	552	3527	7645	3682	3688	3549
7730	3713	1586	7734	2104	2110	1643
7730	3713	1642	7742	2197	2203	1701
7770	3746	1702	7782	2218	2224	1767
7624	3449	1676	7638	2122	2128	1724
7882	6922	6848	8075	6952	6958	6880
7798	3771	1903	7805	2381	2387	1968
7602	554	3559	7670	3707	3713	3579
7747	3687	2015	7778	1593	1599	1973
7831	4153	3578	7884	3797	3803	3556
7665	3572	307	7681	1900	1906	366
7689	3577	1700	7700	1888	1894	1706
2599	7576	7591	2602	7656	7662	7588
7737	3740	1727	7748	2253	2259	1770
7784	3727	1683	7785	2205	2211	1740
669	7571	7595	2367	7613	7619	7588
7617	583	3634	7679	3780	3786	3654

7635	567	3539	7682	3709	3715	3561
7698	3608	1667	7683	2074	2080	1642
7722	3691	1780	7715	2319	2325	1849
2597	7574	7589	2600	7654	7660	7586
7598	565	3563	7639	3721	3727	3585
7598	565	3563	7639	3721	3727	3585
660	7562	7586	2358	7604	7610	7579
7705	3555	1862	7684	2109	2115	1872
7676	3586	1745	7658	2049	2055	1722
7657	1006	3519	7709	3687	3693	3525
7579	533	3633	7652	3758	3764	3655
7644	3572	218	7659	1861	1867	51
7629	3454	1681	7643	2127	2133	1729
7759	3732	1695	7776	2215	2221	1752
7775	3623	2146	7799	2012	2018	2125
3296	7611	7667	3290	7706	7712	7671
7735	3772	1745	7749	2285	2291	1802
2597	7574	7589	2600	7654	7660	7586
2596	7573	7588	2599	7653	7659	7585
7697	3702	1923	7697	5	11	1883
7666	3548	1817	7647	2101	2107	1804
7755	6768	6707	7978	6820	6826	6734
7679	3704	1927	7684	123	129	1887
7679	3704	1927	7684	123	129	1887
2598	7575	7590	2601	7655	7661	7587
7761	3701	1665	7758	2192	2198	1724
7751	3723	1647	7747	2203	2209	1706
7624	3449	1676	7638	2122	2128	1724
7626	551	3583	7684	3740	3746	3605
7621	583	3631	7683	3777	3783	3651
2325	7599	7607	2519	7635	7641	7589
7670	3549	446	7697	1923	1929	289
7696	3590	1732	7708	1908	1914	1738
7665	3585	1807	7644	2074	2080	1775
7718	3570	1877	7697	2124	2130	1887
7666	3572	1514	7667	2112	2118	1457
7664	3570	1512	7665	2110	2116	1455
7693	3601	1543	7694	2141	2147	1486
7774	6785	6742	7989	6839	6845	6772
7592	901	3632	7670	3774	3780	3636
7706	3556	1860	7685	2109	2115	1870
7733	3650	2108	7757	1910	1916	2089
661	7563	7587	2359	7605	7611	7580
	7645	7668	2470	7700	7706	7661
7645		3583	7702	3705	3711	3603
7668	3583		7689	1926	1932	263
2470	7702	7689		7700	7706	7678
7700	3705	1926	7700		14	1886
7706	3711	1932	7706	14		1892
7661	3603	263	7678	1886	1892	
7771	3699	1687	7773	2225	2231	1744
7766	6860	6770	7973	6877	6883	6797
7687	3544	1828	7667	2121	2127	1815
7705	6768	6698	7923	6805	6811	6726
7746	3708	1686	7744	2242	2248	1745

7743	3705	1683	7741	2239	2245	1742
7760	3648	2128	7775	2000	2006	2109
7673	3583	318	7691	1911	1917	377
2323	7597	7605	2517	7633	7639	7587
2324	7596	7606	2518	7634	7640	7588
2467	7699	7686	23	7697	7703	7675
7695	3544	1812	7695	2197	2203	1831

NC_STEC228	CFSAN061770	MS7925	OH-17-6342	FSIS1703155	KPPUTH06	NC_STEC242
3731	6870	3577	6769	3740	3737	3707
1833	6728	2006	6683	1869	1866	2181
1970	6847	585	6782	1934	1931	2092
3896	7088	3820	6962	3922	3919	3848
3654	6829	3503	6720	3668	3665	3605
25043	25028	24981	25010	25034	25033	24969
1829	6768	1779	6690	1907	1904	2050
1920	6726	1881	6650	1864	1861	2327
1992	6836	1962	6767	1972	1969	2386
25042	25027	24980	25009	25033	25032	24968
255	6840	1836	6776	112	109	2283
2349	6909	2226	6846	2313	2310	1709
7674	7750	7586	7702	7646	7643	7697
1957	6733	1852	6652	1863	1860	2267
1974	6855	1901	6761	1935	1932	1957
6987	378	6920	535	6973	6970	6945
1746	6772	1795	6705	1737	1734	2121
2326	6848	2060	6781	2396	2393	151
2326	6848	2060	6781	2396	2393	151
1960	6894	1804	6796	1925	1922	2161
3644	6948	3376	6907	3677	3674	3688
2223	6875	2119	6803	2240	2237	1998
7793	7808	7709	7754	7767	7764	7791
1842	6846	1855	6776	1778	1775	2013
122	6867	1955	6803	317	314	2299
261	6843	1873	6779	14	11	2337
7711	7698	7625	7641	7685	7682	7712
1946	6831	1906	6745	1940	1937	2265
7771	7971	7665	7921	7742	7739	7773
7771	7971	7665	7921	7742	7739	7773
1830	6730	1805	6674	1875	1872	2114
30994	30804	31020	30787	30991	30988	30967
7791	7931	7693	7890	7756	7753	7784
7902	8009	7795	7966	7866	7863	7866
3622	6871	3562	6757	3634	3631	3638
3622	6871	3562	6757	3634	3631	3638
7711	7788	7600	7744	7683	7680	7709
7874	7949	7770	7899	7840	7837	7842
436	6834	1873	6760	257	254	2398
118	6861	1951	6795	313	310	2287
2224	6876	2120	6804	2241	2238	1999
3758	6879	3658	6793	3785	3782	3750
7714	7871	7601	7835	7678	7675	7696
6831	257	6773	125	6815	6812	6806
6809	86	6764	286	6793	6790	6792
3646	6825	3506	6729	3660	3657	3660
451	6787	1984	6801	330	327	2451
6839	116	6794	316	6823	6820	6822
2221	6999	2086	6926	2147	2144	2494
7714	7871	7611	7829	7678	7675	7696
6807	154	6748	372	6793	6790	6768
7711	7898	7550	7858	7674	7671	7694
2228	6881	2125	6809	2246	2243	2004
2082	6838	2088	6767	2006	2003	2394

1945	6742	1886	6668	1895	1892	2323
1945	6761	1886	6690	1833	1830	2254
1755	6816	1802	6730	1756	1753	2120
1925	6781	1865	6716	1919	1916	2202
7864	7966	7752	7921	7829	7826	7828
7685	7812	7574	7768	7657	7654	7689
1964	6816	511	6759	1930	1927	2053
1935	6708	1848	6638	1881	1878	2324
1979	6695	1899	6636	1927	1924	2338
1934	6704	1893	6634	1880	1877	2286
1964	6818	511	6761	1930	1927	2053
6796	138	6749	326	6780	6777	6792
7762	7945	7658	7907	7727	7724	7747
1936	6790	1872	6725	1930	1927	2213
2220	6878	2118	6806	2237	2234	2005
7710	7897	7549	7857	7673	7670	7693
7711	7898	7550	7858	7674	7671	7694
2326	6920	2263	6857	2306	2303	1764
1833	6728	2006	6683	1869	1866	2181
1945	6766	1855	6707	1931	1928	2222
7701	7713	7623	7656	7675	7672	7700
2229	6887	2135	6815	2248	2245	1988
682	6780	2021	6719	541	538	2558
7869	7973	7757	7928	7834	7831	7833
7768	7966	7662	7916	7739	7736	7770
6843	533	6781	287	6825	6822	6842
1914	6729	1876	6650	1856	1853	2316
1960	6842	573	6777	1924	1921	2074
7794	7934	7696	7893	7759	7756	7787
7714	7901	7553	7861	7677	7674	7697
7802	7940	7702	7898	7767	7764	7793
7801	7939	7701	7897	7766	7763	7792
7805	7943	7705	7901	7770	7767	7796
7791	7931	7693	7890	7756	7753	7784
2345	6859	2088	6796	2395	2392	214
7675	7751	7587	7703	7647	7644	7698
411	6801	2113	6737	592	589	2443
7752	7747	7668	7686	7727	7724	7741
2204	6885	2129	6811	2229	2226	2029
256	6845	1836	6781	113	110	2284
335	6854	1885	6790	138	135	2356
160	6887	1941	6810	336	333	2304
1959	6835	576	6770	1923	1920	2079
122	6885	1957	6821	298	295	2309
7805	7943	7705	7901	7770	7767	7796
6745	274	6684	493	6729	6726	6722
6807	90	6768	288	6791	6788	6790
263	6840	1831	6776	105	102	2273
3721	6845	3563	6735	3737	3734	3679
1970	6744	1865	6663	1876	1873	2280
3658	6871	3533	6761	3690	3689	3630
7789	7930	7691	7889	7754	7751	7782
3782	6887	3626	6799	3798	3795	3721
6982	373	6915	530	6968	6965	6940
471	6810	1740	6764	340	337	2229

7796	7934	7698	7893	7761	7758	7790
117	6879	1952	6815	293	290	2304
217	6856	1933	6787	382	379	2336
3765	6854	3585	6769	3765	3762	3721
6941	364	6871	498	6927	6924	6911
7711	7900	7550	7860	7674	7671	7694
7808	7946	7708	7904	7773	7770	7799
7706	7721	7630	7664	7680	7677	7707
155	6859	1956	6795	352	349	2300
1798	6805	855	6755	1763	1760	1996
7864	7966	7752	7921	7829	7826	7828
2187	6858	2150	6779	2188	2185	1489
33	6866	1952	6802	270	267	2302
344	6863	1897	6799	161	158	2365
6742	271	6681	490	6726	6723	6719
3756	6860	3628	6781	3784	3781	3702
7754	7749	7670	7688	7729	7726	7743
337	6856	1887	6792	140	137	2358
2234	6874	2118	6804	2237	2234	1953
1960	6842	573	6777	1924	1921	2074
1746	6807	1793	6721	1747	1744	2111
118	6861	1951	6795	313	310	2287
1925	6795	1903	6730	1927	1924	2240
1966	6852	629	6785	1958	1955	2086
6815	92	6770	292	6799	6796	6798
7758	7754	7674	7693	7733	7730	7747
7713	7900	7552	7860	7676	7673	7696
6743	272	6682	491	6727	6724	6720
6810	103	6751	301	6794	6791	6775
6744	273	6683	492	6728	6725	6721
3671	6835	3502	6719	3688	3685	3640
425	6818	2129	6754	606	603	2457
106	6852	1929	6788	279	276	2287
2179	6857	1907	6790	2207	2204	245
2296	6812	2166	6757	2346	2343	710
3832	6890	3683	6810	3854	3851	3784
3832	6890	3683	6810	3854	3851	3784
7698	7710	7620	7653	7672	7669	7697
1929	6721	1864	6651	1873	1870	2309
3797	6894	3631	6796	3813	3810	3762
1922	6813	508	6754	1863	1860	2022
3730	6872	3558	6756	3726	3723	3695
266	6836	1844	6778	107	104	2294
3757	6862	3623	6698	3789	3786	3733
434	6776	1884	6788	245	242	2413
6824	258	6766	122	6808	6805	6799
1969	6745	1864	6664	1875	1872	2279
6831	98	6786	318	6815	6812	6816
1740	6784	1820	6713	1731	1728	2117
3692	6831	3546	6730	3685	3682	3639
1755	6816	1802	6730	1756	1753	2120
3655	6828	3555	6717	3681	3678	3613
7712	7899	7551	7859	7675	7672	7695
7712	7899	7551	7859	7675	7672	7695
7713	7900	7552	7860	7676	7673	7696

7711	7898	7550	7858	7674	7671	7694
6817	82	6772	302	6801	6798	6800
2230	6880	2120	6808	2247	2244	2008
442	6778	1975	6792	321	318	2442
1958	6840	571	6775	1922	1919	2072
7676	7752	7588	7704	7648	7645	7699
440	6843	1885	6769	259	256	2409
1885	6791	190	6734	1831	1828	1973
1799	6785	1832	6714	1780	1777	2153
6795	150	6746	368	6779	6776	6760
3758	6883	3600	6795	3758	3755	3705
7712	7899	7551	7859	7675	7672	7695
7711	7898	7550	7858	7674	7671	7694
7637	7890	7590	7837	7592	7589	7669
7713	7900	7552	7860	7676	7673	7696
7713	7900	7552	7860	7676	7673	7696
2211	6861	2181	6789	2232	2229	1985
435	6771	1968	6785	314	311	2435
430	6802	2092	6738	607	604	2430
1945	6716	1871	6646	1889	1886	2310
1909	6712	1847	6636	1841	1838	2312
1794	6780	1829	6709	1775	1772	2150
1800	6786	1833	6715	1781	1778	2154
2224	6876	2120	6804	2241	2238	1999
1934	6790	1870	6725	1928	1925	2211
1926	6782	1864	6717	1920	1917	2203
2061	6717	1981	6654	2010	2007	2413
445	6832	1890	6763	264	261	2406
1829	6729	1804	6673	1874	1871	2113
1873	6790	142	6735	1805	1802	1955
1719	6782	1794	6711	1720	1717	2092
3750	6829	3612	6732	3772	3769	3699
6825	251	6767	119	6809	6806	6800
3690	6830	3543	6729	3683	3680	3635
116	6858	1949	6792	311	308	2285
7709	7786	7598	7742	7681	7678	7707
3690	6830	3543	6729	3683	3680	3635
301	6860	1839	6796	138	135	2299
3671	6839	3502	6723	3688	3685	3640
471	6810	1740	6764	340	337	2229
259	6839	1837	6775	112	109	2284
173	6863	1944	6799	323	320	2312
1824	6724	1799	6668	1869	1866	2108
6953	441	6897	454	6939	6936	6917
431	6808	2118	6741	612	609	2459
3709	6818	3520	6716	3719	3716	3654
2120	6858	2060	6779	2103	2100	1588
3633	6938	3365	6897	3666	3663	3677
1729	6773	1809	6702	1720	1717	2106
1823	6724	2010	6675	1859	1856	2169
7710	7787	7599	7743	7682	7679	7708
395	6830	1885	6798	278	275	2330
172	6865	1927	6801	337	334	2283
7687	7749	7602	7692	7660	7657	7680
3762	6866	3612	6784	3784	3781	3729

3704	6844	3565	6734	3709	3706	3668
1808	6803	851	6753	1773	1770	2000
427	6827	1868	6753	246	243	2395
7708	7785	7597	7741	7680	7677	7706
3738	6846	3582	6730	3738	3735	3687
3738	6846	3582	6730	3738	3735	3687
7678	7740	7593	7683	7651	7648	7671
1925	6781	1863	6716	1919	1916	2202
1877	6793	144	6738	1809	1806	1957
3630	6877	3562	6763	3644	3641	3640
3771	6858	3606	6775	3773	3770	3720
1711	6774	1786	6703	1712	1709	2084
1829	6729	1804	6673	1874	1871	2113
171	6865	1932	6801	350	347	2288
2311	6856	2053	6789	2361	2358	174
7789	7929	7691	7888	7754	7751	7782
312	6833	2014	6796	477	474	2364
7708	7785	7597	7741	7680	7677	7706
7707	7784	7596	7740	7679	7676	7705
2222	6874	2118	6802	2239	2236	1997
1934	6778	48	6717	1858	1855	2007
6807	161	6752	379	6791	6788	6776
2230	6880	2120	6808	2247	2244	2008
2230	6880	2120	6808	2247	2244	2008
7709	7786	7598	7742	7681	7678	7707
292	6853	1832	6789	129	126	2294
266	6837	1845	6779	107	104	2294
1824	6724	1799	6668	1869	1866	2108
3732	6874	3561	6758	3728	3725	3697
3759	6878	3609	6796	3781	3778	3726
7676	7752	7588	7704	7648	7645	7699
1792	6777	1827	6706	1773	1770	2148
1835	6724	2016	6679	1873	1870	2193
1965	6851	628	6784	1957	1954	2085
1940	6796	1878	6731	1934	1931	2217
1844	6848	1857	6778	1780	1777	2015
1842	6846	1855	6776	1778	1775	2013
1873	6874	1886	6804	1809	1806	2044
6850	299	6791	280	6834	6831	6807
3746	6855	3644	6764	3737	3734	3708
1926	6782	1864	6717	1920	1917	2203
2293	6847	2079	6780	2351	2348	301
7679	7741	7594	7684	7652	7649	7672
7771	7766	7687	7705	7746	7743	7760
3699	6860	3544	6768	3708	3705	3648
1687	6770	1828	6698	1686	1683	2128
7773	7973	7667	7923	7744	7741	7775
2225	6877	2121	6805	2242	2239	2000
2231	6883	2127	6811	2248	2245	2006
1744	6797	1815	6726	1745	1742	2109
	6857	1947	6793	259	256	2297
6857		6798	362	6841	6838	6842
1947	6798		6737	1871	1868	2027
6793	362	6737		6777	6774	6775
259	6841	1871	6777		9	2335

256	6838	1868	6774	9		2332
2297	6842	2027	6775	2335	2332	
1740	6784	1820	6713	1731	1728	2117
7674	7750	7586	7702	7646	7643	7697
7675	7751	7587	7703	7647	7644	7698
7770	7968	7664	7918	7741	7738	7772
1934	6707	1847	6637	1880	1877	2323

AZ-TG98487	KTE187	KTE141	KTE126	UMEA
3604	7604	7605	7691	3555
1745	7588	7589	7701	1942
1831	7599	7600	7668	1939
3784	7753	7754	7804	3835
3520	7541	7540	7643	3460
25028	25497	25498	25570	24987
1515	7605	7606	7692	1768
1874	7611	7612	7689	148
1953	7606	7607	7693	1780
25027	25496	25497	25569	24986
1683	7647	7648	7740	1840
2145	7765	7766	7818	2418
7610	4	5	2514	7613
1929	7511	7512	7616	1808
1847	7616	7617	7700	2036
6881	7859	7860	8067	6849
383	7567	7568	7648	1791
2166	7715	7716	7786	2374
2166	7715	7716	7786	2374
1788	7643	7644	7730	1975
3618	7770	7771	7895	3488
1909	7631	7632	7695	2195
7700	2557	2558	2612	7705
1514	7578	7579	7662	1852
1754	7679	7680	7776	1952
1732	7648	7649	7743	1882
7611	2382	2383	2466	7636
1920	7633	7634	7674	2033
7689	2515	2516	25	7693
7689	2515	2516	25	7693
1740	7582	7583	7641	1779
30936	31225	31226	31237	31012
7686	3302	3303	3290	7712
7764	3473	3474	3319	7794
3531	7581	7582	7702	3465
3531	7581	7582	7702	3465
7605	2626	2627	2600	7618
7742	3443	3444	3337	7753
1812	7638	7639	7727	1933
1742	7675	7676	7772	1940
1910	7632	7633	7696	2196
3633	7537	7536	7650	3619
7596	3442	3443	3480	7625
6748	7749	7750	7961	6667
6736	7724	7725	7944	6665
3564	7544	7543	7647	3490
1885	7616	7617	7753	2021
6766	7753	7754	7973	6695
2214	7768	7769	7847	2152
7594	3502	3503	3523	7625
6714	7754	7755	7971	6647
7558	3425	3426	3412	7606
1915	7635	7636	7701	2201
2079	7573	7574	7672	2058

1881	7609	7610	7684	177
1934	7527	7528	7628	1867
482	7613	7614	7698	1808
1869	7634	7635	7681	1929
7722	3464	3465	3315	7752
7581	2652	2653	2628	7596
1849	7582	7583	7651	1891
1867	7614	7615	7693	5
1911	7567	7568	7664	205
1852	7622	7623	7696	164
1849	7584	7585	7653	1891
6747	7737	7738	7955	6663
7649	3359	3360	3357	7680
1880	7645	7646	7692	1940
1906	7630	7631	7696	2192
7557	3422	3423	3409	7605
7558	3423	3424	3410	7606
2150	7758	7759	7815	2440
1745	7588	7589	7701	1942
1889	7645	7646	7694	1927
7616	2342	2343	2462	7630
1899	7633	7634	7697	2205
1938	7553	7554	7652	2077
7727	3471	3472	3323	7757
7686	2515	2516	40	7690
6755	7651	7652	7848	6683
1853	7612	7613	7689	139
1808	7603	7604	7656	1924
7689	3302	3303	3292	7715
7561	3426	3427	3413	7609
7695	3290	3291	3292	7720
7694	3289	3290	3291	7719
7698	3293	3294	3295	7723
7686	3301	3302	3289	7712
2161	7712	7713	7789	2365
7611	3	4	2515	7614
1938	7679	7680	7795	2137
7654	2304	2305	2448	7676
1912	7639	7640	7705	2198
1684	7650	7651	7742	1852
1751	7640	7641	7727	1910
1729	7690	7691	7794	1913
1813	7603	7604	7659	1911
1752	7694	7695	7798	1942
7698	3293	3294	3295	7723
6662	7767	7768	7983	6591
6738	7726	7727	7946	6669
1701	7643	7644	7734	1852
3570	7569	7568	7665	3529
1942	7521	7522	7626	1821
3577	7579	7580	7693	3497
7684	3300	3301	3288	7710
3646	7590	7589	7682	3617
6876	7854	7855	8062	6844
1627	7633	7634	7731	1798

7690	3301	3302	3291	7716
1747	7692	7693	7796	1937
1805	7662	7663	7754	1954
3623	7535	7534	7626	3585
6845	7832	7833	8046	6797
7558	3425	3426	3410	7606
7701	3296	3297	3298	7726
7621	2344	2345	2464	7635
1751	7679	7680	7781	1947
1725	7589	7590	7676	1853
7722	3464	3465	3315	7752
2063	7696	7697	7773	2270
1745	7683	7684	7779	1949
1760	7654	7655	7741	1919
6659	7764	7765	7980	6588
3602	7569	7568	7680	3573
7656	2306	2307	2450	7678
1753	7642	7643	7729	1912
1928	7622	7623	7696	2208
1808	7605	7606	7658	1924
473	7604	7605	7689	1799
1742	7675	7676	7772	1940
1910	7632	7633	7697	1960
1822	7586	7587	7642	1944
6742	7730	7731	7950	6671
7660	2311	2312	2455	7682
7560	3425	3426	3412	7608
6660	7765	7766	7981	6589
6719	7715	7716	7933	6648
6661	7766	7767	7982	6590
3527	7542	7541	7642	3470
1954	7694	7695	7812	2155
1724	7685	7686	7780	1908
1973	7651	7652	7729	2242
2051	7704	7705	7779	2362
3722	7616	7615	7722	3640
3722	7616	7615	7722	3640
7613	2339	2340	2459	7627
1855	7612	7613	7693	117
3674	7593	7592	7699	3615
1763	7564	7565	7625	1948
3580	7577	7576	7679	3527
1691	7646	7647	7743	1848
3650	7606	7607	7717	3587
1823	7630	7631	7737	1932
6741	7744	7745	7956	6660
1941	7524	7525	7629	1820
6766	7748	7749	7968	6687
	7610	7611	7688	1864
3568	7558	7559	7669	3480
482	7613	7614	7698	1808
3525	7536	7535	7633	3518
7559	3424	3425	3411	7607
7559	3424	3425	3411	7607
7560	3425	3426	3412	7608

7558	3423	3424	3410	7606
6752	7733	7734	7951	6673
1912	7642	7643	7681	2216
1876	7607	7608	7744	2012
1806	7603	7604	7656	1922
7612	39	40	2516	7615
1824	7642	7643	7728	1945
1757	7584	7585	7661	1875
551	7617	7618	7699	1844
6712	7745	7746	7965	6639
3603	7581	7580	7671	3570
7559	3425	3426	3412	7607
7558	3423	3424	3410	7606
7549	3381	3382	3374	7576
7560	3426	3427	3413	7608
7560	3426	3427	3413	7608
1969	7643	7644	7707	2217
1869	7600	7601	7737	2005
1929	7670	7671	7790	2130
1865	7611	7612	7694	129
1857	7597	7598	7680	103
546	7614	7615	7696	1839
552	7618	7619	7700	1845
1910	7632	7633	7696	2196
1878	7643	7644	7690	1938
1870	7635	7636	7682	1930
1968	7566	7567	7640	422
1821	7637	7638	7723	1940
1739	7579	7580	7638	1778
1737	7574	7575	7653	1840
340	7584	7585	7664	1800
3632	7554	7553	7659	3604
6742	7743	7744	7955	6661
3564	7556	7557	7667	3478
1740	7672	7673	7769	1938
7603	2624	2625	2598	7616
3564	7556	7557	7667	3478
1719	7667	7668	7760	1870
3527	7544	7543	7642	3470
1627	7633	7634	7731	1798
1687	7632	7633	7739	1856
1755	7671	7672	7779	1919
1734	7576	7577	7635	1773
6863	7869	7870	8070	6819
1946	7686	7687	7802	2141
3571	7563	7562	7667	3484
1978	7688	7689	7775	2232
3607	7759	7760	7882	3477
27	7600	7601	7678	1853
1729	7585	7586	7697	1943
7604	2625	2626	2599	7617
1754	7643	7644	7745	1939
1726	7683	7684	7782	1916
7600	2312	2313	2364	7614
3632	7578	7577	7676	3575

3539	7595	7594	7679	3496
1719	7590	7591	7680	1843
1811	7626	7627	7712	1932
7602	2623	2624	2597	7615
3563	7550	7549	7636	3524
3563	7550	7549	7636	3524
7591	2303	2304	2355	7605
1869	7634	7635	7681	1929
1739	7576	7577	7655	1844
3525	7587	7588	7706	3477
3638	7528	7527	7649	3610
332	7576	7577	7656	1792
1739	7581	7582	7640	1778
1738	7660	7661	7773	1934
2133	7717	7718	7796	2320
7684	3297	3298	3287	7710
1788	7644	7645	7746	2015
7602	2623	2624	2597	7615
7601	2622	2623	2596	7614
1908	7630	7631	7694	2194
1809	7568	7569	7644	1834
6721	7739	7740	7973	6652
1912	7642	7643	7681	2216
1912	7642	7643	7681	2216
7603	2624	2625	2598	7616
1710	7660	7661	7755	1861
1692	7649	7650	7744	1849
1734	7576	7577	7635	1773
3583	7581	7580	7681	3529
3629	7583	7582	7680	3564
7612	6	7	2516	7615
544	7612	7613	7694	1837
1761	7589	7590	7705	1950
1821	7585	7586	7641	1943
1884	7649	7650	7694	1944
1516	7580	7581	7664	1854
1514	7578	7579	7662	1852
1545	7606	7607	7691	1883
6759	7763	7764	7984	6685
3636	7544	7545	7667	3580
1867	7635	7636	7682	1930
2080	7690	7691	7754	2363
7592	2304	2305	2356	7606
7673	2323	2324	2467	7695
3583	7597	7596	7699	3544
318	7605	7606	7686	1812
7691	2517	2518	23	7695
1911	7633	7634	7697	2197
1917	7639	7640	7703	2203
377	7587	7588	7675	1831
1740	7674	7675	7770	1934
6784	7750	7751	7968	6707
1820	7586	7587	7664	1847
6713	7702	7703	7918	6637
1731	7646	7647	7741	1880

1728	7643	7644	7738	1877
2117	7697	7698	7772	2323
	7610	7611	7688	1864
7610		1	2514	7613
7611	1		2515	7614
7688	2514	2515		7692
1864	7613	7614	7692	
